# iTRAQ-Based Quantitative Proteomic Analysis of the Inhibitory Effects of Polysaccharides from *Viscum coloratum* (*Kom*.) *Nakai* on HepG2 Cells

**DOI:** 10.1038/s41598-017-04417-x

**Published:** 2017-07-04

**Authors:** Yangyang Chai, Min Zhao

**Affiliations:** Northeast Forestry University, Harbin, PR China

## Abstract

*Viscum coloratum* (Kom.) *Nakai* is one of active medicinal plants, and its active components, especially polysaccharides, have been shown to exhibit bioactivity. In this study, we examined the effects of three polysaccharide fractions from *Viscum coloratum* (Kom.) *Nakai* on HepG2 cell growth in a dose-dependent manner by using a CCK-8 assay kit. Flow cytometry analysis showed that VCP2 treatment delayed the cell cycle in the G1 phase and induced apoptosis in HepG2 cells, a result possibly due to the increased expression of p21^Wafl/Cip1^ and Cyclin D and the decreased expression of Cyclin E and CDK4. The increased expression of Bad, Smac and Caspase-3 and the decreased expression of Bcl-XL and XIAP may be some of the reasons for the induction of apoptosis in VCP2-treated HepG2 cells. Through iTRAQ and 2D-LC-MSMS, 113 and 198 differentially expressed proteins were identified in normal and VCP2-treated HepG2 and Caco2 cells. The mRNA and protein levels of Histone H3.1, Cytoskeletal 9 and Vitronectin agreed with iTRAQ proteomic results. GO, pathways and the PPI of differentially expressed proteins were further analyzed. These findings broaden the understanding of the anti-tumor mechanisms of mistletoe polysaccharides and provide new clues for screening proteins that are responsive to polysaccharides.

## Introduction

Hepatocellular carcinoma (HCC) is the third leading cause of cancer-related death^1^, and more than half a million new patients worldwide are diagnosed with HCC each year^2^. HCC is induced by liver cirrhosis due to viral infection or the excessive use of alcohol and aflatoxin^3^. HCC develops as a result of a complex process of multi-factor, -stage and -gene interactions; thus, it is necessary to select potent tumor markers to monitor and diagnose HCC. For decades, the detection of serum α-fetoprotein (AFP) (gi|178236) has been the most commonly used tumor marker for HCC^4^; in addition, high expression levels of des-gamma-carboxy prothrombin (DCP)^5^ (gi|23238214), Golgi protein 73 (GP73)^6^ (gi|7271867) and cytokeratin 7 (CK7)^7^ (gi|67782365) have also been used as tumor markers of HCC. However, new tumor markers remain to be developed to provide detection and diagnostic information for HCC.

Mistletoe is an evergreen semiparasitic shrub that is located on the upper branches and trunks of *Quercus*, *Betula*, *Populus* and *Pinus*. There are approximately 1,500 species of mistletoe that have been identified worldwide^8^, but *Viscum coloratum* (Kom.) *Nakai* is the only species included in the Pharmacopoeia of the People’s Republic of China. Mistletoe exerts different types of bioactivities, such as anti-tumor^9^, anti-virus^10^, anti-oxidant^11^, and immunoregulatory functions^12^. As a natural anti-tumor agent, mistletoe and the active components of mistletoe have received attention for their anti-tumor activity. Studies on the anti-tumor activity of mistletoe components have mainly focused on alkaloid and lectin^13, 14^. Mistletoe extracts have anti-tumor activity toward several tumor cell types and inhibit cellular proliferation and induce apoptosis in cancers such as colorectal cancers^15^, lymphoblastic leukemia^16, 17^, multiple myeloma^18^, Ehrlich ascites carcinoma^19^. However, the inhibitory effects of polysaccharides extracted from *Viscum coloratum* (Kom.) *Nakai* on the proteins that are responsive to polysaccharides in HepG2 cells (a hepatocellular carcinoma cell line) have not been investigated. A proteomic study could identify proteins as tumor markers that might be used in the early diagnosis and detection of cancer, and could potentially uncover the molecular mechanisms of cancer development. Isobaric tags for relative and absolute quantitation (iTRAQ) is a quantitative proteomic technology of labeling *in vitro* that was developed by ABI Co. iTRAQ is widely used because of its high throughput, high resolution, accurate protein quantification, repeatability and generation of abundant data.

In this study, iTRAQ was combined with two-dimensional liquid chromatography-tandem mass spectrometry (2D-LC-MSMS) to identify differentially expressed proteins in HepG2 cells induced by treatment with polysaccharides extracted from *Viscum coloratum* (Kom.) *Nakai*. These differentially expressed proteins were analyzed by using bioinformatic techniques, including Gene Ontology (GO) analysis, pathway enrichment and Protein-Protein Interaction (PPI) analysis. We investigated the effects of cell proliferation, cell cycle and apoptosis and the expression of several cell cycle-associated or apoptosis-associated proteins in HepG2 cells induced by exposure to polysaccharides extracted from *Viscum coloratum* (Kom.) *Nakai* to reveal their potent molecular mechanisms. This study should lay a foundation for the subsequent screening of responsive proteins to polysaccharides.

## Results

### The polysaccharide fraction inhibits cell growth and induces apoptosis in HepG2 cells

**Figure 1:**
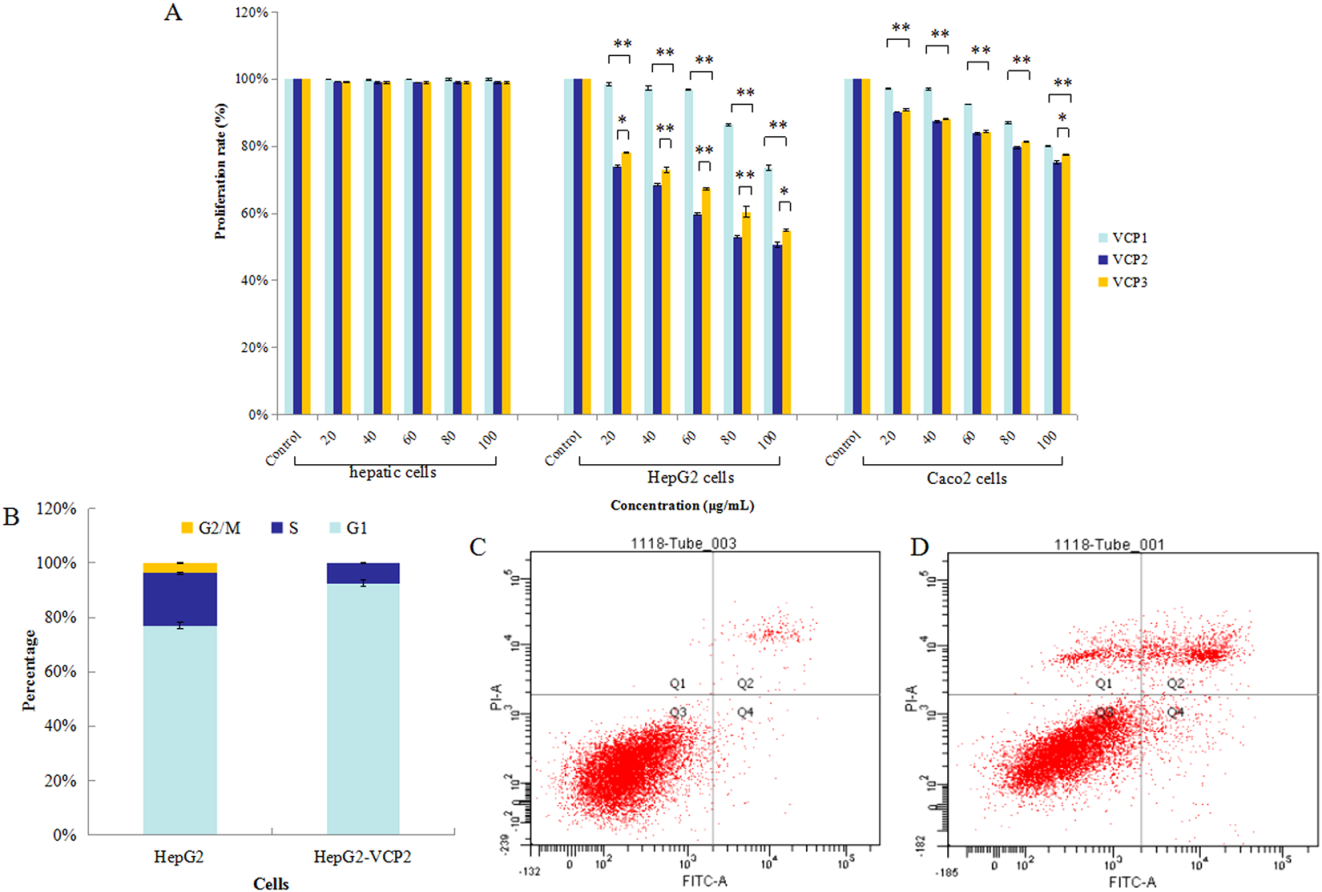
Proliferative inhibited and apoptosis induced by polysaccharides from *Viscum coloratum* (*Kom*.) *Nakai* treatment *in vitro*. (**A**) Proliferative inhibited in polysaccharides-treated hepatic cells, HepG2 cells and Caco2 cells at different concentrations. Cell viability was determined by CCK-8 kit assay. (**B**) Effect of polysaccharides on cell cycle distribution in HepG2 cells. Cell cycle distribution was detected by PI staining with flow cytometry. (**C**) Apoptosis in HepG2 cells. Apoptosis was determined by the flow cytometer based on Annexin V-FITC/PI staining. (**D**) Apoptosis induction in polysaccharides-treated HepG2 cells. Values are means ± SD (n = 3), *p < 0.05 and **p < 0.01.

In this study, the anti-proliferative activity of polysaccharides from *Viscum coloratum* (Kom.) *Nakai* (VCP) against hepatic cells, HepG2 cells and Caco2 cells was examined under different concentrations of VCP1, VCP2 and VCP3 for 48 h *in vitro* by using a Cell Counting Kit-8 (CCK-8) assay (Fig. 1A). All three purified fractions were observed to inhibit HepG2 cell and Caco2 cell proliferation in a dose-dependent manner, and showed the stronger inhibitory ability against HepG2 cells than Caco2 cells (*p* < 0.05). VCP could hardly inhibit hepatic cells proliferation (*p* < 0.05).VCP2 showed the strongest inhibitory ability toward HepG2 cells (*p* < 0.01), and treatment with the 100 μg/mL dose caused nearly a 50% reduction in cell viability, followed by VCP3. VCP1 showed the weakest inhibitory ability toward HepG2 cells, at 80 μg/mL. Therefore, VCP2 was selected for further evaluation.

VCP2 inhibited HepG2 cell proliferation *in vitro*, the growth of HepG2 cells treatment with VCP2 was slower compared with HepG2 cells, and the doubling time was extension with dose-dependent (Supplemental Fig. S1). The cell cycle distribution in normal and VCP2-treated HepG2 cells was examined using propidium iodide (PI) staining and flow cytometry analysis (Fig. 1B). The proportion of HepG2 cells in the G1 phase increased from 77.00 ± 1.30% to 92.42 ± 1.20% after VCP2 treatment. The increase in the G1 cell population led to a decrease in the number of cells in the S and G2/M phases. The proportion of HepG2 cells in S phase decreased sharply from 19.20 ± 0.50% to 7.58 ± 0.30% after VCP2 treatment. The percentage of VCP2-treated HepG2 cells dropped to zero in the G2/M phase. VCP2 treatment therefore increased the G1 cell population, thus suggesting that VCP2 may cause G1 phase arrest. VCP2 treatment induced apoptosis in HepG2 cells, as determined by flow cytometry and Annexin V-FITC/PI staining. VCP2 treatment significantly increased the percentage of apoptotic HepG2 cells compared with that in the control group (Fig. 1C,D). The results of the cell cycle distribution and apoptosis analysis indicated that VCP2 delayed the cell cycle in G1 phase and induced apoptosis in HepG2 cells.

### Quantitative real-time PCR (qRT-PCR) and western blotting (WB) analysis

**Figure 2:**
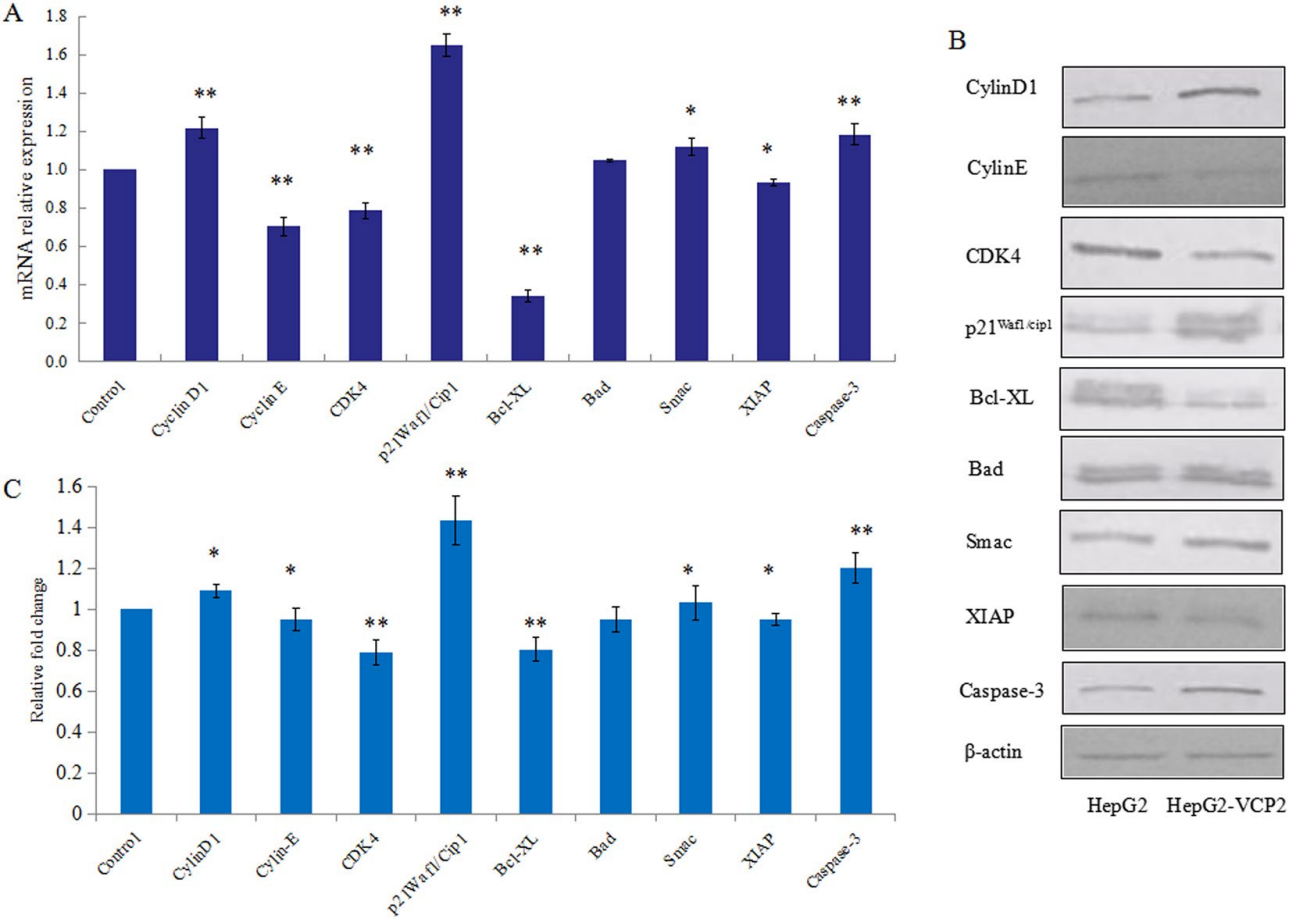
The expression of in HepG2 cells after polysaccharides treatment. (**A**) The mRNA expression in polysaccharides-treated HepG2 cells by qRT-PCR assay. (**B**) The protein expression in polysaccharides-treated HepG2 cells by WB assay. (**C**) Graphical representation of the average fold change in expression of the proteins between HepG2 cells and polysaccharides-treated HepG2 cells. Gene expression is normalized to β-actin expression. Values are means ± SD (n = 3), *p < 0.05, **p < 0.01. The full-length gels and blots are included in Supplemental Fig. S2.

To further assess the effects of VCP2 on the cell cycle and apoptosis, the mRNA and protein expressions of Cyclin D1 (gi|23273807), Cyclin E (gi|6630609), CDK4 (gi|49457488), p21^Wafl/Cip1^ (gi|453135), Bcl-XL (gi|510901), Bad (gi|49456825), XIAP (gi|32528299), Smac (gi|9454219) and Caspase-3 (gi|16516817) were measured via qRT-PCR and WB (Fig. 2, Supplemental Fig. S2). The expression levels of Cyclin D1, p21^Wafl/Cip1^ and Caspase-3 were significantly elevated at both the mRNA and protein levels (*p* < 0.01) in HepG2 cells; the expression of Smac was significantly increased, and the expression of Bad was non-significantly increased (*p* < 0.05). However, the expression levels of Cyclin E, CDK4 and Bcl-XL were significantly lower both at the mRNA and protein levels (*p* < 0.01) in HepG2 cells, and the expression of XIAP was significantly decreased (*p* < 0.05) (Fig. 2A). Densitometry showed that Cyclin D1, p21^Wafl/Cip1^, Smac and Caspase-3 were increased 1.09-fold (*p* < 0.05), 1.44-fold (*p* < 0.01), 1.03-fold (*p* < 0.05) and 1.19-fold (*p* < 0.01), respectively. However, Cyclin E, CDK4 and Bcl-XL, Bad and XIAP were significantly decreased 0.95-fold (*p* < 0.05), 0.79-fold (*p* < 0.01), 0.81-fold (*p* < 0.01), 0.95-fold (*p* < 0.05) and 0.94-fold (*p* < 0.01), respectively, in VCP2-treated HepG2 cells relative to HepG2 cells (Fig. 2C).

### Identification of differentially expressed proteins in normal and VCP2-treated HepG2 cells and Caco2 cells

The samples from normal and VCP2-treated HepG2 cells were labeled with iTRAQ reagents 117 and 119 after reductive alkylation and were trypsin-digested, purified and separated using strong cation exchange reversed phase liquid chromatography (SCX-RPLC) and reversed phase liquid chromatography mass spectrometry (RPLC-MSMS). The ratio of 119:117 reflected the differences in the expression of the protein in normal and VCP2-treated HepG2 cells. After data filtering to eliminate low-scoring spectra, a total of 59923 unique spectra that met the strict confidence criteria for identification were matched to 2914 unique proteins (Supplemental Table S1), of which 2542 were reliably identified and quantified at a 95% confidence level (Supplemental Table S2). Compared with control HepG2 cells, 113 proteins had identified from 2914 proteins according to the condition of FC > 2 or <0.5, *p* < 0.05 (Supplemental Table S3, Supplemental Fig. S3). Notably, 59 proteins were increased (FC > 2, *p* < 0.05), and 54 proteins were decreased (FC < 0.5, *p* < 0.05). The RAW proteomics data were deposited into ProteomeXchange via the PRIDE repository under the dataset identifier PXD005323. The samples from normal and VCP2-treated Caco2 cells were carried out quantitative proteomic analysis using the same iTRAQ method, 198 proteins had identified from 5520 proteins according to the condition of FC > 1.5 or <0.67, *p* < 0.05 (Supplemental Table S4, Supplemental Fig. S4), and 99 proteins were increased (FC > 1.5, *p* < 0.05), 99 proteins were decreased (FC < 0.67, *p* < 0.05).

### Validation by qRT-PCR and WB

**Figure 3:**
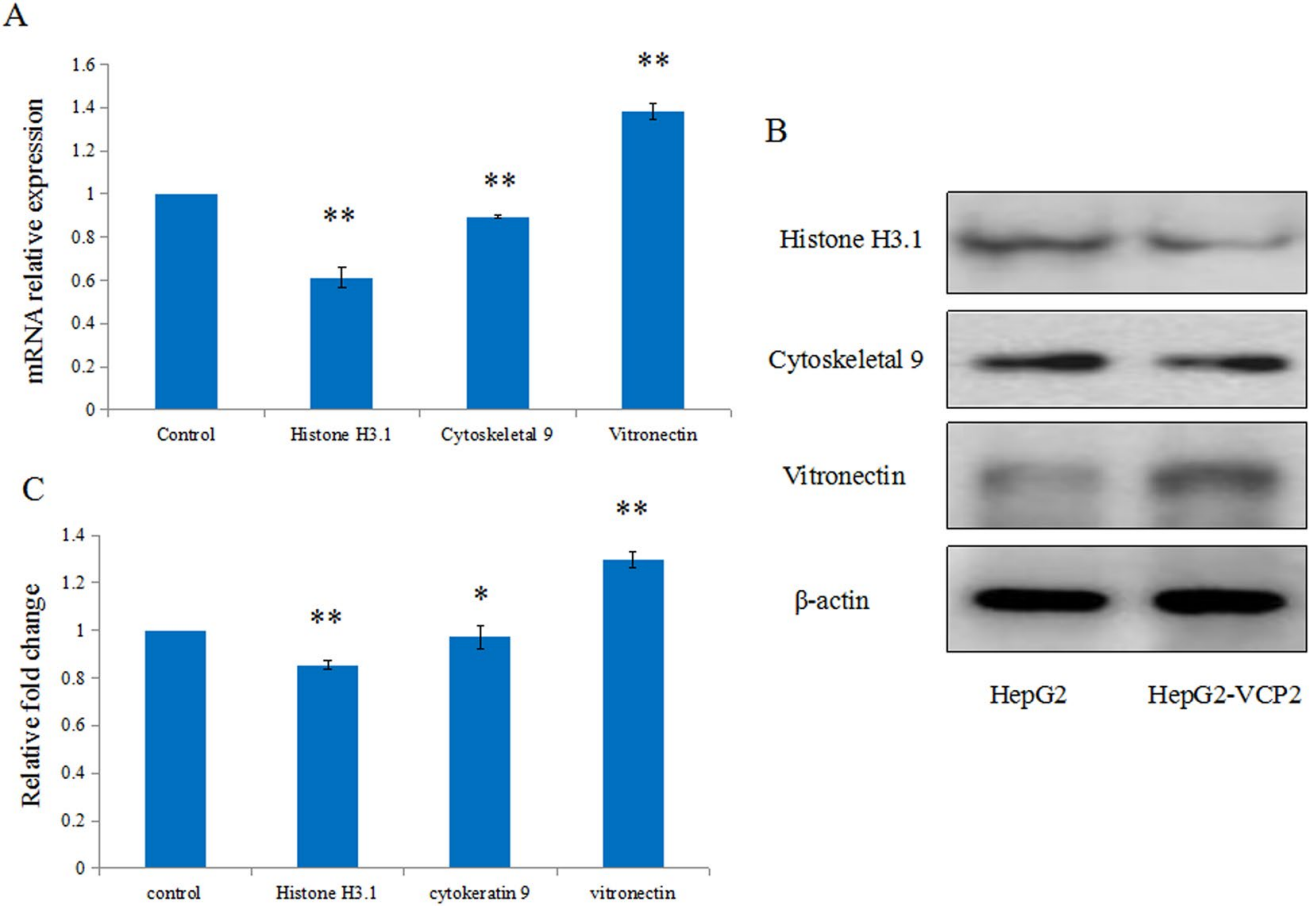
Validation by qRT-PCR and WB. (**A**) Validation of mRNA level in normal and polysaccharides-treated HepG2 cells by qRT-PCR assay. (**B**) Validation of the protein level in normal and polysaccharides-treated HepG2 cells by WB assay. (**C**) Graphical representation of the average fold change in expression of the proteins between HepG2 cells and polysaccharides-treated HepG2 cells. Gene expression is normalized to β-actin expression. Values are means ± SD (n = 3), *p < 0.05, **p < 0.01. The full-length gels and blots are included in Supplemental Fig. S5.

On the basis of two iTRAQ quantitative proteomic results, 3 differentially expressed proteins at the same time in two treatment groups were selected from 113 differentially expressed proteins in normal and VCP2-treated HepG2 cells for further validation study. The mRNA and protein expressions of Histone H3.1 (gi|4504285), Cytoskeletal 9 (gi|55956899) and Vitronectin (gi|88853069) were measured via qRT-PCR and WB (Fig. 3, Supplemental Fig. S5). The expression levels of Histone H3.1 and Cytoskeletal 9 were decreased at both the mRNA and protein levels (*p* < 0.05) in HepG2 cells, the expression levels of Vitronectin significantly increased 1.30-fold in VCP2-treated HepG2 cells relative to HepG2 cells (*p* < 0.01). The statistical analysis showed that the mRNA and protein levels agree with iTRAQ proteomic results.

### GO, KEGG pathway and PPI analysis of the identified proteins

**Figure 4:**
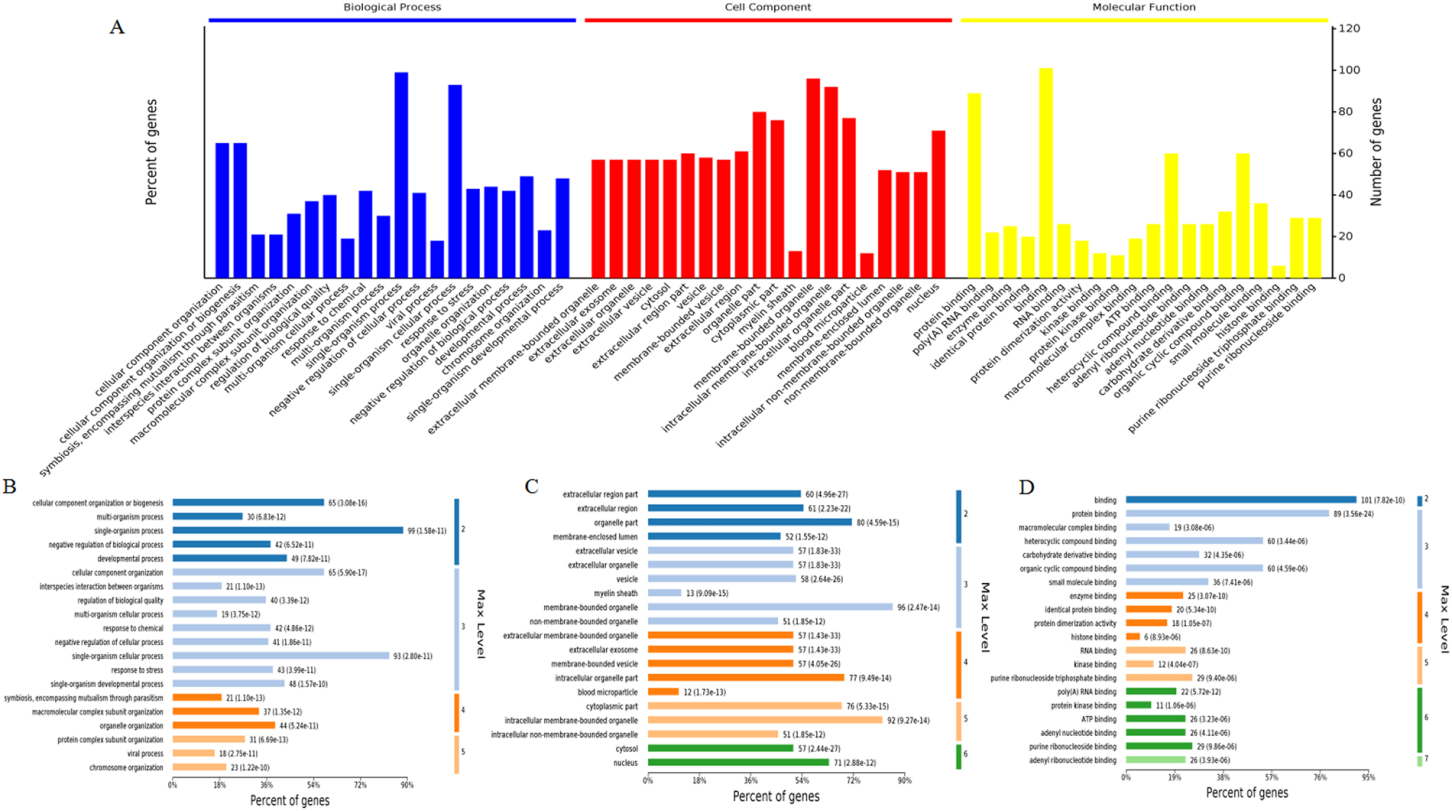
GO annotation of differentially expressed proteins in normal and polysaccharides-treated HepG2 cells. (**A**) The top 20 categories enriched BP, CC and MF. (**B**) The top 20 categories enriched BP terms. (**C**) The top 20 categories enriched CC terms. (**D**) The top 20 categories enriched MF terms.

GO annotation of 113 differentially expressed proteins identified 2753, 402 and 515 categories in the biological process (BP), cellular component (CC) and molecular function (MF) categories, respectively (Supplemental Tables S5–7), of which 1355, 186 and 218 categories were significant (*p* < 0.05). The top 20 categories in BP, CC and MF are shown in Fig. 4A. GO is structured as a graph, terms would appear at different ‘levels’ if different paths were followed through the graph. In BP, the top 20 processes mainly involved 4 maximum levels, and the percentage of genes was highest in the single-organism process and the single-organism cellular process, followed by cellular component organization or biogenesis and multi-organism process (Fig. 4B). In CC, the top 20 components included 5 maximum levels: membrane-bound organelles and intracellular membrane-bound organelles, followed by the organelle part, intracellular organelle part, cytoplasmic part and nucleus, which was associated with more proteins (Fig. 4C). In MF, the top 20 functions contained 6 maximum levels, and most of the proteins were associated with binding and protein binding (Fig. 4D).

**Figure 5:**
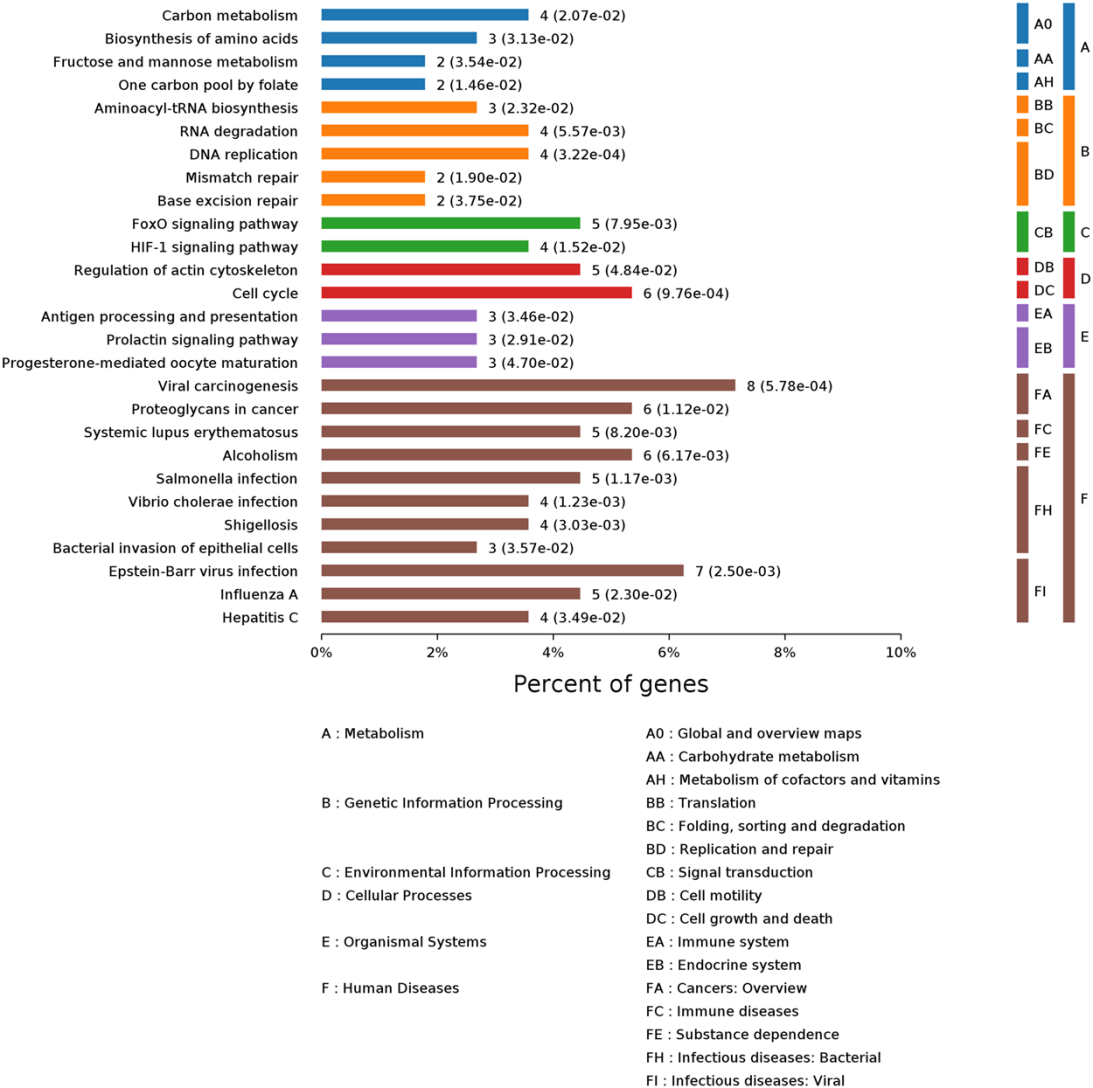
Significantly enriched KEGG pathways of differentially expressed proteins, *p < 0.05.

KEGG pathway enrichment categorized the 113 identified proteins into 6 specific pathways (Fig. 5). The proteins were primarily involved in metabolism, genetic information processing, environmental information processing, cellular processes, organismal systems and human diseases. The upregulated 14-3-3γ protein (gi|6016838) was related to some pathways, including the viral carcinogenesis^20^, cell cycle^21^, Epstein-Barr virus infection^22^, the Hippo signaling pathway^23^, oocyte meiosis^24^ and PI3K-Akt signaling pathway^25^, but the downregulated PLK1 protein (gi|21359873) was associated with the cell cycle^26^, the FoxO signaling pathway^27^, progesterone-mediated oocyte maturation^28^ and oocyte meiosis^29^.

**Figure 6:**
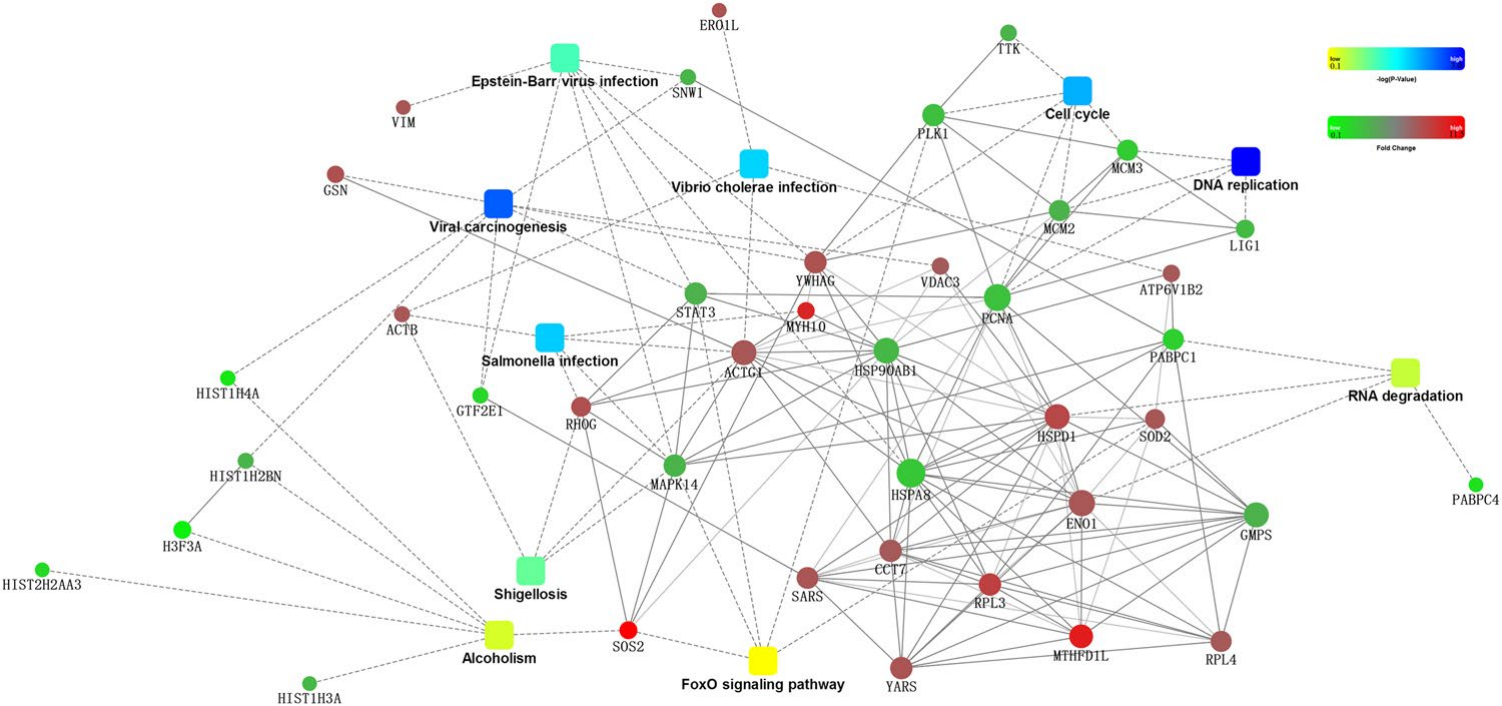
PPI network of differentially expressed proteins constructed in STRING database. The solid lines represent direct interactions between proteins, and the dotted lines indicate indirect interactions between proteins. The dots stand for differentially expressed proteins, different colors shows FC of identified proteins, different diameter representative the number of proteins interacted with the protein. The squares stand for differentially expressed proteins involving in GO or KEGG pathways.

To elucidate the interactions of 113 differentially expressed proteins, a PPI network was generated by using the STRING database (Fig. 6, Supplemental Table S8). The interaction included both direct physical interaction and indirect functional correlations among proteins. In VCP2-treated HepG2 cells, PLK1 interacted directly with 14-3-3γ, the DNA replication licensing factor MCM3 (gi|394582099), the DNA replication licensing factor MCM2 (gi|33356547) and proliferating cell nuclear antigen (gi|33239451); 14-3-3γ also interacted directly with the DNA replication licensing factor MCM2 and interacted indirectly with Gelsolin (gi|121116). PLK1 and 14-3-3γ were involved in many pathways, including the cell cycle and viral carcinogenesis; the interaction relationship between upregulated 14-3-3γ, and downregulated PLK1 was closely related to cell cycle arrest in polysaccharide-treated HepG2 cells.

## Discussion and Conclusion

Recently, plant polysaccharides have drawn attention for their anti-tumor activity. Polysaccharides from pumpkins^30^, apples^31^, *Taxus chinensis var*. *mairei* ^32^, and *Ampelopsis megalophylla* ^33^ have induced cell proliferation inhibition and apoptosis in tumor cells. We have determined that the molecular weights of VCP1, VCP2 and VCP3 are 32 kDa, 280 kDa and 21 kDa, respectively. VCP1 is a neutral polysaccharide and consists of glucose, galactose, arabinose, rhamnose and mannose. VCP2 and VCP3 belong to the RG-I pectin family and are composed of glucose, galactose, arabinose, rhamnose, mannose, glucuronic acid and galacturonic acid. The degree of VCP2 esterification is 50%, and VCP3 exhibit a small amount of acetylation^34^. Three polysaccharides exhibited different anti-proliferation activities because of their various structures. In this study, the anti-proliferation activities of polysaccharides from *Viscum coloratum* (Kom.) *Nakai* were verified in HepG2 cells with CCK-8 assays. VCP2 inhibited cell growth and delayed the cell cycle in G1 phase, as detected by PI staining, and induced apoptosis, as detected by Annexin V-FITC/PI staining and flow cytometry analysis.

Cell cycle regulation is primarily carried out by the phosphorylation and dephosphorylation of cyclin and cyclin dependent kinase (CDK) complexes. Important regulatory cyclins include Cyclin A (gi|1567308), Cyclin B (gi|371905556), Cyclin C (gi|112180464), Cyclin D1, Cyclin D2 (gi|38416), Cyclin D3 (gi|181247) and Cyclin E. CDKs are a category of serine-threonine protein kinase that form Cyclin-CDK complexes. Aberrant G1/S transition is one of the main reasons for tumor formation. Cyclin D and Cyclin E play an important role in the regulation of the transition between G1 and G1/S phases. Combinations of Cyclin D and CDK4/6 are key to the transition from the G0 to the G1 phase^35^. The G1/S phase transition is promoted by Cyclin E in combination with CDK2^36^ (gi|29849). Cyclin D and Cyclin E are overexpressed in tumor cells^37, 38^. In addition, p21 is a CDK inhibitor that inhibits the production of Cyclin-CDK complexes. The overexpression of p21^Wafl/Cip1^ delays the cell cycle in G1 or S phase^39^. In this study, the increased expression of p21^Wafl/Cip1^ and Cyclin D and the decreased expression of Cyclin E and CDK4 in VCP2-treated HepG2 cells may be possible reasons for the arrest at the G1 phase of the cell cycle, thus leading to the inhibition of cellular proliferation.

Apoptosis is a highly complex process involving multi-gene interactions. The caspase family plays an important role in apoptosis, and Caspase-3 ultimately carries out apoptosis^40^. The anti-apoptotic Bcl-XL protein in the Bcl-2 family as well as Bad inhibits the activation of Caspase-3, thereby restraining apoptosis^41^. XIAP is the strongest apoptosis inhibitory protein in the IAP family and is usually overexpressed in tumor cells^42^. Combinations of Smac and IAP (gi|157266292) release caspases, thus promoting apoptosis^43^. In this study, the increased expression of Bad, Smac and Caspase-3 and the decreased expression of Bcl-XL and XIAP may contribute to the induction of apoptosis in VCP2-treated HepG2 cells.

HCC is a malignant cancer with multi-protein interactions. The screening of protein markers for cancer discovery, treatment, prognosis diagnosis is of great value. In the current study, we used iTRAQ combined with 2D-LC-MSMS analysis to identify a total of 2914 proteins, of which 113 differentially expressed proteins were screened in VCP2-treated HepG2 cells, 198 proteins had identified from 5520 proteins in normal and in VCP2-treated Caco2 cells. Three proteins were selected to examine protein abundances from iTRAQ analyses agree with protein abundances from WB analyses or mRNA abundances from qRT-PCR analyses. Further bioinformatics analysis showed that 59 proteins were upregulated and 54 proteins were downregulated in VCP2-treated HepG2 cells. However, 99 proteins were upregulated and 99 proteins were downregulated in VCP2-treated Caco2 cells. And then 113 differentially expressed proteins in VCP2-treated HepG2 cells were involved in many GO annotations, pathways and interactions.

14-3-3 regulates the cell cycle in the transition of G2/M or G1/S by binding many cyclins^44^. Phosphorylated Bad binds 14-3-3, thus leading to the inhibition of apoptosis^45^. 14-3-3 is usually overexpressed in tumor cells, as is 14-3-3γ^46, 47^. 14-3-3 is an important protein that mediates signaling pathways by combining with serine phosphorylation proteins. PLK is a type of highly conservative serine/threonine protein kinase. PLK1 regulates the cell cycle and promotes the beginning of the M phase and the separation of chromosomes during mitosis^48^. PLK1 is involved in almost 75% of the signaling pathways that are closely related to tumorigenesis, including the cell cycle and the DNA damage repair pathway^49^. PLK1 proteins play critical roles in the control of cell cycle progression, and therapeutic approaches aimed at inactivating PLK1 might be highly useful in the treatment of HCC^50^. DNA damage repair pathway, virus carcinogenesis pathway and proteins involved in this pathways were closely related to HCC^51^.

In conclusion, we provide evidence that polysaccharides from *Viscum coloratum* (Kom.) *Nakai* induced HepG2 cell growth inhibition and apoptosis. Importantly, our iTRAQ quantitative proteomics approach identified differentially expressed proteins in VCP2-treated HepG2 cells. We also present the corresponding bioinformatics analysis of the identified proteins. Although the current study demonstrates that some of the differentially expressed proteins may be responsive to polysaccharides, further studies and verification are necessary.

## Methods

### Cell culture and reagents

Three polysaccharides, named VCP1, VCP2 and VCP3, were isolated and purified from *Viscum coloratum* (Kom.) *Nakai* using DEAE-cellulose chromatography as previously described^34^. Human HepG2 cells and Caco2 cells were cultured with Dulbecco’s modified Eagle’s medium (DMEM) (Gibco, Grand Island, NY, USA) containing 10% fetal bovine serum (FBS) (Gibco), 100 units/mL penicillin (Gibco) and 100 μg/mL streptomycin (Gibco) in a 37 °C incubator with a humidified atmosphere of 5% CO_2_. A CCK-8 kit, Annexin V-FITC apoptosis detection kit, RNA extraction kit and enhanced BCA protein assay kit were purchased from Beyotime Institute of Biotechnology (Shanghai, China), a HiFiScript cDNA synthesis kit was purchased from ComWin Biotechnology (Beijing, China), a SYBR^®^ Green Real-time PCR Master Mix kit was purchased from TOYOBO (Osaka, Japan), an SCX column, C18 precolumn and C18 reversed phase column were purchased from Agilent Technologies (Santa Clara, CA, USA), and an iTRAQ Reagents Multi-Plex Kit was obtained from AB SCIEX (Foster City, CA, USA). Primary antibodies, including anti-Cyclin D1 (2926 s), anti-Cyclin E (4129 s), anti-CDK4 (2906 s), anti-p21^Wafl/Cip1^ (2946 s), anti-Bcl-XL (2764 s), anti-Bad (9292 s), anti-XIAP (2045 s), anti-Smac/Diablo (2954 s), anti-Caspase-3 (9662 s), anti-β-actin (4970), and secondary antibodies, including anti-rabbit (7074 s) and anti-mouse (7076 s) were purchased from Cell Signaling Technology (Danvers, MA, USA).

### Cell proliferation assay

Cell proliferation was measured with a CCK-8 kit. HepG2 cells were collected in logarithmic phase, the concentration of the cell suspension was adjusted to 5 × 10^4^ cells/mL, and 100 μL/well was inoculated into 96-well plates, which were maintained at 37 °C and 5% CO_2_ in a humidified incubator. After 24 h, the supernatant was discarded, concentrations of 20 μg/mL, 40 μg/mL, 60 μg/mL, 80 μg/mL, and 100 μg/mL of VCP1, VCP2 and VCP3 were added to the wells at a volume of 100 μL/well, and cells were maintained at 37 °C and 5% CO_2_ in an humidified incubator for 48 h. At the same time, the control group, which received only 100 μL of DMEM, was prepared. Three repeated wells were included in each group; 10 μL of CCK-8 reagents was added to each well for 2 h. OD values were measured at 450 nm with an enzyme-labeling instrument (Bio-Rad, USA). The proliferation rate and inhibition rate were calculated as:

1 $${\rm{Proliferation}}\,{\rm{rate}}\,( \% )=({\rm{A}}450\,{{\rm{nm}}}_{{\rm{experimental}}{\rm{group}}}/{\rm{A}}450\,{{\rm{nm}}}_{{\rm{control}}{\rm{group}}})\times 100 \% $$

2 $${\rm{Inhibition}}\,{\rm{rate}}\,( \% )=1\,-\,{\rm{Proliferation}}\,{\rm{rate}}\,( \% )$$

### Cell cycle distribution analysis

Cell cycle distribution was determined via propidium iodide (PI) staining and flow cytometry. HepG2 cells treated with polysaccharides for 48 h were trypsinized and adjusted to 1–5 × 10^6^ cells/mL, and the cell pellet was collected after cells were centrifuged at 4 °C and 800 rpm for 10 min. The cell pellet was rinsed twice with pre-cooled phosphate buffer solution (PBS) and centrifuged at 4 °C and 800 rpm for 5 min and then was fixed for 2 h at 4 °C in pre-cooled 70% (v/v) ethanol with mixing for 15 min. The cell pellet was collected after being centrifuged and rinsed using pre-cooled PBS. The cell pellet was incubated with PI staining solution and TritonX-100. The cell pellet was re-suspended in pre-cooled PBS after being centrifuged at 4 °C and 800 rpm for 5 min and was assessed via FACSAria flow cytometry (Becton-Dickinson, Mansfield, MA, USA) and Modfit software (Version 3.2, Bio-Rad, CA, USA).

### Apoptosis Assay

Apoptosis was detected with an Annexin V-FITC apoptosis detection kit and flow cytometry. After the treatments were administered, the concentration of the cell suspension was adjusted to 1–5 × 10^5^ cells/mL, and the cell pellet was collected after being centrifuged at 4 °C and 300 g for 5 min. Pre-cooled PBS was added to rinse the cell pellet, and the pellet was centrifuged again. The cell pellet was re-suspended using 100 μL of 1× Binding Buffer and then was incubated with 5 μL of Annexin V-FITC and 5 μL of PI staining solution for 10 min in the dark. Finally, 400 μL of 1× Binding Buffer was added into the mixture. The apoptotic cells were detected via FACSAria flow cytometry (Becton-Dickinson), and the results were analyzed with Modfit software (Bio-Rad).

### qRT-PCR Assay

The mRNA expression of several targets from HepG2 cells and polysaccharide-treated HepG2 cells was evaluated using qRT-PCR. Total RNA was isolated with an RNA extraction kit and reverse-transcribed to cDNA using a HiFiScript cDNA synthesis kit according to the manufacturer’s protocol. qRT-PCR was carried out on ABI7500 (Applied Biosystems, USA) using a SYBR^®^ Green Real-time PCR Master Mix kit. Primer pairs for qRT-PCR are listed in Supplemental Table S9. β-actin was amplified and used as a housekeeping control gene. A control sample that contained no reverse transcriptase was included to confirm the absence of contaminating DNA. The relative expression levels of different targets in HepG2 cells were calculated according to the 2^−ΔΔCt^ method.

### WB assay

Protein expression in the control and polysaccharide-treated HepG2 cells was verified using WB analysis. Cells were harvested, and total protein was extracted with a cell lysis buffer (9 M Urea, 4% CHAPS, 1% DTT, 1% IPG buffer (GE Healthcare)) and PMSF protease inhibitor. The concentration of total protein was quantified with an enhanced BCA protein assay kit. Total protein samples were separated by SDS-PAGE on a 15% or a 5% gel and then transferred to PVDF membranes. Membranes were blocked in TBST containing 5% nonfat milk for 2 h and were incubated with respective primary antibodies (1:2000) for 3 h at room temperature. The membranes were washed with TBST three times for 10 min per wash and subsequently incubated with the HRP-conjugated secondary antibodies (1:2000) for 2 h at room temperature, then washed with TBST three times for 10 min each again. β-actin was used as an internal control. WB bands were visualized using DAB (PIERCE, Rockford, USA) and an LAS-3000 imaging system (FUJIFILM, Japan).

### Protein preparation and iTRAQ labeling

HepG2 cells and Caco2 cells were cultured for 24 h in 6-well plates, and 100 μg/mL of VCP2 was added to each well with 2 mL; cells were maintained at 37 °C and 5% CO_2_ in a humidified incubator for 48 h, and DMEM was used as the control treatment. The experiment was carried out twice. Two parallel group of normal cells and polysaccharide-treated cells were collected and washed twice with pre-cooled PBS. The extraction and the concentration of total protein were performed as described previously for WB assays. Total protein was separated by SDS-PAGE on a 12% separating gel and a 5% stacking gel. A total of 100 μg of denatured protein from each sample was dissolved in dissolution buffer, and then reducing reagent and cysteine-blocking reagent were added into the solution to reductive alkylation. After being washed three times with dissolution buffer, the samples were digested with trypsin. Finally, peptides were washed twice in dissolution buffer and were pooled for iTRAQ labeling according to the manufacturer’s instructions. Samples were labeled with the iTRAQ tags as follows: HepG2 (117 tag), HepG2 (118 tag), polysaccharide-treated HepG2 (119 tag), polysaccharide-treated HepG2 (121 tag) and then were combined and vacuum-dried^52^. The samples from normal and VCP2-treated Caco2 cells were carried out quantitative proteomic analysis using the same iTRAQ tags and assay.

### 2D-LC-MSMS Analysis

The dried fraction of labeled peptides was dissolved in mobile phase A (5% acetonitrile (ACN), 0.1% formic acid (FA) in deionized water) and purified using an SCX column at a constant flow rate of 0.3 mL/min on an Agilent 1200 HPLC (Agilent, Santa Clara, CA, USA) with a gradient from 2 to 25% phase B (90% ACN, 0.1% FA in deionized water) for 40 min, to 90% phase B for 10 min, maintained 90% phase B for 10 min, and 2% phase B for 5 min. Absorbances at 210 nm and 280 nm were monitored, and a total of 10 fractions were collected and vacuum-dried. Subsequently, peptide separation was performed with a Nano-RPLC using an Eksigent nanoLC-Ultra™ 2D system (AB SCIEX). The sample fractions were loaded onto a C18 precolumn (100 µm × 3 cm, 3 µm, 150 Å) and were desalted for 10 min at a flow rate of 2 µL/min. The samples were then loaded onto a C18 reverse phase column (75 μm × 15 cm, 3 μm, 120 Å, ChromXP Eksigent) with a gradient from 5 to 35% mobile phase B for 70 min.

A TripleTOF5600 mass spectrometer (AB SCIEX, Foster City, CA, USA) under a TripleTOF5600 system fitted with a Nanospray III source (AB SCIEX), and a pulled quartz tip as the emitter (New Objectives, MA, USA) was used for all measurements. The parameters were set as follows: the spray voltage was 2.5 kV, the curtain gas was 30 PSI, and the nebulizer gas was 5 PSI; the interface heater temperature was 150 °C, and mass spectra were acquired in an information dependent analysis mode; survey scans were acquired in 250 ms, and 35 product ions maximum were collected. If these product ions exceeded a threshold of 150 counts/s with a 2^+^ to 5^+^ charge-state, the cycle time was 2.5 s; the normalized collision energy was applied to all precursor ions for collision-induced dissociation (CID). Dynamic exclusion was set for 1/2 of peak width (18 s), and then the precursor ion exclusion (PIE) lists were used to minimize redundancy.

### Protein identification and quantification

Protein quantification was also performed with ProteinPilot software (Version 5.0, AB SCIEX) against a Homo sapiens database using the Paragon algorithm. Protein identification was performed with methylmethanethiosulfonate (MMTS) selected for cysteine modification with iodoacetamide as a biological modification; the false discovery rate (FDR) analysis was estimated using an automatic decoy database search strategy in the PSPEP Software (Proteomics System Performance Evaluation Pipeline Software, integrated in the ProteinPilot Software). The peptides in iTRAQ labeling were chosen for protein quantification with unique peptides during the search. Only peptides with a global FDR of ≤1% identified were considered for further analysis. The *p*-values were generated by ProteinPilot Software on the basis of the peptides used to quantify the respective proteins. Finally, FC was calculated as the average ratio of 119/117 and 118/121 for differentially expressed proteins, and samples with an FC > 2 or <0.5 and *p* < 0.05 were considered to be significantly differentially expressed proteins in normal and VCP2-treated HepG2 cells, and samples with an FC > 1.5 or <0.67 and *p* < 0.05 were considered to be significantly differentially expressed proteins in normal and VCP2-treated Caco2 cells.

### Validation by qRT-PCR and WB

Primer pairs for qRT-PCR are listed in Supplemental Table S9. qRT-PCR and WB assay were reference to above-mentioned methods.

### Bioinformatics analysis

All of the differentially expressed proteins in normal and VCP2-treated HepG2 cells were examined using GO annotation and pathway enrichment according to an online DAVID tool (http://david.abcc.ncifcrf.gov/). The GO analysis further classified proteins into three subcategories: BP, CC and MF. KEGG pathway enrichment was performed on the KEGG database (http://www.kegg.jp/). PPI networks were constructed using the STRING database (http://string.embl.de/).

### Statistical analysis

Cell proliferation, cell cycle distribution, apoptosis Assay, qRT-PCR and WB were carried out biologically in triplicate. The data are expressed as the means ± standard error of the mean (SEM). All statistical analyses were performed with SPSS 19.0 software (Chicago, IL, USA). The statistically significant differences were tested using *t*-tests and ANOVA (*p* < 0.05).

## Electronic supplementary material

Supplementary Information
