# Supplementary material for: iTRAQ-Based Quantitative Proteomic Analysis of the Inhibitory Effects of Polysaccharides from Viscum coloratum (Kom.) Nakai on HepG2 Cells
Source: Sci Rep. 2017 Jul 4;7:4596. doi: 10.1038/s41598-017-04417-x (PMC5496916; doi:10.1038/s41598-017-04417-x)
Supplement: Supplementary file 1 — Supplementary Information [file 41598_2017_4417_MOESM1_ESM.pdf]

Supplementary Information

iTRAQ-Based Quantitative Proteomic Analysis of the Inhibitory Effects of Polysaccharides from *Viscum coloratum (Kom.) Nakai* on HepG2 Cells

Yangyang Chai[1], Min Zhao[1]

Supplemental Table S1 Overview of protein quantitation

Supplemental Table S2 A total of identiﬁed proteins of normal and VCP2-treated HepG2 cells by iTRAQ

Supplemental Table S3 Differentially expressed proteins of normal and VCP2-treated HepG2 cells identified by 2D-LC-MSMS

Supplemental Table S4 Differentially expressed proteins of normal and VCP2-treated Caco2 cells identified by 2D-LC-MSMS

Supplemental Table S5 BP enrichment analysis of differentially expressed proteins

Supplemental Table S6 CC enrichment analysis of differentially expressed proteins

Supplemental Table S7 MF enrichment analysis of differentially expressed proteins

Supplemental Table S8 PPI analysis of differentially expressed proteins

Supplemental Table S9 Primer pairs for qRT-PCR

Supplemental Figure S1 The growth of normal and VCP2-treated HepG2 cells

Supplemental Figure S2 The protein expression in polysaccharides-treated HepG2 cells by WB assay

Supplemental Figure S3 A volcano plot of identified proteins in normal and

VCP2-treated HepG2 cells

Supplemental Figure S4 A volcano plot of identified proteins in normal and VCP2-treated Caco2 cells

Supplemental Figure S5 The protein expression validation by WB assay

Table S1 Overview of protein quantitation

Unused (Conf) Cutoff	Proteins Detected	Proteins Before Grouping	Distinct Peptides	Spectra Identified	% Total Spectra
>2.0 (99)	1927	2745	17391	55996	31
>1.3 (95)	2627	4029	18490	58983	32.7
>0.47 (66)	2732	4420	18672	59420	32.9
Cutoff Applied: >0.05 (10%)	2914	5629	18910	59923	33.2

Table S2 A total of identified proteins of normal and VCP2-treated HepG2 cells by iTRAQ

Number	Name	Accession and Gene	% Cov	Peptides (95%)	119:117	P-Val (119:117)
1	Cytoplasmic dynein 1 heavy chain 1 OS=Homo sapiens GN=DYNC1H1 PE=1 SV=5	sp|Q14204|DYHC1_HUMAN	19.9	54	1.888	0.0006
2	Clathrin heavy chain 1 OS=Homo sapiens GN=CLTC PE=1 SV=5	sp|Q00610|CLH1_HUMAN	45.6	74	1.1695	0.2102
3	Talin-1 OS=Homo sapiens GN=TLN1 PE=1 SV=3	sp|Q9Y490|TLN1_HUMAN	39.2	102	1.3183	0.0777
4	Pyruvate kinase PKM OS=Homo sapiens GN=PKM PE=1 SV=4	sp|P14618|KPYM_HUMAN	72.7	193	2.1478	0.0267
5	Myosin-9 OS=Homo sapiens GN=MYH9 PE=1 SV=4	sp|P35579|MYH9_HUMAN	34.2	59	0.9462	0.9316
6	Plectin OS=Homo sapiens GN=PLEC PE=1 SV=3	sp|Q15149|PLEC_HUMAN	19.1	53	1.1695	0.1718
7	Elongation factor 2 OS=Homo sapiens GN=EEF2 PE=1 SV=4	sp|P13639|EF2_HUMAN	61	91	1.1695	0.1856
8	Heat shock cognate 71 kDa protein OS=Homo sapiens GN=HSPA8 PE=1 SV=1	sp|P11142|HSP7C_HUMAN	73.7	174	0.4406	0.019
9	Actin, cytoplasmic 2 OS=Homo sapiens GN=ACTG1 PE=1	sp|P63261|ACTG_HUMAN	78.7	236	1.7865	0.078
10	Translational activator GCN1 OS=Homo sapiens GN=GCN1L1 PE=1 SV=6	sp|Q92616|GCN1L_HUMAN	20.7	35	0.9908	0.5867
11	Transitional endoplasmic reticulum ATPase OS=Homo sapiens GN=VCP PE=1 SV=4	sp|P55072|TERA_HUMAN	58.4	82	1.2023	0.3243
12	E3 ubiquitin-protein ligase UBR4 OS=Homo sapiens GN=UBR4 PE=1 SV=1	sp|Q5T4S7|UBR4_HUMAN	10.6	28	1.5136	0.0489
13	Microtubule-actin cross-linking factor 1, isoforms 1/2/3/5 OS=Homo sapiens GN=MACF1 PE=1 SV=4	sp|Q9UPN3|MACF1_HUMAN	8.6	31	0.8017	0.5069
14	Heat shock protein HSP 90-beta OS=Homo sapiens GN=HSP90AB1 PE=1 SV=4	sp|P08238|HS90B_HUMAN	50	72	0.4786	0.0566
15	Plastin-2 OS=Homo sapiens GN=LCP1 PE=1	sp|P13796|PLSL_HUMAN	59	69	1.5996	0.1337
16	Ubiquitin-like modifier-activating enzyme 1 OS=Homo sapiens GN=UBA1 PE=1 SV=3	sp|P22314|UBA1_HUMAN	36.8	48	1.3428	0.1522

17	Tubulin beta chain OS=Homo sapiens GN=TUBB PE=1 SV=2	sp|P07437|TBB5_HUMAN	73	77	0.929	0.276
18	Heat shock 70 kDa protein 4 OS=Homo sapiens GN=HSPA4 PE=1	sp|P34932|HSP74_HUMAN	44.2	45	1.1272	0.4702
19	Elongation factor 1-alpha 1 OS=Homo sapiens GN=EEF1A1 PE=1 SV=1	sp|P68104|EF1A1_HUMAN	71.4	439	0.5808	0.1633
20	ATP synthase subunit beta, mitochondrial OS=Homo sapiens GN=ATP5B PE=1 SV=3	sp|P06576|ATPB_HUMAN	66.7	83	1.1169	0.5111
21	Alpha-enolase OS=Homo sapiens GN=ENO1 PE=1 SV=2	sp|P06733|ENOA_HUMAN	71.7	103	1.803	0.2671
22	U5 small nuclear ribonucleoprotein 200 kDa helicase OS=Homo sapiens GN=SNRNP200 PE=1 SV=2	sp|O75643|U520_HUMAN	19	23	0.6368	0.0768
23	Stress-70 protein, mitochondrial OS=Homo sapiens GN=HSPA9 PE=1 SV=2	sp|P38646|GRP75_HUMAN	47.4	38	1.3428	0.2104
24	60 kDa heat shock protein, mitochondrial OS=Homo sapiens GN=HSPD1 PE=1 SV=2	sp|P10809|CH60_HUMAN	59.5	111	0.863	0.7666
25	Pre-mRNA-processing-splicing factor 8 OS=Homo sapiens GN=PRPF8 PE=1	sp|Q6P2Q9|PRP8_HUMAN	16.2	24	0.5702	0.0226
26	Ras GTPase-activating-like protein IQGAP1 OS=Homo sapiens GN=IQGAP1 PE=1 SV=1	sp|P46940|IQGA1_HUMAN	24.6	34	1.4191	0.1371
27	Sodium/potassium-transporting ATPase subunit alpha-1 OS=Homo sapiens GN=ATP1A1	sp|P05023|AT1A1_HUMAN	28.5	42	1.2023	0.5568
28	Moesin OS=Homo sapiens GN=MSN PE=1 SV=3	sp|P26038|MOES_HUMAN	51.1	31	2.208	0.0974
29	CAD protein OS=Homo sapiens GN=CAD PE=1 SV=3	sp|P27708|PYR1_HUMAN	19.6	28	0.9727	0.7196
30	Proteasome-associated protein ECM29 homolog OS=Homo sapiens GN=ECM29 PE=1 SV=2	sp|Q5VYK3|ECM29_HUMAN	17.7	18	1.2823	0.6369
31	E3 ubiquitin-protein ligase HUWE1 OS=Homo sapiens GN=HUWE1 PE=1	sp|Q7Z6Z7|HUWE1_HUMAN	10.1	22	0.9036	0.5586
32	116 kDa U5 small nuclear ribonucleoprotein component OS=Homo sapiens GN=EFTUD2 PE=1 SV=1	sp|Q15029|U5S1_HUMAN	35.1	22	0.6607	0.1386

33	Tubulin alpha-1A chain OS=Homo sapiens GN=TUBA1A PE=1 SV=1	sp|Q71U36|TBA1A_HUMAN	59.9	66	0.8954	0.8356
34	Coatomer subunit alpha OS=Homo sapiens GN=COPA PE=1 SV=2	sp|P53621|COPA_HUMAN	23.3	18	1.2246	0.2639
35	Chromodomain-helicase-DNA-binding protein 4 OS=Homo sapiens GN=CHD4 PE=1 SV=2	sp|Q14839|CHD4_HUMAN	16	19	0.673	0.1048
36	T-complex protein 1 subunit beta OS=Homo sapiens GN=CCT2 PE=1 SV=4	sp|P78371|TCPB_HUMAN	59.8	47	1.028	0.8897
37	Glyceraldehyde-3-phosphate dehydrogenase OS=Homo sapiens GN=GAPDH PE=1 SV=3	sp|P04406|G3P_HUMAN	67.8	110	0.955	0.8957
38	ATP-citrate synthase OS=Homo sapiens GN=ACLY PE=1 SV=3	sp|P53396|ACLY_HUMAN	22.5	15	1.2246	0.2321
39	Phosphoglycerate kinase 1 OS=Homo sapiens GN=PGK1 PE=1 SV=3	sp|P00558|PGK1_HUMAN	66.7	66	1.3183	0.1103
40	78 kDa glucose-regulated protein OS=Homo sapiens GN=HSPA5 PE=1 SV=2	sp|P11021|GRP78_HUMAN	52	34	1.3552	0.1359
41	Cullin-associated NEDD8-dissociated protein 1 OS=Homo sapiens GN=CAND1 PE=1	sp|Q86VP6|CAND1_HUMAN	25	17	0.879	0.9143
42	Bifunctional glutamate/proline--tRNA ligase OS=Homo sapiens GN=EPRS PE=1 SV=5	sp|P07814|SYEP_HUMAN	15.8	18	1.4322	0.2245
43	Coatomer subunit gamma-1 OS=Homo sapiens GN=COPG1 PE=1 SV=1	sp|Q9Y678|COPG1_HUMAN	29.8	20	1.0375	0.5925
44	Importin-7 OS=Homo sapiens GN=IPO7 PE=1 SV=1	sp|O95373|IPO7_HUMAN	19	15	0.7727	0.547
45	T-complex protein 1 subunit alpha OS=Homo sapiens GN=TCP1 PE=1 SV=1	sp|P17987|TCPA_HUMAN	44.4	21	1.028	0.9515
46	Importin subunit beta-1 OS=Homo sapiens GN=KPNB1 PE=1	sp|Q14974|IMB1_HUMAN	27.4	23	0.6252	0.3499
47	Histone H2B type 1-N OS=Homo sapiens GN=HIST1H2BN PE=1 SV=3	sp|Q99877|H2B1N_HUMAN	78.6	41	0.5702	0.1178
48	T-complex protein 1 subunit delta OS=Homo sapiens GN=CCT4 PE=1 SV=4	sp|P50991|TCPD_HUMAN	42.5	41	1.0765	0.7252

49	Eukaryotic translation initiation factor 4 gamma 2 OS=Homo sapiens GN=EIF4G2 PE=1	sp|P78344|IF4G2_HUMAN	23.4	14	0.492	0.2087
50	Splicing factor 3B subunit 1 OS=Homo sapiens GN=SF3B1 PE=1	sp|O75533|SF3B1_HUMAN	20.9	15	0.6668	0.1733
51	Hypoxia up-regulated protein 1 OS=Homo sapiens GN=HYOU1 PE=1 SV=1	sp|Q9Y4L1|HYOU1_HUMAN	32.8	30	0.9727	0.9179
52	Kinesin-1 heavy chain OS=Homo sapiens GN=KIF5B PE=1 SV=1	sp|P33176|KINH_HUMAN	24.1	27	1.028	0.4901
53	ATP synthase subunit alpha, mitochondrial OS=Homo sapiens GN=ATP5A1 PE=1 SV=1	sp|P25705|ATPA_HUMAN	47.7	39	1.028	0.7381
54	Dynamin-2 OS=Homo sapiens GN=DNM2 PE=1 SV=2	sp|P50570|DYN2_HUMAN	23.9	15	1.3062	0.2679
55	60S ribosomal protein L3 OS=Homo sapiens GN=RPL3 PE=1 SV=2	sp|P39023|RL3_HUMAN	42.9	72	2.5351	0.8828
56	EH domain-containing protein 1 OS=Homo sapiens GN=EHD1 PE=1 SV=2	sp|Q9H4M9|EHD1_HUMAN	32.6	22	1.3428	0.2688
57	V-type proton ATPase catalytic subunit A OS=Homo sapiens GN=ATP6V1A PE=1 SV=2	sp|P38606|VATA_HUMAN	32.6	18	1.6444	0.0956
58	Cytoplasmic FMR1-interacting protein 1 OS=Homo sapiens GN=CYFIP1 PE=1	sp|Q7L576|CYFP1_HUMAN	18	18	1.1588	0.576
59	Alpha-actinin-4 OS=Homo sapiens GN=ACTN4 PE=1 SV=2	sp|O43707|ACTN4_HUMAN	26.1	19	0.8551	0.6005
60	tRNA (cytosine(34)-C(5))-methyltransferase OS=Homo sapiens GN=NSUN2 PE=1	sp|Q08J23|NSUN2_HUMAN	31.8	36	0.6982	0.2795
61	Vimentin OS=Homo sapiens GN=VIM PE=1	sp|P08670|VIME_HUMAN	43.8	22	2.421	0.0228
62	Exportin-1 OS=Homo sapiens GN=XPO1 PE=1 SV=1	sp|O14980|XPO1_HUMAN	19.4	17	0.8954	0.5589
63	Protein FAM49B OS=Homo sapiens GN=FAM49B PE=1 SV=1	sp|Q9NUQ9|FA49B_HUMAN	59.6	21	1	0.6977
64	T-complex protein 1 subunit eta OS=Homo sapiens GN=CCT7 PE=1 SV=2	sp|Q99832|TCPH_HUMAN	43.1	37	0.9462	0.8439
65	Eukaryotic initiation factor 4A-I OS=Homo sapiens GN=EIF4A1 PE=1	sp|P60842|IF4A1_HUMAN	41.9	33	0.9462	0.4799

66	Heterogeneous nuclear ribonucleoprotein U OS=Homo sapiens GN=HNRNPU	sp|Q00839|HNRPU_HUMAN	25.3	24	0.7311	0.2401
67	Spliceosome RNA helicase DDX39B OS=Homo sapiens GN=DDX39B PE=1 SV=1	sp|Q13838|DX39B_HUMAN	40.4	18	0.9036	0.7144
68	AP-2 complex subunit beta OS=Homo sapiens GN=AP2B1 PE=1 SV=1	sp|P63010|AP2B1_HUMAN	22.3	16	1.0186	0.7163
69	Golgi apparatus protein 1 OS=Homo sapiens GN=GLG1 PE=1	sp|Q92896|GSLG1_HUMAN	22.6	15	1.5996	0.1096
70	Alanine--tRNA ligase, cytoplasmic OS=Homo sapiens GN=AARS PE=1 SV=2	sp|P49588|SYAC_HUMAN	31.6	24	1.5704	0.0415
71	Eukaryotic translation initiation factor 3 subunit A OS=Homo sapiens GN=EIF3A PE=1	sp|Q14152|EIF3A_HUMAN	21.4	18	0.787	0.6013
72	Heat shock protein 105 kDa OS=Homo sapiens GN=HSPH1 PE=1	sp|Q92598|HS105_HUMAN	30.2	17	0.7178	0.2834
73	Fatty acid synthase OS=Homo sapiens GN=FASN PE=1 SV=3	sp|P49327|FAS_HUMAN	12.3	26	0.8954	0.7205
74	26S protease regulatory subunit 8 OS=Homo sapiens GN=PSMC5 PE=1	sp|P62195|PRS8_HUMAN	44.3	12	1.0375	0.6695
75	Dynamin-1-like protein OS=Homo sapiens GN=DNM1L PE=1 SV=2	sp|O00429|DNM1L_HUMAN	30.4	13	0.9908	0.9206
76	Splicing factor 3B subunit 3 OS=Homo sapiens GN=SF3B3 PE=1	sp|Q15393|SF3B3_HUMAN	20.7	13	0.7586	0.606
77	Staphylococcal nuclease domain-containing protein 1 OS=Homo sapiens GN=SND1 PE=1 SV=1	sp|Q7KZF4|SND1_HUMAN	34.3	24	1.977	0.1157
78	ATP-dependent RNA helicase A OS=Homo sapiens GN=DHX9 PE=1 SV=4	sp|Q08211|DHX9_HUMAN	19.2	19	0.6918	0.2495
79	Rab GDP dissociation inhibitor beta OS=Homo sapiens GN=GDI2 PE=1	sp|P50395|GDIB_HUMAN	51.2	20	1.0965	0.6562
80	CCR4-NOT transcription complex subunit 1 OS=Homo sapiens GN=CNOT1 PE=1 SV=2	sp|A5YKK6|CNOT1_HUMAN	9.3	14	0.7798	0.3141
81	Nucleolar protein 56 OS=Homo sapiens GN=NOP56 PE=1	sp|O00567|NOP56_HUMAN	34.5	19	0.9817	0.7205
82	Polyadenylate-binding protein 1 OS=Homo sapiens GN=PABPC1 PE=1 SV=2	sp|P11940|PABP1_HUMAN	39.6	24	0.2443	0.2095

83	Hexokinase-2 OS=Homo sapiens GN=HK2 PE=1 SV=2	sp|P52789|HXK2_HUMAN	20.3	14	1.0186	0.6931
84	Ubiquitin carboxyl-terminal hydrolase 7 OS=Homo sapiens GN=USP7 PE=1 SV=2	sp|Q93009|UBP7_HUMAN	15.7	12	1.0375	0.9714
85	FACT complex subunit SPT16 OS=Homo sapiens GN=SUPT16H PE=1 SV=1	sp|Q9Y5B9|SP16H_HUMAN	22.3	14	0.4742	0.1086
86	Leucine--tRNA ligase, cytoplasmic OS=Homo sapiens GN=LARS PE=1 SV=2	sp|Q9P2J5|SYLC_HUMAN	20.8	18	2.1478	0.0314
87	Phosphatidylinositol 3,4,5-trisphosphate 5-phosphatase 1 OS=Homo sapiens GN=INPP5D PE=1	sp|Q92835|SHIP1_HUMAN	14.8	13	1.2589	0.4701
88	Histone H2A type 2-B OS=Homo sapiens GN=HIST2H2AB	sp|Q8IUE6|H2A2B_HUMAN	69.2	61	0.3981	0.4514
89	Serine--tRNA ligase, cytoplasmic OS=Homo sapiens GN=SARS PE=1 SV=3	sp|P49591|SYSC_HUMAN	39.5	17	2.5823	0.0009
90	DNA damage-binding protein 1 OS=Homo sapiens GN=DDB1 PE=1 SV=1	sp|Q16531|DDB1_HUMAN	21.8	15	0.6792	0.0652
91	Coatomer subunit beta OS=Homo sapiens GN=COPB1 PE=1	sp|P53618|COPB_HUMAN	22.3	12	1.3062	0.5265
92	Endoplasmin OS=Homo sapiens GN=HSP90B1 PE=1 SV=1	sp|P14625|ENPL_HUMAN	30.8	31	0.9817	0.8912
93	RuvB-like 1 OS=Homo sapiens GN=RUVBL1 PE=1 SV=1	sp|Q9Y265|RUVB1_HUMAN	34.7	12	0.9727	0.9725
94	N-alpha-acetyltransferase 15, NatA auxiliary subunit OS=Homo sapiens GN=NAA15 PE=1	sp|Q9BXJ9|NAA15_HUMAN	23.8	13	0.8318	0.5294
95	Fructose-bisphosphate aldolase A OS=Homo sapiens GN=ALDOA PE=1 SV=2	sp|P04075|ALDOA_HUMAN	71.7	163	1.028	0.9316
96	D-3-phosphoglycerate dehydrogenase OS=Homo sapiens GN=PHGDH PE=1 SV=4	sp|O43175|SERA_HUMAN	31	25	1.4322	0.4071
97	26S proteasome non-ATPase regulatory subunit 1 OS=Homo sapiens GN=PSMD1 PE=1	sp|Q99460|PSMD1_HUMAN	23.3	23	1	0.8563
98	Guanine nucleotide-binding protein subunit beta-2-like 1 OS=Homo sapiens GN=GNB2L1	sp|P63244|GBLP_HUMAN	62.2	27	1.1482	0.8256

99	ATP-dependent 6-phosphofructokinase, platelet type OS=Homo sapiens GN=PFKP PE=1 SV=2	sp|Q01813|PFKAP_HUMAN	30.2	23	1.9055	0.2329
100	Valine--tRNA ligase OS=Homo sapiens GN=VARS PE=1 SV=4	sp|P26640|SYVC_HUMAN	27.5	29	1.0093	0.9759
101	Lamin-B1 OS=Homo sapiens GN=LMNB1 PE=1 SV=2	sp|P20700|LMNB1_HUMAN	30.6	15	0.8551	0.361
102	ATP-dependent RNA helicase DDX3X OS=Homo sapiens GN=DDX3X PE=1 SV=3	sp|O00571|DDX3X_HUMAN	26	17	0.6026	0.0316
103	Histone H3.2 OS=Homo sapiens GN=HIST2H3A PE=1 SV=3	sp|Q71DI3|H32_HUMAN	77.9	63	0.4875	0.4474
104	Heat shock protein HSP 90-alpha OS=Homo sapiens GN=HSP90AA1 PE=1 SV=5	sp|P07900|HS90A_HUMAN	45.6	40	1.5136	0.2886
105	Protein disulfide-isomerase A3 OS=Homo sapiens GN=PDIA3 PE=1 SV=4	sp|P30101|PDIA3_HUMAN	38.2	34	0.912	0.6304
106	Nucleoprotein TPR OS=Homo sapiens GN=TPR PE=1 SV=3	sp|P12270|TPR_HUMAN	10.8	12	1.0186	0.5159
107	40S ribosomal protein S4, X isoform OS=Homo sapiens GN=RPS4X PE=1	sp|P62701|RS4X_HUMAN	52.9	17	0.7112	0.2419
108	Threonine--tRNA ligase, cytoplasmic OS=Homo sapiens GN=TARS PE=1 SV=3	sp|P26639|SYTC_HUMAN	25.6	13	1.1482	0.36
109	Heterogeneous nuclear ribonucleoprotein Q OS=Homo sapiens GN=SYNCRIP	sp|O60506|HNRPQ_HUMAN	29.7	16	0.8472	0.5401
110	tRNA-splicing ligase RtcB homolog OS=Homo sapiens GN=RTCB PE=1	sp|Q9Y3I0|RTCB_HUMAN	28.5	12	0.8395	0.5745
111	UDP-glucose:glycoprotein glucosyltransferase 1 OS=Homo sapiens	sp|Q9NYU2|UGGG1_HUMAN	16.1	18	1.0864	0.2754
112	DNA-directed RNA polymerase II subunit RPB2 OS=Homo sapiens GN=POLR2B PE=1 SV=1	sp|P30876|RPB2_HUMAN	13.1	10	0.7656	0.4096
113	Aspartyl/asparaginyl beta-hydroxylase OS=Homo sapiens GN=ASPH PE=1 SV=3	sp|Q12797|ASPH_HUMAN	23.8	15	2.9376	0.0068
114	T-complex protein 1 subunit gamma OS=Homo sapiens GN=CCT3 PE=1 SV=4	sp|P49368|TCPG_HUMAN	39.1	23	1	0.8664

115	Heterogeneous nuclear ribonucleoprotein A3 OS=Homo sapiens GN=HNRNPA3 PE=1 SV=2	sp|P51991|ROA3_HUMAN	34.1	19	1.1376	0.8367
116	ATP-binding cassette sub-family E member 1 OS=Homo sapiens GN=ABCE1 PE=1 SV=1	sp|P61221|ABCE1_HUMAN	34.6	12	0.9727	0.8679
117	Eukaryotic translation initiation factor 2 subunit 3 OS=Homo sapiens GN=EIF2S3 PE=1	sp|P41091|IF2G_HUMAN	36.4	12	1.0471	0.6938
118	Keratin, type II cytoskeletal 1 OS=Homo sapiens GN=KRT1 PE=1 SV=6	sp|P04264|K2C1_HUMAN	33.1	12	0.5105	0.1034
119	Protein transport protein Sec23B OS=Homo sapiens GN=SEC23B PE=1 SV=2	sp|Q15437|SC23B_HUMAN	20.6	11	1.0568	0.7505
120	Tyrosine--tRNA ligase, cytoplasmic OS=Homo sapiens GN=YARS PE=1 SV=4	sp|P54577|SYYC_HUMAN	34.5	14	1.9231	0.0484
121	Protein disulfide-isomerase OS=Homo sapiens GN=P4HB PE=1 SV=3	sp|P07237|PDIA1_HUMAN	45.1	38	1.1695	0.2466
122	DNA-directed RNA polymerase II subunit RPB1 OS=Homo sapiens GN=POLR2A PE=1 SV=2	sp|P24928|RPB1_HUMAN	11	11	0.6138	0.1581
123	Sarcoplasmic/endoplasmic reticulum calcium ATPase 2 OS=Homo sapiens GN=ATP2A2 PE=1 SV=1	sp|P16615|AT2A2_HUMAN	23.1	24	1.2706	0.4659
124	Protein RRP5 homolog OS=Homo sapiens GN=PDCD11 PE=1 SV=3	sp|Q14690|RRP5_HUMAN	14.2	14	1.0093	0.7498
125	L-lactate dehydrogenase A chain OS=Homo sapiens GN=LDHA PE=1 SV=2	sp|P00338|LDHA_HUMAN	64.2	22	0.9908	0.5902
126	Eukaryotic translation initiation factor 3 subunit E OS=Homo sapiens GN=EIF3E PE=1	sp|P60228|EIF3E_HUMAN	36	14	0.7447	0.2268
127	Nucleophosmin OS=Homo sapiens GN=NPM1 PE=1 SV=2	sp|P06748|NPM_HUMAN	43.2	52	0.8318	0.4087
128	AP-2 complex subunit alpha-2 OS=Homo sapiens GN=AP2A2 PE=1 SV=2	sp|O94973|AP2A2_HUMAN	15.1	10	1.0765	0.697
129	Vigilin OS=Homo sapiens GN=HDLBP PE=1 SV=2	sp|Q00341|VIGLN_HUMAN	19.4	11	0.8091	0.4274
130	Cofilin-1 OS=Homo sapiens GN=CFL1 PE=1	sp|P23528|COF1_HUMAN	66.9	18	0.9204	0.6776

131	Vacuolar protein sorting-associated protein 35 OS=Homo sapiens GN=VPS35 PE=1 SV=2	sp|Q96QK1|VPS35_HUMAN	18.6	13	1.6749	0.1459
132	14-3-3 protein epsilon OS=Homo sapiens GN=YWHAE PE=1 SV=1	sp|P62258|1433E_HUMAN	60.4	15	1.028	0.8771
133	Protein disulfide-isomerase A6 OS=Homo sapiens GN=PDIA6 PE=1 SV=1	sp|Q15084|PDIA6_HUMAN	38	33	1.2474	0.8305
134	Importin-5 OS=Homo sapiens GN=IPO5 PE=1 SV=4	sp|O00410|IPO5_HUMAN	18.1	13	1.2246	0.6309
135	26S protease regulatory subunit 10B OS=Homo sapiens GN=PSMC6 PE=1	sp|P62333|PRS10_HUMAN	42.2	10	1.0186	0.8083
136	Structural maintenance of chromosomes protein 3 OS=Homo sapiens GN=SMC3 PE=1	sp|Q9UQE7|SMC3_HUMAN	19.6	11	0.7447	0.3047
137	Coatomer subunit beta' OS=Homo sapiens GN=COPB2 PE=1	sp|P35606|COPB2_HUMAN	18.1	11	1.0471	0.6186
138	DNA replication licensing factor MCM7 OS=Homo sapiens GN=MCM7 PE=1	sp|P33993|MCM7_HUMAN	32	14	0.5346	0.0226
139	Elongation factor 1-gamma OS=Homo sapiens GN=EEF1G PE=1	sp|P26641|EF1G_HUMAN	40.1	22	1.0568	0.5969
140	Vinculin OS=Homo sapiens GN=VCL PE=1	sp|P18206|VINC_HUMAN	20.4	10	1.2134	0.5059
141	V-type proton ATPase subunit B, brain isoform OS=Homo sapiens GN=ATP6V1B2 PE=1 SV=3	sp|P21281|VATB2_HUMAN	40.5	26	2.0893	0.0205
142	Thioredoxin-like protein 1 OS=Homo sapiens GN=TXNL1 PE=1	sp|O43396|TXNL1_HUMAN	46.7	12	0.492	0.0855
143	Transportin-1 OS=Homo sapiens GN=TNPO1 PE=1 SV=2	sp|Q92973|TNPO1_HUMAN	16.6	10	0.5808	0.1147
144	DNA replication licensing factor MCM3 OS=Homo sapiens GN=MCM3 PE=1	sp|P25205|MCM3_HUMAN	22.8	14	0.3664	0.0076
145	Transcription intermediary factor 1-beta OS=Homo sapiens GN=TRIM28 PE=1 SV=5	sp|Q13263|TIF1B_HUMAN	23.5	12	0.8241	0.4598
146	DNA replication licensing factor MCM5 OS=Homo sapiens GN=MCM5 PE=1	sp|P33992|MCM5_HUMAN	25.2	13	0.5105	0.0583
147	26S proteasome non-ATPase regulatory subunit 2 OS=Homo sapiens GN=PSMD2 PE=1	sp|Q13200|PSMD2_HUMAN	25.6	21	0.9036	0.9498

148	FACT complex subunit SSRP1 OS=Homo sapiens GN=SSRP1 PE=1 SV=1	sp|Q08945|SSRP1_HUMAN	22	11	0.7798	0.4294
149	Heterogeneous nuclear ribonucleoprotein K OS=Homo sapiens GN=HNRNPK	sp|P61978|HNRPK_HUMAN	55.1	41	0.5012	0.2992
150	Proliferation-associated protein 2G4 OS=Homo sapiens GN=PA2G4 PE=1	sp|Q9UQ80|PA2G4_HUMAN	49.8	23	0.7178	0.5061
151	26S protease regulatory subunit 6A OS=Homo sapiens GN=PSMC3 PE=1	sp|P17980|PRS6A_HUMAN	45.6	12	0.8318	0.5742
152	Dynactin subunit 1 OS=Homo sapiens GN=DCTN1 PE=1 SV=3	sp|Q14203|DCTN1_HUMAN	15.9	20	1.0765	0.6266
153	Tyrosine-protein kinase SYK OS=Homo sapiens GN=SYK PE=1 SV=1	sp|P43405|KSYK_HUMAN	30.9	13	1.3552	0.1785
154	E3 ubiquitin-protein ligase TRIP12 OS=Homo sapiens GN=TRIP12 PE=1	sp|Q14669|TRIPC_HUMAN	8.4	10	0.929	0.4266
155	Serine/threonine-protein phosphatase 2A 65 kDa regulatory subunit A alpha isoform OS=Homo sapiens GN=PPP2R1A PE=1 SV=4	sp|P30153|2AAA_HUMAN	28	15	0.9908	0.7938
156	Erythrocyte band 7 integral membrane protein OS=Homo sapiens GN=STOM PE=1 SV=3	sp|P27105|STOM_HUMAN	38.2	10	1.5136	0.4137
157	Transforming protein RhoA OS=Homo sapiens GN=RHOA PE=1 SV=1	sp|P61586|RHOA_HUMAN	67.9	12	1.3677	0.2254
158	Phosphoglycerate mutase 1 OS=Homo sapiens GN=PGAM1 PE=1 SV=2	sp|P18669|PGAM1_HUMAN	60.2	16	1.2474	0.6034
159	Exportin-T OS=Homo sapiens GN=XPOT PE=1 SV=2	sp|O43592|XPOT_HUMAN	21.3	16	1.0471	0.4748
160	N-acetyltransferase 10 OS=Homo sapiens GN=NAT10 PE=1	sp|Q9H0A0|NAT10_HUMAN	18.4	10	0.871	0.4042
161	Keratin, type I cytoskeletal 9 OS=Homo sapiens GN=KRT9 PE=1 SV=3	sp|P35527|K1C9_HUMAN	27	24	0.3499	0.0035
162	Coatomer subunit gamma-2 OS=Homo sapiens GN=COPG2 PE=1 SV=1	sp|Q9UBF2|COPG2_HUMAN	23.3	14	1.1272	0.6627
163	Dedicator of cytokinesis protein 2 OS=Homo sapiens GN=DOCK2 PE=1	sp|Q92608|DOCK2_HUMAN	10.9	10	1.2823	0.264

164	Ankyrin repeat domain-containing protein 17 OS=Homo sapiens GN=ANKRD17 PE=1 SV=3	sp|O75179|ANR17_HUMAN	7.3	11	0.863	0.3975
165	26S protease regulatory subunit 6B OS=Homo sapiens GN=PSMC4 PE=1	sp|P43686|PRS6B_HUMAN	48.8	15	1.0375	0.9176
166	60S ribosomal protein L4 OS=Homo sapiens GN=RPL4 PE=1 SV=5	sp|P36578|RL4_HUMAN	39.1	24	1.0864	0.9086
167	Chloride intracellular channel protein 4 OS=Homo sapiens GN=CLIC4 PE=1	sp|Q9Y696|CLIC4_HUMAN	48.6	9	1.4191	0.3647
168	Heterogeneous nuclear ribonucleoprotein L OS=Homo sapiens GN=HNRNPL PE=1 SV=2	sp|P14866|HNRPL_HUMAN	23.8	11	0.7798	0.4655
169	Vesicle-fusing ATPase OS=Homo sapiens GN=NSF PE=1 SV=3	sp|P46459|NSF_HUMAN	18.2	8	2.355	0.0408
170	26S proteasome non-ATPase regulatory subunit 12 OS=Homo sapiens GN=PSMD12 PE=1 SV=3	sp|O00232|PSD12_HUMAN	38.6	12	0.9727	0.9894
171	Triosephosphate isomerase OS=Homo sapiens GN=TPI1 PE=1 SV=3	sp|P60174|TPIS_HUMAN	65.4	19	1.3677	0.4394
172	Tripeptidyl-peptidase 2 OS=Homo sapiens GN=TPP2 PE=1 SV=4	sp|P29144|TPP2_HUMAN	15.9	12	1.0471	0.7755
173	Actin-related protein 3 OS=Homo sapiens GN=ACTR3 PE=1	sp|P61158|ARP3_HUMAN	43.1	12	1.4997	0.0835
174	Arginine--tRNA ligase, cytoplasmic OS=Homo sapiens GN=RARS PE=1 SV=2	sp|P54136|SYRC_HUMAN	23.8	14	1.3428	0.3826
175	Prolyl endopeptidase OS=Homo sapiens GN=PREP PE=1 SV=2	sp|P48147|PPCE_HUMAN	31	22	1.0568	0.7096
176	Pre-mRNA-splicing factor ATP-dependent RNA helicase DHX15 OS=Homo sapiens GN=DHX15 PE=1	sp|O43143|DHX15_HUMAN	26.9	14	0.9638	0.7889
177	Adenylyl cyclase-associated protein 1 OS=Homo sapiens GN=CAP1 PE=1 SV=5	sp|Q01518|CAP1_HUMAN	41.3	22	0.9908	0.9745
178	60S ribosomal protein L7a OS=Homo sapiens GN=RPL7A PE=1 SV=2	sp|P62424|RL7A_HUMAN	42.1	10	0.9638	0.7168
179	Ubiquitin carboxyl-terminal hydrolase 14 OS=Homo sapiens GN=USP14 PE=1	sp|P54578|UBP14_HUMAN	34.4	14	0.8166	0.465

180	U2 snRNP-associated SURP motif-containing protein OS=Homo sapiens GN=U2SURP PE=1 SV=2	sp|O15042|SR140_HUMAN	16.4	11	0.8954	0.7071
181	ADP/ATP translocase 2 OS=Homo sapiens GN=SLC25A5 PE=1 SV=7	sp|P05141|ADT2_HUMAN	35.9	14	1.0471	0.7762
182	GMP synthase [glutamine-hydrolyzing] OS=Homo sapiens GN=GMPS PE=1 SV=1	sp|P49915|GUAA_HUMAN	24.7	10	0.5702	0.1428
183	Vacuolar protein sorting-associated protein 13C OS=Homo sapiens GN=VPS13C PE=1 SV=1	sp|Q709C8|VP13C_HUMAN	9.9	16	1.5996	0.4801
184	Adenylosuccinate synthetase isozyme 2 OS=Homo sapiens GN=ADSS PE=1 SV=3	sp|P30520|PURA2_HUMAN	31.4	13	0.9376	0.7564
185	ATP-dependent RNA helicase DDX1 OS=Homo sapiens GN=DDX1 PE=1 SV=2	sp|Q92499|DDX1_HUMAN	23.7	13	0.9376	0.5814
186	Ras-related protein Rab-1A OS=Homo sapiens GN=RAB1A PE=1	sp|P62820|RAB1A_HUMAN	62	9	1.3305	0.5859
187	ADP-ribosylation factor 1 OS=Homo sapiens GN=ARF1 PE=1 SV=2	sp|P84077|ARF1_HUMAN	61.3	22	1.0186	0.6001
188	Puromycin-sensitive aminopeptidase OS=Homo sapiens GN=NPEPPS	sp|P55786|PSA_HUMAN	15	9	1.3552	0.1579
189	ATP-binding cassette sub-family F member 1 OS=Homo sapiens GN=ABCF1 PE=1 SV=2	sp|Q8NE71|ABCF1_HUMAN	22.7	13	0.9376	0.6367
190	Nuclear pore complex protein Nup155 OS=Homo sapiens GN=NUP155 PE=1 SV=1	sp|O75694|NU155_HUMAN	13.3	9	0.673	0.0074
191	Unconventional myosin-Va OS=Homo sapiens GN=MYO5A PE=1 SV=2	sp|Q9Y4I1|MYO5A_HUMAN	10.9	9	1.0186	0.5079
192	Monofunctional C1-tetrahydrofolate synthase, mitochondrial OS=Homo sapiens GN=MTHFD1L PE=1 SV=1	sp|Q6UB35|C1TM_HUMAN	16.2	12	2.6062	0.0505
193	Eukaryotic translation initiation factor 3 subunit B OS=Homo sapiens GN=EIF3B PE=1	sp|P55884|EIF3B_HUMAN	18.4	12	0.6918	0.4172
194	Actin-related protein 2 OS=Homo sapiens GN=ACTR2 PE=1	sp|P61160|ARP2_HUMAN	34.8	19	1.0093	0.7013

195	Probable ATP-dependent RNA helicase DDX5 OS=Homo sapiens GN=DDX5 PE=1 SV=1	sp|P17844|DDX5_HUMAN	26.6	14	0.5445	0.14
196	Ras-related protein Rab-7a OS=Homo sapiens GN=RAB7A PE=1	sp|P51149|RAB7A_HUMAN	52.2	8	1.4322	0.3877
197	Glucose-6-phosphate isomerase OS=Homo sapiens GN=GPI PE=1	sp|P06744|G6PI_HUMAN	21.7	15	1.0375	0.8728
198	UV excision repair protein RAD23 homolog B OS=Homo sapiens GN=RAD23B PE=1 SV=1	sp|P54727|RD23B_HUMAN	32.3	13	1.0666	0.9586
199	Aspartate aminotransferase, mitochondrial OS=Homo sapiens GN=GOT2 PE=1 SV=3	sp|P00505|AATM_HUMAN	38.1	18	1.1803	0.633
200	T-complex protein 1 subunit theta OS=Homo sapiens GN=CCT8 PE=1 SV=4	sp|P50990|TCPQ_HUMAN	44.7	16	1.0093	0.7236
201	T-complex protein 1 subunit epsilon OS=Homo sapiens GN=CCT5 PE=1 SV=1	sp|P48643|TCPE_HUMAN	38.8	28	1.1588	0.5474
202	Clustered mitochondria protein homolog OS=Homo sapiens GN=CLUH PE=1	sp|O75153|CLU_HUMAN	14.4	12	0.9638	0.6806
203	Nucleolar RNA helicase 2 OS=Homo sapiens GN=DDX21 PE=1 SV=5	sp|Q9NR30|DDX21_HUMAN	22.4	15	0.6792	0.3107
204	Peptidyl-prolyl cis-trans isomerase D OS=Homo sapiens GN=PPID PE=1 SV=3	sp|Q08752|PPID_HUMAN	47.6	16	0.863	0.6512
205	Major vault protein OS=Homo sapiens GN=MVP PE=1 SV=4	sp|Q14764|MVP_HUMAN	22.6	15	1.7378	0.2682
206	Macrophage-capping protein OS=Homo sapiens GN=CAPG PE=1 SV=2	sp|P40121|CAPG_HUMAN	49.1	58	1.1482	0.5014
207	Creatine kinase B-type OS=Homo sapiens GN=CKB PE=1 SV=1	sp|P12277|KCRB_HUMAN	39.6	18	0.1306	0.0467
208	Asparagine--tRNA ligase, cytoplasmic OS=Homo sapiens GN=NARS PE=1 SV=1	sp|O43776|SYNC_HUMAN	27.9	11	2.2491	0.1238
209	Cleavage and polyadenylation specificity factor subunit 1 OS=Homo sapiens GN=CPSF1 PE=1	sp|Q10570|CPSF1_HUMAN	9.5	9	0.879	0.7733
210	Eukaryotic translation initiation factor 2 subunit 1 OS=Homo sapiens GN=EIF2S1 PE=1	sp|P05198|IF2A_HUMAN	42.9	8	1.0765	0.546

211	40S ribosomal protein S9 OS=Homo sapiens GN=RPS9 PE=1 SV=3	sp|P46781|RS9_HUMAN	45.4	7	0.879	0.2393
212	Serine/threonine-protein phosphatase 2A 55 kDa regulatory subunit B alpha isoform OS=Homo sapiens GN=PPP2R2A PE=1 SV=1	sp|P63151|2ABA_HUMAN	21.5	10	1.406	0.2477
213	Cold shock domain-containing protein E1 OS=Homo sapiens GN=CSDE1 PE=1	sp|O75534|CSDE1_HUMAN	19.2	10	0.5808	0.1007
214	Chloride intracellular channel protein 1 OS=Homo sapiens GN=CLIC1 PE=1	sp|O00299|CLIC1_HUMAN	69.7	14	1.2589	0.5422
215	4-trimethylaminobutyraldehyde dehydrogenase OS=Homo sapiens GN=ALDH9A1 PE=1 SV=3	sp|P49189|AL9A1_HUMAN	39.3	17	1.0186	0.7684
216	40S ribosomal protein S3a OS=Homo sapiens GN=RPS3A PE=1 SV=2	sp|P61247|RS3A_HUMAN	37.9	24	0.929	0.3562
217	Ubiquitin-conjugating enzyme E2 K OS=Homo sapiens GN=UBE2K PE=1	sp|P61086|UBE2K_HUMAN	54	11	1.0186	0.7616
218	Brefeldin A-inhibited guanine nucleotide-exchange protein 2 OS=Homo sapiens GN=ARFGEF2 PE=1 SV=3	sp|Q9Y6D5|BIG2_HUMAN	10.4	9	1.2706	0.5735
219	Prelamin-A/C OS=Homo sapiens GN=LMNA PE=1 SV=1	sp|P02545|LMNA_HUMAN	27.7	13	0.871	0.9728
220	40S ribosomal protein SA OS=Homo sapiens GN=RPSA PE=1 SV=4	sp|P08865|RSSA_HUMAN	49.2	14	0.7516	0.9212
221	26S protease regulatory subunit 4 OS=Homo sapiens GN=PSMC1 PE=1	sp|P62191|PRS4_HUMAN	38.4	11	0.9727	0.9041
222	Insulin-degrading enzyme OS=Homo sapiens GN=IDE PE=1 SV=4	sp|P14735|IDE_HUMAN	18.9	11	1.0471	0.5659
223	UDP-N-acetylglucosamine--peptide N-acetylglucosaminyltransferase 110 kDa subunit OS=Homo sapiens GN=OGT PE=1 SV=3	sp|O15294|OGT1_HUMAN	16.2	9	0.7727	0.313
224	Exportin-7 OS=Homo sapiens GN=XPO7 PE=1 SV=3	sp|Q9UIA9|XPO7_HUMAN	17	10	0.9638	0.617
225	6-phosphogluconate dehydrogenase, decarboxylating OS=Homo sapiens GN=PGD PE=1	sp|P52209|6PGD_HUMAN	32.3	34	1.3932	0.4311

226	Transketolase OS=Homo sapiens GN=TKT PE=1 SV=3	sp|P29401|TKT_HUMAN	32.7	35	1.0471	0.6614
227	Peptidyl-prolyl cis-trans isomerase A OS=Homo sapiens GN=PPIA PE=1 SV=2	sp|P62937|PPIA_HUMAN	59.4	66	0.9727	0.8422
228	Protein flightless-1 homolog OS=Homo sapiens GN=FLII PE=1	sp|Q13045|FLII_HUMAN	10.8	9	1.4859	0.39
229	Aconitate hydratase, mitochondrial OS=Homo sapiens GN=ACO2 PE=1	sp|Q99798|ACON_HUMAN	22.2	10	1.0568	0.6697
230	DNA replication licensing factor MCM2 OS=Homo sapiens GN=MCM2 PE=1	sp|P49736|MCM2_HUMAN	21.4	12	0.4571	0.0328
231	Profilin-1 OS=Homo sapiens GN=PFN1 PE=1 SV=2	sp|P07737|PROF1_HUMAN	83.6	60	0.955	0.7216
232	Tyrosine-protein phosphatase non-receptor type 6 OS=Homo sapiens GN=PTPN6 PE=1 SV=1	sp|P29350|PTN6_HUMAN	33.3	15	0.9638	0.7324
233	Probable ATP-dependent RNA helicase DDX46 OS=Homo sapiens GN=DDX46 PE=1 SV=2	sp|Q7L014|DDX46_HUMAN	13	8	0.8472	0.7458
234	Succinate dehydrogenase [ubiquinone] flavoprotein subunit, mitochondrial OS=Homo sapiens GN=SDHA PE=1	sp|P31040|SDHA_HUMAN	26.4	18	1.5136	0.7552
235	Heterogeneous nuclear ribonucleoproteins A2/B1 OS=Homo sapiens GN=HNRNPA2B1 PE=1 SV=2	sp|P22626|ROA2_HUMAN	52.7	12	1.1803	0.9255
236	Polypyrimidine tract-binding protein 1 OS=Homo sapiens GN=PTBP1 PE=1	sp|P26599|PTBP1_HUMAN	36.2	14	0.9638	0.9971
237	Serine/threonine-protein kinase PAK 2 OS=Homo sapiens GN=PAK2 PE=1 SV=3	sp|Q13177|PAK2_HUMAN	26.3	9	0.9376	0.9145
238	CTP synthase 1 OS=Homo sapiens GN=CTPS1 PE=1 SV=2	sp|P17812|PYRG1_HUMAN	24.2	10	1.0375	0.9019
239	Glutaredoxin-3 OS=Homo sapiens GN=GLRX3 PE=1 SV=2	sp|O76003|GLRX3_HUMAN	32.2	9	0.7516	0.4078
240	DNA replication licensing factor MCM4 OS=Homo sapiens GN=MCM4 PE=1	sp|P33991|MCM4_HUMAN	21.9	10	0.6194	0.4341
241	40S ribosomal protein S15a OS=Homo sapiens GN=RPS15A PE=1 SV=2	sp|P62244|RS15A_HUMAN	50.8	12	1.1066	0.5466

242	Host cell factor 1 OS=Homo sapiens GN=HCFC1 PE=1 SV=2	sp|P51610|HCFC1_HUMAN	7.9	13	0.7943	0.4284
243	Heat shock 70 kDa protein 4L OS=Homo sapiens GN=HSPA4L PE=1 SV=3	sp|O95757|HS74L_HUMAN	30	21	1.9588	0.0898
244	Structural maintenance of chromosomes protein 2 OS=Homo sapiens GN=SMC2 PE=1	sp|O95347|SMC2_HUMAN	16.5	9	0.4571	0.096
245	Splicing factor 3B subunit 2 OS=Homo sapiens GN=SF3B2 PE=1	sp|Q13435|SF3B2_HUMAN	22.8	8	0.7112	0.306
246	60S ribosomal protein L7 OS=Homo sapiens GN=RPL7 PE=1 SV=1	sp|P18124|RL7_HUMAN	47.6	10	0.9638	0.8908
247	Eukaryotic translation initiation factor 4 gamma 1 OS=Homo sapiens GN=EIF4G1 PE=1	sp|Q04637|IF4G1_HUMAN	17.1	9	0.6194	0.1731
248	3-ketoacyl-CoA thiolase, peroxisomal OS=Homo sapiens GN=ACAA1 PE=1 SV=2	sp|P09110|THIK_HUMAN	31.8	12	0.9817	0.9558
249	Large proline-rich protein BAG6 OS=Homo sapiens GN=BAG6 PE=1 SV=2	sp|P46379|BAG6_HUMAN	19.4	9	0.6607	0.3373
250	Poly(rC)-binding protein 1 OS=Homo sapiens GN=PCBP1 PE=1	sp|Q15365|PCBP1_HUMAN	43.8	17	0.6546	0.32
251	Transcription activator BRG1 OS=Homo sapiens GN=SMARCA4 PE=1 SV=2	sp|P51532|SMCA4_HUMAN	9.2	11	0.6546	0.4048
252	Nucleosome assembly protein 1-like 1 OS=Homo sapiens GN=NAP1L1 PE=1 SV=1	sp|P55209|NP1L1_HUMAN	27.1	15	0.8318	0.6474
253	Proteasome subunit alpha type-7 OS=Homo sapiens GN=PSMA7 PE=1 SV=1	sp|O14818|PSA7_HUMAN	56.1	7	0.9908	0.8088
254	ATP-dependent 6-phosphofructokinase, liver type OS=Homo sapiens GN=PFKL PE=1 SV=6	sp|P17858|PFKAL_HUMAN	21.5	16	1.888	0.327
255	Catalase OS=Homo sapiens GN=CAT PE=1 SV=3	sp|P04040|CATA_HUMAN	29	14	2.1086	0.0823
256	Glutaminase kidney isoform, mitochondrial OS=Homo sapiens GN=GLS PE=1 SV=1	sp|O94925|GLSK_HUMAN	21.2	8	1.3428	0.2294
257	Radixin OS=Homo sapiens GN=RDX PE=1 SV=1	sp|P35241|RADI_HUMAN	34	23	0.9727	0.9121
258	Splicing factor 1 OS=Homo sapiens GN=SF1 PE=1 SV=4	sp|Q15637|SF01_HUMAN	23.8	8	0.7727	0.698

259	26S proteasome non-ATPase regulatory subunit 6 OS=Homo sapiens GN=PSMD6 PE=1	sp|Q15008|PSMD6_HUMAN	26	11	1.0471	0.7552
260	Importin-9 OS=Homo sapiens GN=IPO9 PE=1 SV=3	sp|Q96P70|IPO9_HUMAN	14	8	1.0093	0.841
261	Plasminogen activator inhibitor 1 RNA-binding protein OS=Homo sapiens GN=SERBP1 PE=1	sp|Q8NC51|PAIRB_HUMAN	27.2	20	1.0471	0.967
262	Probable ATP-dependent RNA helicase DDX6 OS=Homo sapiens GN=DDX6 PE=1 SV=2	sp|P26196|DDX6_HUMAN	24	13	0.6918	0.2298
263	Delta-1-pyrroline-5-carboxylate synthase OS=Homo sapiens GN=ALDH18A1 PE=1 SV=2	sp|P54886|P5CS_HUMAN	13.2	7	1.7061	0.1561
264	Glutamine--fructose-6-phosphate aminotransferase [isomerizing] 1 OS=Homo sapiens GN=GFPT1 PE=1 SV=3	sp|Q06210|GFPT1_HUMAN	22	7	1.0375	0.7471
265	Ras-related protein Rap-1A OS=Homo sapiens GN=RAP1A PE=1	sp|P62834|RAP1A_HUMAN	60.9	10	1.3677	0.6388
266	Ras-related protein Rab-2A OS=Homo sapiens GN=RAB2A PE=1	sp|P61019|RAB2A_HUMAN	42.9	9	1	0.9309
267	Serine/threonine-protein kinase mTOR OS=Homo sapiens GN=MTOR PE=1	sp|P42345|MTOR_HUMAN	7.5	9	1.0375	0.5717
268	Baculoviral IAP repeat-containing protein 6 OS=Homo sapiens GN=BIRC6 PE=1	sp|Q9NR09|BIRC6_HUMAN	6.9	17	0.929	0.4319
269	Transportin-3 OS=Homo sapiens GN=TNPO3 PE=1 SV=3	sp|Q9Y5L0|TNPO3_HUMAN	17	9	0.6368	0.6803
270	Regulator of nonsense transcripts 1 OS=Homo sapiens GN=UPF1 PE=1	sp|Q92900|RENT1_HUMAN	19.8	12	0.5105	0.0723
271	40S ribosomal protein S5 OS=Homo sapiens GN=RPS5 PE=1 SV=4	sp|P46782|RS5_HUMAN	38.2	33	0.8551	0.6511
272	Heat shock protein 75 kDa, mitochondrial OS=Homo sapiens GN=TRAP1 PE=1 SV=3	sp|Q12931|TRAP1_HUMAN	19.9	12	0.9908	0.977
273	60S ribosomal protein L10 OS=Homo sapiens GN=RPL10 PE=1 SV=4	sp|P27635|RL10_HUMAN	45.3	23	0.879	0.8026
274	Ubiquitin conjugation factor E4 B OS=Homo sapiens GN=UBE4B PE=1	sp|O95155|UBE4B_HUMAN	13.1	9	0.9204	0.5211

275	Proteasome subunit beta type-5 OS=Homo sapiens GN=PSMB5 PE=1 SV=3	sp|P28074|PSB5_HUMAN	42.6	8	1.0375	0.5588
276	COP9 signalosome complex subunit 2 OS=Homo sapiens GN=COPS2 PE=1 SV=1	sp|P61201|CSN2_HUMAN	27.5	9	0.9817	0.6048
277	Adenylosuccinate lyase OS=Homo sapiens GN=ADSL PE=1 SV=2	sp|P30566|PUR8_HUMAN	21.9	10	0.9376	0.5302
278	Splicing factor 3A subunit 1 OS=Homo sapiens GN=SF3A1 PE=1	sp|Q15459|SF3A1_HUMAN	14.5	11	0.7178	0.6929
279	Pre-mRNA-processing factor 40 homolog A OS=Homo sapiens GN=PRPF40A PE=1 SV=2	sp|O75400|PR40A_HUMAN	12.5	15	0.9204	0.8464
280	Aspartate--tRNA ligase, cytoplasmic OS=Homo sapiens GN=DARS PE=1 SV=2	sp|P14868|SYDC_HUMAN	23	7	1.2823	0.6099
281	Calmodulin OS=Homo sapiens GN=CALM1 PE=1 SV=2	sp|P62158|CALM_HUMAN	62.4	12	0.7047	0.5648
282	Heterogeneous nuclear ribonucleoprotein U-like protein 2 OS=Homo sapiens GN=HNRNPUL2 PE=1 SV=1	sp|Q1KMD3|HNRL2_HUMAN	16.3	10	0.9908	0.9677
283	ATP-binding cassette sub-family D member 3 OS=Homo sapiens GN=ABCD3 PE=1 SV=1	sp|P28288|ABCD3_HUMAN	21.1	8	1.2942	0.2378
284	SWI/SNF-related matrix-associated actin-dependent regulator of chromatin subfamily A member 5 OS=Homo sapiens GN=SMARCA5 PE=1 SV=1	sp|O60264|SMCA5_HUMAN	12.2	7	0.52	0.0779
285	DNA (cytosine-5)-methyltransferase 1 OS=Homo sapiens GN=DNMT1 PE=1 SV=2	sp|P26358|DNMT1_HUMAN	12.1	8	0.1803	0.022
286	Phosphoglucomutase-1 OS=Homo sapiens GN=PGM1 PE=1 SV=3	sp|P36871|PGM1_HUMAN	23.7	7	1.406	0.5151
287	Multifunctional protein ADE2 OS=Homo sapiens GN=PAICS PE=1 SV=3	sp|P22234|PUR6_HUMAN	35.3	13	0.7178	0.4555
288	Nuclear pore complex protein Nup160 OS=Homo sapiens GN=NUP160 PE=1 SV=3	sp|Q12769|NU160_HUMAN	10.9	11	0.9462	0.7478
289	Glutamate dehydrogenase 1, mitochondrial OS=Homo sapiens GN=GLUD1 PE=1 SV=2	sp|P00367|DHE3_HUMAN	35.7	14	1.2474	0.3983

290	Nucleolar protein 58 OS=Homo sapiens GN=NOP58 PE=1	sp|Q9Y2X3|NOP58_HUMAN	26.7	9	1.2823	0.9554
291	Proto-oncogene vav OS=Homo sapiens GN=VAV1 PE=1 SV=4	sp|P15498|VAV_HUMAN	18.3	10	2.3335	0.2485
292	Protein RCC2 OS=Homo sapiens GN=RCC2 PE=1 SV=2	sp|Q9P258|RCC2_HUMAN	31.2	13	0.6252	0.2804
293	T-complex protein 1 subunit zeta OS=Homo sapiens GN=CCT6A PE=1 SV=3	sp|P40227|TCPZ_HUMAN	30.5	12	1.0568	0.8672
294	40S ribosomal protein S7 OS=Homo sapiens GN=RPS7 PE=1 SV=1	sp|P62081|RS7_HUMAN	61.9	10	0.597	0.1797
295	Pogo transposable element with ZNF domain OS=Homo sapiens GN=POGZ PE=1 SV=2	sp|Q7Z3K3|POGZ_HUMAN	7	7	0.7311	0.4682
296	Bromodomain adjacent to zinc finger domain protein 1A OS=Homo sapiens GN=BAZ1A PE=1 SV=2	sp|Q9NRL2|BAZ1A_HUMAN	9.1	7	0.9376	0.9311
297	ATP-dependent RNA helicase DDX39A OS=Homo sapiens GN=DDX39A PE=1 SV=2	sp|O00148|DX39A_HUMAN	28.8	15	0.6427	0.5946
298	Thimet oligopeptidase OS=Homo sapiens GN=THOP1 PE=1 SV=2	sp|P52888|THOP1_HUMAN	24.1	11	0.9817	0.8943
299	Asparagine synthetase [glutamine-hydrolyzing] OS=Homo sapiens GN=ASNS PE=1 SV=4	sp|P08243|ASNS_HUMAN	22.5	14	1.8707	0.2855
300	Intersectin-2 OS=Homo sapiens GN=ITSN2 PE=1 SV=3	sp|Q9NZM3|ITSN2_HUMAN	8.7	7	0.8395	0.4942
301	Protein arginine N-methyltransferase 5 OS=Homo sapiens GN=PRMT5 PE=1 SV=4	sp|O14744|ANM5_HUMAN	20.7	12	1.0186	0.7837
302	Prohibitin-2 OS=Homo sapiens GN=PHB2 PE=1 SV=2	sp|Q99623|PHB2_HUMAN	46.2	11	1.1272	0.6795
303	Transcription elongation factor SPT6 OS=Homo sapiens GN=SUPT6H PE=1 SV=2	sp|Q7KZ85|SPT6H_HUMAN	8.1	8	0.5808	0.0652
304	Long-chain-fatty-acid--CoA ligase 4 OS=Homo sapiens GN=ACSL4 PE=1 SV=2	sp|O60488|ACSL4_HUMAN	20.1	12	1.2706	0.4898
305	60S ribosomal protein L21 OS=Homo sapiens GN=RPL21 PE=1 SV=2	sp|P46778|RL21_HUMAN	42.5	15	0.6427	0.0831

306	Serine hydroxymethyltransferase, mitochondrial OS=Homo sapiens GN=SHMT2 PE=1 SV=3	sp|P34897|GLYM_HUMAN	37.5	15	1.5704	0.3022
307	U4/U6 small nuclear ribonucleoprotein Prp4 OS=Homo sapiens	sp|O43172|PRP4_HUMAN	23.4	8	0.7656	0.5135
308	Heterogeneous nuclear ribonucleoprotein M OS=Homo sapiens GN=HNRNPM	sp|P52272|HNRPM_HUMAN	20.6	13	0.7656	0.3274
309	Serine/threonine-protein phosphatase PP1-beta catalytic subunit OS=Homo sapiens GN=PPP1CB PE=1 SV=3	sp|P62140|PP1B_HUMAN	33.6	7	0.9908	0.944
310	Nodal modulator 1 OS=Homo sapiens GN=NOMO1 PE=1 SV=5	sp|Q15155|NOMO1_HUMAN	15.6	9	1.6749	0.5034
311	NADH dehydrogenase [ubiquinone] flavoprotein 1, mitochondrial OS=Homo sapiens GN=NDUFV1 PE=1 SV=4	sp|P49821|NDUV1_HUMAN	27.6	8	0.9908	0.8418
312	Inositol 1,4,5-trisphosphate receptor type 2 OS=Homo sapiens GN=ITPR2 PE=1 SV=2	sp|Q14571|ITPR2_HUMAN	6.3	8	1.1376	0.4221
313	Mitochondrial 10-formyltetrahydrofolate dehydrogenase OS=Homo sapiens GN=ALDH1L2 PE=1 SV=2	sp|Q3SY69|AL1L2_HUMAN	17.4	12	2.1478	0.2947
314	Protein SET OS=Homo sapiens GN=SET PE=1 SV=3	sp|Q01105|SET_HUMAN	34.1	14	0.6486	0.3678
315	RRP12-like protein OS=Homo sapiens GN=RRP12 PE=1 SV=2	sp|Q5JTH9|RRP12_HUMAN	12	6	0.9204	0.6548
316	EH domain-containing protein 4 OS=Homo sapiens GN=EHD4 PE=1 SV=1	sp|Q9H223|EHD4_HUMAN	28.1	6	0.8091	0.5633
317	Non-POU domain-containing octamer-binding protein OS=Homo sapiens GN=NONO PE=1	sp|Q15233|NONO_HUMAN	21.7	16	0.7727	0.2647
318	Keratin, type I cytoskeletal 10 OS=Homo sapiens GN=KRT10 PE=1 SV=6	sp|P13645|K1C10_HUMAN	26.7	7	0.8166	0.4912
319	Protein phosphatase 1 regulatory subunit 7 OS=Homo sapiens	sp|Q15435|PP1R7_HUMAN	26.7	7	0.955	0.7918
320	Splicing factor, proline- and glutamine-rich OS=Homo sapiens GN=SFPQ PE=1 SV=2	sp|P23246|SFPQ_HUMAN	21.9	9	0.6486	0.0732

321	Probable ATP-dependent RNA helicase DDX23 OS=Homo sapiens GN=DDX23 PE=1 SV=3	sp|Q9BUQ8|DDX23_HUMAN	14.3	8	0.955	0.9695
322	Aldehyde dehydrogenase, mitochondrial OS=Homo sapiens GN=ALDH2 PE=1 SV=2	sp|P05091|ALDH2_HUMAN	24.8	23	1.0093	0.9734
323	Histone H4 OS=Homo sapiens GN=HIST1H4A PE=1 SV=2	sp|P62805|H4_HUMAN	69.9	25	0.138	0.0973
324	Hypoxanthine-guanine phosphoribosyltransferase OS=Homo sapiens GN=HPRT1 PE=1	sp|P00492|HPRT_HUMAN	39.9	8	0.879	0.768
325	Dolichyl-diphosphooligosaccharide--protein glycosyltransferase subunit 1 OS=Homo sapiens GN=RPN1 PE=1	sp|P04843|RPN1_HUMAN	24.6	11	1.1695	0.5388
326	Dolichyl-diphosphooligosaccharide--protein glycosyltransferase 48 kDa subunit OS=Homo sapiens GN=DDOST PE=1 SV=4	sp|P39656|OST48_HUMAN	25.2	9	1.1803	0.5895
327	Stress-induced-phosphoprotein 1 OS=Homo sapiens GN=STIP1 PE=1 SV=1	sp|P31948|STIP1_HUMAN	19	7	0.9376	0.9549
328	Superkiller viralicidic activity 2-like 2 OS=Homo sapiens GN=SKIV2L2 PE=1 SV=3	sp|P42285|SK2L2_HUMAN	11.8	7	0.8395	0.6345
329	Importin subunit alpha-1 OS=Homo sapiens GN=KPNA2 PE=1 SV=1	sp|P52292|IMA1_HUMAN	25.3	7	0.207	0.0696
330	26S protease regulatory subunit 7 OS=Homo sapiens GN=PSMC2 PE=1	sp|P35998|PRS7_HUMAN	40.4	9	1	0.8756
331	HEAT repeat-containing protein 1 OS=Homo sapiens GN=HEATR1	sp|Q9H583|HEAT1_HUMAN	9	6	0.9376	0.8234
332	Very-long-chain enoyl-CoA reductase OS=Homo sapiens GN=TECR PE=1 SV=1	sp|Q9NZ01|TECR_HUMAN	20.8	7	1.0666	0.5427
333	Ubiquitin thioesterase OTUB1 OS=Homo sapiens GN=OTUB1 PE=1	sp|Q96FW1|OTUB1_HUMAN	25.8	7	1.4997	0.4289
334	Transcription elongation factor A protein 1 OS=Homo sapiens GN=TCEA1 PE=1	sp|P23193|TCEA1_HUMAN	27.6	6	0.9638	0.7668

335	Inosine-5'-monophosphate dehydrogenase 2 OS=Homo sapiens GN=IMPDH2 PE=1 SV=2	sp|P12268|IMDH2_HUMAN	22.2	6	0.5248	0.2696
336	Trifunctional purine biosynthetic protein adenosine-3 OS=Homo sapiens GN=GART PE=1 SV=1	sp|P22102|PUR2_HUMAN	16.1	11	0.6026	0.3087
337	Adenylate kinase 2, mitochondrial OS=Homo sapiens GN=AK2 PE=1 SV=2	sp|P54819|KAD2_HUMAN	41.4	15	0.9462	0.8576
338	Tubulin beta-6 chain OS=Homo sapiens GN=TUBB6 PE=1	sp|Q9BUF5|TBB6_HUMAN	44	28	0.9638	0.7044
339	Eukaryotic translation initiation factor 3 subunit C OS=Homo sapiens GN=EIF3C PE=1	sp|Q99613|EIF3C_HUMAN	14	7	0.929	0.4975
340	Cytochrome b-245 heavy chain OS=Homo sapiens GN=CYBB PE=1 SV=2	sp|P04839|CY24B_HUMAN	15.3	8	1.1376	0.7029
341	Signal recognition particle receptor subunit alpha OS=Homo sapiens GN=SRPR PE=1 SV=2	sp|P08240|SRPR_HUMAN	16.8	10	1.4454	0.457
342	Malate dehydrogenase, mitochondrial OS=Homo sapiens GN=MDH2 PE=1 SV=3	sp|P40926|MDHM_HUMAN	32.3	20	1.2134	0.5446
343	Enhancer of mRNA-decapping protein 4 OS=Homo sapiens GN=EDC4 PE=1	sp|Q6P2E9|EDC4_HUMAN	9.6	10	1.0666	0.9723
344	26S proteasome non-ATPase regulatory subunit 7 OS=Homo sapiens GN=PSMD7 PE=1	sp|P51665|PSMD7_HUMAN	33.3	9	0.9908	0.9184
345	Guanine nucleotide-binding protein G(i) subunit alpha-2 OS=Homo sapiens GN=GNAI2 PE=1	sp|P04899|GNAI2_HUMAN	38.3	16	1.0666	0.5624
346	AP-1 complex subunit mu-1 OS=Homo sapiens GN=AP1M1 PE=1 SV=3	sp|Q9BXS5|AP1M1_HUMAN	24.8	6	1.1912	0.7507
347	Voltage-dependent anion-selective channel protein 3 OS=Homo sapiens GN=VDAC3 PE=1 SV=1	sp|Q9Y277|VDAC3_HUMAN	31.5	9	1.6144	0.186
348	Ras-related protein Rab-5C OS=Homo sapiens GN=RAB5C PE=1	sp|P51148|RAB5C_HUMAN	51.9	8	0.912	0.8006
349	Spermidine synthase OS=Homo sapiens GN=SRM PE=1 SV=1	sp|P19623|SPEE_HUMAN	35.1	15	0.9462	0.7915

350	40S ribosomal protein S17-like OS=Homo sapiens GN=RPS17L PE=1 SV=1	sp|P0CW22|RS17L_HUMAN	51.1	13	1.0093	0.8696
351	Developmentally-regulated GTP-binding protein 1 OS=Homo sapiens GN=DRG1 PE=1 SV=1	sp|Q9Y295|DRG1_HUMAN	22.1	6	0.673	0.2619
352	Serine/threonine-protein kinase 4 OS=Homo sapiens GN=STK4 PE=1 SV=2	sp|Q13043|STK4_HUMAN	16.4	10	0.9817	0.9769
353	Phosphoribosyl pyrophosphate synthase-associated protein 2 OS=Homo sapiens GN=PRPSAP2 PE=1 SV=1	sp|O60256|KPRB_HUMAN	18.7	8	0.9817	0.9929
354	Histone acetyltransferase type B catalytic subunit OS=Homo sapiens GN=HAT1 PE=1 SV=1	sp|O14929|HAT1_HUMAN	21	6	0.5152	0.1545
355	ATP-dependent RNA helicase DDX19A OS=Homo sapiens GN=DDX19A PE=1 SV=1	sp|Q9NUU7|DD19A_HUMAN	19.3	6	0.929	0.6089
356	Nucleolin OS=Homo sapiens GN=NCL PE=1 SV=3	sp|P19338|NUCL_HUMAN	24.1	15	0.9908	0.7756
357	Plexin-B2 OS=Homo sapiens GN=PLXNB2 PE=1 SV=3	sp|O15031|PLXB2_HUMAN	8.7	7	1.1376	0.5234
358	Basic leucine zipper and W2 domain-containing protein 1 OS=Homo sapiens GN=BZW1 PE=1	sp|Q7L1Q6|BZW1_HUMAN	23.6	9	0.6855	0.475
359	40S ribosomal protein S16 OS=Homo sapiens GN=RPS16 PE=1 SV=2	sp|P62249|RS16_HUMAN	45.2	8	0.7112	0.3552
360	Far upstream element-binding protein 2 OS=Homo sapiens GN=KHSRP PE=1	sp|Q92945|FUBP2_HUMAN	12.2	8	0.6427	0.2588
361	Glucose-6-phosphate 1-dehydrogenase OS=Homo sapiens GN=G6PD PE=1 SV=4	sp|P11413|G6PD_HUMAN	25.1	10	1.888	0.0977
362	Protein O-GlcNAcase OS=Homo sapiens GN=MGEA5 PE=1 SV=2	sp|O60502|OGA_HUMAN	15.9	8	1.0375	0.7859
363	Tyrosine-protein kinase Lyn OS=Homo sapiens GN=LYN PE=1 SV=3	sp|P07948|LYN_HUMAN	28.7	10	1.0186	0.8798
364	2-oxoglutarate dehydrogenase, mitochondrial OS=Homo sapiens GN=OGDH PE=1 SV=3	sp|Q02218|ODO1_HUMAN	15.6	9	1.1695	0.5489

365	14-3-3 protein gamma OS=Homo sapiens GN=YWHAG PE=1 SV=2	sp|P61981|1433G_HUMAN	51	20	2.2284	0.0865
366	Leucine-rich PPR motif-containing protein, mitochondrial OS=Homo sapiens GN=LRPPRC PE=1 SV=3	sp|P42704|LPPRC_HUMAN	10	8	0.7178	0.5631
367	Beta-adrenergic receptor kinase 1 OS=Homo sapiens GN=ADRBK1 PE=1 SV=2	sp|P25098|ARBK1_HUMAN	15.2	7	0.5808	0.1711
368	Developmentally-regulated GTP-binding protein 2 OS=Homo sapiens GN=DRG2 PE=1 SV=1	sp|P55039|DRG2_HUMAN	24.5	6	0.9376	0.768
369	60S ribosomal protein L5 OS=Homo sapiens GN=RPL5 PE=1 SV=3	sp|P46777|RL5_HUMAN	30	18	0.8318	0.52
370	NADH-ubiquinone oxidoreductase 75 kDa subunit, mitochondrial OS=Homo sapiens GN=NDUFS1 PE=1 SV=3	sp|P28331|NDUS1_HUMAN	12.1	6	1.0765	0.726
371	Protein arginine N-methyltransferase 1 OS=Homo sapiens GN=PRMT1 PE=1 SV=2	sp|Q99873|ANM1_HUMAN	30.5	7	0.787	0.4522
372	Lon protease homolog, mitochondrial OS=Homo sapiens GN=LONP1 PE=1 SV=2	sp|P36776|LONM_HUMAN	15.9	9	2.0324	0.3116
373	Pre-mRNA-processing factor 6 OS=Homo sapiens GN=PRPF6 PE=1 SV=1	sp|O94906|PRP6_HUMAN	18.3	6	0.8166	0.994
374	La-related protein 1 OS=Homo sapiens GN=LARP1 PE=1 SV=2	sp|Q6PKG0|LARP1_HUMAN	12.1	6	0.9908	0.8938
375	40S ribosomal protein S18 OS=Homo sapiens GN=RPS18 PE=1 SV=3	sp|P62269|RS18_HUMAN	42.8	8	0.6982	0.0525
376	Histidine--tRNA ligase, cytoplasmic OS=Homo sapiens GN=HARS PE=1 SV=2	sp|P12081|SYHC_HUMAN	26.3	15	1.3677	0.5808
377	Actin-related protein 2/3 complex subunit 2 OS=Homo sapiens GN=ARPC2 PE=1	sp|O15144|ARPC2_HUMAN	45.3	13	0.9908	0.8035
378	Vam6/Vps39-like protein OS=Homo sapiens GN=VPS39 PE=1	sp|Q96JC1|VPS39_HUMAN	12.9	7	1.6444	0.2347
379	Nuclear protein localization protein 4 homolog OS=Homo sapiens GN=NPLOC4 PE=1 SV=3	sp|Q8TAT6|NPL4_HUMAN	14.3	6	1.1066	0.37

380	Nuclear pore complex protein Nup205 OS=Homo sapiens GN=NUP205 PE=1 SV=3	sp|Q92621|NU205_HUMAN	8.5	7	0.9036	0.7951
381	Heterogeneous nuclear ribonucleoprotein A1 OS=Homo sapiens GN=HNRNPA1 PE=1 SV=5	sp|P09651|ROA1_HUMAN	32	12	0.871	0.2691
382	Annexin A5 OS=Homo sapiens GN=ANXA5 PE=1 SV=2	sp|P08758|ANXA5_HUMAN	35	12	2.7797	0.0389
383	Regulation of nuclear pre-mRNA domain-containing protein 1B OS=Homo sapiens GN=RPRD1B PE=1 SV=1	sp|Q9NQG5|RPR1B_HUMAN	26.7	6	0.9462	0.7826
384	40S ribosomal protein S2 OS=Homo sapiens GN=RPS2 PE=1 SV=2	sp|P15880|RS2_HUMAN	33.5	10	0.9462	0.8173
385	Neutral alpha-glucosidase AB OS=Homo sapiens GN=GANAB PE=1 SV=3	sp|Q14697|GANAB_HUMAN	9.6	5	1.0965	0.748
386	Exportin-2 OS=Homo sapiens GN=CSE1L PE=1 SV=3	sp|P55060|XPO2_HUMAN	16.8	32	0.7047	0.2072
387	FAS-associated factor 1 OS=Homo sapiens GN=FAF1 PE=1 SV=2	sp|Q9UNN5|FAF1_HUMAN	13.7	5	1.0568	0.4308
388	Serrate RNA effector molecule homolog OS=Homo sapiens GN=SRRT PE=1	sp|Q9BXP5|SRRT_HUMAN	15.3	7	0.5649	0.4823
389	Succinyl-CoA ligase [GDP-forming] subunit beta, mitochondrial OS=Homo sapiens GN=SUCLG2 PE=1 SV=2	sp|Q96I99|SUCB2_HUMAN	29.2	7	1.2134	0.582
390	Phosphoserine phosphatase OS=Homo sapiens GN=PSPH PE=1 SV=2	sp|P78330|SERB_HUMAN	55.1	8	1.6444	0.2428
391	60S ribosomal protein L18a OS=Homo sapiens GN=RPL18A PE=1 SV=2	sp|Q02543|RL18A_HUMAN	32.4	5	1.0568	0.8841
392	Rabankyrin-5 OS=Homo sapiens GN=ANKFY1 PE=1 SV=2	sp|Q9P2R3|ANFY1_HUMAN	11.2	8	1.1272	0.8084
393	NADPH--cytochrome P450 reductase OS=Homo sapiens GN=POR PE=1 SV=2	sp|P16435|NCPR_HUMAN	21.3	12	1.6444	0.1853
394	NAD(P) transhydrogenase, mitochondrial OS=Homo sapiens GN=NNT PE=1 SV=3	sp|Q13423|NNTM_HUMAN	9	6	1.2134	0.6309
395	Annexin A7 OS=Homo sapiens GN=ANXA7 PE=1 SV=3	sp|P20073|ANXA7_HUMAN	18.9	7	1.803	0.092

396	Squamous cell carcinoma antigen recognized by T-cells 3 OS=Homo sapiens GN=SART3 PE=1	sp|Q15020|SART3_HUMAN	13.9	10	0.7112	0.3549
397	Leukotriene A-4 hydrolase OS=Homo sapiens GN=LTA4H PE=1 SV=2	sp|P09960|LKHA4_HUMAN	18.7	9	0.9376	0.7814
398	Isoleucine--tRNA ligase, cytoplasmic OS=Homo sapiens GN=IARS PE=1 SV=2	sp|P41252|SYIC_HUMAN	11.4	7	1.7701	0.5852
399	Ras-related protein Rab-14 OS=Homo sapiens GN=RAB14 PE=1	sp|P61106|RAB14_HUMAN	48.4	7	1.2134	0.6491
400	Dihydrolipoyl dehydrogenase, mitochondrial OS=Homo sapiens GN=DLD PE=1 SV=2	sp|P09622|DLDH_HUMAN	34	38	1.6904	0.2214
401	DnaJ homolog subfamily C member 11 OS=Homo sapiens GN=DNAJC11 PE=1 SV=2	sp|Q9NVH1|DJC11_HUMAN	17	6	1	0.9524
402	Cysteine--tRNA ligase, cytoplasmic OS=Homo sapiens GN=CARS PE=1 SV=3	sp|P49589|SYCC_HUMAN	17.5	13	1.5417	0.2644
403	Dolichyl-diphosphooligosaccharide--protein glycosyltransferase subunit 2 OS=Homo sapiens GN=RPN2 PE=1	sp|P04844|RPN2_HUMAN	17.8	10	1.0965	0.8385
404	DNA-directed RNA polymerase I subunit RPA2 OS=Homo sapiens GN=POLR1B PE=1 SV=2	sp|Q9H9Y6|RPA2_HUMAN	13	5	0.5297	0.1435
405	Nucleoporin NUP188 homolog OS=Homo sapiens GN=NUP188 PE=1 SV=1	sp|Q5SRE5|NU188_HUMAN	8	8	0.6486	0.2987
406	Thyroid hormone receptor-associated protein 3 OS=Homo sapiens GN=THRAP3 PE=1 SV=2	sp|Q9Y2W1|TR150_HUMAN	10.3	9	0.8241	0.5557
407	Cytoplasmic aconitate hydratase OS=Homo sapiens GN=ACO1 PE=1 SV=3	sp|P21399|ACOC_HUMAN	13.7	6	1.3804	0.3565
408	Probable ATP-dependent RNA helicase DDX27 OS=Homo sapiens GN=DDX27 PE=1 SV=2	sp|Q96GQ7|DDX27_HUMAN	11.1	6	0.8954	0.5096
409	Interleukin enhancer-binding factor 3 OS=Homo sapiens GN=ILF3 PE=1 SV=3	sp|Q12906|ILF3_HUMAN	12.6	33	1.0186	0.7356
410	14-3-3 protein zeta/delta OS=Homo sapiens GN=YWHAZ PE=1 SV=1	sp|P63104|1433Z_HUMAN	55.1	18	1.0471	0.6972

411	DNA topoisomerase 2-beta OS=Homo sapiens	sp|Q02880|TOP2B_HUMAN	9	6	0.9817	0.8977
412	TATA-binding protein-associated factor 172 OS=Homo sapiens GN=BTAF1 PE=1 SV=2	sp|O14981|BTAF1_HUMAN	6.8	5	0.7586	0.5747
413	Rho guanine nucleotide exchange factor 6 OS=Homo sapiens GN=ARHGEF6 PE=1 SV=2	sp|Q15052|ARHG6_HUMAN	16.9	10	1.0093	0.8464
414	Dihydropyrimidinase-related protein 2 OS=Homo sapiens GN=DPYSL2 PE=1 SV=1	sp|Q16555|DPYL2_HUMAN	22	8	1.1912	0.5944
415	Glutamine--tRNA ligase OS=Homo sapiens GN=QARS PE=1 SV=1	sp|P47897|SYQ_HUMAN	19.5	9	0.9376	0.7828
416	Eukaryotic peptide chain release factor GTP-binding subunit ERF3A OS=Homo sapiens GN=GSPT1 PE=1	sp|P15170|ERF3A_HUMAN	20.8	7	0.9036	0.8579
417	Putative ATP-dependent RNA helicase DHX30 OS=Homo sapiens GN=DHX30 PE=1 SV=1	sp|Q7L2E3|DHX30_HUMAN	14.2	7	0.7447	0.5763
418	Receptor-type tyrosine-protein phosphatase C OS=Homo sapiens GN=PTPRC PE=1 SV=2	sp|P08575|PTPRC_HUMAN	10.1	13	1.3305	0.4672
419	ATP-binding cassette sub-family F member 2 OS=Homo sapiens GN=ABCF2 PE=1 SV=2	sp|Q9UG63|ABCF2_HUMAN	15.7	8	0.6982	0.3383
420	Eukaryotic translation initiation factor 5A-1 OS=Homo sapiens GN=EIF5A PE=1 SV=2	sp|P63241|IF5A1_HUMAN	66.9	29	1.0471	0.5946
421	Nuclear valosin-containing protein-like OS=Homo sapiens GN=NVL PE=1 SV=1	sp|O15381|NVL_HUMAN	18.9	14	0.6081	0.2306
422	Stomatin-like protein 2, mitochondrial OS=Homo sapiens GN=STOML2	sp|Q9UJZ1|STML2_HUMAN	30.9	6	1.0965	0.3912
423	Cullin-3 OS=Homo sapiens GN=CUL3 PE=1 SV=2	sp|Q13618|CUL3_HUMAN	11.7	7	0.9036	0.4993
424	Eukaryotic initiation factor 4A-III OS=Homo sapiens GN=EIF4A3 PE=1	sp|P38919|IF4A3_HUMAN	30.7	8	0.7244	0.581
425	Unconventional myosin-Ie OS=Homo sapiens GN=MYO1E PE=1 SV=2	sp|Q12965|MYO1E_HUMAN	12.6	11	1.0568	0.807
426	5'-3' exoribonuclease 2 OS=Homo sapiens GN=XRN2 PE=1 SV=1	sp|Q9H0D6|XRN2_HUMAN	9.2	7	0.9204	0.5206

427	Tropomyosin alpha-3 chain OS=Homo sapiens GN=TPM3 PE=1	sp|P06753|TPM3_HUMAN	35.8	10	0.929	0.9002
428	Nucleolar GTP-binding protein 1 OS=Homo sapiens GN=GTPBP4 PE=1 SV=3	sp|Q9BZE4|NOG1_HUMAN	15.1	6	0.9376	0.5802
429	Afadin OS=Homo sapiens GN=MLLT4 PE=1	sp|P55196|AFAD_HUMAN	9	5	1.1482	0.3319
430	AMP deaminase 2 OS=Homo sapiens GN=AMPD2 PE=1 SV=2	sp|Q01433|AMPD2_HUMAN	14.6	7	1.4191	0.8079
431	Proteasome subunit beta type-4 OS=Homo sapiens GN=PSMB4 PE=1 SV=4	sp|P28070|PSB4_HUMAN	42.8	15	1.0093	0.6766
432	Proteasome subunit alpha type-1 OS=Homo sapiens GN=PSMA1 PE=1 SV=1	sp|P25786|PSA1_HUMAN	46.4	7	0.9204	0.9035
433	Eukaryotic peptide chain release factor subunit 1 OS=Homo sapiens GN=ETF1 PE=1	sp|P62495|ERF1_HUMAN	23.3	8	1.0186	0.7466
434	Cell cycle and apoptosis regulator protein 2 OS=Homo sapiens GN=CCAR2 PE=1	sp|Q8N163|CCAR2_HUMAN	17.7	10	0.9462	0.7138
435	Ubiquitin carboxyl-terminal hydrolase 24 OS=Homo sapiens GN=USP24 PE=1	sp|Q9UPU5|UBP24_HUMAN	6.8	8	1.1272	0.8891
436	Peroxiredoxin-6 OS=Homo sapiens GN=PRDX6 PE=1 SV=3	sp|P30041|PRDX6_HUMAN	49.6	17	1.2942	0.5523
437	Multidrug resistance-associated protein 1 OS=Homo sapiens GN=ABCC1 PE=1 SV=3	sp|P33527|MRP1_HUMAN	7.4	5	2.0324	0.1801
438	Protein-tyrosine kinase 2-beta OS=Homo sapiens GN=PTK2B PE=1 SV=2	sp|Q14289|FAK2_HUMAN	12.5	9	0.9908	0.9847
439	Eukaryotic translation initiation factor 5 OS=Homo sapiens GN=EIF5 PE=1	sp|P55010|IF5_HUMAN	23.7	6	1.3305	0.4113
440	Sister chromatid cohesion protein PDS5 homolog A OS=Homo sapiens GN=PDS5A PE=1 SV=1	sp|Q29RF7|PDS5A_HUMAN	9.6	5	0.879	0.5043
441	Protein deglycase DJ-1 OS=Homo sapiens GN=PARK7 PE=1	sp|Q99497|PARK7_HUMAN	46	7	1.0666	0.812
442	Signal recognition particle subunit SRP68 OS=Homo sapiens GN=SRP68 PE=1 SV=2	sp|Q9UHB9|SRP68_HUMAN	17.2	5	1	0.9174
443	Ras GTPase-activating protein 1 OS=Homo sapiens GN=RASA1 PE=1 SV=1	sp|P20936|RASA1_HUMAN	8.8	5	1.0666	0.5336

444	Mitogen-activated protein kinase 1 OS=Homo sapiens GN=MAPK1 PE=1 SV=3	sp|P28482|MK01_HUMAN	20	5	0.912	0.8351
445	Glycerol-3-phosphate dehydrogenase, mitochondrial OS=Homo sapiens GN=GPD2 PE=1 SV=3	sp|P43304|GPDM_HUMAN	15.4	5	1.0965	0.5612
446	60S ribosomal protein L9 OS=Homo sapiens GN=RPL9 PE=1 SV=1	sp|P32969|RL9_HUMAN	54.7	8	0.9727	0.98
447	Phosphoinositide 3-kinase adapter protein 1 OS=Homo sapiens GN=PIK3AP1 PE=1 SV=2	sp|Q6ZUJ8|BCAP_HUMAN	12.2	6	0.9817	0.9919
448	Beta-catenin-like protein 1 OS=Homo sapiens GN=CTNNBL1 PE=1 SV=1	sp|Q8WYA6|CTBL1_HUMAN	12.1	8	0.6252	0.2052
449	60S ribosomal protein L10a OS=Homo sapiens GN=RPL10A PE=1 SV=2	sp|P62906|RL10A_HUMAN	30.4	5	0.955	0.8058
450	Protein transport protein Sec31A OS=Homo sapiens GN=SEC31A PE=1 SV=3	sp|O94979|SC31A_HUMAN	12.2	9	1.0375	0.8673
451	Gelsolin OS=Homo sapiens GN=GSN PE=1 SV=1	sp|P06396|GELS_HUMAN	18.2	7	1.977	0.2355
452	Sialic acid synthase OS=Homo sapiens GN=NANS PE=1 SV=2	sp|Q9NR45|SIAS_HUMAN	32	7	1.1066	0.6084
453	Eukaryotic translation initiation factor 3 subunit D OS=Homo sapiens GN=EIF3D PE=1	sp|O15371|EIF3D_HUMAN	22.3	11	0.7112	0.4688
454	Brefeldin A-inhibited guanine nucleotide-exchange protein 1 OS=Homo sapiens GN=ARFGEF1 PE=1 SV=2	sp|Q9Y6D6|BIG1_HUMAN	8.7	7	1.0375	0.8187
455	Isocitrate dehydrogenase [NADP], mitochondrial OS=Homo sapiens GN=IDH2 PE=1 SV=2	sp|P48735|IDHP_HUMAN	15.3	8	0.8954	0.9007
456	Cytosolic non-specific dipeptidase OS=Homo sapiens GN=CNDP2 PE=1 SV=2	sp|Q96KP4|CNDP2_HUMAN	16	7	2.0701	0.4222
457	Myosin regulatory light chain 12A OS=Homo sapiens GN=MYL12A PE=1 SV=2	sp|P19105|ML12A_HUMAN	45.6	10	1.028	0.5873
458	Fumarate hydratase, mitochondrial OS=Homo sapiens	sp|P07954|FUMH_HUMAN	28.8	9	1.2823	0.6433

459	Translocon-associated protein subunit alpha OS=Homo sapiens GN=SSR1 PE=1 SV=3	sp|P43307|SSRA_HUMAN	26.9	8	0.9817	0.9287
460	Luc7-like protein 3 OS=Homo sapiens GN=LUC7L3 PE=1 SV=2	sp|O95232|LC7L3_HUMAN	20.1	6	0.9462	0.6239
461	DNA-directed RNA polymerase I subunit RPA1 OS=Homo sapiens GN=POLR1A PE=1 SV=2	sp|O95602|RPA1_HUMAN	9.5	5	0.9817	0.6486
462	Condensin complex subunit 3 OS=Homo sapiens GN=NCAPG PE=1 SV=1	sp|Q9BPX3|CND3_HUMAN	11.3	6	0.6138	0.3701
463	AT-rich interactive domain-containing protein 1A OS=Homo sapiens GN=ARID1A PE=1 SV=3	sp|O14497|ARI1A_HUMAN	4.7	5	0.863	0.9402
464	Elongation factor Tu, mitochondrial OS=Homo sapiens GN=TUFM PE=1 SV=2	sp|P49411|EFTU_HUMAN	26.6	5	1.0186	0.8896
465	Ubiquilin-1 OS=Homo sapiens GN=UBQLN1 PE=1 SV=2	sp|Q9UMX0|UBQL1_HUMAN	21.1	8	1.0093	0.8872
466	Translation initiation factor eIF-2B subunit delta OS=Homo sapiens GN=EIF2B4 PE=1	sp|Q9UI10|EI2BD_HUMAN	22	5	0.955	0.9628
467	Ubiquitin-associated protein 2-like OS=Homo sapiens GN=UBAP2L	sp|Q14157|UBP2L_HUMAN	11.3	6	0.4207	0.4454
468	Dynactin subunit 2 OS=Homo sapiens GN=DCTN2 PE=1 SV=4	sp|Q13561|DCTN2_HUMAN	26.9	5	1.1169	0.5151
469	Histone deacetylase 1 OS=Homo sapiens GN=HDAC1 PE=1	sp|Q13547|HDAC1_HUMAN	16.8	8	0.787	0.7965
470	Myosin light polypeptide 6 OS=Homo sapiens GN=MYL6 PE=1 SV=2	sp|P60660|MYL6_HUMAN	60.9	6	0.8872	0.6336
471	Chromobox protein homolog 3 OS=Homo sapiens GN=CBX3 PE=1 SV=4	sp|Q13185|CBX3_HUMAN	49.2	7	0.4831	0.2771
472	60S ribosomal protein L24 OS=Homo sapiens GN=RPL24 PE=1 SV=1	sp|P83731|RL24_HUMAN	23.6	7	0.9638	0.9149
473	Ribose-phosphate pyrophosphokinase 1 OS=Homo sapiens GN=PRPS1 PE=1	sp|P60891|PRPS1_HUMAN	25.5	7	0.8954	0.7625
474	Eukaryotic translation initiation factor 3 subunit H OS=Homo sapiens GN=EIF3H PE=1	sp|O15372|EIF3H_HUMAN	23.9	8	0.8954	0.7664

475	Vacuolar protein sorting-associated protein 29 OS=Homo sapiens GN=VPS29 PE=1 SV=1	sp|Q9UBQ0|VPS29_HUMAN	34.6	5	1	0.846
476	Cell division control protein 42 homolog OS=Homo sapiens GN=CDC42 PE=1	sp|P60953|CDC42_HUMAN	35.1	7	1	0.896
477	Prefoldin subunit 3 OS=Homo sapiens GN=VBP1 PE=1 SV=3	sp|P61758|PFD3_HUMAN	28.4	5	1	0.9841
478	1-phosphatidylinositol 4,5-bisphosphate phosphodiesterase gamma-2 OS=Homo sapiens GN=PLCG2 PE=1 SV=4	sp|P16885|PLCG2_HUMAN	12.9	8	0.9908	0.5996
479	Ras-related C3 botulinum toxin substrate 1 OS=Homo sapiens GN=RAC1 PE=1 SV=1	sp|P63000|RAC1_HUMAN	46.4	7	0.955	0.9314
480	Gephyrin OS=Homo sapiens GN=GPHN PE=1 SV=1	sp|Q9NQX3|GEPH_HUMAN	17.7	5	1.1272	0.3398
481	Oxysterol-binding protein-related protein 8 OS=Homo sapiens GN=OSBPL8 PE=1 SV=3	sp|Q9BZF1|OSBL8_HUMAN	12.6	5	1.1803	0.4442
482	Eukaryotic translation initiation factor 3 subunit L OS=Homo sapiens GN=EIF3L PE=1	sp|Q9Y262|EIF3L_HUMAN	22	8	0.7311	0.4771
483	General transcription factor IIE subunit 1 OS=Homo sapiens GN=GTF2E1 PE=1 SV=2	sp|P29083|T2EA_HUMAN	21.4	5	0.5495	0.2108
484	Engulfment and cell motility protein 1 OS=Homo sapiens GN=ELMO1 PE=1	sp|Q92556|ELMO1_HUMAN	12.5	6	0.9727	0.9324
485	40S ribosomal protein S3 OS=Homo sapiens GN=RPS3 PE=1 SV=2	sp|P23396|RS3_HUMAN	35	7	0.9204	0.5436
486	Cullin-5 OS=Homo sapiens GN=CUL5 PE=1 SV=4	sp|Q93034|CUL5_HUMAN	13.2	7	1.0186	0.2881
487	40S ribosomal protein S8 OS=Homo sapiens GN=RPS8 PE=1 SV=2	sp|P62241|RS8_HUMAN	44.2	16	0.9908	0.9294
488	Aldose reductase OS=Homo sapiens GN=AKR1B1 PE=1 SV=3	sp|P15121|ALDR_HUMAN	31	12	1.4997	0.4643
489	DNA topoisomerase 1 OS=Homo sapiens GN=TOP1 PE=1	sp|P11387|TOP1_HUMAN	13.1	6	0.9036	0.6911
490	Nucleoside diphosphate kinase B OS=Homo sapiens GN=NME2 PE=1 SV=1	sp|P22392|NDKB_HUMAN	47.4	10	0.9727	0.9686

491	Heterogeneous nuclear ribonucleoprotein L-like OS=Homo sapiens GN=HNRNPLL PE=1 SV=1	sp|Q8WVV9|HNRLL_HUMAN	16.2	6	0.6855	0.7668
492	Heterogeneous nuclear ribonucleoprotein H OS=Homo sapiens GN=HNRNPH1	sp|P31943|HNRH1_HUMAN	28.5	18	0.879	0.8096
493	Polyadenylate-binding protein 4 OS=Homo sapiens GN=PABPC4 PE=1 SV=1	sp|Q13310|PABP4_HUMAN	28.9	11	0.1629	0.0437
494	60S ribosomal protein L13a OS=Homo sapiens GN=RPL13A PE=1 SV=2	sp|P40429|RL13A_HUMAN	24.6	6	0.3837	0.2178
495	GTP-binding nuclear protein Ran OS=Homo sapiens GN=RAN PE=1 SV=3	sp|P62826|RAN_HUMAN	44.4	14	0.6026	0.3071
496	Probable ATP-dependent RNA helicase DDX17 OS=Homo sapiens GN=DDX17 PE=1 SV=2	sp|Q92841|DDX17_HUMAN	22.2	6	0.9817	0.8735
497	RalBP1-associated Eps domain-containing protein 1 OS=Homo sapiens GN=REPS1 PE=1	sp|Q96D71|REPS1_HUMAN	13.2	6	1.0471	0.769
498	Endoplasmic reticulum resident protein 29 OS=Homo sapiens GN=ERP29 PE=1 SV=4	sp|P30040|ERP29_HUMAN	34.5	5	0.7516	0.6357
499	Acetyl-CoA carboxylase 1 OS=Homo sapiens GN=ACACA PE=1 SV=2	sp|Q13085|ACACA_HUMAN	8.4	5	1.0375	0.7397
500	GTP-binding protein SAR1a OS=Homo sapiens GN=SAR1A PE=1	sp|Q9NR31|SAR1A_HUMAN	59.1	6	1	0.8494
501	Proteasome subunit alpha type-2 OS=Homo sapiens GN=PSMA2 PE=1 SV=2	sp|P25787|PSA2_HUMAN	42.7	6	1.0093	0.99
502	Bifunctional purine biosynthesis protein PURH OS=Homo sapiens GN=ATIC PE=1	sp|P31939|PUR9_HUMAN	34.8	27	1.2023	0.9189
503	DnaJ homolog subfamily B member 11 OS=Homo sapiens GN=DNAJB11 PE=1 SV=1	sp|Q9UBS4|DJB11_HUMAN	32.4	6	0.929	0.7389
504	Mitochondrial carrier homolog 2 OS=Homo sapiens GN=MTCH2 PE=1 SV=1	sp|Q9Y6C9|MTCH2_HUMAN	32.3	9	0.9727	0.9991
505	DNA ligase 1 OS=Homo sapiens GN=LIG1 PE=1 SV=1	sp|P18858|DNLI1_HUMAN	12.2	7	0.3837	0.1494

506	Tricarboxylate transport protein, mitochondrial OS=Homo sapiens GN=SLC25A1 PE=1 SV=2	sp|P53007|TXTP_HUMAN	25.4	5	2.1878	0.0739
507	Ras GTPase-activating protein-binding protein 1 OS=Homo sapiens GN=G3BP1 PE=1 SV=1	sp|Q13283|G3BP1_HUMAN	19.7	6	0.4487	0.6031
508	60S acidic ribosomal protein P0 OS=Homo sapiens GN=RPLP0 PE=1	sp|P05388|RLA0_HUMAN	24	9	0.9638	0.972
509	YLP motif-containing protein 1 OS=Homo sapiens GN=YLPM1 PE=1	sp|P49750|YLPM1_HUMAN	6.5	6	0.8166	0.7759
510	Protein kinase C delta type OS=Homo sapiens GN=PRKCD PE=1 SV=2	sp|Q05655|KPCD_HUMAN	18.5	8	1.2823	0.5757
511	Exportin-4 OS=Homo sapiens GN=XPO4 PE=1 SV=2	sp|Q9C0E2|XPO4_HUMAN	12.6	11	0.6486	0.4593
512	Prolow-density lipoprotein receptor-related protein 1 OS=Homo sapiens GN=LRP1 PE=1	sp|Q07954|LRP1_HUMAN	3.6	7	1.0568	0.7781
513	DNA polymerase delta catalytic subunit OS=Homo sapiens GN=POLD1 PE=1	sp|P28340|DPOD1_HUMAN	8.7	5	0.8472	0.6339
514	Septin-7 OS=Homo sapiens GN=SEPT7 PE=1	sp|Q16181|SEPT7_HUMAN	23.8	8	1.1169	0.7732
515	Polycomb protein SUZ12 OS=Homo sapiens GN=SUZ12 PE=1	sp|Q15022|SUZ12_HUMAN	10.8	6	0.5445	0.4166
516	Procollagen-lysine,2-oxoglutarate 5-dioxygenase 3 OS=Homo sapiens GN=PLOD3 PE=1 SV=1	sp|O60568|PLOD3_HUMAN	9.6	6	1.1272	0.8061
517	Voltage-dependent anion-selective channel protein 1 OS=Homo sapiens GN=VDAC1 PE=1 SV=2	sp|P21796|VDAC1_HUMAN	48.1	9	1.1169	0.7225
518	V-type proton ATPase subunit E 1 OS=Homo sapiens GN=ATP6V1E1 PE=1 SV=1	sp|P36543|VATE1_HUMAN	26.6	6	1.4859	0.1534
519	Bleomycin hydrolase OS=Homo sapiens GN=BLMH PE=1 SV=1	sp|Q13867|BLMH_HUMAN	24.4	7	1.0965	0.8771
520	Leucyl-cystinyl aminopeptidase OS=Homo sapiens GN=LNPEP PE=1 SV=3	sp|Q9UIQ6|LCAP_HUMAN	11.9	8	1.9588	0.1512
521	Annexin A2 OS=Homo sapiens GN=ANXA2 PE=1 SV=2	sp|P07355|ANXA2_HUMAN	20.4	10	1.4859	0.5038

522	26S proteasome non-ATPase regulatory subunit 3 OS=Homo sapiens GN=PSMD3 PE=1	sp|O43242|PSMD3_HUMAN	30.7	7	1.1376	0.7178
523	Fermitin family homolog 3 OS=Homo sapiens GN=FERMT3 PE=1 SV=1	sp|Q86UX7|URP2_HUMAN	27.3	11	1.8535	0.925
524	MICOS complex subunit MIC60 OS=Homo sapiens GN=IMMT PE=1 SV=1	sp|Q16891|MIC60_HUMAN	13.2	7	1.5849	0.68
525	Heterogeneous nuclear ribonucleoprotein A0 OS=Homo sapiens GN=HNRNPA0 PE=1 SV=1	sp|Q13151|ROA0_HUMAN	36.4	7	0.8954	0.5407
526	Calreticulin OS=Homo sapiens GN=CALR PE=1 SV=1	sp|P27797|CALR_HUMAN	35.5	8	0.9908	0.6102
527	COP9 signalosome complex subunit 4 OS=Homo sapiens GN=COPS4 PE=1 SV=1	sp|Q9BT78|CSN4_HUMAN	23.7	7	0.7727	0.8896
528	MMS19 nucleotide excision repair protein homolog OS=Homo sapiens GN=MMS19 PE=1 SV=2	sp|Q96T76|MMS19_HUMAN	9.7	5	0.9727	0.9067
529	Dedicator of cytokinesis protein 11 OS=Homo sapiens GN=DOCK11 PE=1 SV=2	sp|Q5JSL3|DOC11_HUMAN	5.6	5	1.1588	0.3049
530	F-actin-capping protein subunit beta OS=Homo sapiens GN=CAPZB PE=1 SV=4	sp|P47756|CAPZB_HUMAN	26.7	8	1.028	0.6094
531	Rho GDP-dissociation inhibitor 2 OS=Homo sapiens GN=ARHGDIB	sp|P52566|GDIR2_HUMAN	35.3	6	1.5417	0.5447
532	AP-1 complex subunit gamma-1 OS=Homo sapiens GN=AP1G1 PE=1 SV=5	sp|O43747|AP1G1_HUMAN	15.3	6	1.0186	0.6583
533	Probable ubiquitin carboxyl-terminal hydrolase FAF-X OS=Homo sapiens GN=USP9X PE=1 SV=3	sp|Q93008|USP9X_HUMAN	6.3	7	1.2823	0.2664
534	Serine/threonine-protein phosphatase 2B catalytic subunit beta isoform OS=Homo sapiens GN=PPP3CB PE=1 SV=2	sp|P16298|PP2BB_HUMAN	18.3	4	0.9908	0.9487
535	Serine/threonine-protein kinase N1 OS=Homo sapiens GN=PKN1 PE=1 SV=2	sp|Q16512|PKN1_HUMAN	11.2	5	1.1482	0.4974
536	Protein kinase C alpha type OS=Homo sapiens GN=PRKCA PE=1 SV=4	sp|P17252|KPCA_HUMAN	22.2	5	1.4322	0.3864

537	Ubiquitin-40S ribosomal protein S27a OS=Homo sapiens GN=RPS27A PE=1 SV=2	sp|P62979|RS27A_HUMAN	54.5	9	0.9817	0.9395
538	Elongation factor 1-delta OS=Homo sapiens GN=EEF1D PE=1	sp|P29692|EF1D_HUMAN	42	5	0.9727	0.9531
539	Calcium/calmodulin-dependent protein kinase type II subunit gamma OS=Homo sapiens GN=CAMK2G PE=1 SV=3	sp|Q13555|KCC2G_HUMAN	16.1	5	1.0186	0.6829
540	Adenosylhomocysteinase 2 OS=Homo sapiens GN=AHCYL1 PE=1 SV=2	sp|O43865|SAHH2_HUMAN	14.2	5	1.0965	0.7908
541	Protein transport protein Sec24C OS=Homo sapiens GN=SEC24C PE=1 SV=3	sp|P53992|SC24C_HUMAN	8.2	7	0.9638	0.8041
542	Cytosol aminopeptidase OS=Homo sapiens GN=LAP3 PE=1 SV=3	sp|P28838|AMPL_HUMAN	15.6	7	1.2359	0.4679
543	ELAV-like protein 1 OS=Homo sapiens GN=ELAVL1	sp|Q15717|ELAV1_HUMAN	29.5	8	0.9036	0.5322
544	60S acidic ribosomal protein P2 OS=Homo sapiens GN=RPLP2 PE=1	sp|P05387|RLA2_HUMAN	58.3	9	0.8472	0.9418
545	Serine/threonine-protein phosphatase 2A activator OS=Homo sapiens GN=PPP2R4 PE=1 SV=3	sp|Q15257|PTPA_HUMAN	20.4	6	0.9817	0.9896
546	Voltage-gated potassium channel subunit beta-2 OS=Homo sapiens GN=KCNAB2 PE=1 SV=2	sp|Q13303|KCAB2_HUMAN	39.2	24	1.5136	0.3611
547	ERO1-like protein alpha OS=Homo sapiens GN=ERO1L PE=1	sp|Q96HE7|ERO1A_HUMAN	21.4	6	2.5823	0.3565
548	Ras suppressor protein 1 OS=Homo sapiens GN=RSU1 PE=1	sp|Q15404|RSU1_HUMAN	26.7	6	1.3183	0.4542
549	C-1-tetrahydrofolate synthase, cytoplasmic OS=Homo sapiens GN=MTHFD1	sp|P11586|C1TC_HUMAN	21.2	17	0.8954	0.7497
550	Activating signal cointegrator 1 complex subunit 3 OS=Homo sapiens GN=ASCC3 PE=1 SV=3	sp|Q8N3C0|ASCC3_HUMAN	7.2	7	1.1169	0.6695
551	Nucleolar protein 10 OS=Homo sapiens GN=NOL10 PE=1	sp|Q9BSC4|NOL10_HUMAN	13.1	6	1.0471	0.7998
552	Dipeptidyl peptidase 3 OS=Homo sapiens GN=DPP3 PE=1	sp|Q9NY33|DPP3_HUMAN	21.7	13	1.1169	0.6932

553	Rho-associated protein kinase 2 OS=Homo sapiens GN=ROCK2 PE=1 SV=4	sp|O75116|ROCK2_HUMAN	10.3	5	1.0186	0.7402
554	WD repeat-containing protein 36 OS=Homo sapiens GN=WDR36 PE=1	sp|Q8NI36|WDR36_HUMAN	11.8	6	0.8872	0.7203
555	Integrin-linked protein kinase OS=Homo sapiens GN=ILK PE=1 SV=2	sp|Q13418|ILK_HUMAN	15	5	1.7539	0.6029
556	Hsp70-binding protein 1 OS=Homo sapiens GN=HSPBP1 PE=1 SV=1	sp|Q9NZL4|HPBP1_HUMAN	34.5	5	0.7516	0.3602
557	Golgi-specific brefeldin A-resistance guanine nucleotide exchange factor 1 OS=Homo sapiens GN=GBF1 PE=1	sp|Q92538|GBF1_HUMAN	6.5	7	0.9727	0.8538
558	Integrator complex subunit 3 OS=Homo sapiens GN=INTS3 PE=1	sp|Q68E01|INT3_HUMAN	8.5	5	0.6855	0.4967
559	Guanine nucleotide-binding protein-like 1 OS=Homo sapiens GN=GNL1 PE=1	sp|P36915|GNL1_HUMAN	12.4	4	1.028	0.6811
560	Protein transport protein Sec23A OS=Homo sapiens GN=SEC23A PE=1 SV=2	sp|Q15436|SC23A_HUMAN	15.3	8	1.6596	0.4184
561	Heterogeneous nuclear ribonucleoprotein R OS=Homo sapiens GN=HNRNPR	sp|O43390|HNRPR_HUMAN	19.3	11	0.879	0.555
562	Septin-9 OS=Homo sapiens GN=SEPT9 PE=1	sp|Q9UHD8|SEPT9_HUMAN	20.5	5	1.3677	0.3752
563	Beta-enolase OS=Homo sapiens GN=ENO3 PE=1 SV=5	sp|P13929|ENOB_HUMAN	49.3	75	0.9638	0.9611
564	14-3-3 protein eta OS=Homo sapiens GN=YWHAH PE=1 SV=4	sp|Q04917|1433F_HUMAN	50.8	30	1	0.77
565	Phosphoinositide 3-kinase regulatory subunit 4 OS=Homo sapiens GN=PIK3R4 PE=1 SV=3	sp|Q99570|PI3R4_HUMAN	8	4	1.0375	0.4904
566	Cleavage stimulation factor subunit 3 OS=Homo sapiens GN=CSTF3 PE=1	sp|Q12996|CSTF3_HUMAN	15.5	6	0.9036	0.7493
567	Serine/threonine-protein phosphatase 6 regulatory ankyrin repeat subunit A OS=Homo sapiens GN=ANKRD28	sp|O15084|ANR28_HUMAN	9.1	4	4.4875	0.5221
568	60S ribosomal protein L32 OS=Homo sapiens GN=RPL32 PE=1 SV=2	sp|P62910|RL32_HUMAN	43.7	6	1.0965	0.836

569	Phosphatidylinositol transfer protein beta isoform OS=Homo sapiens GN=PITPNB PE=1 SV=2	sp|P48739|PIPNB_HUMAN	28.8	5	0.8166	0.691
570	Lysine--tRNA ligase OS=Homo sapiens GN=KARS PE=1 SV=3	sp|Q15046|SYK_HUMAN	17.1	15	1.0093	0.9148
571	60S ribosomal protein L18 OS=Homo sapiens GN=RPL18 PE=1 SV=2	sp|Q07020|RL18_HUMAN	40.4	10	1.1169	0.7346
572	60S ribosomal protein L15 OS=Homo sapiens GN=RPL15 PE=1 SV=2	sp|P61313|RL15_HUMAN	40.2	6	0.9727	0.9083
573	ATP-dependent RNA helicase DDX42 OS=Homo sapiens GN=DDX42 PE=1	sp|Q86XP3|DDX42_HUMAN	11.1	4	1	0.9019
574	Calcium-binding mitochondrial carrier protein Aralar1 OS=Homo sapiens GN=SLC25A12 PE=1 SV=2	sp|O75746|CMC1_HUMAN	14.5	9	0.9817	0.9957
575	Serine/threonine-protein phosphatase 4 regulatory subunit 1 OS=Homo sapiens GN=PPP4R1 PE=1	sp|Q8TF05|PP4R1_HUMAN	10.8	4	1.3183	0.4695
576	Serine/threonine-protein kinase 10 OS=Homo sapiens GN=STK10 PE=1 SV=1	sp|O94804|STK10_HUMAN	8	5	1.0375	0.7692
577	Peroxiredoxin-5, mitochondrial OS=Homo sapiens GN=PRDX5 PE=1 SV=4	sp|P30044|PRDX5_HUMAN	40.2	14	2.1478	0.1542
578	Cullin-4A OS=Homo sapiens GN=CUL4A PE=1 SV=3	sp|Q13619|CUL4A_HUMAN	10.5	5	0.9908	0.3305
579	40S ribosomal protein S23 OS=Homo sapiens GN=RPS23 PE=1 SV=3	sp|P62266|RS23_HUMAN	32.9	28	0.673	0.6586
580	THO complex subunit 2 OS=Homo sapiens GN=THOC2 PE=1	sp|Q8NI27|THOC2_HUMAN	6.2	4	1.0093	0.9704
581	60S ribosomal protein L13 OS=Homo sapiens GN=RPL13 PE=1 SV=4	sp|P26373|RL13_HUMAN	26.1	6	0.871	0.8695
582	Thioredoxin reductase 1, cytoplasmic OS=Homo sapiens GN=TXNRD1 PE=1 SV=3	sp|Q16881|TRXR1_HUMAN	14.2	5	1.2823	0.6425
583	Structural maintenance of chromosomes protein 4 OS=Homo sapiens GN=SMC4 PE=1	sp|Q9NTJ3|SMC4_HUMAN	10.6	5	0.5012	0.2254
584	Proteasome subunit alpha type-5 OS=Homo sapiens GN=PSMA5 PE=1 SV=3	sp|P28066|PSA5_HUMAN	48.1	6	0.9638	0.807

585	Phosphoserine aminotransferase OS=Homo sapiens GN=PSAT1 PE=1 SV=2	sp|Q9Y617|SERC_HUMAN	20	6	1.0568	0.9705
586	cAMP-dependent protein kinase type II-beta regulatory subunit OS=Homo sapiens GN=PRKAR2B PE=1 SV=3	sp|P31323|KAP3_HUMAN	31.3	7	0.9908	0.9994
587	RuvB-like 2 OS=Homo sapiens GN=RUVBL2 PE=1 SV=3	sp|Q9Y230|RUVB2_HUMAN	27.4	12	0.9817	0.8797
588	60S ribosomal protein L8 OS=Homo sapiens GN=RPL8 PE=1 SV=2	sp|P62917|RL8_HUMAN	26.1	5	0.9376	0.8898
589	Transmembrane protein 33 OS=Homo sapiens GN=TMEM33 PE=1 SV=2	sp|P57088|TMM33_HUMAN	36.4	10	1.0093	0.9733
590	UTP--glucose-1-phosphate uridylyltransferase OS=Homo sapiens GN=UGP2 PE=1 SV=5	sp|Q16851|UGPA_HUMAN	13.4	7	1.0471	0.5651
591	Tyrosine-protein kinase BAZ1B OS=Homo sapiens GN=BAZ1B PE=1 SV=2	sp|Q9UIG0|BAZ1B_HUMAN	9.7	13	0.5105	0.0836
592	Sorting nexin-5 OS=Homo sapiens GN=SNX5 PE=1 SV=1	sp|Q9Y5X3|SNX5_HUMAN	23.8	10	0.9204	0.8033
593	AP-2 complex subunit mu OS=Homo sapiens GN=AP2M1 PE=1 SV=2	sp|Q96CW1|AP2M1_HUMAN	27.6	6	1.0864	0.6192
594	S-formylglutathione hydrolase OS=Homo sapiens GN=ESD PE=1	sp|P10768|ESTD_HUMAN	40.8	5	2.355	0.1859
595	SUMO-activating enzyme subunit 2 OS=Homo sapiens GN=UBA2 PE=1 SV=2	sp|Q9UBT2|SAE2_HUMAN	14.7	5	0.929	0.5166
596	Alpha-centractin OS=Homo sapiens GN=ACTR1A PE=1 SV=1	sp|P61163|ACTZ_HUMAN	23.9	5	0.955	0.9335
597	CLIP-associating protein 1 OS=Homo sapiens GN=CLASP1 PE=1 SV=1	sp|Q7Z460|CLAP1_HUMAN	8.8	5	0.912	0.4037
598	Pre-mRNA-splicing factor SYF1 OS=Homo sapiens GN=XAB2 PE=1 SV=2	sp|Q9HCS7|SYF1_HUMAN	12.3	4	0.5861	0.4148
599	Guanine nucleotide-binding protein G(I)/G(S)/G(T) subunit beta-1 OS=Homo sapiens GN=GNB1 PE=1	sp|P62873|GBB1_HUMAN	22.4	8	1.0666	0.7005

600	Sorting and assembly machinery component 50 homolog OS=Homo sapiens GN=SAMM50 PE=1 SV=3	sp|Q9Y512|SAM50_HUMAN	22.6	8	1.1272	0.6389
601	TAR DNA-binding protein 43 OS=Homo sapiens GN=TARDBP PE=1 SV=1	sp|Q13148|TADBP_HUMAN	26.6	8	0.9204	0.6082
602	DnaJ homolog subfamily C member 13 OS=Homo sapiens GN=DNAJC13 PE=1 SV=5	sp|O75165|DJC13_HUMAN	7.2	8	1.3552	0.5256
603	Obg-like ATPase 1 OS=Homo sapiens GN=OLA1 PE=1 SV=2	sp|Q9NTK5|OLA1_HUMAN	27.8	8	0.912	0.9342
604	General vesicular transport factor p115 OS=Homo sapiens GN=USO1 PE=1 SV=2	sp|O60763|USO1_HUMAN	12.8	5	1	0.8687
605	Serine/threonine-protein phosphatase 2A catalytic subunit alpha isoform OS=Homo sapiens GN=PPP2CA	sp|P67775|PP2AA_HUMAN	24.3	9		
606	60S ribosomal protein L23 OS=Homo sapiens GN=RPL23 PE=1 SV=1	sp|P62829|RL23_HUMAN	46.4	6	0.9036	0.9617
607	Lysine-specific histone demethylase 1A OS=Homo sapiens GN=KDM1A PE=1 SV=2	sp|O60341|KDM1A_HUMAN	12.9	4	0.9204	0.4601
608	Protein argonaute-2 OS=Homo sapiens GN=AGO2 PE=1 SV=3	sp|Q9UKV8|AGO2_HUMAN	12.8	4	1.0965	0.6314
609	Inositol-3-phosphate synthase 1 OS=Homo sapiens GN=ISYNA1 PE=1	sp|Q9NPH2|INO1_HUMAN	20.3	8	0.5105	0.1785
610	Fructose-bisphosphate aldolase C OS=Homo sapiens GN=ALDOC PE=1 SV=2	sp|P09972|ALDOC_HUMAN	45.1	29	2.1677	0.3508
611	WASH complex subunit strumpellin OS=Homo sapiens GN=KIAA0196 PE=1 SV=1	sp|Q12768|STRUM_HUMAN	5.9	5	1.1066	0.3871
612	Proteasome activator complex subunit 3 OS=Homo sapiens GN=PSME3 PE=1	sp|P61289|PSME3_HUMAN	32.7	5	0.5598	0.153
613	Inositol 1,4,5-trisphosphate receptor type 3 OS=Homo sapiens GN=ITPR3 PE=1 SV=2	sp|Q14573|ITPR3_HUMAN	7.5	6	1.0375	0.8135
614	Signal transducer and activator of transcription 1-alpha/beta OS=Homo sapiens GN=STAT1 PE=1 SV=2	sp|P42224|STAT1_HUMAN	14.7	4	1.9588	0.3448

615	Platelet-activating factor acetylhydrolase IB subunit alpha OS=Homo sapiens GN=PAFAH1B1 PE=1 SV=2	sp|P43034|LIS1_HUMAN	16.3	4	1.3552	0.4018
616	Interleukin enhancer-binding factor 2 OS=Homo sapiens GN=ILF2 PE=1 SV=2	sp|Q12905|ILF2_HUMAN	19.2	5	0.9727	0.8641
617	Alkyldihydroxyacetonephosphate synthase, peroxisomal OS=Homo sapiens GN=AGPS PE=1 SV=1	sp|O00116|ADAS_HUMAN	17.9	6	0.955	0.7315
618	ATP-dependent RNA helicase DDX18 OS=Homo sapiens GN=DDX18 PE=1	sp|Q9NVP1|DDX18_HUMAN	13.9	5	0.955	0.8406
619	3-hydroxyacyl-CoA dehydrogenase type-2 OS=Homo sapiens GN=HSD17B10 PE=1 SV=3	sp|Q99714|HCD2_HUMAN	45.6	9	1.3305	0.4352
620	Eukaryotic translation initiation factor 3 subunit F OS=Homo sapiens GN=EIF3F PE=1	sp|O00303|EIF3F_HUMAN	18.2	6	0.9638	0.8549
621	Methionine--tRNA ligase, cytoplasmic OS=Homo sapiens GN=MARS PE=1 SV=2	sp|P56192|SYMC_HUMAN	17.4	7	1.0666	0.5857
622	U1 small nuclear ribonucleoprotein 70 kDa OS=Homo sapiens GN=SNRNP70 PE=1 SV=2	sp|P08621|RU17_HUMAN	13.7	6	0.9727	0.6421
623	NADH dehydrogenase [ubiquinone] iron-sulfur protein 2, mitochondrial OS=Homo sapiens GN=NDUFS2 PE=1 SV=2	sp|O75306|NDUS2_HUMAN	15.8	8	1.0666	0.8847
624	RNA-binding protein 39 OS=Homo sapiens GN=RBM39 PE=1	sp|Q14498|RBM39_HUMAN	16.8	4	0.9817	0.8901
625	Acidic leucine-rich nuclear phosphoprotein 32 family member E OS=Homo sapiens GN=ANP32E PE=1 SV=1	sp|Q9BTT0|AN32E_HUMAN	30.6	11	0.8954	0.8944
626	60S ribosomal protein L12 OS=Homo sapiens GN=RPL12 PE=1 SV=1	sp|P30050|RL12_HUMAN	52.1	6	1.0186	0.9265
627	Intron-binding protein aquarius OS=Homo sapiens GN=AQR PE=1 SV=4	sp|O60306|AQR_HUMAN	8.3	6	0.929	0.5579
628	Glycogen phosphorylase, brain form OS=Homo sapiens GN=PYGB PE=1	sp|P11216|PYGB_HUMAN	15	5	0.955	0.9655

629	CUGBP Elav-like family member 2 OS=Homo sapiens GN=CELF2 PE=1 SV=1	sp|O95319|CELF2_HUMAN	15.2	5	0.7656	0.4984
630	Putative RNA-binding protein Luc7-like 2 OS=Homo sapiens GN=LUC7L2 PE=1 SV=2	sp|Q9Y383|LC7L2_HUMAN	19.9	4	0.9376	0.5153
631	F-actin-capping protein subunit alpha-1 OS=Homo sapiens GN=CAPZA1 PE=1 SV=3	sp|P52907|CAZA1_HUMAN	47.6	18	1.0093	0.9844
632	Eukaryotic translation initiation factor 3 subunit J OS=Homo sapiens GN=EIF3J PE=1	sp|O75822|EIF3J_HUMAN	28.7	5	0.9817	0.9792
633	Acetoacetyl-CoA synthetase OS=Homo sapiens GN=AACS PE=1 SV=1	sp|Q86V21|AACS_HUMAN	12.2	4	1.2706	0.6429
634	FAS-associated factor 2 OS=Homo sapiens GN=FAF2 PE=1 SV=2	sp|Q96CS3|FAF2_HUMAN	15.7	6	1.1482	0.7184
635	Ribosome production factor 2 homolog OS=Homo sapiens GN=RPF2 PE=1	sp|Q9H7B2|RPF2_HUMAN	18.6	5	0.8472	0.7996
636	Ataxin-10 OS=Homo sapiens GN=ATXN10 PE=1 SV=1	sp|Q9UBB4|ATX10_HUMAN	13.9	4	0.673	0.5505
637	Ran-specific GTPase-activating protein OS=Homo sapiens GN=RANBP1 PE=1 SV=1	sp|P43487|RANG_HUMAN	36.3	4	0.8166	0.6549
638	Splicing factor U2AF 65 kDa subunit OS=Homo sapiens GN=U2AF2 PE=1	sp|P26368|U2AF2_HUMAN	20	11	0.879	0.7166
639	Condensin complex subunit 1 OS=Homo sapiens GN=NCAPD2 PE=1 SV=3	sp|Q15021|CND1_HUMAN	8.3	7	0.5754	0.4968
640	Syntaxin-binding protein 2 OS=Homo sapiens GN=STXBP2 PE=1 SV=2	sp|Q15833|STXB2_HUMAN	18.6	5	1.1803	0.4799
641	Protein phosphatase methylesterase 1 OS=Homo sapiens GN=PPME1 PE=1	sp|Q9Y570|PPME1_HUMAN	15.8	4	1.0186	0.3971
642	SWI/SNF-related matrix-associated actin-dependent regulator of chromatin subfamily B member 1 OS=Homo sapiens GN=SMARCB1 PE=1 SV=2	sp|Q12824|SNF5_HUMAN	23.4	6	0.673	0.6241
643	Phosphatidylinositol 4-kinase alpha OS=Homo sapiens GN=PI4KA PE=1 SV=3	sp|P42356|PI4KA_HUMAN	4.6	4	1	0.9496

644	Cleavage and polyadenylation specificity factor subunit 2 OS=Homo sapiens GN=CPSF2 PE=1	sp|Q9P2I0|CPSF2_HUMAN	12.8	5	0.9376	0.7412
645	Phosphate carrier protein, mitochondrial OS=Homo sapiens GN=SLC25A3 PE=1 SV=2	sp|Q00325|MPCP_HUMAN	27.1	43	1.0186	0.9164
646	Anaphase-promoting complex subunit 4 OS=Homo sapiens GN=ANAPC4 PE=1 SV=2	sp|Q9UJX5|APC4_HUMAN	10.3	4	0.6368	0.4428
647	Nucleoporin p54 OS=Homo sapiens GN=NUP54 PE=1 SV=2	sp|Q7Z3B4|NUP54_HUMAN	16.2	4	0.912	0.6873
648	Helicase SKI2W OS=Homo sapiens GN=SKIV2L PE=1 SV=3	sp|Q15477|SKIV2_HUMAN	6.8	4	1.0471	0.6763
649	Mitochondrial 2-oxoglutarate/malate carrier protein OS=Homo sapiens GN=SLC25A11 PE=1 SV=3	sp|Q02978|M2OM_HUMAN	27.4	4	1.4191	0.5664
650	Ras-related GTP-binding protein A OS=Homo sapiens GN=RRAGA PE=1 SV=1	sp|Q7L523|RRAGA_HUMAN	21.1	6	1.6904	0.2845
651	Cullin-1 OS=Homo sapiens GN=CUL1 PE=1 SV=2	sp|Q13616|CUL1_HUMAN	12.2	5	1.1482	0.5928
652	Phosphatidylinositol-binding clathrin assembly protein OS=Homo sapiens GN=PICALM PE=1 SV=2	sp|Q13492|PICAL_HUMAN	16.1	5	1	0.9934
653	Tyrosine-protein kinase CSK OS=Homo sapiens GN=CSK PE=1 SV=1	sp|P41240|CSK_HUMAN	17.8	5	0.955	0.7217
654	Actin-related protein 2/3 complex subunit 1B OS=Homo sapiens GN=ARPC1B	sp|O15143|ARC1B_HUMAN	30.7	6	1	0.9654
655	COP9 signalosome complex subunit 3 OS=Homo sapiens GN=COPS3 PE=1 SV=3	sp|Q9UNS2|CSN3_HUMAN	25.1	4	1.0186	0.8964
656	Importin-11 OS=Homo sapiens GN=IPO11 PE=1 SV=1	sp|Q9UI26|IPO11_HUMAN	9.1	4	0.673	0.3969
657	Ribosomal RNA small subunit methyltransferase NEP1 OS=Homo sapiens GN=EMG1 PE=1 SV=4	sp|Q92979|NEP1_HUMAN	27.5	4	0.7447	0.6792
658	RNA-binding protein FUS OS=Homo sapiens GN=FUS PE=1	sp|P35637|FUS_HUMAN	17.7	4	0.912	0.7357
659	Nuclear factor of activated T-cells 5 OS=Homo sapiens GN=NFAT5 PE=1 SV=1	sp|O94916|NFAT5_HUMAN	5.6	8	0.5248	0.3466

660	Catenin delta-1 OS=Homo sapiens GN=CTNND1 PE=1 SV=1	sp|O60716|CTND1_HUMAN	9.6	4	0.8318	0.8138
661	Heterogeneous nuclear ribonucleoprotein A/B OS=Homo sapiens GN=HNRNPAB PE=1 SV=2	sp|Q99729|ROAA_HUMAN	13.6	5	0.863	0.619
662	Ubiquitin carboxyl-terminal hydrolase 47 OS=Homo sapiens GN=USP47 PE=1	sp|Q96K76|UBP47_HUMAN	5.7	5	1.2474	0.6563
663	Endoplasmic reticulum lectin 1 OS=Homo sapiens GN=ERLEC1 PE=1 SV=1	sp|Q96DZ1|ERLEC_HUMAN	18	4	1.1376	0.778
664	Serine/threonine-protein phosphatase 5 OS=Homo sapiens GN=PPP5C PE=1	sp|P53041|PPP5_HUMAN	19.2	4	1.1169	0.6675
665	F-actin-capping protein subunit alpha-2 OS=Homo sapiens GN=CAPZA2 PE=1 SV=3	sp|P47755|CAZA2_HUMAN	37.1	14	1.1695	0.8046
666	Glutathione reductase, mitochondrial OS=Homo sapiens GN=GSR PE=1 SV=2	sp|P00390|GSHR_HUMAN	14.6	4	1.1169	0.7606
667	Sorting nexin-6 OS=Homo sapiens GN=SNX6 PE=1 SV=1	sp|Q9UNH7|SNX6_HUMAN	12.8	4	1.0093	0.924
668	Glycine amidinotransferase, mitochondrial OS=Homo sapiens GN=GATM PE=1 SV=1	sp|P50440|GATM_HUMAN	22	8	0.8395	0.6606
669	Dihydrolipoyllysine-residue succinyltransferase component of 2-oxoglutarate dehydrogenase complex, mitochondrial OS=Homo sapiens GN=DLST PE=1 SV=4	sp|P36957|ODO2_HUMAN	14.6	4	1.5849	0.5971
670	Ribonucleoside-diphosphate reductase subunit M2 OS=Homo sapiens GN=RRM2 PE=1 SV=1	sp|P31350|RIR2_HUMAN	16.2	4	0.2805	0.0389
671	V-type proton ATPase subunit C 1 OS=Homo sapiens GN=ATP6V1C1	sp|P21283|VATC1_HUMAN	14.9	4	1.0666	0.8804
672	Serine/threonine-protein kinase OSR1 OS=Homo sapiens GN=OXSR1 PE=1	sp|O95747|OXSR1_HUMAN	15.4	4	1.0186	0.9305
673	Golgi phosphoprotein 3 OS=Homo sapiens GN=GOLPH3 PE=1 SV=1	sp|Q9H4A6|GOLP3_HUMAN	24.2	4	0.8551	0.5517

674	Chitinase domain-containing protein 1 OS=Homo sapiens GN=CHID1 PE=1	sp|Q9BWS9|CHID1_HUMAN	15.3	4	1.4723	0.5643
675	Protein C10 OS=Homo sapiens GN=C12orf57 PE=1 SV=1	sp|Q99622|C10_HUMAN	43.7	6	0.7447	0.7305
676	Volume-regulated anion channel subunit LRRC8D OS=Homo sapiens GN=LRRC8D PE=1 SV=1	sp|Q7L1W4|LRC8D_HUMAN	8.6	4	1.6144	0.6876
677	S-phase kinase-associated protein 1 OS=Homo sapiens GN=SKP1 PE=1 SV=2	sp|P63208|SKP1_HUMAN	41.1	4	0.6792	0.4768
678	Succinyl-CoA ligase [ADP/GDP-forming] subunit alpha, mitochondrial OS=Homo sapiens GN=SUCLG1	sp|P53597|SUCA_HUMAN	18.5	4	1.028	0.6735
679	ADP-ribosylation factor-like protein 1 OS=Homo sapiens GN=ARL1 PE=1 SV=1	sp|P40616|ARL1_HUMAN	33.7	4	1.0093	0.8962
680	Beta-arrestin-2 OS=Homo sapiens GN=ARRB2 PE=1 SV=2	sp|P32121|ARRB2_HUMAN	17.6	4	0.9817	0.979
681	Charged multivesicular body protein 2a OS=Homo sapiens GN=CHMP2A PE=1 SV=1	sp|O43633|CHM2A_HUMAN	17.6	4	1.0093	0.8197
682	Protein MEMO1 OS=Homo sapiens GN=MEMO1 PE=1 SV=1	sp|Q9Y316|MEMO1_HUMAN	28	4	0.7379	0.4884
683	COP9 signalosome complex subunit 5 OS=Homo sapiens GN=COPS5 PE=1 SV=4	sp|Q92905|CSN5_HUMAN	17.7	4	1.0765	0.9778
684	5'-AMP-activated protein kinase catalytic subunit alpha-1 OS=Homo sapiens GN=PRKAA1 PE=1 SV=4	sp|Q13131|AAPK1_HUMAN	12	4	1.0765	0.6813
685	60S ribosomal protein L11 OS=Homo sapiens GN=RPL11 PE=1 SV=2	sp|P62913|RL11_HUMAN	30.3	4	0.9462	0.8535
686	Cytosolic phospholipase A2 OS=Homo sapiens GN=PLA2G4A PE=1 SV=2	sp|P47712|PA24A_HUMAN	8.3	4	0.912	0.9715
687	Serine/threonine-protein phosphatase 6 catalytic subunit OS=Homo sapiens GN=PPP6C PE=1 SV=1	sp|O00743|PPP6_HUMAN	22.6	4	1.028	0.9307
688	BAG family molecular chaperone regulator 5 OS=Homo sapiens GN=BAG5 PE=1 SV=1	sp|Q9UL15|BAG5_HUMAN	11.4	4	0.6486	0.241
689	Protein PRRC1 OS=Homo sapiens GN=PRRC1 PE=1 SV=1	sp|Q96M27|PRRC1_HUMAN	20.9	8	1.1066	0.7832

690	Flavin reductase (NADPH) OS=Homo sapiens GN=BLVRB PE=1 SV=3	sp|P30043|BLVRB_HUMAN	34	4	1.7539	0.3348
691	60S ribosomal protein L30 OS=Homo sapiens GN=RPL30 PE=1 SV=2	sp|P62888|RL30_HUMAN	41.7	11	1	0.8336
692	HEAT repeat-containing protein 5B OS=Homo sapiens GN=HEATR5B PE=1 SV=2	sp|Q9P2D3|HTR5B_HUMAN	6.3	4	0.9462	0.939
693	Macrophage migration inhibitory factor OS=Homo sapiens GN=MIF PE=1	sp|P14174|MIF_HUMAN	60.9	35	1.5276	0.6872
694	Nipped-B-like protein OS=Homo sapiens GN=NIPBL PE=1	sp|Q6KC79|NIPBL_HUMAN	5.4	5	0.9376	0.6692
695	UMP-CMP kinase OS=Homo sapiens GN=CMPK1 PE=1 SV=3	sp|P30085|KCY_HUMAN	33.7	5	1.1376	0.683
696	Small subunit processome component 20 homolog OS=Homo sapiens GN=UTP20 PE=1	sp|O75691|UTP20_HUMAN	7	4	0.8017	0.665
697	Mitochondrial import receptor subunit TOM70 OS=Homo sapiens GN=TOMM70A PE=1 SV=1	sp|O94826|TOM70_HUMAN	11	7	1	0.9518
698	Filamin-B OS=Homo sapiens GN=FLNB PE=1 SV=2	sp|O75369|FLNB_HUMAN	5.1	4	1.3677	0.3102
699	Serine/threonine-protein kinase WNK1 OS=Homo sapiens GN=WNK1 PE=1	sp|Q9H4A3|WNK1_HUMAN	5.1	4	1.0864	0.3941
700	EH domain-containing protein 2 OS=Homo sapiens GN=EHD2 PE=1 SV=2	sp|Q9NZN4|EHD2_HUMAN	16.2	4	0.6026	0.281
701	Pre-mRNA-splicing factor SPF27 OS=Homo sapiens GN=BCAS2 PE=1	sp|O75934|SPF27_HUMAN	39.6	4	1.028	0.7774
702	Paired amphipathic helix protein Sin3a OS=Homo sapiens GN=SIN3A PE=1 SV=2	sp|Q96ST3|SIN3A_HUMAN	6	4	0.8091	0.9931
703	Deubiquitinating protein VCIP135 OS=Homo sapiens GN=VCPIP1 PE=1 SV=2	sp|Q96JH7|VCIP1_HUMAN	5.7	5	1.3932	0.3846
704	Bifunctional UDP-N-acetylglucosamine 2-epimerase/N-acetylmannosamine kinase OS=Homo sapiens GN=GNE PE=1 SV=1	sp|Q9Y223|GLCNE_HUMAN	15.7	4	0.7112	0.6719
705	Abhydrolase domain-containing protein 16A OS=Homo sapiens GN=ABHD16A PE=1 SV=3	sp|O95870|ABHGA_HUMAN	13.4	4	1	0.9219

706	Keratin, type II cytoskeletal 2 epidermal OS=Homo sapiens GN=KRT2 PE=1 SV=2	sp|P35908|K22E_HUMAN	7.4	5	0.7244	0.493
707	General transcription factor II-I OS=Homo sapiens GN=GTF2I PE=1 SV=2	sp|P78347|GTF2I_HUMAN	10.2	4	0.6792	0.4131
708	Eukaryotic translation initiation factor 4H OS=Homo sapiens GN=EIF4H PE=1 SV=5	sp|Q15056|IF4H_HUMAN	35.5	4	0.2109	0.3278
709	Pyruvate dehydrogenase E1 component subunit alpha, somatic form, mitochondrial OS=Homo sapiens GN=PDHA1 PE=1	sp|P08559|ODPA_HUMAN	15.6	4	1.0375	0.8096
710	Surfeit locus protein 4 OS=Homo sapiens GN=SURF4 PE=1	sp|O15260|SURF4_HUMAN	25.7	8	1.2474	0.4973
711	DNA replication licensing factor MCM6 OS=Homo sapiens GN=MCM6 PE=1	sp|Q14566|MCM6_HUMAN	18.4	11	0.4656	0.1957
712	V-type proton ATPase subunit d 1 OS=Homo sapiens GN=ATP6V0D1 PE=1 SV=1	sp|P61421|VA0D1_HUMAN	21.1	6	1.6904	0.2476
713	Regulator of nonsense transcripts 2 OS=Homo sapiens GN=UPF2 PE=1	sp|Q9HAU5|RENT2_HUMAN	6	6	1.0375	0.9163
714	Nucleolar transcription factor 1 OS=Homo sapiens GN=UBTF PE=1 SV=1	sp|P17480|UBF1_HUMAN	10.7	5	0.7586	0.3115
715	ADP-ribosylation factor 5 OS=Homo sapiens GN=ARF5 PE=1 SV=2	sp|P84085|ARF5_HUMAN	56.7	14	0.9817	0.9866
716	Splicing factor U2AF 35 kDa subunit OS=Homo sapiens GN=U2AF1 PE=1	sp|Q01081|U2AF1_HUMAN	33.8	6	0.8091	0.8047
717	DnaJ homolog subfamily A member 2 OS=Homo sapiens GN=DNAJA2 PE=1 SV=1	sp|O60884|DNJA2_HUMAN	25.2	7	0.9727	0.9881
718	Protein quaking OS=Homo sapiens GN=QKI PE=1 SV=1	sp|Q96PU8|QKI_HUMAN	19.1	4	0.8017	0.6628
719	C-terminal-binding protein 2 OS=Homo sapiens GN=CTBP2 PE=1	sp|P56545|CTBP2_HUMAN	13.5	5	0.9376	0.4616
720	Putative helicase MOV-10 OS=Homo sapiens GN=MOV10 PE=1 SV=2	sp|Q9HCE1|MOV10_HUMAN	12.4	5	1.0666	0.4984
721	Coronin-1C OS=Homo sapiens GN=CORO1C PE=1 SV=1	sp|Q9ULV4|COR1C_HUMAN	19.8	8	1.0765	0.7377

722	Acetyl-CoA acetyltransferase, cytosolic OS=Homo sapiens GN=ACAT2 PE=1	sp|Q9BWD1|THIC_HUMAN	36.8	10	2.0137	0.4288
723	Importin subunit alpha-4 OS=Homo sapiens GN=KPNA3 PE=1 SV=2	sp|O00505|IMA4_HUMAN	18.2	4	0.8472	0.6708
724	Protein 4.1 OS=Homo sapiens GN=EPB41 PE=1 SV=4	sp|P11171|41_HUMAN	7.6	5	1.0666	0.5236
725	40S ribosomal protein S13 OS=Homo sapiens GN=RPS13 PE=1 SV=2	sp|P62277|RS13_HUMAN	45	4	0.8472	0.3198
726	Testis-expressed sequence 10 protein OS=Homo sapiens GN=TEX10 PE=1 SV=2	sp|Q9NXF1|TEX10_HUMAN	11.5	5	0.9727	0.833
727	Adenylosuccinate synthetase isozyme 1 OS=Homo sapiens GN=ADSSL1	sp|Q8N142|PURA1_HUMAN	18.2	5	0.9036	0.6818
728	Single-stranded DNA-binding protein, mitochondrial OS=Homo sapiens GN=SSBP1 PE=1 SV=1	sp|Q04837|SSBP_HUMAN	40.5	6	1.2706	0.4103
729	MORC family CW-type zinc finger protein 3 OS=Homo sapiens GN=MORC3 PE=1 SV=3	sp|Q14149|MORC3_HUMAN	8.1	4	0.9204	0.7155
730	Ragulator complex protein LAMTOR2 OS=Homo sapiens GN=LAMTOR2 PE=1 SV=1	sp|Q9Y2Q5|LTOR2_HUMAN	35.2	5	13.1826	0.113
731	Sorting nexin-2 OS=Homo sapiens GN=SNX2 PE=1 SV=2	sp|O60749|SNX2_HUMAN	19.1	6	0.9638	0.9117
732	Vasodilator-stimulated phosphoprotein OS=Homo sapiens GN=VASP PE=1 SV=3	sp|P50552|VASP_HUMAN	26.8	7	1.0375	0.8304
733	Leucine-rich repeat-containing protein 47 OS=Homo sapiens GN=LRRC47	sp|Q8N1G4|LRC47_HUMAN	21.4	10	0.9727	0.9563
734	26S proteasome non-ATPase regulatory subunit 11 OS=Homo sapiens GN=PSMD11 PE=1 SV=3	sp|O00231|PSD11_HUMAN	18.3	4	1.028	0.6463
735	Tyrosine-protein phosphatase non-receptor type 11 OS=Homo sapiens GN=PTPN11 PE=1 SV=2	sp|Q06124|PTN11_HUMAN	23	5	1.028	0.9785
736	Periodic tryptophan protein 2 homolog OS=Homo sapiens GN=PWP2 PE=2 SV=2	sp|Q15269|PWP2_HUMAN	12.5	9	0.9908	0.9719

737	Serine hydroxymethyltransferase, cytosolic OS=Homo sapiens GN=SHMT1 PE=1 SV=1	sp|P34896|GLYC_HUMAN	17.2	6	0.9376	0.6727
738	Dedicator of cytokinesis protein 7 OS=Homo sapiens GN=DOCK7 PE=1	sp|Q96N67|DOCK7_HUMAN	5.7	5	2.1478	0.1646
739	Vacuolar protein sorting-associated protein 45 OS=Homo sapiens GN=VPS45 PE=1 SV=1	sp|Q9NRW7|VPS45_HUMAN	16.8	4	1	0.9723
740	Transgelin-2 OS=Homo sapiens GN=TAGLN2 PE=1 SV=3	sp|P37802|TAGL2_HUMAN	55.3	9	0.6982	0.4588
741	ATP-dependent RNA helicase DDX24 OS=Homo sapiens GN=DDX24 PE=1	sp|Q9GZR7|DDX24_HUMAN	10	7	0.929	0.9507
742	Voltage-dependent anion-selective channel protein 2 OS=Homo sapiens GN=VDAC2 PE=1 SV=2	sp|P45880|VDAC2_HUMAN	37.8	6	1.5417	0.5511
743	Hsp90 co-chaperone Cdc37 OS=Homo sapiens GN=CDC37 PE=1	sp|Q16543|CDC37_HUMAN	15.9	5	0.9462	0.8164
744	Phosphoribosylformylglycinamidine synthase OS=Homo sapiens GN=PFAS PE=1 SV=4	sp|O15067|PUR4_HUMAN	11.1	6	0.9204	0.7759
745	GRIP1-associated protein 1 OS=Homo sapiens GN=GRIPAP1 PE=1 SV=1	sp|Q4V328|GRAP1_HUMAN	20	6	1.0965	0.6477
746	Coronin-1A OS=Homo sapiens GN=CORO1A PE=1 SV=4	sp|P31146|COR1A_HUMAN	23.6	6	0.7727	0.7411
747	Isocitrate dehydrogenase [NADP] cytoplasmic OS=Homo sapiens GN=IDH1 PE=1 SV=2	sp|O75874|IDHC_HUMAN	11.4	5	1.4723	0.4816
748	Exosome complex exonuclease RRP44 OS=Homo sapiens GN=DIS3 PE=1 SV=2	sp|Q9Y2L1|RRP44_HUMAN	13.7	5	1.1272	0.7848
749	Poly(U)-binding-splicing factor PUF60 OS=Homo sapiens GN=PUF60 PE=1 SV=1	sp|Q9UHX1|PUF60_HUMAN	21.7	5	0.4406	0.2607
750	Formin-binding protein 1 OS=Homo sapiens GN=FNBP1 PE=1	sp|Q96RU3|FNBP1_HUMAN	17.5	5	1	0.978
751	AP-3 complex subunit beta-1 OS=Homo sapiens GN=AP3B1 PE=1 SV=3	sp|O00203|AP3B1_HUMAN	10.4	7	1.0765	0.5427
752	Ubiquitin-conjugating enzyme E2 L3 OS=Homo sapiens GN=UBE2L3 PE=1 SV=1	sp|P68036|UB2L3_HUMAN	53.9	7	0.9727	0.9616

753	Mesencephalic astrocyte-derived neurotrophic factor OS=Homo sapiens GN=MANF PE=1 SV=3	sp|P55145|MANF_HUMAN	31.9	4	0.8872	0.7989
754	Active breakpoint cluster region-related protein OS=Homo sapiens GN=ABR PE=2 SV=2	sp|Q12979|ABR_HUMAN	14.1	5	1.1066	0.3998
755	Vacuolar protein sorting-associated protein 51 homolog OS=Homo sapiens GN=VPS51 PE=1 SV=2	sp|Q9UID3|VPS51_HUMAN	15.6	4	0.955	0.6807
756	Integrin alpha-M OS=Homo sapiens GN=ITGAM PE=1 SV=2	sp|P11215|ITAM_HUMAN	8.9	6	0.9817	0.9877
757	Peptidyl-prolyl cis-trans isomerase FKBP4 OS=Homo sapiens GN=FKBP4 PE=1	sp|Q02790|FKBP4_HUMAN	19	8	0.7447	0.6676
758	Presequence protease, mitochondrial OS=Homo sapiens GN=PITRM1 PE=1 SV=3	sp|Q5JRX3|PREP_HUMAN	14.1	6	0.879	0.808
759	Ornithine aminotransferase, mitochondrial OS=Homo sapiens GN=OAT PE=1 SV=1	sp|P04181|OAT_HUMAN	17.1	6	0.912	0.8713
760	Derlin-1 OS=Homo sapiens GN=DERL1 PE=1	sp|Q9BUN8|DERL1_HUMAN	15.9	4	1.4859	0.3864
761	Paraspeckle component 1 OS=Homo sapiens GN=PSPC1 PE=1 SV=1	sp|Q8WXF1|PSPC1_HUMAN	15.1	4	0.8472	0.4407
762	Coiled-coil domain-containing protein 47 OS=Homo sapiens GN=CCDC47	sp|Q96A33|CCD47_HUMAN	18.4	6	1.2359	0.6202
763	E3 ubiquitin-protein ligase HECTD1 OS=Homo sapiens GN=HECTD1	sp|Q9ULT8|HECD1_HUMAN	5.3	6	0.6668	0.4919
764	Pre-rRNA-processing protein TSR1 homolog OS=Homo sapiens GN=TSR1 PE=1 SV=1	sp|Q2NL82|TSR1_HUMAN	11	6		
765	Ubiquitin carboxyl-terminal hydrolase isozyme L5 OS=Homo sapiens GN=UCHL5 PE=1 SV=3	sp|Q9Y5K5|UCHL5_HUMAN	24.6	14	0.955	0.5921
766	Peroxisomal membrane protein 11B OS=Homo sapiens GN=PEX11B PE=1 SV=1	sp|O96011|PX11B_HUMAN	22.8	7	1.0186	0.8763
767	V-type proton ATPase 116 kDa subunit a isoform 1 OS=Homo sapiens GN=ATP6V0A1 PE=1 SV=3	sp|Q93050|VPP1_HUMAN	9.2	4	1.7061	0.2142

768	Ribosome biogenesis protein BMS1 homolog OS=Homo sapiens GN=BMS1 PE=1	sp|Q14692|BMS1_HUMAN	8.9	5	1.028	0.8965
769	Mini-chromosome maintenance complex-binding protein OS=Homo sapiens GN=MCMBP PE=1 SV=2	sp|Q9BTE3|MCMBP_HUMAN	10.9	6	0.4875	0.5132
770	Dedicator of cytokinesis protein 8 OS=Homo sapiens GN=DOCK8 PE=1	sp|Q8NF50|DOCK8_HUMAN	7.3	7	0.8318	0.6615
771	PERQ amino acid-rich with GYF domain-containing protein 2 OS=Homo sapiens GN=GIGYF2 PE=1 SV=1	sp|Q6Y7W6|PERQ2_HUMAN	9.9	5	1.2023	0.6285
772	Leucine-rich repeat-containing protein 59 OS=Homo sapiens GN=LRRC59	sp|Q96AG4|LRC59_HUMAN	20.9	3	1.1376	0.8481
773	Mothers against decapentaplegic homolog 9 OS=Homo sapiens GN=SMAD9 PE=1 SV=1	sp|O15198|SMAD9_HUMAN	11.8	3	1.0093	0.9663
774	NADH dehydrogenase [ubiquinone] flavoprotein 2, mitochondrial OS=Homo sapiens GN=NDUFV2 PE=1 SV=2	sp|P19404|NDUV2_HUMAN	28.1	4	0.9462	0.9956
775	Glycylpeptide N-tetradecanoyltransferase 1 OS=Homo sapiens GN=NMT1 PE=1 SV=2	sp|P30419|NMT1_HUMAN	18.2	4	1.406	0.8141
776	Eukaryotic translation initiation factor 3 subunit M OS=Homo sapiens GN=EIF3M PE=1	sp|Q7L2H7|EIF3M_HUMAN	20.3	10	0.929	0.8062
777	40S ribosomal protein S19 OS=Homo sapiens GN=RPS19 PE=1 SV=2	sp|P39019|RS19_HUMAN	35.2	4	0.4169	0.182
778	60S ribosomal protein L6 OS=Homo sapiens GN=RPL6 PE=1 SV=3	sp|Q02878|RL6_HUMAN	27.1	7	0.871	0.214
779	Ubiquitin carboxyl-terminal hydrolase 4 OS=Homo sapiens GN=USP4 PE=1 SV=3	sp|Q13107|UBP4_HUMAN	11.7	9	1.0568	0.6719
780	Importin subunit alpha-5 OS=Homo sapiens GN=KPNA1 PE=1 SV=3	sp|P52294|IMA5_HUMAN	14.1	5		
781	Formin-like protein 1 OS=Homo sapiens GN=FMNL1 PE=1	sp|O95466|FMNL1_HUMAN	10	8	0.9462	0.9188
782	UPF0505 protein C16orf62 OS=Homo sapiens GN=C16orf62 PE=1 SV=2	sp|Q7Z3J2|CP062_HUMAN	10.2	5	1.0864	0.6336

783	Transducin beta-like protein 3 OS=Homo sapiens GN=TBL3 PE=1	sp|Q12788|TBL3_HUMAN	20.4	5	0.929	0.578
784	Cirhin OS=Homo sapiens GN=CIRH1A PE=1 SV=1	sp|Q969X6|CIR1A_HUMAN	11.5	9	0.9376	0.6131
785	Eukaryotic translation initiation factor 2A OS=Homo sapiens GN=EIF2A PE=1 SV=3	sp|Q9BY44|EIF2A_HUMAN	14.4	5	0.6026	0.7279
786	SH3 domain-binding glutamic acid-rich-like protein OS=Homo sapiens GN=SH3BGRL	sp|O75368|SH3L1_HUMAN	50.9	5	1.2023	0.3636
787	60S ribosomal protein L27 OS=Homo sapiens GN=RPL27 PE=1 SV=2	sp|P61353|RL27_HUMAN	33.1	8	0.9036	0.8394
788	Nuclear pore complex protein Nup85 OS=Homo sapiens GN=NUP85 PE=1	sp|Q9BW27|NUP85_HUMAN	11.7	6	0.929	0.8527
789	Actin-related protein 2/3 complex subunit 3 OS=Homo sapiens GN=ARPC3 PE=1	sp|O15145|ARPC3_HUMAN	36.5	5	1.1169	0.8599
790	5'-3' exoribonuclease 1 OS=Homo sapiens GN=XRN1 PE=1 SV=1	sp|Q8IZH2|XRN1_HUMAN	6	3	1.0568	0.4727
791	DNA-(apurinic or apyrimidinic site) lyase OS=Homo sapiens GN=APEX1 PE=1	sp|P27695|APEX1_HUMAN	30.2	6	1.028	0.737
792	Tryptophan--tRNA ligase, cytoplasmic OS=Homo sapiens GN=WARS PE=1 SV=2	sp|P23381|SYWC_HUMAN	20.2	8	1.5704	0.4062
793	Poly [ADP-ribose] polymerase 1 OS=Homo sapiens GN=PARP1 PE=1 SV=4	sp|P09874|PARP1_HUMAN	12.5	16	0.912	0.7534
794	40S ribosomal protein S6 OS=Homo sapiens GN=RPS6 PE=1 SV=1	sp|P62753|RS6_HUMAN	27.7	8	0.9204	0.6397
795	DNA mismatch repair protein Msh2 OS=Homo sapiens GN=MSH2 PE=1 SV=1	sp|P43246|MSH2_HUMAN	9.2	5	0.8954	0.8434
796	COP9 signalosome complex subunit 7a OS=Homo sapiens GN=COPS7A PE=1 SV=1	sp|Q9UBW8|CSN7A_HUMAN	17.8	4	0.929	0.8606
797	60S ribosome subunit biogenesis protein NIP7 homolog OS=Homo sapiens GN=NIP7 PE=1	sp|Q9Y221|NIP7_HUMAN	35.6	6	0.929	0.8625
798	Eukaryotic translation initiation factor 6 OS=Homo sapiens GN=EIF6 PE=1	sp|P56537|IF6_HUMAN	26.1	6	1.0864	0.7517

799	Dihydrofolate reductase OS=Homo sapiens GN=DHFR PE=1 SV=2	sp|P00374|DYR_HUMAN	48.1	7	0.7379	0.5922
800	Rab3 GTPase-activating protein catalytic subunit OS=Homo sapiens GN=RAB3GAP1 PE=1 SV=3	sp|Q15042|RB3GP_HUMAN	9.4	6	1.0666	0.8569
801	Metastasis-associated protein MTA2 OS=Homo sapiens GN=MTA2 PE=1 SV=1	sp|O94776|MTA2_HUMAN	14.4	3	0.9462	0.8839
802	Xaa-Pro aminopeptidase 1 OS=Homo sapiens GN=XPNPEP1 PE=1 SV=3	sp|Q9NQW7|XPP1_HUMAN	14	3	1.0186	0.741
803	Constitutive coactivator of PPAR-gamma-like protein 1 OS=Homo sapiens GN=FAM120A PE=1 SV=2	sp|Q9NZB2|F120A_HUMAN	9.9	5	0.9638	0.7201
804	Hsc70-interacting protein OS=Homo sapiens GN=ST13 PE=1 SV=2	sp|P50502|F10A1_HUMAN	17.1	6	0.9204	0.8154
805	Nuclear pore complex protein Nup107 OS=Homo sapiens GN=NUP107 PE=1 SV=1	sp|P57740|NU107_HUMAN	6.2	3	0.879	0.8042
806	Sequestosome-1 OS=Homo sapiens GN=SQSTM1 PE=1 SV=1	sp|Q13501|SQSTM_HUMAN	32.5	24	6.0813	0.1795
807	Dedicator of cytokinesis protein 5 OS=Homo sapiens GN=DOCK5 PE=1	sp|Q9H7D0|DOCK5_HUMAN	7.4	6	1.0666	0.8788
808	Zinc finger CCCH domain-containing protein 15 OS=Homo sapiens GN=ZC3H15 PE=1 SV=1	sp|Q8WU90|ZC3HF_HUMAN	17.1	3	0.5649	0.2995
809	Phosphatidylinositide phosphatase SAC1 OS=Homo sapiens GN=SACM1L	sp|Q9NTJ5|SAC1_HUMAN	13.1	5	1.6444	0.5729
810	Nuclear pore complex protein Nup93 OS=Homo sapiens GN=NUP93 PE=1	sp|Q8N1F7|NUP93_HUMAN	12.6	6	1.0186	0.5641
811	Coiled-coil domain-containing protein 22 OS=Homo sapiens GN=CCDC22	sp|O60826|CCD22_HUMAN	14.4	7	1.0568	0.9012
812	Peripheral plasma membrane protein CASK OS=Homo sapiens GN=CASK PE=1 SV=3	sp|O14936|CSKP_HUMAN	7.8	3	0.5395	0.1877
813	Eukaryotic translation initiation factor 5B OS=Homo sapiens GN=EIF5B PE=1	sp|O60841|IF2P_HUMAN	5	4	1.028	0.7562
814	Prohibitin OS=Homo sapiens GN=PHB PE=1 SV=1	sp|P35232|PHB_HUMAN	34.2	5	1.028	0.737

815	Prefoldin subunit 5 OS=Homo sapiens GN=PFDN5 PE=1 SV=2	sp|Q99471|PFD5_HUMAN	39.6	4	0.929	0.8909
816	Sideroflexin-1 OS=Homo sapiens GN=SFXN1 PE=1 SV=4	sp|Q9H9B4|SFXN1_HUMAN	20.8	7	0.9817	0.9346
817	CCAAT/enhancer-binding protein zeta OS=Homo sapiens GN=CEBPZ PE=1 SV=3	sp|Q03701|CEBPZ_HUMAN	6	5	1.0186	0.9827
818	Dolichyl-diphosphooligosaccharide--protein glycosyltransferase subunit STT3B OS=Homo sapiens GN=STT3B PE=1 SV=1	sp|Q8TCJ2|STT3B_HUMAN	8.5	3	1.0186	0.9087
819	Peptidyl-prolyl cis-trans isomerase FKBP2 OS=Homo sapiens GN=FKBP2 PE=1	sp|P26885|FKBP2_HUMAN	38	6	0.9727	0.9229
820	Ubiquitin carboxyl-terminal hydrolase 10 OS=Homo sapiens GN=USP10 PE=1	sp|Q14694|UBP10_HUMAN	9.8	3	0.9204	0.5483
821	Hepatoma-derived growth factor OS=Homo sapiens GN=HDGF PE=1 SV=1	sp|P51858|HDGF_HUMAN	20.8	3	1.0375	0.838
822	Adapter molecule crk OS=Homo sapiens GN=CRK PE=1 SV=2	sp|P46108|CRK_HUMAN	19.1	4	1.4588	0.3394
823	Replication factor C subunit 4 OS=Homo sapiens GN=RFC4 PE=1	sp|P35249|RFC4_HUMAN	17.4	4	0.5598	0.2005
824	rRNA 2'-O-methyltransferase fibrillarin OS=Homo sapiens GN=FBL PE=1	sp|P22087|FBRL_HUMAN	31.5	4	0.9462	0.9508
825	Peroxiredoxin-1 OS=Homo sapiens GN=PRDX1 PE=1 SV=1	sp|Q06830|PRDX1_HUMAN	45.7	5	1.2246	0.6833
826	Cullin-2 OS=Homo sapiens GN=CUL2 PE=1 SV=2	sp|Q13617|CUL2_HUMAN	7.2	5	0.9817	0.9571
827	Proteasome subunit alpha type-6 OS=Homo sapiens GN=PSMA6 PE=1 SV=1	sp|P60900|PSA6_HUMAN	26.8	4	1.1588	0.7505
828	MOB-like protein phocein OS=Homo sapiens GN=MOB4 PE=1 SV=1	sp|Q9Y3A3|PHOCN_HUMAN	27.6	3	1	0.7371
829	Eukaryotic translation initiation factor 4 gamma 3 OS=Homo sapiens GN=EIF4G3 PE=1	sp|O43432|IF4G3_HUMAN	5.6	3	0.879	0.5187
830	Nucleolar protein 9 OS=Homo sapiens GN=NOP9 PE=1 SV=1	sp|Q86U38|NOP9_HUMAN	12.4	5	0.912	0.856
831	Protein disulfide-isomerase A4 OS=Homo sapiens GN=PDIA4 PE=1 SV=2	sp|P13667|PDIA4_HUMAN	16.6	11	1.1066	0.7788

832	Peptidylprolyl isomerase domain and WD repeat-containing protein 1 OS=Homo sapiens GN=PPWD1 PE=1	sp|Q96BP3|PPWD1_HUMAN	9.4	3	0.9727	0.7259
833	Notchless protein homolog 1 OS=Homo sapiens GN=NLE1 PE=1 SV=4	sp|Q9NVX2|NLE1_HUMAN	15.5	4	0.9817	0.9743
834	Carbonyl reductase [NADPH] 1 OS=Homo sapiens GN=CBR1 PE=1 SV=3	sp|P16152|CBR1_HUMAN	27.8	5	1.2589	0.5706
835	Serine/threonine-protein phosphatase 1 regulatory subunit 10 OS=Homo sapiens GN=PPP1R10 PE=1 SV=1	sp|Q96QC0|PP1RA_HUMAN	13.8	5	0.6486	0.2821
836	Signal recognition particle 54 kDa protein OS=Homo sapiens GN=SRP54 PE=1 SV=1	sp|P61011|SRP54_HUMAN	17.9	4	0.9638	0.8032
837	Nuclear receptor corepressor 1 OS=Homo sapiens GN=NCOR1 PE=1 SV=2	sp|O75376|NCOR1_HUMAN	5.2	5	0.8872	0.8633
838	GTP-binding protein SAR1b OS=Homo sapiens GN=SAR1B PE=1	sp|Q9Y6B6|SAR1B_HUMAN	40.9	5	0.9727	0.8857
839	Ubiquitin-like modifier-activating enzyme ATG7 OS=Homo sapiens GN=ATG7 PE=1 SV=1	sp|O95352|ATG7_HUMAN	7.3	3	2.0512	0.4856
840	Apoptosis inhibitor 5 OS=Homo sapiens GN=API5 PE=1 SV=3	sp|Q9BZZ5|API5_HUMAN	15.3	33	0.7943	0.777
841	Glycine--tRNA ligase OS=Homo sapiens GN=GARS PE=1 SV=3	sp|P41250|SYG_HUMAN	16.9	7	1.5704	0.3618
842	Serine/threonine-protein phosphatase 4 regulatory subunit 3A OS=Homo sapiens GN=SMEK1 PE=1	sp|Q6IN85|P4R3A_HUMAN	7.8	3	0.8551	0.6202
843	Heterogeneous nuclear ribonucleoprotein U-like protein 1 OS=Homo sapiens GN=HNRNPUL1 PE=1 SV=2	sp|Q9BUJ2|HNRL1_HUMAN	11.7	3	0.9908	0.7849
844	Serine/arginine repetitive matrix protein 2 OS=Homo sapiens GN=SRRM2 PE=1	sp|Q9UQ35|SRRM2_HUMAN	5.3	5	0.9817	0.9794
845	Hexokinase-1 OS=Homo sapiens GN=HK1 PE=1 SV=3	sp|P19367|HXK1_HUMAN	17.2	12	1.028	0.8559
846	Proteasome activator complex subunit 1 OS=Homo sapiens GN=PSME1 PE=1	sp|Q06323|PSME1_HUMAN	27.7	10	1.0965	0.638
847	La-related protein 4 OS=Homo sapiens GN=LARP4 PE=1 SV=3	sp|Q71RC2|LARP4_HUMAN	7.2	4	0.7244	0.5626

848	Coiled-coil domain-containing protein 58 OS=Homo sapiens GN=CCDC58	sp|Q4VC31|CCD58_HUMAN	53.5	5	1.0765	0.8371
849	Polypyrimidine tract-binding protein 3 OS=Homo sapiens GN=PTBP3 PE=1	sp|O95758|PTBP3_HUMAN	22.6	6	1.0666	0.8571
850	Short-chain specific acyl-CoA dehydrogenase, mitochondrial OS=Homo sapiens GN=ACADS PE=1 SV=1	sp|P16219|ACADS_HUMAN	24.8	6	1.0568	0.9063
851	Protein unc-45 homolog A OS=Homo sapiens GN=UNC45A PE=1 SV=1	sp|Q9H3U1|UN45A_HUMAN	8.6	4	0.9204	0.8619
852	ADP-ribosylation factor-like protein 8B OS=Homo sapiens GN=ARL8B PE=1	sp|Q9NVJ2|ARL8B_HUMAN	23.1	5	1.2246	0.6682
853	Proliferating cell nuclear antigen OS=Homo sapiens GN=PCNA PE=1 SV=1	sp|P12004|PCNA_HUMAN	24.9	6	0.3802	0.26
854	LisH domain and HEAT repeat-containing protein KIAA1468 OS=Homo sapiens GN=KIAA1468 PE=1 SV=2	sp|Q9P260|K1468_HUMAN	6.9	3	1.6749	0.4065
855	Pyruvate carboxylase, mitochondrial OS=Homo sapiens GN=PC PE=1	sp|P11498|PYC_HUMAN	9.7	6	1.4859	0.5251
856	Interferon regulatory factor 2-binding protein-like OS=Homo sapiens GN=IRF2BPL PE=1 SV=1	sp|Q9H1B7|I2BPL_HUMAN	13.1	5	1.0093	0.7169
857	Double-stranded RNA-specific adenosine deaminase OS=Homo sapiens GN=ADAR PE=1 SV=4	sp|P55265|DSRAD_HUMAN	9.1	4	1.0864	0.7293
858	GEM-interacting protein OS=Homo sapiens GN=GMIP PE=1 SV=2	sp|Q9P107|GMIP_HUMAN	11.8	4	0.8395	0.7761
859	NAD-dependent malic enzyme, mitochondrial OS=Homo sapiens GN=ME2 PE=1 SV=1	sp|P23368|MAOM_HUMAN	12.5	3	1.0093	0.9245
860	ER membrane protein complex subunit 1 OS=Homo sapiens GN=EMC1 PE=1	sp|Q8N766|EMC1_HUMAN	9.4	3	0.871	0.7713
861	Ubiquitin fusion degradation protein 1 homolog OS=Homo sapiens GN=UFD1L PE=1 SV=3	sp|Q92890|UFD1_HUMAN	16.3	3	0.9638	0.853
862	AP-1 complex subunit gamma-like 2 OS=Homo sapiens GN=AP1G2 PE=1	sp|O75843|AP1G2_HUMAN	11.1	4	0.871	0.6513

863	E3 ubiquitin-protein ligase UBR5 OS=Homo sapiens GN=UBR5 PE=1 SV=2	sp|O95071|UBR5_HUMAN	4.3	4	0.8551	0.7859
864	Galectin-1 OS=Homo sapiens GN=LGALS1 PE=1 SV=2	sp|P09382|LEG1_HUMAN	39.3	3	1.6749	0.4028
865	Serine/threonine-protein phosphatase 6 regulatory ankyrin repeat subunit C OS=Homo sapiens GN=ANKRD52	sp|Q8NB46|ANR52_HUMAN	10.4	3	0.5861	0.4715
866	DNA-directed RNA polymerase III subunit RPC1 OS=Homo sapiens GN=POLR3A PE=1 SV=2	sp|O14802|RPC1_HUMAN	9.5	4	0.912	0.6624
867	Integrator complex subunit 10 OS=Homo sapiens GN=INTS10 PE=1 SV=2	sp|Q9NVR2|INT10_HUMAN	8.3	7	0.9204	0.8309
868	Rho guanine nucleotide exchange factor 1 OS=Homo sapiens GN=ARHGEF1 PE=1 SV=2	sp|Q92888|ARHG1_HUMAN	12	3	1.0666	0.8732
869	Rho GTPase-activating protein 1 OS=Homo sapiens GN=ARHGAP1 PE=1 SV=1	sp|Q07960|RHG01_HUMAN	24.8	10	1.2942	0.5035
870	COP9 signalosome complex subunit 6 OS=Homo sapiens GN=COPS6 PE=1 SV=1	sp|Q7L5N1|CSN6_HUMAN	22	5	1	0.9997
871	[Pyruvate dehydrogenase (acetyl-transferring)] kinase isozyme 3, mitochondrial OS=Homo sapiens GN=PDK3 PE=1 SV=1	sp|Q15120|PDK3_HUMAN	14.3	4	1	0.8849
872	Nuclear RNA export factor 1 OS=Homo sapiens GN=NXF1 PE=1	sp|Q9UBU9|NXF1_HUMAN	14.7	5	0.9727	0.9204
873	Eukaryotic translation initiation factor 4B OS=Homo sapiens GN=EIF4B PE=1	sp|P23588|IF4B_HUMAN	12.3	3	0.8017	0.6733
874	Niban-like protein 1 OS=Homo sapiens GN=FAM129B	sp|Q96TA1|NIBL1_HUMAN	10.3	3	0.9204	0.719
875	Sorting nexin-9 OS=Homo sapiens GN=SNX9 PE=1 SV=1	sp|Q9Y5X1|SNX9_HUMAN	13.6	4	1.0666	0.7055
876	Leucine-rich repeat-containing protein 40 OS=Homo sapiens GN=LRRC40	sp|Q9H9A6|LRC40_HUMAN	13	4	0.8318	0.6372
877	UPF0668 protein C10orf76 OS=Homo sapiens GN=C10orf76 PE=2 SV=1	sp|Q5T2E6|CJ076_HUMAN	8	3	1.2023	0.5414
878	Vesicle-trafficking protein SEC22b OS=Homo sapiens GN=SEC22B PE=1 SV=4	sp|O75396|SC22B_HUMAN	28.4	4	1.2706	0.5903

879	40S ribosomal protein S12 OS=Homo sapiens GN=RPS12 PE=1 SV=3	sp|P25398|RS12_HUMAN	47.7	24	0.8954	0.8286
880	Histone H2A.Z OS=Homo sapiens GN=H2AFZ PE=1 SV=2	sp|P0C0S5|H2AZ_HUMAN	75.8	5	0.787	0.9117
881	Non-specific lipid-transfer protein OS=Homo sapiens GN=SCP2 PE=1 SV=2	sp|P22307|NLTP_HUMAN	17	5	0.9462	0.8289
882	NudC domain-containing protein 1 OS=Homo sapiens GN=NUDCD1	sp|Q96RS6|NUDC1_HUMAN	9.9	4	0.9727	0.9529
883	Vacuolar protein sorting-associated protein 16 homolog OS=Homo sapiens GN=VPS16 PE=1 SV=2	sp|Q9H269|VPS16_HUMAN	8.9	3	1.0568	0.864
884	SWI/SNF-related matrix-associated actin-dependent regulator of chromatin subfamily D member 2 OS=Homo sapiens GN=SMARCD2 PE=1 SV=3	sp|Q92925|SMRD2_HUMAN	10	4	0.929	0.7174
885	Matrin-3 OS=Homo sapiens GN=MATR3 PE=1 SV=2	sp|P43243|MATR3_HUMAN	19.4	11	0.8954	0.7324
886	Transmembrane 9 superfamily member 4 OS=Homo sapiens GN=TM9SF4 PE=1 SV=2	sp|Q92544|TM9S4_HUMAN	10.1	3	2.421	0.4817
887	von Willebrand factor A domain-containing protein 8 OS=Homo sapiens GN=VWA8 PE=1	sp|A3KMH1|VWA8_HUMAN	5.1	3	1.1169	0.8397
888	Rab GTPase-binding effector protein 1 OS=Homo sapiens GN=RABEP1	sp|Q15276|RABE1_HUMAN	7.8	4	1.1588	0.5216
889	Ancient ubiquitous protein 1 OS=Homo sapiens GN=AUP1 PE=1	sp|Q9Y679|AUP1_HUMAN	15.1	4	1.1482	0.7762
890	Mitochondrial carrier homolog 1 OS=Homo sapiens GN=MTCH1 PE=1 SV=1	sp|Q9NZJ7|MTCH1_HUMAN	18	4	1.406	0.5076
891	WD repeat-containing protein 3 OS=Homo sapiens GN=WDR3 PE=1	sp|Q9UNX4|WDR3_HUMAN	8.3	4	0.8318	0.5923
892	Mitogen-activated protein kinase 14 OS=Homo sapiens GN=MAPK14 PE=1 SV=3	sp|Q16539|MK14_HUMAN	24.7	3	1.0375	0.4581
893	Annexin A1 OS=Homo sapiens GN=ANXA1 PE=1 SV=2	sp|P04083|ANXA1_HUMAN	26.6	14	1.0864	0.7937

894	2-amino-3-ketobutyrate coenzyme A ligase, mitochondrial OS=Homo sapiens GN=GCAT PE=1 SV=1	sp|O75600|KBL_HUMAN	27.5	16	1.028	0.938
895	Parafibromin OS=Homo sapiens GN=CDC73 PE=1 SV=1	sp|Q6P1J9|CDC73_HUMAN	9	3	0.8091	0.5916
896	Clathrin interactor 1 OS=Homo sapiens GN=CLINT1 PE=1	sp|Q14677|EPN4_HUMAN	9.3	4	0.6427	0.9405
897	U2 small nuclear ribonucleoprotein A' OS=Homo sapiens GN=SNRPA1	sp|P09661|RU2A_HUMAN	19.6	3	0.879	0.5599
898	SWI/SNF-related matrix-associated actin-dependent regulator of chromatin subfamily E member 1 OS=Homo sapiens GN=SMARCE1 PE=1 SV=2	sp|Q969G3|SMCE1_HUMAN	17.5	3	1.2023	0.5467
899	Tubulin beta-4B chain OS=Homo sapiens GN=TUBB4B PE=1 SV=1	sp|P68371|TBB4B_HUMAN	69.9	69	0.8017	0.597
900	Histone H3.1 OS=Homo sapiens GN=HIST1H3A PE=1 SV=2	sp|P68431|H31_HUMAN	77.9	25	0.4831	0.4578
901	Ras-related protein Rap-1b OS=Homo sapiens GN=RAP1B PE=1	sp|P61224|RAP1B_HUMAN	60.9	11	1.5276	0.6065
902	14-3-3 protein theta OS=Homo sapiens GN=YWHAQ PE=1 SV=1	sp|P27348|1433T_HUMAN	40.8	13	1.0093	0.9453
903	Ubiquilin-4 OS=Homo sapiens GN=UBQLN4 PE=1 SV=2	sp|Q9NRR5|UBQL4_HUMAN	18.8	5	0.7516	0.4092
904	Putative RNA-binding protein Luc7-like 1 OS=Homo sapiens GN=LUC7L PE=1 SV=1	sp|Q9NQ29|LUC7L_HUMAN	17	4	0.7379	0.3896
905	Cation-independent mannose-6-phosphate receptor OS=Homo sapiens GN=IGF2R PE=1 SV=3	sp|P11717|MPRI_HUMAN	4.4	4	1.4859	0.5179
906	Phosphoribosyl pyrophosphate synthase-associated protein 1 OS=Homo sapiens GN=PRPSAP1 PE=1 SV=2	sp|Q14558|KPRA_HUMAN	17.4	5	0.929	0.8362
907	Procollagen-lysine,2-oxoglutarate 5-dioxygenase 1 OS=Homo sapiens GN=PLOD1 PE=1 SV=2	sp|Q02809|PLOD1_HUMAN	8.5	4	0.9376	0.9331
908	Structural maintenance of chromosomes protein 1A OS=Homo sapiens GN=SMC1A PE=1 SV=2	sp|Q14683|SMC1A_HUMAN	12.4	4	1.0765	0.9462

909	Septin-2 OS=Homo sapiens GN=SEPT2 PE=1	sp|Q15019|SEPT2_HUMAN	20.8	7	1	0.9135
910	Phosphatidylinositol 3-kinase catalytic subunit type 3 OS=Homo sapiens GN=PIK3C3 PE=1 SV=1	sp|Q8NEB9|PK3C3_HUMAN	8.7	4	1.0568	0.6349
911	Adenosylhomocysteinase OS=Homo sapiens GN=AHCY PE=1 SV=4	sp|P23526|SAHH_HUMAN	18.1	4	0.929	0.871
912	Dolichyl-diphosphooligosaccharide--protein glycosyltransferase subunit STT3A OS=Homo sapiens GN=STT3A PE=1 SV=2	sp|P46977|STT3A_HUMAN	9.1	4	1.2246	0.5464
913	Heat shock 70 kDa protein 14 OS=Homo sapiens GN=HSPA14 PE=1 SV=1	sp|Q0VDF9|HSP7E_HUMAN	11.2	4	0.9817	0.9457
914	Very-long-chain (3R)-3-hydroxyacyl-CoA dehydratase 3 OS=Homo sapiens GN=HACD3 PE=1 SV=2	sp|Q9P035|HACD3_HUMAN	17.7	4	1.0965	0.6371
915	Methionine adenosyltransferase 2 subunit beta OS=Homo sapiens GN=MAT2B PE=1 SV=1	sp|Q9NZL9|MAT2B_HUMAN	12.9	4	1.0093	0.9821
916	Rap1 GTPase-GDP dissociation stimulator 1 OS=Homo sapiens GN=RAP1GDS1 PE=1 SV=3	sp|P52306|GDS1_HUMAN	16.1	5	0.9462	0.6363
917	Nucleobindin-1 OS=Homo sapiens GN=NUCB1 PE=1 SV=4	sp|Q02818|NUCB1_HUMAN	12.2	5	1.5276	0.8224
918	Dynamin-like 120 kDa protein, mitochondrial OS=Homo sapiens GN=OPA1 PE=1 SV=3	sp|O60313|OPA1_HUMAN	7.3	3	0.5346	0.4549
919	Unconventional myosin-Ic OS=Homo sapiens GN=MYO1C PE=1 SV=4	sp|O00159|MYO1C_HUMAN	9.1	4	1.1695	0.6815
920	Catechol O-methyltransferase OS=Homo sapiens GN=COMT PE=1 SV=2	sp|P21964|COMT_HUMAN	28.4	4	1.1272	0.7853
921	ADP-ribosylation factor 6 OS=Homo sapiens GN=ARF6 PE=1 SV=2	sp|P62330|ARF6_HUMAN	30.3	3	0.9727	0.9156
922	Cleavage stimulation factor subunit 2 OS=Homo sapiens GN=CSTF2 PE=1	sp|P33240|CSTF2_HUMAN	18	4	1.0471	0.9249
923	Nuclear export mediator factor NEMF OS=Homo sapiens GN=NEMF PE=1 SV=4	sp|O60524|NEMF_HUMAN	4.9	3	1	0.8578
924	Proteasome activator complex subunit 4 OS=Homo sapiens GN=PSME4 PE=1	sp|Q14997|PSME4_HUMAN	6.2	3	1.0965	0.5044

925	tRNA pseudouridine synthase A, mitochondrial OS=Homo sapiens GN=PUS1 PE=1	sp|Q9Y606|TRUA_HUMAN	16.9	5	0.9462	0.8775
926	Uridine 5'-monophosphate synthase OS=Homo sapiens GN=UMPS PE=1 SV=1	sp|P11172|UMPS_HUMAN	14.6	3	0.6918	0.4773
927	Dynactin subunit 4 OS=Homo sapiens GN=DCTN4 PE=1 SV=1	sp|Q9UJW0|DCTN4_HUMAN	8.3	3	1.0093	0.7823
928	Cytosolic acyl coenzyme A thioester hydrolase OS=Homo sapiens GN=ACOT7 PE=1 SV=3	sp|O00154|BACH_HUMAN	9.7	3	1	0.8048
929	Unconventional myosin-Ig OS=Homo sapiens GN=MYO1G PE=1 SV=2	sp|B0I1T2|MYO1G_HUMAN	9.4	4	1.1272	0.7407
930	Myelin expression factor 2 OS=Homo sapiens GN=MYEF2 PE=1	sp|Q9P2K5|MYEF2_HUMAN	15	3	1.0375	0.7638
931	Mannosyl-oligosaccharide glucosidase OS=Homo sapiens GN=MOGS PE=1 SV=5	sp|Q13724|MOGS_HUMAN	8.2	4	1.0471	0.8752
932	Syndetin OS=Homo sapiens GN=CCDC132 PE=1 SV=3	sp|Q96JG6|SYNDE_HUMAN	5.2	4	0.9908	0.9719
933	U3 small nucleolar RNA-associated protein 15 homolog OS=Homo sapiens GN=UTP15 PE=1 SV=3	sp|Q8TED0|UTP15_HUMAN	13.1	3	0.9817	0.9941
934	Transaldolase OS=Homo sapiens GN=TALDO1 PE=1 SV=2	sp|P37837|TALDO_HUMAN	14.5	4	1.1066	0.8717
935	Ras-related protein Rab-10 OS=Homo sapiens GN=RAB10 PE=1	sp|P61026|RAB10_HUMAN	34	4	1.2823	0.8285
936	SEC23-interacting protein OS=Homo sapiens GN=SEC23IP PE=1 SV=1	sp|Q9Y6Y8|S23IP_HUMAN	5.5	3	1.0666	0.8867
937	40S ribosomal protein S14 OS=Homo sapiens GN=RPS14 PE=1 SV=3	sp|P62263|RS14_HUMAN	33.8	4	0.9908	0.9853
938	Vacuolar protein-sorting-associated protein 36 OS=Homo sapiens GN=VPS36 PE=1 SV=1	sp|Q86VN1|VPS36_HUMAN	14.3	3	0.955	0.6786
939	Centromere protein V OS=Homo sapiens GN=CENPV PE=1 SV=1	sp|Q7Z7K6|CENPV_HUMAN	18.6	3	1.028	0.9033
940	Striatin-interacting protein 1 OS=Homo sapiens GN=STRIP1 PE=1	sp|Q5VSL9|STRP1_HUMAN	7	3	0.9817	0.8484
941	Protein CutA OS=Homo sapiens GN=CUTA PE=1 SV=2	sp|O60888|CUTA_HUMAN	27.4	3	1.0093	0.8231

942	Nucleolar protein 6 OS=Homo sapiens GN=NOL6 PE=1 SV=2	sp|Q9H6R4|NOL6_HUMAN	8.8	4	0.9204	0.9725
943	Trafficking protein particle complex subunit 8 OS=Homo sapiens GN=TRAPPC8 PE=1 SV=2	sp|Q9Y2L5|TPPC8_HUMAN	6.3	3	1	0.9304
944	Pre-mRNA-splicing factor ATP-dependent RNA helicase PRP16 OS=Homo sapiens GN=DHX38 PE=1	sp|Q92620|PRP16_HUMAN	7.3	3	0.7447	0.5405
945	Guanine nucleotide-binding protein G(s) subunit alpha isoforms XLas OS=Homo sapiens GN=GNAS PE=1	sp|Q5JWF2|GNAS1_HUMAN	8.3	3	1.7061	0.4168
946	IQ motif and SEC7 domain-containing protein 1 OS=Homo sapiens GN=IQSEC1 PE=1 SV=1	sp|Q6DN90|IQEC1_HUMAN	8.6	3	0.5445	0.399
947	NADP-dependent malic enzyme OS=Homo sapiens GN=ME1 PE=1 SV=1	sp|P48163|MAOX_HUMAN	18.7	3	1.4322	0.4977
948	CCA tRNA nucleotidyltransferase 1, mitochondrial OS=Homo sapiens GN=TRNT1 PE=1 SV=2	sp|Q96Q11|TRNT1_HUMAN	13.6	3	0.9817	0.9066
949	Cohesin subunit SA-2 OS=Homo sapiens GN=STAG2 PE=1	sp|Q8N3U4|STAG2_HUMAN	5.2	4	0.9727	0.8943
950	Dual specificity mitogen-activated protein kinase kinase 2 OS=Homo sapiens GN=MAP2K2	sp|P36507|MP2K2_HUMAN	17	3	1.0765	0.6187
951	STE20-like serine/threonine-protein kinase OS=Homo sapiens GN=SLK PE=1 SV=1	sp|Q9H2G2|SLK_HUMAN	6	4	0.863	0.7166
952	Polyadenylate-binding protein-interacting protein 1 OS=Homo sapiens GN=PAIP1 PE=1 SV=1	sp|Q9H074|PAIP1_HUMAN	17.8	3	1.028	0.6485
953	Minor histocompatibility protein HA-1 OS=Homo sapiens GN=HMHA1 PE=1 SV=2	sp|Q92619|HMHA1_HUMAN	9.3	16	0.9462	0.9353
954	U4/U6.U5 tri-snRNP-associated protein 2 OS=Homo sapiens GN=USP39 PE=1	sp|Q53GS9|SNUT2_HUMAN	11.2	3	0.6855	0.4647
955	Succinyl-CoA:3-ketoacid coenzyme A transferase 1, mitochondrial OS=Homo sapiens GN=OXCT1 PE=1 SV=1	sp|P55809|SCOT1_HUMAN	19.6	7		

956	Transcription factor BTF3 OS=Homo sapiens GN=BTF3 PE=1 SV=1	sp|P20290|BTF3_HUMAN	49	6	0.2188	0.1856
957	THUMP domain-containing protein 1 OS=Homo sapiens GN=THUMPD1	sp|Q9NXG2|THUM1_HUMAN	13.6	3	0.9817	0.9313
958	Transformer-2 protein homolog beta OS=Homo sapiens GN=TRA2B PE=1	sp|P62995|TRA2B_HUMAN	26.7	3	0.9727	0.9596
959	Fragile X mental retardation syndrome-related protein 1 OS=Homo sapiens GN=FXR1 PE=1	sp|P51114|FXR1_HUMAN	11.4	3	0.9727	0.852
960	Transferrin receptor protein 1 OS=Homo sapiens GN=TFRC PE=1	sp|P02786|TFR1_HUMAN	6.8	4	0.3802	0.3645
961	Choline transporter-like protein 1 OS=Homo sapiens GN=SLC44A1	sp|Q8WWI5|CTL1_HUMAN	8.5	3	1.1588	0.9401
962	Transmembrane emp24 domain-containing protein 4 OS=Homo sapiens GN=TMED4 PE=1	sp|Q7Z7H5|TMED4_HUMAN	20.7	3	1.1695	0.6012
963	PCI domain-containing protein 2 OS=Homo sapiens GN=PCID2 PE=1 SV=2	sp|Q5JVF3|PCID2_HUMAN	11.8	3	0.9638	0.9022
964	Protein FAM98B OS=Homo sapiens GN=FAM98B PE=1 SV=1	sp|Q52LJ0|FA98B_HUMAN	14.9	3	0.955	0.9084
965	Nucleolysin TIAR OS=Homo sapiens GN=TIAL1 PE=1 SV=1	sp|Q01085|TIAR_HUMAN	18.9	5	0.9817	0.9672
966	Conserved oligomeric Golgi complex subunit 7 OS=Homo sapiens GN=COG7 PE=1 SV=1	sp|P83436|COG7_HUMAN	7.3	3	0.9817	0.9462
967	Methionine aminopeptidase 2 OS=Homo sapiens GN=METAP2 PE=1 SV=1	sp|P50579|MAP2_HUMAN	11.7	3	0.871	0.7535
968	Selenide, water dikinase 1 OS=Homo sapiens GN=SEPHS1 PE=1 SV=2	sp|P49903|SPS1_HUMAN	18.4	3	1.0864	0.7711
969	Coatomer subunit delta OS=Homo sapiens GN=ARCN1 PE=1	sp|P48444|COPD_HUMAN	10	4	1.028	0.8711
970	Proteasome subunit alpha type-4 OS=Homo sapiens GN=PSMA4 PE=1 SV=1	sp|P25789|PSA4_HUMAN	28.4	7	1.0375	0.9738
971	Beta-hexosaminidase subunit alpha OS=Homo sapiens GN=HEXA PE=1 SV=2	sp|P06865|HEXA_HUMAN	9.6	3	2.0512	0.2331
972	Peroxisomal membrane protein PEX14 OS=Homo sapiens GN=PEX14 PE=1	sp|O75381|PEX14_HUMAN	12.7	3	1.0375	0.9687

973	Isocitrate dehydrogenase [NAD] subunit beta, mitochondrial OS=Homo sapiens GN=IDH3B PE=1 SV=2	sp|O43837|IDH3B_HUMAN	11.4	5	1.0093	0.9517
974	Inhibitor of nuclear factor kappa-B kinase subunit alpha OS=Homo sapiens GN=CHUK PE=1 SV=2	sp|O15111|IKKA_HUMAN	5.6	3	1.028	0.9579
975	Phosphoglycolate phosphatase OS=Homo sapiens GN=PGP PE=1 SV=1	sp|A6NDG6|PGP_HUMAN	19	3	1	0.9323
976	E3 ubiquitin-protein ligase ARIH1 OS=Homo sapiens GN=ARIH1 PE=1	sp|Q9Y4X5|ARI1_HUMAN	6.8	3	0.9817	0.9182
977	Endophilin-B1 OS=Homo sapiens GN=SH3GLB1 PE=1 SV=1	sp|Q9Y371|SHLB1_HUMAN	13.7	3	1.0666	0.5956
978	Probable dimethyladenosine transferase OS=Homo sapiens GN=DIMT1 PE=1 SV=1	sp|Q9UNQ2|DIM1_HUMAN	13.1	3	0.8318	0.6039
979	DCC-interacting protein 13-alpha OS=Homo sapiens GN=APPL1 PE=1 SV=1	sp|Q9UKG1|DP13A_HUMAN	6.8	3	0.9638	0.9826
980	Pre-mRNA-splicing factor 38A OS=Homo sapiens GN=PRPF38A PE=1 SV=1	sp|Q8NAV1|PR38A_HUMAN	14.7	3	0.9204	0.624
981	Putative E3 ubiquitin-protein ligase UBR7 OS=Homo sapiens GN=UBR7 PE=1 SV=2	sp|Q8N806|UBR7_HUMAN	9.6	3	0.7798	0.3544
982	WD repeat-containing protein 43 OS=Homo sapiens GN=WDR43 PE=1	sp|Q15061|WDR43_HUMAN	9.7	5	0.6607	0.585
983	Proteasome subunit beta type-2 OS=Homo sapiens GN=PSMB2 PE=1 SV=1	sp|P49721|PSB2_HUMAN	22.9	3	1.0186	0.9587
984	Casein kinase I isoform delta OS=Homo sapiens GN=CSNK1D PE=1 SV=2	sp|P48730|KC1D_HUMAN	11.1	3	0.9204	0.771
985	Replication factor C subunit 5 OS=Homo sapiens GN=RFC5 PE=1	sp|P40937|RFC5_HUMAN	20.3	4	0.6026	0.3349
986	Mitotic checkpoint protein BUB3 OS=Homo sapiens GN=BUB3 PE=1 SV=1	sp|O43684|BUB3_HUMAN	15.6	3	0.5105	0.2577
987	Mannose-1-phosphate guanyltransferase beta OS=Homo sapiens GN=GMPPB PE=1 SV=2	sp|Q9Y5P6|GMPPB_HUMAN	15	3	1.0375	0.8739

988	UPF0568 protein C14orf166 OS=Homo sapiens GN=C14orf166 PE=1 SV=1	sp|Q9Y224|CN166_HUMAN	18.9	3	1	0.9656
989	Integrator complex subunit 9 OS=Homo sapiens GN=INTS9 PE=1	sp|Q9NV88|INT9_HUMAN	8.7	3	0.8395	0.5346
990	Calcium-binding protein 39-like OS=Homo sapiens GN=CAB39L PE=1 SV=3	sp|Q9H9S4|CB39L_HUMAN	9.5	3	1.0093	0.9823
991	Gamma-soluble NSF attachment protein OS=Homo sapiens GN=NAPG PE=1 SV=1	sp|Q99747|SNAG_HUMAN	16.7	3	1.0093	0.9676
992	Dynein light chain 2, cytoplasmic OS=Homo sapiens GN=DYNLL2 PE=1 SV=1	sp|Q96FJ2|DYL2_HUMAN	42.7	5	0.955	0.9248
993	Protein FAM45B OS=Homo sapiens GN=FAM45B PE=2 SV=1	sp|Q6NSW5|FA45B_HUMAN	12	5	1.0765	0.7971
994	Ubiquitin-conjugating enzyme E2 variant 2 OS=Homo sapiens GN=UBE2V2	sp|Q15819|UB2V2_HUMAN	31	6	0.9638	0.9919
995	Transcriptional activator protein Pur-alpha OS=Homo sapiens GN=PURA PE=1 SV=2	sp|Q00577|PURA_HUMAN	25.5	5	1.2589	0.7324
996	Rho-related GTP-binding protein RhoG OS=Homo sapiens GN=RHOG PE=1 SV=1	sp|P84095|RHOG_HUMAN	28.8	5	2.208	0.2489
997	Casein kinase II subunit beta OS=Homo sapiens GN=CSNK2B PE=1 SV=1	sp|P67870|CSK2B_HUMAN	19.1	3	0.9727	0.9779
998	Isocitrate dehydrogenase [NAD] subunit gamma, mitochondrial OS=Homo sapiens GN=IDH3G PE=1 SV=1	sp|P51553|IDH3G_HUMAN	16.5	8	0.9727	0.971
999	Dual specificity protein phosphatase 3 OS=Homo sapiens GN=DUSP3 PE=1	sp|P51452|DUS3_HUMAN	22.2	3	1.1169	0.7914
1000	Paxillin OS=Homo sapiens GN=PXN PE=1 SV=3	sp|P49023|PAXI_HUMAN	7.4	3	0.9036	0.7043
1001	Casein kinase I isoform alpha OS=Homo sapiens GN=CSNK1A1 PE=1 SV=2	sp|P48729|KC1A_HUMAN	11.3	3	0.7943	0.676
1002	Dual specificity mitogen-activated protein kinase kinase 3 OS=Homo sapiens GN=MAP2K3	sp|P46734|MP2K3_HUMAN	15.9	4	0.5861	0.5659
1003	Eukaryotic translation initiation factor 1 OS=Homo sapiens GN=EIF1 PE=1	sp|P41567|EIF1_HUMAN	48.7	3	0.7586	0.6155

1004	Heterogeneous nuclear ribonucleoprotein H3 OS=Homo sapiens GN=HNRNPH3 PE=1 SV=2	sp|P31942|HNRH3_HUMAN	15.3	3	1.0568	0.8265
1005	Succinate dehydrogenase [ubiquinone] iron-sulfur subunit, mitochondrial OS=Homo sapiens GN=SDHB PE=1 SV=3	sp|P21912|SDHB_HUMAN	14.6	3	0.955	0.9344
1006	Thymidylate synthase OS=Homo sapiens GN=TYMS PE=1 SV=3	sp|P04818|TYSY_HUMAN	15.3	5	0.4966	0.3919
1007	Histone acetyltransferase KAT7 OS=Homo sapiens GN=KAT7 PE=1 SV=1	sp|O95251|KAT7_HUMAN	7.5	6	0.8091	0.5435
1008	Huntingtin-interacting protein K OS=Homo sapiens GN=HYPK PE=1 SV=2	sp|Q9NX55|HYPK_HUMAN	24.8	3	0.8395	0.9342
1009	Nucleoporin SEH1 OS=Homo sapiens GN=SEH1L PE=1 SV=3	sp|Q96EE3|SEH1_HUMAN	15.3	4	1.0093	0.8998
1010	Ribulose-phosphate 3-epimerase OS=Homo sapiens GN=RPE PE=1 SV=1	sp|Q96AT9|RPE_HUMAN	14.5	3	1.1169	0.5709
1011	U4/U6 small nuclear ribonucleoprotein Prp31 OS=Homo sapiens GN=PRPF31 PE=1	sp|Q8WWY3|PRP31_HUMAN	8.2	3	0.5649	0.3945
1012	Serine/arginine-rich splicing factor 6 OS=Homo sapiens GN=SRSF6 PE=1 SV=2	sp|Q13247|SRSF6_HUMAN	10.5	3	0.9376	0.8836
1013	Protein Dr1 OS=Homo sapiens GN=DR1 PE=1 SV=1	sp|Q01658|NC2B_HUMAN	29	3	1.0471	0.8686
1014	Enhancer of rudimentary homolog OS=Homo sapiens GN=ERH PE=1	sp|P84090|ERH_HUMAN	32.7	4	0.8241	0.7409
1015	28S ribosomal protein S9, mitochondrial OS=Homo sapiens GN=MRPS9 PE=1 SV=2	sp|P82933|RT09_HUMAN	9.3	3	0.9817	0.9253
1016	Protein BUD31 homolog OS=Homo sapiens GN=BUD31 PE=1	sp|P41223|BUD31_HUMAN	14.6	3	0.5152	0.2252
1017	Importin subunit alpha-3 OS=Homo sapiens GN=KPNA4 PE=1 SV=1	sp|O00629|IMA3_HUMAN	10	3	0.8872	0.8502
1018	Pinin OS=Homo sapiens GN=PNN PE=1 SV=4	sp|Q9H307|PININ_HUMAN	9.5	3	0.3908	0.2302
1019	ARF GTPase-activating protein GIT1 OS=Homo sapiens GN=GIT1 PE=1 SV=2	sp|Q9Y2X7|GIT1_HUMAN	4.2	4		

1020	Ras-related protein Rab-1B OS=Homo sapiens GN=RAB1B PE=1	sp|Q9H0U4|RAB1B_HUMAN	57.2	11	0.9727	0.995
1021	Tyrosine-protein kinase BTK OS=Homo sapiens GN=BTK PE=1 SV=3	sp|Q06187|BTK_HUMAN	7.9	3	0.8872	0.8566
1022	NHP2-like protein 1 OS=Homo sapiens GN=NHP2L1	sp|P55769|NH2L1_HUMAN	28.1	3	0.9204	0.7501
1023	Golgin subfamily A member 3 OS=Homo sapiens GN=GOLGA3 PE=1 SV=2	sp|Q08378|GOGA3_HUMAN	7.9	4	1.7219	0.4864
1024	Sortilin-related receptor OS=Homo sapiens GN=SORL1 PE=1	sp|Q92673|SORL_HUMAN	3.7	3	1.2589	0.7001
1025	Translocation protein SEC63 homolog OS=Homo sapiens GN=SEC63 PE=1	sp|Q9UGP8|SEC63_HUMAN	12.9	6	1.1588	0.4502
1026	ATP synthase subunit gamma, mitochondrial OS=Homo sapiens GN=ATP5C1 PE=1 SV=1	sp|P36542|ATPG_HUMAN	22.5	6	1.0186	0.9815
1027	Mitochondrial pyruvate carrier 2 OS=Homo sapiens GN=MPC2 PE=1 SV=1	sp|O95563|MPC2_HUMAN	33.9	4	1.0186	0.9039
1028	Caspase-3 OS=Homo sapiens GN=CASP3 PE=1 SV=2	sp|P42574|CASP3_HUMAN	18.4	5	0.9036	0.7352
1029	Ubiquitin-like modifier-activating enzyme 6 OS=Homo sapiens GN=UBA6 PE=1 SV=1	sp|A0AVT1|UBA6_HUMAN	9.6	7	1.0568	0.9002
1030	RNA 3'-terminal phosphate cyclase-like protein OS=Homo sapiens GN=RCL1 PE=1 SV=3	sp|Q9Y2P8|RCL1_HUMAN	7.5	3	0.955	0.9484
1031	Ras-related protein Rab-18 OS=Homo sapiens GN=RAB18 PE=1	sp|Q9NP72|RAB18_HUMAN	26.7	3	1.0965	0.8141
1032	H(+)/Cl(-) exchange transporter 3 OS=Homo sapiens GN=CLCN3 PE=1	sp|P51790|CLCN3_HUMAN	9.3	4	1.0568	0.6503
1033	Cytoskeleton-associated protein 5 OS=Homo sapiens GN=CKAP5 PE=1 SV=3	sp|Q14008|CKAP5_HUMAN	6.5	4	0.912	0.3225
1034	U3 small nucleolar RNA-associated protein 18 homolog OS=Homo sapiens GN=UTP18 PE=1 SV=3	sp|Q9Y5J1|UTP18_HUMAN	15.3	3	0.9204	0.5955
1035	Guanine nucleotide exchange factor VAV3 OS=Homo sapiens GN=VAV3 PE=1 SV=1	sp|Q9UKW4|VAV3_HUMAN	14.3	3	1.4322	0.3753
1036	Ubiquitin carboxyl-terminal hydrolase 5 OS=Homo sapiens GN=USP5 PE=1 SV=2	sp|P45974|UBP5_HUMAN	6.2	5	1.0965	0.8284

1037	Phosphoenolpyruvate carboxykinase [GTP], mitochondrial OS=Homo sapiens GN=PCK2 PE=1 SV=3	sp|Q16822|PCKGM_HUMAN	16.1	5	1.6596	0.4706
1038	Probable ATP-dependent RNA helicase DDX41 OS=Homo sapiens GN=DDX41 PE=1 SV=2	sp|Q9UJV9|DDX41_HUMAN	8.2	3	0.8551	0.4062
1039	Arf-GAP with Rho-GAP domain, ANK repeat and PH domain-containing protein 1 OS=Homo sapiens GN=ARAP1 PE=1	sp|Q96P48|ARAP1_HUMAN	10.3	8	1.3183	0.7518
1040	E3 UFM1-protein ligase 1 OS=Homo sapiens GN=UFL1 PE=1 SV=2	sp|O94874|UFL1_HUMAN	5.2	3	1.1695	0.6592
1041	Methyltransferase-like protein 13 OS=Homo sapiens GN=METTL13 PE=1 SV=1	sp|Q8N6R0|MET13_HUMAN	9.7	5	0.4831	0.5911
1042	Symplekin OS=Homo sapiens GN=SYMPK PE=1 SV=2	sp|Q92797|SYMPK_HUMAN	9.7	5	0.5152	0.438
1043	Ral GTPase-activating protein subunit beta OS=Homo sapiens GN=RALGAPB PE=1 SV=1	sp|Q86X10|RLGPB_HUMAN	4.2	3	1.3804	0.7163
1044	Myb-binding protein 1A OS=Homo sapiens GN=MYBBP1A PE=1 SV=2	sp|Q9BQG0|MBB1A_HUMAN	10.5	12	0.9638	0.9388
1045	Fatty acyl-CoA reductase 1 OS=Homo sapiens GN=FAR1 PE=1 SV=1	sp|Q8WVX9|FACR1_HUMAN	12.8	4	1.0666	0.8581
1046	Formin-binding protein 1-like OS=Homo sapiens GN=FNBP1L PE=1 SV=3	sp|Q5T0N5|FBP1L_HUMAN	9.6	3	1.2246	0.6833
1047	Unconventional myosin-XVIIIa OS=Homo sapiens GN=MYO18A PE=1 SV=3	sp|Q92614|MY18A_HUMAN	7.7	4	1.406	0.7434
1048	Charged multivesicular body protein 7 OS=Homo sapiens GN=CHMP7 PE=1 SV=1	sp|Q8WUX9|CHMP7_HUMAN	23.4	17	0.9204	0.9146
1049	Tropomyosin alpha-4 chain OS=Homo sapiens GN=TPM4 PE=1	sp|P67936|TPM4_HUMAN	43.6	8	1.1695	0.5053
1050	26S proteasome non-ATPase regulatory subunit 4 OS=Homo sapiens GN=PSMD4 PE=1	sp|P55036|PSMD4_HUMAN	34.2	4	1.1066	0.8229
1051	40S ribosomal protein S15 OS=Homo sapiens GN=RPS15 PE=1 SV=2	sp|P62841|RS15_HUMAN	55.9	5	0.9376	0.7554

1052	Alcohol dehydrogenase [NADP(+)] OS=Homo sapiens GN=AKR1A1 PE=1 SV=3	sp|P14550|AK1A1_HUMAN	24.3	9	1.2942	0.6146
1053	Pumilio homolog 1 OS=Homo sapiens GN=PUM1 PE=1 SV=3	sp|Q14671|PUM1_HUMAN	8.6	4	0.8872	0.8695
1054	Serine palmitoyltransferase 2 OS=Homo sapiens GN=SPTLC2 PE=1	sp|O15270|SPTC2_HUMAN	10.1	3	1.0093	0.8968
1055	Syntaxin-binding protein 1 OS=Homo sapiens GN=STXBP1 PE=1 SV=1	sp|P61764|STXB1_HUMAN	8.4	3	0.9727	0.9635
1056	15 kDa selenoprotein OS=Homo sapiens GN=SEP15 PE=1	sp|O60613|SEP15_HUMAN	25.9	3	1.0093	0.9754
1057	Serine/threonine-protein phosphatase 6 regulatory subunit 3 OS=Homo sapiens GN=PPP6R3 PE=1	sp|Q5H9R7|PP6R3_HUMAN	10.1	4	0.8166	0.5013
1058	Protein LYRIC OS=Homo sapiens GN=MTDH PE=1 SV=2	sp|Q86UE4|LYRIC_HUMAN	19.4	4	1.406	0.5251
1059	Exosome component 10 OS=Homo sapiens GN=EXOSC10 PE=1 SV=2	sp|Q01780|EXOSX_HUMAN	8.4	6	0.871	0.7939
1060	Rho guanine nucleotide exchange factor 2 OS=Homo sapiens GN=ARHGEF2 PE=1 SV=4	sp|Q92974|ARHG2_HUMAN	13.2	6	1	0.9737
1061	Exosome complex component MTR3 OS=Homo sapiens GN=EXOSC6 PE=1 SV=1	sp|Q5RKV6|EXOS6_HUMAN	29	3	0.7447	0.7931
1062	Phosphomevalonate kinase OS=Homo sapiens GN=PMVK PE=1	sp|Q15126|PMVK_HUMAN	28.1	3	1.2474	0.6313
1063	Tyrosine-protein phosphatase non-receptor type 23 OS=Homo sapiens GN=PTPN23 PE=1 SV=1	sp|Q9H3S7|PTN23_HUMAN	6.6	5	1.1695	0.4096
1064	Unconventional myosin-Id OS=Homo sapiens GN=MYO1D PE=1 SV=2	sp|O94832|MYO1D_HUMAN	13.6	5	1.0375	0.6765
1065	Digestive organ expansion factor homolog OS=Homo sapiens GN=DIEXF PE=1	sp|Q68CQ4|DIEXF_HUMAN	10.1	4	0.8241	0.9005
1066	Nardilysin OS=Homo sapiens GN=NRD1 PE=1 SV=2	sp|O43847|NRDC_HUMAN	10.1	3	0.9908	0.9932
1067	Protein SDA1 homolog OS=Homo sapiens GN=SDAD1 PE=1	sp|Q9NVU7|SDA1_HUMAN	10.6	4	0.912	0.9872
1068	Splicing factor 3B subunit 4 OS=Homo sapiens GN=SF3B4 PE=1	sp|Q15427|SF3B4_HUMAN	14.4	3	0.7656	0.4312

1069	Integrator complex subunit 4 OS=Homo sapiens GN=INTS4 PE=1	sp|Q96HW7|INT4_HUMAN	5.1	5	0.6252	0.477
1070	Disintegrin and metalloproteinase domain-containing protein 10 OS=Homo sapiens GN=ADAM10 PE=1 SV=1	sp|O14672|ADA10_HUMAN	7	3	0.7112	0.2094
1071	U6 snRNA-associated Sm-like protein LSm2 OS=Homo sapiens GN=LSM2 PE=1 SV=1	sp|Q9Y333|LSM2_HUMAN	46.3	3	0.8017	0.6311
1072	Transcription elongation factor B polypeptide 1 OS=Homo sapiens GN=TCEB1 PE=1 SV=1	sp|Q15369|ELOC_HUMAN	33	3	0.955	0.8657
1073	40S ribosomal protein S24 OS=Homo sapiens GN=RPS24 PE=1 SV=1	sp|P62847|RS24_HUMAN	30.8	5	0.9204	0.747
1074	WD repeat-containing protein 26 OS=Homo sapiens GN=WDR26 PE=1	sp|Q9H7D7|WDR26_HUMAN	9.1	3	1.0965	0.5031
1075	Heterogeneous nuclear ribonucleoprotein H2 OS=Homo sapiens GN=HNRNPH2 PE=1 SV=1	sp|P55795|HNRH2_HUMAN	23.2	9	1.0186	0.9175
1076	Eukaryotic translation initiation factor 2 subunit 2 OS=Homo sapiens GN=EIF2S2 PE=1	sp|P20042|IF2B_HUMAN	24.3	11	1.0965	0.7758
1077	Influenza virus NS1A-binding protein OS=Homo sapiens GN=IVNS1ABP PE=1 SV=3	sp|Q9Y6Y0|NS1BP_HUMAN	11.7	6	0.6668	0.3853
1078	Probable aminopeptidase NPEPL1 OS=Homo sapiens GN=NPEPL1 PE=1 SV=3	sp|Q8NDH3|PEPL1_HUMAN	17.2	7	0.9036	0.7741
1079	Enhancer of mRNA-decapping protein 3 OS=Homo sapiens GN=EDC3 PE=1	sp|Q96F86|EDC3_HUMAN	16.3	3	0.955	0.8565
1080	Ubiquitin carboxyl-terminal hydrolase 34 OS=Homo sapiens GN=USP34 PE=1	sp|Q70CQ2|UBP34_HUMAN	3.6	4	0.8318	0.4123
1081	WD repeat- and FYVE domain-containing protein 4 OS=Homo sapiens GN=WDFY4 PE=1	sp|Q6ZS81|WDFY4_HUMAN	6.6	5	1.0864	0.8482
1082	Pyridoxal-dependent decarboxylase domain-containing protein 1 OS=Homo sapiens GN=PDXDC1 PE=1 SV=2	sp|Q6P996|PDXD1_HUMAN	8.8	3	1.0093	0.9928

1083	UBX domain-containing protein 1 OS=Homo sapiens GN=UBXN1 PE=1	sp|Q04323|UBXN1_HUMAN	29.6	6	1.0765	0.7041
1084	Elongation factor 1-beta OS=Homo sapiens GN=EEF1B2 PE=1 SV=3	sp|P24534|EF1B_HUMAN	28.4	5	0.9908	0.9965
1085	Caspase recruitment domain-containing protein 11 OS=Homo sapiens GN=CARD11	sp|Q9BXL7|CAR11_HUMAN	10.5	5	1.0666	0.7727
1086	Ribosome-recycling factor, mitochondrial OS=Homo sapiens GN=MRRF PE=1 SV=1	sp|Q96E11|RRFM_HUMAN	23.3	4	1.1803	0.7156
1087	TFIIH basal transcription factor complex helicase XPB subunit OS=Homo sapiens GN=ERCC3 PE=1 SV=1	sp|P19447|ERCC3_HUMAN	9.7	4	0.871	0.7728
1088	E3 SUMO-protein ligase RanBP2 OS=Homo sapiens GN=RANBP2 PE=1 SV=2	sp|P49792|RBP2_HUMAN	5.2	4	1	0.9804
1089	60S ribosomal protein L17 OS=Homo sapiens GN=RPL17 PE=1 SV=3	sp|P18621|RL17_HUMAN	47.8	3	0.9036	0.6407
1090	Flap endonuclease 1 OS=Homo sapiens GN=FEN1 PE=1 SV=1	sp|P39748|FEN1_HUMAN	26.3	6	0.8166	0.7775
1091	Lamina-associated polypeptide 2, isoforms beta/gamma OS=Homo sapiens GN=TMPO PE=1 SV=2	sp|P42167|LAP2B_HUMAN	17.4	5	0.9908	0.956
1092	MICOS complex subunit MIC13 OS=Homo sapiens GN=MIC13 PE=1 SV=1	sp|Q5XKP0|MIC13_HUMAN	40.7	4	1.028	0.82
1093	Programmed cell death protein 5 OS=Homo sapiens GN=PDCD5 PE=1 SV=3	sp|O14737|PDCD5_HUMAN	36.8	3	1	0.9924
1094	Poly(ADP-ribose) glycohydrolase OS=Homo sapiens GN=PARG PE=1 SV=1	sp|Q86W56|PARG_HUMAN	5.5	3	0.879	0.6204
1095	SH3 domain-containing kinase-binding protein 1 OS=Homo sapiens GN=SH3KBP1 PE=1 SV=2	sp|Q96B97|SH3K1_HUMAN	5	3	1.406	0.8504
1096	DNA topoisomerase 2-alpha OS=Homo sapiens	sp|P11388|TOP2A_HUMAN	6.9	6	0.4325	0.3294
1097	General transcription factor 3C polypeptide 3 OS=Homo sapiens GN=GTF3C3 PE=1 SV=1	sp|Q9Y5Q9|TF3C3_HUMAN	9	4	1.0471	0.8596

1098	Poly(A) polymerase alpha OS=Homo sapiens GN=PAPOLA PE=1 SV=4	sp|P51003|PAPOA_HUMAN	10.7	3	0.8472	0.6949
1099	E3 ubiquitin-protein ligase HECTD3 OS=Homo sapiens GN=HECTD3	sp|Q5T447|HECD3_HUMAN	11.3	4	1.028	0.8598
1100	Regulator of chromosome condensation OS=Homo sapiens GN=RCC1 PE=1 SV=1	sp|P18754|RCC1_HUMAN	19.7	5	1.028	0.8316
1101	SNW domain-containing protein 1 OS=Homo sapiens GN=SNW1 PE=1 SV=1	sp|Q13573|SNW1_HUMAN	16	8	0.5346	0.3031
1102	40S ribosomal protein S11 OS=Homo sapiens GN=RPS11 PE=1 SV=3	sp|P62280|RS11_HUMAN	27.9	4	0.9638	0.8889
1103	WD repeat-containing protein 75 OS=Homo sapiens GN=WDR75 PE=1	sp|Q8IWA0|WDR75_HUMAN	6.5	3	1.0471	0.8983
1104	E2/E3 hybrid ubiquitin-protein ligase UBE2O OS=Homo sapiens GN=UBE2O PE=1 SV=3	sp|Q9C0C9|UBE2O_HUMAN	6	3	1.028	0.9575
1105	Stathmin OS=Homo sapiens GN=STMN1 PE=1	sp|P16949|STMN1_HUMAN	20.8	3		
1106	Aminoacyl tRNA synthase complex-interacting multifunctional protein 1 OS=Homo sapiens GN=AIMP1 PE=1	sp|Q12904|AIMP1_HUMAN	25.3	5	1.0666	0.8653
1107	Uroporphyrinogen decarboxylase OS=Homo sapiens GN=UROD PE=1 SV=2	sp|P06132|DCUP_HUMAN	16.9	4	0.9727	0.8625
1108	Casein kinase II subunit alpha OS=Homo sapiens GN=CSNK2A1 PE=1 SV=1	sp|P68400|CSK21_HUMAN	14.3	5	0.9817	0.9989
1109	Isocitrate dehydrogenase [NAD] subunit alpha, mitochondrial OS=Homo sapiens GN=IDH3A PE=1	sp|P50213|IDH3A_HUMAN	14.2	5	1	0.9934
1110	Transmembrane emp24 domain-containing protein 10 OS=Homo sapiens GN=TMED10	sp|P49755|TMEDA_HUMAN	21.9	3	0.9727	0.9974
1111	DNA ligase 3 OS=Homo sapiens GN=LIG3 PE=1 SV=2	sp|P49916|DNLI3_HUMAN	7.5	3	1	0.894
1112	ATP-dependent RNA helicase DDX54 OS=Homo sapiens GN=DDX54 PE=1	sp|Q8TDD1|DDX54_HUMAN	9.3	4	0.8954	0.5317

1113	Signal transducer and activator of transcription 3 OS=Homo sapiens GN=STAT3 PE=1 SV=2	sp|P40763|STAT3_HUMAN	11	3	0.4325	0.2213
1114	Aminopeptidase B OS=Homo sapiens GN=RNPEP PE=1 SV=2	sp|Q9H4A4|AMPB_HUMAN	12.9	3	1.0864	0.531
1115	Serine/arginine-rich splicing factor 9 OS=Homo sapiens GN=SRSF9 PE=1 SV=1	sp|Q13242|SRSF9_HUMAN	16.3	2	0.8872	0.6811
1116	Nascent polypeptide-associated complex subunit alpha, muscle-specific form OS=Homo sapiens GN=NACA PE=1 SV=1	sp|E9PAV3|NACAM_HUMAN	9.9	16	0.9817	0.9503
1117	Thioredoxin-dependent peroxide reductase, mitochondrial OS=Homo sapiens GN=PRDX3 PE=1 SV=3	sp|P30048|PRDX3_HUMAN	18.4	4		
1118	AFG3-like protein 2 OS=Homo sapiens GN=AFG3L2	sp|Q9Y4W6|AFG32_HUMAN	7	2	1.0666	0.5088
1119	UDP-glucose 6-dehydrogenase OS=Homo sapiens GN=UGDH PE=1 SV=1	sp|O60701|UGDH_HUMAN	15	4	0.912	0.9949
1120	Biliverdin reductase A OS=Homo sapiens GN=BLVRA PE=1 SV=2	sp|P53004|BIEA_HUMAN	17.9	3	1.1169	0.5979
1121	Cytosolic purine 5'-nucleotidase OS=Homo sapiens GN=NT5C2 PE=1 SV=1	sp|P49902|5NTC_HUMAN	10.2	2	0.955	0.6173
1122	Inorganic pyrophosphatase OS=Homo sapiens GN=PPA1 PE=1 SV=2	sp|Q15181|IPYR_HUMAN	20.1	3	0.9727	0.9838
1123	Rab GTPase-activating protein 1 OS=Homo sapiens GN=RABGAP1 PE=1 SV=3	sp|Q9Y3P9|RBGP1_HUMAN	6.8	3	1.1482	0.7663
1124	Protein bicaudal D homolog 2 OS=Homo sapiens GN=BICD2 PE=1 SV=1	sp|Q8TD16|BICD2_HUMAN	10.7	3	0.9462	0.7668
1125	Ran GTPase-activating protein 1 OS=Homo sapiens GN=RANGAP1 PE=1 SV=1	sp|P46060|RAGP1_HUMAN	15.3	5	0.8872	0.711
1126	Deoxyribose-phosphate aldolase OS=Homo sapiens GN=DERA PE=1 SV=2	sp|Q9Y315|DEOC_HUMAN	20.8	3	1.0568	0.9011
1127	Proteasomal ubiquitin receptor ADRM1 OS=Homo sapiens GN=ADRM1 PE=1 SV=2	sp|Q16186|ADRM1_HUMAN	17	3	0.9462	0.846
1128	RNA-binding protein 14 OS=Homo sapiens GN=RBM14 PE=1	sp|Q96PK6|RBM14_HUMAN	11.7	3	0.9817	0.9313

1129	Endoribonuclease Dicer OS=Homo sapiens GN=DICER1 PE=1 SV=3	sp|Q9UPY3|DICER_HUMAN	5.7	6	0.9638	0.8924
1130	Protein SEC13 homolog OS=Homo sapiens GN=SEC13 PE=1	sp|P55735|SEC13_HUMAN	17.4	3	0.9817	0.9509
1131	Histone H1.4 OS=Homo sapiens GN=HIST1H1E PE=1 SV=2	sp|P10412|H14_HUMAN	29.7	8	0.5649	0.1658
1132	RNA-binding protein 27 OS=Homo sapiens GN=RBM27 PE=1	sp|Q9P2N5|RBM27_HUMAN	5.4	2	0.929	0.5586
1133	60S ribosomal protein L23a OS=Homo sapiens GN=RPL23A PE=1 SV=1	sp|P62750|RL23A_HUMAN	24.4	3	0.912	0.686
1134	Far upstream element-binding protein 1 OS=Homo sapiens GN=FUBP1 PE=1	sp|Q96AE4|FUBP1_HUMAN	6.8	4	0.9817	0.8999
1135	Programmed cell death 6-interacting protein OS=Homo sapiens GN=PDCD6IP PE=1 SV=1	sp|Q8WUM4|PDC6I_HUMAN	10.9	5	1.556	0.7646
1136	Keratin, type I cytoskeletal 18 OS=Homo sapiens GN=KRT18 PE=1 SV=2	sp|P05783|K1C18_HUMAN	10.2	2	0.863	0.8733
1137	Endoplasmic reticulum aminopeptidase 1 OS=Homo sapiens GN=ERAP1 PE=1 SV=3	sp|Q9NZ08|ERAP1_HUMAN	5.8	4	0.912	0.8604
1138	WD repeat and FYVE domain-containing protein 3 OS=Homo sapiens GN=WDFY3 PE=1	sp|Q8IZQ1|WDFY3_HUMAN	3.9	5	1.0568	0.5711
1139	Ras-related protein Rap-2c OS=Homo sapiens GN=RAP2C PE=1	sp|Q9Y3L5|RAP2C_HUMAN	29	2	1.1272	0.5369
1140	WASH complex subunit 7 OS=Homo sapiens GN=KIAA1033 PE=1 SV=2	sp|Q2M389|WASH7_HUMAN	5.6	3	1.2359	0.668
1141	Trifunctional enzyme subunit beta, mitochondrial OS=Homo sapiens GN=HADHB PE=1 SV=3	sp|P55084|ECHB_HUMAN	14.1	5	1.0093	0.9393
1142	Solute carrier family 12 member 6 OS=Homo sapiens GN=SLC12A6	sp|Q9UHW9|S12A6_HUMAN	7	3	1.2134	0.5041
1143	Pescadillo homolog OS=Homo sapiens GN=PES1 PE=1 SV=1	sp|O00541|PESC_HUMAN	9.9	3	0.8395	0.6328
1144	Fibronectin type III domain-containing protein 3B OS=Homo sapiens GN=FNDC3B PE=1 SV=2	sp|Q53EP0|FND3B_HUMAN	5.8	3	2.3988	0.2004

1145	Aminoacyl tRNA synthase complex-interacting multifunctional protein 2 OS=Homo sapiens GN=AIMP2 PE=1	sp|Q13155|AIMP2_HUMAN	13.1	3	0.9376	0.9329
1146	tRNA (uracil-5-)-methyltransferase homolog A OS=Homo sapiens GN=TRMT2A PE=1 SV=2	sp|Q8IZ69|TRM2A_HUMAN	9.3	3	0.8872	0.7967
1147	Zinc phosphodiesterase ELAC protein 2 OS=Homo sapiens GN=ELAC2 PE=1 SV=2	sp|Q9BQ52|RNZ2_HUMAN	8.8	5	0.9462	0.9104
1148	PHD finger-like domain-containing protein 5A OS=Homo sapiens GN=PHF5A PE=1 SV=1	sp|Q7RTV0|PHF5A_HUMAN	33.6	2	0.6982	0.4489
1149	Heterochromatin protein 1-binding protein 3 OS=Homo sapiens GN=HP1BP3 PE=1 SV=1	sp|Q5SSJ5|HP1B3_HUMAN	9.2	3	1.0666	0.874
1150	Golgi to ER traffic protein 4 homolog OS=Homo sapiens GN=GET4 PE=1 SV=1	sp|Q7L5D6|GET4_HUMAN	14.4	2	0.8395	0.8491
1151	ER membrane protein complex subunit 2 OS=Homo sapiens GN=EMC2 PE=1	sp|Q15006|EMC2_HUMAN	13.8	3	1.028	0.8661
1152	Glia maturation factor beta OS=Homo sapiens GN=GMFB PE=1 SV=2	sp|P60983|GMFB_HUMAN	38.7	8	2.3121	0.5297
1153	Isoleucine--tRNA ligase, mitochondrial OS=Homo sapiens GN=IARS2 PE=1	sp|Q9NSE4|SYIM_HUMAN	9.3	5	1.0765	0.8531
1154	Serine/threonine-protein phosphatase 2A 56 kDa regulatory subunit gamma isoform OS=Homo sapiens GN=PPP2R5C PE=1 SV=3	sp|Q13362|2A5G_HUMAN	12.2	3	0.929	0.8263
1155	Tubulin gamma-1 chain OS=Homo sapiens GN=TUBG1 PE=1	sp|P23258|TBG1_HUMAN	23.1	3	0.3404	0.2536
1156	Legumain OS=Homo sapiens GN=LGMN PE=1 SV=1	sp|Q99538|LGMN_HUMAN	16.6	4	1.4859	0.4825
1157	RNA-binding protein 6 OS=Homo sapiens GN=RBM6 PE=1	sp|P78332|RBM6_HUMAN	3.8	3		
1158	E3 ubiquitin-protein ligase CHIP OS=Homo sapiens GN=STUB1 PE=1 SV=2	sp|Q9UNE7|CHIP_HUMAN	19.1	3	1.0186	0.8646
1159	Dr1-associated corepressor OS=Homo sapiens GN=DRAP1 PE=1 SV=3	sp|Q14919|NC2A_HUMAN	21	3	1	0.9865
1160	Kinectin OS=Homo sapiens GN=KTN1 PE=1	sp|Q86UP2|KTN1_HUMAN	8	4	1.0666	0.7976

1161	Nuclear pore glycoprotein p62 OS=Homo sapiens GN=NUP62 PE=1 SV=3	sp|P37198|NUP62_HUMAN	11.7	3	0.9204	0.876
1162	Protein transport protein Sec24A OS=Homo sapiens GN=SEC24A PE=1 SV=2	sp|O95486|SC24A_HUMAN	6.2	4	1.0093	0.8638
1163	EF-hand domain-containing protein D2 OS=Homo sapiens GN=EFHD2 PE=1	sp|Q96C19|EFHD2_HUMAN	23.8	4	1.9588	0.4365
1164	Rho GTPase-activating protein 17 OS=Homo sapiens GN=ARHGAP17	sp|Q68EM7|RHG17_HUMAN	13.9	6	1.2589	0.7225
1165	Protein virilizer homolog OS=Homo sapiens GN=KIAA1429 PE=1 SV=2	sp|Q69YN4|VIR_HUMAN	4	4	0.955	0.9872
1166	Ras-related GTP-binding protein C OS=Homo sapiens GN=RRAGC PE=1 SV=1	sp|Q9HB90|RRAGC_HUMAN	21.1	4	1.7378	0.3157
1167	GMP reductase 2 OS=Homo sapiens GN=GMPR2 PE=1 SV=1	sp|Q9P2T1|GMPR2_HUMAN	10.1	3	1.1376	0.7889
1168	Aladin OS=Homo sapiens GN=AAAS PE=1 SV=1	sp|Q9NRG9|AAAS_HUMAN	17.6	5	0.8872	0.8281
1169	Protein OS-9 OS=Homo sapiens GN=OS9 PE=1 SV=1	sp|Q13438|OS9_HUMAN	10.9	3	1.0666	0.7898
1170	Vacuolar protein sorting-associated protein 28 homolog OS=Homo sapiens GN=VPS28 PE=1 SV=1	sp|Q9UK41|VPS28_HUMAN	24.4	3	1.1803	0.5952
1171	Transcriptional regulator ATRX OS=Homo sapiens GN=ATRX PE=1 SV=5	sp|P46100|ATRX_HUMAN	3.6	3	0.929	0.7887
1172	Neurochondrin OS=Homo sapiens GN=NCDN PE=1 SV=1	sp|Q9UBB6|NCDN_HUMAN	9.9	3	0.5702	0.4187
1173	Calnexin OS=Homo sapiens GN=CANX PE=1	sp|P27824|CALX_HUMAN	15.5	5	1.2246	0.5532
1174	Protein kinase C-binding protein 1 OS=Homo sapiens GN=ZMYND8 PE=1 SV=2	sp|Q9ULU4|PKCB1_HUMAN	7.3	2	0.929	0.5641
1175	DIS3-like exonuclease 2 OS=Homo sapiens GN=DIS3L2 PE=1 SV=4	sp|Q8IYB7|DI3L2_HUMAN	7	3	1.0375	0.9553
1176	DENN domain-containing protein 4B OS=Homo sapiens GN=DENND4B PE=1 SV=4	sp|O75064|DEN4B_HUMAN	6.2	5	1.028	0.9471
1177	Tensin-3 OS=Homo sapiens GN=TNS3 PE=1	sp|Q68CZ2|TENS3_HUMAN	7.6	5	0.8551	0.7651
1178	Transformer-2 protein homolog alpha OS=Homo sapiens GN=TRA2A PE=1	sp|Q13595|TRA2A_HUMAN	29.8	4	1.0765	0.6004

1179	Cell division cycle 5-like protein OS=Homo sapiens GN=CDC5L PE=1 SV=2	sp|Q99459|CDC5L_HUMAN	13	6	0.631	0.5511
1180	Neuroblast differentiation-associated protein AHNAK OS=Homo sapiens GN=AHNAK PE=1 SV=2	sp|Q09666|AHNK_HUMAN	9.4	5	1.5849	0.329
1181	U6 snRNA-associated Sm-like protein LSm1 OS=Homo sapiens GN=LSM1 PE=1 SV=1	sp|O15116|LSM1_HUMAN	29.3	2	0.6081	0.5112
1182	Protein PBDC1 OS=Homo sapiens GN=PBDC1 PE=1 SV=1	sp|Q9BVG4|PBDC1_HUMAN	22.3	4	0.9727	0.9382
1183	Fragile X mental retardation protein 1 OS=Homo sapiens GN=FMR1 PE=1 SV=1	sp|Q06787|FMR1_HUMAN	10.4	2	0.9817	0.9451
1184	Vacuolar protein sorting-associated protein 18 homolog OS=Homo sapiens GN=VPS18 PE=1 SV=2	sp|Q9P253|VPS18_HUMAN	7	4	1.2246	0.6809
1185	Huntingtin OS=Homo sapiens GN=HTT PE=1 SV=2	sp|P42858|HD_HUMAN	3.5	3	1.0471	0.9318
1186	WD repeat-containing protein 82 OS=Homo sapiens GN=WDR82 PE=1	sp|Q6UXN9|WDR82_HUMAN	15	3	1.0471	0.8931
1187	DNA-directed RNA polymerases I, II, and III subunit RPABC3 OS=Homo sapiens GN=POLR2H PE=1 SV=4	sp|P52434|RPAB3_HUMAN	34.7	3	1.0864	0.889
1188	Aspartate aminotransferase, cytoplasmic OS=Homo sapiens GN=GOT1 PE=1 SV=3	sp|P17174|AATC_HUMAN	18.9	5	1.2246	0.6982
1189	Stromal cell-derived factor 2-like protein 1 OS=Homo sapiens GN=SDF2L1 PE=1	sp|Q9HCN8|SDF2L_HUMAN	30.3	5	0.9204	0.8783
1190	Protein SON OS=Homo sapiens GN=SON PE=1 SV=4	sp|P18583|SON_HUMAN	4.3	3	0.9376	0.873
1191	Guanine nucleotide-binding protein G(I)/G(S)/G(T) subunit beta-2 OS=Homo sapiens GN=GNB2 PE=1	sp|P62879|GBB2_HUMAN	23.5	8	1.2134	0.6903
1192	Glutamate-rich WD repeat-containing protein 1 OS=Homo sapiens GN=GRWD1 PE=1 SV=1	sp|Q9BQ67|GRWD1_HUMAN	20.9	11	0.912	0.7767
1193	Protein MAK16 homolog OS=Homo sapiens GN=MAK16 PE=1 SV=2	sp|Q9BXY0|MAK16_HUMAN	19.7	3	1.0666	0.7484

1194	Multidrug resistance-associated protein 4 OS=Homo sapiens GN=ABCC4 PE=1 SV=3	sp|O15439|MRP4_HUMAN	7	4	1.1169	0.7865
1195	Spermine synthase OS=Homo sapiens GN=SMS PE=1 SV=2	sp|P52788|SPSY_HUMAN	16.7	3	0.9908	0.9118
1196	High mobility group protein B2 OS=Homo sapiens GN=HMGB2 PE=1 SV=2	sp|P26583|HMGB2_HUMAN	29.2	8	0.7798	0.5728
1197	Structural maintenance of chromosomes flexible hinge domain-containing protein 1 OS=Homo sapiens GN=SMCHD1 PE=1 SV=2	sp|A6NHR9|SMHD1_HUMAN	5.9	3	1	0.9737
1198	AP-3 complex subunit delta-1 OS=Homo sapiens GN=AP3D1 PE=1 SV=1	sp|O14617|AP3D1_HUMAN	8.2	3	0.9727	0.633
1199	NudC domain-containing protein 3 OS=Homo sapiens GN=NUDCD3	sp|Q8IVD9|NUDC3_HUMAN	14.1	4	0.5754	0.3529
1200	Procollagen galactosyltransferase 1 OS=Homo sapiens GN=COLGALT1 PE=1 SV=1	sp|Q8NBJ5|GT251_HUMAN	10.3	4	1.0471	0.9158
1201	Cytoplasmic dynein 1 intermediate chain 2 OS=Homo sapiens GN=DYNC1I2	sp|Q13409|DC1I2_HUMAN	23.2	8	1.0965	0.7182
1202	Leucine-rich repeat and calponin homology domain-containing protein 4 OS=Homo sapiens GN=LRCH4 PE=1	sp|O75427|LRCH4_HUMAN	10.1	3	1.0186	0.9696
1203	GDP-mannose 4,6 dehydratase OS=Homo sapiens GN=GMDS PE=1 SV=1	sp|O60547|GMDS_HUMAN	14.3	3	1.1376	0.5003
1204	Band 4.1-like protein 2 OS=Homo sapiens GN=EPB41L2	sp|O43491|E41L2_HUMAN	9.5	6	1.028	0.8932
1205	60S ribosomal protein L37a OS=Homo sapiens GN=RPL37A PE=1 SV=2	sp|P61513|RL37A_HUMAN	39.1	5	0.9817	0.8989
1206	THO complex subunit 1 OS=Homo sapiens GN=THOC1 PE=1	sp|Q96FV9|THOC1_HUMAN	9.9	6	0.9817	0.9923
1207	Unconventional myosin-XIX OS=Homo sapiens GN=MYO19 PE=2 SV=2	sp|Q96H55|MYO19_HUMAN	5.1	2	0.929	0.8307
1208	Small nuclear ribonucleoprotein-associated proteins B and B' OS=Homo sapiens GN=SNRPB PE=1	sp|P14678|RSMB_HUMAN	21.3	3	0.9462	0.8553

1209	N-acetylgalactosaminyltransferase 7 OS=Homo sapiens GN=GALNT7 PE=1 SV=1	sp|Q86SF2|GALT7_HUMAN	7.8	3	0.9908	0.9758
1210	mRNA cap guanine-N7 methyltransferase OS=Homo sapiens GN=RNMT PE=1 SV=1	sp|O43148|MCES_HUMAN	8.6	2	1.0864	0.8473
1211	C-Jun-amino-terminal kinase-interacting protein 4 OS=Homo sapiens GN=SPAG9 PE=1	sp|O60271|JIP4_HUMAN	6.9	6	1.803	0.4583
1212	3-hydroxyisobutyrate dehydrogenase, mitochondrial OS=Homo sapiens GN=HIBADH PE=1 SV=2	sp|P31937|3HIDH_HUMAN	14.9	4	0.9638	0.927
1213	Thioredoxin domain-containing protein 5 OS=Homo sapiens GN=TXNDC5 PE=1 SV=2	sp|Q8NBS9|TXND5_HUMAN	14.1	3	0.9908	0.9333
1214	Nucleoporin NUP53 OS=Homo sapiens GN=NUP35 PE=1	sp|Q8NFH5|NUP53_HUMAN	12.6	3	1.0093	0.957
1215	WW domain-binding protein 11 OS=Homo sapiens GN=WBP11 PE=1 SV=1	sp|Q9Y2W2|WBP11_HUMAN	10.3	3	0.7447	0.6621
1216	Protein MON2 homolog OS=Homo sapiens GN=MON2 PE=1	sp|Q7Z3U7|MON2_HUMAN	4.4	3	0.9817	0.9064
1217	Tudor and KH domain-containing protein OS=Homo sapiens GN=TDRKH PE=1 SV=2	sp|Q9Y2W6|TDRKH_HUMAN	12.5	7	0.8551	0.7471
1218	Ribosome biogenesis protein BOP1 OS=Homo sapiens GN=BOP1 PE=1 SV=2	sp|Q14137|BOP1_HUMAN	7.9	4	0.8318	0.6537
1219	Protein DEK OS=Homo sapiens GN=DEK PE=1 SV=1	sp|P35659|DEK_HUMAN	12.3	3	0.8551	0.5187
1220	NEDD8-activating enzyme E1 catalytic subunit OS=Homo sapiens GN=UBA3 PE=1	sp|Q8TBC4|UBA3_HUMAN	13.4	3	1.1066	0.9639
1221	Nucleolar pre-ribosomal-associated protein 1 OS=Homo sapiens GN=URB1 PE=1 SV=4	sp|O60287|NPA1P_HUMAN	3.7	4	1.1066	0.821
1222	Kinesin light chain 1 OS=Homo sapiens GN=KLC1 PE=1 SV=2	sp|Q07866|KLC1_HUMAN	12.9	3	1.0375	0.8804
1223	Nuclear migration protein nudC OS=Homo sapiens GN=NUDC PE=1 SV=1	sp|Q9Y266|NUDC_HUMAN	24.2	5	0.7727	0.6526
1224	Elongator complex protein 2 OS=Homo sapiens GN=ELP2 PE=1	sp|Q6IA86|ELP2_HUMAN	6.4	2	0.9908	0.925

1225	Peroxiredoxin-4 OS=Homo sapiens GN=PRDX4 PE=1 SV=1	sp|Q13162|PRDX4_HUMAN	20.7	3	1.1169	0.5872
1226	Epidermal growth factor receptor substrate 15 OS=Homo sapiens GN=EPS15 PE=1 SV=2	sp|P42566|EPS15_HUMAN	14.8	8	1.0965	0.5857
1227	GrpE protein homolog 1, mitochondrial OS=Homo sapiens GN=GRPEL1 PE=1 SV=2	sp|Q9HAV7|GRPE1_HUMAN	21.2	4	1.0965	0.8324
1228	ATPase family AAA domain-containing protein 3A OS=Homo sapiens GN=ATAD3A PE=1 SV=2	sp|Q9NVI7|ATD3A_HUMAN	9.1	3	0.863	0.7796
1229	Vacuolar protein sorting-associated protein 4B OS=Homo sapiens GN=VPS4B PE=1 SV=2	sp|O75351|VPS4B_HUMAN	21	5	0.9462	0.8525
1230	Syntaxin-12 OS=Homo sapiens GN=STX12 PE=1 SV=1	sp|Q86Y82|STX12_HUMAN	19.6	4	1.1588	0.7531
1231	Mitochondrial-processing peptidase subunit alpha OS=Homo sapiens GN=PMPCA PE=1	sp|Q10713|MPPA_HUMAN	9.3	4	0.929	0.7311
1232	Testin OS=Homo sapiens GN=TES PE=1 SV=1	sp|Q9UGI8|TES_HUMAN	8.6	3	0.9727	0.9351
1233	Dephospho-CoA kinase domain-containing protein OS=Homo sapiens GN=DCAKD PE=1 SV=1	sp|Q8WVC6|DCAKD_HUMAN	14.3	3	0.6546	0.574
1234	Ubiquitin carboxyl-terminal hydrolase isozyme L3 OS=Homo sapiens GN=UCHL3 PE=1 SV=1	sp|P15374|UCHL3_HUMAN	22.2	3	1.0765	0.853
1235	Cytospin-B OS=Homo sapiens GN=SPECC1 PE=1 SV=1	sp|Q5M775|CYTSB_HUMAN	6.3	4	0.7798	0.9324
1236	Nuclear cap-binding protein subunit 1 OS=Homo sapiens GN=NCBP1 PE=1	sp|Q09161|NCBP1_HUMAN	8.5	4		
1237	Synaptojanin-1 OS=Homo sapiens GN=SYNJ1 PE=1 SV=2	sp|O43426|SYNJ1_HUMAN	5.5	4	1.1695	0.7178
1238	BRCA2 and CDKN1A-interacting protein OS=Homo sapiens GN=BCCIP PE=1 SV=1	sp|Q9P287|BCCIP_HUMAN	18.8	3	0.8472	0.7623
1239	NudC domain-containing protein 2 OS=Homo sapiens GN=NUDCD2	sp|Q8WVJ2|NUDC2_HUMAN	26.1	4	0.9908	0.9781
1240	U3 small nucleolar ribonucleoprotein protein IMP3 OS=Homo sapiens GN=IMP3 PE=1 SV=1	sp|Q9NV31|IMP3_HUMAN	33.7	3	0.9462	0.9083

1241	Signal transducer and activator of transcription 6 OS=Homo sapiens GN=STAT6 PE=1 SV=1	sp|P42226|STAT6_HUMAN	10	5	0.929	0.3153
1242	Calcium/calmodulin-dependent protein kinase type II subunit delta OS=Homo sapiens GN=CAMK2D PE=1 SV=3	sp|Q13557|KCC2D_HUMAN	13.6	4	2.2699	0.26
1243	Gem-associated protein 4 OS=Homo sapiens GN=GEMIN4 PE=1 SV=2	sp|P57678|GEMI4_HUMAN	8.4	5	1	0.9823
1244	Acetolactate synthase-like protein OS=Homo sapiens GN=ILVBL PE=1	sp|A1L0T0|ILVBL_HUMAN	13.5	5		
1245	EH domain-binding protein 1-like protein 1 OS=Homo sapiens GN=EHBP1L1	sp|Q8N3D4|EH1L1_HUMAN	6.1	3	1.0375	0.9071
1246	MAP kinase-activated protein kinase 2 OS=Homo sapiens GN=MAPKAPK2 PE=1 SV=1	sp|P49137|MAPK2_HUMAN	13.3	3	0.5702	0.5132
1247	Coactosin-like protein OS=Homo sapiens GN=COTL1 PE=1	sp|Q14019|COTL1_HUMAN	43.7	5		
1248	Purine nucleoside phosphorylase OS=Homo sapiens GN=PNP PE=1 SV=2	sp|P00491|PNPH_HUMAN	12.8	4	1.1272	0.8244
1249	Prefoldin subunit 6 OS=Homo sapiens GN=PFDN6 PE=1 SV=1	sp|O15212|PFD6_HUMAN	23.3	2	0.9817	0.8596
1250	WD repeat-containing protein 91 OS=Homo sapiens GN=WDR91 PE=1	sp|A4D1P6|WDR91_HUMAN	9.8	2	2.0324	0.2655
1251	E3 ubiquitin-protein ligase RNF213 OS=Homo sapiens GN=RNF213 PE=1	sp|Q63HN8|RN213_HUMAN	2.5	4	1.1272	0.7173
1252	Hexokinase-3 OS=Homo sapiens GN=HK3 PE=1 SV=2	sp|P52790|HXK3_HUMAN	8.8	4	1.5996	0.4348
1253	Importin-4 OS=Homo sapiens GN=IPO4 PE=1 SV=2	sp|Q8TEX9|IPO4_HUMAN	12.5	5	1.0864	0.8506
1254	Protein diaphanous homolog 2 OS=Homo sapiens GN=DIAPH2 PE=1 SV=1	sp|O60879|DIAP2_HUMAN	7	3	1.3428	0.6308
1255	28S ribosomal protein S25, mitochondrial OS=Homo sapiens GN=MRPS25 PE=1 SV=1	sp|P82663|RT25_HUMAN	20.8	2	0.955	0.7285
1256	DnaJ homolog subfamily C member 7 OS=Homo sapiens GN=DNAJC7 PE=1 SV=2	sp|Q99615|DNJC7_HUMAN	13	2	0.7943	0.5268

1257	General transcription factor 3C polypeptide 2 OS=Homo sapiens GN=GTF3C2 PE=1 SV=2	sp|Q8WUA4|TF3C2_HUMAN	6.3	2	0.863	0.4143
1258	Ubiquitin-protein ligase E3C OS=Homo sapiens GN=UBE3C PE=1 SV=3	sp|Q15386|UBE3C_HUMAN	8	2	0.9817	0.9843
1259	Zinc finger protein ZPR1 OS=Homo sapiens GN=ZPR1 PE=1 SV=1	sp|O75312|ZPR1_HUMAN	9.8	3	0.9727	0.9303
1260	Pyruvate dehydrogenase E1 component subunit beta, mitochondrial OS=Homo sapiens GN=PDHB PE=1 SV=3	sp|P11177|ODPB_HUMAN	16.7	5	1.2134	0.8124
1261	Coiled-coil domain-containing protein 25 OS=Homo sapiens GN=CCDC25	sp|Q86WR0|CCD25_HUMAN	16.8	2	0.871	0.515
1262	S-methyl-5'-thioadenosine phosphorylase OS=Homo sapiens GN=MTAP PE=1 SV=2	sp|Q13126|MTAP_HUMAN	24.4	4	1.0471	0.9147
1263	60S ribosomal protein L19 OS=Homo sapiens GN=RPL19 PE=1 SV=1	sp|P84098|RL19_HUMAN	22.5	11	0.5346	0.6924
1264	SH3 domain-binding glutamic acid-rich-like protein 3 OS=Homo sapiens GN=SH3BGRL3	sp|Q9H299|SH3L3_HUMAN	31.2	5	1.0471	0.8787
1265	Tyrosine-protein kinase HCK OS=Homo sapiens GN=HCK PE=1 SV=5	sp|P08631|HCK_HUMAN	15.6	5	1.2246	0.5958
1266	Long-chain-fatty-acid--CoA ligase 3 OS=Homo sapiens GN=ACSL3 PE=1 SV=3	sp|O95573|ACSL3_HUMAN	9.2	3	1.1482	0.6807
1267	Tubulin--tyrosine ligase-like protein 12 OS=Homo sapiens GN=TTLL12 PE=1	sp|Q14166|TTL12_HUMAN	9.6	3	0.9817	0.9829
1268	Trafficking protein particle complex subunit 11 OS=Homo sapiens GN=TRAPPC11 PE=1 SV=2	sp|Q7Z392|TPC11_HUMAN	6.6	3	1.028	0.9158
1269	Alpha-2-macroglobulin OS=Homo sapiens GN=A2M PE=1 SV=3	sp|P01023|A2MG_HUMAN	3.3	4	0.492	0.3217
1270	Formin-like protein 3 OS=Homo sapiens GN=FMNL3 PE=1	sp|Q8IVF7|FMNL3_HUMAN	5.4	2	1.028	0.8587
1271	Cytochrome c1, heme protein, mitochondrial OS=Homo sapiens GN=CYC1 PE=1 SV=3	sp|P08574|CY1_HUMAN	28	4	0.7656	0.632

1272	Cyclin-dependent kinase 11A OS=Homo sapiens GN=CDK11A PE=1 SV=4	sp|Q9UQ88|CD11A_HUMAN	7.5	2	0.9908	0.9872
1273	Splicing factor, suppressor of white-apricot homolog OS=Homo sapiens GN=SFSWAP	sp|Q12872|SFSWA_HUMAN	6.9	2	0.6668	0.7046
1274	WD repeat-containing protein 37 OS=Homo sapiens GN=WDR37 PE=1	sp|Q9Y2I8|WDR37_HUMAN	8.7	2	1.4997	0.5728
1275	U3 small nucleolar ribonucleoprotein protein IMP4 OS=Homo sapiens GN=IMP4 PE=1 SV=1	sp|Q96G21|IMP4_HUMAN	12.7	2	1.0568	0.8689
1276	60S ribosomal protein L31 OS=Homo sapiens GN=RPL31 PE=1 SV=1	sp|P62899|RL31_HUMAN	23.2	6	1.0186	0.9599
1277	Centrosomal protein of 170 kDa OS=Homo sapiens GN=CEP170 PE=1 SV=1	sp|Q5SW79|CE170_HUMAN	5.1	2	1.0666	0.8749
1278	Centromere/kinetochore protein zw10 homolog OS=Homo sapiens GN=ZW10 PE=1	sp|O43264|ZW10_HUMAN	13.1	5	0.7656	0.8208
1279	Putative pre-mRNA-splicing factor ATP-dependent RNA helicase DHX16 OS=Homo sapiens GN=DHX16 PE=1 SV=2	sp|O60231|DHX16_HUMAN	8.3	3	0.9036	0.8228
1280	ATP-binding cassette sub-family F member 3 OS=Homo sapiens GN=ABCF3 PE=1 SV=2	sp|Q9NUQ8|ABCF3_HUMAN	8.3	2	0.9204	0.3254
1281	DNA mismatch repair protein Msh6 OS=Homo sapiens GN=MSH6 PE=1 SV=2	sp|P52701|MSH6_HUMAN	4.9	4	0.8551	0.7433
1282	Crk-like protein OS=Homo sapiens GN=CRKL PE=1 SV=1	sp|P46109|CRKL_HUMAN	27.1	4	1.0765	0.1959
1283	Replication factor C subunit 2 OS=Homo sapiens GN=RFC2 PE=1	sp|P35250|RFC2_HUMAN	18.6	2	1.0765	0.8526
1284	Syntaxin-7 OS=Homo sapiens GN=STX7 PE=1 SV=4	sp|O15400|STX7_HUMAN	17.2	4	1.4191	0.3434
1285	Omega-amidase NIT2 OS=Homo sapiens GN=NIT2 PE=1 SV=1	sp|Q9NQR4|NIT2_HUMAN	20.3	5	1.0965	0.8383
1286	GA-binding protein alpha chain OS=Homo sapiens GN=GABPA PE=1 SV=1	sp|Q06546|GABPA_HUMAN	7	2	0.9036	0.683
1287	Gamma-enolase OS=Homo sapiens GN=ENO2 PE=1 SV=3	sp|P09104|ENOG_HUMAN	39.4	58	1.1912	0.7901

1288	Protein FAM49A OS=Homo sapiens GN=FAM49A PE=2 SV=1	sp|Q9H0Q0|FA49A_HUMAN	29.7	5	1.2023	0.6681
1289	Sarcoplasmic/endoplasmic reticulum calcium ATPase 3 OS=Homo sapiens GN=ATP2A3 PE=1 SV=2	sp|Q93084|AT2A3_HUMAN	9.3	4	1.1803	0.7169
1290	Nucleosome assembly protein 1-like 4 OS=Homo sapiens GN=NAP1L4 PE=1 SV=1	sp|Q99733|NP1L4_HUMAN	30.7	4	1.0093	0.9787
1291	Core-binding factor subunit beta OS=Homo sapiens GN=CBFB PE=1 SV=2	sp|Q13951|PEBB_HUMAN	27.5	3	0.6855	0.9018
1292	Solute carrier family 2, facilitated glucose transporter member 1 OS=Homo sapiens GN=SLC2A1 PE=1 SV=2	sp|P11166|GTR1_HUMAN	11.8	4	5.3456	0.2128
1293	Docking protein 3 OS=Homo sapiens GN=DOK3 PE=1 SV=2	sp|Q7L591|DOK3_HUMAN	8.1	3	1.2823	0.6304
1294	Pericentriolar material 1 protein OS=Homo sapiens GN=PCM1 PE=1 SV=4	sp|Q15154|PCM1_HUMAN	3.6	3	1.0375	0.766
1295	Small nuclear ribonucleoprotein Sm D3 OS=Homo sapiens GN=SNRPD3 PE=1 SV=1	sp|P62318|SMD3_HUMAN	31	2	0.9638	0.9155
1296	Thioredoxin-interacting protein OS=Homo sapiens GN=TXNIP PE=1 SV=1	sp|Q9H3M7|TXNIP_HUMAN	14.8	2	2.0893	0.2237
1297	N-acetylgalactosamine kinase OS=Homo sapiens GN=GALK2 PE=1 SV=1	sp|Q01415|GALK2_HUMAN	12.7	2	1.1695	0.6787
1298	Nuclear transport factor 2 OS=Homo sapiens GN=NUTF2 PE=1	sp|P61970|NTF2_HUMAN	23.6	3	1	0.9884
1299	SUMO-activating enzyme subunit 1 OS=Homo sapiens GN=SAE1 PE=1 SV=1	sp|Q9UBE0|SAE1_HUMAN	25.4	5	0.7178	0.9124
1300	tRNA (guanine(26)-N(2))-dimethyltransferase OS=Homo sapiens GN=TRMT1 PE=1	sp|Q9NXH9|TRM1_HUMAN	10.6	3	0.871	0.7954
1301	Methylcrotonoyl-CoA carboxylase beta chain, mitochondrial OS=Homo sapiens GN=MCCC2 PE=1 SV=1	sp|Q9HCC0|MCCB_HUMAN	15.6	17	1.1376	0.7856
1302	FAD synthase OS=Homo sapiens GN=FLAD1 PE=1 SV=1	sp|Q8NFF5|FAD1_HUMAN	8.2	2	0.8954	0.839

1303	StAR-related lipid transfer protein 3 OS=Homo sapiens GN=STARD3 PE=1 SV=2	sp|Q14849|STAR3_HUMAN	15.1	4	1.028	0.9521
1304	Arf-GAP domain and FG repeat-containing protein 2 OS=Homo sapiens GN=AGFG2 PE=1	sp|O95081|AGFG2_HUMAN	8.7	2	0.929	0.7351
1305	Pre-mRNA-splicing factor RBM22 OS=Homo sapiens GN=RBM22 PE=1	sp|Q9NW64|RBM22_HUMAN	11.9	2	0.9638	0.8593
1306	Synaptic vesicle membrane protein VAT-1 homolog OS=Homo sapiens GN=VAT1 PE=1 SV=2	sp|Q99536|VAT1_HUMAN	19.6	3	2.3768	0.3079
1307	Protein IWS1 homolog OS=Homo sapiens GN=IWS1 PE=1	sp|Q96ST2|IWS1_HUMAN	6.3	2	0.7112	0.7668
1308	Phosphorylated adapter RNA export protein OS=Homo sapiens GN=PHAX PE=1	sp|Q9H814|PHAX_HUMAN	9.1	2	0.912	0.8739
1309	Cleft lip and palate transmembrane protein 1 OS=Homo sapiens GN=CLPTM1 PE=1 SV=1	sp|O96005|CLPT1_HUMAN	6.7	2	0.7112	0.7669
1310	Alpha-soluble NSF attachment protein OS=Homo sapiens GN=NAPA PE=1 SV=3	sp|P54920|SNAA_HUMAN	13.9	3	1.0375	0.8193
1311	Low molecular weight phosphotyrosine protein phosphatase OS=Homo sapiens GN=ACP1 PE=1 SV=3	sp|P24666|PPAC_HUMAN	30.4	5	0.9036	0.8863
1312	NADH-cytochrome b5 reductase 3 OS=Homo sapiens GN=CYB5R3 PE=1 SV=3	sp|P00387|NB5R3_HUMAN	17.3	6	1.7061	0.626
1313	Peroxisomal membrane protein PMP34 OS=Homo sapiens GN=SLC25A17 PE=1 SV=1	sp|O43808|PM34_HUMAN	12.4	2	0.9817	0.9863
1314	Small acidic protein OS=Homo sapiens GN=SMAP PE=1 SV=1	sp|O00193|SMAP_HUMAN	21.3	3	0.6081	0.6309
1315	Alpha-actinin-1 OS=Homo sapiens GN=ACTN1 PE=1 SV=2	sp|P12814|ACTN1_HUMAN	17.9	14	2.2699	0.3512
1316	Heat shock 70 kDa protein 1B OS=Homo sapiens GN=HSPA1B PE=1 SV=1	sp|P0DMV9|HS71B_HUMAN	47	32	1.1588	0.5866
1317	AP-2 complex subunit alpha-1 OS=Homo sapiens GN=AP2A1 PE=1 SV=3	sp|O95782|AP2A1_HUMAN	11.4	7	1.0375	0.9051
1318	Poly(rC)-binding protein 2 OS=Homo sapiens GN=PCBP2 PE=1	sp|Q15366|PCBP2_HUMAN	24.9	10	0.6081	0.5819

1319	Histone deacetylase 2 OS=Homo sapiens GN=HDAC2 PE=1	sp|Q92769|HDAC2_HUMAN	15.8	7	0.8954	0.7257
1320	14-3-3 protein beta/alpha OS=Homo sapiens GN=YWHAB PE=1 SV=3	sp|P31946|1433B_HUMAN	45.9	29	0.8551	0.8348
1321	Ubiquitin carboxyl-terminal hydrolase 8 OS=Homo sapiens GN=USP8 PE=1 SV=1	sp|P40818|UBP8_HUMAN	9.1	5	0.8318	0.6933
1322	Differentially expressed in FDCP 6 homolog OS=Homo sapiens GN=DEF6 PE=1	sp|Q9H4E7|DEFI6_HUMAN	10.1	3	1.0375	0.8896
1323	Inosine-5'-monophosphate dehydrogenase 1 OS=Homo sapiens GN=IMPDH1 PE=1 SV=2	sp|P20839|IMDH1_HUMAN	14	5	0.9817	0.9735
1324	Leucine-rich repeat-containing protein 14 OS=Homo sapiens GN=LRRC14	sp|Q15048|LRC14_HUMAN	18.9	3	0.929	0.8939
1325	Probable ATP-dependent RNA helicase DDX10 OS=Homo sapiens GN=DDX10 PE=1 SV=2	sp|Q13206|DDX10_HUMAN	6.4	3	0.863	0.7099
1326	Importin-8 OS=Homo sapiens GN=IPO8 PE=1 SV=2	sp|O15397|IPO8_HUMAN	5.2	3	0.5012	0.6145
1327	Sister chromatid cohesion protein PDS5 homolog B OS=Homo sapiens GN=PDS5B PE=1 SV=1	sp|Q9NTI5|PDS5B_HUMAN	3.7	3	0.912	0.2527
1328	Delta-aminolevulinic acid dehydratase OS=Homo sapiens GN=ALAD PE=1 SV=1	sp|P13716|HEM2_HUMAN	10	3	0.9462	0.889
1329	Ras-related protein Rab-5A OS=Homo sapiens GN=RAB5A PE=1	sp|P20339|RAB5A_HUMAN	27.4	5	1.2359	0.6404
1330	Peptidyl-prolyl cis-trans isomerase H OS=Homo sapiens GN=PPIH PE=1 SV=1	sp|O43447|PPIH_HUMAN	20.9	4	0.9817	0.9906
1331	Exportin-5 OS=Homo sapiens GN=XPO5 PE=1 SV=1	sp|Q9HAV4|XPO5_HUMAN	9.2	5	0.929	0.697
1332	Filamin-A OS=Homo sapiens GN=FLNA PE=1 SV=4	sp|P21333|FLNA_HUMAN	3.2	3	1.3677	0.3594
1333	Small nuclear ribonucleoprotein Sm D2 OS=Homo sapiens GN=SNRPD2 PE=1 SV=1	sp|P62316|SMD2_HUMAN	32.2	3	0.8872	0.653
1334	Glucose-induced degradation protein 8 homolog OS=Homo sapiens GN=GID8 PE=1 SV=1	sp|Q9NWU2|GID8_HUMAN	21.1	3	0.9638	0.8742

1335	Probable ATP-dependent RNA helicase DDX47 OS=Homo sapiens GN=DDX47 PE=1 SV=1	sp|Q9H0S4|DDX47_HUMAN	9.2	3	0.929	0.8046
1336	Protein FAM98A OS=Homo sapiens GN=FAM98A PE=1 SV=1	sp|Q8NCA5|FA98A_HUMAN	8.9	4	0.9908	0.9351
1337	Transmembrane 9 superfamily member 3 OS=Homo sapiens GN=TM9SF3 PE=1 SV=2	sp|Q9HD45|TM9S3_HUMAN	6.8	3	0.7586	0.7362
1338	Transcription elongation regulator 1 OS=Homo sapiens GN=TCERG1	sp|O14776|TCRG1_HUMAN	6.5	3	0.9908	0.9854
1339	Ubiquitin carboxyl-terminal hydrolase 15 OS=Homo sapiens GN=USP15 PE=1	sp|Q9Y4E8|UBP15_HUMAN	7	4	0.9727	0.9641
1340	High affinity cationic amino acid transporter 1 OS=Homo sapiens GN=SLC7A1 PE=1 SV=1	sp|P30825|CTR1_HUMAN	6.4	4	1.1376	0.7556
1341	TFIIH basal transcription factor complex helicase XPD subunit OS=Homo sapiens GN=ERCC2 PE=1 SV=1	sp|P18074|ERCC2_HUMAN	8	3	0.9462	0.7414
1342	NEDD8-activating enzyme E1 regulatory subunit OS=Homo sapiens GN=NAE1 PE=1 SV=1	sp|Q13564|ULA1_HUMAN	9.7	3	1.0186	0.9119
1343	Amyloid beta A4 precursor protein-binding family B member 1-interacting protein OS=Homo sapiens GN=APBB1IP PE=1 SV=1	sp|Q7Z5R6|AB1IP_HUMAN	5.6	2	1.1912	0.7181
1344	GPI transamidase component PIG-S OS=Homo sapiens GN=PIGS PE=1 SV=3	sp|Q96S52|PIGS_HUMAN	9.7	4	0.9376	0.906
1345	Probable RNA-binding protein 19 OS=Homo sapiens GN=RBM19 PE=1 SV=3	sp|Q9Y4C8|RBM19_HUMAN	5.5	3	0.8872	0.8273
1346	Conserved oligomeric Golgi complex subunit 3 OS=Homo sapiens GN=COG3 PE=1 SV=3	sp|Q96JB2|COG3_HUMAN	3.9	3	1.0375	0.8375
1347	Kinesin-like protein KIF2A OS=Homo sapiens GN=KIF2A PE=1	sp|O00139|KIF2A_HUMAN	8.1	3	0.9908	0.9909
1348	DNA-directed RNA polymerase III subunit RPC6 OS=Homo sapiens GN=POLR3F PE=1 SV=1	sp|Q9H1D9|RPC6_HUMAN	16.5	2	0.871	0.7812
1349	Ubiquitin-associated protein 2 OS=Homo sapiens GN=UBAP2 PE=1	sp|Q5T6F2|UBAP2_HUMAN	5.6	2		

1350	Reticulon-4 OS=Homo sapiens GN=RTN4 PE=1 SV=2	sp|Q9NQC3|RTN4_HUMAN	3.7	2	0.955	0.9918
1351	U4/U6 small nuclear ribonucleoprotein Prp3 OS=Homo sapiens	sp|O43395|PRPF3_HUMAN	11.1	5	0.6081	0.6079
1352	Proteasome assembly chaperone 2 OS=Homo sapiens GN=PSMG2 PE=1	sp|Q969U7|PSMG2_HUMAN	10.6	4	0.9817	0.9409
1353	Ubiquinone biosynthesis protein COQ9, mitochondrial OS=Homo sapiens GN=COQ9 PE=1 SV=1	sp|O75208|COQ9_HUMAN	17	2	0.9638	0.8727
1354	Glucosamine 6-phosphate N-acetyltransferase OS=Homo sapiens GN=GNPNAT1 PE=1 SV=1	sp|Q96EK6|GNA1_HUMAN	23.9	2	1.1066	0.7028
1355	Bifunctional 3'-phosphoadenosine 5'-phosphosulfate synthase 1 OS=Homo sapiens GN=PAPSS1 PE=1 SV=2	sp|O43252|PAPS1_HUMAN	9.1	4	1.0568	0.9023
1356	Protein arginine N-methyltransferase 6 OS=Homo sapiens GN=PRMT6 PE=1 SV=1	sp|Q96LA8|ANM6_HUMAN	8.3	2	0.9204	0.8937
1357	Serine/arginine-rich splicing factor 2 OS=Homo sapiens GN=SRSF2 PE=1 SV=4	sp|Q01130|SRSF2_HUMAN	21.7	5	0.6486	0.8463
1358	Ras association domain-containing protein 2 OS=Homo sapiens GN=RASSF2 PE=1 SV=1	sp|P50749|RASF2_HUMAN	21.5	2	0.9638	0.9526
1359	La-related protein 4B OS=Homo sapiens GN=LARP4B PE=1 SV=3	sp|Q92615|LAR4B_HUMAN	10	2	1.0666	0.8547
1360	Mitochondrial-processing peptidase subunit beta OS=Homo sapiens GN=PMPCB PE=1	sp|O75439|MPPB_HUMAN	9	2	0.9376	0.8064
1361	Eukaryotic translation initiation factor 4E type 2 OS=Homo sapiens GN=EIF4E2 PE=1	sp|O60573|IF4E2_HUMAN	13.1	2	0.7047	0.5466
1362	Cleavage stimulation factor subunit 1 OS=Homo sapiens GN=CSTF1 PE=1	sp|Q05048|CSTF1_HUMAN	7.4	3	1.0471	0.8588
1363	AP-1 complex subunit sigma-1A OS=Homo sapiens GN=AP1S1 PE=1 SV=1	sp|P61966|AP1S1_HUMAN	25.3	3	1.0471	0.7976
1364	WD repeat-containing protein 81 OS=Homo sapiens GN=WDR81 PE=1	sp|Q562E7|WDR81_HUMAN	4.4	2	1.0864	0.7748

1365	CAP-Gly domain-containing linker protein 1 OS=Homo sapiens GN=CLIP1 PE=1	sp|P30622|CLIP1_HUMAN	7.7	4	1.1169	0.7831
1366	Phospholipid-transporting ATPase IA OS=Homo sapiens GN=ATP8A1 PE=1 SV=1	sp|Q9Y2Q0|AT8A1_HUMAN	5.6	2	0.9727	0.9669
1367	Evolutionarily conserved signaling intermediate in Toll pathway, mitochondrial OS=Homo sapiens GN=ECSIT PE=1 SV=1	sp|Q9BQ95|ECSIT_HUMAN	13	2	1.0093	0.9786
1368	Ceramide synthase 2 OS=Homo sapiens GN=CERS2 PE=1	sp|Q96G23|CERS2_HUMAN	17.1	4	1.1169	0.8074
1369	PDZ and LIM domain protein 2 OS=Homo sapiens GN=PDLIM2 PE=1 SV=1	sp|Q96JY6|PDLI2_HUMAN	16.2	3	0.955	0.9204
1370	DnaJ homolog subfamily C member 10 OS=Homo sapiens GN=DNAJC10 PE=1 SV=2	sp|Q8IXB1|DJC10_HUMAN	6.4	2	1.1272	0.5731
1371	Elongation factor Tu GTP-binding domain-containing protein 1 OS=Homo sapiens GN=EFTUD1 PE=1 SV=2	sp|Q7Z2Z2|ETUD1_HUMAN	7.7	2	1.0864	0.8191
1372	Probable E3 ubiquitin-protein ligase HERC4 OS=Homo sapiens GN=HERC4 PE=1 SV=1	sp|Q5GLZ8|HERC4_HUMAN	5.6	2	1.5417	0.3917
1373	ATP-dependent Clp protease ATP-binding subunit clpX-like, mitochondrial OS=Homo sapiens GN=CLPX PE=1 SV=2	sp|O76031|CLPX_HUMAN	9.5	2	1.0568	0.7301
1374	WD repeat and HMG-box DNA-binding protein 1 OS=Homo sapiens GN=WDHD1 PE=1 SV=1	sp|O75717|WDHD1_HUMAN	4.9	5	0.6792	0.5071
1375	Oxysterol-binding protein-related protein 2 OS=Homo sapiens GN=OSBPL2 PE=1 SV=1	sp|Q9H1P3|OSBL2_HUMAN	7.9	2	1.0666	0.9241
1376	Integrin-linked kinase-associated serine/threonine phosphatase 2C OS=Homo sapiens GN=ILKAP PE=1 SV=1	sp|Q9H0C8|ILKAP_HUMAN	11	4	1.0093	0.9201
1377	Tether containing UBX domain for GLUT4 OS=Homo sapiens GN=ASPSCR1 PE=1 SV=1	sp|Q9BZE9|ASPC1_HUMAN	10.7	2	0.9817	0.8927

1378	Zinc finger CCHC domain-containing protein 8 OS=Homo sapiens GN=ZCCHC8 PE=1 SV=2	sp|Q6NZY4|ZCHC8_HUMAN	5.8	2	0.8551	0.7003
1379	Pleckstrin OS=Homo sapiens GN=PLEK PE=1 SV=3	sp|P08567|PLEK_HUMAN	16	3	1.9588	0.3315
1380	NFU1 iron-sulfur cluster scaffold homolog, mitochondrial OS=Homo sapiens GN=NFU1 PE=1 SV=2	sp|Q9UMS0|NFU1_HUMAN	17.7	2	0.8017	0.6735
1381	tRNA (guanine-N(7)-)-methyltransferase OS=Homo sapiens GN=METTL1 PE=1 SV=1	sp|Q9UBP6|TRMB_HUMAN	12.3	2	0.7943	0.6566
1382	Ethanolamine-phosphate cytidylyltransferase OS=Homo sapiens GN=PCYT2 PE=1 SV=1	sp|Q99447|PCY2_HUMAN	8.2	2	1.1912	0.6309
1383	Eukaryotic translation initiation factor 1A, X-chromosomal OS=Homo sapiens GN=EIF1AX PE=1 SV=2	sp|P47813|IF1AX_HUMAN	21.5	2	0.9376	0.7671
1384	Kinase D-interacting substrate of 220 kDa OS=Homo sapiens	sp|Q9ULH0|KDIS_HUMAN	6.7	2	1.0864	0.7701
1385	AP-4 complex subunit epsilon-1 OS=Homo sapiens GN=AP4E1 PE=1 SV=2	sp|Q9UPM8|AP4E1_HUMAN	6.6	2	1.1376	0.7121
1386	Remodeling and spacing factor 1 OS=Homo sapiens GN=RSF1 PE=1 SV=2	sp|Q96T23|RSF1_HUMAN	4.8	2	1.0765	0.6134
1387	Multidrug resistance protein 1 OS=Homo sapiens GN=ABCB1 PE=1	sp|P08183|MDR1_HUMAN	7.1	2	1	0.9845
1388	Manganese-transporting ATPase 13A1 OS=Homo sapiens GN=ATP13A1 PE=1 SV=2	sp|Q9HD20|AT131_HUMAN	5.3	2	0.9908	0.9176
1389	ATP-dependent zinc metalloprotease YME1L1 OS=Homo sapiens GN=YME1L1	sp|Q96TA2|YMEL1_HUMAN	8.7	2	0.6138	0.5447
1390	Metastasis-associated protein MTA1 OS=Homo sapiens GN=MTA1 PE=1 SV=2	sp|Q13330|MTA1_HUMAN	10.5	2	0.879	0.8227
1391	CDK5 regulatory subunit-associated protein 3 OS=Homo sapiens GN=CDK5RAP3 PE=1 SV=2	sp|Q96JB5|CK5P3_HUMAN	9.3	2	1.0666	0.8384
1392	TBC1 domain family member 9B OS=Homo sapiens GN=TBC1D9B PE=1 SV=3	sp|Q66K14|TBC9B_HUMAN	3.8	2	1.2134	0.6009

1393	Pre-mRNA-splicing factor 38B OS=Homo sapiens GN=PRPF38B PE=1 SV=1	sp|Q5VTL8|PR38B_HUMAN	13.6	2	1.028	0.7514
1394	Alpha-mannosidase 2C1 OS=Homo sapiens GN=MAN2C1 PE=1 SV=1	sp|Q9NTJ4|MA2C1_HUMAN	6.3	3	1.2134	0.6987
1395	Nuclear pore complex protein Nup153 OS=Homo sapiens GN=NUP153 PE=1 SV=2	sp|P49790|NU153_HUMAN	4.7	2	0.879	0.8283
1396	Nuclear autoantigenic sperm protein OS=Homo sapiens GN=NASP PE=1	sp|P49321|NASP_HUMAN	16	14	0.6138	0.6225
1397	GDH/6PGL endoplasmic bifunctional protein OS=Homo sapiens GN=H6PD PE=1 SV=2	sp|O95479|G6PE_HUMAN	6.2	3	1.0864	0.6355
1398	ATP-dependent RNA helicase DHX36 OS=Homo sapiens GN=DHX36 PE=1	sp|Q9H2U1|DHX36_HUMAN	6.9	2	0.871	0.7489
1399	Armadillo repeat-containing protein 8 OS=Homo sapiens GN=ARMC8 PE=1	sp|Q8IUR7|ARMC8_HUMAN	10	3	0.9462	0.9129
1400	Condensin-2 complex subunit G2 OS=Homo sapiens GN=NCAPG2	sp|Q86XI2|CNDG2_HUMAN	4.7	2	0.631	0.4512
1401	Ubiquitin-protein ligase E3A OS=Homo sapiens GN=UBE3A PE=1 SV=4	sp|Q05086|UBE3A_HUMAN	7.2	2	1.0568	0.738
1402	N-alpha-acetyltransferase 10 OS=Homo sapiens GN=NAA10 PE=1 SV=1	sp|P41227|NAA10_HUMAN	21.7	2	0.871	0.6493
1403	Citrate synthase, mitochondrial OS=Homo sapiens GN=CS PE=1	sp|O75390|CISY_HUMAN	8.2	3	1.1695	0.6309
1404	RNA polymerase II subunit A C-terminal domain phosphatase OS=Homo sapiens GN=CTDP1 PE=1	sp|Q9Y5B0|CTDP1_HUMAN	4.5	2	0.929	0.7857
1405	Serine/threonine-protein kinase SIK3 OS=Homo sapiens GN=SIK3 PE=1 SV=3	sp|Q9Y2K2|SIK3_HUMAN	3.9	2	1.028	0.9542
1406	Anaphase-promoting complex subunit 5 OS=Homo sapiens GN=ANAPC5 PE=1 SV=2	sp|Q9UJX4|APC5_HUMAN	7	4	0.929	0.821
1407	tRNA (adenine(58)-N(1))-methyltransferase non-catalytic subunit TRM6 OS=Homo sapiens GN=TRMT6 PE=1	sp|Q9UJA5|TRM6_HUMAN	12.3	3	1.028	0.9929

1408	WD repeat-containing protein mio OS=Homo sapiens GN=MIOS PE=1 SV=2	sp|Q9NXC5|MIO_HUMAN	4.5	2	1.3428	0.5757
1409	Phenylalanine--tRNA ligase beta subunit OS=Homo sapiens GN=FARSB PE=1	sp|Q9NSD9|SYFB_HUMAN	10.5	3	1.028	0.9369
1410	CCR4-NOT transcription complex subunit 10 OS=Homo sapiens GN=CNOT10 PE=1 SV=1	sp|Q9H9A5|CNO10_HUMAN	7.1	2	0.8241	0.7117
1411	DnaJ homolog subfamily C member 2 OS=Homo sapiens GN=DNAJC2 PE=1 SV=4	sp|Q99543|DNJC2_HUMAN	10.5	2	0.8472	0.7597
1412	RNA polymerase-associated protein RTF1 homolog OS=Homo sapiens GN=RTF1 PE=1 SV=4	sp|Q92541|RTF1_HUMAN	6.5	3	0.9204	0.9602
1413	Heterogeneous nuclear ribonucleoprotein D0 OS=Homo sapiens GN=HNRNPD PE=1 SV=1	sp|Q14103|HNRPD_HUMAN	18.9	5	1.0471	0.8989
1414	V-type proton ATPase 116 kDa subunit a isoform 3 OS=Homo sapiens GN=TCIRG1 PE=1 SV=3	sp|Q13488|VPP3_HUMAN	5.8	2	1.4191	0.5205
1415	Immunoglobulin-binding protein 1 OS=Homo sapiens GN=IGBP1 PE=1 SV=1	sp|P78318|IGBP1_HUMAN	15	2	0.929	0.9156
1416	Cyclin-dependent kinase 7 OS=Homo sapiens GN=CDK7 PE=1 SV=1	sp|P50613|CDK7_HUMAN	19.7	2	0.929	0.9268
1417	Splicing factor, arginine/serine-rich 15 OS=Homo sapiens GN=SCAF4 PE=1	sp|O95104|SFR15_HUMAN	3.7	2	1.0186	0.9639
1418	SH2B adapter protein 2 OS=Homo sapiens GN=SH2B2 PE=1	sp|O14492|SH2B2_HUMAN	7.1	2	0.9462	0.9274
1419	GTP-binding protein 1 OS=Homo sapiens GN=GTPBP1	sp|O00178|GTPB1_HUMAN	6.9	3	1.0666	0.8436
1420	Syntaxin-8 OS=Homo sapiens GN=STX8 PE=1 SV=2	sp|Q9UNK0|STX8_HUMAN	17.8	2	1.1169	0.7932
1421	ADP-ribosylation factor-binding protein GGA1 OS=Homo sapiens GN=GGA1 PE=1 SV=1	sp|Q9UJY5|GGA1_HUMAN	7.7	3	1.3932	0.6979
1422	Nuclear distribution protein nudE homolog 1 OS=Homo sapiens GN=NDE1 PE=1 SV=2	sp|Q9NXR1|NDE1_HUMAN	14.7	2	0.3698	0.3508
1423	Myotubularin-related protein 9 OS=Homo sapiens GN=MTMR9 PE=1 SV=1	sp|Q96QG7|MTMR9_HUMAN	5.8	2	0.879	0.7713

1424	DAZ-associated protein 1 OS=Homo sapiens GN=DAZAP1 PE=1 SV=1	sp|Q96EP5|DAZP1_HUMAN	16.7	2	1.028	0.9286
1425	General transcription factor IIH subunit 4 OS=Homo sapiens GN=GTF2H4 PE=2 SV=1	sp|Q92759|TF2H4_HUMAN	10.6	2	1.0186	0.8293
1426	Histone-arginine methyltransferase CARM1 OS=Homo sapiens GN=CARM1 PE=1 SV=3	sp|Q86X55|CARM1_HUMAN	7.1	3		
1427	UPF0553 protein C9orf64 OS=Homo sapiens GN=C9orf64 PE=1 SV=1	sp|Q5T6V5|CI064_HUMAN	13.2	2	1.0471	0.7907
1428	Putative oxidoreductase GLYR1 OS=Homo sapiens GN=GLYR1 PE=1	sp|Q49A26|GLYR1_HUMAN	8.1	2	0.9036	0.7676
1429	Electron transfer flavoprotein-ubiquinone oxidoreductase, mitochondrial OS=Homo sapiens GN=ETFDH PE=1 SV=2	sp|Q16134|ETFD_HUMAN	9.7	2	1.0765	0.7979
1430	Lymphoid-restricted membrane protein OS=Homo sapiens GN=LRMP PE=1	sp|Q12912|LRMP_HUMAN	7	2	1.1695	0.7448
1431	Tyrosine-protein phosphatase non-receptor type 7 OS=Homo sapiens GN=PTPN7 PE=1 SV=3	sp|P35236|PTN7_HUMAN	10	2	1.0093	0.9857
1432	cAMP-dependent protein kinase type I-alpha regulatory subunit OS=Homo sapiens GN=PRKAR1A PE=1 SV=1	sp|P10644|KAP0_HUMAN	19.7	2	0.9376	0.9237
1433	Regulator of G-protein signaling 14 OS=Homo sapiens GN=RGS14 PE=1 SV=4	sp|O43566|RGS14_HUMAN	10.3	2	0.8395	0.7368
1434	Probable ATP-dependent RNA helicase DDX49 OS=Homo sapiens GN=DDX49 PE=1 SV=1	sp|Q9Y6V7|DDX49_HUMAN	9.3	3	0.8872	0.8562
1435	EKC/KEOPS complex subunit TPRKB OS=Homo sapiens GN=TPRKB PE=1	sp|Q9Y3C4|TPRKB_HUMAN	14.9	2	0.9376	0.9085
1436	Polymerase delta-interacting protein 2 OS=Homo sapiens GN=POLDIP2	sp|Q9Y2S7|PDIP2_HUMAN	8.7	2	0.912	0.8491
1437	Tyrosyl-DNA phosphodiesterase 1 OS=Homo sapiens GN=TDP1 PE=1 SV=2	sp|Q9NUW8|TYDP1_HUMAN	5.6	2	0.9908	0.9886
1438	UPF0515 protein C19orf66 OS=Homo sapiens GN=C19orf66 PE=1 SV=2	sp|Q9NUL5|CS066_HUMAN	15.8	2	0.912	0.8307

1439	Retinol dehydrogenase 14 OS=Homo sapiens GN=RDH14 PE=1 SV=1	sp|Q9HBH5|RDH14_HUMAN	11.3	2	1.1588	0.577
1440	Acyl-CoA dehydrogenase family member 9, mitochondrial OS=Homo sapiens GN=ACAD9 PE=1 SV=1	sp|Q9H845|ACAD9_HUMAN	7.7	2	1.0666	0.6323
1441	Exocyst complex component 4 OS=Homo sapiens GN=EXOC4 PE=1 SV=1	sp|Q96A65|EXOC4_HUMAN	4.8	2	0.9908	0.93
1442	Ras-related protein Rab-31 OS=Homo sapiens GN=RAB31 PE=1	sp|Q13636|RAB31_HUMAN	23.7	3	1.2942	0.6224
1443	F-BAR domain only protein 2 OS=Homo sapiens GN=FCHO2 PE=1	sp|Q0JRZ9|FCHO2_HUMAN	4.3	2	1.0186	0.9646
1444	Apoptosis regulator BAX OS=Homo sapiens GN=BAX PE=1 SV=1	sp|Q07812|BAX_HUMAN	29.2	4		
1445	Exosome complex component RRP45 OS=Homo sapiens GN=EXOSC9 PE=1 SV=3	sp|Q06265|EXOS9_HUMAN	10	2	0.9036	0.7124
1446	Cytochrome c OS=Homo sapiens GN=CYCS PE=1 SV=2	sp|P99999|CYC_HUMAN	31.4	5	0.9638	0.9764
1447	Annexin A11 OS=Homo sapiens GN=ANXA11 PE=1 SV=1	sp|P50995|ANX11_HUMAN	7.1	2	1.028	0.9378
1448	DNA polymerase delta subunit 2 OS=Homo sapiens GN=POLD2 PE=1 SV=1	sp|P49005|DPOD2_HUMAN	14.1	3	0.8954	0.9923
1449	Protein S100-A11 OS=Homo sapiens GN=S100A11 PE=1 SV=2	sp|P31949|S10AB_HUMAN	33.3	3	2.5119	0.3996
1450	Cyclin-dependent kinase 1 OS=Homo sapiens GN=CDK1 PE=1 SV=3	sp|P06493|CDK1_HUMAN	17.5	2	0.4742	0.3872
1451	LETM1 and EF-hand domain-containing protein 1, mitochondrial OS=Homo sapiens GN=LETM1 PE=1 SV=1	sp|O95202|LETM1_HUMAN	7.2	11	1.1272	0.7526
1452	Aflatoxin B1 aldehyde reductase member 2 OS=Homo sapiens GN=AKR7A2 PE=1 SV=3	sp|O43488|ARK72_HUMAN	9.7	2	1.0471	0.8921
1453	WASH complex subunit CCDC53 OS=Homo sapiens GN=CCDC53 PE=1 SV=1	sp|Q9Y3C0|CCD53_HUMAN	24.2	2	1.0471	0.9423
1454	Synergin gamma OS=Homo sapiens GN=SYNRG PE=1 SV=2	sp|Q9UMZ2|SYNRG_HUMAN	2.5	2	1.0186	0.9155
1455	Protein NDRG3 OS=Homo sapiens GN=NDRG3 PE=1 SV=2	sp|Q9UGV2|NDRG3_HUMAN	12	2	1	0.9946

1456	Mitochondrial dicarboxylate carrier OS=Homo sapiens GN=SLC25A10 PE=1 SV=2	sp|Q9UBX3|DIC_HUMAN	25.8	6	1.0093	0.8612
1457	Ubiquitin-associated protein 1 OS=Homo sapiens GN=UBAP1 PE=1	sp|Q9NZ09|UBAP1_HUMAN	8.6	2	1.1169	0.8309
1458	Serine/threonine-protein phosphatase 4 regulatory subunit 2 OS=Homo sapiens GN=PPP4R2 PE=1	sp|Q9NY27|PP4R2_HUMAN	9.4	2	0.863	0.7845
1459	Transmembrane protein 165 OS=Homo sapiens GN=TMEM165 PE=1 SV=1	sp|Q9HC07|TM165_HUMAN	14.2	2	0.929	0.7823
1460	Protein FAM192A OS=Homo sapiens GN=FAM192A PE=1 SV=1	sp|Q9GZU8|F192A_HUMAN	12.2	2	0.4529	0.5135
1461	Dehydrogenase/reductase SDR family member 4 OS=Homo sapiens GN=DHRS4 PE=1 SV=3	sp|Q9BTZ2|DHRS4_HUMAN	13.7	2	1.0568	0.8884
1462	COP9 signalosome complex subunit 8 OS=Homo sapiens GN=COPS8 PE=1 SV=1	sp|Q99627|CSN8_HUMAN	17.7	2	1.0093	0.9826
1463	Dehydrogenase/reductase SDR family member 1 OS=Homo sapiens GN=DHRS1 PE=1 SV=1	sp|Q96LJ7|DHRS1_HUMAN	13.4	2	1.1066	0.8191
1464	Coiled-coil domain-containing protein 93 OS=Homo sapiens GN=CCDC93	sp|Q567U6|CCD93_HUMAN	7.6	2	1.0568	0.7925
1465	Mortality factor 4-like protein 2 OS=Homo sapiens GN=MORF4L2 PE=1 SV=1	sp|Q15014|MO4L2_HUMAN	11.5	2	0.673	0.3794
1466	Eukaryotic translation initiation factor 3 subunit I OS=Homo sapiens GN=EIF3I PE=1	sp|Q13347|EIF3I_HUMAN	12.6	2	0.9908	0.9668
1467	Serine/arginine-rich splicing factor 1 OS=Homo sapiens GN=SRSF1 PE=1 SV=2	sp|Q07955|SRSF1_HUMAN	14.5	2	0.9638	0.9227
1468	Tyrosine-protein phosphatase non-receptor type 12 OS=Homo sapiens GN=PTPN12 PE=1 SV=3	sp|Q05209|PTN12_HUMAN	4.4	2	1.1588	0.7064
1469	Tumor protein D52 OS=Homo sapiens GN=TPD52 PE=1 SV=2	sp|P55327|TPD52_HUMAN	17.4	2	1.1482	0.7526
1470	Proteasome subunit beta type-3 OS=Homo sapiens GN=PSMB3 PE=1 SV=2	sp|P49720|PSB3_HUMAN	19.5	3	1.0666	0.873
1471	Glycogenin-1 OS=Homo sapiens GN=GYG1 PE=1 SV=4	sp|P46976|GLYG_HUMAN	12.6	3	1.0093	0.9977

1472	ADP-ribosylation factor-like protein 2 OS=Homo sapiens GN=ARL2 PE=1 SV=4	sp|P36404|ARL2_HUMAN	22.3	2	1.1272	0.7664
1473	Glutaredoxin-1 OS=Homo sapiens GN=GLRX PE=1 SV=2	sp|P35754|GLRX1_HUMAN	40.6	2	1.4588	0.5997
1474	General transcription factor IIF subunit 1 OS=Homo sapiens GN=GTF2F1 PE=1 SV=2	sp|P35269|T2FA_HUMAN	10.6	3	0.912	0.7729
1475	Deoxyuridine 5'-triphosphate nucleotidohydrolase, mitochondrial OS=Homo sapiens GN=DUT PE=1 SV=4	sp|P33316|DUT_HUMAN	13.9	2	0.787	0.6125
1476	Phosphatidylethanolamine-binding protein 1 OS=Homo sapiens GN=PEBP1 PE=1	sp|P30086|PEBP1_HUMAN	28.3	4	1.0471	0.8871
1477	Proteasome subunit beta type-8 OS=Homo sapiens GN=PSMB8 PE=1 SV=3	sp|P28062|PSB8_HUMAN	20.7	2	1.4322	0.5194
1478	POU domain, class 2, transcription factor 1 OS=Homo sapiens GN=POU2F1 PE=1 SV=2	sp|P14859|PO2F1_HUMAN	5.1	2	0.6982	0.5279
1479	Translationally-controlled tumor protein OS=Homo sapiens GN=TPT1 PE=1 SV=1	sp|P13693|TCTP_HUMAN	20.4	2	1	0.9866
1480	Serine/arginine-rich splicing factor 10 OS=Homo sapiens GN=SRSF10 PE=1 SV=1	sp|O75494|SRS10_HUMAN	15.7	2	0.9204	0.7882
1481	Origin recognition complex subunit 4 OS=Homo sapiens GN=ORC4 PE=1 SV=2	sp|O43929|ORC4_HUMAN	6.4	2	0.6546	0.5516
1482	ATPase ASNA1 OS=Homo sapiens GN=ASNA1 PE=1 SV=2	sp|O43681|ASNA_HUMAN	14.9	2	0.8395	0.9025
1483	Density-regulated protein OS=Homo sapiens GN=DENR PE=1 SV=2	sp|O43583|DENR_HUMAN	27.8	3	0.929	0.9524
1484	Synaptobrevin homolog YKT6 OS=Homo sapiens GN=YKT6 PE=1 SV=1	sp|O15498|YKT6_HUMAN	21.2	3	1.0568	0.6448
1485	Membrane-associated progesterone receptor component 2 OS=Homo sapiens GN=PGRMC2	sp|O15173|PGRC2_HUMAN	11.2	2	1.1695	0.6932
1486	Syntenin-1 OS=Homo sapiens GN=SDCBP PE=1 SV=1	sp|O00560|SDCB1_HUMAN	17.8	2		
1487	Serine/threonine-protein kinase 25 OS=Homo sapiens GN=STK25 PE=1 SV=1	sp|O00506|STK25_HUMAN	7	2	0.9727	0.9723

1488	26S proteasome non-ATPase regulatory subunit 14 OS=Homo sapiens GN=PSMD14 PE=1 SV=1	sp|O00487|PSDE_HUMAN	10	2	1.0471	0.9171
1489	Immediate early response 3-interacting protein 1 OS=Homo sapiens GN=IER3IP1 PE=1	sp|Q9Y5U9|IR3IP_HUMAN	40.2	2	1.0375	0.8565
1490	Mitotic spindle-associated MMXD complex subunit MIP18 OS=Homo sapiens GN=FAM96B PE=1 SV=1	sp|Q9Y3D0|MIP18_HUMAN	30.1	2	1.0375	0.5775
1491	Malignant T-cell-amplified sequence 1 OS=Homo sapiens GN=MCTS1 PE=1	sp|Q9ULC4|MCTS1_HUMAN	22.1	2	1.0765	0.861
1492	V-type proton ATPase subunit H OS=Homo sapiens GN=ATP6V1H PE=1 SV=1	sp|Q9UI12|VATH_HUMAN	9.3	2	1.5704	0.658
1493	39S ribosomal protein L15, mitochondrial OS=Homo sapiens GN=MRPL15 PE=1 SV=1	sp|Q9P015|RM15_HUMAN	10.1	2	0.8872	0.8233
1494	Mitochondrial import receptor subunit TOM22 homolog OS=Homo sapiens GN=TOMM22 PE=1 SV=3	sp|Q9NS69|TOM22_HUMAN	28.9	2	1.0093	0.9665
1495	Pleckstrin homology domain-containing family A member 2 OS=Homo sapiens GN=PLEKHA2	sp|Q9HB19|PKHA2_HUMAN	8.2	2	1.0864	0.8203
1496	Nuclear ubiquitous casein and cyclin-dependent kinase substrate 1 OS=Homo sapiens GN=NUCKS1 PE=1 SV=1	sp|Q9H1E3|NUCKS_HUMAN	9.9	2	0.879	0.7711
1497	THUMP domain-containing protein 3 OS=Homo sapiens GN=THUMPD3	sp|Q9BV44|THUM3_HUMAN	4.9	2	1.0765	0.7729
1498	Protein syndesmos OS=Homo sapiens GN=NUDT16L1 PE=1 SV=1	sp|Q9BRJ7|SDOS_HUMAN	12.8	2	0.6546	0.5445
1499	RNA-binding protein Musashi homolog 2 OS=Homo sapiens GN=MSI2 PE=1	sp|Q96DH6|MSI2H_HUMAN	11.6	2	1.0965	0.8708
1500	Protein tyrosine phosphatase type IVA 1 OS=Homo sapiens GN=PTP4A1 PE=1 SV=2	sp|Q93096|TP4A1_HUMAN	19.1	2	1.5996	0.4564
1501	Estradiol 17-beta-dehydrogenase 8 OS=Homo sapiens GN=HSD17B8 PE=1 SV=2	sp|Q92506|DHB8_HUMAN	14.6	2	1.0765	0.8564

1502	RNA polymerase II-associated factor 1 homolog OS=Homo sapiens GN=PAF1 PE=1 SV=2	sp|Q8N7H5|PAF1_HUMAN	6.4	2	0.9638	0.9388
1503	Iron-sulfur cluster assembly 2 homolog, mitochondrial OS=Homo sapiens GN=ISCA2 PE=1 SV=2	sp|Q86U28|ISCA2_HUMAN	23.4	2	1.1912	0.6822
1504	Glutaredoxin-related protein 5, mitochondrial OS=Homo sapiens GN=GLRX5 PE=1 SV=2	sp|Q86SX6|GLRX5_HUMAN	23.6	2	1.3552	0.5633
1505	Glycosyltransferase 8 domain-containing protein 1 OS=Homo sapiens GN=GLT8D1	sp|Q68CQ7|GL8D1_HUMAN	7.8	2	1.0765	0.8633
1506	Putative mitochondrial import inner membrane translocase subunit Tim23B OS=Homo sapiens GN=TIMM23B PE=5 SV=2	sp|Q5SRD1|TI23B_HUMAN	13.2	2	0.871	0.6758
1507	Microtubule-associated protein RP/EB family member 2 OS=Homo sapiens GN=MAPRE2 PE=1 SV=1	sp|Q15555|MARE2_HUMAN	10.7	2	1.0093	0.9998
1508	Prostaglandin E synthase 3 OS=Homo sapiens GN=PTGES3 PE=1 SV=1	sp|Q15185|TEBP_HUMAN	15	2	0.9376	0.889
1509	UDP-glucose 4-epimerase OS=Homo sapiens GN=GALE PE=1 SV=2	sp|Q14376|GALE_HUMAN	8.3	2	1.2023	0.6913
1510	Vesicular integral-membrane protein VIP36 OS=Homo sapiens GN=LMAN2 PE=1 SV=1	sp|Q12907|LMAN2_HUMAN	16	3	1.6144	0.4821
1511	Splicing factor 3A subunit 3 OS=Homo sapiens GN=SF3A3 PE=1	sp|Q12874|SF3A3_HUMAN	10	2	0.955	0.9228
1512	Hemoglobin subunit alpha OS=Homo sapiens GN=HBA1 PE=1	sp|P69905|HBA_HUMAN	16.9	2	1.8365	0.424
1513	Ras-related protein R-Ras2 OS=Homo sapiens GN=RRAS2 PE=1	sp|P62070|RRAS2_HUMAN	14.7	2	1.0666	0.8852
1514	Adenosine kinase OS=Homo sapiens GN=ADK PE=1 SV=2	sp|P55263|ADK_HUMAN	6.6	4	1.1695	0.7459
1515	Transcription initiation factor IIA subunit 1 OS=Homo sapiens GN=GTF2A1 PE=1 SV=1	sp|P52655|TF2AA_HUMAN	8.5	2	1.0471	0.8697

1516	ATP synthase subunit O, mitochondrial OS=Homo sapiens GN=ATP5O PE=1 SV=1	sp|P48047|ATPO_HUMAN	23.5	11	1.0471	0.9135
1517	DNA-directed RNA polymerases I, II, and III subunit RPABC1 OS=Homo sapiens GN=POLR2E PE=1 SV=4	sp|P19388|RPAB1_HUMAN	16.7	2	0.871	0.6296
1518	Electron transfer flavoprotein subunit alpha, mitochondrial OS=Homo sapiens GN=ETFA PE=1	sp|P13804|ETFA_HUMAN	15	4	1.1482	0.6739
1519	Erlin-2 OS=Homo sapiens GN=ERLIN2 PE=1 SV=1	sp|O94905|ERLN2_HUMAN	9.4	2	1.6293	0.6354
1520	Src kinase-associated phosphoprotein 2 OS=Homo sapiens GN=SKAP2 PE=1 SV=1	sp|O75563|SKAP2_HUMAN	10.9	2	1.2023	0.6251
1521	V-type proton ATPase subunit G 1 OS=Homo sapiens GN=ATP6V1G1	sp|O75348|VATG1_HUMAN	22.9	2	1.406	0.537
1522	DnaJ homolog subfamily B member 6 OS=Homo sapiens GN=DNAJB6 PE=1 SV=2	sp|O75190|DNJB6_HUMAN	7.7	2	1.2474	0.6568
1523	Pre-mRNA-processing factor 17 OS=Homo sapiens GN=CDC40 PE=1	sp|O60508|PRP17_HUMAN	6	2	0.9908	0.9343
1524	Ubiquitin domain-containing protein UBFD1 OS=Homo sapiens GN=UBFD1 PE=1	sp|O14562|UBFD1_HUMAN	11	2	0.955	0.9228
1525	26S proteasome non-ATPase regulatory subunit 9 OS=Homo sapiens GN=PSMD9 PE=1	sp|O00233|PSMD9_HUMAN	14.4	2	1	0.9623
1526	MORF4 family-associated protein 1 OS=Homo sapiens GN=MRFAP1 PE=1 SV=1	sp|Q9Y605|MOFA1_HUMAN	17.3	3	0.5012	0.3338
1527	Zinc finger protein 330 OS=Homo sapiens GN=ZNF330 PE=1 SV=1	sp|Q9Y3S2|ZN330_HUMAN	5.9	2	1	0.9793
1528	Mitochondrial fission 1 protein OS=Homo sapiens GN=FIS1 PE=1	sp|Q9Y3D6|FIS1_HUMAN	15.1	2	1	0.8425
1529	Charged multivesicular body protein 5 OS=Homo sapiens GN=CHMP5 PE=1 SV=1	sp|Q9NZZ3|CHMP5_HUMAN	14.6	2	1.0375	0.8518
1530	OCIA domain-containing protein 1 OS=Homo sapiens GN=OCIAD1	sp|Q9NX40|OCAD1_HUMAN	10.2	2	0.7244	0.6431

1531	MKI67 FHA domain-interacting nucleolar phosphoprotein OS=Homo sapiens GN=NIFK PE=1 SV=1	sp|Q9BYG3|MK67I_HUMAN	10.9	4		
1532	Inosine triphosphate pyrophosphatase OS=Homo sapiens GN=ITPA PE=1	sp|Q9BY32|ITPA_HUMAN	20.1	2	0.879	0.804
1533	Vacuolar protein-sorting-associated protein 25 OS=Homo sapiens GN=VPS25 PE=1 SV=1	sp|Q9BRG1|VPS25_HUMAN	12.5	2	0.929	0.8438
1534	Methylthioribulose-1-phosphate dehydratase OS=Homo sapiens GN=APIP PE=1 SV=1	sp|Q96GX9|MTNB_HUMAN	13.2	2	1.0471	0.9031
1535	Riboflavin kinase OS=Homo sapiens GN=RFK PE=1 SV=2	sp|Q969G6|RIFK_HUMAN	21.3	2	0.9817	0.9664
1536	Protein NDRG1 OS=Homo sapiens GN=NDRG1 PE=1 SV=1	sp|Q92597|NDRG1_HUMAN	8.1	4	15.1356	0.3485
1537	Laccase domain-containing protein 1 OS=Homo sapiens GN=LACC1 PE=2	sp|Q8IV20|LACC1_HUMAN	4	2	1.1695	0.7333
1538	Acylglycerol kinase, mitochondrial OS=Homo sapiens GN=AGK PE=1	sp|Q53H12|AGK_HUMAN	5.5	2	1.1588	0.755
1539	Cleavage and polyadenylation specificity factor subunit 6 OS=Homo sapiens GN=CPSF6 PE=1	sp|Q16630|CPSF6_HUMAN	7.4	7	1.1066	0.8199
1540	Transmembrane emp24 domain-containing protein 2 OS=Homo sapiens GN=TMED2 PE=1	sp|Q15363|TMED2_HUMAN	14.4	3	1.1695	0.4934
1541	Calcium/calmodulin-dependent protein kinase type 1 OS=Homo sapiens GN=CAMK1 PE=1	sp|Q14012|KCC1A_HUMAN	5.4	2	1.1912	0.68
1542	SAP domain-containing ribonucleoprotein OS=Homo sapiens GN=SARNP PE=1 SV=3	sp|P82979|SARNP_HUMAN	9	2	1.0375	0.9708
1543	Ubiquitin-conjugating enzyme E2 D2 OS=Homo sapiens GN=UBE2D2 PE=1 SV=1	sp|P62837|UB2D2_HUMAN	28.6	5	0.9638	0.9766
1544	Small nuclear ribonucleoprotein E OS=Homo sapiens GN=SNRPE PE=1 SV=1	sp|P62304|RUXE_HUMAN	25	2	0.912	0.8363
1545	Serine/threonine-protein phosphatase 4 catalytic subunit OS=Homo sapiens GN=PPP4C PE=1 SV=1	sp|P60510|PP4C_HUMAN	12.4	3	0.9817	0.7611

1546	Cyclin-H OS=Homo sapiens GN=CCNH PE=1	sp|P51946|CCNH_HUMAN	6.8	2	0.5346	0.6728
1547	Signal recognition particle 9 kDa protein OS=Homo sapiens GN=SRP9 PE=1 SV=2	sp|P49458|SRP09_HUMAN	20.9	2	0.8017	0.6859
1548	Signal recognition particle 14 kDa protein OS=Homo sapiens GN=SRP14 PE=1 SV=2	sp|P37108|SRP14_HUMAN	17.7	2	0.7447	0.8356
1549	Diphosphoinositol polyphosphate phosphohydrolase 1 OS=Homo sapiens GN=NUDT3 PE=1 SV=1	sp|O95989|NUDT3_HUMAN	14	2	1.0375	0.823
1550	Nucleoplasmin-3 OS=Homo sapiens GN=NPM3 PE=1 SV=3	sp|O75607|NPM3_HUMAN	23.6	2	1.0568	0.894
1551	Phosphatidylinositol 3-kinase regulatory subunit beta OS=Homo sapiens GN=PIK3R2 PE=1	sp|O00459|P85B_HUMAN	5.1	2	0.9462	0.81
1552	Mitochondrial Rho GTPase 1 OS=Homo sapiens GN=RHOT1 PE=1 SV=2	sp|Q8IXI2|MIRO1_HUMAN	7.6	3	1.0186	0.7893
1553	Malate dehydrogenase, cytoplasmic OS=Homo sapiens GN=MDH1 PE=1 SV=4	sp|P40925|MDHC_HUMAN	13.5	2	1.2246	0.688
1554	Recombining binding protein suppressor of hairless OS=Homo sapiens GN=RBPJ PE=1 SV=3	sp|Q06330|SUH_HUMAN	13	3	1.2023	0.8158
1555	SWI/SNF-related matrix-associated actin-dependent regulator of chromatin subfamily D member 1 OS=Homo sapiens GN=SMARCD1 PE=1 SV=2	sp|Q96GM5|SMRD1_HUMAN	9.7	4	0.8318	0.5577
1556	Sperm-associated antigen 7 OS=Homo sapiens GN=SPAG7 PE=1	sp|O75391|SPAG7_HUMAN	21.2	5	0.7178	0.5814
1557	Trafficking protein particle complex subunit 1 OS=Homo sapiens GN=TRAPPC1 PE=1 SV=1	sp|Q9Y5R8|TPPC1_HUMAN	20	2	0.929	0.9114
1558	Polymerase delta-interacting protein 3 OS=Homo sapiens GN=POLDIP3	sp|Q9BY77|PDIP3_HUMAN	12.6	2	0.7943	0.6981
1559	Son of sevenless homolog 1 OS=Homo sapiens GN=SOS1 PE=1 SV=1	sp|Q07889|SOS1_HUMAN	4.7	3	2.0893	0.7583
1560	HCLS1-associated protein X-1 OS=Homo sapiens GN=HAX1 PE=1 SV=2	sp|O00165|HAX1_HUMAN	5.7	2	0.9462	0.9081

1561	Hydroxyacyl-coenzyme A dehydrogenase, mitochondrial OS=Homo sapiens GN=HADH PE=1 SV=3	sp|Q16836|HCDH_HUMAN	19.1	3	0.9204	0.8364
1562	Anaphase-promoting complex subunit 1 OS=Homo sapiens GN=ANAPC1 PE=1 SV=1	sp|Q9H1A4|APC1_HUMAN	5	3	0.8954	0.8191
1563	Elongation factor G, mitochondrial OS=Homo sapiens GN=GFM1 PE=1 SV=2	sp|Q96RP9|EFGM_HUMAN	6.3	2	0.6026	0.5972
1564	Activator of 90 kDa heat shock protein ATPase homolog 1 OS=Homo sapiens GN=AHSA1 PE=1	sp|O95433|AHSA1_HUMAN	18.3	3	0.929	0.8841
1565	Ras-related C3 botulinum toxin substrate 2 OS=Homo sapiens GN=RAC2 PE=1 SV=1	sp|P15153|RAC2_HUMAN	45.8	8	1.0186	0.7354
1566	Coiled-coil domain-containing protein 88B OS=Homo sapiens GN=CCDC88B PE=1 SV=1	sp|A6NC98|CC88B_HUMAN	9.6	5	0.912	0.8754
1567	Flotillin-2 OS=Homo sapiens GN=FLOT2 PE=1	sp|Q14254|FLOT2_HUMAN	7.5	2	1.6144	0.4697
1568	Ezrin OS=Homo sapiens GN=EZR PE=1 SV=4	sp|P15311|EZRI_HUMAN	18.4	6	0.9817	0.7985
1569	60S ribosomal protein L22 OS=Homo sapiens GN=RPL22 PE=1 SV=2	sp|P35268|RL22_HUMAN	35.9	3	1.2134	0.4699
1570	Protein furry homolog-like OS=Homo sapiens GN=FRYL PE=1 SV=2	sp|O94915|FRYL_HUMAN	3.7	3	0.9638	0.9111
1571	ATP-dependent Clp protease proteolytic subunit, mitochondrial OS=Homo sapiens GN=CLPP PE=1 SV=1	sp|Q16740|CLPP_HUMAN	8.3	4	1.0568	0.8099
1572	Transcription elongation factor SPT5 OS=Homo sapiens GN=SUPT5H PE=1 SV=1	sp|O00267|SPT5H_HUMAN	7.3	5	0.7379	0.7832
1573	UDP-N-acetylhexosamine pyrophosphorylase-like protein 1 OS=Homo sapiens GN=UAP1L1 PE=1 SV=2	sp|Q3KQV9|UAP1L_HUMAN	16.6	4	1.3932	0.5358
1574	NEDD8 ultimate buster 1 OS=Homo sapiens GN=NUB1 PE=1 SV=2	sp|Q9Y5A7|NUB1_HUMAN	14.3	3	2.1086	0.6411
1575	Mitogen-activated protein kinase kinase kinase kinase 1 OS=Homo sapiens GN=MAP4K1	sp|Q92918|M4K1_HUMAN	8.4	4	0.8395	0.6875

1576	Ran-binding protein 10 OS=Homo sapiens GN=RANBP10 PE=1 SV=1	sp|Q6VN20|RBP10_HUMAN	13.9	3		
1577	Neutrophil cytosol factor 1 OS=Homo sapiens GN=NCF1 PE=1 SV=3	sp|P14598|NCF1_HUMAN	19.2	4	0.9817	0.9962
1578	Protein HGH1 homolog OS=Homo sapiens GN=HGH1 PE=1	sp|Q9BTY7|HGH1_HUMAN	6.4	2	1.028	0.9266
1579	NEDD8-conjugating enzyme Ubc12 OS=Homo sapiens GN=UBE2M PE=1	sp|P61081|UBC12_HUMAN	19.1	2	1.0186	0.969
1580	Phosphatidylinositol 5-phosphate 4-kinase type-2 alpha OS=Homo sapiens GN=PIP4K2A PE=1 SV=2	sp|P48426|PI42A_HUMAN	9.4	2	1.028	0.8827
1581	Protein TASOR OS=Homo sapiens GN=FAM208A PE=1 SV=3	sp|Q9UK61|TASOR_HUMAN	2.8	2	0.863	0.6727
1582	von Willebrand factor A domain-containing protein 9 OS=Homo sapiens GN=VWA9 PE=1	sp|Q96SY0|VWA9_HUMAN	10.4	2	0.7943	0.7415
1583	PRKR-interacting protein 1 OS=Homo sapiens GN=PRKRIP1 PE=1 SV=1	sp|Q9H875|PKRI1_HUMAN	10.3	2	0.7178	0.5107
1584	RAC-beta serine/threonine-protein kinase OS=Homo sapiens GN=AKT2 PE=1 SV=2	sp|P31751|AKT2_HUMAN	10.2	2	0.9462	0.8469
1585	Heterogeneous nuclear ribonucleoprotein F OS=Homo sapiens GN=HNRNPF PE=1 SV=3	sp|P52597|HNRPF_HUMAN	29.6	14	0.7447	0.5176
1586	Rho GTPase-activating protein 25 OS=Homo sapiens GN=ARHGAP25	sp|P42331|RHG25_HUMAN	13.3	5	1.3552	0.6243
1587	Succinyl-CoA ligase [ADP-forming] subunit beta, mitochondrial OS=Homo sapiens GN=SUCLA2 PE=1 SV=3	sp|Q9P2R7|SUCB1_HUMAN	5.6	2	1.0864	0.8477
1588	TATA box-binding protein-like protein 2 OS=Homo sapiens GN=TBPL2 PE=2	sp|Q6SJ96|TBPL2_HUMAN	8.5	2	0.9638	0.8398
1589	Coatomer subunit zeta-1 OS=Homo sapiens GN=COPZ1 PE=1	sp|P61923|COPZ1_HUMAN	14.1	2		
1590	Thyroid receptor-interacting protein 11 OS=Homo sapiens GN=TRIP11 PE=1	sp|Q15643|TRIPB_HUMAN	5.9	4	1.028	0.8524
1591	IST1 homolog OS=Homo sapiens GN=IST1 PE=1 SV=1	sp|P53990|IST1_HUMAN	9.9	2	1.1588	0.6554

1592	Splicing factor 3A subunit 2 OS=Homo sapiens GN=SF3A2 PE=1	sp|Q15428|SF3A2_HUMAN	13.4	5	0.787	0.7986
1593	Nuclear pore complex protein Nup133 OS=Homo sapiens GN=NUP133 PE=1 SV=2	sp|Q8WUM0|NU133_HUMAN	9.8	6	0.9727	0.9477
1594	Lipoamide acyltransferase component of branched-chain alpha-keto acid dehydrogenase complex, mitochondrial OS=Homo sapiens GN=DBT PE=1 SV=3	sp|P11182|ODB2_HUMAN	10.6	2	1.2706	0.6038
1595	FAST kinase domain-containing protein 5 OS=Homo sapiens GN=FASTKD5 PE=1 SV=1	sp|Q7L8L6|FAKD5_HUMAN	6.5	3	0.8318	0.7183
1596	ATPase family AAA domain-containing protein 2 OS=Homo sapiens GN=ATAD2 PE=1	sp|Q6PL18|ATAD2_HUMAN	3.7	2	0.8551	0.6697
1597	U6 snRNA-associated Sm-like protein LSm5 OS=Homo sapiens GN=LSM5 PE=1 SV=3	sp|Q9Y4Y9|LSM5_HUMAN	46.2	24		
1598	Serine/arginine-rich splicing factor 5 OS=Homo sapiens GN=SRSF5 PE=1 SV=1	sp|Q13243|SRSF5_HUMAN	18.4	3	0.863	0.7853
1599	Cellular nucleic acid-binding protein OS=Homo sapiens GN=CNBP PE=1 SV=1	sp|P62633|CNBP_HUMAN	28.8	4	0.5395	0.3248
1600	Sperm-specific antigen 2 OS=Homo sapiens GN=SSFA2 PE=1	sp|P28290|SSFA2_HUMAN	4.8	2	0.9817	0.9359
1601	Cell differentiation protein RCD1 homolog OS=Homo sapiens GN=RQCD1 PE=1	sp|Q92600|RCD1_HUMAN	12	2	0.6252	0.4677
1602	Heterogeneous nuclear ribonucleoprotein C-like 2 OS=Homo sapiens GN=HNRNPCL2 PE=2 SV=1	sp|B2RXH8|HNRC2_HUMAN	18.8	6	0.8954	0.7857
1603	Protein sel-1 homolog 1 OS=Homo sapiens GN=SEL1L PE=1 SV=3	sp|Q9UBV2|SE1L1_HUMAN	6.9	2	1.0186	0.8578
1604	ATP-dependent RNA helicase SUPV3L1, mitochondrial OS=Homo sapiens GN=SUPV3L1 PE=1 SV=1	sp|Q8IYB8|SUV3_HUMAN	10.3	4	0.9908	0.9861
1605	Nuclear mitotic apparatus protein 1 OS=Homo sapiens GN=NUMA1 PE=1 SV=2	sp|Q14980|NUMA1_HUMAN	11.4	6	0.7447	0.3542

1606	Protein canopy homolog 2 OS=Homo sapiens GN=CNPY2 PE=1 SV=1	sp|Q9Y2B0|CNPY2_HUMAN	15.9	2	0.9638	0.9496
1607	Midasin OS=Homo sapiens GN=MDN1 PE=1 SV=2	sp|Q9NU22|MDN1_HUMAN	4.9	4	0.9908	0.9847
1608	Peroxisomal acyl-coenzyme A oxidase 1 OS=Homo sapiens GN=ACOX1 PE=1 SV=3	sp|Q15067|ACOX1_HUMAN	10.5	3	1.0965	0.827
1609	Serine/threonine-protein phosphatase 2A 56 kDa regulatory subunit epsilon isoform OS=Homo sapiens GN=PPP2R5E PE=1 SV=1	sp|Q16537|2A5E_HUMAN	9.8	2	0.879	0.7796
1610	Endophilin-A2 OS=Homo sapiens GN=SH3GL1 PE=1 SV=1	sp|Q99961|SH3G1_HUMAN	9	2	1.1066	0.663
1611	GTP-binding protein Rheb OS=Homo sapiens GN=RHEB PE=1	sp|Q15382|RHEB_HUMAN	12.5	2	0.9727	0.9567
1612	Adipocyte plasma membrane-associated protein OS=Homo sapiens GN=APMAP PE=1 SV=2	sp|Q9HDC9|APMAP_HUMAN	10.3	7		
1613	Apoptosis-inducing factor 1, mitochondrial OS=Homo sapiens GN=AIFM1 PE=1 SV=1	sp|O95831|AIFM1_HUMAN	20.1	5	1.1169	0.7799
1614	Sterol O-acyltransferase 1 OS=Homo sapiens GN=SOAT1 PE=1 SV=3	sp|P35610|SOAT1_HUMAN	8.9	2	1.2246	0.7554
1615	Ribonuclease H2 subunit A OS=Homo sapiens GN=RNASEH2A PE=1 SV=2	sp|O75792|RNH2A_HUMAN	11.4	2	0.8166	0.7061
1616	Protein Red OS=Homo sapiens GN=IK PE=1	sp|Q13123|RED_HUMAN	7.4	2	0.5346	0.5215
1617	SWI/SNF complex subunit SMARCC1 OS=Homo sapiens GN=SMARCC1 PE=1 SV=3	sp|Q92922|SMRC1_HUMAN	5.9	3	0.787	0.6669
1618	Membrane magnesium transporter 1 OS=Homo sapiens GN=MMGT1 PE=1 SV=1	sp|Q8N4V1|MMGT1_HUMAN	15.3	2	0.912	0.8314
1619	Isopentenyl-diphosphate Delta-isomerase 1 OS=Homo sapiens GN=IDI1 PE=1 SV=2	sp|Q13907|IDI1_HUMAN	20.7	2	1.1376	0.7433
1620	Guanine nucleotide exchange factor MSS4 OS=Homo sapiens GN=RABIF PE=1	sp|P47224|MSS4_HUMAN	16.3	2	0.9817	0.9659

1621	Mitochondrial import inner membrane translocase subunit Tim8 A OS=Homo sapiens GN=TIMM8A PE=1 SV=1	sp|O60220|TIM8A_HUMAN	26.8	2	0.9908	0.999
1622	HEAT repeat-containing protein 6 OS=Homo sapiens GN=HEATR6	sp|Q6AI08|HEAT6_HUMAN	4.7	2	0.9204	0.8764
1623	Dedicator of cytokinesis protein 10 OS=Homo sapiens GN=DOCK10 PE=1 SV=3	sp|Q96BY6|DOC10_HUMAN	4.6	5	1.3305	0.5867
1624	Exocyst complex component 1 OS=Homo sapiens GN=EXOC1 PE=1 SV=4	sp|Q9NV70|EXOC1_HUMAN	8.2	2	1.028	0.7849
1625	Zinc finger CCCH domain-containing protein 14 OS=Homo sapiens GN=ZC3H14 PE=1 SV=1	sp|Q6PJT7|ZC3HE_HUMAN	8.2	4	1.0568	0.8783
1626	Epsin-1 OS=Homo sapiens GN=EPN1 PE=1 SV=2	sp|Q9Y6I3|EPN1_HUMAN	5	2	0.9908	0.9968
1627	Sialidase-1 OS=Homo sapiens GN=NEU1 PE=1 SV=1	sp|Q99519|NEUR1_HUMAN	14	17	1.1272	0.7805
1628	Activity-dependent neuroprotector homeobox protein OS=Homo sapiens GN=ADNP PE=1 SV=1	sp|Q9H2P0|ADNP_HUMAN	6.3	3	0.8241	0.7018
1629	Pre-mRNA-processing factor 19 OS=Homo sapiens GN=PRPF19 PE=1	sp|Q9UMS4|PRP19_HUMAN	16.3	3	1.3932	0.5459
1630	NAD kinase 2, mitochondrial OS=Homo sapiens GN=NADK2 PE=1 SV=2	sp|Q4G0N4|NAKD2_HUMAN	11.5	2	1.2023	0.713
1631	OTU domain-containing protein 6B OS=Homo sapiens GN=OTUD6B PE=1 SV=1	sp|Q8N6M0|OTU6B_HUMAN	11.6	2	0.9727	0.9384
1632	Switch-associated protein 70 OS=Homo sapiens GN=SWAP70 PE=1 SV=1	sp|Q9UH65|SWP70_HUMAN	7.7	3	0.9727	0.8773
1633	Protein argonaute-1 OS=Homo sapiens GN=AGO1 PE=1 SV=3	sp|Q9UL18|AGO1_HUMAN	7.4	2	0.879	0.8249
1634	Serine/threonine-protein kinase A-Raf OS=Homo sapiens GN=ARAF PE=1 SV=2	sp|P10398|ARAF_HUMAN	8.1	3	1.1169	0.7031
1635	Pumilio homolog 2 OS=Homo sapiens GN=PUM2 PE=1 SV=2	sp|Q8TB72|PUM2_HUMAN	10.5	5	0.9727	0.9585
1636	MAGUK p55 subfamily member 6 OS=Homo sapiens GN=MPP6 PE=1 SV=2	sp|Q9NZW5|MPP6_HUMAN	12.8	4	1.1272	0.7672

1637	Signal transducing adapter molecule 2 OS=Homo sapiens GN=STAM2 PE=1 SV=1	sp|O75886|STAM2_HUMAN	9.7	3	1.1803	0.7331
1638	O-phosphoseryl-tRNA(Sec) selenium transferase OS=Homo sapiens GN=SEPSECS	sp|Q9HD40|SPCS_HUMAN	10.8	2	0.9727	0.9939
1639	WD repeat-containing protein 74 OS=Homo sapiens GN=WDR74 PE=1	sp|Q6RFH5|WDR74_HUMAN	12.2	2	0.929	0.8631
1640	Drebrin-like protein OS=Homo sapiens GN=DBNL PE=1 SV=1	sp|Q9UJU6|DBNL_HUMAN	6.3	2	1.0471	0.8486
1641	Fatty-acid amide hydrolase 1 OS=Homo sapiens GN=FAAH PE=1 SV=2	sp|O00519|FAAH1_HUMAN	14.7	6	0.955	0.9469
1642	Cyclin-G-associated kinase OS=Homo sapiens GN=GAK PE=1	sp|O14976|GAK_HUMAN	3.4	2	0.8241	0.7196
1643	Histone deacetylase 3 OS=Homo sapiens GN=HDAC3 PE=1	sp|O15379|HDAC3_HUMAN	9.1	2	0.8954	0.8104
1644	D-beta-hydroxybutyrate dehydrogenase, mitochondrial OS=Homo sapiens GN=BDH1 PE=1 SV=3	sp|Q02338|BDH_HUMAN	6.4	2	1.0864	0.7617
1645	m7GpppX diphosphatase OS=Homo sapiens GN=DCPS PE=1 SV=2	sp|Q96C86|DCPS_HUMAN	8.6	2	0.9462	0.8621
1646	Negative elongation factor C/D OS=Homo sapiens GN=NELFCD	sp|Q8IXH7|NELFD_HUMAN	9.3	2	0.912	0.8475
1647	RNA-binding motif protein, X chromosome OS=Homo sapiens GN=RBMX PE=1 SV=3	sp|P38159|RBMX_HUMAN	14.6	4	0.929	0.8909
1648	Retinol dehydrogenase 13 OS=Homo sapiens GN=RDH13 PE=1 SV=2	sp|Q8NBN7|RDH13_HUMAN	10.3	2	1.1695	0.749
1649	Nicalin OS=Homo sapiens GN=NCLN PE=1 SV=2	sp|Q969V3|NCLN_HUMAN	11.9	2	1.0375	0.9493
1650	Double-stranded RNA-binding protein Staufen homolog 1 OS=Homo sapiens GN=STAU1 PE=1	sp|O95793|STAU1_HUMAN	7.5	3	0.9204	0.8796
1651	Core histone macro-H2A.1 OS=Homo sapiens GN=H2AFY PE=1	sp|O75367|H2AY_HUMAN	24.7	2	0.9638	0.989
1652	Zinc finger MIZ domain-containing protein 1 OS=Homo sapiens GN=ZMIZ1 PE=1	sp|Q9ULJ6|ZMIZ1_HUMAN	2.8	3	0.9908	0.8768

1653	Actin-related protein 2/3 complex subunit 5-like protein OS=Homo sapiens GN=ARPC5L	sp|Q9BPX5|ARP5L_HUMAN	24.8	3	0.863	0.5891
1654	Disintegrin and metalloproteinase domain-containing protein 17 OS=Homo sapiens GN=ADAM17 PE=1 SV=1	sp|P78536|ADA17_HUMAN	6.4	2	0.9908	0.9777
1655	Vitronectin OS=Homo sapiens GN=VTN PE=1 SV=1	sp|P04004|VTNC_HUMAN	8.6	2	2.208	0.2963
1656	Histone-binding protein RBBP7 OS=Homo sapiens GN=RBBP7 PE=1 SV=1	sp|Q16576|RBBP7_HUMAN	10.4	2	0.3404	0.4283
1657	AP-2 complex subunit sigma OS=Homo sapiens GN=AP2S1 PE=1 SV=2	sp|P53680|AP2S1_HUMAN	15.5	2	1.0965	0.723
1658	Ribosome biogenesis protein BRX1 homolog OS=Homo sapiens GN=BRIX1 PE=1	sp|Q8TDN6|BRX1_HUMAN	12.8	2	0.9817	0.9494
1659	Golgin subfamily A member 5 OS=Homo sapiens GN=GOLGA5 PE=1 SV=3	sp|Q8TBA6|GOGA5_HUMAN	6.7	2	1.1169	0.8065
1660	Phosphofurin acidic cluster sorting protein 1 OS=Homo sapiens GN=PACS1 PE=1	sp|Q6VY07|PACS1_HUMAN	6.8	2	1.2823	0.7501
1661	Integrator complex subunit 1 OS=Homo sapiens GN=INTS1 PE=1	sp|Q8N201|INT1_HUMAN	6.3	5	0.9462	0.8902
1662	Peroxisomal multifunctional enzyme type 2 OS=Homo sapiens GN=HSD17B4 PE=1 SV=3	sp|P51659|DHB4_HUMAN	10.1	3	1.0864	0.8507
1663	Serine/threonine-protein kinase PAK 1 OS=Homo sapiens GN=PAK1 PE=1 SV=2	sp|Q13153|PAK1_HUMAN	21.1	8	0.9817	0.9721
1664	Chromobox protein homolog 5 OS=Homo sapiens GN=CBX5 PE=1 SV=1	sp|P45973|CBX5_HUMAN	20.9	3	0.7047	0.539
1665	Nucleoporin Nup37 OS=Homo sapiens GN=NUP37 PE=1 SV=1	sp|Q8NFH4|NUP37_HUMAN	12.6	3	0.8954	0.9453
1666	CD2 antigen cytoplasmic tail-binding protein 2 OS=Homo sapiens GN=CD2BP2 PE=1 SV=1	sp|O95400|CD2B2_HUMAN	12.9	2	1.1482	0.7182
1667	Mannose-1-phosphate guanyltransferase alpha OS=Homo sapiens GN=GMPPA PE=1 SV=1	sp|Q96IJ6|GMPPA_HUMAN	11	3	1.0864	0.7147
1668	Nitrogen permease regulator 3-like protein OS=Homo sapiens GN=NPRL3 PE=1	sp|Q12980|NPRL3_HUMAN	7	2	0.9204	0.4499

1669	Platelet-activating factor acetylhydrolase IB subunit gamma OS=Homo sapiens GN=PAFAH1B3 PE=1 SV=1	sp|Q15102|PA1B3_HUMAN	23.8	3	0.9908	0.95
1670	Chromodomain-helicase-DNA-binding protein 1-like OS=Homo sapiens GN=CHD1L PE=1	sp|Q86WJ1|CHD1L_HUMAN	7.1	3	0.8954	0.8318
1671	Exosome complex component RRP40 OS=Homo sapiens GN=EXOSC3 PE=1 SV=3	sp|Q9NQT5|EXOS3_HUMAN	15.6	2		
1672	Proteasome subunit beta type-7 OS=Homo sapiens GN=PSMB7 PE=1 SV=1	sp|Q99436|PSB7_HUMAN	27.4	4	0.8395	0.7454
1673	Ribosome-releasing factor 2, mitochondrial OS=Homo sapiens GN=GFM2 PE=1 SV=1	sp|Q969S9|RRF2M_HUMAN	8.7	3	0.9638	0.93
1674	E3 ubiquitin-protein ligase RNF31 OS=Homo sapiens GN=RNF31 PE=1	sp|Q96EP0|RNF31_HUMAN	5.4	2	1.0093	0.9744
1675	CTTNBP2 N-terminal-like protein OS=Homo sapiens GN=CTTNBP2NL	sp|Q9P2B4|CT2NL_HUMAN	5.9	1	1.2023	0.7126
1676	Nuclear pore membrane glycoprotein 210 OS=Homo sapiens GN=NUP210 PE=1 SV=3	sp|Q8TEM1|PO210_HUMAN	3.9	3	0.9204	0.7654
1677	Copine-3 OS=Homo sapiens GN=CPNE3 PE=1	sp|O75131|CPNE3_HUMAN	6.1	3		
1678	Signal recognition particle 19 kDa protein OS=Homo sapiens GN=SRP19 PE=1 SV=3	sp|P09132|SRP19_HUMAN	27.1	2	0.9727	0.9747
1679	Histone acetyltransferase KAT2A OS=Homo sapiens GN=KAT2A PE=1 SV=3	sp|Q92830|KAT2A_HUMAN	4.9	2	0.9376	0.8834
1680	Glycerol-3-phosphate acyltransferase 4 OS=Homo sapiens GN=AGPAT6 PE=1 SV=1	sp|Q86UL3|GPAT4_HUMAN	8.1	2	1	0.9792
1681	Nicotinamide phosphoribosyltransferase OS=Homo sapiens GN=NAMPT PE=1 SV=1	sp|P43490|NAMPT_HUMAN	10	4	1.1169	0.7796
1682	Nuclear receptor-binding protein OS=Homo sapiens GN=NRBP1 PE=1 SV=1	sp|Q9UHY1|NRBP_HUMAN	9.2	3	1.0666	0.9316
1683	Calcium-binding mitochondrial carrier protein Aralar2 OS=Homo sapiens GN=SLC25A13 PE=1 SV=2	sp|Q9UJS0|CMC2_HUMAN	16.9	10	1.0666	0.8752

1684	10 kDa heat shock protein, mitochondrial OS=Homo sapiens GN=HSPE1 PE=1 SV=2	sp|P61604|CH10_HUMAN	29.4	4	1.028	0.8802
1685	WW domain-containing oxidoreductase OS=Homo sapiens GN=WWOX PE=1 SV=1	sp|Q9NZC7|WWOX_HUMAN	14	2	0.9204	0.8806
1686	60S ribosomal protein L26 OS=Homo sapiens GN=RPL26 PE=1 SV=1	sp|P61254|RL26_HUMAN	22.1	3	1.0765	0.8432
1687	Lactoylglutathione lyase OS=Homo sapiens GN=GLO1 PE=1 SV=4	sp|Q04760|LGUL_HUMAN	21.7	2	1.0093	0.9944
1688	CD180 antigen OS=Homo sapiens GN=CD180 PE=1 SV=2	sp|Q99467|CD180_HUMAN	6.2	3	1.2246	0.6876
1689	LIM and senescent cell antigen-like-containing domain protein 1 OS=Homo sapiens GN=LIMS1 PE=1	sp|P48059|LIMS1_HUMAN	21.5	4	1.1169	0.2148
1690	Caspase recruitment domain-containing protein 9 OS=Homo sapiens	sp|Q9H257|CARD9_HUMAN	19.2	3	1.0186	0.9653
1691	Nuclear factor NF-kappa-B p105 subunit OS=Homo sapiens GN=NFKB1 PE=1	sp|P19838|NFKB1_HUMAN	3.6	2	0.9204	0.9209
1692	SHC-transforming protein 1 OS=Homo sapiens GN=SHC1 PE=1	sp|P29353|SHC1_HUMAN	8.4	3	1.0186	0.9126
1693	Sorting nexin-1 OS=Homo sapiens GN=SNX1 PE=1 SV=3	sp|Q13596|SNX1_HUMAN	12.6	5	0.7112	0.8992
1694	Tyrosine-protein phosphatase non-receptor type 1 OS=Homo sapiens GN=PTPN1 PE=1 SV=1	sp|P18031|PTN1_HUMAN	6.7	2	1.1695	0.7429
1695	RNA polymerase II-associated protein 1 OS=Homo sapiens GN=RPAP1 PE=1	sp|Q9BWH6|RPAP1_HUMAN	6.3	2	1.0666	0.7618
1696	ATPase family AAA domain-containing protein 1 OS=Homo sapiens GN=ATAD1 PE=1	sp|Q8NBU5|ATAD1_HUMAN	13	2	1.0093	0.9177
1697	Eukaryotic translation initiation factor 4E OS=Homo sapiens GN=EIF4E PE=1	sp|P06730|IF4E_HUMAN	17.1	2	0.9908	0.9582
1698	Transcriptional adapter 1 OS=Homo sapiens GN=TADA1 PE=1 SV=1	sp|Q96BN2|TADA1_HUMAN	11	2	1.2474	0.6627
1699	Aldehyde dehydrogenase family 3 member B1 OS=Homo sapiens GN=ALDH3B1 PE=1 SV=1	sp|P43353|AL3B1_HUMAN	8.1	1	1.3183	0.5954

1700	Coiled-coil domain-containing protein 6 OS=Homo sapiens GN=CCDC6 PE=1	sp|Q16204|CCDC6_HUMAN	13.7	2	0.929	0.8732
1701	BUB3-interacting and GLEBS motif-containing protein ZNF207 OS=Homo sapiens GN=ZNF207 PE=1 SV=1	sp|O43670|ZN207_HUMAN	9.4	2	0.8241	0.6894
1702	Serine/threonine-protein phosphatase PP1-alpha catalytic subunit OS=Homo sapiens GN=PPP1CA PE=1 SV=1	sp|P62136|PP1A_HUMAN	39.7	8	0.912	0.7562
1703	GTPase-activating protein and VPS9 domain-containing protein 1 OS=Homo sapiens GN=GAPVD1 PE=1 SV=2	sp|Q14C86|GAPD1_HUMAN	5	2		
1704	Prefoldin subunit 2 OS=Homo sapiens GN=PFDN2 PE=1 SV=1	sp|Q9UHV9|PFD2_HUMAN	29.2	2	0.9727	0.9644
1705	Pantothenate kinase 2, mitochondrial OS=Homo sapiens GN=PANK2 PE=1 SV=3	sp|Q9BZ23|PANK2_HUMAN	6.3	2	0.929	0.8733
1706	TBC1 domain family member 15 OS=Homo sapiens GN=TBC1D15 PE=1 SV=2	sp|Q8TC07|TBC15_HUMAN	7.7	2	1.0186	0.8801
1707	Myotubularin-related protein 3 OS=Homo sapiens GN=MTMR3 PE=1 SV=3	sp|Q13615|MTMR3_HUMAN	1.8	1	0.9727	0.9882
1708	Glycerol-3-phosphate dehydrogenase [NAD(+)], cytoplasmic OS=Homo sapiens GN=GPD1 PE=1 SV=4	sp|P21695|GPDA_HUMAN	10.3	3	1.4588	0.76
1709	Long-chain specific acyl-CoA dehydrogenase, mitochondrial OS=Homo sapiens GN=ACADL PE=1 SV=2	sp|P28330|ACADL_HUMAN	14.7	3	1.2246	0.6714
1710	60S ribosomal protein L36 OS=Homo sapiens GN=RPL36 PE=1 SV=3	sp|Q9Y3U8|RL36_HUMAN	25.7	1	0.6194	0.3003
1711	Arf-GAP domain and FG repeat-containing protein 1 OS=Homo sapiens GN=AGFG1 PE=1	sp|P52594|AGFG1_HUMAN	4.6	1	1.0666	0.8076
1712	Redox-regulatory protein FAM213A OS=Homo sapiens GN=FAM213A PE=1 SV=3	sp|Q9BRX8|F213A_HUMAN	8.7	1	1.028	0.7724
1713	Rho GDP-dissociation inhibitor 1 OS=Homo sapiens GN=ARHGDIA	sp|P52565|GDIR1_HUMAN	13.7	5	1.7865	0.8078

1714	BAG family molecular chaperone regulator 3 OS=Homo sapiens GN=BAG3 PE=1 SV=3	sp|O95817|BAG3_HUMAN	9.7	2	0.8954	0.8088
1715	Putative glycerol kinase 3 OS=Homo sapiens GN=GK3P PE=5 SV=2	sp|Q14409|GLPK3_HUMAN	11.9	3	1.2359	0.6753
1716	Double-strand-break repair protein rad21 homolog OS=Homo sapiens GN=RAD21 PE=1	sp|O60216|RAD21_HUMAN	3.3	2	0.631	0.7955
1717	U4/U6.U5 tri-snRNP-associated protein 1 OS=Homo sapiens GN=SART1 PE=1	sp|O43290|SNUT1_HUMAN	14.5	2	0.912	0.9154
1718	Reticulocalbin-2 OS=Homo sapiens GN=RCN2 PE=1 SV=1	sp|Q14257|RCN2_HUMAN	13.6	2	0.9727	0.9938
1719	Calcium homeostasis endoplasmic reticulum protein OS=Homo sapiens GN=CHERP PE=1 SV=3	sp|Q8IWX8|CHERP_HUMAN	8.3	2	0.9727	0.9189
1720	Disco-interacting protein 2 homolog B OS=Homo sapiens GN=DIP2B PE=1 SV=3	sp|Q9P265|DIP2B_HUMAN	3	1	0.9036	0.7747
1721	Peptidyl-prolyl cis-trans isomerase-like 4 OS=Homo sapiens GN=PPIL4 PE=1 SV=1	sp|Q8WUA2|PPIL4_HUMAN	7.3	2	0.8091	0.6882
1722	ATP synthase subunit e, mitochondrial OS=Homo sapiens GN=ATP5I PE=1 SV=2	sp|P56385|ATP5I_HUMAN	27.5	2	0.9376	0.913
1723	UV excision repair protein RAD23 homolog A OS=Homo sapiens GN=RAD23A PE=1 SV=1	sp|P54725|RD23A_HUMAN	19.8	2	1.0864	0.844
1724	pre-rRNA processing protein FTSJ3 OS=Homo sapiens GN=FTSJ3 PE=1 SV=2	sp|Q8IY81|SPB1_HUMAN	7.6	2	0.8166	0.6073
1725	SURP and G-patch domain-containing protein 1 OS=Homo sapiens GN=SUGP1 PE=1	sp|Q8IWZ8|SUGP1_HUMAN	10.7	3	0.5598	0.5539
1726	60S ribosomal protein L14 OS=Homo sapiens GN=RPL14 PE=1 SV=4	sp|P50914|RL14_HUMAN	15.8	5		
1727	DNA-directed RNA polymerases I and III subunit RPAC1 OS=Homo sapiens GN=POLR1C	sp|O15160|RPAC1_HUMAN	15.9	3	0.8872	0.8255
1728	NADH dehydrogenase [ubiquinone] iron-sulfur protein 4, mitochondrial OS=Homo sapiens GN=NDUFS4 PE=1 SV=1	sp|O43181|NDUS4_HUMAN	20	2	1.1272	0.7982

1729	Rho-associated protein kinase 1 OS=Homo sapiens GN=ROCK1 PE=1 SV=1	sp|Q13464|ROCK1_HUMAN	6.1	3	1.0186	0.8942
1730	Autophagy-related protein 2 homolog B OS=Homo sapiens GN=ATG2B PE=1	sp|Q96BY7|ATG2B_HUMAN	4.4	5	0.9817	0.9588
1731	Vesicle-associated membrane protein 4 OS=Homo sapiens GN=VAMP4 PE=1	sp|O75379|VAMP4_HUMAN	17.7	1	1.0186	0.9341
1732	60S ribosomal protein L35 OS=Homo sapiens GN=RPL35 PE=1 SV=2	sp|P42766|RL35_HUMAN	25.2	1	0.9908	0.9382
1733	Cytochrome b5 OS=Homo sapiens GN=CYB5A PE=1 SV=2	sp|P00167|CYB5_HUMAN	29.9	2	0.9817	0.9923
1734	RNA-binding protein with serine-rich domain 1 OS=Homo sapiens GN=RNPS1 PE=1	sp|Q15287|RNPS1_HUMAN	14.4	2	0.9204	0.5951
1735	Uncharacterized protein C9orf78 OS=Homo sapiens GN=C9orf78 PE=1 SV=1	sp|Q9NZ63|CI078_HUMAN	12.8	2	0.7656	0.6341
1736	Myotrophin OS=Homo sapiens GN=MTPN PE=1 SV=2	sp|P58546|MTPN_HUMAN	24.6	3		
1737	Putative phospholipase B-like 2 OS=Homo sapiens GN=PLBD2 PE=1	sp|Q8NHP8|PLBL2_HUMAN	6.3	3	2.1878	0.4213
1738	Oligoribonuclease, mitochondrial OS=Homo sapiens GN=REXO2 PE=1 SV=3	sp|Q9Y3B8|ORN_HUMAN	19.4	5	1.0765	0.8294
1739	Splicing factor 45 OS=Homo sapiens GN=RBM17 PE=1 SV=1	sp|Q96I25|SPF45_HUMAN	6.7	1	0.9638	0.9487
1740	Sterol-4-alpha-carboxylate 3-dehydrogenase, decarboxylating OS=Homo sapiens GN=NSDHL PE=1 SV=2	sp|Q15738|NSDHL_HUMAN	18.2	3	1.1912	0.7015
1741	Chromatin target of PRMT1 protein OS=Homo sapiens GN=CHTOP PE=1 SV=2	sp|Q9Y3Y2|CHTOP_HUMAN	13.7	1	0.9638	0.8744
1742	Prefoldin subunit 4 OS=Homo sapiens GN=PFDN4 PE=1 SV=1	sp|Q9NQP4|PFD4_HUMAN	17.9	2	1.0568	0.873
1743	Keratin, type I cytoskeletal 16 OS=Homo sapiens GN=KRT16 PE=1 SV=4	sp|P08779|K1C16_HUMAN	8	2	0.955	0.9451
1744	DNA-directed RNA polymerase II subunit RPB4 OS=Homo sapiens GN=POLR2D PE=1 SV=1	sp|O15514|RPB4_HUMAN	21.8	2	0.929	0.8891
1745	Metaxin-1 OS=Homo sapiens GN=MTX1 PE=1 SV=2	sp|Q13505|MTX1_HUMAN	11.2	2	0.9204	0.7311

1746	cTAGE family member 5 OS=Homo sapiens GN=CTAGE5 PE=1 SV=4	sp|O15320|CTGE5_HUMAN	6.6	1	1	0.8963
1747	Ataxin-2 OS=Homo sapiens GN=ATXN2 PE=1 SV=2	sp|Q99700|ATX2_HUMAN	4.4	1	0.8954	0.8338
1748	Glyoxalase domain-containing protein 4 OS=Homo sapiens GN=GLOD4 PE=1	sp|Q9HC38|GLOD4_HUMAN	17.6	2	1.0864	0.6732
1749	39S ribosomal protein L47, mitochondrial OS=Homo sapiens GN=MRPL47 PE=1 SV=2	sp|Q9HD33|RM47_HUMAN	6.8	1	0.8166	0.6453
1750	Sodium bicarbonate cotransporter 3 OS=Homo sapiens GN=SLC4A7 PE=1	sp|Q9Y6M7|S4A7_HUMAN	4.9	3	1.2823	0.6428
1751	Serine/threonine-protein phosphatase 4 regulatory subunit 3B OS=Homo sapiens GN=SMEK2 PE=1	sp|Q5MIZ7|P4R3B_HUMAN	4.5	1	0.8017	0.7728
1752	Pyridoxal kinase OS=Homo sapiens GN=PDXK PE=1 SV=1	sp|O00764|PDXK_HUMAN	16	4	1.0186	0.9068
1753	Actin, cytoplasmic 1 OS=Homo sapiens GN=ACTB PE=1 SV=1	sp|P60709|ACTB_HUMAN	78.7	259	1.7865	0.078
1754	Regulation of nuclear pre-mRNA domain-containing protein 1A OS=Homo sapiens GN=RPRD1A PE=1 SV=1	sp|Q96P16|RPR1A_HUMAN	15.4	3	0.9036	0.8531
1755	ATP-dependent RNA helicase DHX8 OS=Homo sapiens GN=DHX8 PE=1 SV=1	sp|Q14562|DHX8_HUMAN	6.6	6	0.8472	0.7607
1756	Nuclear pore complex protein Nup50 OS=Homo sapiens GN=NUP50 PE=1	sp|Q9UKX7|NUP50_HUMAN	7.9	4	0.8166	0.6991
1757	Probable leucine--tRNA ligase, mitochondrial OS=Homo sapiens GN=LARS2 PE=1 SV=2	sp|Q15031|SYLM_HUMAN	5.6	2	1.1482	0.7695
1758	Caprin-1 OS=Homo sapiens GN=CAPRIN1 PE=1 SV=2	sp|Q14444|CAPR1_HUMAN	11.1	4	0.631	0.4565
1759	SUN domain-containing protein 2 OS=Homo sapiens GN=SUN2 PE=1 SV=3	sp|Q9UH99|SUN2_HUMAN	10.6	2	0.912	0.5778
1760	Protein zwilch homolog OS=Homo sapiens GN=ZWILCH PE=1 SV=2	sp|Q9H900|ZWILC_HUMAN	3.7	1	0.4875	0.3998
1761	Protein phosphatase 1B OS=Homo sapiens GN=PPM1B PE=1	sp|O75688|PPM1B_HUMAN	5.8	2	1.0093	0.6339

1762	N-acylglucosamine 2-epimerase OS=Homo sapiens GN=RENBP PE=1 SV=2	sp|P51606|RENBP_HUMAN	6.1	2	1.5704	0.4231
1763	Proline-serine-threonine phosphatase-interacting protein 2 OS=Homo sapiens GN=PSTPIP2	sp|Q9H939|PPIP2_HUMAN	10.5	2	1.5996	0.4338
1764	Integrator complex subunit 5 OS=Homo sapiens GN=INTS5 PE=1	sp|Q6P9B9|INT5_HUMAN	7.1	4	0.8872	0.8292
1765	E3 ubiquitin-protein ligase BRE1A OS=Homo sapiens GN=RNF20 PE=1	sp|Q5VTR2|BRE1A_HUMAN	7.9	2	0.8318	0.7292
1766	Ras-related protein Rab-11B OS=Homo sapiens GN=RAB11B PE=1 SV=4	sp|Q15907|RB11B_HUMAN	11	2	1.2023	0.7068
1767	Dihydrolipoyllysine-residue acetyltransferase component of pyruvate dehydrogenase complex, mitochondrial OS=Homo sapiens GN=DLAT PE=1 SV=3	sp|P10515|ODP2_HUMAN	13.8	3	1.0568	0.8999
1768	ADP-ribosylation factor-like protein 3 OS=Homo sapiens GN=ARL3 PE=1 SV=2	sp|P36405|ARL3_HUMAN	12.6	2	1.0666	0.8171
1769	Bromodomain-containing protein 2 OS=Homo sapiens GN=BRD2 PE=1 SV=2	sp|P25440|BRD2_HUMAN	4.1	3	0.929	0.8987
1770	ADP/ATP translocase 1 OS=Homo sapiens GN=SLC25A4 PE=1 SV=4	sp|P12235|ADT1_HUMAN	30.9	11	1.0765	0.8677
1771	Pumilio domain-containing protein KIAA0020 OS=Homo sapiens GN=KIAA0020 PE=1 SV=3	sp|Q15397|K0020_HUMAN	12.7	5	0.9204	0.8866
1772	Anamorsin OS=Homo sapiens GN=CIAPIN1 PE=1 SV=2	sp|Q6FI81|CPIN1_HUMAN	14.4	1	0.863	0.7143
1773	Oxygen-dependent coproporphyrinogen-III oxidase, mitochondrial OS=Homo sapiens GN=CPOX PE=1 SV=3	sp|P36551|HEM6_HUMAN	7.7	1	0.7379	0.6041
1774	Homeobox protein cut-like 1 OS=Homo sapiens GN=CUX1 PE=1	sp|P39880|CUX1_HUMAN	6.4	2	0.9727	0.9059
1775	RNA-binding protein EWS OS=Homo sapiens GN=EWSR1 PE=1	sp|Q01844|EWS_HUMAN	14.8	5	0.8472	0.7634
1776	Syntaxin-6 OS=Homo sapiens GN=STX6 PE=1 SV=1	sp|O43752|STX6_HUMAN	9.8	3	0.9727	0.9752

1777	Probable rRNA-processing protein EBP2 OS=Homo sapiens GN=EBNA1BP2 PE=1 SV=2	sp|Q99848|EBP2_HUMAN	15.4	1	0.9817	0.9603
1778	NADH dehydrogenase [ubiquinone] 1 alpha subcomplex subunit 13 OS=Homo sapiens GN=NDUFA13 PE=1 SV=3	sp|Q9P0J0|NDUAD_HUMAN	16.7	2	0.9727	0.9731
1779	AMP deaminase 3 OS=Homo sapiens GN=AMPD3 PE=1 SV=1	sp|Q01432|AMPD3_HUMAN	3.9	2	1.1376	0.6816
1780	Collagen type IV alpha-3-binding protein OS=Homo sapiens GN=COL4A3BP PE=1 SV=1	sp|Q9Y5P4|C43BP_HUMAN	5.6	1	0.6918	0.7374
1781	Glycerol-3-phosphate acyltransferase 1, mitochondrial OS=Homo sapiens GN=GPAM PE=1 SV=3	sp|Q9HCL2|GPAT1_HUMAN	4	2	1.1588	0.7551
1782	Acyl-protein thioesterase 2 OS=Homo sapiens GN=LYPLA2 PE=1 SV=1	sp|O95372|LYPA2_HUMAN	17.3	2	0.955	0.9652
1783	Regulation of nuclear pre-mRNA domain-containing protein 2 OS=Homo sapiens GN=RPRD2 PE=1	sp|Q5VT52|RPRD2_HUMAN	2.7	3	0.9727	0.9775
1784	Carbonic anhydrase 2 OS=Homo sapiens GN=CA2 PE=1 SV=2	sp|P00918|CAH2_HUMAN	12.7	2	0.6855	0.482
1785	Ribosomal protein S6 kinase alpha-3 OS=Homo sapiens GN=RPS6KA3 PE=1 SV=1	sp|P51812|KS6A3_HUMAN	9.2	2	1.0765	0.7946
1786	Pachytene checkpoint protein 2 homolog OS=Homo sapiens GN=TRIP13 PE=1	sp|Q15645|PCH2_HUMAN	8.8	3	1	0.9904
1787	Protein phosphatase 1 regulatory subunit 12A OS=Homo sapiens GN=PPP1R12A	sp|O14974|MYPT1_HUMAN	6.7	3	0.929	0.9031
1788	Breast carcinoma-amplified sequence 3 OS=Homo sapiens GN=BCAS3 PE=1	sp|Q9H6U6|BCAS3_HUMAN	6.1	2	1.1695	0.7222
1789	Putative tRNA pseudouridine synthase Pus10 OS=Homo sapiens GN=PUS10 PE=1 SV=1	sp|Q3MIT2|PUS10_HUMAN	15.1	2	1.0186	0.9492
1790	Cofilin-2 OS=Homo sapiens GN=CFL2 PE=1	sp|Q9Y281|COF2_HUMAN	45.2	10	1.3804	0.3891
1791	PHD finger protein 3 OS=Homo sapiens GN=PHF3 PE=1 SV=3	sp|Q92576|PHF3_HUMAN	2.3	1	0.8872	0.8376

1792	PDZ domain-containing protein GIPC1 OS=Homo sapiens GN=GIPC1 PE=1 SV=2	sp|O14908|GIPC1_HUMAN	14.4	3	0.879	0.8351
1793	tRNA:m(4)X modification enzyme TRM13 homolog OS=Homo sapiens GN=TRMT13 PE=1 SV=2	sp|Q9NUP7|TRM13_HUMAN	5.4	2	0.863	0.7846
1794	UBX domain-containing protein 7 OS=Homo sapiens GN=UBXN7 PE=1	sp|O94888|UBXN7_HUMAN	6.3	1	1.0666	0.8678
1795	Replication factor C subunit 1 OS=Homo sapiens GN=RFC1 PE=1	sp|P35251|RFC1_HUMAN	4.7	3		
1796	FYVE, RhoGEF and PH domain-containing protein 3 OS=Homo sapiens GN=FGD3 PE=1 SV=1	sp|Q5JSP0|FGD3_HUMAN	7.6	2	1.1376	0.7786
1797	Histone H1.5 OS=Homo sapiens GN=HIST1H1B PE=1 SV=3	sp|P16401|H15_HUMAN	20.8	3	0.6427	0.4663
1798	DNA methyltransferase 1-associated protein 1 OS=Homo sapiens GN=DMAP1 PE=1	sp|Q9NPF5|DMAP1_HUMAN	12	1	0.5297	0.5073
1799	Cilia- and flagella-associated protein 20 OS=Homo sapiens GN=CFAP20 PE=1	sp|Q9Y6A4|CFA20_HUMAN	11.9	1	0.8472	0.7486
1800	Probable E3 ubiquitin-protein ligase HERC1 OS=Homo sapiens GN=HERC1 PE=1 SV=2	sp|Q15751|HERC1_HUMAN	3.9	5	1.4322	0.8655
1801	RNA-binding protein 25 OS=Homo sapiens GN=RBM25 PE=1	sp|P49756|RBM25_HUMAN	15.8	1	1	0.8466
1802	SLIT-ROBO Rho GTPase-activating protein 2 OS=Homo sapiens GN=SRGAP2 PE=1 SV=2	sp|O75044|SRGP2_HUMAN	5.7	2	0.9817	0.9884
1803	Guanine nucleotide-binding protein subunit alpha-13 OS=Homo sapiens GN=GNA13 PE=1	sp|Q14344|GNA13_HUMAN	11.4	2	1.1588	0.7652
1804	Thioredoxin OS=Homo sapiens GN=TXN PE=1 SV=3	sp|P10599|THIO_HUMAN	38.1	2	1.1066	0.8978
1805	Bifunctional methylenetetrahydrofolate dehydrogenase/cyclohydrolase, mitochondrial OS=Homo sapiens GN=MTHFD2 PE=1 SV=2	sp|P13995|MTDC_HUMAN	10.6	3	0.9204	0.9813
1806	Ras-related protein Rab-21 OS=Homo sapiens GN=RAB21 PE=1	sp|Q9UL25|RAB21_HUMAN	14.7	2	0.9908	0.9194

1807	Ras GTPase-activating protein-binding protein 2 OS=Homo sapiens GN=G3BP2 PE=1 SV=2	sp|Q9UN86|G3BP2_HUMAN	13.9	3	0.8954	0.8384
1808	Dystonin OS=Homo sapiens GN=DST PE=1	sp|Q03001|DYST_HUMAN	3.6	3	1.2023	0.6977
1809	E3 ubiquitin-protein ligase UHRF1 OS=Homo sapiens GN=UHRF1 PE=1	sp|Q96T88|UHRF1_HUMAN	5.8	1	0.5598	0.3632
1810	Inositol monophosphatase 1 OS=Homo sapiens GN=IMPA1 PE=1 SV=1	sp|P29218|IMPA1_HUMAN	13.4	2	1.2246	0.5362
1811	Phenylalanine--tRNA ligase alpha subunit OS=Homo sapiens GN=FARSA PE=1	sp|Q9Y285|SYFA_HUMAN	19.3	4	1.1588	0.726
1812	Integrator complex subunit 6 OS=Homo sapiens GN=INTS6 PE=1	sp|Q9UL03|INT6_HUMAN	5.6	1	0.8954	0.5579
1813	Aspartyl aminopeptidase OS=Homo sapiens GN=DNPEP PE=1 SV=1	sp|Q9ULA0|DNPEP_HUMAN	15.2	3	1.977	0.8472
1814	Vacuolar protein sorting-associated protein 53 homolog OS=Homo sapiens GN=VPS53 PE=1 SV=1	sp|Q5VIR6|VPS53_HUMAN	9	1	0.9204	0.7615
1815	PC4 and SFRS1-interacting protein OS=Homo sapiens GN=PSIP1 PE=1 SV=1	sp|O75475|PSIP1_HUMAN	11.7	2	0.7798	0.6464
1816	60S ribosomal protein L27a OS=Homo sapiens GN=RPL27A PE=1 SV=2	sp|P46776|RL27A_HUMAN	29.7	2	1.0186	0.5505
1817	Serine/arginine-rich splicing factor 7 OS=Homo sapiens GN=SRSF7 PE=1 SV=1	sp|Q16629|SRSF7_HUMAN	21.4	2	0.9204	0.8841
1818	Glucosidase 2 subunit beta OS=Homo sapiens GN=PRKCSH PE=1 SV=2	sp|P14314|GLU2B_HUMAN	16.3	2	1.1912	0.7174
1819	HEAT repeat-containing protein 3 OS=Homo sapiens GN=HEATR3	sp|Q7Z4Q2|HEAT3_HUMAN	9.6	3	0.787	0.6664
1820	Cilia- and flagella-associated protein 36 OS=Homo sapiens GN=CFAP36 PE=1	sp|Q96G28|CFA36_HUMAN	11.1	1	1.0093	0.9102
1821	Up-regulated during skeletal muscle growth protein 5 OS=Homo sapiens GN=USMG5 PE=1	sp|Q96IX5|USMG5_HUMAN	19	1	0.955	0.9378
1822	Protein transport protein Sec61 subunit beta OS=Homo sapiens GN=SEC61B PE=1 SV=2	sp|P60468|SC61B_HUMAN	18.8	1	0.929	0.8945

1823	REVERSED NACHT domain- and WD repeat-containing protein 1 OS=Homo sapiens GN=NWD1 PE=1	RRRRRsp|Q149M9|NWD1_HUMAN	5.3	4	0.9908	0.9949
1824	Heterogeneous nuclear ribonucleoprotein D-like OS=Homo sapiens GN=HNRNPDL PE=1 SV=3	sp|O14979|HNRDL_HUMAN	19.3	3	0.8872	0.7614
1825	Probable ATP-dependent RNA helicase DDX52 OS=Homo sapiens GN=DDX52 PE=1 SV=3	sp|Q9Y2R4|DDX52_HUMAN	9	2	0.9462	0.8892
1826	FK506-binding protein 15 OS=Homo sapiens GN=FKBP15 PE=1 SV=2	sp|Q5T1M5|FKB15_HUMAN	6	2	1.3552	0.5885
1827	Insulin-like growth factor 2 mRNA-binding protein 3 OS=Homo sapiens GN=IGF2BP3 PE=1 SV=2	sp|O00425|IF2B3_HUMAN	6.9	1	1.2023	0.8928
1828	Exosome complex component RRP4 OS=Homo sapiens GN=EXOSC2 PE=1 SV=2	sp|Q13868|EXOS2_HUMAN	17.4	1	0.9204	0.741
1829	Mitochondrial Rho GTPase 2 OS=Homo sapiens GN=RHOT2 PE=1 SV=2	sp|Q8IXI1|MIRO2_HUMAN	5.8	2	0.9908	0.9923
1830	Lys-63-specific deubiquitinase BRCC36 OS=Homo sapiens GN=BRCC3 PE=1	sp|P46736|BRCC3_HUMAN	10.4	2	0.8318	0.7261
1831	Peptidyl-prolyl cis-trans isomerase E OS=Homo sapiens GN=PPIE PE=1 SV=1	sp|Q9UNP9|PPIE_HUMAN	18.3	2	0.9908	0.9779
1832	Probable Xaa-Pro aminopeptidase 3 OS=Homo sapiens GN=XPNPEP3 PE=1 SV=1	sp|Q9NQH7|XPP3_HUMAN	8.1	2	1.2589	0.6559
1833	Protein polybromo-1 OS=Homo sapiens GN=PBRM1 PE=1	sp|Q86U86|PB1_HUMAN	4.9	1	0.7586	0.7609
1834	Dual specificity protein phosphatase 12 OS=Homo sapiens GN=DUSP12 PE=1 SV=1	sp|Q9UNI6|DUS12_HUMAN	6.5	2	0.8954	0.8361
1835	Cytoplasmic polyadenylation element-binding protein 3 OS=Homo sapiens GN=CPEB3 PE=1	sp|Q8NE35|CPEB3_HUMAN	5.6	2	0.9727	0.7557
1836	Nucleoporin p58/p45 OS=Homo sapiens GN=NUPL1 PE=1	sp|Q9BVL2|NUPL1_HUMAN	6.5	2	0.912	0.8873
1837	Cytoplasmic dynein 1 light intermediate chain 1 OS=Homo sapiens GN=DYNC1LI1 PE=1 SV=3	sp|Q9Y6G9|DC1L1_HUMAN	7.3	2	1.1376	0.7583

1838	Diphthine methyl ester synthase OS=Homo sapiens GN=DPH5 PE=1 SV=2	sp|Q9H2P9|DPH5_HUMAN	11.2	2	0.9204	0.8538
1839	Mannose-P-dolichol utilization defect 1 protein OS=Homo sapiens GN=MPDU1 PE=1 SV=2	sp|O75352|MPU1_HUMAN	10.1	2	0.8241	0.7237
1840	RNA-binding protein 10 OS=Homo sapiens GN=RBM10 PE=1	sp|P98175|RBM10_HUMAN	4.8	1	0.8954	0.5179
1841	Nuclear pore complex protein Nup98-Nup96 OS=Homo sapiens GN=NUP98 PE=1 SV=4	sp|P52948|NUP98_HUMAN	3.2	2	0.879	0.5973
1842	Thyroid adenoma-associated protein OS=Homo sapiens GN=THADA PE=1 SV=1	sp|Q6YHU6|THADA_HUMAN	3.9	2	0.8241	0.7207
1843	Rho GTPase-activating protein 11A OS=Homo sapiens GN=ARHGAP11A PE=1 SV=2	sp|Q6P4F7|RHGBA_HUMAN	5.4	1	0.863	0.5823
1844	Actin-related protein 2/3 complex subunit 4 OS=Homo sapiens GN=ARPC4 PE=1	sp|P59998|ARPC4_HUMAN	26.2	4	1.1482	0.767
1845	GPI-anchor transamidase OS=Homo sapiens GN=PIGK PE=1 SV=2	sp|Q92643|GPI8_HUMAN	16.7	2	1.0666	0.9213
1846	Activator of basal transcription 1 OS=Homo sapiens GN=ABT1 PE=1 SV=1	sp|Q9ULW3|ABT1_HUMAN	14	1	0.6368	0.558
1847	Scaffold attachment factor B1 OS=Homo sapiens GN=SAFB	sp|Q15424|SAFB1_HUMAN	4.2	1	1.0568	0.4021
1848	THO complex subunit 4 OS=Homo sapiens GN=ALYREF PE=1 SV=3	sp|Q86V81|THOC4_HUMAN	20.2	6	1.0965	0.839
1849	Transmembrane emp24 domain-containing protein 9 OS=Homo sapiens GN=TMED9 PE=1	sp|Q9BVK6|TMED9_HUMAN	25.5	3	1.0864	0.8841
1850	Melanoma inhibitory activity protein 3 OS=Homo sapiens GN=MIA3 PE=1	sp|Q5JRA6|MIA3_HUMAN	4.4	2	1.3428	0.3029
1851	Phosphatidylinositol 3,4,5-trisphosphate 5-phosphatase 2 OS=Homo sapiens GN=INPPL1 PE=1	sp|O15357|SHIP2_HUMAN	5	2	1.0186	0.9618
1852	REVERSED Serine/threonine-protein kinase RIO1 OS=Homo sapiens GN=RIOK1 PE=1	RRRRRsp|Q9BRS2|RIOK1_HUMAN	4.8	2	1.0765	0.955
1853	Keratin, type II cytoskeletal 8 OS=Homo sapiens GN=KRT8 PE=1 SV=7	sp|P05787|K2C8_HUMAN	9.9	1	0.7943	0.6468

1854	Vacuolar protein sorting-associated protein 41 homolog OS=Homo sapiens GN=VPS41 PE=1 SV=3	sp|P49754|VPS41_HUMAN	6.9	2	1.3305	0.8055
1855	Toll-interacting protein OS=Homo sapiens GN=TOLLIP PE=1 SV=1	sp|Q9H0E2|TOLIP_HUMAN	16.4	2	1.0666	0.8827
1856	KIF1-binding protein OS=Homo sapiens GN=KIF1BP PE=1	sp|Q96EK5|KBP_HUMAN	7.4	1	0.9376	0.8492
1857	BTB/POZ domain-containing protein KCTD12 OS=Homo sapiens GN=KCTD12 PE=1 SV=1	sp|Q96CX2|KCD12_HUMAN	10.2	2	1.5276	0.469
1858	Mitochondrial import inner membrane translocase subunit Tim9 OS=Homo sapiens GN=TIMM9 PE=1	sp|Q9Y5J7|TIM9_HUMAN	30.3	1	1.0765	0.8877
1859	Calcium-regulated heat stable protein 1 OS=Homo sapiens GN=CARHSP1	sp|Q9Y2V2|CHSP1_HUMAN	23.8	2	1.2359	0.6749
1860	Twinfilin-1 OS=Homo sapiens GN=TWF1 PE=1 SV=3	sp|Q12792|TWF1_HUMAN	14.3	2	1.0965	0.7941
1861	Arfaptin-1 OS=Homo sapiens GN=ARFIP1 PE=1 SV=2	sp|P53367|ARFP1_HUMAN	10.5	2	1.6144	0.6708
1862	40S ribosomal protein S30 OS=Homo sapiens GN=FAU PE=1 SV=1	sp|P62861|RS30_HUMAN	35.6	1	0.912	0.5941
1863	Exosome complex component RRP42 OS=Homo sapiens GN=EXOSC7 PE=1 SV=3	sp|Q15024|EXOS7_HUMAN	17.2	4		
1864	Cytochrome b-c1 complex subunit Rieske, mitochondrial OS=Homo sapiens GN=UQCRFS1 PE=1 SV=2	sp|P47985|UCRI_HUMAN	17.2	2	1	0.8679
1865	Trafficking protein particle complex subunit 3 OS=Homo sapiens GN=TRAPPC3 PE=1 SV=1	sp|O43617|TPPC3_HUMAN	15.6	1	1.3677	0.8127
1866	TraB domain-containing protein OS=Homo sapiens GN=TRABD PE=1 SV=1	sp|Q9H4I3|TRABD_HUMAN	6.1	1	1	0.9707
1867	N-alpha-acetyltransferase 35, NatC auxiliary subunit OS=Homo sapiens GN=NAA35 PE=1	sp|Q5VZE5|NAA35_HUMAN	3.4	1	1.028	0.9114
1868	AP-1 complex subunit beta-1 OS=Homo sapiens GN=AP1B1 PE=1 SV=2	sp|Q10567|AP1B1_HUMAN	11.4	6	1.2942	0.6294

1869	Spermatid perinuclear RNA-binding protein OS=Homo sapiens GN=STRBP PE=1 SV=1	sp|Q96SI9|STRBP_HUMAN	7.7	2	0.9376	0.897
1870	Neurolysin, mitochondrial OS=Homo sapiens GN=NLN PE=1 SV=1	sp|Q9BYT8|NEUL_HUMAN	10.1	2	0.7047	0.5412
1871	Calcium-binding protein 39 OS=Homo sapiens GN=CAB39 PE=1 SV=1	sp|Q9Y376|CAB39_HUMAN	18.5	2	0.9462	0.99
1872	Caspase-6 OS=Homo sapiens GN=CASP6 PE=1 SV=2	sp|P55212|CASP6_HUMAN	7.9	3	1.2246	0.689
1873	Terminal uridylyltransferase 4 OS=Homo sapiens GN=ZCCHC11	sp|Q5TAX3|TUT4_HUMAN	2.9	2	0.6855	0.734
1874	Bystin OS=Homo sapiens GN=BYSL PE=1 SV=3	sp|Q13895|BYST_HUMAN	12.4	2	0.929	0.9147
1875	Golgin subfamily B member 1 OS=Homo sapiens GN=GOLGB1 PE=1 SV=2	sp|Q14789|GOGB1_HUMAN	4.4	2	1.0666	0.9824
1876	PHD finger protein 14 OS=Homo sapiens GN=PHF14 PE=1 SV=2	sp|O94880|PHF14_HUMAN	3.5	2	0.7178	0.616
1877	Eukaryotic translation initiation factor 4E-binding protein 1 OS=Homo sapiens GN=EIF4EBP1 PE=1 SV=3	sp|Q13541|4EBP1_HUMAN	25.4	3	1.2023	0.4083
1878	Tyrosine-protein kinase Fgr OS=Homo sapiens GN=FGR PE=1 SV=2	sp|P09769|FGR_HUMAN	4.5	1	1.0471	0.8719
1879	RNA-binding protein 12 OS=Homo sapiens GN=RBM12 PE=1	sp|Q9NTZ6|RBM12_HUMAN	5.2	1	0.8318	0.739
1880	NADH dehydrogenase [ubiquinone] iron-sulfur protein 3, mitochondrial OS=Homo sapiens GN=NDUFS3 PE=1 SV=1	sp|O75489|NDUS3_HUMAN	13.6	1	1.0375	0.7655
1881	Death-inducer obliterator 1 OS=Homo sapiens GN=DIDO1 PE=1 SV=5	sp|Q9BTC0|DIDO1_HUMAN	3.7	1	0.912	0.8955
1882	ATP-dependent RNA helicase DHX29 OS=Homo sapiens GN=DHX29 PE=1	sp|Q7Z478|DHX29_HUMAN	7.8	3	0.9727	0.9657
1883	Protein VAC14 homolog OS=Homo sapiens GN=VAC14 PE=1	sp|Q08AM6|VAC14_HUMAN	10.1	4	1.0375	0.8617
1884	Probable ATP-dependent RNA helicase YTHDC2 OS=Homo sapiens GN=YTHDC2 PE=1 SV=2	sp|Q9H6S0|YTDC2_HUMAN	3.6	1	1.0568	0.9258

1885	NEDD4-like E3 ubiquitin-protein ligase WWP1 OS=Homo sapiens GN=WWP1 PE=1 SV=1	sp|Q9H0M0|WWP1_HUMAN	5.2	1	0.8954	0.8711
1886	S1 RNA-binding domain-containing protein 1 OS=Homo sapiens GN=SRBD1 PE=1	sp|Q8N5C6|SRBD1_HUMAN	6.4	2	0.8954	0.8419
1887	Electron transfer flavoprotein subunit beta OS=Homo sapiens GN=ETFB PE=1	sp|P38117|ETFB_HUMAN	31.8	2	1.0093	0.9751
1888	Membrane-associated phosphatidylinositol transfer protein 1 OS=Homo sapiens GN=PITPNM1 PE=1 SV=4	sp|O00562|PITM1_HUMAN	3.2	2	1.5704	0.4505
1889	Golgi resident protein GCP60 OS=Homo sapiens GN=ACBD3 PE=1 SV=4	sp|Q9H3P7|GCP60_HUMAN	8.7	3	1.2942	0.6132
1890	Surfeit locus protein 6 OS=Homo sapiens GN=SURF6 PE=1	sp|O75683|SURF6_HUMAN	11.9	1	0.7943	0.4053
1891	Protoporphyrinogen oxidase OS=Homo sapiens GN=PPOX PE=1	sp|P50336|PPOX_HUMAN	5.2	1	0.8395	0.7524
1892	1,2-dihydroxy-3-keto-5-methylthiopentene dioxygenase OS=Homo sapiens GN=ADI1 PE=1 SV=1	sp|Q9BV57|MTND_HUMAN	17.3	2	0.7379	0.5927
1893	Protein farnesyltransferase subunit beta OS=Homo sapiens GN=FNTB PE=1 SV=1	sp|P49356|FNTB_HUMAN	5.7	2	0.8954	0.8254
1894	1-phosphatidylinositol 3-phosphate 5-kinase OS=Homo sapiens GN=PIKFYVE	sp|Q9Y2I7|FYV1_HUMAN	4.5	1	1.1803	0.683
1895	REVERSED Unconventional myosin-XVI OS=Homo sapiens GN=MYO16 PE=2 SV=3	RRRRRsp|Q9Y6X6|MYO16_HUMAN	3.2	1	0.955	0.8098
1896	Anaphase-promoting complex subunit 2 OS=Homo sapiens GN=ANAPC2 PE=1 SV=1	sp|Q9UJX6|ANC2_HUMAN	7.3	1	0.9462	0.9274
1897	FH2 domain-containing protein 1 OS=Homo sapiens GN=FHDC1 PE=1	sp|Q9C0D6|FHDC1_HUMAN	3.9	1	0.7379	0.5854
1898	Ran-binding protein 9 OS=Homo sapiens GN=RANBP9	sp|Q96S59|RANB9_HUMAN	11	1	0.9638	0.9819

1899	TAF6-like RNA polymerase II p300/CBP-associated factor-associated factor 65 kDa subunit 6L OS=Homo sapiens GN=TAF6L PE=1 SV=1	sp|Q9Y6J9|TAF6L_HUMAN	7.2	2	1.0093	0.9635
1900	Methylthioribose-1-phosphate isomerase OS=Homo sapiens GN=MRI1 PE=1	sp|Q9BV20|MTNA_HUMAN	17.1	1	1.0568	0.9183
1901	Neutrophil cytosol factor 2 OS=Homo sapiens GN=NCF2 PE=1 SV=2	sp|P19878|NCF2_HUMAN	13.1	3	1.1066	0.8225
1902	Exostosin-like 3 OS=Homo sapiens GN=EXTL3 PE=1 SV=1	sp|O43909|EXTL3_HUMAN	4.7	1	0.9462	0.8685
1903	Calcineurin B homologous protein 1 OS=Homo sapiens GN=CHP1 PE=1	sp|Q99653|CHP1_HUMAN	24.1	1	1.1376	0.7763
1904	Vacuolar protein sorting-associated protein 52 homolog OS=Homo sapiens GN=VPS52 PE=1 SV=1	sp|Q8N1B4|VPS52_HUMAN	4.3	1	0.9036	0.7486
1905	WD repeat-containing protein 44 OS=Homo sapiens GN=WDR44 PE=1	sp|Q5JSH3|WDR44_HUMAN	4.9	2	1.0186	0.9696
1906	Neutral amino acid transporter B(0) OS=Homo sapiens GN=SLC1A5 PE=1 SV=2	sp|Q15758|AAAT_HUMAN	10.4	3	0.7943	0.8633
1907	AH receptor-interacting protein OS=Homo sapiens GN=AIP PE=1 SV=2	sp|O00170|AIP_HUMAN	11.5	1	0.871	0.7888
1908	Nuclear receptor coactivator 5 OS=Homo sapiens GN=NCOA5 PE=1 SV=2	sp|Q9HCD5|NCOA5_HUMAN	8.3	2	0.8318	0.7374
1909	Rhophilin-2 OS=Homo sapiens GN=RHPN2 PE=1 SV=1	sp|Q8IUC4|RHPN2_HUMAN	4.7	1	1.1376	0.788
1910	26S proteasome non-ATPase regulatory subunit 8 OS=Homo sapiens GN=PSMD8 PE=1	sp|P48556|PSMD8_HUMAN	10.6	2	1.0666	0.873
1911	Nibrin OS=Homo sapiens GN=NBN PE=1 SV=1	sp|O60934|NBN_HUMAN	4.6	1	0.8954	0.6707
1912	Hydroxymethylglutaryl-CoA lyase, mitochondrial OS=Homo sapiens GN=HMGCL PE=1 SV=2	sp|P35914|HMGCL_HUMAN	8.9	1	0.8017	0.6427
1913	26S proteasome non-ATPase regulatory subunit 10 OS=Homo sapiens GN=PSMD10 PE=1 SV=1	sp|O75832|PSD10_HUMAN	21.2	2	0.4699	0.6239
1914	TIP41-like protein OS=Homo sapiens GN=TIPRL PE=1 SV=2	sp|O75663|TIPRL_HUMAN	15.8	2	1.0375	0.9052

1915	RNA-binding protein 7 OS=Homo sapiens GN=RBM7 PE=1	sp|Q9Y580|RBM7_HUMAN	12.8	1	0.9204	0.826
1916	THO complex subunit 6 homolog OS=Homo sapiens GN=THOC6 PE=1 SV=1	sp|Q86W42|THOC6_HUMAN	7.6	2		
1917	UPF0469 protein KIAA0907 OS=Homo sapiens GN=KIAA0907 PE=1 SV=1	sp|Q7Z7F0|K0907_HUMAN	4.6	1	0.8166	0.7094
1918	Protein Smaug homolog 2 OS=Homo sapiens GN=SAMD4B PE=1 SV=1	sp|Q5PRF9|SMAG2_HUMAN	4.5	1	0.8395	0.7754
1919	N-alpha-acetyltransferase 20 OS=Homo sapiens GN=NAA20 PE=1 SV=1	sp|P61599|NAA20_HUMAN	14.6	1	1.0666	0.7831
1920	Transcription factor ETV6 OS=Homo sapiens GN=ETV6 PE=1 SV=1	sp|P41212|ETV6_HUMAN	4	1	0.871	0.657
1921	REVERSED Signal-transducing adaptor protein 1 OS=Homo sapiens GN=STAP1 PE=1	RRRRRsp|Q9ULZ2|STAP1_HUMAN	9.5	1	1.1803	0.7284
1922	Peptidyl-prolyl cis-trans isomerase-like 1 OS=Homo sapiens GN=PPIL1 PE=1 SV=1	sp|Q9Y3C6|PPIL1_HUMAN	10.8	1	0.6546	0.7415
1923	Neudesin OS=Homo sapiens GN=NENF PE=1	sp|Q9UMX5|NENF_HUMAN	15.1	1	0.912	0.7682
1924	THO complex subunit 3 OS=Homo sapiens GN=THOC3 PE=1	sp|Q96J01|THOC3_HUMAN	12	2	0.9376	0.914
1925	1-acylglycerol-3-phosphate O-acyltransferase ABHD5 OS=Homo sapiens GN=ABHD5 PE=1 SV=1	sp|Q8WTS1|ABHD5_HUMAN	5.2	1	0.8954	0.7791
1926	S-adenosylmethionine synthase isoform type-2 OS=Homo sapiens GN=MAT2A PE=1 SV=1	sp|P31153|METK2_HUMAN	6.1	1	0.863	0.7484
1927	Cytochrome b-c1 complex subunit 7 OS=Homo sapiens GN=UQCRB PE=1 SV=2	sp|P14927|QCR7_HUMAN	9.9	1	1.028	0.8736
1928	Tubulin alpha-4A chain OS=Homo sapiens GN=TUBA4A PE=1 SV=1	sp|P68366|TBA4A_HUMAN	57.8	45	0.9376	0.8702
1929	Tubulin alpha-1C chain OS=Homo sapiens GN=TUBA1C PE=1 SV=1	sp|Q9BQE3|TBA1C_HUMAN	59.5	46	0.9462	0.9351
1930	Sodium/potassium-transporting ATPase subunit alpha-3 OS=Homo sapiens GN=ATP1A3	sp|P13637|AT1A3_HUMAN	20.3	30	1.0864	0.8394
1931	Actin, alpha cardiac muscle 1 OS=Homo sapiens GN=ACTC1 PE=1	sp|P68032|ACTC_HUMAN	57.6	149	1.1272	0.5907

1932	Histone H2A type 2-A OS=Homo sapiens GN=HIST2H2AA3 PE=1 SV=3	sp|Q6FI13|H2A2A_HUMAN	82.3	57	0.1754	0.6361
1933	Histone H3.3 OS=Homo sapiens GN=H3F3A PE=1 SV=2	sp|P84243|H33_HUMAN	68.4	17	0.166	0.8449
1934	Ankyrin repeat and KH domain-containing protein 1 OS=Homo sapiens GN=ANKHD1	sp|Q8IWZ3|ANKH1_HUMAN	5.2	7	1.1482	0.7763
1935	Serine/threonine-protein phosphatase 2A 65 kDa regulatory subunit A beta isoform OS=Homo sapiens GN=PPP2R1B PE=1 SV=3	sp|P30154|2AAB_HUMAN	19.3	9	1.0765	0.8788
1936	Myosin-10 OS=Homo sapiens GN=MYH10 PE=1 SV=3	sp|P35580|MYH10_HUMAN	10.1	5	1.4723	0.9436
1937	Eukaryotic peptide chain release factor GTP-binding subunit ERF3B OS=Homo sapiens GN=GSPT2 PE=1	sp|Q8IYD1|ERF3B_HUMAN	16.2	6		
1938	Nucleoside diphosphate kinase A OS=Homo sapiens GN=NME1 PE=1 SV=1	sp|P15531|NDKA_HUMAN	34.9	5	0.8954	0.8436
1939	Beta-actin-like protein 2 OS=Homo sapiens GN=ACTBL2 PE=1 SV=2	sp|Q562R1|ACTBL_HUMAN	33.5	20	1.1803	0.6111
1940	Serine/threonine-protein phosphatase 2A catalytic subunit beta isoform OS=Homo sapiens GN=PPP2CB PE=1	sp|P62714|PP2AB_HUMAN	24.3	9	1.2823	0.6169
1941	Ubiquilin-2 OS=Homo sapiens GN=UBQLN2 PE=1 SV=2	sp|Q9UHD9|UBQL2_HUMAN	15.9	6	0.4055	0.3978
1942	Serine/threonine-protein kinase 3 OS=Homo sapiens GN=STK3 PE=1 SV=2	sp|Q13188|STK3_HUMAN	10.4	7	1.0186	0.953
1943	Inositol 1,4,5-trisphosphate receptor type 1 OS=Homo sapiens GN=ITPR1 PE=1 SV=3	sp|Q14643|ITPR1_HUMAN	3.3	4	1.2359	0.6723
1944	Importin subunit alpha-7 OS=Homo sapiens GN=KPNA6 PE=1 SV=1	sp|O60684|IMA7_HUMAN	12.5	3		
1945	Basic leucine zipper and W2 domain-containing protein 2 OS=Homo sapiens GN=BZW2 PE=1	sp|Q9Y6E2|BZW2_HUMAN	11.5	3	0.8472	0.7519
1946	Dynein light chain 1, cytoplasmic OS=Homo sapiens GN=DYNLL1 PE=1 SV=1	sp|P63167|DYL1_HUMAN	42.7	5	0.871	0.8275

1947	Putative ciliary rootlet coiled-coil protein-like 3 protein OS=Homo sapiens PE=5 SV=3	sp|H7BZ55|CROL3_HUMAN	8.6	3	1.2023	0.704
1948	Proline-, glutamic acid- and leucine-rich protein 1 OS=Homo sapiens GN=PELP1 PE=1 SV=2	sp|Q8IZL8|PELP1_HUMAN	4.7	3	0.879	0.808
1949	Sideroflexin-3 OS=Homo sapiens GN=SFXN3 PE=1 SV=2	sp|Q9BWM7|SFXN3_HUMAN	11.4	4	1.2474	0.6612
1950	Methionine--tRNA ligase, mitochondrial OS=Homo sapiens GN=MARS2 PE=1	sp|Q96GW9|SYMM_HUMAN	7.3	3		
1951	Protein transport protein Sec24B OS=Homo sapiens GN=SEC24B PE=1 SV=2	sp|O95487|SC24B_HUMAN	5.8	3	1	0.9968
1952	Tetratricopeptide repeat protein 27 OS=Homo sapiens GN=TTC27 PE=1 SV=1	sp|Q6P3X3|TTC27_HUMAN	9.1	4	0.879	0.77
1953	Tyrosine-protein kinase SgK223 OS=Homo sapiens GN=SGK223 PE=1 SV=4	sp|Q86YV5|SG223_HUMAN	3.6	2	1.1272	0.8003
1954	Probable ribosome biogenesis protein RLP24 OS=Homo sapiens GN=RSL24D1 PE=1 SV=1	sp|Q9UHA3|RLP24_HUMAN	14.1	3	0.8241	0.724
1955	RANBP2-like and GRIP domain-containing protein 1 OS=Homo sapiens GN=RGPD1 PE=2	sp|P0DJD0|RGPD1_HUMAN	5.3	2	1.0864	0.2925
1956	Dual specificity protein kinase TTK OS=Homo sapiens GN=TTK PE=1 SV=2	sp|P33981|TTK_HUMAN	4.4	2	0.492	0.3246
1957	Solute carrier family 12 member 5 OS=Homo sapiens GN=SLC12A5	sp|Q9H2X9|S12A5_HUMAN	4.7	2	1.1588	0.7628
1958	Cytosolic carboxypeptidase 1 OS=Homo sapiens GN=AGTPBP1 PE=1 SV=3	sp|Q9UPW5|CBPC1_HUMAN	4.7	2	1	0.9091
1959	Guanine nucleotide-binding protein G(k) subunit alpha OS=Homo sapiens GN=GNAI3 PE=1	sp|P08754|GNAI3_HUMAN	22	3	1.1803	0.6605
1960	Cohesin subunit SA-1 OS=Homo sapiens GN=STAG1 PE=1	sp|Q8WVM7|STAG1_HUMAN	5.5	2	0.955	0.9425
1961	TBC domain-containing protein kinase-like protein OS=Homo sapiens GN=TBCK PE=1 SV=4	sp|Q8TEA7|TBCK_HUMAN	4.7	2		
1962	Mitogen-activated protein kinase 3 OS=Homo sapiens GN=MAPK3 PE=1 SV=4	sp|P27361|MK03_HUMAN	11.9	2	1.1272	0.7988

1963	Protein sidekick-1 OS=Homo sapiens GN=SDK1 PE=2 SV=3	sp|Q7Z5N4|SDK1_HUMAN	3.3	2	1.0864	0.8581
1964	Prenylcysteine oxidase-like OS=Homo sapiens GN=PCYOX1L PE=1 SV=2	sp|Q8NBM8|PCYXL_HUMAN	9.3	2	0.7656	0.6041
1965	Spermatogenesis-associated protein 5 OS=Homo sapiens GN=SPATA5 PE=1 SV=3	sp|Q8NB90|SPAT5_HUMAN	6.8	3	1.0093	0.9657
1966	Adenosylhomocysteinase 3 OS=Homo sapiens GN=AHCYL2 PE=1 SV=1	sp|Q96HN2|SAHH3_HUMAN	8.2	3	1	0.9929
1967	Interferon regulatory factor 5 OS=Homo sapiens GN=IRF5 PE=1 SV=2	sp|Q13568|IRF5_HUMAN	10.6	2	0.6668	0.4984
1968	GTP-binding protein 8 OS=Homo sapiens GN=GTPBP8	sp|Q8N3Z3|GTPB8_HUMAN	9.9	2	0.9638	0.9678
1969	Adenylyl cyclase-associated protein 2 OS=Homo sapiens GN=CAP2 PE=1 SV=1	sp|P40123|CAP2_HUMAN	4.8	2	0.9817	0.9842
1970	Ras association domain-containing protein 4 OS=Homo sapiens GN=RASSF4 PE=1 SV=2	sp|Q9H2L5|RASF4_HUMAN	11.2	2		
1971	Protein-associating with the carboxyl-terminal domain of ezrin OS=Homo sapiens GN=SCYL3 PE=1	sp|Q8IZE3|PACE1_HUMAN	2.6	2	1.2474	0.6681
1972	[Pyruvate dehydrogenase (acetyl-transferring)] kinase isozyme 1, mitochondrial OS=Homo sapiens GN=PDK1 PE=1 SV=1	sp|Q15118|PDK1_HUMAN	6.9	3	0.8954	0.8109
1973	Septin-6 OS=Homo sapiens GN=SEPT6 PE=1	sp|Q14141|SEPT6_HUMAN	6.7	2	1.028	0.9344
1974	Protein tyrosine phosphatase type IVA 2 OS=Homo sapiens GN=PTP4A2 PE=1 SV=1	sp|Q12974|TP4A2_HUMAN	19.8	2	0.3873	0.5206
1975	Chromobox protein homolog 1 OS=Homo sapiens GN=CBX1 PE=1 SV=1	sp|P83916|CBX1_HUMAN	16.8	3	0.929	0.8985
1976	Thioredoxin domain-containing protein 12 OS=Homo sapiens GN=TXNDC12 PE=1 SV=1	sp|O95881|TXD12_HUMAN	16.3	2	1.5136	0.4733
1977	GMP reductase 1 OS=Homo sapiens GN=GMPR PE=1 SV=1	sp|P36959|GMPR1_HUMAN	7.2	2	1.0765	0.8692
1978	Ras-related protein R-Ras OS=Homo sapiens GN=RRAS PE=1 SV=1	sp|P10301|RRAS_HUMAN	12.8	2	1.1912	0.6063

1979	ATP-dependent RNA helicase DDX50 OS=Homo sapiens GN=DDX50 PE=1	sp|Q9BQ39|DDX50_HUMAN	7.3	2	0.9204	0.8677
1980	ARF GTPase-activating protein GIT2 OS=Homo sapiens GN=GIT2 PE=1 SV=2	sp|Q14161|GIT2_HUMAN	3.7	2	1.1272	0.7926
1981	REVERSED Plectin OS=Homo sapiens GN=PLEC PE=1 SV=3	RRRRRsp|Q15149|PLEC_HUMAN	7.8	4		
1982	High mobility group protein B3 OS=Homo sapiens GN=HMGB3 PE=1 SV=4	sp|O15347|HMGB3_HUMAN	9.5	2	0.7516	0.6066
1983	Solute carrier family 25 member 36 OS=Homo sapiens GN=SLC25A36 PE=1 SV=1	sp|Q96CQ1|S2536_HUMAN	5.8	2	1.028	0.9461
1984	Telomere-associated protein RIF1 OS=Homo sapiens GN=RIF1 PE=1 SV=2	sp|Q5UIP0|RIF1_HUMAN	2.2	3	0.6792	0.5081
1985	Cyclin-A1 OS=Homo sapiens GN=CCNA1 PE=1 SV=1	sp|P78396|CCNA1_HUMAN	5.8	2	0.5702	0.381
1986	Structural maintenance of chromosomes protein 6 OS=Homo sapiens GN=SMC6 PE=1	sp|Q96SB8|SMC6_HUMAN	3.8	2	0.6918	0.5266
1987	Xylosyltransferase 2 OS=Homo sapiens GN=XYLT2 PE=2	sp|Q9H1B5|XYLT2_HUMAN	2.4	2	1.1695	0.7448
1988	Solute carrier family 2, facilitated glucose transporter member 3 OS=Homo sapiens GN=SLC2A3 PE=2 SV=1	sp|P11169|GTR3_HUMAN	8.5	4	1.1376	0.7553
1989	UV radiation resistance-associated gene protein OS=Homo sapiens GN=UVRAG PE=1 SV=1	sp|Q9P2Y5|UVRAG_HUMAN	5.6	2	1.0765	0.8695
1990	Histone-binding protein RBBP4 OS=Homo sapiens GN=RBBP4 PE=1 SV=3	sp|Q09028|RBBP4_HUMAN	10.8	2	0.6918	0.5233
1991	Conserved oligomeric Golgi complex subunit 5 OS=Homo sapiens GN=COG5 PE=1 SV=3	sp|Q9UP83|COG5_HUMAN	5.2	2	1	0.967
1992	SEC14-like protein 1 OS=Homo sapiens GN=SEC14L1	sp|Q92503|S14L1_HUMAN	4.3	2	0.8954	0.8387
1993	Protein LCHN OS=Homo sapiens GN=LCHN PE=2 SV=1	sp|A4D1U4|LCHN_HUMAN	7	2	1.1169	0.8055
1994	Oxidoreductase HTATIP2 OS=Homo sapiens GN=HTATIP2 PE=1 SV=2	sp|Q9BUP3|HTAI2_HUMAN	11.2	2	1.1912	0.7221

1995	Zinc finger protein 827 OS=Homo sapiens GN=ZNF827 PE=2 SV=1	sp|Q17R98|ZN827_HUMAN	2.3	2	0.9817	0.9826
1996	Serine/threonine-protein kinase VRK1 OS=Homo sapiens GN=VRK1 PE=1 SV=1	sp|Q99986|VRK1_HUMAN	5.1	2		
1997	Serine/arginine repetitive matrix protein 1 OS=Homo sapiens GN=SRRM1 PE=1	sp|Q8IYB3|SRRM1_HUMAN	2.5	2		
1998	Rho GTPase-activating protein 33 OS=Homo sapiens GN=ARHGAP33	sp|O14559|RHG33_HUMAN	3.8	1	0.8551	0.7482
1999	Cleavage and polyadenylation specificity factor subunit 5 OS=Homo sapiens GN=NUDT21 PE=1 SV=1	sp|O43809|CPSF5_HUMAN	15.4	2	0.9036	0.8467
2000	UDP-N-acetylhexosamine pyrophosphorylase OS=Homo sapiens GN=UAP1 PE=1 SV=3	sp|Q16222|UAP1_HUMAN	7.5	3	1	0.9574
2001	Probable ATP-dependent RNA helicase DHX37 OS=Homo sapiens GN=DHX37 PE=1 SV=1	sp|Q8IY37|DHX37_HUMAN	5.6	1	0.7311	0.578
2002	Chondroitin sulfate proteoglycan 4 OS=Homo sapiens GN=CSPG4 PE=1 SV=2	sp|Q6UVK1|CSPG4_HUMAN	3.5	2	1.6144	0.4655
2003	Fanconi anemia group I protein OS=Homo sapiens GN=FANCI PE=1 SV=4	sp|Q9NVI1|FANCI_HUMAN	4	1	0.6081	0.4261
2004	Sin3 histone deacetylase corepressor complex component SDS3 OS=Homo sapiens GN=SUDS3 PE=1	sp|Q9H7L9|SDS3_HUMAN	10.7	2	0.7943	0.6769
2005	Superoxide dismutase [Cu-Zn] OS=Homo sapiens GN=SOD1 PE=1 SV=2	sp|P00441|SODC_HUMAN	17.5	2		
2006	RNA-binding protein 5 OS=Homo sapiens GN=RBM5 PE=1	sp|P52756|RBM5_HUMAN	5.3	1	0.9036	0.8611
2007	N-acetylglucosamine-6-sulfatase OS=Homo sapiens GN=GNS PE=1 SV=3	sp|P15586|GNS_HUMAN	10.5	2	1.1376	0.8719
2008	Mucosa-associated lymphoid tissue lymphoma translocation protein 1 OS=Homo sapiens GN=MALT1 PE=1	sp|Q9UDY8|MALT1_HUMAN	4	2	1.0375	0.8954
2009	Stereocilin OS=Homo sapiens GN=STRC PE=2 SV=1	sp|Q7RTU9|STRC_HUMAN	4.1	2		

2010	REVERSED Zinc finger protein 420 OS=Homo sapiens GN=ZNF420 PE=1 SV=1	RRRRRsp|Q8TAQ5|ZN420_HUMAN	4.4	2		
2011	DNA repair protein RAD50 OS=Homo sapiens GN=RAD50 PE=1	sp|Q92878|RAD50_HUMAN	6.8	4	0.7047	0.5441
2012	NF-kappa-B-repressing factor OS=Homo sapiens GN=NKRF PE=1 SV=2	sp|O15226|NKRF_HUMAN	6.8	1	0.9376	0.9237
2013	Kinesin-like protein KIF15 OS=Homo sapiens GN=KIF15 PE=1	sp|Q9NS87|KIF15_HUMAN	5.8	1	0.9638	0.5541
2014	Protein diaphanous homolog 3 OS=Homo sapiens GN=DIAPH3 PE=1 SV=4	sp|Q9NSV4|DIAP3_HUMAN	4.3	3	1.2589	0.6479
2015	Protein SZT2 OS=Homo sapiens GN=SZT2 PE=1 SV=3	sp|Q5T011|SZT2_HUMAN	3.6	3	1.0093	0.9622
2016	Forkhead box protein K1 OS=Homo sapiens GN=FOXK1 PE=1	sp|P85037|FOXK1_HUMAN	5.6	1	1.0186	0.9505
2017	Phospholipase D4 OS=Homo sapiens GN=PLD4 PE=2 SV=2	sp|Q96BZ4|PLD4_HUMAN	8.3	2	1.6596	0.4127
2018	Metastasis-associated protein MTA3 OS=Homo sapiens GN=MTA3 PE=1 SV=2	sp|Q9BTC8|MTA3_HUMAN	7.2	2	1.0765	0.8669
2019	Hydroxyacylglutathione hydrolase, mitochondrial OS=Homo sapiens GN=HAGH PE=1 SV=2	sp|Q16775|GLO2_HUMAN	14	3	1.1803	0.7299
2020	39S ribosomal protein L13, mitochondrial OS=Homo sapiens GN=MRPL13 PE=1 SV=1	sp|Q9BYD1|RM13_HUMAN	20.8	3	0.863	0.7782
2021	GTPase Era, mitochondrial OS=Homo sapiens GN=ERAL1 PE=1 SV=2	sp|O75616|ERAL1_HUMAN	4.1	2	0.863	0.78
2022	E3 ubiquitin-protein ligase RNF123 OS=Homo sapiens GN=RNF123 PE=1	sp|Q5XPI4|RN123_HUMAN	4.2	3	1.2359	0.6702
2023	Putative ATP-dependent RNA helicase DDX12 OS=Homo sapiens GN=DDX12P PE=5 SV=3	sp|Q92771|DDX12_HUMAN	6.5	2	0.5495	0.3726
2024	Arf-GAP with GTPase, ANK repeat and PH domain-containing protein 3 OS=Homo sapiens GN=AGAP3 PE=1 SV=2	sp|Q96P47|AGAP3_HUMAN	7.2	2	0.871	0.7667
2025	Golgi-associated PDZ and coiled-coil motif-containing protein OS=Homo sapiens GN=GOPC PE=1	sp|Q9HD26|GOPC_HUMAN	2.8	3	0.9204	0.8798

2026	Apoptotic chromatin condensation inducer in the nucleus OS=Homo sapiens	sp|Q9UKV3|ACINU_HUMAN	4.4	3	0.9908	0.9982
2027	Son of sevenless homolog 2 OS=Homo sapiens GN=SOS2 PE=1 SV=2	sp|Q07890|SOS2_HUMAN	2.7	2	0.0163	0.032
2028	Protein EFR3 homolog A OS=Homo sapiens GN=EFR3A PE=1 SV=2	sp|Q14156|EFR3A_HUMAN	3.7	2	0.8318	0.7081
2029	Zinc finger B-box domain-containing protein 1 OS=Homo sapiens GN=ZBBX PE=2	sp|A8MT70|ZBBX_HUMAN	3	2	1.0864	0.8325
2030	Protein phosphatase 1 regulatory subunit 21 OS=Homo sapiens GN=PPP1R21	sp|Q6ZMI0|PPR21_HUMAN	9.2	2	1.0864	0.8678
2031	Multidrug resistance-associated protein 5 OS=Homo sapiens GN=ABCC5 PE=1 SV=2	sp|O15440|MRP5_HUMAN	4.5	1		
2032	Arginine and glutamate-rich protein 1 OS=Homo sapiens GN=ARGLU1 PE=1 SV=1	sp|Q9NWB6|ARGL1_HUMAN	9.9	2		
2033	Coiled-coil domain-containing protein 169 OS=Homo sapiens GN=CCDC169 PE=2 SV=4	sp|A6NNP5|CC169_HUMAN	11.2	1		
2034	Probable cytosolic iron-sulfur protein assembly protein CIAO1 OS=Homo sapiens GN=CIAO1 PE=1	sp|O76071|CIAO1_HUMAN	9.1	2	0.9462	0.9633
2035	Golgin subfamily A member 2 OS=Homo sapiens GN=GOLGA2 PE=1 SV=3	sp|Q08379|GOGA2_HUMAN	6	2	0.9727	0.9682
2036	Leucine carboxyl methyltransferase 1 OS=Homo sapiens GN=LCMT1 PE=1 SV=2	sp|Q9UIC8|LCMT1_HUMAN	8.4	2	1.1588	0.7578
2037	Calcium uniporter regulatory subunit MCUb, mitochondrial OS=Homo sapiens GN=CCDC109B PE=1 SV=2	sp|Q9NWR8|MCUB_HUMAN	6.5	1	0.9376	0.9083
2038	N-terminal Xaa-Pro-Lys N-methyltransferase 1 OS=Homo sapiens GN=NTMT1 PE=1 SV=3	sp|Q9BV86|NTM1A_HUMAN	13	2	0.9638	0.9339
2039	Short coiled-coil protein OS=Homo sapiens GN=SCOC PE=1 SV=2	sp|Q9UIL1|SCOC_HUMAN	14.5	2	1.1169	0.8245
2040	DNA helicase MCM8 OS=Homo sapiens GN=MCM8 PE=1	sp|Q9UJA3|MCM8_HUMAN	5.5	1	1.1588	0.757

2041	3-keto-steroid reductase OS=Homo sapiens GN=HSD17B7 PE=1 SV=1	sp|P56937|DHB7_HUMAN	9.4	2	1.7219	0.5485
2042	V-type proton ATPase subunit S1 OS=Homo sapiens GN=ATP6AP1 PE=1 SV=2	sp|Q15904|VAS1_HUMAN	8.7	2	0.871	0.7932
2043	AT-rich interactive domain-containing protein 3B OS=Homo sapiens GN=ARID3B PE=1 SV=2	sp|Q8IVW6|ARI3B_HUMAN	6.6	3	1	0.9621
2044	2-oxoisovalerate dehydrogenase subunit alpha, mitochondrial OS=Homo sapiens GN=BCKDHA PE=1 SV=2	sp|P12694|ODBA_HUMAN	6.1	2	1.0093	0.9627
2045	Sorting nexin-4 OS=Homo sapiens GN=SNX4 PE=1 SV=1	sp|O95219|SNX4_HUMAN	7.6	1	1.1376	0.791
2046	Protein scribble homolog OS=Homo sapiens GN=SCRIB PE=1	sp|Q14160|SCRIB_HUMAN	4.2	2	1.1803	0.7345
2047	Absent in melanoma 1 protein OS=Homo sapiens GN=AIM1 PE=1	sp|Q9Y4K1|AIM1_HUMAN	2.8	1	1.0093	0.9644
2048	Hermansky-Pudlak syndrome 5 protein OS=Homo sapiens GN=HPS5 PE=1 SV=2	sp|Q9UPZ3|HPS5_HUMAN	5	2	1.0471	0.9039
2049	Pseudopodium-enriched atypical kinase 1 OS=Homo sapiens GN=PEAK1 PE=1	sp|Q9H792|PEAK1_HUMAN	2.7	1	1.0093	0.9675
2050	Protein misato homolog 1 OS=Homo sapiens GN=MSTO1 PE=1 SV=1	sp|Q9BUK6|MSTO1_HUMAN	9.5	2	1.028	0.92
2051	Cation channel sperm-associated protein subunit delta OS=Homo sapiens GN=CATSPERD	sp|Q86XM0|CTSRD_HUMAN	4.1	1		
2052	Adaptin ear-binding coat-associated protein 1 OS=Homo sapiens GN=NECAP1 PE=1 SV=2	sp|Q8NC96|NECP1_HUMAN	20	1	1.0864	0.8552
2053	Adaptin ear-binding coat-associated protein 2 OS=Homo sapiens GN=NECAP2 PE=1 SV=1	sp|Q9NVZ3|NECP2_HUMAN	19.4	1	1.1376	0.8005
2054	REVERSED Adenomatous polyposis coli protein 2 OS=Homo sapiens GN=APC2 PE=1	RRRRRsp|O95996|APC2_HUMAN	5.6	2	0.879	0.8163
2055	Uncharacterized protein KIAA1211 OS=Homo sapiens GN=KIAA1211 PE=1 SV=3	sp|Q6ZU35|K1211_HUMAN	6	1	1.1272	0.803
2056	Gem-associated protein 5 OS=Homo sapiens GN=GEMIN5 PE=1 SV=3	sp|Q8TEQ6|GEMI5_HUMAN	4.9	2	1.0471	0.9125

2057	Uncharacterized protein C9orf84 OS=Homo sapiens GN=C9orf84 PE=2 SV=1	sp|Q5VXU9|CI084_HUMAN	4.1	2	1.2706	0.6348
2058	Exocyst complex component 6 OS=Homo sapiens GN=EXOC6 PE=1 SV=3	sp|Q8TAG9|EXOC6_HUMAN	4.5	1	0.912	0.8678
2059	DnaJ homolog subfamily A member 3, mitochondrial OS=Homo sapiens GN=DNAJA3 PE=1 SV=2	sp|Q96EY1|DNJA3_HUMAN	12.5	2	0.879	0.7966
2060	MAP7 domain-containing protein 1 OS=Homo sapiens GN=MAP7D1	sp|Q3KQU3|MA7D1_HUMAN	5.1	1	0.6427	0.4677
2061	ATP-dependent RNA helicase DDX55 OS=Homo sapiens GN=DDX55 PE=1	sp|Q8NHQ9|DDX55_HUMAN	6	2	0.9376	0.9028
2062	ATP-binding cassette sub-family B member 8, mitochondrial OS=Homo sapiens GN=ABCB8 PE=1 SV=3	sp|Q9NUT2|ABCB8_HUMAN	5.7	2		
2063	Ubiquitin carboxyl-terminal hydrolase 48 OS=Homo sapiens GN=USP48 PE=1	sp|Q86UV5|UBP48_HUMAN	3.2	1	0.912	0.8588
2064	ATP synthase mitochondrial F1 complex assembly factor 1 OS=Homo sapiens GN=ATPAF1 PE=1 SV=1	sp|Q5TC12|ATPF1_HUMAN	12.2	2	1.028	0.9489
2065	Syntaxin-binding protein 5 OS=Homo sapiens GN=STXBP5 PE=1 SV=1	sp|Q5T5C0|STXB5_HUMAN	2.7	1		
2066	N-alpha-acetyltransferase 50 OS=Homo sapiens GN=NAA50 PE=1 SV=1	sp|Q9GZZ1|NAA50_HUMAN	17.2	1	0.9204	0.8769
2067	Thioredoxin-related transmembrane protein 4 OS=Homo sapiens GN=TMX4 PE=1	sp|Q9H1E5|TMX4_HUMAN	6.3	1	0.863	0.7834
2068	Transmembrane protein 263 OS=Homo sapiens GN=TMEM263 PE=1 SV=1	sp|Q8WUH6|TM263_HUMAN	25	1	0.9817	0.9846
2069	Homeobox-containing protein 1 OS=Homo sapiens GN=HMBOX1	sp|Q6NT76|HMBX1_HUMAN	5.2	2	0.9638	0.9469
2070	DNA polymerase epsilon catalytic subunit A OS=Homo sapiens GN=POLE PE=1	sp|Q07864|DPOE1_HUMAN	3.8	1	0.9204	0.8852
2071	Acetyl-CoA carboxylase 2 OS=Homo sapiens GN=ACACB PE=1 SV=3	sp|O00763|ACACB_HUMAN	3.1	1	0.9204	0.8726

2072	Lon protease homolog 2, peroxisomal OS=Homo sapiens GN=LONP2 PE=1 SV=1	sp|Q86WA8|LONP2_HUMAN	8	2	1.1588	0.7586
2073	ELKS/Rab6-interacting/CAST family member 1 OS=Homo sapiens GN=ERC1 PE=1 SV=1	sp|Q8IUD2|RB6I2_HUMAN	5	1	0.9817	0.9776
2074	Zinc finger protein 638 OS=Homo sapiens GN=ZNF638 PE=1 SV=2	sp|Q14966|ZN638_HUMAN	3.5	2	0.9817	0.9894
2075	Nucleolar complex protein 2 homolog OS=Homo sapiens GN=NOC2L PE=1 SV=4	sp|Q9Y3T9|NOC2L_HUMAN	8.4	1	0.9462	0.9018
2076	OTU domain-containing protein 4 OS=Homo sapiens GN=OTUD4 PE=1	sp|Q01804|OTUD4_HUMAN	4.4	1	0.8551	0.7687
2077	Crooked neck-like protein 1 OS=Homo sapiens GN=CRNKL1 PE=1 SV=4	sp|Q9BZJ0|CRNL1_HUMAN	8.5	3	0.8241	0.719
2078	Oxysterol-binding protein-related protein 11 OS=Homo sapiens GN=OSBPL11 PE=1 SV=2	sp|Q9BXB4|OSB11_HUMAN	7.5	1	1.028	0.9412
2079	OTU domain-containing protein 7A OS=Homo sapiens GN=OTUD7A PE=1 SV=1	sp|Q8TE49|OTU7A_HUMAN	7.6	2	1	0.9425
2080	Ras guanyl-releasing protein 3 OS=Homo sapiens GN=RASGRP3 PE=1 SV=1	sp|Q8IV61|GRP3_HUMAN	5.8	1	0.6427	0.4712
2081	ATP-binding cassette sub-family D member 1 OS=Homo sapiens GN=ABCD1 PE=1 SV=2	sp|P33897|ABCD1_HUMAN	5.5	1	1.0864	0.8539
2082	Rap guanine nucleotide exchange factor 2 OS=Homo sapiens GN=RAPGEF2 PE=1 SV=1	sp|Q9Y4G8|RPGF2_HUMAN	3.9	1	1	0.9772
2083	Exocyst complex component 7 OS=Homo sapiens GN=EXOC7 PE=1 SV=3	sp|Q9UPT5|EXOC7_HUMAN	6	1	0.863	0.7508
2084	tRNA-dihydrouridine(47) synthase [NAD(P)(+)]-like OS=Homo sapiens GN=DUS3L PE=1 SV=2	sp|Q96G46|DUS3L_HUMAN	9.1	3	0.9036	0.8869
2085	E3 ubiquitin-protein ligase UBR1 OS=Homo sapiens GN=UBR1 PE=1 SV=1	sp|Q8IWV7|UBR1_HUMAN	3.2	2	0.9908	0.9986
2086	CREB-regulated transcription coactivator 2 OS=Homo sapiens GN=CRTC2 PE=1 SV=2	sp|Q53ET0|CRTC2_HUMAN	6.6	2	0.9462	0.9075

2087	AP2-associated protein kinase 1 OS=Homo sapiens GN=AAK1 PE=1 SV=3	sp|Q2M2I8|AAK1_HUMAN	6.9	1	1.0375	0.8812
2088	1-phosphatidylinositol 4,5-bisphosphate phosphodiesterase beta-4 OS=Homo sapiens GN=PLCB4 PE=1	sp|Q15147|PLCB4_HUMAN	4.3	1	1.0375	0.8839
2089	Sarcolemmal membrane-associated protein OS=Homo sapiens GN=SLMAP PE=1 SV=1	sp|Q14BN4|SLMAP_HUMAN	8.1	1	1.4723	0.4818
2090	REST corepressor 3 OS=Homo sapiens GN=RCOR3 PE=1	sp|Q9P2K3|RCOR3_HUMAN	13.1	3		
2091	Phosphatidylinositol 4-phosphate 5-kinase type-1 alpha OS=Homo sapiens GN=PIP5K1A PE=1 SV=1	sp|Q99755|PI51A_HUMAN	9.1	1	1.0093	0.9927
2092	COP9 signalosome complex subunit 1 OS=Homo sapiens GN=GPS1 PE=1 SV=4	sp|Q13098|CSN1_HUMAN	10.8	1	0.879	0.8051
2093	Mitogen-activated protein kinase kinase kinase kinase 5 OS=Homo sapiens GN=MAP4K5	sp|Q9Y4K4|M4K5_HUMAN	5	1	0.9908	0.9963
2094	Nucleotide exchange factor SIL1 OS=Homo sapiens GN=SIL1 PE=1 SV=1	sp|Q9H173|SIL1_HUMAN	12.2	1	1.1272	0.7698
2095	DnaJ homolog subfamily C member 1 OS=Homo sapiens GN=DNAJC1 PE=1 SV=1	sp|Q96KC8|DNJC1_HUMAN	7.6	2	1.3062	0.6106
2096	N-acylneuraminate cytidylyltransferase OS=Homo sapiens GN=CMAS PE=1 SV=2	sp|Q8NFW8|NEUA_HUMAN	7.6	1	1.1376	0.774
2097	6-phosphofructo-2-kinase/fructose-2,6-bisphosphatase 4 OS=Homo sapiens GN=PFKFB4 PE=2 SV=6	sp|Q16877|F264_HUMAN	8.7	1	1.1066	0.8147
2098	Zinc finger CCCH domain-containing protein 11A OS=Homo sapiens GN=ZC3H11A PE=1 SV=3	sp|O75152|ZC11A_HUMAN	4.3	2	1.0666	0.8823
2099	Serine protease HTRA2, mitochondrial OS=Homo sapiens GN=HTRA2 PE=1 SV=2	sp|O43464|HTRA2_HUMAN	8.3	1	0.9908	0.9801
2100	REVERSED E3 ISG15--protein ligase HERC5 OS=Homo sapiens GN=HERC5 PE=1 SV=2	RRRRRsp|Q9UII4|HERC5_HUMAN	3.6	1		
2101	Vesicle transport protein USE1 OS=Homo sapiens GN=USE1 PE=1 SV=2	sp|Q9NZ43|USE1_HUMAN	15.8	1	0.9462	0.4468

2102	Ribonuclease 3 OS=Homo sapiens GN=DROSHA PE=1 SV=2	sp|Q9NRR4|RNC_HUMAN	1.9	1	0.8395	0.7317
2103	WD repeat-containing protein 13 OS=Homo sapiens GN=WDR13 PE=1	sp|Q9H1Z4|WDR13_HUMAN	8.2	1	1.1912	0.7196
2104	Oxysterol-binding protein-related protein 9 OS=Homo sapiens GN=OSBPL9 PE=1 SV=2	sp|Q96SU4|OSBL9_HUMAN	5.3	1	1.0666	0.8785
2105	Protein FAM63B OS=Homo sapiens GN=FAM63B PE=1 SV=2	sp|Q8NBR6|FA63B_HUMAN	4.8	1	1.3062	0.6045
2106	Retinoblastoma-binding protein 5 OS=Homo sapiens GN=RBBP5 PE=1 SV=2	sp|Q15291|RBBP5_HUMAN	8.4	1	0.879	0.8045
2107	Argininosuccinate lyase OS=Homo sapiens GN=ASL PE=1 SV=4	sp|P04424|ARLY_HUMAN	9.1	1	2.0701	0.4121
2108	Toll-like receptor 2 OS=Homo sapiens GN=TLR2 PE=1 SV=1	sp|O60603|TLR2_HUMAN	5.6	2	1.2023	0.7416
2109	Mitochondrial ribonuclease P protein 3 OS=Homo sapiens GN=KIAA0391 PE=1 SV=2	sp|O15091|MRRP3_HUMAN	5.5	1	1.028	0.9413
2110	UPF0609 protein C4orf27 OS=Homo sapiens GN=C4orf27 PE=1 SV=2	sp|Q9NWY4|CD027_HUMAN	10.4	1	1	0.9846
2111	Large subunit GTPase 1 homolog OS=Homo sapiens GN=LSG1 PE=1 SV=2	sp|Q9H089|LSG1_HUMAN	6.5	1	0.9817	0.9882
2112	Uncharacterized protein CXorf57 OS=Homo sapiens GN=CXorf57 PE=1 SV=2	sp|Q6NSI4|CX057_HUMAN	3.5	1	1.0093	0.9647
2113	U2 small nuclear ribonucleoprotein auxiliary factor 35 kDa subunit-related protein 2 OS=Homo sapiens GN=ZRSR2 PE=1	sp|Q15696|U2AFM_HUMAN	5.2	1	0.929	0.8854
2114	Peptidyl-prolyl cis-trans isomerase NIMA-interacting 1 OS=Homo sapiens GN=PIN1 PE=1 SV=1	sp|Q13526|PIN1_HUMAN	20.9	1	0.8241	0.7097
2115	40S ribosomal protein S26 OS=Homo sapiens GN=RPS26 PE=1 SV=3	sp|P62854|RS26_HUMAN	33	2	0.8318	0.6052
2116	Receptor-type tyrosine-protein phosphatase alpha OS=Homo sapiens GN=PTPRA PE=1 SV=2	sp|P18433|PTPRA_HUMAN	4	1	0.9908	0.9943
2117	Choline/ethanolaminephosphotransferase 1 OS=Homo sapiens GN=CEPT1 PE=1	sp|Q9Y6K0|CEPT1_HUMAN	7	1	1.1376	0.7305

2118	Cell growth-regulating nucleolar protein OS=Homo sapiens GN=LYAR PE=1	sp|Q9NX58|LYAR_HUMAN	8.2	2	0.7943	0.6749
2119	Growth hormone-inducible transmembrane protein OS=Homo sapiens GN=GHITM PE=1 SV=2	sp|Q9H3K2|GHITM_HUMAN	12.5	5	1.5996	0.522
2120	Protein-L-isoaspartate O-methyltransferase domain-containing protein 1 OS=Homo sapiens GN=PCMTD1 PE=2 SV=2	sp|Q96MG8|PCMD1_HUMAN	13.7	1	1.1376	0.7776
2121	N-terminal kinase-like protein OS=Homo sapiens GN=SCYL1 PE=1 SV=1	sp|Q96KG9|NTKL_HUMAN	4	3	1.0568	0.8903
2122	Histone-lysine N-methyltransferase setd3 OS=Homo sapiens GN=SETD3 PE=1	sp|Q86TU7|SETD3_HUMAN	4.2	1	0.8872	0.83
2123	B-cell receptor-associated protein 31 OS=Homo sapiens GN=BCAP31	sp|P51572|BAP31_HUMAN	10.6	1	1.0568	0.892
2124	Annexin A4 OS=Homo sapiens GN=ANXA4 PE=1 SV=4	sp|P09525|ANXA4_HUMAN	10	2		
2125	Phosphoacetylglucosamine mutase OS=Homo sapiens GN=PGM3 PE=1 SV=1	sp|O95394|AGM1_HUMAN	2.8	1	1.0965	0.8398
2126	Interferon-inducible double-stranded RNA-dependent protein kinase activator A OS=Homo sapiens GN=PRKRA PE=1 SV=1	sp|O75569|PRKRA_HUMAN	10.9	1	1.0186	0.9392
2127	Polyglutamine-binding protein 1 OS=Homo sapiens GN=PQBP1 PE=1 SV=1	sp|O60828|PQBP1_HUMAN	8.3	1	0.9036	0.8511
2128	REVERSED Cancer-associated gene 1 protein OS=Homo sapiens GN=CAGE1 PE=1 SV=2	RRRRRsp|Q8TC20|CAGE1_HUMAN	3.2	1	1.3932	0.5452
2129	Glycerophosphodiester phosphodiesterase 1 OS=Homo sapiens GN=GDE1 PE=1 SV=1	sp|Q9NZC3|GDE1_HUMAN	5.1	1	0.9817	0.9834
2130	Tubulin-folding cofactor B OS=Homo sapiens GN=TBCB PE=1 SV=2	sp|Q99426|TBCB_HUMAN	7.4	3	0.871	0.7906
2131	Chromosome alignment-maintaining phosphoprotein 1 OS=Homo sapiens GN=CHAMP1 PE=1 SV=2	sp|Q96JM3|CHAP1_HUMAN	3.9	2	0.912	0.8651

2132	StAR-related lipid transfer protein 4 OS=Homo sapiens GN=STARD4 PE=2 SV=1	sp|Q96DR4|STAR4_HUMAN	16.1	2	0.912	0.9866
2133	Isochorismatase domain-containing protein 1 OS=Homo sapiens GN=ISOC1 PE=1	sp|Q96CN7|ISOC1_HUMAN	6.7	1	0.9727	0.9592
2134	Probable ATP-dependent RNA helicase DHX58 OS=Homo sapiens GN=DHX58 PE=1 SV=1	sp|Q96C10|DHX58_HUMAN	2.5	1	1.2942	0.6
2135	Bifunctional lysine-specific demethylase and histidyl-hydroxylase MINA OS=Homo sapiens GN=MINA PE=1	sp|Q8IUF8|MINA_HUMAN	3.4	1	0.9036	0.8584
2136	ER lumen protein-retaining receptor 1 OS=Homo sapiens GN=KDELR1 PE=1 SV=1	sp|P24390|ERD21_HUMAN	13.2	1	1.0765	0.841
2137	Secretory carrier-associated membrane protein 3 OS=Homo sapiens GN=SCAMP3	sp|O14828|SCAM3_HUMAN	8.1	1	1.2246	0.6858
2138	Costars family protein ABRACL OS=Homo sapiens GN=ABRACL PE=1 SV=1	sp|Q9P1F3|ABRAL_HUMAN	35.8	2	1.1169	0.8044
2139	E3 ubiquitin-protein ligase RNF126 OS=Homo sapiens GN=RNF126 PE=1	sp|Q9BV68|RN126_HUMAN	6.7	1	1.0093	0.9678
2140	Phosphatidylinositol 4-kinase type 2-alpha OS=Homo sapiens GN=PI4K2A PE=1 SV=1	sp|Q9BTU6|P4K2A_HUMAN	4.8	1	1.028	0.9365
2141	SUMO-conjugating enzyme UBC9 OS=Homo sapiens GN=UBE2I PE=1 SV=1	sp|P63279|UBC9_HUMAN	22.8	6	1.2359	0.6506
2142	U6 snRNA-associated Sm-like protein LSm6 OS=Homo sapiens GN=LSM6 PE=1 SV=1	sp|P62312|LSM6_HUMAN	12.5	2	0.7311	0.5774
2143	Translocon-associated protein subunit delta OS=Homo sapiens GN=SSR4 PE=1 SV=1	sp|P51571|SSRD_HUMAN	13.3	1	1.028	0.9382
2144	Survival of motor neuron-related-splicing factor 30 OS=Homo sapiens GN=SMNDC1 PE=1 SV=1	sp|O75940|SPF30_HUMAN	13	1	1	0.9911
2145	Cadherin EGF LAG seven-pass G-type receptor 1 OS=Homo sapiens GN=CELSR1 PE=1 SV=1	sp|Q9NYQ6|CELR1_HUMAN	2.3	1	0.9817	0.9821

2146	Serine/threonine-protein kinase PRP4 homolog OS=Homo sapiens GN=PRPF4B PE=1 SV=3	sp|Q13523|PRP4B_HUMAN	7.2	1	1.2823	0.6262
2147	Protein PRRC2A OS=Homo sapiens GN=PRRC2A PE=1 SV=3	sp|P48634|PRC2A_HUMAN	2.8	1	1.2023	0.7071
2148	HEAT repeat-containing protein 5A OS=Homo sapiens GN=HEATR5A PE=1 SV=2	sp|Q86XA9|HTR5A_HUMAN	2.3	1	1.0375	0.9352
2149	Lysophospholipid acyltransferase LPCAT4 OS=Homo sapiens GN=LPCAT4 PE=1 SV=1	sp|Q643R3|LPCT4_HUMAN	7.4	1	0.9638	0.9538
2150	Attractin OS=Homo sapiens GN=ATRN PE=1	sp|O75882|ATRN_HUMAN	5.2	1	1.2706	0.6433
2151	ATP-binding cassette sub-family B member 7, mitochondrial OS=Homo sapiens GN=ABCB7 PE=1 SV=2	sp|O75027|ABCB7_HUMAN	10.8	2	1.0864	0.85
2152	Protein TANC2 OS=Homo sapiens GN=TANC2 PE=1 SV=3	sp|Q9HCD6|TANC2_HUMAN	2	1	1.0471	0.9122
2153	Protein KIAA0100 OS=Homo sapiens GN=KIAA0100 PE=1 SV=3	sp|Q14667|K0100_HUMAN	2.3	1	0.879	0.8052
2154	Myotubularin-related protein 5 OS=Homo sapiens GN=SBF1 PE=1 SV=3	sp|O95248|MTMR5_HUMAN	3.2	1	1.0186	0.9478
2155	Probable phospholipid-transporting ATPase IIB OS=Homo sapiens GN=ATP9B PE=2	sp|O43861|ATP9B_HUMAN	3.7	1	1.2474	0.5507
2156	SWI/SNF-related matrix-associated actin-dependent regulator of chromatin subfamily A containing DEAD/H box 1 OS=Homo sapiens GN=SMARCAD1 PE=1 SV=2	sp|Q9H4L7|SMRCD_HUMAN	5.1	1	0.7447	0.6031
2157	Cell division cycle and apoptosis regulator protein 1 OS=Homo sapiens GN=CCAR1 PE=1 SV=2	sp|Q8IX12|CCAR1_HUMAN	4	1	1.0186	0.9557
2158	Mediator of RNA polymerase II transcription subunit 14 OS=Homo sapiens GN=MED14 PE=1	sp|O60244|MED14_HUMAN	3.1	1	0.8472	0.7535
2159	TSC22 domain family protein 4 OS=Homo sapiens GN=TSC22D4 PE=1 SV=2	sp|Q9Y3Q8|T22D4_HUMAN	7.6	1	0.8395	0.7155

2160	Anaphase-promoting complex subunit 7 OS=Homo sapiens GN=ANAPC7 PE=1 SV=4	sp|Q9UJX3|APC7_HUMAN	8.2	1	0.8472	0.7592
2161	Protein capicua homolog OS=Homo sapiens GN=CIC PE=1	sp|Q96RK0|CIC_HUMAN	2.4	1	1.0471	0.9049
2162	Ataxin-2-like protein OS=Homo sapiens GN=ATXN2L	sp|Q8WWM7|ATX2L_HUMAN	3.9	2	0.879	0.8102
2163	Wings apart-like protein homolog OS=Homo sapiens GN=WAPAL PE=1 SV=1	sp|Q7Z5K2|WAPL_HUMAN	4.3	1	0.8091	0.6729
2164	Rab11 family-interacting protein 1 OS=Homo sapiens GN=RAB11FIP1	sp|Q6WKZ4|RFIP1_HUMAN	5	1	1.2706	0.6351
2165	Dipeptidyl peptidase 8 OS=Homo sapiens GN=DPP8 PE=1	sp|Q6V1X1|DPP8_HUMAN	6.2	1	1.0471	0.9059
2166	Roquin-1 OS=Homo sapiens GN=RC3H1 PE=1	sp|Q5TC82|RC3H1_HUMAN	4.9	1	0.9204	0.8819
2167	Early endosome antigen 1 OS=Homo sapiens GN=EEA1 PE=1	sp|Q15075|EEA1_HUMAN	4	1	1.2589	0.6535
2168	Treacle protein OS=Homo sapiens GN=TCOF1 PE=1 SV=3	sp|Q13428|TCOF_HUMAN	2.8	1	1.1066	0.8131
2169	DNA excision repair protein ERCC-6 OS=Homo sapiens GN=ERCC6 PE=1	sp|Q03468|ERCC6_HUMAN	2.7	1	1.028	0.9389
2170	E3 ubiquitin-protein ligase BRE1B OS=Homo sapiens GN=RNF40 PE=1	sp|O75150|BRE1B_HUMAN	4.6	1		
2171	H/ACA ribonucleoprotein complex subunit 4 OS=Homo sapiens GN=DKC1 PE=1 SV=3	sp|O60832|DKC1_HUMAN	13.8	1	0.7943	0.8864
2172	Zinc finger protein 451 OS=Homo sapiens GN=ZNF451 PE=1 SV=2	sp|Q9Y4E5|ZN451_HUMAN	2.9	1	0.7447	0.5977
2173	Protein AATF OS=Homo sapiens GN=AATF PE=1 SV=1	sp|Q9NY61|AATF_HUMAN	11.4	3	1.1272	0.7953
2174	Ribonucleases P/MRP protein subunit POP1 OS=Homo sapiens GN=POP1 PE=1 SV=2	sp|Q99575|POP1_HUMAN	4.3	1	0.9204	0.8843
2175	Sorting nexin-27 OS=Homo sapiens GN=SNX27 PE=1 SV=2	sp|Q96L92|SNX27_HUMAN	5.5	1	1.0375	0.866
2176	Sec1 family domain-containing protein 1 OS=Homo sapiens GN=SCFD1 PE=1	sp|Q8WVM8|SCFD1_HUMAN	7.9	1	0.9727	0.9534
2177	Autophagy-related protein 9A OS=Homo sapiens GN=ATG9A PE=1 SV=3	sp|Q7Z3C6|ATG9A_HUMAN	4.5	1	1.3183	0.6015

2178	E3 ubiquitin-protein ligase TRIM32 OS=Homo sapiens GN=TRIM32 PE=1	sp|Q13049|TRI32_HUMAN	8.4	1	0.9036	0.7765
2179	Focal adhesion kinase 1 OS=Homo sapiens GN=PTK2 PE=1 SV=2	sp|Q05397|FAK1_HUMAN	5	1	1.1066	0.8208
2180	Growth factor receptor-bound protein 2 OS=Homo sapiens GN=GRB2 PE=1	sp|P62993|GRB2_HUMAN	23	1	1.0093	0.9592
2181	60 kDa SS-A/Ro ribonucleoprotein OS=Homo sapiens GN=TROVE2 PE=1 SV=2	sp|P10155|RO60_HUMAN	5.6	1	0.8472	0.7484
2182	Putative uncharacterized protein FRMD6-AS1 OS=Homo sapiens GN=FRMD6-AS1	sp|P0C7T7|FMAS1_HUMAN	12.4	1	1.0765	0.9484
2183	Serum albumin OS=Homo sapiens GN=ALB PE=1 SV=2	sp|P02768|ALBU_HUMAN	11.8	3	0.1854	0.1454
2184	Mediator of RNA polymerase II transcription subunit 24 OS=Homo sapiens GN=MED24 PE=1	sp|O75448|MED24_HUMAN	4.6	1	0.9036	0.7919
2185	Glycosylphosphatidylinositol anchor attachment 1 protein OS=Homo sapiens GN=GPAA1 PE=1	sp|O43292|GPAA1_HUMAN	6.3	1	0.9908	0.9829
2186	Epidermal growth factor receptor substrate 15-like 1 OS=Homo sapiens GN=EPS15L1 PE=1 SV=1	sp|Q9UBC2|EP15R_HUMAN	4.4	1	1.0093	0.9737
2187	Thymocyte nuclear protein 1 OS=Homo sapiens GN=THYN1 PE=1 SV=1	sp|Q9P016|THYN1_HUMAN	20.9	1	0.8551	0.7637
2188	Rho guanine nucleotide exchange factor 10-like protein OS=Homo sapiens GN=ARHGEF10L	sp|Q9HCE6|ARGAL_HUMAN	2.9	1	0.929	0.9244
2189	Echinoderm microtubule-associated protein-like 4 OS=Homo sapiens GN=EML4 PE=1 SV=3	sp|Q9HC35|EMAL4_HUMAN	3.4	2	0.9462	0.9312
2190	Semaphorin-4C OS=Homo sapiens GN=SEMA4C PE=1 SV=2	sp|Q9C0C4|SEM4C_HUMAN	4.3	1	1.3428	0.5752
2191	Protein FAM118B OS=Homo sapiens GN=FAM118B PE=1 SV=1	sp|Q9BPY3|F118B_HUMAN	10.5	1	0.7311	0.5757
2192	Histone-lysine N-methyltransferase EHMT2 OS=Homo sapiens GN=EHMT2 PE=1 SV=3	sp|Q96KQ7|EHMT2_HUMAN	2.7	1	0.8017	0.6867
2193	F-box only protein 22 OS=Homo sapiens GN=FBXO22 PE=1 SV=1	sp|Q8NEZ5|FBX22_HUMAN	5.7	1	0.8472	0.7604

2194	Transmembrane protein 199 OS=Homo sapiens GN=TMEM199 PE=1 SV=1	sp|Q8N511|TM199_HUMAN	24	2	0.912	0.8553
2195	Nuclear fragile X mental retardation-interacting protein 2 OS=Homo sapiens GN=NUFIP2 PE=1	sp|Q7Z417|NUFP2_HUMAN	5.2	2	0.912	0.8412
2196	LisH domain-containing protein ARMC9 OS=Homo sapiens GN=ARMC9 PE=1 SV=2	sp|Q7Z3E5|ARMC9_HUMAN	3.8	1	1.4454	0.5083
2197	Protein SHQ1 homolog OS=Homo sapiens GN=SHQ1 PE=1	sp|Q6PI26|SHQ1_HUMAN	6.4	1	0.8472	0.7602
2198	Rap guanine nucleotide exchange factor 1 OS=Homo sapiens GN=RAPGEF1 PE=1 SV=3	sp|Q13905|RPGF1_HUMAN	3.4	1	1.1169	0.8043
2199	Stromal interaction molecule 1 OS=Homo sapiens GN=STIM1 PE=1 SV=3	sp|Q13586|STIM1_HUMAN	5.7	1	0.9817	0.9188
2200	28 kDa heat- and acid-stable phosphoprotein OS=Homo sapiens GN=PDAP1 PE=1 SV=1	sp|Q13442|HAP28_HUMAN	23.2	3	0.9727	0.9184
2201	Hydroxymethylglutaryl-CoA synthase, cytoplasmic OS=Homo sapiens GN=HMGCS1 PE=1 SV=2	sp|Q01581|HMCS1_HUMAN	5.8	1	0.5808	0.4061
2202	Calcium-transporting ATPase type 2C member 1 OS=Homo sapiens GN=ATP2C1 PE=1	sp|P98194|AT2C1_HUMAN	4.6	1	1	0.9532
2203	Adenylate cyclase type 7 OS=Homo sapiens GN=ADCY7 PE=2 SV=1	sp|P51828|ADCY7_HUMAN	3.1	1	1.0864	0.7822
2204	Protein ERGIC-53 OS=Homo sapiens GN=LMAN1 PE=1 SV=2	sp|P49257|LMAN1_HUMAN	11.4	1		
2205	DNA mismatch repair protein Mlh1 OS=Homo sapiens GN=MLH1 PE=1 SV=1	sp|P40692|MLH1_HUMAN	4.1	1	0.9036	0.8551
2206	Propionyl-CoA carboxylase alpha chain, mitochondrial OS=Homo sapiens GN=PCCA PE=1	sp|P05165|PCCA_HUMAN	3.7	1	1.0765	0.8663
2207	Protein strawberry notch homolog 1 OS=Homo sapiens GN=SBNO1 PE=1 SV=1	sp|A3KN83|SBNO1_HUMAN	2.9	1	0.8551	0.7459
2208	Charged multivesicular body protein 3 OS=Homo sapiens GN=CHMP3 PE=1 SV=3	sp|Q9Y3E7|CHMP3_HUMAN	11.3	1	0.955	0.8718

2209	Thioredoxin-related transmembrane protein 2 OS=Homo sapiens GN=TMX2 PE=1	sp|Q9Y320|TMX2_HUMAN	8.4	2	0.8872	0.6944
2210	Cell division cycle protein 23 homolog OS=Homo sapiens GN=CDC23 PE=1 SV=3	sp|Q9UJX2|CDC23_HUMAN	4	1	1.028	0.9323
2211	Peptidyl-prolyl cis-trans isomerase FKBP11 OS=Homo sapiens GN=FKBP11 PE=1 SV=1	sp|Q9NYL4|FKB11_HUMAN	14.4	1	1.2942	0.6229
2212	Ras association domain-containing protein 1 OS=Homo sapiens GN=RASSF1 PE=1 SV=1	sp|Q9NS23|RASF1_HUMAN	8.1	1		
2213	RNA polymerase II-associated protein 3 OS=Homo sapiens GN=RPAP3 PE=1	sp|Q9H6T3|RPAP3_HUMAN	5.1	1		
2214	Vacuolar protein sorting-associated protein 11 homolog OS=Homo sapiens GN=VPS11 PE=1 SV=1	sp|Q9H270|VPS11_HUMAN	3.4	1	0.955	0.9436
2215	Protein unc-93 homolog B1 OS=Homo sapiens GN=UNC93B1 PE=1 SV=2	sp|Q9H1C4|UN93B_HUMAN	4.2	1	1.2134	0.7777
2216	Ubiquitin-like protein 7 OS=Homo sapiens GN=UBL7 PE=1	sp|Q96S82|UBL7_HUMAN	10.8	1	1.2359	0.6809
2217	U5 small nuclear ribonucleoprotein 40 kDa protein OS=Homo sapiens GN=SNRNP40 PE=1 SV=1	sp|Q96DI7|SNR40_HUMAN	14.9	2	0.9908	0.9993
2218	HLA class I histocompatibility antigen, Cw-17 alpha chain OS=Homo sapiens GN=HLA-C PE=1 SV=1	sp|Q95604|1C17_HUMAN	7.5	1	1.1803	0.7367
2219	Transmembrane protein 131 OS=Homo sapiens GN=TMEM131 PE=1 SV=3	sp|Q92545|TM131_HUMAN	1.3	1	1.0965	0.8442
2220	Putative vomeronasal receptor-like protein 4 OS=Homo sapiens GN=VN1R17P	sp|Q8TDU5|VNRL4_HUMAN	8.7	1		
2221	Melanoma-associated antigen C3 OS=Homo sapiens GN=MAGEC3 PE=1 SV=1	sp|Q8TD91|MAGC3_HUMAN	4.4	1	0.5152	0.4827
2222	Solute carrier family 25 member 40 OS=Homo sapiens GN=SLC25A40 PE=2 SV=1	sp|Q8TBP6|S2540_HUMAN	10.4	1	1.1695	0.6997
2223	Lysophosphatidylcholine acyltransferase 1 OS=Homo sapiens GN=LPCAT1 PE=1 SV=2	sp|Q8NF37|PCAT1_HUMAN	6.6	1	0.955	0.9347

2224	Liprin-beta-2 OS=Homo sapiens GN=PPFIBP2 PE=1 SV=3	sp|Q8ND30|LIPB2_HUMAN	3.4	2	1.406	0.5326
2225	Tetratricopeptide repeat protein 13 OS=Homo sapiens GN=TTC13 PE=2 SV=3	sp|Q8NBP0|TTC13_HUMAN	4.3	1	0.912	0.8722
2226	Late secretory pathway protein AVL9 homolog OS=Homo sapiens GN=AVL9 PE=1 SV=1	sp|Q8NBF6|AVL9_HUMAN	4.6	1	1.2134	0.6987
2227	Activating signal cointegrator 1 complex subunit 1 OS=Homo sapiens GN=ASCC1 PE=1 SV=1	sp|Q8N9N2|ASCC1_HUMAN	10	1	1.0965	0.8126
2228	Aldehyde dehydrogenase family 16 member A1 OS=Homo sapiens GN=ALDH16A1 PE=1 SV=2	sp|Q8IZ83|A16A1_HUMAN	3.2	1	1.0568	0.8947
2229	Trafficking protein particle complex subunit 5 OS=Homo sapiens GN=TRAPPC5 PE=1 SV=1	sp|Q8IUR0|TPPC5_HUMAN	28.2	4	0.7112	0.5504
2230	CREB-regulated transcription coactivator 1 OS=Homo sapiens GN=CRTC1 PE=1 SV=2	sp|Q6UUV9|CRTC1_HUMAN	5.4	1	0.8318	0.7124
2231	Tetratricopeptide repeat protein 38 OS=Homo sapiens GN=TTC38 PE=1 SV=1	sp|Q5R3I4|TTC38_HUMAN	4.7	1	1.0375	0.9082
2232	Deoxynucleotidyltransferase terminal-interacting protein 2 OS=Homo sapiens GN=DNTTIP2	sp|Q5QJE6|TDIF2_HUMAN	4.6	2	1	0.9762
2233	Glucoside xylosyltransferase 1 OS=Homo sapiens GN=GXYLT1 PE=1 SV=2	sp|Q4G148|GXLT1_HUMAN	5	1	0.955	0.9641
2234	Macrophage-expressed gene 1 protein OS=Homo sapiens GN=MPEG1 PE=2 SV=1	sp|Q2M385|MPEG1_HUMAN	4.6	1	7.4473	0.2847
2235	Methenyltetrahydrofolate synthase domain-containing protein OS=Homo sapiens GN=MTHFSD PE=1 SV=2	sp|Q2M296|MTHSD_HUMAN	6.5	1	0.8472	0.7534
2236	CD166 antigen OS=Homo sapiens GN=ALCAM PE=1 SV=2	sp|Q13740|CD166_HUMAN	6	1	1.977	0.485
2237	Peptidyl-prolyl cis-trans isomerase G OS=Homo sapiens GN=PPIG PE=1 SV=2	sp|Q13427|PPIG_HUMAN	4.2	1		
2238	DDB1- and CUL4-associated factor 7 OS=Homo sapiens GN=DCAF7 PE=1 SV=1	sp|P61962|DCAF7_HUMAN	12.6	1	1.1066	0.8225

2239	RNA polymerase II elongation factor ELL OS=Homo sapiens GN=ELL PE=1 SV=1	sp|P55199|ELL_HUMAN	4.8	1	0.7656	0.6122
2240	Glutathione synthetase OS=Homo sapiens GN=GSS PE=1 SV=1	sp|P48637|GSHB_HUMAN	5.7	1	0.912	0.8683
2241	40S ribosomal protein S10 OS=Homo sapiens GN=RPS10 PE=1 SV=1	sp|P46783|RS10_HUMAN	15.2	2	0.8017	0.7501
2242	Mitogen-activated protein kinase 8 OS=Homo sapiens GN=MAPK8 PE=1 SV=2	sp|P45983|MK08_HUMAN	8	1	0.8472	0.7436
2243	Delta-1-pyrroline-5-carboxylate dehydrogenase, mitochondrial OS=Homo sapiens GN=ALDH4A1 PE=1 SV=3	sp|P30038|AL4A1_HUMAN	6.4	1	1.1803	0.7314
2244	Beta-galactosidase OS=Homo sapiens GN=GLB1 PE=1 SV=2	sp|P16278|BGAL_HUMAN	5.3	1	1.0568	0.8919
2245	Golgi SNAP receptor complex member 1 OS=Homo sapiens GN=GOSR1 PE=1 SV=1	sp|O95249|GOSR1_HUMAN	10.4	1	1.0965	0.8689
2246	Eukaryotic translation initiation factor 3 subunit G OS=Homo sapiens GN=EIF3G PE=1	sp|O75821|EIF3G_HUMAN	8.4	1	1.1066	0.821
2247	Cell division cycle protein 123 homolog OS=Homo sapiens GN=CDC123 PE=1	sp|O75794|CD123_HUMAN	6	1	0.3698	0.3413
2248	PRA1 family protein 2 OS=Homo sapiens GN=PRAF2 PE=1	sp|O60831|PRAF2_HUMAN	18	6	1.1912	0.6867
2249	Mannosyl-oligosaccharide 1,2-alpha-mannosidase IB OS=Homo sapiens GN=MAN1A2 PE=1 SV=1	sp|O60476|MA1A2_HUMAN	5.6	1	0.9204	0.8806
2250	U3 small nucleolar ribonucleoprotein protein MPP10 OS=Homo sapiens GN=MPHOSPH10 PE=1 SV=2	sp|O00566|MPP10_HUMAN	6	3	1.0093	0.9938
2251	Ubiquinone biosynthesis protein COQ4 homolog, mitochondrial OS=Homo sapiens GN=COQ4 PE=1	sp|Q9Y3A0|COQ4_HUMAN	10.2	1	1.0093	0.944
2252	Nitric oxide synthase-interacting protein OS=Homo sapiens GN=NOSIP PE=1	sp|Q9Y314|NOSIP_HUMAN	8.3	1	0.5598	0.4253
2253	Lysosomal thioesterase PPT2 OS=Homo sapiens GN=PPT2 PE=1 SV=4	sp|Q9UMR5|PPT2_HUMAN	10.6	1	1.3804	0.6771

2254	ADP-sugar pyrophosphatase OS=Homo sapiens GN=NUDT5 PE=1 SV=1	sp|Q9UKK9|NUDT5_HUMAN	14.2	2	0.8091	0.7084
2255	Glyoxylate reductase/hydroxypyruvate reductase OS=Homo sapiens GN=GRHPR PE=1 SV=1	sp|Q9UBQ7|GRHPR_HUMAN	10.4	2	0.7798	0.6556
2256	Glioma tumor suppressor candidate region gene 2 protein OS=Homo sapiens GN=GLTSCR2 PE=1 SV=2	sp|Q9NZM5|GSCR2_HUMAN	7.3	1		
2257	Poly(ADP-ribose) glycohydrolase ARH3 OS=Homo sapiens GN=ADPRHL2 PE=1 SV=1	sp|Q9NX46|ARHL2_HUMAN	6.1	1	1.0186	0.7858
2258	Beta-parvin OS=Homo sapiens GN=PARVB PE=1 SV=1	sp|Q9HBI1|PARVB_HUMAN	6.3	1	1.1169	0.7385
2259	UPF0160 protein MYG1, mitochondrial OS=Homo sapiens GN=C12orf10 PE=1 SV=2	sp|Q9HB07|MYG1_HUMAN	7.4	2	1.0093	0.964
2260	Vacuolar protein sorting-associated protein 37B OS=Homo sapiens GN=VPS37B PE=1 SV=1	sp|Q9H9H4|VP37B_HUMAN	9.8	1	1.1482	0.7617
2261	Potassium channel subfamily K member 15 OS=Homo sapiens GN=KCNK15 PE=1 SV=2	sp|Q9H427|KCNKF_HUMAN	10.6	1	0.7798	0.6559
2262	Negative elongation factor A OS=Homo sapiens GN=NELFA PE=1	sp|Q9H3P2|NELFA_HUMAN	3.6	1	0.8954	0.8484
2263	Protein lunapark OS=Homo sapiens GN=LNP PE=1 SV=2	sp|Q9C0E8|LNP_HUMAN	5.6	1	0.9817	0.9797
2264	RUN and SH3 domain-containing protein 1 OS=Homo sapiens GN=RUSC1 PE=1	sp|Q9BVN2|RUSC1_HUMAN	2.9	1	0.8551	0.8089
2265	DNA replication complex GINS protein SLD5 OS=Homo sapiens GN=GINS4 PE=1 SV=1	sp|Q9BRT9|SLD5_HUMAN	13.5	1	1.3804	0.5497
2266	Tumor susceptibility gene 101 protein OS=Homo sapiens GN=TSG101 PE=1	sp|Q99816|TS101_HUMAN	6.2	1	0.9908	0.9948
2267	Sorting nexin-18 OS=Homo sapiens GN=SNX18 PE=1 SV=2	sp|Q96RF0|SNX18_HUMAN	5.7	1	1.0568	0.8965
2268	Transmembrane protein 68 OS=Homo sapiens GN=TMEM68 PE=2 SV=2	sp|Q96MH6|TMM68_HUMAN	7.4	1	1.028	0.9392
2269	Signal transducing adapter molecule 1 OS=Homo sapiens GN=STAM PE=1 SV=3	sp|Q92783|STAM1_HUMAN	8.7	2	1.4859	0.5898

2270	Zinc transporter 5 OS=Homo sapiens GN=SLC30A5 PE=1 SV=1	sp|Q8TAD4|ZNT5_HUMAN	3	1	1.0666	0.8766
2271	C-Maf-inducing protein OS=Homo sapiens GN=CMIP PE=1 SV=3	sp|Q8IY22|CMIP_HUMAN	2.8	1	0.9817	0.9777
2272	Hydroxyacid-oxoacid transhydrogenase, mitochondrial OS=Homo sapiens GN=ADHFE1 PE=1 SV=1	sp|Q8IWW8|HOT_HUMAN	4.9	1	1.406	0.5351
2273	ATP-dependent (S)-NAD(P)H-hydrate dehydratase OS=Homo sapiens GN=CARKD PE=1 SV=1	sp|Q8IW45|NNRD_HUMAN	6.9	1	1.0568	0.8736
2274	Probable peptidyl-tRNA hydrolase OS=Homo sapiens GN=PTRH1 PE=1 SV=1	sp|Q86Y79|PTH_HUMAN	12.2	1	0.871	0.7969
2275	Ankyrin repeat domain-containing protein 13D OS=Homo sapiens GN=ANKRD13D PE=1 SV=2	sp|Q6ZTN6|AN13D_HUMAN	4.8	1	0.9376	0.9142
2276	Autophagy-related protein 16-1 OS=Homo sapiens GN=ATG16L1 PE=1 SV=2	sp|Q676U5|A16L1_HUMAN	3	1	1.0568	0.875
2277	Protein FAM133B OS=Homo sapiens GN=FAM133B PE=1 SV=1	sp|Q5BKY9|F133B_HUMAN	9.7	1	0.7943	0.6661
2278	NGFI-A-binding protein 2 OS=Homo sapiens GN=NAB2 PE=1	sp|Q15742|NAB2_HUMAN	7.4	1	0.955	0.9045
2279	Transcription elongation factor B polypeptide 2 OS=Homo sapiens GN=TCEB2 PE=1 SV=1	sp|Q15370|ELOB_HUMAN	32.2	1	0.9462	0.9067
2280	Arf-GAP with coiled-coil, ANK repeat and PH domain-containing protein 2 OS=Homo sapiens GN=ACAP2 PE=1	sp|Q15057|ACAP2_HUMAN	4	1	0.879	0.8169
2281	Serine/arginine-rich splicing factor 11 OS=Homo sapiens GN=SRSF11 PE=1 SV=1	sp|Q05519|SRS11_HUMAN	4.5	1	0.9638	0.9569
2282	U7 snRNA-associated Sm-like protein LSm11 OS=Homo sapiens GN=LSM11 PE=1 SV=2	sp|P83369|LSM11_HUMAN	6.4	1	1.0375	0.9288
2283	mRNA export factor OS=Homo sapiens GN=RAE1 PE=1 SV=1	sp|P78406|RAE1L_HUMAN	9.8	1	0.929	0.8983
2284	Ras-related protein Rab-4B OS=Homo sapiens GN=RAB4B PE=1	sp|P61018|RAB4B_HUMAN	16	1	1.2246	0.6866
2285	Ras-related protein Rab-8A OS=Homo sapiens GN=RAB8A PE=1	sp|P61006|RAB8A_HUMAN	20.3	1	0.9376	0.9133

2286	Sorting nexin-16 OS=Homo sapiens GN=SNX16 PE=1 SV=2	sp|P57768|SNX16_HUMAN	9.6	1	1.2823	0.626
2287	Mismatch repair endonuclease PMS2 OS=Homo sapiens GN=PMS2 PE=1 SV=2	sp|P54278|PMS2_HUMAN	2.2	1	0.6855	0.5191
2288	Geranylgeranyl transferase type-1 subunit beta OS=Homo sapiens GN=PGGT1B PE=1 SV=2	sp|P53609|PGTB1_HUMAN	12.7	1	1.0093	0.9483
2289	Serine/threonine-protein kinase PLK1 OS=Homo sapiens GN=PLK1 PE=1 SV=1	sp|P53350|PLK1_HUMAN	6.6	1	0.4831	0.3584
2290	CCAAT/enhancer-binding protein delta OS=Homo sapiens GN=CEBPD PE=1	sp|P49716|CEBPD_HUMAN	8.2	1	1.4997	0.4844
2291	Serpin B6 OS=Homo sapiens GN=SERPINB6 PE=1 SV=3	sp|P35237|SPB6_HUMAN	8	1	1.0375	0.9203
2292	DnaJ homolog subfamily A member 1 OS=Homo sapiens GN=DNAJA1 PE=1 SV=2	sp|P31689|DNJA1_HUMAN	8.6	1	0.6546	0.4837
2293	GTP cyclohydrolase 1 OS=Homo sapiens GN=GCH1 PE=1 SV=1	sp|P30793|GCH1_HUMAN	11.2	2	4.2462	0.3164
2294	Transcription initiation factor IIE subunit beta OS=Homo sapiens GN=GTF2E2 PE=1 SV=1	sp|P29084|T2EB_HUMAN	8.2	1	0.8241	0.7005
2295	Tyrosine-protein kinase JAK1 OS=Homo sapiens GN=JAK1 PE=1 SV=2	sp|P23458|JAK1_HUMAN	2.9	1	1.2474	0.6616
2296	E3 ubiquitin-protein ligase CBL OS=Homo sapiens GN=CBL PE=1 SV=2	sp|P22681|CBL_HUMAN	2.8	1	0.929	0.8948
2297	Alpha-galactosidase A OS=Homo sapiens GN=GLA PE=1	sp|P06280|AGAL_HUMAN	5.4	1	1.2246	0.685
2298	Transcription factor AP-1 OS=Homo sapiens GN=JUN PE=1 SV=2	sp|P05412|JUN_HUMAN	8.8	1	1.3677	0.5614
2299	Superoxide dismutase [Mn], mitochondrial OS=Homo sapiens GN=SOD2 PE=1 SV=2	sp|P04179|SODM_HUMAN	12.2	1	2.0512	0.3542
2300	Flotillin-1 OS=Homo sapiens GN=FLOT1 PE=1	sp|O75955|FLOT1_HUMAN	6.3	1	1.4191	0.5286
2301	Protein tyrosine phosphatase type IVA 3 OS=Homo sapiens GN=PTP4A3 PE=1 SV=2	sp|O75365|TP4A3_HUMAN	14.5	1	0.3436	0.4033

2302	Huntingtin-interacting protein 1-related protein OS=Homo sapiens GN=HIP1R PE=1 SV=2	sp|O75146|HIP1R_HUMAN	2.1	1	0.9036	0.8196
2303	Dolichol-phosphate mannosyltransferase subunit 1 OS=Homo sapiens GN=DPM1 PE=1 SV=1	sp|O60762|DPM1_HUMAN	9.2	1	1.1066	0.8085
2304	Striatin OS=Homo sapiens GN=STRN PE=1 SV=4	sp|O43815|STRN_HUMAN	3.7	1	1.0568	0.8964
2305	Mitogen-activated protein kinase kinase kinase 7 OS=Homo sapiens GN=MAP3K7 PE=1 SV=1	sp|O43318|M3K7_HUMAN	3.3	1	1.0186	0.9858
2306	Cytoplasmic dynein 1 light intermediate chain 2 OS=Homo sapiens GN=DYNC1LI2 PE=1 SV=1	sp|O43237|DC1L2_HUMAN	8.5	2	1.2246	0.4261
2307	CDP-diacylglycerol--inositol 3-phosphatidyltransferase OS=Homo sapiens GN=CDIPT PE=1	sp|O14735|CDIPT_HUMAN	11.7	1	1.0765	0.8388
2308	Protein HEATR9 OS=Homo sapiens GN=HEATR9 PE=2 SV=2	sp|A2RTY3|HEAT9_HUMAN	4.4	1	4.529	0.1592
2309	Cysteine desulfurase, mitochondrial OS=Homo sapiens GN=NFS1 PE=1 SV=3	sp|Q9Y697|NFS1_HUMAN	5.7	1	1.0375	0.9023
2310	Serine-threonine kinase receptor-associated protein OS=Homo sapiens GN=STRAP PE=1 SV=1	sp|Q9Y3F4|STRAP_HUMAN	9.1	2	0.8551	0.7462
2311	Transmembrane emp24 domain-containing protein 5 OS=Homo sapiens GN=TMED5 PE=1	sp|Q9Y3A6|TMED5_HUMAN	9.2	1		
2312	AP-3 complex subunit mu-1 OS=Homo sapiens GN=AP3M1 PE=1 SV=1	sp|Q9Y2T2|AP3M1_HUMAN	4.8	1	1.1803	0.723
2313	Myotubularin-related protein 6 OS=Homo sapiens GN=MTMR6 PE=1 SV=3	sp|Q9Y217|MTMR6_HUMAN	4.2	2	1.0471	0.9157
2314	Cytosolic phospholipase A2 gamma OS=Homo sapiens GN=PLA2G4C PE=1 SV=2	sp|Q9UP65|PA24C_HUMAN	4.3	1	1.1376	0.791
2315	Ras-related protein Rab-22A OS=Homo sapiens GN=RAB22A PE=1 SV=2	sp|Q9UL26|RB22A_HUMAN	8.2	1	0.9462	0.9302
2316	COMM domain-containing protein 9 OS=Homo sapiens GN=COMMD9	sp|Q9P000|COMD9_HUMAN	10.1	1	0.9908	0.9769

2317	Tropomodulin-3 OS=Homo sapiens GN=TMOD3 PE=1 SV=1	sp|Q9NYL9|TMOD3_HUMAN	10.2	1	1.0666	0.8839
2318	39S ribosomal protein L39, mitochondrial OS=Homo sapiens GN=MRPL39 PE=1 SV=3	sp|Q9NYK5|RM39_HUMAN	8.6	1	0.9908	0.995
2319	Pyroglutamyl-peptidase 1 OS=Homo sapiens GN=PGPEP1 PE=1 SV=1	sp|Q9NXJ5|PGPI_HUMAN	15.3	1	1.2589	0.6521
2320	Transmembrane prolyl 4-hydroxylase OS=Homo sapiens GN=P4HTM PE=1 SV=2	sp|Q9NXG6|P4HTM_HUMAN	5.6	1	1.028	0.943
2321	Ubiquinol-cytochrome-c reductase complex assembly factor 1 OS=Homo sapiens GN=UQCC1 PE=1 SV=3	sp|Q9NVA1|UQCC1_HUMAN	6.7	1	1.0093	0.9731
2322	1-acyl-sn-glycerol-3-phosphate acyltransferase gamma OS=Homo sapiens GN=AGPAT3 PE=1 SV=1	sp|Q9NRZ7|PLCC_HUMAN	4	1	0.8395	0.7511
2323	Leucine zipper transcription factor-like protein 1 OS=Homo sapiens GN=LZTFL1 PE=1 SV=1	sp|Q9NQ48|LZTL1_HUMAN	7.7	1	1.0864	0.8283
2324	Protein-glutamate O-methyltransferase OS=Homo sapiens GN=ARMT1 PE=1 SV=1	sp|Q9H993|ARMT1_HUMAN	4.3	1	1.1803	0.7315
2325	Protein RMD5 homolog A OS=Homo sapiens GN=RMND5A PE=1 SV=1	sp|Q9H871|RMD5A_HUMAN	5.1	1	1.1912	0.7247
2326	5'-nucleotidase domain-containing protein 2 OS=Homo sapiens GN=NT5DC2 PE=1 SV=1	sp|Q9H857|NT5D2_HUMAN	3.8	1	0.929	0.8628
2327	Charged multivesicular body protein 4b OS=Homo sapiens GN=CHMP4B PE=1 SV=1	sp|Q9H444|CHM4B_HUMAN	9.4	1	1.2706	0.6358
2328	tRNA dimethylallyltransferase, mitochondrial OS=Homo sapiens GN=TRIT1 PE=1	sp|Q9H3H1|MOD5_HUMAN	5.1	1	1.0965	0.8353
2329	Protein FAM107B OS=Homo sapiens GN=FAM107B PE=1 SV=1	sp|Q9H098|F107B_HUMAN	17.6	1	0.7943	0.6826
2330	Alpha-ketoglutarate-dependent dioxygenase FTO OS=Homo sapiens GN=FTO PE=1	sp|Q9C0B1|FTO_HUMAN	3.6	1	0.6138	0.4242

2331	Transmembrane protein 147 OS=Homo sapiens GN=TMEM147 PE=1 SV=1	sp|Q9BVK8|TM147_HUMAN	7.6	1	0.9638	0.9477
2332	Target of rapamycin complex subunit LST8 OS=Homo sapiens GN=MLST8 PE=1 SV=1	sp|Q9BVC4|LST8_HUMAN	8.3	1	1.2706	0.6906
2333	Protein YIPF4 OS=Homo sapiens GN=YIPF4 PE=1 SV=1	sp|Q9BSR8|YIPF4_HUMAN	7.4	1	1.0093	0.8242
2334	H/ACA ribonucleoprotein complex non-core subunit NAF1 OS=Homo sapiens GN=NAF1 PE=1 SV=2	sp|Q96HR8|NAF1_HUMAN	3.8	1	0.9204	0.8764
2335	Probable D-tyrosyl-tRNA(Tyr) deacylase 2 OS=Homo sapiens GN=DTD2 PE=2 SV=1	sp|Q96FN9|DTD2_HUMAN	6	1	0.8872	0.752
2336	Acyl-CoA:lysophosphatidylglycerol acyltransferase 1 OS=Homo sapiens GN=LPGAT1 PE=1 SV=1	sp|Q92604|LGAT1_HUMAN	5.9	1	0.912	0.8681
2337	B-cell CLL/lymphoma 7 protein family member C OS=Homo sapiens GN=BCL7C PE=1 SV=3	sp|Q8WUZ0|BCL7C_HUMAN	9.2	1	0.9908	0.9806
2338	Ubiquitin-associated and SH3 domain-containing protein B OS=Homo sapiens GN=UBASH3B	sp|Q8TF42|UBS3B_HUMAN	3.9	1	1.0186	0.9759
2339	GrpE protein homolog 2, mitochondrial OS=Homo sapiens GN=GRPEL2 PE=1 SV=1	sp|Q8TAA5|GRPE2_HUMAN	11.1	2	0.9908	0.9997
2340	Protein LSM14 homolog A OS=Homo sapiens GN=LSM14A PE=1 SV=3	sp|Q8ND56|LS14A_HUMAN	6.9	1	1	0.9851
2341	Protein KRI1 homolog OS=Homo sapiens GN=KRI1 PE=1	sp|Q8N9T8|KRI1_HUMAN	3.4	1	1.0471	0.9169
2342	WD and tetratricopeptide repeats protein 1 OS=Homo sapiens GN=WDTC1 PE=1 SV=2	sp|Q8N5D0|WDTC1_HUMAN	3.7	1	1.0093	0.9702
2343	Tetratricopeptide repeat protein 5 OS=Homo sapiens GN=TTC5 PE=1 SV=2	sp|Q8N0Z6|TTC5_HUMAN	6.8	1	0.8241	0.7103
2344	Palmitoyltransferase ZDHHC13 OS=Homo sapiens GN=ZDHHC13 PE=1 SV=3	sp|Q8IUH4|ZDH13_HUMAN	2.7	1	0.9376	0.9228

2345	Trafficking protein particle complex subunit 6B OS=Homo sapiens GN=TRAPPC6B PE=1 SV=1	sp|Q86SZ2|TPC6B_HUMAN	8.9	1	0.9817	0.9728
2346	Ubiquitin-conjugating enzyme E2 Q1 OS=Homo sapiens GN=UBE2Q1 PE=1 SV=1	sp|Q7Z7E8|UB2Q1_HUMAN	3.8	1	1.0568	0.8945
2347	17-beta-hydroxysteroid dehydrogenase 13 OS=Homo sapiens GN=HSD17B13 PE=1 SV=1	sp|Q7Z5P4|DHB13_HUMAN	7.7	1	1.2023	0.7102
2348	Heparan sulfate 2-O-sulfotransferase 1 OS=Homo sapiens GN=HS2ST1 PE=1	sp|Q7LGA3|HS2ST_HUMAN	7.6	1	0.955	0.9142
2349	Rho GTPase-activating protein 27 OS=Homo sapiens GN=ARHGAP27	sp|Q6ZUM4|RHG27_HUMAN	2.1	1	0.9204	0.8563
2350	Rho GTPase-activating protein 36 OS=Homo sapiens GN=ARHGAP36	sp|Q6ZRI8|RHG36_HUMAN	3.7	1	1.9231	0.3386
2351	NF-X1-type zinc finger protein NFXL1 OS=Homo sapiens GN=NFXL1 PE=1	sp|Q6ZNB6|NFXL1_HUMAN	2.2	1		
2352	Molybdate-anion transporter OS=Homo sapiens GN=MFSD5 PE=1 SV=2	sp|Q6N075|MFSD5_HUMAN	4.7	1	1.1066	0.8267
2353	THO complex subunit 7 homolog OS=Homo sapiens GN=THOC7 PE=1 SV=3	sp|Q6I9Y2|THOC7_HUMAN	10.3	1	0.8318	0.7346
2354	Ribonuclease H2 subunit B OS=Homo sapiens GN=RNASEH2B PE=1 SV=1	sp|Q5TBB1|RNH2B_HUMAN	4.8	1		
2355	NEDD8 OS=Homo sapiens GN=NEDD8 PE=1 SV=1	sp|Q15843|NEDD8_HUMAN	21	1	0.8091	0.6738
2356	Myeloid leukemia factor 2 OS=Homo sapiens GN=MLF2 PE=1 SV=1	sp|Q15773|MLF2_HUMAN	13.3	1	0.9462	0.935
2357	DNA polymerase delta subunit 3 OS=Homo sapiens GN=POLD3 PE=1 SV=2	sp|Q15054|DPOD3_HUMAN	4.5	1	0.8091	0.6856
2358	Kelch-like ECH-associated protein 1 OS=Homo sapiens GN=KEAP1 PE=1 SV=2	sp|Q14145|KEAP1_HUMAN	1.9	1	0.6607	0.4892
2359	Serine incorporator 3 OS=Homo sapiens GN=SERINC3	sp|Q13530|SERC3_HUMAN	7.4	1	0.8872	0.8181
2360	Nuclear inhibitor of protein phosphatase 1 OS=Homo sapiens GN=PPP1R8 PE=1	sp|Q12972|PP1R8_HUMAN	8.5	1	0.9462	0.8728
2361	Secernin-3 OS=Homo sapiens GN=SCRN3 PE=1 SV=1	sp|Q0VDG4|SCRN3_HUMAN	5	1		

2362	Transcription factor p65 OS=Homo sapiens GN=RELA PE=1 SV=2	sp|Q04206|TF65_HUMAN	6	3	1.0568	0.974
2363	Forkhead box protein K2 OS=Homo sapiens GN=FOXK2 PE=1	sp|Q01167|FOXK2_HUMAN	4.5	1	0.9908	0.9998
2364	Dermcidin OS=Homo sapiens GN=DCD PE=1 SV=2	sp|P81605|DCD_HUMAN	15.5	1	1	0.9872
2365	Interferon-induced 35 kDa protein OS=Homo sapiens GN=IFI35 PE=1 SV=5	sp|P80217|IN35_HUMAN	9.1	1	1.3183	0.6037
2366	Signal peptidase complex catalytic subunit SEC11A OS=Homo sapiens GN=SEC11A PE=1 SV=1	sp|P67812|SC11A_HUMAN	9.5	1	1.1272	0.7977
2367	WD repeat-containing protein 5 OS=Homo sapiens GN=WDR5 PE=1	sp|P61964|WDR5_HUMAN	7.2	1	0.9817	0.9882
2368	Phosphatidylinositol 3,4,5-trisphosphate 3-phosphatase and dual-specificity protein phosphatase PTEN OS=Homo sapiens GN=PTEN	sp|P60484|PTEN_HUMAN	6.7	1	0.9036	0.8514
2369	cAMP-regulated phosphoprotein 19 OS=Homo sapiens GN=ARPP19 PE=1 SV=2	sp|P56211|ARP19_HUMAN	26.8	1	0.8241	0.7154
2370	Ribosomal RNA processing protein 1 homolog A OS=Homo sapiens GN=RRP1 PE=1 SV=1	sp|P56182|RRP1_HUMAN	3	1	0.8872	0.8216
2371	Mitogen-activated protein kinase 10 OS=Homo sapiens GN=MAPK10 PE=1 SV=2	sp|P53779|MK10_HUMAN	5.4	1	0.8954	0.8377
2372	Beta-arrestin-1 OS=Homo sapiens GN=ARRB1 PE=1 SV=2	sp|P49407|ARRB1_HUMAN	6	1	0.9908	0.9978
2373	Wiskott-Aldrich syndrome protein OS=Homo sapiens GN=WAS PE=1 SV=4	sp|P42768|WASP_HUMAN	4	2	1.2474	0.6717
2374	2-oxoisovalerate dehydrogenase subunit beta, mitochondrial OS=Homo sapiens GN=BCKDHB PE=1 SV=2	sp|P21953|ODBB_HUMAN	13.3	1	1	0.99
2375	Proteasome subunit beta type-1 OS=Homo sapiens GN=PSMB1 PE=1 SV=2	sp|P20618|PSB1_HUMAN	21.2	3	1.0375	0.9268
2376	DNA repair protein XRCC1 OS=Homo sapiens GN=XRCC1 PE=1	sp|P18887|XRCC1_HUMAN	3.3	1	0.9462	0.9285
2377	Prolyl 4-hydroxylase subunit alpha-1 OS=Homo sapiens GN=P4HA1 PE=1	sp|P13674|P4HA1_HUMAN	6.9	1	1.0375	0.9253

2378	Lysosome-associated membrane glycoprotein 1 OS=Homo sapiens GN=LAMP1 PE=1 SV=3	sp|P11279|LAMP1_HUMAN	5.5	2	1.556	0.4558
2379	Arachidonate 5-lipoxygenase OS=Homo sapiens GN=ALOX5 PE=1 SV=2	sp|P09917|LOX5_HUMAN	3.4	1	0.955	0.908
2380	Histone H1.0 OS=Homo sapiens GN=H1F0 PE=1 SV=3	sp|P07305|H10_HUMAN	13.9	3	1.2706	0.6393
2381	Thymidine kinase, cytosolic OS=Homo sapiens GN=TK1 PE=1	sp|P04183|KITH_HUMAN	9.4	1	0.7244	0.5768
2382	BAG family molecular chaperone regulator 2 OS=Homo sapiens GN=BAG2 PE=1 SV=1	sp|O95816|BAG2_HUMAN	14.2	1	1.0965	0.7631
2383	Persulfide dioxygenase ETHE1, mitochondrial OS=Homo sapiens GN=ETHE1 PE=1 SV=2	sp|O95571|ETHE1_HUMAN	7.9	1	1.0186	0.9377
2384	Ribosome biogenesis protein NSA2 homolog OS=Homo sapiens GN=NSA2 PE=1	sp|O95478|NSA2_HUMAN	9.2	1	0.8954	0.8212
2385	Zinc finger and BTB domain-containing protein 7A OS=Homo sapiens GN=ZBTB7A	sp|O95365|ZBT7A_HUMAN	4.1	1	1.0765	0.8195
2386	F-box only protein 21 OS=Homo sapiens GN=FBXO21 PE=2 SV=2	sp|O94952|FBX21_HUMAN	3.5	1	0.9462	0.9321
2387	D-glucuronyl C5-epimerase OS=Homo sapiens GN=GLCE PE=1 SV=3	sp|O94923|GLCE_HUMAN	3.2	1	1.4454	0.5179
2388	Tubulin-specific chaperone A OS=Homo sapiens GN=TBCA PE=1 SV=3	sp|O75347|TBCA_HUMAN	17.6	1	0.0112	0.0251
2389	Programmed cell death protein 6 OS=Homo sapiens GN=PDCD6 PE=1 SV=1	sp|O75340|PDCD6_HUMAN	8.4	1	1.0093	0.9492
2390	Mitochondrial import inner membrane translocase subunit TIM44 OS=Homo sapiens GN=TIMM44 PE=1 SV=2	sp|O43615|TIM44_HUMAN	4.9	1	1.0965	0.8376
2391	ER membrane protein complex subunit 8 OS=Homo sapiens GN=EMC8 PE=1	sp|O43402|EMC8_HUMAN	11.4	1	1.1272	0.7964
2392	Centrin-3 OS=Homo sapiens GN=CETN3 PE=1	sp|O15182|CETN3_HUMAN	12	1	0.4571	0.4175

2393	Secretory carrier-associated membrane protein 2 OS=Homo sapiens GN=SCAMP2	sp|O15127|SCAM2_HUMAN	8.8	1	1.2706	0.6403
2394	Syntaxin-16 OS=Homo sapiens GN=STX16 PE=1 SV=3	sp|O14662|STX16_HUMAN	5.2	1	1.0666	0.8944
2395	Interferon-related developmental regulator 1 OS=Homo sapiens GN=IFRD1 PE=1 SV=4	sp|O00458|IFRD1_HUMAN	5.5	1	1.3305	0.5932
2396	RNA 3'-terminal phosphate cyclase OS=Homo sapiens GN=RTCA PE=1 SV=1	sp|O00442|RTCA_HUMAN	5.5	1	1.0375	0.9303
2397	Putative prolyl-tRNA synthetase associated domain-containing protein 1 OS=Homo sapiens GN=PRORSD1P	sp|A6NEY8|PRXD1_HUMAN	13	1	1.0375	0.9099
2398	REVERSED Metabotropic glutamate receptor 4 OS=Homo sapiens GN=GRM4 PE=2 SV=1	RRRRRsp|Q14833|GRM4_HUMAN	2.3	1	1.1376	0.7816
2399	Mitochondrial import inner membrane translocase subunit Tim13 OS=Homo sapiens GN=TIMM13 PE=1 SV=1	sp|Q9Y5L4|TIM13_HUMAN	19	1	1	0.9838
2400	DNA-directed RNA polymerase III subunit RPC8 OS=Homo sapiens GN=POLR3H PE=1 SV=1	sp|Q9Y535|RPC8_HUMAN	7.8	1	0.7798	0.6643
2401	U6 snRNA-associated Sm-like protein LSm4 OS=Homo sapiens GN=LSM4 PE=1 SV=1	sp|Q9Y4Z0|LSM4_HUMAN	13	2	0.879	0.8042
2402	Splicing factor 3B subunit 6 OS=Homo sapiens GN=SF3B6 PE=1	sp|Q9Y3B4|SF3B6_HUMAN	13.6	1	1	0.9832
2403	Serine/threonine-protein kinase 38-like OS=Homo sapiens GN=STK38L PE=1	sp|Q9Y2H1|ST38L_HUMAN	3.2	1	1.1169	0.7884
2404	HIG1 domain family member 1A, mitochondrial OS=Homo sapiens GN=HIGD1A PE=1 SV=1	sp|Q9Y241|HIG1A_HUMAN	9.7	1	0.9462	0.9338
2405	mRNA turnover protein 4 homolog OS=Homo sapiens GN=MRTO4 PE=1 SV=2	sp|Q9UKD2|MRT4_HUMAN	5	1	0.8872	0.8185
2406	Mortality factor 4-like protein 1 OS=Homo sapiens GN=MORF4L1 PE=1 SV=2	sp|Q9UBU8|MO4L1_HUMAN	5.5	1	0.5861	0.3972
2407	ER membrane protein complex subunit 3 OS=Homo sapiens GN=EMC3 PE=1	sp|Q9P0I2|EMC3_HUMAN	8	1	0.9727	0.9974

2408	CCR4-NOT transcription complex subunit 2 OS=Homo sapiens GN=CNOT2 PE=1 SV=1	sp|Q9NZN8|CNOT2_HUMAN	3.3	1	1.0471	0.9066
2409	Transmembrane protein 160 OS=Homo sapiens GN=TMEM160 PE=1 SV=1	sp|Q9NX00|TM160_HUMAN	9.6	1	1.1912	0.7175
2410	ATP synthase subunit s-like protein OS=Homo sapiens GN=ATP5SL PE=2	sp|Q9NW81|AT5SL_HUMAN	6.2	1	0.9462	0.931
2411	Protein FAM114A2 OS=Homo sapiens GN=FAM114A2	sp|Q9NRY5|F1142_HUMAN	5.9	1	1.0568	0.8957
2412	RNA-binding protein PNO1 OS=Homo sapiens GN=PNO1 PE=1	sp|Q9NRX1|PNO1_HUMAN	7.1	1	0.8872	0.7843
2413	Oligosaccharyltransferase complex subunit OSTC OS=Homo sapiens GN=OSTC PE=1 SV=1	sp|Q9NRP0|OSTC_HUMAN	12.1	1	1.2023	0.707
2414	PDZ and LIM domain protein 7 OS=Homo sapiens GN=PDLIM7 PE=1 SV=1	sp|Q9NR12|PDLI7_HUMAN	3.3	1	1	0.9933
2415	39S ribosomal protein L44, mitochondrial OS=Homo sapiens GN=MRPL44 PE=1 SV=1	sp|Q9H9J2|RM44_HUMAN	6.9	1	1.0765	0.872
2416	RING finger protein 121 OS=Homo sapiens GN=RNF121 PE=1 SV=1	sp|Q9H920|RN121_HUMAN	6.4	1	1.1803	0.7319
2417	MOB kinase activator 1A OS=Homo sapiens GN=MOB1A PE=1 SV=4	sp|Q9H8S9|MOB1A_HUMAN	5.6	1	1.0093	0.9647
2418	RWD domain-containing protein 1 OS=Homo sapiens GN=RWDD1 PE=1	sp|Q9H446|RWDD1_HUMAN	7.4	1	0.9376	0.9151
2419	BolA-like protein 2 OS=Homo sapiens GN=BOLA2 PE=1 SV=1	sp|Q9H3K6|BOLA2_HUMAN	16.3	1	0.9462	0.9183
2420	Magnesium transporter protein 1 OS=Homo sapiens GN=MAGT1 PE=1	sp|Q9H0U3|MAGT1_HUMAN	5.4	1	1.0471	0.9114
2421	Signal peptidase complex catalytic subunit SEC11C OS=Homo sapiens GN=SEC11C PE=1 SV=3	sp|Q9BY50|SC11C_HUMAN	7.8	1	1.2589	0.6615
2422	Vesicle-associated membrane protein 8 OS=Homo sapiens GN=VAMP8 PE=1	sp|Q9BV40|VAMP8_HUMAN	14	1	0.9817	0.9801
2423	Monoacylglycerol lipase ABHD6 OS=Homo sapiens GN=ABHD6 PE=1 SV=1	sp|Q9BV23|ABHD6_HUMAN	5.6	1	0.9204	0.8691

2424	Migration and invasion enhancer 1 OS=Homo sapiens GN=MIEN1 PE=1 SV=1	sp|Q9BRT3|MIEN1_HUMAN	17.4	2		
2425	Programmed cell death 1 ligand 2 OS=Homo sapiens GN=PDCD1LG2 PE=1 SV=2	sp|Q9BQ51|PD1L2_HUMAN	5.9	1	0.8472	0.7603
2426	39S ribosomal protein L34, mitochondrial OS=Homo sapiens GN=MRPL34 PE=1 SV=1	sp|Q9BQ48|RM34_HUMAN	17.4	1	0.929	0.8858
2427	E3 ubiquitin-protein ligase RING2 OS=Homo sapiens GN=RNF2 PE=1 SV=1	sp|Q99496|RING2_HUMAN	3.3	1	0.863	0.7898
2428	Cleft lip and palate transmembrane protein 1-like protein OS=Homo sapiens GN=CLPTM1L	sp|Q96KA5|CLP1L_HUMAN	3.7	1	1.0864	0.8517
2429	Vesicle-trafficking protein SEC22a OS=Homo sapiens GN=SEC22A PE=1 SV=1	sp|Q96IW7|SC22A_HUMAN	7.5	1	1.0965	0.843
2430	UNC119-binding protein C5orf30 OS=Homo sapiens GN=C5orf30 PE=1 SV=1	sp|Q96GV9|CE030_HUMAN	6.8	1	0.8241	0.7225
2431	Charged multivesicular body protein 6 OS=Homo sapiens GN=CHMP6 PE=1 SV=3	sp|Q96FZ7|CHMP6_HUMAN	9	1	1.0965	0.8324
2432	Dehydrodolichyl diphosphate syntase complex subunit NUS1 OS=Homo sapiens GN=NUS1 PE=1 SV=1	sp|Q96E22|NGBR_HUMAN	8.9	1	0.871	0.7927
2433	5-methylcytosine rRNA methyltransferase NSUN4 OS=Homo sapiens GN=NSUN4 PE=1	sp|Q96CB9|NSUN4_HUMAN	2.9	1	1.0965	0.8446
2434	Vesicle transport through interaction with t-SNAREs homolog 1A OS=Homo sapiens GN=VTI1A PE=1 SV=2	sp|Q96AJ9|VTI1A_HUMAN	6.9	1	1.2023	0.7081
2435	Transmembrane protein 230 OS=Homo sapiens GN=TMEM230 PE=1 SV=1	sp|Q96A57|TM230_HUMAN	13.3	1	1.2246	0.7074
2436	39S ribosomal protein L24, mitochondrial OS=Homo sapiens GN=MRPL24 PE=1 SV=1	sp|Q96A35|RM24_HUMAN	10.2	1	1.2023	0.7067
2437	RNA-binding protein with multiple splicing OS=Homo sapiens GN=RBPMS PE=1	sp|Q93062|RBPMS_HUMAN	12.8	1	0.7656	0.7975
2438	Ubiquitin-like domain-containing CTD phosphatase 1 OS=Homo sapiens GN=UBLCP1 PE=1 SV=2	sp|Q8WVY7|UBCP1_HUMAN	8.5	1	1.2023	0.7083

2439	Alanine aminotransferase 2 OS=Homo sapiens GN=GPT2 PE=1 SV=1	sp|Q8TD30|ALAT2_HUMAN	4	1	1.0864	0.8152
2440	Nucleoporin Nup43 OS=Homo sapiens GN=NUP43 PE=1 SV=1	sp|Q8NFH3|NUP43_HUMAN	3.9	1	1.0186	0.9538
2441	DTW domain-containing protein 1 OS=Homo sapiens GN=DTWD1 PE=1	sp|Q8N5C7|DTWD1_HUMAN	5.3	1	1.0375	0.9155
2442	Carnosine N-methyltransferase OS=Homo sapiens GN=C9orf41 PE=1 SV=1	sp|Q8N4J0|CARME_HUMAN	4.9	1	1.2134	0.6901
2443	Protein canopy homolog 4 OS=Homo sapiens GN=CNPY4 PE=2 SV=1	sp|Q8N129|CNPY4_HUMAN	6.5	1	1.3932	0.541
2444	Kelch repeat and BTB domain-containing protein 2 OS=Homo sapiens GN=KBTBD2	sp|Q8IY47|KBTB2_HUMAN	2.9	1	0.879	0.8129
2445	Cytokine receptor-like factor 3 OS=Homo sapiens GN=CRLF3 PE=1 SV=2	sp|Q8IUI8|CRLF3_HUMAN	3.4	1	1.1482	0.749
2446	RNA-binding protein 45 OS=Homo sapiens GN=RBM45 PE=1	sp|Q8IUH3|RBM45_HUMAN	2.9	1	0.8954	0.7991
2447	Nuclear receptor 2C2-associated protein OS=Homo sapiens GN=NR2C2AP PE=1 SV=1	sp|Q86WQ0|NR2CA_HUMAN	5	1	0.8872	0.8087
2448	BRCA1-associated protein OS=Homo sapiens GN=BRAP PE=1 SV=2	sp|Q7Z569|BRAP_HUMAN	2.5	1	1.2589	0.6456
2449	39S ribosomal protein L2, mitochondrial OS=Homo sapiens GN=MRPL2 PE=1 SV=2	sp|Q5T653|RM02_HUMAN	4.6	1	0.9908	0.9928
2450	Putative methyltransferase C9orf114 OS=Homo sapiens GN=C9orf114 PE=1 SV=3	sp|Q5T280|CI114_HUMAN	4.3	1	0.9204	0.8697
2451	Vacuolar protein sorting-associated protein 26B OS=Homo sapiens GN=VPS26B PE=1 SV=2	sp|Q4G0F5|VP26B_HUMAN	5.4	1	0.879	0.8188
2452	Putative uncharacterized protein ZNRD1-AS1 OS=Homo sapiens GN=ZNRD1-AS1	sp|Q2KJ03|ZRAS1_HUMAN	8.6	1		
2453	V-type proton ATPase subunit F OS=Homo sapiens GN=ATP6V1F PE=1 SV=2	sp|Q16864|VATF_HUMAN	17.7	1	1.0375	0.8906
2454	Translocating chain-associated membrane protein 1 OS=Homo sapiens GN=TRAM1 PE=1	sp|Q15629|TRAM1_HUMAN	5.9	1	1.1482	0.748

2455	Ras-related protein Rab-35 OS=Homo sapiens GN=RAB35 PE=1	sp|Q15286|RAB35_HUMAN	11	1	0.8954	0.8353
2456	Serum paraoxonase/lactonase 3 OS=Homo sapiens GN=PON3 PE=1 SV=3	sp|Q15166|PON3_HUMAN	9.6	1	1.0186	0.9309
2457	Protein FRG1 OS=Homo sapiens GN=FRG1 PE=1 SV=1	sp|Q14331|FRG1_HUMAN	7.4	1	0.8318	0.7295
2458	Mediator of RNA polymerase II transcription subunit 21 OS=Homo sapiens GN=MED21 PE=1	sp|Q13503|MED21_HUMAN	13.9	1	1.1482	0.7693
2459	Transmembrane emp24 domain-containing protein 1 OS=Homo sapiens GN=TMED1 PE=1	sp|Q13445|TMED1_HUMAN	5.7	1	1.0471	0.911
2460	Interferon-induced transmembrane protein 2 OS=Homo sapiens GN=IFITM2 PE=1	sp|Q01629|IFM2_HUMAN	15.9	1	4.0926	0.2335
2461	Thioredoxin-like protein 4A OS=Homo sapiens GN=TXNL4A PE=1 SV=1	sp|P83876|TXN4A_HUMAN	10.6	1	0.9376	0.9061
2462	60S ribosomal protein L38 OS=Homo sapiens GN=RPL38 PE=1 SV=2	sp|P63173|RL38_HUMAN	27.1	1	0.9817	0.9911
2463	Vesicle-associated membrane protein 2 OS=Homo sapiens GN=VAMP2 PE=1	sp|P63027|VAMP2_HUMAN	18.1	1	1.2023	0.702
2464	TATA box-binding protein-like protein 1 OS=Homo sapiens GN=TBPL1 PE=1	sp|P62380|TBPL1_HUMAN	11.3	1	1.1169	0.8314
2465	Small nuclear ribonucleoprotein Sm D1 OS=Homo sapiens GN=SNRPD1 PE=1 SV=1	sp|P62314|SMD1_HUMAN	20.2	1	0.8241	0.7941
2466	40S ribosomal protein S29 OS=Homo sapiens GN=RPS29 PE=1 SV=2	sp|P62273|RS29_HUMAN	26.8	4	0.4875	0.7322
2467	Ubiquitin-fold modifier 1 OS=Homo sapiens GN=UFM1 PE=1 SV=1	sp|P61960|UFM1_HUMAN	23.5	1	0.9817	0.9968
2468	Ubiquitin-conjugating enzyme E2 N OS=Homo sapiens GN=UBE2N PE=1	sp|P61088|UBE2N_HUMAN	13.2	1	0.955	0.9271
2469	Branched-chain-amino-acid aminotransferase, cytosolic OS=Homo sapiens GN=BCAT1 PE=1	sp|P54687|BCAT1_HUMAN	5.2	1	1.0093	0.9626
2470	Methylosome subunit pICln OS=Homo sapiens GN=CLNS1A PE=1 SV=1	sp|P54105|ICLN_HUMAN	9.7	1	1.0864	0.8602

2471	Activated RNA polymerase II transcriptional coactivator p15 OS=Homo sapiens GN=SUB1 PE=1 SV=3	sp|P53999|TCP4_HUMAN	15.8	1		
2472	Methionine aminopeptidase 1 OS=Homo sapiens GN=METAP1 PE=1 SV=2	sp|P53582|MAP11_HUMAN	4.9	1	1.0186	0.9568
2473	Ribose-5-phosphate isomerase OS=Homo sapiens GN=RPIA PE=1 SV=3	sp|P49247|RPIA_HUMAN	11.3	1	1.1272	0.6901
2474	Glutamate--cysteine ligase catalytic subunit OS=Homo sapiens GN=GCLC PE=1	sp|P48506|GSH1_HUMAN	2	1	1.4859	0.4896
2475	Glucosamine-6-phosphate isomerase 1 OS=Homo sapiens GN=GNPDA1	sp|P46926|GNPI1_HUMAN	4.2	1	1.3804	0.5513
2476	Tristetraprolin OS=Homo sapiens GN=ZFP36 PE=1 SV=1	sp|P26651|TTP_HUMAN	6.4	1	2.0701	0.4484
2477	Transcriptional repressor protein YY1 OS=Homo sapiens GN=YY1 PE=1 SV=2	sp|P25490|TYY1_HUMAN	2.9	1	0.5598	0.5077
2478	Protein-L-isoaspartate(D-aspartate) O-methyltransferase OS=Homo sapiens GN=PCMT1 PE=1 SV=4	sp|P22061|PIMT_HUMAN	9.7	1	0.912	0.8636
2479	60S ribosomal protein L35a OS=Homo sapiens GN=RPL35A PE=1 SV=2	sp|P18077|RL35A_HUMAN	10.9	1	0.871	0.8707
2480	Transcription factor jun-B OS=Homo sapiens GN=JUNB PE=1 SV=1	sp|P17275|JUNB_HUMAN	4	1	1.2246	0.6542
2481	Ras-related protein Ral-B OS=Homo sapiens GN=RALB PE=1 SV=1	sp|P11234|RALB_HUMAN	6.8	1	1.0471	0.9116
2482	Dihydropteridine reductase OS=Homo sapiens GN=QDPR PE=1 SV=2	sp|P09417|DHPR_HUMAN	14.8	1	0.9727	0.9728
2483	U2 small nuclear ribonucleoprotein B'' OS=Homo sapiens GN=SNRPB2	sp|P08579|RU2B_HUMAN	13.8	1	0.8472	0.7594
2484	Apolipoprotein A-I OS=Homo sapiens GN=APOA1 PE=1 SV=1	sp|P02647|APOA1_HUMAN	7.9	1	1.1066	0.8249
2485	Probable ATP-dependent RNA helicase DDX58 OS=Homo sapiens GN=DDX58 PE=1 SV=2	sp|O95786|DDX58_HUMAN	1.5	1	1.3932	0.5391
2486	Potassium channel subfamily K member 5 OS=Homo sapiens GN=KCNK5 PE=1 SV=1	sp|O95279|KCNK5_HUMAN	3.4	1	0.9462	0.9303

2487	Importin-13 OS=Homo sapiens GN=IPO13 PE=1 SV=3	sp|O94829|IPO13_HUMAN	2.6	1	1	0.9639
2488	Cyclin-dependent kinase 2-associated protein 2 OS=Homo sapiens GN=CDK2AP2 PE=1 SV=1	sp|O75956|CDKA2_HUMAN	9.5	1	0.8551	0.7397
2489	Dynactin subunit 3 OS=Homo sapiens GN=DCTN3 PE=1 SV=1	sp|O75935|DCTN3_HUMAN	7	1	1.1588	0.7534
2490	PRA1 family protein 3 OS=Homo sapiens GN=ARL6IP5 PE=1 SV=1	sp|O75915|PRAF3_HUMAN	8	1	0.9727	0.975
2491	Barrier-to-autointegration factor OS=Homo sapiens GN=BANF1 PE=1	sp|O75531|BAF_HUMAN	18	2	0.871	0.8835
2492	Docking protein 2 OS=Homo sapiens GN=DOK2 PE=1 SV=2	sp|O60496|DOK2_HUMAN	6.8	1	1.028	0.9396
2493	Sorting nexin-3 OS=Homo sapiens GN=SNX3 PE=1 SV=3	sp|O60493|SNX3_HUMAN	11.1	1	0.6918	0.8525
2494	Calumenin OS=Homo sapiens GN=CALU PE=1 SV=2	sp|O43852|CALU_HUMAN	8.9	1	1	0.9924
2495	Zinc finger and BTB domain-containing protein 14 OS=Homo sapiens GN=ZBTB14 PE=1	sp|O43829|ZBT14_HUMAN	2.9	1	0.9727	0.9755
2496	Protein phosphatase 1G OS=Homo sapiens GN=PPM1G PE=1 SV=1	sp|O15355|PPM1G_HUMAN	3.1	1	1.0471	0.9096
2497	Transmembrane 9 superfamily member 1 OS=Homo sapiens GN=TM9SF1 PE=2 SV=2	sp|O15321|TM9S1_HUMAN	2.6	1	1.0864	0.8491
2498	Acyl carrier protein, mitochondrial OS=Homo sapiens GN=NDUFAB1	sp|O14561|ACPM_HUMAN	11.5	1	1	0.9921
2499	NADH dehydrogenase [ubiquinone] iron-sulfur protein 8, mitochondrial OS=Homo sapiens GN=NDUFS8 PE=1 SV=1	sp|O00217|NDUS8_HUMAN	10.5	1	1.1169	0.8201
2500	Protein unc-119 homolog B OS=Homo sapiens GN=UNC119B PE=1 SV=1	sp|A6NIH7|U119B_HUMAN	5.2	1	1.0186	0.9558
2501	REVERSED CapZ-interacting protein OS=Homo sapiens GN=RCSD1 PE=1 SV=1	RRRRRsp|Q6JBY9|CPZIP_HUMAN	4.1	1	0.929	0.8901
2502	Zinc finger protein 706 OS=Homo sapiens GN=ZNF706 PE=1 SV=1	sp|Q9Y5V0|ZN706_HUMAN	14.5	1	0.3631	0.3139

2503	Trafficking protein particle complex subunit 4 OS=Homo sapiens GN=TRAPPC4 PE=1 SV=1	sp|Q9Y296|TPPC4_HUMAN	4.6	1	1.0568	0.894
2504	Translocon-associated protein subunit gamma OS=Homo sapiens GN=SSR3 PE=1 SV=1	sp|Q9UNL2|SSRG_HUMAN	7.6	5	1.406	0.6477
2505	Post-GPI attachment to proteins factor 2 OS=Homo sapiens GN=PGAP2 PE=1 SV=2	sp|Q9UHJ9|PGAP2_HUMAN	3.9	1	0.9376	0.9031
2506	Protein lin-7 homolog C OS=Homo sapiens GN=LIN7C PE=1 SV=1	sp|Q9NUP9|LIN7C_HUMAN	4.1	1	1	0.9871
2507	Ethylmalonyl-CoA decarboxylase OS=Homo sapiens GN=ECHDC1 PE=1 SV=2	sp|Q9NTX5|ECHD1_HUMAN	3.6	1	1.0765	0.8647
2508	14 kDa phosphohistidine phosphatase OS=Homo sapiens GN=PHPT1 PE=1 SV=1	sp|Q9NRX4|PHP14_HUMAN	10.4	1		
2509	DNA polymerase epsilon subunit 3 OS=Homo sapiens GN=POLE3 PE=1 SV=1	sp|Q9NRF9|DPOE3_HUMAN	12.2	2	0.879	0.811
2510	NAD-dependent protein deacetylase sirtuin-7 OS=Homo sapiens GN=SIRT7 PE=1 SV=1	sp|Q9NRC8|SIR7_HUMAN	3.3	1	0.9817	0.9814
2511	Exosome complex component RRP41 OS=Homo sapiens GN=EXOSC4 PE=1 SV=3	sp|Q9NPD3|EXOS4_HUMAN	5.7	1	1	0.9566
2512	Rho-related GTP-binding protein RhoF OS=Homo sapiens GN=RHOF PE=1 SV=1	sp|Q9HBH0|RHOF_HUMAN	4.7	1	1.0864	0.8555
2513	Calcyclin-binding protein OS=Homo sapiens GN=CACYBP PE=1 SV=2	sp|Q9HB71|CYBP_HUMAN	4.4	1	0.6918	0.5284
2514	Protein FAM188A OS=Homo sapiens GN=FAM188A PE=1 SV=1	sp|Q9H8M7|F188A_HUMAN	3.4	1	1.2823	0.6322
2515	Peptidyl-prolyl cis-trans isomerase-like 3 OS=Homo sapiens GN=PPIL3 PE=1 SV=1	sp|Q9H2H8|PPIL3_HUMAN	8.7	1	0.9727	0.9775
2516	Mediator of RNA polymerase II transcription subunit 28 OS=Homo sapiens GN=MED28 PE=1	sp|Q9H204|MED28_HUMAN	3.9	1	0.8318	0.7308
2517	COMM domain-containing protein 4 OS=Homo sapiens GN=COMMD4	sp|Q9H0A8|COMD4_HUMAN	10.1	1	1.0965	0.794
2518	Uridine-cytidine kinase 2 OS=Homo sapiens GN=UCK2 PE=1 SV=1	sp|Q9BZX2|UCK2_HUMAN	4.6	1	0.6982	0.5368

2519	39S ribosomal protein L20, mitochondrial OS=Homo sapiens GN=MRPL20 PE=1 SV=1	sp|Q9BYC9|RM20_HUMAN	4	1	0.9817	0.9788
2520	Splicing factor 3B subunit 5 OS=Homo sapiens GN=SF3B5 PE=1	sp|Q9BWJ5|SF3B5_HUMAN	10.5	1	0.9908	0.9892
2521	45 kDa calcium-binding protein OS=Homo sapiens GN=SDF4 PE=1 SV=1	sp|Q9BRK5|CAB45_HUMAN	1.9	1	1.4588	0.5027
2522	Copine-1 OS=Homo sapiens GN=CPNE1 PE=1	sp|Q99829|CPNE1_HUMAN	3	1	1.4588	0.6213
2523	Thioredoxin, mitochondrial OS=Homo sapiens GN=TXN2 PE=1 SV=2	sp|Q99757|THIOM_HUMAN	6	1	1.1376	0.7751
2524	WD repeat and FYVE domain-containing protein 2 OS=Homo sapiens GN=WDFY2 PE=2	sp|Q96P53|WDFY2_HUMAN	3.5	1	0.9204	0.8026
2525	DCN1-like protein 1 OS=Homo sapiens GN=DCUN1D1	sp|Q96GG9|DCNL1_HUMAN	6.6	1	1.0375	0.9916
2526	Adiponectin receptor protein 1 OS=Homo sapiens GN=ADIPOR1 PE=1 SV=1	sp|Q96A54|ADR1_HUMAN	4.5	1	1.1695	0.7849
2527	Myeloid-derived growth factor OS=Homo sapiens GN=MYDGF PE=1 SV=1	sp|Q969H8|MYDGF_HUMAN	5.2	1	0.9638	0.9785
2528	Ninjurin-1 OS=Homo sapiens GN=NINJ1 PE=1 SV=2	sp|Q92982|NINJ1_HUMAN	13.2	1	2.421	0.4893
2529	Osteoclast-stimulating factor 1 OS=Homo sapiens GN=OSTF1 PE=1 SV=2	sp|Q92882|OSTF1_HUMAN	5.6	1	1.2474	0.7777
2530	AP-3 complex subunit sigma-1 OS=Homo sapiens GN=AP3S1 PE=1 SV=1	sp|Q92572|AP3S1_HUMAN	6.2	1	1.1803	0.7175
2531	Ankyrin repeat domain-containing protein 49 OS=Homo sapiens GN=ANKRD49 PE=1 SV=1	sp|Q8WVL7|ANR49_HUMAN	5.4	1	0.6918	0.5981
2532	Small integral membrane protein 4 OS=Homo sapiens GN=SMIM4 PE=1	sp|Q8WVI0|SMIM4_HUMAN	15.7	1	0.7727	0.6343
2533	Serine/threonine/tyrosine-interacting protein OS=Homo sapiens GN=STYX PE=1 SV=1	sp|Q8WUJ0|STYX_HUMAN	4.5	1	0.7244	0.5642
2534	CDGSH iron-sulfur domain-containing protein 2 OS=Homo sapiens GN=CISD2 PE=1	sp|Q8N5K1|CISD2_HUMAN	10.4	1	0.9204	0.8721
2535	Chromatin complexes subunit BAP18 OS=Homo sapiens GN=BAP18 PE=1	sp|Q8IXM2|BAP18_HUMAN	5.8	1	0.8091	0.6847

2536	PHD finger protein 6 OS=Homo sapiens GN=PHF6 PE=1 SV=1	sp|Q8IWS0|PHF6_HUMAN	1.9	1	0.9462	0.9204
2537	Ubiquitin-conjugating enzyme E2 R2 OS=Homo sapiens GN=UBE2R2 PE=1 SV=1	sp|Q712K3|UB2R2_HUMAN	5.5	1	1.0093	0.9692
2538	60S ribosomal protein L22-like 1 OS=Homo sapiens GN=RPL22L1 PE=1 SV=2	sp|Q6P5R6|RL22L_HUMAN	9.8	1	0.879	0.8157
2539	Metal transporter CNNM4 OS=Homo sapiens GN=CNNM4 PE=1 SV=3	sp|Q6P4Q7|CNNM4_HUMAN	1.4	1	1.0765	0.8596
2540	Heme transporter HRG1 OS=Homo sapiens GN=SLC48A1 PE=1 SV=1	sp|Q6P1K1|HRG1_HUMAN	8.2	1	1.1912	0.7183
2541	60S ribosomal protein L7-like 1 OS=Homo sapiens GN=RPL7L1 PE=1 SV=1	sp|Q6DKI1|RL7L_HUMAN	7.3	1	0.6918	0.6218
2542	Protein Hikeshi OS=Homo sapiens GN=C11orf73 PE=1 SV=2	sp|Q53FT3|HIKES_HUMAN	4.6	2	0.9908	0.999
2543	Vacuolar ATPase assembly integral membrane protein VMA21 OS=Homo sapiens GN=VMA21 PE=1 SV=1	sp|Q3ZAQ7|VMA21_HUMAN	11.9	1	1.0093	0.9633
2544	Survival motor neuron protein OS=Homo sapiens GN=SMN1 PE=1 SV=1	sp|Q16637|SMN_HUMAN	6.1	1	0.8166	0.6962
2545	Male-enhanced antigen 1 OS=Homo sapiens GN=MEA1 PE=1	sp|Q16626|MEA1_HUMAN	7	1	0.863	0.7609
2546	Vesicle-associated membrane protein 3 OS=Homo sapiens GN=VAMP3 PE=1	sp|Q15836|VAMP3_HUMAN	16	1	1.2706	0.6215
2547	Mitochondrial import receptor subunit TOM20 homolog OS=Homo sapiens GN=TOMM20 PE=1 SV=1	sp|Q15388|TOM20_HUMAN	9	1	0.9376	0.9478
2548	Signal peptidase complex subunit 2 OS=Homo sapiens GN=SPCS2 PE=1 SV=3	sp|Q15005|SPCS2_HUMAN	3.5	1	1.2589	0.8556
2549	General transcription factor IIH subunit 3 OS=Homo sapiens GN=GTF2H3 PE=1 SV=2	sp|Q13889|TF2H3_HUMAN	2.3	1	1.0375	0.9229
2550	Angio-associated migratory cell protein OS=Homo sapiens GN=AAMP PE=1	sp|Q13685|AAMP_HUMAN	3.9	1	0.8472	0.761
2551	Mitotic spindle assembly checkpoint protein MAD2A OS=Homo sapiens GN=MAD2L1	sp|Q13257|MD2L1_HUMAN	7.8	1	1.1272	0.7863

2552	Chromatin assembly factor 1 subunit B OS=Homo sapiens GN=CHAF1B	sp|Q13112|CAF1B_HUMAN	2	1	0.3133	0.3686
2553	Vesicle transport protein SEC20 OS=Homo sapiens GN=BNIP1 PE=1 SV=3	sp|Q12981|SEC20_HUMAN	4.4	1	0.7516	0.6137
2554	Cyclin-dependent-like kinase 5 OS=Homo sapiens GN=CDK5 PE=1 SV=3	sp|Q00535|CDK5_HUMAN	4.8	1	1.1272	0.8042
2555	Serine/arginine-rich splicing factor 3 OS=Homo sapiens GN=SRSF3 PE=1 SV=1	sp|P84103|SRSF3_HUMAN	5.5	2	1.028	0.9845
2556	Neuron-specific calcium-binding protein hippocalcin OS=Homo sapiens GN=HPCA PE=2 SV=2	sp|P84074|HPCA_HUMAN	6.2	1	1.1376	0.776
2557	Solute carrier family 35 member B1 OS=Homo sapiens GN=SLC35B1 PE=1 SV=1	sp|P78383|S35B1_HUMAN	3.1	1	1.1066	0.8137
2558	DNA-directed RNA polymerase II subunit RPB7 OS=Homo sapiens GN=POLR2G PE=1 SV=1	sp|P62487|RPB7_HUMAN	6.4	1	0.929	0.8849
2559	Mitochondrial import inner membrane translocase subunit Tim10 OS=Homo sapiens GN=TIMM10 PE=1 SV=1	sp|P62072|TIM10_HUMAN	21.1	1	1.0666	0.8059
2560	Ubiquitin carboxyl-terminal hydrolase 46 OS=Homo sapiens GN=USP46 PE=1	sp|P62068|UBP46_HUMAN	4.9	1	1.1376	0.7065
2561	Signal peptidase complex subunit 3 OS=Homo sapiens GN=SPCS3 PE=1 SV=1	sp|P61009|SPCS3_HUMAN	6.1	1		
2562	Myelin proteolipid protein OS=Homo sapiens GN=PLP1 PE=1 SV=2	sp|P60201|MYPR_HUMAN	4.7	1	5.8614	0.6406
2563	Vesicle-associated membrane protein 7 OS=Homo sapiens GN=VAMP7 PE=1	sp|P51809|VAMP7_HUMAN	8.2	1	1.0471	0.9064
2564	Peroxisomal targeting signal 1 receptor OS=Homo sapiens GN=PEX5 PE=1 SV=3	sp|P50542|PEX5_HUMAN	3.4	1		
2565	Deoxyhypusine synthase OS=Homo sapiens GN=DHPS PE=1 SV=1	sp|P49366|DHYS_HUMAN	2.7	1	0.9204	0.882
2566	Phosphatidylserine synthase 1 OS=Homo sapiens GN=PTDSS1 PE=1 SV=1	sp|P48651|PTSS1_HUMAN	3	1	1.0471	0.9259
2567	Sorcin OS=Homo sapiens GN=SRI PE=1 SV=1	sp|P30626|SORCN_HUMAN	5.6	1	1.2359	0.6667

2568	Proteasome subunit alpha type-3 OS=Homo sapiens GN=PSMA3 PE=1 SV=2	sp|P25788|PSA3_HUMAN	5.5	2	1.028	0.9266
2569	U1 small nuclear ribonucleoprotein A OS=Homo sapiens GN=SNRPA PE=1	sp|P09012|SNRPA_HUMAN	6.4	1	1.0965	0.844
2570	NADH dehydrogenase [ubiquinone] 1 alpha subcomplex subunit 3 OS=Homo sapiens GN=NDUFA3	sp|O95167|NDUA3_HUMAN	13.1	1	1.0568	0.9013
2571	Adapter protein CIKS OS=Homo sapiens GN=TRAF3IP2 PE=1 SV=3	sp|O43734|CIKS_HUMAN	1.7	1	1	0.979
2572	Histone deacetylase complex subunit SAP18 OS=Homo sapiens GN=SAP18 PE=1	sp|O00422|SAP18_HUMAN	10.5	1	0.8395	0.7416
2573	Dynactin subunit 6 OS=Homo sapiens GN=DCTN6 PE=1 SV=1	sp|O00399|DCTN6_HUMAN	7.4	1	0.9817	0.9706
2574	Cytochrome c oxidase subunit 7C, mitochondrial OS=Homo sapiens GN=COX7C PE=1 SV=1	sp|P15954|COX7C_HUMAN	14.3	1	1.1695	0.7321
2575	Anaphase-promoting complex subunit 16 OS=Homo sapiens GN=ANAPC16 PE=1 SV=1	sp|Q96DE5|APC16_HUMAN	10.9	1	0.7178	0.5607
2576	F-box-like/WD repeat-containing protein TBL1X OS=Homo sapiens GN=TBL1X PE=1 SV=3	sp|O60907|TBL1X_HUMAN	5	1	1.028	0.9342
2577	Dehydrogenase/reductase SDR family member 13 OS=Homo sapiens GN=DHRS13 PE=2 SV=1	sp|Q6UX07|DHR13_HUMAN	4.5	2	0.8954	0.8323
2578	Ubiquitin-like protein 5 OS=Homo sapiens GN=UBL5 PE=1	sp|Q9BZL1|UBL5_HUMAN	12.3	1	0.6194	0.4401
2579	Cytosolic Fe-S cluster assembly factor NUBP2 OS=Homo sapiens GN=NUBP2 PE=1 SV=1	sp|Q9Y5Y2|NUBP2_HUMAN	13.7	1	1.0568	0.8993
2580	CXXC-type zinc finger protein 1 OS=Homo sapiens GN=CXXC1 PE=1 SV=2	sp|Q9P0U4|CXXC1_HUMAN	5.2	1	0.8872	0.8228
2581	Peptidyl-prolyl cis-trans isomerase F, mitochondrial OS=Homo sapiens GN=PPIF PE=1 SV=1	sp|P30405|PPIF_HUMAN	16.4	1	0.9638	0.948
2582	Iron-responsive element-binding protein 2 OS=Homo sapiens GN=IREB2 PE=1	sp|P48200|IREB2_HUMAN	2	1	0.8241	0.8674

2583	Coiled-coil domain-containing protein 50 OS=Homo sapiens GN=CCDC50	sp|Q8IVM0|CCD50_HUMAN	12.4	1	0.8954	0.8369
2584	Pleiotropic regulator 1 OS=Homo sapiens GN=PLRG1 PE=1	sp|O43660|PLRG1_HUMAN	7.6	1	0.955	0.946
2585	Vacuolar fusion protein MON1 homolog A OS=Homo sapiens GN=MON1A PE=1 SV=2	sp|Q86VX9|MON1A_HUMAN	3.1	1	1.0471	0.8508
2586	Coiled-coil domain-containing protein 43 OS=Homo sapiens GN=CCDC43	sp|Q96MW1|CCD43_HUMAN	7.6	1	1.1803	0.7314
2587	RNA-binding protein 8A OS=Homo sapiens GN=RBM8A PE=1 SV=1	sp|Q9Y5S9|RBM8A_HUMAN	10.9	1	0.863	0.7756
2588	Protein YIPF6 OS=Homo sapiens GN=YIPF6 PE=1 SV=2	sp|Q96EC8|YIPF6_HUMAN	5.1	1	1.0765	0.8454
2589	Transcriptional repressor p66-beta OS=Homo sapiens GN=GATAD2B PE=1 SV=1	sp|Q8WXI9|P66B_HUMAN	4.4	3	1.0186	0.9671
2590	Conserved oligomeric Golgi complex subunit 2 OS=Homo sapiens GN=COG2 PE=1 SV=1	sp|Q14746|COG2_HUMAN	5.6	1	1.1482	0.7726
2591	MARCKS-related protein OS=Homo sapiens GN=MARCKSL1 PE=1 SV=2	sp|P49006|MRP_HUMAN	16.9	1	1.8707	0.3468
2592	Transcription elongation factor SPT4 OS=Homo sapiens GN=SUPT4H1 PE=1 SV=1	sp|P63272|SPT4H_HUMAN	13.7	1	0.8091	0.6854
2593	Immunoglobulin-like domain-containing receptor 2 OS=Homo sapiens GN=ILDR2 PE=2 SV=1	sp|Q71H61|ILDR2_HUMAN	5.2	1	0.8954	0.8397
2594	Glycolipid transfer protein OS=Homo sapiens GN=GLTP PE=1 SV=3	sp|Q9NZD2|GLTP_HUMAN	5.7	1	0.9727	0.9593
2595	Leucine-rich repeat serine/threonine-protein kinase 2 OS=Homo sapiens GN=LRRK2 PE=1 SV=2	sp|Q5S007|LRRK2_HUMAN	4.1	2		
2596	Kinesin-like protein KIF2C OS=Homo sapiens GN=KIF2C PE=1	sp|Q99661|KIF2C_HUMAN	6.2	3	0.5702	0.383
2597	Neuropathy target esterase OS=Homo sapiens GN=PNPLA6 PE=1 SV=2	sp|Q8IY17|PLPL6_HUMAN	4.7	2	1.1803	0.7321
2598	Protein LDOC1 OS=Homo sapiens GN=LDOC1 PE=1 SV=1	sp|O95751|LDOC1_HUMAN	9.6	1	0.955	0.9533

2599	5'-nucleotidase domain-containing protein 3 OS=Homo sapiens GN=NT5DC3 PE=2 SV=1	sp|Q86UY8|NT5D3_HUMAN	3.5	1	0.9638	0.9257
2600	Putative 60S ribosomal protein L39-like 5 OS=Homo sapiens GN=RPL39P5 PE=5 SV=2	sp|Q59GN2|R39L5_HUMAN	19.6	2	0.8472	0.9223
2601	Integrator complex subunit 7 OS=Homo sapiens GN=INTS7 PE=1	sp|Q9NVH2|INT7_HUMAN	6.7	1	1.0186	0.8649
2602	Neurobeachin-like protein 2 OS=Homo sapiens GN=NBEAL2 PE=1 SV=2	sp|Q6ZNJ1|NBEL2_HUMAN	5.7	3	0.912	0.9042
2603	Adenylate kinase isoenzyme 1 OS=Homo sapiens GN=AK1 PE=1 SV=3	sp|P00568|KAD1_HUMAN	13.4	1	1.1066	0.8236
2604	Transcription elongation factor B polypeptide 3 OS=Homo sapiens GN=TCEB3 PE=1 SV=2	sp|Q14241|ELOA1_HUMAN	6.4	2	0.9638	0.9718
2605	Protein CIP2A OS=Homo sapiens GN=KIAA1524 PE=1 SV=2	sp|Q8TCG1|CIP2A_HUMAN	2.5	1	0.6026	0.4214
2606	Protein TBRG4 OS=Homo sapiens GN=TBRG4 PE=1 SV=1	sp|Q969Z0|TBRG4_HUMAN	10.1	5	0.9462	0.9395
2607	Serine/threonine-protein kinase TAO3 OS=Homo sapiens GN=TAOK3 PE=1	sp|Q9H2K8|TAOK3_HUMAN	2.8	1	1	0.9714
2608	Uncharacterized protein CXorf38 OS=Homo sapiens GN=CXorf38 PE=1 SV=1	sp|Q8TB03|CX038_HUMAN	4.4	2	0.9638	0.962
2609	Transmembrane and coiled-coil domain-containing protein 1 OS=Homo sapiens GN=TMCO1 PE=1 SV=1	sp|Q9UM00|TMCO1_HUMAN	4.3	1	1.1376	0.7826
2610	Prefoldin subunit 1 OS=Homo sapiens GN=PFDN1 PE=1 SV=2	sp|O60925|PFD1_HUMAN	13.1	1	0.9204	0.8876
2611	2'-deoxynucleoside 5'-phosphate N-hydrolase 1 OS=Homo sapiens GN=DNPH1 PE=1 SV=1	sp|O43598|DNPH1_HUMAN	6.9	1	0.9908	0.9826
2612	Transformation/transcription domain-associated protein OS=Homo sapiens GN=TRRAP PE=1 SV=3	sp|Q9Y4A5|TRRAP_HUMAN	3.4	4	0.787	0.2855
2613	Bromodomain-containing protein 8 OS=Homo sapiens GN=BRD8 PE=1 SV=2	sp|Q9H0E9|BRD8_HUMAN	3.2	1	1.2023	0.5889
2614	DNA polymerase epsilon subunit 2 OS=Homo sapiens GN=POLE2 PE=1 SV=2	sp|P56282|DPOE2_HUMAN	5.5	1	0.879	0.7951

2615	Heat shock protein beta-8 OS=Homo sapiens GN=HSPB8 PE=1	sp|Q9UJY1|HSPB8_HUMAN	10.2	1	1.2359	0.6692
2616	Nurim OS=Homo sapiens GN=NRM PE=1 SV=1	sp|Q8IXM6|NRM_HUMAN	9.2	1	0.7798	0.639
2617	Translin-associated protein X OS=Homo sapiens GN=TSNAX PE=1 SV=1	sp|Q99598|TSNAX_HUMAN	10.7	1	0.9908	0.9988
2618	Transmembrane protein 168 OS=Homo sapiens GN=TMEM168 PE=2 SV=2	sp|Q9H0V1|TM168_HUMAN	3.9	1	1	0.9063
2619	Merlin OS=Homo sapiens GN=NF2 PE=1 SV=1	sp|P35240|MERL_HUMAN	11.4	5	0.9462	0.8831
2620	High mobility group protein B1 OS=Homo sapiens GN=HMGB1 PE=1 SV=3	sp|P09429|HMGB1_HUMAN	18.6	7	0.7943	0.7477
2621	Syntaxin-binding protein 3 OS=Homo sapiens GN=STXBP3 PE=1 SV=2	sp|O00186|STXB3_HUMAN	6.3	2	1.3552	0.5667
2622	Propionyl-CoA carboxylase beta chain, mitochondrial OS=Homo sapiens GN=PCCB PE=1	sp|P05166|PCCB_HUMAN	5.6	1	1.1272	0.7916
2623	Growth arrest-specific protein 7 OS=Homo sapiens GN=GAS7 PE=1 SV=3	sp|O60861|GAS7_HUMAN	10.7	2	1.1803	0.7319
2624	Alpha-ketoglutarate-dependent dioxygenase alkB homolog 4 OS=Homo sapiens GN=ALKBH4	sp|Q9NXW9|ALKB4_HUMAN	5.3	1	0.9638	0.8975
2625	Shootin-1 OS=Homo sapiens GN=SHTN1 PE=1	sp|A0MZ66|SHOT1_HUMAN	8.2	1	0.929	0.8347
2626	Cytochrome c oxidase subunit NDUFA4 OS=Homo sapiens GN=NDUFA4 PE=1 SV=1	sp|O00483|NDUA4_HUMAN	44.4	3	0.9036	0.8466
2627	Keratin, type II cytoskeletal 5 OS=Homo sapiens GN=KRT5 PE=1 SV=3	sp|P13647|K2C5_HUMAN	5.3	2	0.8166	0.692
2628	Ras GTPase-activating-like protein IQGAP3 OS=Homo sapiens GN=IQGAP3 PE=1 SV=2	sp|Q86VI3|IQGA3_HUMAN	5.8	2	0.2754	0.2644
2629	Guanine nucleotide-binding protein G(I)/G(S)/G(O) subunit gamma-5 OS=Homo sapiens GN=GNG5 PE=1	sp|P63218|GBG5_HUMAN	10.3	1	1.3428	0.5821
2630	Long-chain fatty acid transport protein 1 OS=Homo sapiens GN=SLC27A1 PE=2 SV=1	sp|Q6PCB7|S27A1_HUMAN	7.3	2	1.1169	0.6023

2631	Histone lysine demethylase PHF8 OS=Homo sapiens GN=PHF8 PE=1 SV=3	sp|Q9UPP1|PHF8_HUMAN	4.1	2	0.879	0.8081
2632	SH3 and PX domain-containing protein 2B OS=Homo sapiens GN=SH3PXD2B PE=1 SV=3	sp|A1X283|SPD2B_HUMAN	4.5	1	1.1272	0.7944
2633	Transmembrane protein 11, mitochondrial OS=Homo sapiens GN=TMEM11 PE=1 SV=1	sp|P17152|TMM11_HUMAN	10.4	1	0.912	0.8577
2634	Kinesin-associated protein 3 OS=Homo sapiens GN=KIFAP3 PE=1 SV=2	sp|Q92845|KIFA3_HUMAN	4.2	2	1.1066	0.8255
2635	Polycomb protein EED OS=Homo sapiens GN=EED PE=1 SV=2	sp|O75530|EED_HUMAN	3.9	2	0.8241	0.7217
2636	Disheveled-associated activator of morphogenesis 1 OS=Homo sapiens GN=DAAM1 PE=1 SV=2	sp|Q9Y4D1|DAAM1_HUMAN	4.5	2	1.6904	0.4851
2637	Torsin-1A-interacting protein 2, isoform IFRG15 OS=Homo sapiens GN=TOR1AIP2 PE=1 SV=1	sp|Q9H496|IFG15_HUMAN	13.7	1	0.9462	0.9073
2638	NK-tumor recognition protein OS=Homo sapiens GN=NKTR PE=1 SV=2	sp|P30414|NKTR_HUMAN	2.5	1	0.955	0.9467
2639	Myeloid-associated differentiation marker OS=Homo sapiens GN=MYADM PE=1 SV=2	sp|Q96S97|MYADM_HUMAN	4.3	1	1.7219	0.5694
2640	Inward rectifier potassium channel 16 OS=Homo sapiens GN=KCNJ16 PE=2 SV=1	sp|Q9NPI9|KCJ16_HUMAN	4.1	1	0.6855	0.5195
2641	FH1/FH2 domain-containing protein 1 OS=Homo sapiens GN=FHOD1 PE=1	sp|Q9Y613|FHOD1_HUMAN	4.6	2	1.0965	0.8352
2642	INO80 complex subunit C OS=Homo sapiens GN=INO80C PE=1 SV=1	sp|Q6PI98|IN80C_HUMAN	7.3	1	1.0666	0.8547
2643	Echinoderm microtubule-associated protein-like 2 OS=Homo sapiens GN=EML2 PE=1 SV=1	sp|O95834|EMAL2_HUMAN	4.6	3	0.673	0.4987
2644	Endoplasmic reticulum-Golgi intermediate compartment protein 2 OS=Homo sapiens GN=ERGIC2 PE=1 SV=2	sp|Q96RQ1|ERGI2_HUMAN	2.1	1	1.0471	0.9118
2645	Desmocollin-3 OS=Homo sapiens GN=DSC3 PE=1 SV=3	sp|Q14574|DSC3_HUMAN	4.2	2	0.9036	0.8533

2646	Histone-lysine N-methyltransferase EZH2 OS=Homo sapiens GN=EZH2 PE=1 SV=2	sp|Q15910|EZH2_HUMAN	7.2	2	0.8318	0.609
2647	Calcium-binding mitochondrial carrier protein SCaMC-3 OS=Homo sapiens GN=SLC25A23 PE=1 SV=2	sp|Q9BV35|SCMC3_HUMAN	9.8	1	1.5996	0.4392
2648	Nuclear factor of activated T-cells, cytoplasmic 2 OS=Homo sapiens GN=NFATC2 PE=1 SV=2	sp|Q13469|NFAC2_HUMAN	3	1	0.8551	0.752
2649	Alpha-adducin OS=Homo sapiens GN=ADD1 PE=1 SV=2	sp|P35611|ADDA_HUMAN	3.1	1	0.8954	0.8408
2650	Neuroblastoma-amplified sequence OS=Homo sapiens GN=NBAS PE=1 SV=2	sp|A2RRP1|NBAS_HUMAN	5.2	1	0.9036	0.6728
2651	REVERSED Dynein heavy chain 9, axonemal OS=Homo sapiens GN=DNAH9 PE=1 SV=3	RRRRRsp|Q9NYC9|DYH9_HUMAN	2.4	2	0.8872	0.8158
2652	DNA-directed RNA polymerase III subunit RPC10 OS=Homo sapiens GN=POLR3K PE=1 SV=2	sp|Q9Y2Y1|RPC10_HUMAN	8.3	1	0.8954	0.8452
2653	TBC1 domain family member 17 OS=Homo sapiens GN=TBC1D17 PE=1 SV=2	sp|Q9HA65|TBC17_HUMAN	6.9	2	0.9727	0.9535
2654	SWI/SNF-related matrix-associated actin-dependent regulator of chromatin subfamily A-like protein 1 OS=Homo sapiens GN=SMARCAL1 PE=1 SV=1	sp|Q9NZC9|SMAL1_HUMAN	5.7	1	0.879	0.6412
2655	Ubiquitin-fold modifier-conjugating enzyme 1 OS=Homo sapiens GN=UFC1 PE=1	sp|Q9Y3C8|UFC1_HUMAN	7.8	1	0.871	0.7893
2656	U6 snRNA-associated Sm-like protein LSm7 OS=Homo sapiens GN=LSM7 PE=1 SV=1	sp|Q9UK45|LSM7_HUMAN	25.2	1		
2657	Protein PRRC2C OS=Homo sapiens GN=PRRC2C PE=1 SV=4	sp|Q9Y520|PRC2C_HUMAN	2.6	2	0.863	0.781
2658	Nuclear factor of activated T-cells, cytoplasmic 1 OS=Homo sapiens GN=NFATC1 PE=1 SV=3	sp|O95644|NFAC1_HUMAN	5.1	1	0.863	0.7862
2659	Kinesin light chain 2 OS=Homo sapiens GN=KLC2 PE=1 SV=1	sp|Q9H0B6|KLC2_HUMAN	4.5	1	1.1803	0.7272

2660	Mitogen-activated protein kinase kinase kinase kinase 4 OS=Homo sapiens GN=MAP4K4	sp|O95819|M4K4_HUMAN	4	3	0.9908	0.9854
2661	Prolyl endopeptidase-like OS=Homo sapiens GN=PREPL PE=1 SV=1	sp|Q4J6C6|PPCEL_HUMAN	3.9	1	1.028	0.9371
2662	Uncharacterized protein KIAA0513 OS=Homo sapiens GN=KIAA0513 PE=2 SV=1	sp|O60268|K0513_HUMAN	7.3	1	0.7727	0.6338
2663	Ubiquitin carboxyl-terminal hydrolase 19 OS=Homo sapiens GN=USP19 PE=1	sp|O94966|UBP19_HUMAN	4.6	2	0.8241	0.7152
2664	Grancalcin OS=Homo sapiens GN=GCA PE=1 SV=2	sp|P28676|GRAN_HUMAN	3.2	1	1.0568	0.8887
2665	Serine/threonine-protein kinase MRCK beta OS=Homo sapiens GN=CDC42BPB PE=1 SV=2	sp|Q9Y5S2|MRCKB_HUMAN	2.9	3	1.0375	0.9637
2666	Muskelin OS=Homo sapiens GN=MKLN1 PE=1 SV=2	sp|Q9UL63|MKLN1_HUMAN	1.6	1	0.9204	0.8718
2667	Von Hippel-Lindau disease tumor suppressor OS=Homo sapiens GN=VHL PE=1 SV=2	sp|P40337|VHL_HUMAN	3.3	1	0.955	0.9022
2668	39S ribosomal protein L46, mitochondrial OS=Homo sapiens GN=MRPL46 PE=1 SV=1	sp|Q9H2W6|RM46_HUMAN	5.4	1	0.8954	0.8326
2669	Kelch repeat and BTB domain-containing protein 11 OS=Homo sapiens GN=KBTBD11	sp|O94819|KBTBB_HUMAN	7.9	1	0.6194	0.5405
2670	Calcium uniporter protein, mitochondrial OS=Homo sapiens GN=MCU PE=1 SV=1	sp|Q8NE86|MCU_HUMAN	12	1	1.0471	0.9155
2671	Ovarian cancer-associated gene 2 protein OS=Homo sapiens GN=OVCA2 PE=1 SV=1	sp|Q8WZ82|OVCA2_HUMAN	6.6	1	0.955	0.9459
2672	Formin-binding protein 4 OS=Homo sapiens GN=FNBP4 PE=1	sp|Q8N3X1|FNBP4_HUMAN	2	1	0.8395	0.7012
2673	MIP18 family protein FAM96A OS=Homo sapiens GN=FAM96A PE=1 SV=1	sp|Q9H5X1|FA96A_HUMAN	10.6	1	0.8017	0.9118
2674	Phosphorylated CTD-interacting factor 1 OS=Homo sapiens GN=PCIF1 PE=1 SV=1	sp|Q9H4Z3|PCIF1_HUMAN	3.8	1	1.0186	0.9514

2675	Aryl hydrocarbon receptor nuclear translocator OS=Homo sapiens GN=ARNT PE=1 SV=1	sp|P27540|ARNT_HUMAN	4.9	1	0.871	0.8041
2676	Advillin OS=Homo sapiens GN=AVIL PE=1 SV=3	sp|O75366|AVIL_HUMAN	2.8	1	1.5136	0.4788
2677	Mothers against decapentaplegic homolog 5 OS=Homo sapiens GN=SMAD5 PE=1 SV=1	sp|Q99717|SMAD5_HUMAN	11.8	3	1.0765	0.8811
2678	Phosphatase and actin regulator 4 OS=Homo sapiens GN=PHACTR4 PE=1 SV=1	sp|Q8IZ21|PHAR4_HUMAN	4.8	1	0.9817	0.9824
2679	Rab GDP dissociation inhibitor alpha OS=Homo sapiens GN=GDI1 PE=1	sp|P31150|GDIA_HUMAN	39.6	22		
2680	HERV-H LTR-associating protein 2 OS=Homo sapiens GN=HHLA2 PE=1	sp|Q9UM44|HHLA2_HUMAN	3.6	1		
2681	Activating signal cointegrator 1 OS=Homo sapiens GN=TRIP4 PE=1 SV=4	sp|Q15650|TRIP4_HUMAN	4.3	1	1.1482	0.7572
2682	F-box only protein 33 OS=Homo sapiens GN=FBXO33 PE=1 SV=1	sp|Q7Z6M2|FBX33_HUMAN	5.9	1	0.7244	0.5737
2683	40S ribosomal protein S28 OS=Homo sapiens GN=RPS28 PE=1 SV=1	sp|P62857|RS28_HUMAN	52.2	5	1.3183	0.5967
2684	39S ribosomal protein L12, mitochondrial OS=Homo sapiens GN=MRPL12 PE=1 SV=2	sp|P52815|RM12_HUMAN	17.2	1	0.912	0.8623
2685	YTH domain-containing protein 1 OS=Homo sapiens GN=YTHDC1	sp|Q96MU7|YTDC1_HUMAN	5	1	1.0093	0.9291
2686	40S ribosomal protein S20 OS=Homo sapiens GN=RPS20 PE=1 SV=1	sp|P60866|RS20_HUMAN	14.3	2	1.3183	0.5864
2687	ADP-ribosylation factor 4 OS=Homo sapiens GN=ARF4 PE=1 SV=3	sp|P18085|ARF4_HUMAN	31.7	6	1.1169	0.8134
2688	Bromodomain-containing protein 4 OS=Homo sapiens GN=BRD4 PE=1 SV=2	sp|O60885|BRD4_HUMAN	3.4	3	0.8551	0.7669
2689	Phosphoinositide 3-kinase regulatory subunit 5 OS=Homo sapiens GN=PIK3R5 PE=1 SV=1	sp|Q8WYR1|PI3R5_HUMAN	5.5	1	1.0471	0.9136
2690	Semaphorin-3E OS=Homo sapiens GN=SEMA3E PE=1 SV=1	sp|O15041|SEM3E_HUMAN	7.1	2	1.803	0.3681
2691	Borealin OS=Homo sapiens GN=CDCA8 PE=1	sp|Q53HL2|BOREA_HUMAN	10.7	1	0.6427	0.4695

2692	THO complex subunit 5 homolog OS=Homo sapiens GN=THOC5 PE=1 SV=2	sp|Q13769|THOC5_HUMAN	4	1	1.028	0.9429
2693	cAMP-specific 3',5'-cyclic phosphodiesterase 4D OS=Homo sapiens GN=PDE4D PE=1	sp|Q08499|PDE4D_HUMAN	2.8	1	1.3677	0.5418
2694	REVERSED Zinc finger protein 40 OS=Homo sapiens GN=HIVEP1 PE=1 SV=3	RRRRRsp|P15822|ZEP1_HUMAN	1.3	1	0.9204	0.8915
2695	Lipopolysaccharide-responsive and beige-like anchor protein OS=Homo sapiens GN=LRBA PE=1 SV=4	sp|P50851|LRBA_HUMAN	4.9	2	1.1272	0.7795
2696	UAP56-interacting factor OS=Homo sapiens GN=FYTTD1 PE=1 SV=3	sp|Q96QD9|UIF_HUMAN	11.6	1	1.0375	0.9219
2697	Myosin-3 OS=Homo sapiens GN=MYH3 PE=1 SV=3	sp|P11055|MYH3_HUMAN	2.3	2	0.6546	0.4822
2698	Mitogen-activated protein kinase kinase kinase 3 OS=Homo sapiens GN=MAP3K3 PE=1 SV=2	sp|Q99759|M3K3_HUMAN	6.1	1	1.1066	0.4115
2699	RNA-binding protein 4 OS=Homo sapiens GN=RBM4 PE=1	sp|Q9BWF3|RBM4_HUMAN	4.9	1	0.8872	0.7965
2700	Rab-like protein 6 OS=Homo sapiens GN=RABL6 PE=1 SV=2	sp|Q3YEC7|RABL6_HUMAN	8.4	1	0.879	0.4117
2701	Probable cation-transporting ATPase 13A3 OS=Homo sapiens GN=ATP13A3 PE=1 SV=4	sp|Q9H7F0|AT133_HUMAN	3	1	0.9036	0.6825
2702	Clathrin light chain A OS=Homo sapiens GN=CLTA PE=1 SV=1	sp|P09496|CLCA_HUMAN	4.4	1	1	0.8794
2703	Zinc finger ZZ-type and EF-hand domain-containing protein 1 OS=Homo sapiens GN=ZZEF1 PE=1	sp|O43149|ZZEF1_HUMAN	4	4	1.3062	0.5817
2704	Kinesin-like protein KIF20B OS=Homo sapiens GN=KIF20B PE=1	sp|Q96Q89|KI20B_HUMAN	3.8	2	0.2148	0.1947
2705	Tripartite motif-containing protein 26 OS=Homo sapiens GN=TRIM26 PE=1	sp|Q12899|TRI26_HUMAN	4.1	1	0.6792	0.5052
2706	Cytoplasmic tRNA 2-thiolation protein 2 OS=Homo sapiens GN=CTU2 PE=1 SV=1	sp|Q2VPK5|CTU2_HUMAN	5	1	0.8472	0.7591
2707	Protein YIPF3 OS=Homo sapiens GN=YIPF3 PE=1 SV=1	sp|Q9GZM5|YIPF3_HUMAN	4.9	1	1.1169	0.8098
2708	Sentrin-specific protease 7 OS=Homo sapiens GN=SENP7 PE=1 SV=4	sp|Q9BQF6|SENP7_HUMAN	3.3	1	1.1482	0.7887

2709	Histidine decarboxylase OS=Homo sapiens GN=HDC PE=1 SV=2	sp|P19113|DCHS_HUMAN	4.4	1	1	0.9916
2710	Protein kish-A OS=Homo sapiens GN=TMEM167A PE=1 SV=1	sp|Q8TBQ9|KISHA_HUMAN	12.5	1	1.1912	0.7205
2711	Acylphosphatase-1 OS=Homo sapiens GN=ACYP1 PE=1 SV=2	sp|P07311|ACYP1_HUMAN	17.2	1	1.0965	0.8385
2712	Cysteine-rich hydrophobic domain-containing protein 2 OS=Homo sapiens GN=CHIC2 PE=1	sp|Q9UKJ5|CHIC2_HUMAN	3.6	1	1.0471	0.9177
2713	RING finger protein 219 OS=Homo sapiens GN=RNF219 PE=1 SV=1	sp|Q5W0B1|RN219_HUMAN	2.1	1	0.871	0.7766
2714	Mediator of RNA polymerase II transcription subunit 23 OS=Homo sapiens GN=MED23 PE=1	sp|Q9ULK4|MED23_HUMAN	4.5	2	0.871	0.7559
2715	Elongator complex protein 1 OS=Homo sapiens GN=IKBKAP PE=1 SV=3	sp|O95163|ELP1_HUMAN	4.4	3	0.912	0.8688
2716	KRR1 small subunit processome component homolog OS=Homo sapiens GN=KRR1 PE=1	sp|Q13601|KRR1_HUMAN	3.9	1	0.9376	0.9091
2717	Hypermethylated in cancer 2 protein OS=Homo sapiens GN=HIC2 PE=1 SV=2	sp|Q96JB3|HIC2_HUMAN	5.7	2	0.5346	0.4819
2718	Methylmalonyl-CoA epimerase, mitochondrial OS=Homo sapiens GN=MCEE PE=1 SV=1	sp|Q96PE7|MCEE_HUMAN	6.8	1	1.0765	0.8706
2719	NADH dehydrogenase [ubiquinone] 1 beta subcomplex subunit 9 OS=Homo sapiens GN=NDUFB9	sp|Q9Y6M9|NDUB9_HUMAN	20.1	1	1.0375	0.9202
2720	Proline-rich protein PRCC OS=Homo sapiens GN=PRCC PE=1 SV=1	sp|Q92733|PRCC_HUMAN	3.5	1	1.0568	0.9094
2721	Polyribonucleotide 5'-hydroxyl-kinase Clp1 OS=Homo sapiens GN=CLP1 PE=1 SV=1	sp|Q92989|CLP1_HUMAN	5.9	1	0.8954	0.8173
2722	Death-associated protein 1 OS=Homo sapiens GN=DAP PE=1	sp|P51397|DAP1_HUMAN	15.7	1	1.2706	0.6408
2723	B-cell CLL/lymphoma 7 protein family member A OS=Homo sapiens GN=BCL7A PE=1 SV=1	sp|Q4VC05|BCL7A_HUMAN	9.5	1	0.7244	0.5672
2724	Ubiquitin carboxyl-terminal hydrolase 36 OS=Homo sapiens GN=USP36 PE=1	sp|Q9P275|UBP36_HUMAN	4.4	1	0.5152	0.5085

2725	Delphilin OS=Homo sapiens GN=GRID2IP PE=3 SV=2	sp|A4D2P6|GRD2I_HUMAN	1.6	2	1.5849	0.4392
2726	Histone-lysine N-methyltransferase SMYD3 OS=Homo sapiens GN=SMYD3 PE=1 SV=4	sp|Q9H7B4|SMYD3_HUMAN	5.4	2	0.9376	0.8161
2727	Dynein regulatory complex subunit 7 OS=Homo sapiens GN=DRC7 PE=1 SV=3	sp|Q8IY82|DRC7_HUMAN	1.6	1	0.9727	0.9831
2728	Apolipoprotein B-100 OS=Homo sapiens GN=APOB PE=1 SV=2	sp|P04114|APOB_HUMAN	3.3	2	1.1588	0.7636
2729	NSFL1 cofactor p47 OS=Homo sapiens GN=NSFL1C	sp|Q9UNZ2|NSF1C_HUMAN	23	5	0.9817	0.9994
2730	TOM1-like protein 2 OS=Homo sapiens GN=TOM1L2	sp|Q6ZVM7|TM1L2_HUMAN	6.1	1	1.1482	0.7737
2731	39S ribosomal protein L4, mitochondrial OS=Homo sapiens GN=MRPL4 PE=1 SV=1	sp|Q9BYD3|RM04_HUMAN	6.1	1	0.8318	0.9154
2732	Beta-1,4-galactosyltransferase 1 OS=Homo sapiens GN=B4GALT1 PE=1 SV=5	sp|P15291|B4GT1_HUMAN	3.3	1	1.0375	0.9287
2733	Beta-1,4-galactosyltransferase 5 OS=Homo sapiens GN=B4GALT5 PE=2 SV=1	sp|O43286|B4GT5_HUMAN	5.7	1	1.0093	0.9767
2734	Cyclin-dependent kinase 19 OS=Homo sapiens GN=CDK19 PE=1	sp|Q9BWU1|CDK19_HUMAN	3	1	0.8551	0.7714
2735	REVERSED Autophagy-related protein 2 homolog B OS=Homo sapiens GN=ATG2B PE=1	RRRRRsp|Q96BY7|ATG2B_HUMAN	4.2	1	0.3802	0.2465
2736	REVERSED Serine/threonine-protein phosphatase 2A 65 kDa regulatory subunit A alpha isoform OS=Homo sapiens GN=PPP2R1A PE=1 SV=4	RRRRRsp|P30153|2AAA_HUMAN	7.3	1	0.879	0.7988
2737	Cytochrome b5 reductase 4 OS=Homo sapiens GN=CYB5R4 PE=1 SV=1	sp|Q7L1T6|NB5R4_HUMAN	1.2	1	1.0965	0.8448
2738	Sodium channel protein type 8 subunit alpha OS=Homo sapiens GN=SCN8A PE=1 SV=1	sp|Q9UQD0|SCN8A_HUMAN	3.9	1	0.9817	0.9
2739	Chromodomain-helicase-DNA-binding protein 1 OS=Homo sapiens GN=CHD1 PE=1 SV=2	sp|O14646|CHD1_HUMAN	3.6	2	0.7586	0.6147

2740	WD repeat-containing protein 48 OS=Homo sapiens GN=WDR48 PE=1	sp|Q8TAF3|WDR48_HUMAN	3.3	1	0.7379	0.5866
2741	Solute carrier family 12 member 9 OS=Homo sapiens GN=SLC12A9	sp|Q9BXP2|S12A9_HUMAN	6	3	1.3305	0.5864
2742	PCNA-associated factor OS=Homo sapiens GN=KIAA0101 PE=1 SV=1	sp|Q15004|PAF15_HUMAN	16.2	1	0.2805	0.2226
2743	[3-methyl-2-oxobutanoate dehydrogenase [lipoamide]] kinase, mitochondrial OS=Homo sapiens GN=BCKDK PE=1	sp|O14874|BCKD_HUMAN	4.9	2		
2744	DEP domain-containing protein 5 OS=Homo sapiens GN=DEPDC5	sp|O75140|DEPD5_HUMAN	2.1	1	1.0666	0.8955
2745	XK-related protein 7 OS=Homo sapiens GN=XKR7 PE=2 SV=1	sp|Q5GH72|XKR7_HUMAN	3.5	1	0.8551	0.7798
2746	Ras-related protein Rab-8B OS=Homo sapiens GN=RAB8B PE=1	sp|Q92930|RAB8B_HUMAN	16.4	1	1.1376	0.7872
2747	Prothrombin OS=Homo sapiens GN=F2 PE=1	sp|P00734|THRB_HUMAN	1.9	1	0.7516	0.6214
2748	NEDD4-like E3 ubiquitin-protein ligase WWP2 OS=Homo sapiens GN=WWP2 PE=1 SV=2	sp|O00308|WWP2_HUMAN	4.1	1	1.1376	0.88
2749	N-acetylglucosamine-1-phosphotransferase subunits alpha/beta OS=Homo sapiens GN=GNPTAB PE=1 SV=1	sp|Q3T906|GNPTA_HUMAN	4	2	0.8954	0.8751
2750	Multifunctional methyltransferase subunit TRM112-like protein OS=Homo sapiens GN=TRMT112 PE=1 SV=1	sp|Q9UI30|TR112_HUMAN	24.8	2	0.4246	0.6219
2751	Nuclear receptor coactivator 2 OS=Homo sapiens GN=NCOA2 PE=1 SV=2	sp|Q15596|NCOA2_HUMAN	4.7	2	0.9817	0.9839
2752	Williams-Beuren syndrome chromosomal region 16 protein OS=Homo sapiens GN=WBSCR16 PE=1 SV=2	sp|Q96I51|WBS16_HUMAN	5.4	1	0.8318	0.7345
2753	Guanosine-3',5'-bis(diphosphate) 3'-pyrophosphohydrolase MESH1 OS=Homo sapiens GN=HDDC3 PE=1 SV=3	sp|Q8N4P3|MESH1_HUMAN	16.8	2	0.8872	0.8181
2754	Integrator complex subunit 11 OS=Homo sapiens GN=CPSF3L PE=1 SV=2	sp|Q5TA45|INT11_HUMAN	3.5	1	0.929	0.8999

2755	Acyl-coenzyme A thioesterase 8 OS=Homo sapiens GN=ACOT8 PE=1 SV=1	sp|O14734|ACOT8_HUMAN	1.9	1	1.0765	0.8528
2756	Putative uncharacterized protein encoded by LINC01559 OS=Homo sapiens GN=LINC01559 PE=2 SV=1	sp|Q495D7|CL036_HUMAN	12.3	1	1.1803	0.7372
2757	Cyclin-dependent kinase 2 OS=Homo sapiens GN=CDK2 PE=1 SV=2	sp|P24941|CDK2_HUMAN	6.4	1	1.1912	0.7252
2758	Dual specificity mitogen-activated protein kinase kinase 1 OS=Homo sapiens GN=MAP2K1	sp|Q02750|MP2K1_HUMAN	17.6	3	1.0375	0.8154
2759	Ribonucleoside-diphosphate reductase large subunit OS=Homo sapiens GN=RRM1 PE=1 SV=1	sp|P23921|RIR1_HUMAN	6.9	2	0.5012	0.1233
2760	Tyrosine-protein kinase Tec OS=Homo sapiens GN=TEC PE=1 SV=2	sp|P42680|TEC_HUMAN	3.6	1	0.9462	0.921
2761	Zinc finger matrin-type protein 2 OS=Homo sapiens GN=ZMAT2 PE=1 SV=1	sp|Q96NC0|ZMAT2_HUMAN	8	1	0.5248	0.351
2762	Perilipin-3 OS=Homo sapiens GN=PLIN3 PE=1 SV=3	sp|O60664|PLIN3_HUMAN	3.5	1	1.2589	0.6391
2763	REVERSED Coiled-coil domain-containing protein 178 OS=Homo sapiens GN=CCDC178 PE=2 SV=3	RRRRRsp|Q5BJE1|CC178_HUMAN	0.8	1	0.9727	0.9735
2764	Copine-2 OS=Homo sapiens GN=CPNE2 PE=1	sp|Q96FN4|CPNE2_HUMAN	2.7	2	1.2942	0.6036
2765	REVERSED Fibrous sheath-interacting protein 2 OS=Homo sapiens GN=FSIP2	RRRRRsp|Q5CZC0|FSIP2_HUMAN	2	3	3.4356	0.249
2766	Ral GTPase-activating protein subunit alpha-1 OS=Homo sapiens GN=RALGAPA1 PE=1 SV=1	sp|Q6GYQ0|RGPA1_HUMAN	3.6	2	1.0471	0.9217
2767	Glutamine-rich protein 1 OS=Homo sapiens GN=QRICH1 PE=1 SV=1	sp|Q2TAL8|QRIC1_HUMAN	3.9	1	1.1272	0.8033
2768	DNA primase small subunit OS=Homo sapiens GN=PRIM1 PE=1	sp|P49642|PRI1_HUMAN	7.9	1	0.7798	0.6537
2769	HIV Tat-specific factor 1 OS=Homo sapiens GN=HTATSF1 PE=1 SV=1	sp|O43719|HTSF1_HUMAN	2.9	1	0.8551	0.842

2770	Serine/threonine-protein phosphatase 2A 56 kDa regulatory subunit delta isoform OS=Homo sapiens GN=PPP2R5D PE=1 SV=1	sp|Q14738|2A5D_HUMAN	7.3	2	1.0093	0.972
2771	Caspase-7 OS=Homo sapiens GN=CASP7 PE=1 SV=1	sp|P55210|CASP7_HUMAN	15.8	2	0.8241	0.7173
2772	Golgin subfamily A member 4 OS=Homo sapiens GN=GOLGA4 PE=1 SV=1	sp|Q13439|GOGA4_HUMAN	4.6	3	0.8166	0.7109
2773	Pleckstrin homology domain-containing family F member 2 OS=Homo sapiens GN=PLEKHF2	sp|Q9H8W4|PKHF2_HUMAN	4.8	1	1	0.9952
2774	REVERSED Matrix-remodeling-associated protein 5 OS=Homo sapiens GN=MXRA5 PE=2 SV=3	RRRRRsp|Q9NR99|MXRA5_HUMAN	2.2	3	1.4588	0.485
2775	REVERSED Pecanex-like protein 2 OS=Homo sapiens GN=PCNXL2	RRRRRsp|A6NKB5|PCX2_HUMAN	1.8	1	1.0666	0.8237
2776	Protein DDI1 homolog 1 OS=Homo sapiens GN=DDI1 PE=1 SV=1	sp|Q8WTU0|DDI1_HUMAN	5.8	2	1.1066	0.8251
2777	DNA polymerase alpha catalytic subunit OS=Homo sapiens GN=POLA1 PE=1	sp|P09884|DPOLA_HUMAN	2.2	1	0.7447	0.5864
2778	60S ribosomal protein L37 OS=Homo sapiens GN=RPL37 PE=1 SV=2	sp|P61927|RL37_HUMAN	13.4	1	1.3677	0.5577
2779	Forkhead box protein P1 OS=Homo sapiens GN=FOXP1 PE=1	sp|Q9H334|FOXP1_HUMAN	3.1	1	1.0375	0.9208
2780	REVERSED H(+)/Cl(-) exchange transporter 4 OS=Homo sapiens GN=CLCN4 PE=1	RRRRRsp|P51793|CLCN4_HUMAN	3.6	1	0.8872	0.8228
2781	REVERSED Apoptosis inhibitor 5 OS=Homo sapiens GN=API5 PE=1 SV=3	RRRRRsp|Q9BZZ5|API5_HUMAN	2.1	0	0.7178	0.5589
2782	CDKN2A-interacting protein OS=Homo sapiens GN=CDKN2AIP PE=1 SV=3	sp|Q9NXV6|CARF_HUMAN	4.7	1	1.0471	0.9108
2783	REVERSED MAM and LDL-receptor class A domain-containing protein 1 OS=Homo sapiens GN=MALRD1	RRRRRsp|Q5VYJ5|MALR1_HUMAN	1.6	1	1.028	0.9275
2784	Histone deacetylase complex subunit SAP130 OS=Homo sapiens GN=SAP130 PE=1 SV=1	sp|Q9H0E3|SP130_HUMAN	1.8	1	0.7516	0.5754

2785	Nucleus accumbens-associated protein 1 OS=Homo sapiens GN=NACC1 PE=1 SV=1	sp|Q96RE7|NACC1_HUMAN	3.6	1	0.7047	0.5464
2786	Polycomb group RING finger protein 2 OS=Homo sapiens GN=PCGF2 PE=1	sp|P35227|PCGF2_HUMAN	2.3	1	0.9727	0.9337
2787	Disks large-associated protein 4 OS=Homo sapiens GN=DLGAP4 PE=1 SV=3	sp|Q9Y2H0|DLGP4_HUMAN	3.3	1		
2788	REVERSED Neuroblast differentiation-associated protein AHNAK OS=Homo sapiens GN=AHNAK PE=1 SV=2	RRRRRsp|Q09666|AHNK_HUMAN	1.8	1	1.1272	0.7957
2789	GDP-Man:Man(3)GlcNAc(2)-PP-Dol alpha-1,2-mannosyltransferase OS=Homo sapiens GN=ALG11 PE=1	sp|Q2TAA5|ALG11_HUMAN	6.3	1	1.0375	0.9255
2790	Spindle assembly abnormal protein 6 homolog OS=Homo sapiens GN=SASS6 PE=1	sp|Q6UVJ0|SAS6_HUMAN	2.4	1	0.6792	0.5127
2791	Protein dpy-30 homolog OS=Homo sapiens GN=DPY30 PE=1	sp|Q9C005|DPY30_HUMAN	20.2	1	0.5598	0.5167
2792	Selenocysteine-specific elongation factor OS=Homo sapiens GN=EEFSEC PE=1 SV=4	sp|P57772|SELB_HUMAN	7.9	0	1.0568	0.8951
2793	Golgin subfamily A member 1 OS=Homo sapiens GN=GOLGA1 PE=1 SV=3	sp|Q92805|GOGA1_HUMAN	4.2	0	0.879	0.7922
2794	Dedicator of cytokinesis protein 6 OS=Homo sapiens GN=DOCK6 PE=1	sp|Q96HP0|DOCK6_HUMAN	4.4	1	1.6596	0.4109
2795	Sortilin OS=Homo sapiens GN=SORT1 PE=1	sp|Q99523|SORT_HUMAN	1.7	1	1.2359	0.6743
2796	Echinoderm microtubule-associated protein-like 5 OS=Homo sapiens GN=EML5 PE=2 SV=3	sp|Q05BV3|EMAL5_HUMAN	2.4	1	1.1376	0.7883
2797	Cilia- and flagella-associated protein 58 OS=Homo sapiens GN=CFAP58 PE=1	sp|Q5T655|CFA58_HUMAN	3.1	1	1.0186	0.9561
2798	SRSF protein kinase 1 OS=Homo sapiens GN=SRPK1 PE=1	sp|Q96SB4|SRPK1_HUMAN	8.6	1	0.863	0.7855
2799	LDLR chaperone MESD OS=Homo sapiens GN=MESDC2 PE=1 SV=2	sp|Q14696|MESD_HUMAN	12.8	0	0.9727	0.9691

2800	Serine/threonine-protein kinase N2 OS=Homo sapiens GN=PKN2 PE=1 SV=1	sp|Q16513|PKN2_HUMAN	5.9	1	0.929	0.8938
2801	Nuclear factor NF-kappa-B p100 subunit OS=Homo sapiens GN=NFKB2 PE=1	sp|Q00653|NFKB2_HUMAN	7.4	1	1.4997	0.4821
2802	Sentrin-specific protease 1 OS=Homo sapiens GN=SENP1 PE=1 SV=2	sp|Q9P0U3|SENP1_HUMAN	2.8	1	1.028	0.9377
2803	Tropomodulin-2 OS=Homo sapiens GN=TMOD2 PE=1 SV=1	sp|Q9NZR1|TMOD2_HUMAN	5.4	2	0.912	0.8776
2804	WD40 repeat-containing protein SMU1 OS=Homo sapiens GN=SMU1 PE=1 SV=2	sp|Q2TAY7|SMU1_HUMAN	8.6	2	0.8472	0.7496
2805	MICOS complex subunit MIC26 OS=Homo sapiens GN=APOO PE=1 SV=1	sp|Q9BUR5|MIC26_HUMAN	8.1	0	1.0864	0.8465
2806	Protein HEXIM1 OS=Homo sapiens GN=HEXIM1 PE=1 SV=1	sp|O94992|HEXI1_HUMAN	3.6	0	0.7798	0.6573
2807	Neurogenic locus notch homolog protein 4 OS=Homo sapiens GN=NOTCH4 PE=1 SV=2	sp|Q99466|NOTC4_HUMAN	3	1	0.929	0.8777
2808	Plakophilin-4 OS=Homo sapiens GN=PKP4 PE=1 SV=2	sp|Q99569|PKP4_HUMAN	4.1	1	1.0965	0.7509
2809	WD repeat-containing protein 7 OS=Homo sapiens GN=WDR7 PE=1	sp|Q9Y4E6|WDR7_HUMAN	3.1	1	1	0.9865
2810	Peroxisome assembly factor 2 OS=Homo sapiens GN=PEX6 PE=1 SV=2	sp|Q13608|PEX6_HUMAN	8	1	1.0093	0.9832
2811	Pre-mRNA-splicing factor ISY1 homolog OS=Homo sapiens GN=ISY1 PE=1	sp|Q9ULR0|ISY1_HUMAN	2.8	1	0.6486	0.689
2812	Putative uncharacterized protein LOC100128429 OS=Homo sapiens PE=5 SV=1	sp|Q6ZWC4|YS043_HUMAN	5.1	0	1.4191	0.645
2813	Mediator of RNA polymerase II transcription subunit 15 OS=Homo sapiens GN=MED15 PE=1	sp|Q96RN5|MED15_HUMAN	4.9	0	0.8017	0.022
2814	REVERSED E3 ubiquitin-protein ligase PDZRN3 OS=Homo sapiens GN=PDZRN3 PE=1 SV=2	RRRRRsp|Q9UPQ7|PZRN3_HUMAN	3.5	1		
2815	Beta-2-syntrophin OS=Homo sapiens GN=SNTB2 PE=1 SV=1	sp|Q13425|SNTB2_HUMAN	5	0	0.9638	0.815

2816	Copper homeostasis protein cutC homolog OS=Homo sapiens GN=CUTC PE=1	sp|Q9NTM9|CUTC_HUMAN	6.2	0	1.3552	0.5747
2817	Serine/threonine-protein kinase PAK 3 OS=Homo sapiens GN=PAK3 PE=1 SV=2	sp|O75914|PAK3_HUMAN	14.3	5	0.7311	0.5826
2818	Chromodomain-helicase-DNA-binding protein 8 OS=Homo sapiens GN=CHD8 PE=1 SV=5	sp|Q9HCK8|CHD8_HUMAN	2.7	1	0.9727	0.9736
2819	Sterol regulatory element-binding protein 1 OS=Homo sapiens GN=SREBF1 PE=1 SV=2	sp|P36956|SRBP1_HUMAN	3.5	0	0.9727	0.9805
2820	Gem-associated protein 2 OS=Homo sapiens GN=GEMIN2 PE=1 SV=1	sp|O14893|GEMI2_HUMAN	9.6	1	0.7943	0.5996
2821	REVERSED Cullin-1 OS=Homo sapiens GN=CUL1 PE=1 SV=2	RRRRRsp|Q13616|CUL1_HUMAN	1.8	0	0.7516	0.5734
2822	Myoferlin OS=Homo sapiens GN=MYOF PE=1 SV=1	sp|Q9NZM1|MYOF_HUMAN	3	1	1.2246	0.7073
2823	tRNA modification GTPase GTPBP3, mitochondrial OS=Homo sapiens GN=GTPBP3 PE=1 SV=2	sp|Q969Y2|GTPB3_HUMAN	5.7	1	0.929	0.8984
2824	Programmed cell death protein 7 OS=Homo sapiens GN=PDCD7 PE=1 SV=1	sp|Q8N8D1|PDCD7_HUMAN	4.9	0	0.8241	0.6481
2825	REVERSED Centrosomal protein of 104 kDa OS=Homo sapiens GN=CEP104 PE=1 SV=1	RRRRRsp|O60308|CE104_HUMAN	2.1	0	0.8091	0.6887
2826	Ubiquitin carboxyl-terminal hydrolase 16 OS=Homo sapiens GN=USP16 PE=1	sp|Q9Y5T5|UBP16_HUMAN	2.1	1	0.871	0.795
2827	Protein prune homolog 2 OS=Homo sapiens GN=PRUNE2 PE=1 SV=3	sp|Q8WUY3|PRUN2_HUMAN	1.9	0	0.9204	0.876
2828	85/88 kDa calcium-independent phospholipase A2 OS=Homo sapiens GN=PLA2G6 PE=1 SV=2	sp|O60733|PLPL9_HUMAN	3.2	1	1.1066	0.8317
2829	Bicaudal D-related protein 2 OS=Homo sapiens GN=CCDC64B PE=1 SV=2	sp|A1A5D9|BICR2_HUMAN	6.7	1	0.955	0.9459
2830	LIM and SH3 domain protein 1 OS=Homo sapiens GN=LASP1 PE=1 SV=2	sp|Q14847|LASP1_HUMAN	7.7	0	1.0965	0.8389

2831	1-aminocyclopropane-1-carboxylate synthase-like protein 1 OS=Homo sapiens GN=ACCS PE=1	sp|Q96QU6|1A1L1_HUMAN	2	0	0.8091	0.6913
2832	N6-adenosine-methyltransferase subunit METTL14 OS=Homo sapiens GN=METTL14 PE=1 SV=2	sp|Q9HCE5|MET14_HUMAN	4.4	0	0.912	0.7167
2833	Squalene monooxygenase OS=Homo sapiens GN=SQLE PE=1 SV=3	sp|Q14534|ERG1_HUMAN	2.4	1	1.028	0.9354
2834	Lysosomal alpha-glucosidase OS=Homo sapiens GN=GAA PE=1 SV=4	sp|P10253|LYAG_HUMAN	5.4	0	2.9376	0.4154
2835	Zinc fingers and homeoboxes protein 3 OS=Homo sapiens GN=ZHX3 PE=1	sp|Q9H4I2|ZHX3_HUMAN	2.1	0	0.9204	0.881
2836	REVERSED Dedicator of cytokinesis protein 1 OS=Homo sapiens GN=DOCK1 PE=1	RRRRRsp|Q14185|DOCK1_HUMAN	3.6	0	0.8954	0.8403
2837	Histone-lysine N-methyltransferase EHMT1 OS=Homo sapiens GN=EHMT1 PE=1 SV=4	sp|Q9H9B1|EHMT1_HUMAN	3.4	0	1.0093	0.978
2838	Focadhesin OS=Homo sapiens GN=FOCAD PE=1 SV=1	sp|Q5VW36|FOCAD_HUMAN	3.1	0	0.8872	0.819
2839	Immunoglobulin superfamily member 8 OS=Homo sapiens GN=IGSF8 PE=1 SV=1	sp|Q969P0|IGSF8_HUMAN	7.5	0	1	0.9766
2840	ADP-ribosylation factor-like protein 6 OS=Homo sapiens GN=ARL6 PE=1 SV=1	sp|Q9H0F7|ARL6_HUMAN	4.8	0	0.8954	0.839
2841	COMM domain-containing protein 6 OS=Homo sapiens GN=COMMD6	sp|Q7Z4G1|COMD6_HUMAN	10.6	0	1.3062	0.6103
2842	Cytoplasmic FMR1-interacting protein 2 OS=Homo sapiens GN=CYFIP2 PE=1	sp|Q96F07|CYFP2_HUMAN	10.4	4	1.0864	0.8553
2843	REVERSED DNA-dependent protein kinase catalytic subunit OS=Homo sapiens GN=PRKDC PE=1 SV=3	RRRRRsp|P78527|PRKDC_HUMAN	3.3	1	0.9462	0.8909
2844	Zinc finger protein 787 OS=Homo sapiens GN=ZNF787 PE=1 SV=3	sp|Q6DD87|ZN787_HUMAN	7.8	0	1.0093	0.9681
2845	Nuclear receptor subfamily 1 group I member 3 OS=Homo sapiens GN=NR1I3 PE=1 SV=2	sp|Q14994|NR1I3_HUMAN	7.4	0	0.8872	0.8186

2846	REVERSED Palmitoyltransferase ZDHHC2 OS=Homo sapiens GN=ZDHHC2 PE=2 SV=1	RRRRRsp|Q9UIJ5|ZDHC2_HUMAN	4.9	1	0.879	0.8092
2847	Ubiquitin-conjugating enzyme E2 B OS=Homo sapiens GN=UBE2B PE=1	sp|P63146|UBE2B_HUMAN	7.9	0	1.0186	0.9679
2848	Cysteine protease ATG4B OS=Homo sapiens GN=ATG4B PE=1	sp|Q9Y4P1|ATG4B_HUMAN	9.7	2	0.879	0.7918
2849	ADP-ribosylation factor-related protein 1 OS=Homo sapiens GN=ARFRP1 PE=1 SV=1	sp|Q13795|ARFRP_HUMAN	8.5	1	1.1169	0.8164
2850	Tubulin-specific chaperone D OS=Homo sapiens GN=TBCD PE=1 SV=2	sp|Q9BTW9|TBCD_HUMAN	3.4	1	0.9908	0.9984
2851	REVERSED Mitogen-activated protein kinase kinase kinase 1 OS=Homo sapiens GN=MAP3K1 PE=1 SV=4	RRRRRsp|Q13233|M3K1_HUMAN	3.6	0	1.1912	0.7216
2852	ERO1-like protein beta OS=Homo sapiens GN=ERO1LB PE=1 SV=2	sp|Q86YB8|ERO1B_HUMAN	7.9	0	0.9817	0.9793
2853	Nuclease-sensitive element-binding protein 1 OS=Homo sapiens GN=YBX1 PE=1	sp|P67809|YBOX1_HUMAN	6.2	0	0.4487	0.523
2854	Integrin alpha-6 OS=Homo sapiens GN=ITGA6 PE=1 SV=5	sp|P23229|ITA6_HUMAN	4.1	1	0.9036	0.8544
2855	REVERSED Synaptonemal complex central element protein 1 OS=Homo sapiens GN=SYCE1 PE=1 SV=2	RRRRRsp|Q8N0S2|SYCE1_HUMAN	15.4	1	0.8872	0.7065
2856	NADH dehydrogenase [ubiquinone] 1 alpha subcomplex assembly factor 3 OS=Homo sapiens GN=NDUFAF3 PE=1 SV=1	sp|Q9BU61|NDUF3_HUMAN	10.9	1	1.1376	0.7778
2857	REVERSED U5 small nuclear ribonucleoprotein 200 kDa helicase OS=Homo sapiens GN=SNRNP200 PE=1 SV=2	RRRRRsp|O75643|U520_HUMAN	2.8	2		
2858	REVERSED Lamin-B2 OS=Homo sapiens GN=LMNB2 PE=1 SV=3	RRRRRsp|Q03252|LMNB2_HUMAN	8	0	0.6792	0.6668
2859	REVERSED Glia maturation factor beta OS=Homo sapiens GN=GMFB PE=1 SV=2	RRRRRsp|P60983|GMFB_HUMAN	4.2	0	0.7447	0.6017

2860	REVERSED Mitogen-activated protein kinase kinase kinase MLK4 OS=Homo sapiens GN=MLK4 PE=1 SV=1	RRRRRsp|Q5TCX8|M3KL4_HUMAN	2.8	0	1.1066	0.8284
2861	REVERSED BEN domain-containing protein 2 OS=Homo sapiens GN=BEND2 PE=2	RRRRRsp|Q8NDZ0|BEND2_HUMAN	2.5	0	0.9817	0.9838
2862	Immunoglobulin lambda-like polypeptide 5 OS=Homo sapiens GN=IGLL5 PE=2 SV=2	sp|B9A064|IGLL5_HUMAN	7.9	1		
2863	REVERSED Transcription elongation factor A protein 1 OS=Homo sapiens GN=TCEA1 PE=1	RRRRRsp|P23193|TCEA1_HUMAN	6	0	0.9817	0.9404
2864	Cytochrome c-type heme lyase OS=Homo sapiens GN=HCCS PE=1 SV=1	sp|P53701|CCHL_HUMAN	3.4	0	1.1376	0.7843
2865	GDP-D-glucose phosphorylase 1 OS=Homo sapiens GN=GDPGP1 PE=1 SV=2	sp|Q6ZNW5|GDPP1_HUMAN	8.3	2		
2866	REVERSED Transcription elongation factor A protein-like 7 OS=Homo sapiens GN=TCEAL7 PE=2 SV=2	RRRRRsp|Q9BRU2|TCAL7_HUMAN	15	1		
2867	55 kDa erythrocyte membrane protein OS=Homo sapiens GN=MPP1 PE=1 SV=2	sp|Q00013|EM55_HUMAN	5.2	1		
2868	Coronin-1B OS=Homo sapiens GN=CORO1B PE=1 SV=1	sp|Q9BR76|COR1B_HUMAN	5.3	1	1.3183	0.6058
2869	Intersectin-1 OS=Homo sapiens GN=ITSN1 PE=1 SV=3	sp|Q15811|ITSN1_HUMAN	2.9	1	1.0375	0.8984
2870	G-protein coupled receptor 6 OS=Homo sapiens GN=GPR6 PE=1 SV=1	sp|P46095|GPR6_HUMAN	6.4	0	0.4786	0.7473
2871	REVERSED tRNA (guanine(37)-N1)-methyltransferase OS=Homo sapiens GN=TRMT5 PE=1 SV=2	RRRRRsp|Q32P41|TRM5_HUMAN	3.5	0	0.9817	0.988
2872	TBC1 domain family member 23 OS=Homo sapiens GN=TBC1D23 PE=1 SV=3	sp|Q9NUY8|TBC23_HUMAN	1.7	0	0.9462	0.9269
2873	Aldo-keto reductase family 1 member B10 OS=Homo sapiens GN=AKR1B10 PE=1 SV=2	sp|O60218|AK1BA_HUMAN	19	2	1.4454	0.5119
2874	Protein Jade-2 OS=Homo sapiens GN=JADE2 PE=1 SV=2	sp|Q9NQC1|JADE2_HUMAN	5.6	2		

2875	Lysine-specific demethylase 3A OS=Homo sapiens GN=KDM3A PE=1 SV=4	sp|Q9Y4C1|KDM3A_HUMAN	1.8	1	0.6607	0.492
2876	REVERSED Replication factor C subunit 5 OS=Homo sapiens GN=RFC5 PE=1	RRRRRsp|P40937|RFC5_HUMAN	4.7	1		
2877	Inositol monophosphatase 3 OS=Homo sapiens GN=IMPAD1 PE=1 SV=1	sp|Q9NX62|IMPA3_HUMAN	5	1		
2878	Ubiquitin thioesterase otulin OS=Homo sapiens GN=OTULIN PE=1 SV=3	sp|Q96BN8|OTUL_HUMAN	10.5	2		
2879	REVERSED Protein kinase C zeta type OS=Homo sapiens GN=PRKCZ PE=1	RRRRRsp|Q05513|KPCZ_HUMAN	2.9	1	1.1066	0.8622
2880	MAP kinase-activating death domain protein OS=Homo sapiens GN=MADD PE=1 SV=2	sp|Q8WXG6|MADD_HUMAN	3.5	1	1.028	0.9434
2881	REVERSED Sodium channel protein type 11 subunit alpha OS=Homo sapiens GN=SCN11A	RRRRRsp|Q9UI33|SCNBA_HUMAN	2.2	0	1.1169	0.8013
2882	REVERSED Serine/threonine-protein kinase WNK1 OS=Homo sapiens GN=WNK1 PE=1	RRRRRsp|Q9H4A3|WNK1_HUMAN	2.1	0	0.912	0.8667
2883	ER membrane protein complex subunit 4 OS=Homo sapiens GN=EMC4 PE=1	sp|Q5J8M3|EMC4_HUMAN	5.5	0	1.3305	0.5865
2884	Tumor necrosis factor ligand superfamily member 18 OS=Homo sapiens GN=TNFSF18 PE=1 SV=2	sp|Q9UNG2|TNF18_HUMAN	7.5	1		
2885	ESF1 homolog OS=Homo sapiens GN=ESF1 PE=1 SV=1	sp|Q9H501|ESF1_HUMAN	2.2	0	0.8954	0.3226
2886	Signal recognition particle subunit SRP72 OS=Homo sapiens GN=SRP72 PE=1 SV=3	sp|O76094|SRP72_HUMAN	1.6	0	0.912	0.8729
2887	REVERSED Transcription factor EC OS=Homo sapiens GN=TFEC PE=1 SV=1	RRRRRsp|O14948|TFEC_HUMAN	3.2	0	0.0203	0.0312
2888	Little elongation complex subunit 1 OS=Homo sapiens GN=ICE1 PE=1 SV=5	sp|Q9Y2F5|ICE1_HUMAN	1.7	2	0.955	0.8288
2889	pre-mRNA 3' end processing protein WDR33 OS=Homo sapiens GN=WDR33 PE=1 SV=2	sp|Q9C0J8|WDR33_HUMAN	2.6	1	0.9376	0.9583

2890	Ribosome production factor 1 OS=Homo sapiens GN=RPF1 PE=1 SV=2	sp|Q9H9Y2|RPF1_HUMAN	13.5	1		
2891	REVERSED Inositol-3-phosphate synthase 1 OS=Homo sapiens GN=ISYNA1 PE=1	RRRRRsp|Q9NPH2|INO1_HUMAN	3.2	0		
2892	Bromodomain and PHD finger-containing protein 3 OS=Homo sapiens GN=BRPF3 PE=1	sp|Q9ULD4|BRPF3_HUMAN	3.7	2	1.0186	0.9486
2893	REVERSED E3 ubiquitin-protein ligase RNF43 OS=Homo sapiens GN=RNF43 PE=1 SV=1	RRRRRsp|Q68DV7|RNF43_HUMAN	5	0	0.7447	0.6054
2894	Lysine-specific demethylase 6B OS=Homo sapiens GN=KDM6B PE=1 SV=4	sp|O15054|KDM6B_HUMAN	2.7	0	0.912	0.867
2895	REVERSED NACHT, LRR and PYD domains-containing protein 10 OS=Homo sapiens GN=NLRP10 PE=1	RRRRRsp|Q86W26|NAL10_HUMAN	4.1	0	1.0864	0.8587
2896	REVERSED Ribosomal RNA processing protein 1 homolog A OS=Homo sapiens GN=RRP1 PE=1 SV=1	RRRRRsp|P56182|RRP1_HUMAN	8.7	0	0.6368	0.5667
2897	Collagen alpha-2(XI) chain OS=Homo sapiens GN=COL11A2 PE=1 SV=5	sp|P13942|COBA2_HUMAN	2.7	0	0.8091	0.698
2898	NLR family member X1 OS=Homo sapiens GN=NLRX1 PE=1 SV=1	sp|Q86UT6|NLRX1_HUMAN	4.1	0	1.2246	0.6859
2899	WD repeat-containing protein 34 OS=Homo sapiens GN=WDR34 PE=1	sp|Q96EX3|WDR34_HUMAN	6.3	0	1.2134	0.2557
2900	REVERSED Cadherin-17 OS=Homo sapiens GN=CDH17 PE=2 SV=3	RRRRRsp|Q12864|CAD17_HUMAN	5.2	0	0.3048	0.3031
2901	Sulfhydryl oxidase 2 OS=Homo sapiens GN=QSOX2 PE=1	sp|Q6ZRP7|QSOX2_HUMAN	6.4	0	1.2359	0.6748
2902	REVERSED Nitrogen permease regulator 3-like protein OS=Homo sapiens GN=NPRL3 PE=1	RRRRRsp|Q12980|NPRL3_HUMAN	5.6	0	0.955	0.9352
2903	Exonuclease 1 OS=Homo sapiens GN=EXO1 PE=1 SV=2	sp|Q9UQ84|EXO1_HUMAN	2.7	0	1.2246	0.6899
2904	Catenin alpha-1 OS=Homo sapiens GN=CTNNA1 PE=1 SV=1	sp|P35221|CTNA1_HUMAN	2.3	0	1.0568	0.898

2905	Polycomb complex protein BMI-1 OS=Homo sapiens GN=BMI1 PE=1 SV=2	sp|P35226|BMI1_HUMAN	2.5	0	0.6855	0.5142
2906	High affinity immunoglobulin epsilon receptor subunit gamma OS=Homo sapiens GN=FCER1G PE=1 SV=1	sp|P30273|FCERG_HUMAN	11.6	0	1.4588	0.7331
2907	Tumor necrosis factor alpha-induced protein 8 OS=Homo sapiens GN=TNFAIP8 PE=1 SV=1	sp|O95379|TFIP8_HUMAN	5.6	0	1.9231	0.6305
2908	Eukaryotic translation elongation factor 1 epsilon-1 OS=Homo sapiens GN=EEF1E1 PE=1 SV=1	sp|O43324|MCA3_HUMAN	8	1		
2909	Cytochrome b-c1 complex subunit 9 OS=Homo sapiens GN=UQCR10 PE=1 SV=3	sp|Q9UDW1|QCR9_HUMAN	27	0	0.0107	0.021
2910	Snurportin-1 OS=Homo sapiens GN=SNUPN PE=1 SV=1	sp|O95149|SPN1_HUMAN	2.8	0	0.8954	0.8306
2911	Acetyl-coenzyme A transporter 1 OS=Homo sapiens GN=SLC33A1 PE=1 SV=1	sp|O00400|ACATN_HUMAN	1.6	0	0.7798	0.7125
2912	SLAIN motif-containing protein 2 OS=Homo sapiens GN=SLAIN2 PE=1	sp|Q9P270|SLAI2_HUMAN	9	0	0.8472	0.7442
2913	REVERSED E3 ubiquitin-protein ligase RNF25 OS=Homo sapiens GN=RNF25 PE=1 SV=1	RRRRRsp|Q96BH1|RNF25_HUMAN	4.6	0	1.1912	0.6584
2914	Cyclin-Y OS=Homo sapiens GN=CCNY PE=1	sp|Q8ND76|CCNY_HUMAN	3.2	0	1.2246	0.6837

Table S3 Differentially expressed proteins of normal and VCP2-treated HepG2 cells identified by 2D-LC-MSMS

Number	Protein name	Accession	Gene	Coverage (%)	Peptides (95%)	Expression
8	Heat shock cognate 71 kDa	P11142	HSP7C	73.7	174	downregulation
9	Actin, cytoplasmic 2	P63261	ACTG	78.7	236	upregulation
14	Heat shock protein HSP 90-	P08238	HS90B	50	72	downregulation
15	Plastin-2	P13796	PLSL	59	69	upregulation
21	Alpha-enolase	P06733	ENOA	71.7	103	upregulation
24	60 kDa heat shock protein,	P10809	CH60	59.5	111	upregulation
47	Histone H2B type 1-N	Q99877	H2B1N	78.6	41	downregulation
55	60S ribosomal protein L3	P39023	RL3	42.9	72	upregulation
61	Vimentin	P08670	VIME	43.8	22	upregulation
64	T-complex protein 1 subunit	Q99832	TCPH	43.1	37	upregulation
82	Polyadenylate-binding protein	P11940	PABP1	39.6	24	downregulation
89	Serine--tRNA ligase,	P49591	SYSC	39.5	17	upregulation
113	Aspartyl/asparaginyl beta-	Q12797	ASPH	23.8	15	upregulation
118	Keratin, type II cytoskeletal 1	P04264	K2C1	33.1	12	downregulation
120	Tyrosine--tRNA ligase,	P54577	SYYC	34.5	14	upregulation
141	V-type proton ATPase subunit	P21281	VATB2	40.5	26	upregulation
144	DNA replication licensing factor	P25205	MCM3	22.8	14	downregulation
161	Keratin, type I cytoskeletal 9	P35527	K1C9	27	24	downregulation
166	60S ribosomal protein L4	P36578	RL4	39.1	24	upregulation
169	Vesicle-fusing ATPase	P46459	NSF	18.2	8	upregulation
182	GMP synthase [glutamine-	P49915	GUAA	24.7	10	downregulation
192	Monofunctional C1-	Q6UB35	C1TM	16.2	12	upregulation
207	Creatine kinase B-type	P12277	KCRB	39.6	18	downregulation
208	Asparagine--tRNA ligase,	O43776	SYNC	27.9	11	upregulation
230	DNA replication licensing factor	P49736	MCM2	21.4	12	downregulation
243	Heat shock 70 kDa protein 4L	O95757	HS74L	30	21	upregulation
244	Structural maintenance of	O95347	SMC2	16.5	9	downregulation
285	DNA (cytosine-5)-	P26358	DNMT1	12.1	8	downregulation
313	Mitochondrial 10-	Q3SY69	AL1L2	17.4	12	upregulation
323	Histone H4	P62805	H4	69.9	25	downregulation
329	Importin subunit alpha-1	P52292	IMA1	25.3	7	downregulation
347	Voltage-dependent	Q9Y277	VDAC3	31.5	9	upregulation
365	14-3-3 protein gamma	P61981	1433G	51	20	upregulation
382	Annexin A5	P08758	ANXA5	35	12	upregulation
388	Serrate RNA effector	Q9BXP5	SRRT	15.3	7	downregulation

451	Gelsolin	P06396	GELS	18.2	7	upregulation
456	Cytosolic non-specific	Q96KP4	CNDP2	16	7	upregulation
462	Condensin complex subunit	Q9BPX3	CND3	11.3	6	downregulation
471	Chromobox protein homolog	Q13185	CBX3	49.2	7	downregulation
483	General transcription	P29083	T2EA	21.4	5	downregulation
488	Aldose reductase	P15121	ALDR	31	12	upregulation
493	Polyadenylate-binding protein	Q13310	PABP4	28.9	11	downregulation
505	DNA ligase 1	P18858	DNLI1	12.2	7	downregulation
506	Tricarboxylate transport	P53007	TXTP	25.4	5	upregulation
507	Ras GTPase-activating	Q13283	G3BP1	19.7	6	downregulation
547	ERO1-like protein alpha	Q96HE7	ERO1A	21.4	6	upregulation
567	Serine/threonine -protein	O15084	ANR28	9.1	4	upregulation
577	Peroxiredoxin-5, mitochondrial	P30044	PRDX5	40.2	14	upregulation
591	Tyrosine-protein kinase BAZ1B	Q9UIG0	BAZ1B	9.7	13	downregulation
594	S-formylglutathion	P10768	ESTD	40.8	5	upregulation
609	Inositol-3-phosphate	Q9NPH2	INO1	20.3	8	downregulation
610	Fructose-bisphosphate	P09972	ALDOC	45.1	29	upregulation
612	Proteasome activator	P61289	PSME3	32.7	5	downregulation
670	Ribonucleoside-diphosphate	P31350	RIR2	16.2	4	downregulation
708	Eukaryotic translation	Q15056	IF4H	35.5	4	downregulation
722	Acetyl-CoA acetyltransferase	Q9BWD1	THIC	36.8	10	upregulation
730	Ragulator complex protein	Q9Y2Q5	LTOR2	35.2	5	upregulation
769	Mini-chromosome	Q9BTE3	MCMBP	10.9	6	downregulation
787	60S ribosomal protein L27	P61353	RL27	33.1	8	upregulation
806	Sequestosome-1	Q13501	SQSTM	32.5	24	upregulation
839	Ubiquitin-like modifier-	O95352	ATG7	7.3	3	upregulation
853	Proliferating cell nuclear antigen	P12004	PCNA	24.9	6	downregulation
886	Transmembrane 9 superfamily	Q92544	TM9S4	10.1	3	upregulation
892	Mitogen-activated protein	Q16539	MK14	24.7	3	downregulation
900	Histone H3.1	P68431	H31	77.9	25	downregulation
956	Transcription factor BTF3	P20290	BTF3	49	6	downregulation
960	Transferrin receptor protein	P02786	TFR1	6.8	4	downregulation
996	Rho-related GTP-binding	P84095	RHOG	28.8	5	upregulation
1096	DNA topoisomerase	P11388	TOP2A	6.9	6	downregulation
1101	SNW domain-containing	Q13573	SNW1	16	8	downregulation
1113	Signal transducer and	P40763	STAT3	11	3	downregulation
1144	Fibronectin type III domain-	Q53EP0	FND3B	5.8	3	upregulation
1155	Tubulin gamma-1 chain	P23258	TBG1	23.1	3	downregulation

1163	EF-hand domain-	Q96C19	EFHD2	23.8	4	upregulation
1172	Neurochondrin	Q9UBB6	NCDN	9.9	3	downregulation
1292	Solute carrier family 2,	P11166	GTR1	11.8	4	upregulation
1296	Thioredoxin-interacting	Q9H3M7	TXNIP	14.8	2	upregulation
1306	Synaptic vesicle membrane	Q99536	VAT1	19.6	3	upregulation
1326	Importin-8	O15397	IPO8	5.2	3	downregulation
1379	Pleckstrin	P08567	PLEK	16	3	upregulation
1422	Nuclear distribution	Q9NXR1	NDE1	14.7	2	downregulation
1449	Protein S100-A11	P31949	S10AB	33.3	3	upregulation
1460	Protein FAM192A	Q9GZU8	F192A	12.2	2	downregulation
1512	Hemoglobin subunit alpha	P69905	HBA	16.9	2	upregulation
1536	Protein NDRG1	Q92597	NDRG1	8.1	4	upregulation
1616	Protein Red	Q13123	RED	7.4	2	downregulation
1655	Vitronectin	P04004	VTNC	8.6	2	upregulation
1656	Histone-binding protein RBBP7	Q16576	RBBP7	10.4	2	downregulation
1713	Rho GDP-dissociation	P52565	GDIR1	13.7	5	upregulation
1737	Putative phospholipase	Q8NHP8	PLBL2	6.3	3	upregulation
1753	Actin, cytoplasmic 1	P60709	ACTB	78.7	259	upregulation
1813	Aspartyl aminopeptidase	Q9ULA0	DNPEP	15.2	3	upregulation
1932	Histone H2A type 2-A	Q6FI13	H2A2A	82.3	57	downregulation
1933	Histone H3.3	P84243	H33	68.4	17	downregulation
1936	Myosin-10	P35580	MYH10	10.1	5	upregulation
1956	Dual specificity protein kinase	P33981	TTK	4.4	2	downregulation
2027	Son of sevenless homolog 2	Q07890	SOS2	2.7	2	upregulation
2107	Argininosuccinate lyase	P04424	ARLY	9.1	1	upregulation
2183	Serum albumin	P02768	ALBU	11.8	3	downregulation
2221	Melanoma-associated	Q8TD91	MAGC3	4.4	1	downregulation
2234	Macrophage-expressed gene	Q2M385	MPEG1	4.6	1	upregulation
2247	Cell division cycle protein	O75794	CD123	6	1	downregulation
2289	Serine/threonine-protein kinase	P53350	PLK1	6.6	1	downregulation
2293	GTP cyclohydrolase 1	P30793	GCH1	11.2	2	upregulation
2299	Superoxide dismutase [Mn],	P04179	SODM	12.2	1	upregulation
2301	Protein tyrosine phosphatase	O75365	TP4A3	14.5	1	downregulation
2308	Protein HEATR9	A2RTY3	HEAT9	4.4	1	upregulation
2388	Tubulin-specific chaperone A	O75347	TBCA	17.6	1	downregulation
2392	Centrin-3	O15182	CETN3	12	1	downregulation
2460	Interferon-induced	Q01629	IFM2	15.9	1	upregulation
2502	Zinc finger protein 706	Q9Y5V0	ZN706	14.5	1	downregulation

2552	Chromatin assembly factor	Q13112	CAF1B	2	1	downregulation
2562	Myelin proteolipid	P60201	MYPR	4.7	1	upregulation

Table S4 Differentially expressed proteins of normal and VCP2-treated Caco2 cells identified by 2D-LC-MSMS

Number	Protein name	Accession	Gene	Molecular Weight	FC	Expression
17	Fatty acid synthase	P49327	FASN	273 kDa	1.6021	upregulation
93	Hydroxymethylglutaryl-CoA synthase, cytoplasmic	Q01581	HMGCS1	57 kDa	1.8661	upregulation
161	Glucose-6-phosphate 1-dehydrogenase	P11413	G6PD	59 kDa	1.5157	upregulation
234	Splicing factor, proline- and glutamine-rich	P23246	SFPQ	76 kDa	0.6598	downregulation
258	Histone H2B type F-S	P57053	H2BFS	14 kDa	1.8790	upregulation
555	Transaldolase	P37837	TALDO1	38 kDa	1.5263	upregulation
579	Histone H4	P62805	HIST1H4A	11 kDa	2.1735	upregulation
593	Carbonyl reductase [NADPH] 1	P16152	CBR1	30 kDa	1.5801	upregulation
791	Aspartate aminotransferase, cytoplasmic	P17174	GOT1	46 kDa	1.6702	upregulation
796	Squalene synthase	P37268	FDFT1	48 kDa	1.7654	upregulation
840	Carboxymethylenebutenolidase homolog	Q96DG6	CMBL	28 kDa	1.5369	upregulation
903	Surfeit 4	Q5T8U5	SURF4	21 kDa	1.7411	upregulation
909	Ubiquitin-conjugating enzyme E2 N	P61088	UBE2N	17 kDa	1.7654	upregulation
1000	Ubiquitin-like protein ISG15	P05161	ISG15	18 kDa	1.9862	upregulation
1062	Endoplasmic reticulum resident protein 44	Q9BS26	ERP44	47 kDa	1.5052	upregulation
1066	Putative RNA-binding protein Luc7-like 2	Q9Y383	LUC7L2	47 kDa	1.5369	upregulation
1114	Ubiquitin-conjugating enzyme E2 variant 1	Q13404	UBE2V1	16 kDa	1.6133	upregulation
1133	Heterogeneous nuclear ribonucleoprotein H3	P31942	HNRNPH3	37 kDa	0.6071	downregulation
1160	Alpha-2-HS-glycoprotein	P02765	AHSG	39 kDa	0.2932	downregulation
1235	Membrane-associated progesterone receptor component 1	O00264	PGRMC1	22 kDa	1.6472	upregulation
1261	D-dopachrome decarboxylase	P30046	DDT	13 kDa	1.5052	upregulation
1310	Folate receptor alpha	P15328	FOLR1	30 kDa	0.5586	downregulation
1343	Alpha-2-macroglobulin	P01023	A2M	163 kDa	0.4061	downregulation
1434	Protein S100-A10	P60903	S100A10	11 kDa	0.5510	downregulation
1465	Delta(24)-sterol reductase	Q15392	DHCR24	60 kDa	1.5369	upregulation
1585	Non-histone chromosomal protein HMG-17	P05204	HMGN2	9 kDa	0.5322	downregulation
1727	RNA-binding protein 14	Q96PK6	RBM14	69 kDa	0.5359	downregulation
1765	Lanosterol 14-alpha demethylase	Q16850	CYP51A1	57 kDa	1.7532	upregulation
1826	Thymosin beta-10	P63313	TMSB10	5 kDa	0.5471	downregulation
1836	Protein argonaute-2	Q9UKV8	AGO2	97 kDa	0.6643	downregulation
1854	C-terminal-binding protein 2	P56545	CTBP2	49 kDa	1.5052	upregulation
1940	ADP-ribosylation factor-like protein 3	P36405	ARL3	20 kDa	1.5911	upregulation
1958	7-dehydrocholesterol reductase	Q9UBM7	DHCR7	54 kDa	2.1287	upregulation
1972	Histone H3.1	P68431	HIST1H3A	15 kDa	0.3842	downregulation
2072	Keratin, type I cytoskeletal 10	P13645	KRT10	59 kDa	0.5212	downregulation
2095	Cellular retinoic acid-binding protein 2	P29373	CRABP2	16 kDa	1.9319	upregulation
2114	Protein S100-A4	P26447	S100A4	12 kDa	1.6133	upregulation
2146	Vitamin D-binding protein	D6RF35	GC	53 kDa	0.4506	downregulation
2174	RNA-binding motif protein, X chromosome, N-terminally processed	H3BT71	RBMX	32 kDa	1.6021	upregulation

2239	Acetyl-coenzyme A synthetase, cytoplasmic	Q9NR19	ACSS2	79 kDa	1.6586	upregulation
2250	Charged multivesicular body protein 1b	Q7LBR1	CHMP1B	22 kDa	1.9053	upregulation
2251	CDGSH iron-sulfur domain-containing protein 2	Q8N5K1	CISD2	15 kDa	2.2974	upregulation
2276	Glutaredoxin-1	P35754	GLRX	12 kDa	2.3457	upregulation
2376	Cytochrome c oxidase subunit 2	P00403	MT-CO2	26 kDa	1.9588	upregulation
2432	Metallothionein-2	P02795	MT2A	6 kDa	0.5396	downregulation
2498	Lactotransferrin (Fragment)	E7EQB2	LTF	77 kDa	0.5783	downregulation
2641	Cytochrome c oxidase subunit 6C	P09669	COX6C	9 kDa	1.9453	upregulation
2653	InaD-like protein	Q8NI35	INADL	196 kDa	0.5625	downregulation
2655	Dynein heavy chain 17, axonemal	Q9UFH2	DNAH17	512 kDa	2.0705	upregulation
2660	WW domain-binding protein 2	A6NG10	WBP2	26 kDa	1.5263	upregulation
2731	Very-long-chain (3R)-3-hydroxyacyl-CoA dehydratase 3	Q9P035	PTPLAD1	43 kDa	1.6818	upregulation
2801	Tudor domain-containing protein 7	Q8NHU6	TDRD7	124 kDa	0.6643	downregulation
2820	60S ribosomal protein L36	Q9Y3U8	RPL36	12 kDa	1.9862	upregulation
2824	Non-erythrocytic beta-spectrin 4	C9JY79	SPTBN4	289 kDa	1.7171	upregulation
2931	GTP-binding protein Rheb	Q15382	RHEB	20 kDa	1.5801	upregulation
2975	Tetratricopeptide repeat protein 28	A0A087WW06	TTC28	257 kDa	1.6702	upregulation
3060	Inositol monophosphatase 2	O14732	IMPA2	31 kDa	2.0000	upregulation
3063	Adenylate kinase 4, mitochondrial	P27144	AK4	25 kDa	1.5369	upregulation
3096	Patatin-like phospholipase domain-containing protein 2	Q96AD5	PNPLA2	55 kDa	0.6462	downregulation
3168	Gamma-interferon-inducible lysosomal thiol reductase	P13284	IFI30	28 kDa	1.7053	upregulation
3181	E3 ubiquitin-protein ligase RNF31 (Fragment)	H0YKX0	RNF31	102 kDa	0.6462	downregulation
3184	Histone H2B type 1-O	P23527	HIST1H2BO	14 kDa	2.0562	upregulation
3192	Myosin-7B	A7E2Y1	MYH7B	221 kDa	1.6358	upregulation
3263	U6 snRNA-associated Sm-like protein LSm7	Q9UK45	LSM7	12 kDa	0.6598	downregulation
3278	NADH dehydrogenase [ubiquinone] iron-sulfur protein 8, mitochondrial	O00217	NDUFS8	24 kDa	1.5583	upregulation
3361	Protein QIL1	Q5XKP0	QIL1	13 kDa	0.6329	downregulation
3365	E3 ubiquitin-protein ligase	P62877	RBX1	12 kDa	0.6507	downregulation
3381	Collagen alpha-1(IV) chain	P02462	COL4A1	161 kDa	0.6373	downregulation
3393	Transforming acidic coiled-coil-containing protein 2	O95359	TACC2	309 kDa	0.5664	downregulation
3400	EVI5-like protein	Q96CN4	EVI5L	91 kDa	1.5801	upregulation
3413	Acylphosphatase-1	P07311	ACYP1	11 kDa	2.3620	upregulation
3417	Aldo-keto reductase family 1 member C1	Q04828	AKR1C1	37 kDa	1.8404	upregulation
3476	Keratin, type I cytoskeletal 9	P35527	KRT9	62 kDa	0.6598	downregulation
3477	Keratin, type II cytoskeletal 2 epidermal	P35908	KRT2	65 kDa	0.6643	downregulation
3545	Transmembrane protein 109	Q9BVC6	TMEM109	26 kDa	1.5801	upregulation
3552	Thrombospondin-1	P07996	THBS1	129 kDa	0.6285	downregulation
3576	SET and MYND domain-containing protein 4	Q8IYR2	SMYD4	89 kDa	0.5824	downregulation
3592	Charged multivesicular body protein 3	Q9Y3E7	CHMP3	25 kDa	1.8532	upregulation
3593	Merlin	P35240	NF2	70 kDa	0.6462	downregulation
3608	Keratin, type II cuticular Hb5	P78386	KRT85	56 kDa	0.4830	downregulation

3640	Spectrin alpha chain, erythrocytic 1	A0A087WZE4	SPTA1	281 kDa	0.3439	downregulation
3648	Zinc finger protein 570	Q96NI8	ZNF570	62 kDa	1.7411	upregulation
3654	Inter-alpha-trypsin inhibitor heavy chain H2	A0A087WTE1	ITIH2	107 kDa	0.5176	downregulation
3692	Uncharacterized protein C19orf43	Q9BQ61	C19orf43	18 kDa	0.6643	downregulation
3822	Histone H3.3	P84243	H3F3A	15 kDa	1.5911	upregulation
3825	Tax1-binding protein 3	O14907	TAX1BP3	14 kDa	0.5035	downregulation
3841	NADH dehydrogenase [ubiquinone] 1 beta subcomplex subunit 11, mitochondrial	Q9NX14	NDUFB11	17 kDa	78.7932	upregulation
3844	Eukaryotic translation initiation factor 4E	D6RBW1	EIF4E	29 kDa	1.5157	upregulation
3877	Plasminogen	P00747	PLG	91 kDa	0.4353	downregulation
3897	Vitronectin	P04004	VTN	54 kDa	1.5911	upregulation
3908	EH domain-binding protein 1	Q8NDI1	EHBP1	140 kDa	1.7532	upregulation
3918	Chloride intracellular channel protein 3 (Fragment)	Q5SQ17	CLIC3	19 kDa	1.5263	upregulation
3919	Protocadherin Fat 4	Q6V0I7	FAT4	543 kDa	0.5946	downregulation
3928	Acyl-CoA desaturase	O00767	SCD	42 kDa	2.3457	upregulation
3932	Testis-specific serine/threonine-protein kinase 4	Q6SA08	TSSK4	37 kDa	0.4175	downregulation
3936	Coiled-coil domain-containing protein 40	Q4G0X9	CCDC40	130 kDa	1.5911	upregulation
3938	WD repeat- and FYVE domain-containing protein 4	Q6ZS81	WDFY4	354 kDa	0.5987	downregulation
4012	Proteasome assembly chaperone 3	Q9BT73	PSMG3	13 kDa	1.6935	upregulation
4015	Putative RNA-binding protein 3	P98179	RBM3	17 kDa	0.5783	downregulation
4019	Folate transporter 1	P41440	SLC19A1	65 kDa	0.4830	downregulation
4026	TBC1 domain family member 5	Q92609	TBC1D5	89 kDa	0.6417	downregulation
4028	Transmembrane protein 65	Q6PI78	TMEM65	25 kDa	2.3620	upregulation
4058	TBC1 domain family member 10B	Q4KMP7	TBC1D10B	87 kDa	0.6285	downregulation
4089	Transcription termination factor 2	Q9UNY4	TTF2	130 kDa	0.5396	downregulation
4100	Ankyrin repeat domain-containing protein 27	Q96NW4	ANKRD27	117 kDa	0.6507	downregulation
4117	Synaptogyrin-2	O43760	SYNGR2	25 kDa	2.0849	upregulation
4119	Ubiquitin-fold modifier-conjugating enzyme 1	Q9Y3C8	UFC1	19 kDa	1.5157	upregulation
4121	Pigment epithelium-derived factor	P36955	SERPINF1	46 kDa	0.6029	downregulation
4141	Protein FAM134C	Q86VR2	FAM134C	51 kDa	0.6598	downregulation
4152	Coagulation factor V	P12259	F5	252 kDa	0.5987	downregulation
4162	Antithrombin-III	P01008	SERPINC1	53 kDa	0.4569	downregulation
4180	Transgelin (Fragment)	H0YCU9	TAGLN	17 kDa	0.4444	downregulation
4191	Ras GTPase-activating protein nGAP	Q9UJF2	RASAL2	129 kDa	0.5510	downregulation
4195	Transient receptor potential cation channel subfamily M member 6	Q9BX84	TRPM6	232 kDa	0.3763	downregulation
4209	Telomerase protein component 1	G3V2A4	TEP1	208 kDa	0.5905	downregulation
4214	Zinc finger protein 841	Q6ZN19	ZNF841	93 kDa	0.6552	downregulation
4296	Eukaryotic translation initiation factor 4E-binding protein 1	Q13541	EIF4EBP1	13 kDa	0.6598	downregulation
4298	Biogenesis of lysosome-related organelles complex 1 subunit 1	P78537	BLOC1S1	17 kDa	0.6598	downregulation
4370	Chloride channel CLIC-like protein 1	Q96S66	CLCC1	62 kDa	1.8404	upregulation

4380	Telomeric repeat-binding factor 2	Q15554	TERF2	60 kDa	0.5905	downregulation
4399	Histone H2A.V	Q71UI9	H2AFV	14 kDa	2.1435	upregulation
4401	RelA-associated inhibitor	Q8WUF5	PPP1R13L	89 kDa	0.5824	downregulation
4426	NAD-dependent protein deacetylase sirtuin-6	Q8N6T7	SIRT6	39 kDa	0.6643	downregulation
4449	Inter-alpha-trypsin inhibitor heavy chain H3	E7ET33	ITIH3	78 kDa	0.5000	downregulation
4468	Codanin-1	Q8IWY9	CDAN1	134 kDa	0.4147	downregulation
4481	ITIH4 protein	B7ZKJ8	ITIH4	104 kDa	0.4569	downregulation
4506	WD repeat-containing protein 47	O94967	WDR47	102 kDa	0.6029	downregulation
4515	Rhophilin-2	Q8IUC4	RHPN2	77 kDa	1.8661	upregulation
4516	Mitochondrial import receptor subunit TOM5 homolog	Q8N4H5	TOMM5	6 kDa	1.5692	upregulation
4523	Protein piccolo	Q9Y6V0	PCLO	553 kDa	0.6643	downregulation
4529	A-kinase anchor protein 4	Q5JQC9	AKAP4	94 kDa	0.4931	downregulation
4611	Annexin A8-like protein 1	A0A075B752	ANXA8L1	41 kDa	0.5946	downregulation
4627	Thioredoxin domain-containing protein 9	O14530	TXNDC9	27 kDa	1.9725	upregulation
4639	Vitamin K epoxide reductase complex subunit 1-like protein 1	Q8N0U8	VKORC1L1	20 kDa	2.3457	upregulation
4673	RNA-binding protein 5	P52756	RBM5	92 kDa	0.6242	downregulation
4701	Integrator complex subunit 8	Q75QN2	INTS8	113 kDa	0.5285	downregulation
4758	Exocyst complex component 6B	Q9Y2D4	EXOC6B	94 kDa	0.6373	downregulation
4783	FAST kinase domain-containing protein 1	D3DPC4	FLJ21901	95 kDa	0.4897	downregulation
4787	Cytoskeleton-associated protein 2	Q8WWK9	CKAP2	77 kDa	0.6417	downregulation
4807	DnaJ homolog subfamily C member 15	Q9Y5T4	DNAJC15	16 kDa	0.6643	downregulation
4812	Collagen alpha-1(XI) chain	P12107	COL11A1	181 kDa	1.9185	upregulation
4819	RING finger protein unkempt homolog	Q9C0B0	UNK	88 kDa	2.2346	upregulation
4829	NAD-dependent protein deacetylase sirtuin-3, mitochondrial	E9PM75	SIRT3	38 kDa	2.3295	upregulation
4835	Nuclear pore complex protein Nup153	P49790	NUP153	154 kDa	1.5911	upregulation
4847	Sorting nexin-15	Q9NRS6	SNX15	38 kDa	0.6373	downregulation
4857	RNA-binding protein 40 (Fragment)	H0YEB8	RNPC3	21 kDa	1.7411	upregulation
4862	Coiled-coil domain-containing protein 38	Q502W7	CCDC38	65 kDa	1.5583	upregulation
4888	Methylsterol monooxygenase 1	Q15800	MSMO1	35 kDa	2.4967	upregulation
4893	39S ribosomal protein L43, mitochondrial	B1AL05	MRPL43	21 kDa	1.5911	upregulation
4899	Mitochondrial peptide methionine sulfoxide reductase	Q9UJ68	MSRA	26 kDa	0.6373	downregulation
4904	Trafficking protein particle complex subunit 2	P0DI81	TRAPPC2	16 kDa	2.0562	upregulation
4919	Uncharacterized protein (Fragment)	H0YAE9		22 kDa	2.0279	upregulation
4921	Selenoprotein T	A0A087WTN3	SELT	19 kDa	0.6643	downregulation
5046	Allograft inflammatory factor 1-like	Q9BQI0	AIF1L	17 kDa	1.5369	upregulation
5075	Putative peripheral benzodiazepine receptor-related protein (Fragment)	B1AH87	TSPO	12 kDa	1.7411	upregulation

5076	Krev interaction trapped protein 1	O00522	KRIT1	84 kDa	1.7171	upregulation
5080	Nidogen-2	Q14112	NID2	151 kDa	0.5322	downregulation
5091	Fatty acid-binding protein, heart	P05413	FABP3	15 kDa	0.6507	downregulation
5125	Phosphorylase b kinase regulatory subunit alpha, liver isoform	P46019	PHKA2	138 kDa	0.6598	downregulation
5127	Zinc finger protein 346	D6RJ07	ZNF346	35 kDa	0.2588	downregulation
5131	[Pyruvate dehydrogenase [acetyl-transferring]]-phosphatase 1, mitochondrial	Q9P0J1	PDP1	61 kDa	0.5946	downregulation
5137	Protein BRICK1	Q8WUW1	BRK1	9 kDa	2.2038	upregulation
5138	Rho GTPase-activating protein 42	E9PJK4	ARHGAP42	95 kDa	0.5864	downregulation
5144	Sorbin and SH3 domain-containing protein 2	O94875	SORBS2	124 kDa	0.5824	downregulation
5155	Transient receptor potential cation channel subfamily M member 8	Q7Z2W7	TRPM8	128 kDa	0.5322	downregulation
5156	Inward rectifier potassium channel 13 (Fragment)	H7C4D1	KCNJ13	16 kDa	1.5476	upregulation
5164	Protein RTEL1-TNFRSF6B	F6WH68	RTEL1-TNFRSF6B	153 kDa	2.4967	upregulation
5168	Crossover junction endonuclease MUS81	H0YDU2	MUS81	45 kDa	2.2038	upregulation
5170	Kinesin-like protein KIF28P	B7ZC32	KIF28P	108 kDa	1.8150	upregulation
5178	Perilipin-4	Q96Q06	PLIN4	134 kDa	2.1435	upregulation
5213	DNA-binding protein RFXANK	O14593	RFXANK	28 kDa	0.6643	downregulation
5240	Protein FAM98C	Q17RN3	FAM98C	37 kDa	0.6643	downregulation
5245	1-acyl-sn-glycerol-3-phosphate acyltransferase alpha	Q99943	AGPAT1	32 kDa	0.6417	downregulation
5248	Protein kish-A	Q8TBQ9	TMEM167A	8 kDa	1.6021	upregulation
5253	Proline-rich AKT1 substrate 1	Q96B36	AKT1S1	27 kDa	1.5911	upregulation
5280	Protein phosphatase 1 regulatory subunit 11	O60927	PPP1R11	14 kDa	0.1948	downregulation
5287	Actin-related protein 6	Q9GZN1	ACTR6	46 kDa	0.6462	downregulation
5294	Protein jagunal homolog 1	A0A087WY88	JAGN1	21 kDa	1.6702	upregulation
5295	Complement component C7	P10643	C7	94 kDa	0.5864	downregulation
5297	Hemoglobin subunit delta	E9PFT6	HBD	15 kDa	1.5476	upregulation
5331	Cell adhesion molecule 1	A0A087WZR6	CADM1	48 kDa	0.6643	downregulation
5346	Tissue factor pathway inhibitor (Fragment)	C9JBB3	TFPI	33 kDa	0.6329	downregulation
5352	Glutathione peroxidase 3	A0A087X1J7	GPX3	25 kDa	0.5704	downregulation
5354	RNMT-activating mini protein	Q9BTL3	FAM103A1	14 kDa	0.4538	downregulation
5374	Neurogranin	Q92686	NRGN	8 kDa	2.5669	upregulation
5375	39S ribosomal protein L33, mitochondrial	O75394	MRPL33	8 kDa	1.6358	upregulation
5385	Interferon-stimulated gene 20 kDa protein	Q96AZ6	ISG20	20 kDa	3.1167	upregulation
5388	Protein SCAI	Q8N9R8	SCAI	70 kDa	0.5070	downregulation
5403	Prenylated Rab acceptor protein 1 (Fragment)	M0R1H9	RABAC1	17 kDa	1.5157	upregulation
5417	Proton-coupled folate transporter	Q96NT5	SLC46A1	50 kDa	0.5396	downregulation
5422	YY1-associated factor 2	Q8IY57	YAF2	20 kDa	0.6113	downregulation
5423	Golgi-associated plant pathogenesis-related protein 1	Q9H4G4	GLIPR2	17 kDa	0.6552	downregulation
5426	Aldo-keto reductase family 1 member C2	P52895	AKR1C2	37 kDa	1.5583	upregulation

5436	B-cell CLL/lymphoma 7 protein family member B	Q9BQE9	BCL7B	22 kDa	0.6552	downregulation
5445	Receptor expression-enhancing protein 6	Q96HR9	REEP6	21 kDa	1.6472	upregulation
5488	TAF5-like RNA polymerase II p300/CBP-associated factor-associated factor 65 kDa subunit 5L	O75529	TAF5L	66 kDa	0.4569	downregulation
5519	Solute carrier family 12 member 8	A0AV02	SLC12A8	78 kDa	0.6373	downregulation
5520	Inactive serine protease PAMR1 (Fragment)	E9PQ70	PAMR1	75 kDa	2.1140	upregulation

Table S5 BP enrichment analysis of differentially expressed proteins

<table>
<tr><th>levels</th><th>Genes</th><th>GO_Name</th><th>GO_ID</th><th>Pvalue</th><th>Pvalue_adjusted</th><th>Count</th><th>maxLevel</th></tr>
<tr><td>3</td><td>HSPA8|0.3721;ACTG1|2.1999;HSP90AB1|0.458775;LCP1|2.170475;ENO1|2.1939;HSPD1|2.88995;HIST1H2BN|0.481625;RPL3|3.30745;VIM|2.241075;ASPH|2.59965;ATP6V1B2|2.0575;MCM3|0.35205;KRT9|0.268525;RPL4|2.063325;NSF|2.017425;MCM2|0.491625;SMC2|0.485025;DNMT1|0.172;HIST1H4A|0.23835;VDAC3|2.015475;YWHAG|2.359475;ANXA5|3.637725;GSN|2.4555;NCAPG|0.4528;CBX3|0.485775;LIG1|0.440425;G3BP1|0.3904;ERO1L|2.370575;BAZ1B|0.460625;RRM2|0.21675;MCMBP|0.4769;RPL27|2.15885;SQSTM1|6.007525;ATG7|2.120975;PCNA|0.404975;MAPK14|0.483325;HIST1H3A|0.455625;TFRC|0.403125;RHOG|2.449775;TOP2A|0.481675;SNW1|0.473175;STAT3|0.4995;TUBG1|0.4102;NCDN|0.4114;SLC2A1|5.081875;VAT1|2.702925;PLEK|2.071125;NDE1|0.446425;HBA1|2.13735;NDRG1|10.0073;VTN|2.01125;RBBP7|0.218475;ARHGDIA|3.8215;ACTB|2.1999;H3F3A|0.20985;MYH10|5.579275;TTK|0.488375;SOS2|11.48525;PLK1|0.415475;GCH1|4.01735;SOD2|2.175425;TBCA|0.3515;CETN3|0[illegible]</td><td>cellular component organization</td><td>GO:0016043</td><td>5.90E-17</td><td>1.62E-13</td><td>65</td><td>3</td></tr>
<tr><td>2</td><td>HSPA8|0.3721;ACTG1|2.1999;HSP90AB1|0.458775;LCP1|2.170475;ENO1|2.1939;HSPD1|2.88995;HIST1H2BN|0.481625;RPL3|3.30745;VIM|2.241075;ASPH|2.59965;ATP6V1B2|2.0575;MCM3|0.35205;KRT9|0.268525;RPL4|2.063325;NSF|2.017425;MCM2|0.491625;SMC2|0.485025;DNMT1|0.172;HIST1H4A|0.23835;VDAC3|2.015475;YWHAG|2.359475;ANXA5|3.637725;GSN|2.4555;NCAPG|0.4528;CBX3|0.485775;LIG1|0.440425;G3BP1|0.3904;ERO1L|2.370575;BAZ1B|0.460625;RRM2|0.21675;MCMBP|0.4769;RPL27|2.15885;SQSTM1|6.007525;ATG7|2.120975;PCNA|0.404975;MAPK14|0.483325;HIST1H3A|0.455625;TFRC|0.403125;RHOG|2.449775;TOP2A|0.481675;SNW1|0.473175;STAT3|0.4995;TUBG1|0.4102;NCDN|0.4114;SLC2A1|5.081875;VAT1|2.702925;PLEK|2.071125;NDE1|0.446425;HBA1|2.13735;NDRG1|10.0073;VTN|2.01125;RBBP7|0.218475;ARHGDIA|3.8215;ACTB|2.1999;H3F3A|0.20985;MYH10|5.579275;TTK|0.488375;SOS2|11.48525;PLK1|0.415475;GCH1|4.01735;SOD2|2.175425;TBCA|0.3515;CETN3|0[illegible]</td><td>cellular component organization or biogenesis</td><td>GO:0071840</td><td>3.08E-16</td><td>4.24E-13</td><td>65</td><td>2</td></tr>
<tr><td>4</td><td>HSPA8|0.3721;HSP90AB1|0.458775;HSPD1|2.88995;RPL3|3.30745;VIM|2.241075;ATP6V1B2|2.0575;RPL4|2.063325;KPNA2|0.186675;GSN|2.4555;GTF2E1|0.300475;PSME3|0.401575;EIF4H|0.275675;RPL27|2.15885;SQSTM1|6.007525;ATG7|2.120975;TFRC|0.403125;TOP2A|0.481675;SNW1|0.473175;STAT3|0.4995;ALB|0.23405;IFITM2|5.683</td><td>symbiosis, encompassing mutualism through parasitism</td><td>GO:0044403</td><td>1.10E-13</td><td>7.58E-11</td><td>21</td><td>4</td></tr>
<tr><td>3</td><td>HSPA8|0.3721;HSP90AB1|0.458775;HSPD1|2.88995;RPL3|3.30745;VIM|2.241075;ATP6V1B2|2.0575;RPL4|2.063325;KPNA2|0.186675;GSN|2.4555;GTF2E1|0.300475;PSME3|0.401575;EIF4H|0.275675;RPL27|2.15885;SQSTM1|6.007525;ATG7|2.120975;TFRC|0.403125;TOP2A|0.481675;SNW1|0.473175;STAT3|0.4995;ALB|0.23405;IFITM2|5.683</td><td>interspecies interaction between organisms</td><td>GO:0044419</td><td>1.10E-13</td><td>7.58E-11</td><td>21</td><td>3</td></tr>
<tr><td>5</td><td>HSPA8|0.3721;LCP1|2.170475;HSPD1|2.88995;HIST1H2BN|0.481625;RPL3|3.30745;VIM|2.241075;ASPH|2.59965;KRT9|0.268525;RPL4|2.063325;MCM2|0.491625;SMC2|0.485025;HIST1H4A|0.23835;ANXA5|3.637725;GSN|2.4555;RRM2|0.21675;RPL27|2.15885;SQSTM1|6.007525;HIST1H3A|0.455625;TUBG1|0.4102;SLC2A1|5.081875;PLEK|2.071125;NDE1|0.446425;HBA1|2.13735;RBBP7|0.218475;H3F3A|0.20985;TTK|0.488375;PLK1|0.415475;GCH1|4.01735;SOD2|2.175425;TBCA|0.3515;CHAF1B|0.2723</td><td>protein complex subunit organization</td><td>GO:0071822</td><td>6.69E-13</td><td>3.69E-10</td><td>31</td><td>5</td></tr>
<tr><td>4</td><td>HSPA8|0.3721;LCP1|2.170475;HSPD1|2.88995;HIST1H2BN|0.481625;RPL3|3.30745;VIM|2.241075;ASPH|2.59965;KRT9|0.268525;RPL4|2.063325;MCM2|0.491625;SMC2|0.485025;DNMT1|0.172;HIST1H4A|0.23835;ANXA5|3.637725;GSN|2.4555;CBX3|0.485775;BAZ1B|0.460625;RRM2|0.21675;RPL27|2.15885;SQSTM1|6.007525;ATG7|2.120975;HIST1H3A|0.455625;SNW1|0.473175;TUBG1|0.4102;SLC2A1|5.081875;PLEK|2.071125;NDE1|0.446425;HBA1|2.13735;RBBP7|0.218475;ACTB|2.1999;H3F3A|0.20985;TTK|0.488375;PLK1|0.415475;GCH1|4.01735;SOD2|2.175425;</td><td>macromolecular complex subunit organization</td><td>GO:0043933</td><td>1.35E-12</td><td>6.19E-10</td><td>37</td><td>4</td></tr>
<tr><td>3</td><td>HSPA8|0.3721;ACTG1|2.1999;HSP90AB1|0.458775;HSPD1|2.88995;PABPC1|0.3285;ASPH|2.59965;KRT1|0.4198;ATP6V1B2|2.0575;CKB|0.486625;HIST1H4A|0.23835;YWHAG|2.359475;ANXA5|3.637725;GSN|2.4555;AKR1B1|2.235325;PABPC4|0.26245;LIG1|0.440425;ERO1L|2.370575;PRDX5|3.0143;ATG7|2.120975;PCNA|0.404975;MAPK14|0.483325;HIST1H3A|0.455625;TFRC|0.403125;RHOG|2.449775;SNW1|0.473175;STAT3|0.4995;NCDN|0.4114;SLC2A1|5.081875;PLEK|2.071125;VTN|2.01125;ARHGDIA|3.8215;ACTB|2.1999;H3F3A|0.20985;MYH10|5.579275;SOS2|11.48525;ALB|0.23405;PLK1|0.415475;GCH1|4.017</td><td>regulation of biological quality</td><td>GO:0065008</td><td>3.39E-12</td><td>1.29E-09</td><td>40</td><td>3</td></tr>
<tr><td>3</td><td>HSPA8|0.3721;HSP90AB1|0.458775;HSPD1|2.88995;RPL3|3.30745;VIM|2.241075;RPL4|2.063325;KPNA2|0.186675;GSN|2.4555;GTF2E1|0.300475;PSME3|0.401575;EIF4H|0.275675;RPL27|2.15885;ATG7|2.120975;TFRC|0.403125;TOP2A|0.481675;SNW1|0.473175;STAT3|0.4995;ALB|0.2</td><td>multi-organism cellular process</td><td>GO:0044764</td><td>3.75E-12</td><td>1.29E-09</td><td>19</td><td>3</td></tr>
</table>

3	HSPA8|0.3721;ACTG1|2.1999;HSP90AB1|0.458775;HSPD1|2.88995;RPL3|3.30745;ASPH|2.59965;ATP6V1B2|2.0575;MCM2|0.491625;HSPA4L|2.13105;DNMT1|0.172;KPNA2|0.186675;ANXA5|3.637725;SRRT|0.39875;GSN|2.4555;CNDP2|2.0938;AKR1B1|2.235325;LIG1|0.440425;ERO1L|2.370575;PRDX5|3.0143;LAMTOR2|7.841025;SQSTM1|6.007525;ATG7|2.120975;PCNA|0.404975;MAPK14|0.483325;TFRC|0.403125;RHOG|2.449775;SNW1|0.473175;STAT3|0.4995;TXNIP|2.0553;HBA1|2.13735;NDRG1|10.0073;VTN|2.01125;RBBP7|0.218475;ARHGDIA|3.8215;ACTB|2.1999;H3F3A|0.20985;MYH10|5.579275;SOS2|11.48525;GCH1|4.01735;SOD2|2.175425;PTP4A3|0.3433;IFITM2|	response to chemical	GO:0042221	4.86E-12	1.49E-09	42	3
2	HSPA8|0.3721;HSP90AB1|0.458775;ENO1|2.1939;HSPD1|2.88995;RPL3|3.30745;VIM|2.241075;CCT7|2.06135;ATP6V1B2|2.0575;KRT9|0.268525;RPL4|2.063325;KPNA2|0.186675;ANXA5|3.637725;GSN|2.4555;GTF2E1|0.300475;AKR1B1|2.235325;PSME3|0.401575;EIF4H|0.275675;RPL27|2.15885;SQSTM1|6.007525;ATG7|2.120975;MAPK14|0.483325;HIST1H3A|0.455625;TFRC|0.403125;TOP2A|0.481675;SNW1|0.473175;STAT3|0.4995;ALB|0.23405;GCH1|4.01735;IFITM2|5.68315;CHAF1B|0.2723	multi-organism process	GO:0051704	6.83E-12	1.88E-09	30	2
2	HSPA8|0.3721;ACTG1|2.1999;HSP90AB1|0.458775;LCP1|2.170475;ENO1|2.1939;HSPD1|2.88995;RPL3|3.30745;VIM|2.241075;CCT7|2.06135;PABPC1|0.3285;SARS|2.2366;ASPH|2.59965;KRT1|0.4198;YARS|2.288275;ATP6V1B2|2.0575;MCM3|0.35205;KRT9|0.268525;RPL4|2.063325;NSF|2.017425;GMPS|0.496875;MTHFD1L|6.667175;CKB|0.486625;NARS|2.162;MCM2|0.491625;SMC2|0.485025;DNMT1|0.172;ALDH1L2|2.148;HIST1H4A|0.23835;KPNA2|0.186675;VDAC3|2.015475;YWHAG|2.359475;ANXA5|3.637725;SRRT|0.39875;GSN|2.4555;CNDP2|2.0938;NCAPG|0.4528;AKR1B1|2.235325;PABPC4|0.26245;LIG1|0.440425;SLC25A1|2.1121;G3BP1|0.3904;ERO1L|2.370575;PRDX5|3.0143;BAZ1B|0.460625;ESD|2.155525;ISYNA1|0.49065;ALDOC|2.04725;PSME3|0.401575;RRM2|0.21675;EIF4H|0.275675;ACAT2|2.108325;LAMTOR2|7.841025;MCMBP|0.4769;RPL27|2.15885;SQSTM1|6.007525;ATG7|2.120975;PCNA|0.404975;MAPK14|0.483325;HIST1H3A|0.455625;BTF3|0.2449;TFRC|0.403125;RHOG|2.449775;TOP2A|0.481675;SNW1|0.473175;STAT3|0.4995;TUBG1|0.4102;NCDN|0.4114;SLC2A1|5.081875;TXNIP|2.0553;VAT1|2.702925;IPO8|0.443925;PLEK|2.071125;NDE1|0.446425;S100A11|2.531875;HBA1|2.13735;NDRG1|10.0073;IK|0.480125;VTN|2.01125;RBBP7|0.218475;ARHGDIA|3.8215;PLBD2|2.666725;ACTB|2.1999;H3F3A|0.20985;MYH10|5.579275;TTK|0.488375;SOS2|11.48525;ASL|2.252975;ALB|0.23405;CDC123|0.29925;PLK1|0.415475;GCH1|4.01735;SOD2|2.175425;PTP4A3|0.3433;HEATR9|2.53905;CETN3 [illegible]	single-organism process	GO:0044699	1.58E-11	3.94E-09	99	2
3	HSPA8|0.3721;HSP90AB1|0.458775;ENO1|2.1939;HSPD1|2.88995;VIM|2.241075;PABPC1|0.3285;ASPH|2.59965;DNMT1|0.172;HIST1H4A|0.23835;YWHAG|2.359475;ANXA5|3.637725;SRRT|0.39875;GSN|2.4555;CBX3|0.485775;G3BP1|0.3904;ERO1L|2.370575;PRDX5|3.0143;PSME3|0.401575;SQSTM1|6.007525;ATG7|2.120975;MAPK14|0.483325;HIST1H3A|0.455625;TOP2A|0.481675;SNW1|0.473175;STAT3|0.4995;TXNIP|2.0553;VAT1|2.702925;PLEK|2.071125;S100A11|2.531875;NDRG1|10.0073;VTN|2.01125;RBBP7|0.218475;ARHGDIA|3.8215;H3F3A|0.20985;TTK|0.488375;ALB|0.23405;CDC123|0.29925;PLK1|0.415475;SOD2|2.175425;IFITM2|5.68315;ZNF706|0.3974	negative regulation of cellular process	GO:0048523	1.86E-11	4.26E-09	41	3
4,5	HSPA8|0.3721;HSP90AB1|0.458775;HSPD1|2.88995;RPL3|3.30745;VIM|2.241075;RPL4|2.063325;KPNA2|0.186675;GSN|2.4555;GTF2E1|0.300475;PSME3|0.401575;EIF4H|0.275675;RPL27|2.15885;ATG7|2.120975;TFRC|0.403125;TOP2A|0.481675;SNW1|0.473175;STAT3|0.4995;IFITM2|	viral process	GO:0016032	2.75E-11	5.50E-09	18	5

3	HSPA8|0.3721;ACTG1|2.1999;HSP90AB1|0.458775;LCP1|2.170475;ENO1|2.1939;HSPD1|2.88995;RPL3|3.30745;VIM|2.241075;CCT7|2.06135;PABPC1|0.3285;SARS|2.2366;ASPH|2.59965;YARS|2.288275;ATP6V1B2|2.0575;MCM3|0.35205;KRT9|0.268525;RPL4|2.063325;NSF|2.017425;GMPS|0.496875;MTHFD1L|6.667175;CKB|0.486625;NARS|2.162;MCM2|0.491625;SMC2|0.485025;DNMT1|0.172;ALDH1L2|2.148;HIST1H4A|0.23835;KPNA2|0.186675;VDAC3|2.015475;YWHAG|2.359475;ANXA5|3.637725;SRRT|0.39875;GSN|2.4555;CNDP2|2.0938;NCAPG|0.4528;AKR1B1|2.235325;LIG1|0.440425;SLC25A1|2.1121;G3BP1|0.3904;ERO1L|2.370575;PRDX5|3.0143;BAZ1B|0.460625;ESD|2.155525;ISYNA1|0.49065;ALDOC|2.04725;PSME3|0.401575;RRM2|0.21675;LAMTOR2|7.841025;MCMBP|0.4769;RPL27|2.15885;SQSTM1|6.007525;ATG7|2.120975;PCNA|0.404975;MAPK14|0.483325;HIST1H3A|0.455625;TFRC|0.403125;RHOG|2.449775;TOP2A|0.481675;SNW1|0.473175;STAT3|0.4995;TUBG1|0.4102;NCDN|0.4114;SLC2A1|5.081875;TXNIP|2.0553;VAT1|2.702925;IPO8|0.443925;PLEK|2.071125;NDE1|0.446425;S100A11|2.531875;HBA1|2.13735;NDRG1|10.0073;IK|0.480125;VTN|2.01125;RBBP7|0.218475;ARHGDIA|3.8215;ACTB|2.1999;H3F3A|0.20985;MYH10|5.579275;TTK|0.488375;SOS2|11.48525;ASL|2.252975;ALB|0.23405;CDC123|0.29925;PLK1|0.415475;GCH1|4.01735;SOD2|2.175425;PTP4A3|0.3433;HEATR9|2.53905;CETN3|0.4171;IFITM2|5.68315;ZNF706|0.3974;	single-organism cellular process	GO:0044763	2.80E-11	5.50E-09	93	3
3	HSPA8|0.3721;ACTG1|2.1999;HSP90AB1|0.458775;LCP1|2.170475;HSPD1|2.88995;KRT1|0.4198;HSPA4L|2.13105;ANXA5|3.637725;GSN|2.4555;AKR1B1|2.235325;PABPC4|0.26245;LIG1|0.440425;ERO1L|2.370575;PRDX5|3.0143;BAZ1B|0.460625;PSME3|0.401575;SQSTM1|6.007525;ATG7|2.120975;PCNA|0.404975;MAPK14|0.483325;HIST1H3A|0.455625;TFRC|0.403125;RHOG|2.449775;TOP2A|0.481675;SNW1|0.473175;STAT3|0.4995;SLC2A1|5.081875;TXNIP|2.0553;PLEK|2.071125;HBA1|2.13735;NDRG1|10.0073;VTN|2.01125;RBBP7|0.218475;ACTB|2.1999;H3F3A|0.20985;MYH10|5.579275;ALB|0.23405;PLK1|0.415475;GCH1|4.01735;SOD2|2.175425;IFITM2|5.68315;CHAF1B	response to stress	GO:0006950	3.99E-11	7.32E-09	43	3
4	ACTG1|2.1999;LCP1|2.170475;HIST1H2BN|0.481625;RPL3|3.30745;VIM|2.241075;ATP6V1B2|2.0575;MCM3|0.35205;KRT9|0.268525;MCM2|0.491625;SMC2|0.485025;DNMT1|0.172;HIST1H4A|0.23835;VDAC3|2.015475;YWHAG|2.359475;ANXA5|3.637725;GSN|2.4555;NCAPG|0.4528;CBX3|0.485775;LIG1|0.440425;G3BP1|0.3904;BAZ1B|0.460625;MCMBP|0.4769;SQSTM1|6.007525;ATG7|2.120975;PCNA|0.404975;MAPK14|0.483325;HIST1H3A|0.455625;RHOG|2.449775;TOP2A|0.481675;SNW1|0.473175;STAT3|0.4995;TUBG1|0.4102;VAT1|2.702925;PLEK|2.071125;NDE1|0.446425;RBBP7|0.218475;ACTB|2.1999;H3F3A|0.20985;MYH10|5.579275;TTK|0.488375;PLK1|0.415475;S	organelle organization	GO:0006996	5.24E-11	9.01E-09	44	4
2	HSPA8|0.3721;HSP90AB1|0.458775;ENO1|2.1939;HSPD1|2.88995;VIM|2.241075;PABPC1|0.3285;ASPH|2.59965;KRT1|0.4198;DNMT1|0.172;HIST1H4A|0.23835;YWHAG|2.359475;ANXA5|3.637725;SRRT|0.39875;GSN|2.4555;CBX3|0.485775;G3BP1|0.3904;ERO1L|2.370575;PRDX5|3.0143;PSME3|0.401575;SQSTM1|6.007525;ATG7|2.120975;MAPK14|0.483325;HIST1H3A|0.455625;TOP2A|0.481675;SNW1|0.473175;STAT3|0.4995;TXNIP|2.0553;VAT1|2.702925;PLEK|2.071125;S100A11|2.531875;NDRG1|10.0073;VTN|2.01125;RBBP7|0.218475;ARHGDIA|3.8215;H3F3A|0.20985;TTK|0.488375;ALB|0.23405;CDC123|0.29925;PLK1|0.415475;SOD2|2.175425;IFITM2|5.68315;ZNF706|0.3	negative regulation of biological process	GO:0048519	6.52E-11	1.06E-08	42	2
2	HSPA8|0.3721;ACTG1|2.1999;HSP90AB1|0.458775;LCP1|2.170475;HSPD1|2.88995;VIM|2.241075;ASPH|2.59965;KRT1|0.4198;KRT9|0.268525;MTHFD1L|6.667175;CKB|0.486625;HIST1H4A|0.23835;VDAC3|2.015475;YWHAG|2.359475;SRRT|0.39875;GSN|2.4555;AKR1B1|2.235325;LIG1|0.440425;ERO1L|2.370575;BAZ1B|0.460625;ALDOC|2.04725;EIF4H|0.275675;SQSTM1|6.007525;ATG7|2.120975;PCNA|0.404975;MAPK14|0.483325;BTF3|0.2449;TFRC|0.403125;RHOG|2.449775;TOP2A|0.481675;SNW1|0.473175;STAT3|0.4995;NCDN|0.4114;TXNIP|2.0553;VAT1|2.702925;PLEK|2.071125;NDE1|0.446425;NDRG1|10.0073;VTN|2.01125;RBBP7|0.218475;ARHGDIA|3.8215;ACTB|2.1999;H3F3A|0.20985;MYH10|5.579275;SOS2|11.48525;SOD2|2.175425;HEATR9|2.53905;ZNF706|0.3974;PLP1|3.9	developmental process	GO:0032502	7.82E-11	1.20E-08	49	2
5	HIST1H2BN|0.481625;MCM3|0.35205;MCM2|0.491625;SMC2|0.485025;DNMT1|0.172;HIST1H4A|0.23835;NCAPG|0.4528;CBX3|0.485775;LIG1|0.440425;G3BP1|0.3904;BAZ1B|0.460625;MCMBP|0.4769;ATG7|2.120975;PCNA|0.404975;HIST1H3A|0.455625;TOP2A|0.481675;SNW1|0.473175;RBBP7|0.218475;ACTB|2.1999;H3F3A|0.20985;TTK|0.488375;PLK1|0.415475;CHAF1B|0.2723	chromosome organization	GO:0051276	1.22E-10	1.77E-08	23	5

3	HSPA8|0.3721;ACTG1|2.1999;HSP90AB1|0.458775;LCP1|2.170475;HSPD1|2.88995;VIM|2.241075;ASPH|2.59965;KRT1|0.4198;KRT9|0.268525;MTHFD1L|6.667175;CKB|0.486625;HIST1H4A|0.23835;VDAC3|2.015475;YWHAG|2.359475;SRRT|0.39875;GSN|2.4555;AKR1B1|2.235325;LIG1|0.440425;ERO1L|2.370575;BAZ1B|0.460625;ALDOC|2.04725;EIF4H|0.275675;SQSTM1|6.007525;ATG7|2.120975;PCNA|0.404975;MAPK14|0.483325;BTF3|0.2449;TFRC|0.403125;RHOG|2.449775;TOP2A|0.481675;SNW1|0.473175;STAT3|0.4995;NCDN|0.4114;TXNIP|2.0553;PLEK|2.071125;NDE1|0.446425;NDRG1|10.0073;VTN|2.01125;RBBP7|0.218475;ARHGDIA|3.8215;ACTB|2.1999;H3F3A|0.20985;MYH10|5.579275;SOS2|11.48525;SOD2|2.175425;HE[illegible]	single-organism developmental process	GO:0044767	1.57E-10	2.16E-08	48	3
4	ACTG1|2.1999;HSPD1|2.88995;ASPH|2.59965;ANXA5|3.637725;GSN|2.4555;AKR1B1|2.235325;LIG1|0.440425;PCNA|0.404975;TFRC|0.403125;TXNIP|2.0553;HBA1|2.13735;NDRG1|10.0073;SOD2|2.175425	response to inorganic substance	GO:0010035	3.73E-10	4.89E-08	13	4
3	HSPA8|0.3721;ACTG1|2.1999;HSP90AB1|0.458775;LCP1|2.170475;HSPD1|2.88995;VIM|2.241075;ASPH|2.59965;KRT1|0.4198;KRT9|0.268525;MTHFD1L|6.667175;CKB|0.486625;HIST1H4A|0.23835;VDAC3|2.015475;YWHAG|2.359475;SRRT|0.39875;GSN|2.4555;AKR1B1|2.235325;LIG1|0.440425;BAZ1B|0.460625;ALDOC|2.04725;ATG7|2.120975;PCNA|0.404975;MAPK14|0.483325;BTF3|0.2449;TFRC|0.403125;RHOG|2.449775;TOP2A|0.481675;SNW1|0.473175;STAT3|0.4995;NCDN|0.4114;TXNIP|2.0553;VAT1|2.702925;PLEK|2.071125;NDE1|0.446425;NDRG1|10.0073;VTN|2.01125;ARHGDIA|3.8215;ACTB|2.1999;H3F3A|0.20985;MYH10|5.579275;SOS2|11.48525;HEATR9|2.5390	anatomical structure development	GO:0048856	4.46E-10	5.58E-08	44	3
6	HIST1H2BN|0.481625;MCM3|0.35205;MCM2|0.491625;SMC2|0.485025;HIST1H4A|0.23835;NCAPG|0.4528;G3BP1|0.3904;HIST1H3A|0.455625;TOP2A|0.481675;RBBP7|0.218475;H3F3A|0.20985;CHAF1B|0.2723	DNA conformation change	GO:0071103	6.49E-10	7.77E-08	12	6
7	HIST1H2BN|0.481625;MCM2|0.491625;SMC2|0.485025;HIST1H4A|0.23835;NCAPG|0.4528;HIST1H3A|0.455625;TOP2A|0.481675;RBBP7|0.218475;H3F3A|0.20985;CHAF	DNA packaging	GO:0006323	1.31E-09	1.50E-07	10	7
4	HSPA8|0.3721;HSP90AB1|0.458775;HSPD1|2.88995;RPL3|3.30745;ASPH|2.59965;ATP6V1B2|2.0575;MCM2|0.491625;HSPA4L|2.13105;DNMT1|0.172;KPNA2|0.186675;ANXA5|3.637725;SRRT|0.39875;GSN|2.4555;AKR1B1|2.235325;ERO1L|2.370575;LAMTOR2|7.841025;SQSTM1|6.007525;PCNA|0.404975;MAPK14|0.483325;TFRC|0.403125;SNW1|0.473175;STAT3|0.4995;TXNIP|2.0553;VTN|2.01125;RBBP7|0.218475;ARHGDIA|3.8215;H3F3A|0.20985;SOS2|11.48525;GCH1|4.01735;PTP4A3|0.3433;IFITM2|5.68	response to organic substance	GO:0010033	1.41E-09	1.55E-07	31	4
3	HSPA8|0.3721;ACTG1|2.1999;HSP90AB1|0.458775;HSPD1|2.88995;VIM|2.241075;ASPH|2.59965;KRT1|0.4198;KRT9|0.268525;MTHFD1L|6.667175;CKB|0.486625;HIST1H4A|0.23835;YWHAG|2.359475;ANXA5|3.637725;SRRT|0.39875;GSN|2.4555;AKR1B1|2.235325;PABPC4|0.26245;LIG1|0.440425;PRDX5|3.0143;BAZ1B|0.460625;ATG7|2.120975;PCNA|0.404975;MAPK14|0.483325;HIST1H3A|0.455625;BTF3|0.2449;TFRC|0.403125;RHOG|2.449775;TOP2A|0.481675;SNW1|0.473175;STAT3|0.4995;NCDN|0.4114;TXNIP|2.0553;PLEK|2.071125;NDE1|0.446425;NDRG1|10.0073;VTN|2.01125;RBBP7|0.218475;ARHGDIA|3.8215;ACTB|2.1999;H3F3A|0.20985;MYH10|5.579275;SOS2|11.48525;ALB|0.23405;GCH1|4.01735;SOD2|2.175425;PTP4[illegible]	single-multicellular organism process	GO:0044707	1.64E-09	1.74E-07	49	3
5	ACTG1|2.1999;LCP1|2.170475;KRT1|0.4198;ANXA5|3.637725;GSN|2.4555;PABPC4|0.26245;MAPK14|0.483325;HIST1H3A|0.455625;RHOG|2.449775;PLEK|2.071125;VTN|2.01125;ACTB|2.1999;H3F3A|0.20985;MYH10|5.579275;	wound healing	GO:0042060	2.55E-09	2.41E-07	15	5
2	HSPA8|0.3721;ACTG1|2.1999;HSP90AB1|0.458775;LCP1|2.170475;ENO1|2.1939;HSPD1|2.88995;RPL3|3.30745;VIM|2.241075;ASPH|2.59965;KRT1|0.4198;YARS|2.288275;ATP6V1B2|2.0575;MCM2|0.491625;HSPA4L|2.13105;DNMT1|0.172;HIST1H4A|0.23835;KPNA2|0.186675;YWHAG|2.359475;ANXA5|3.637725;SRRT|0.39875;GSN|2.4555;CNDP2|2.0938;AKR1B1|2.235325;PABPC4|0.26245;LIG1|0.440425;G3BP1|0.3904;ERO1L|2.370575;PRDX5|3.0143;BAZ1B|0.460625;PSME3|0.401575;LAMTOR2|7.841025;SQSTM1|6.007525;ATG7|2.120975;PCNA|0.404975;MAPK14|0.483325;HIST1H3A|0.455625;TFRC|0.403125;RHOG|2.449775;TOP2A|0.481675;SNW1|0.473175;STAT3|0.4995;SLC2A1|5.081875;TXNIP|2.0553;IPO8|0.443925;PLEK|2.071125;NDE1|0.446425;S100A11|2.531875;HBA1|2.13735;NDRG1|10.0073;IK|0.480125;VTN|2.01125;RBBP7|0.218475;ARHGDIA|3.8215;ACTB|2.1999;H3F3A|0.20985;MYH10|5.579275;TTK|0.488375;SOS2|11.48525;ALB|0.23405;PLK1|0.415475;GCH1|4.01735;SOD2|2.175425;PTP4A[illegible]	response to stimulus	GO:0050896	2.55E-09	2.41E-07	66	2
4	LCP1|2.170475;HSPD1|2.88995;HIST1H2BN|0.481625;MCM2|0.491625;HIST1H4A|0.23835;ANXA5|3.637725;GSN|2.4555;RRM2|0.21675;SQSTM1|6.007525;HIST1H3A|0.455625;TUBG1|0.4102;SLC2A1|5.081875;PLEK|2.071125;NDE1|0.446425;HBA1|2.13735;RBBP7|0.218475;H3F3A|0.20985;PLK1|0.415475;GCH1|4.01735;SOD2|2.175425;T	protein complex biogenesis	GO:0070271	2.62E-09	2.41E-07	22	4

5,6	LCP1|2.170475;HSPD1|2.88995;HIST1H2BN|0.481625;MCM2|0.491625;HIST1H4A|0.23835;ANXA5|3.637725;GSN|2.4555;RRM2|0.21675;SQSTM1|6.007525;HIST1H3A|0.455625;TUBG1|0.4102;SLC2A1|5.081875;PLEK|2.071125;NDE1|0.446425;HBA1|2.13735;RBBP7|0.218475;H3F3A|0.20985;PLK1|0.415475;GCH1|4.01735;SOD2|2.175425;T	protein complex assembly	GO:0006461	2.62E-09	2.41E-07	22	6
4	HSPA8|0.3721;ACTG1|2.1999;HSP90AB1|0.458775;HSPD1|2.88995;VIM|2.241075;ASPH|2.59965;KRT1|0.4198;KRT9|0.268525;MTHFD1L|6.667175;CKB|0.486625;HIST1H4A|0.23835;YWHAG|2.359475;SRRT|0.39875;GSN|2.4555;AKR1B1|2.235325;LIG1|0.440425;BAZ1B|0.460625;ATG7|2.120975;PCNA|0.404975;MAPK14|0.483325;BTF3|0.2449;TFRC|0.403125;RHOG|2.449775;TOP2A|0.481675;SNW1|0.473175;STAT3|0.4995;NCDN|0.4114;TXNIP|2.0553;PLEK|2.071125;NDE1|0.446425;NDRG1|10.0073;VTN|2.01125;RBBP7|0.218475;ARHGDIA|3.8215;ACTB|2.1999;H3F3A|0.20985;MYH10|5.579275;SOS2|11.48525;HEA	multicellular organismal development	GO:0007275	3.80E-09	3.37E-07	40	4
8	HSPA8|0.3721;HSPD1|2.88995;CCT7|2.06135;ERO1L|2.370575;ACTB|2.1999;TBCA|0.3515	'de novo' posttranslational protein folding	GO:0051084	3.97E-09	3.41E-07	6	8
5	ACTG1|2.1999;KRT1|0.4198;HIST1H4A|0.23835;AKR1B1|2.235325;LIG1|0.440425;PRDX5|3.0143;PCNA|0.404975;TFRC|0.403125;NCDN|0.4114;ACTB|2.1999;ALB|0.2340	anatomical structure homeostasis	GO:0060249	4.14E-09	3.45E-07	11	5
4	HSPA8|0.3721;ACTG1|2.1999;HSP90AB1|0.458775;HSPD1|2.88995;VIM|2.241075;KRT1|0.4198;KRT9|0.268525;MTHFD1L|6.667175;CKB|0.486625;HIST1H4A|0.23835;YWHAG|2.359475;SRRT|0.39875;GSN|2.4555;AKR1B1|2.235325;LIG1|0.440425;BAZ1B|0.460625;ATG7|2.120975;PCNA|0.404975;MAPK14|0.483325;TFRC|0.403125;RHOG|2.449775;TOP2A|0.481675;SNW1|0.473175;STAT3|0.4995;NCDN|0.4114;TXNIP|2.0553;PLEK|2.071125;NDE1|0.446425;NDRG1|10.0073;VTN|2.01125;ARHGDIA|3.8215;ACTB|2.1999;H3F3A|0.20985;MYH10|5.579275;SOS2|11.4	system development	GO:0048731	5.55E-09	4.41E-07	37	4
2	HSPA8|0.3721;ACTG1|2.1999;HSP90AB1|0.458775;HSPD1|2.88995;VIM|2.241075;ASPH|2.59965;KRT1|0.4198;KRT9|0.268525;MTHFD1L|6.667175;CKB|0.486625;HIST1H4A|0.23835;YWHAG|2.359475;ANXA5|3.637725;SRRT|0.39875;GSN|2.4555;AKR1B1|2.235325;PABPC4|0.26245;LIG1|0.440425;PRDX5|3.0143;BAZ1B|0.460625;ATG7|2.120975;PCNA|0.404975;MAPK14|0.483325;HIST1H3A|0.455625;BTF3|0.2449;TFRC|0.403125;RHOG|2.449775;TOP2A|0.481675;SNW1|0.473175;STAT3|0.4995;NCDN|0.4114;TXNIP|2.0553;PLEK|2.071125;NDE1|0.446425;NDRG1|10.0073;VTN|2.01125;RBBP7|0.218475;ARHGDIA|3.8215;ACTB|2.1999;H3F3A|0.20985;MYH10|5.579275;SOS2|11.48525;ALB|0.23405;GCH1|4.01735;SOD2|2.175425;PTP4	multicellular organismal process	GO:0032501	5.60E-09	4.41E-07	49	2
7	HSPA8|0.3721;HSPD1|2.88995;CCT7|2.06135;ERO1L|2.370575;ACTB|2.1999;TBCA|0.3515	'de novo' protein folding	GO:0006458	6.53E-09	4.99E-07	6	7
3	HSPA8|0.3721;ACTG1|2.1999;HSP90AB1|0.458775;ENO1|2.1939;HSPD1|2.88995;KRT1|0.4198;ANXA5|3.637725;GSN|2.4555;SQSTM1|6.007525;ATG7|2.120975;MAPK14|0.483325;HIST1H3A|0.455625;TFRC|0.403125;RHOG|2.449775;SNW1|0.473175;SLC2A1|5.081875;TXNIP|2.0553;PLEK|2.071125;VTN|2.01125;ARHGDIA|3.8215;ACTB|2.1999;MYH10|5.579275;SOS2|11.48525;ALB|0.23405;GCH1|4.01735;IFITM2|5.68315;CHAF1B|0.2723	response to external stimulus	GO:0009605	7.50E-09	5.58E-07	27	3
6	ENO1|2.1939;SARS|2.2366;YARS|2.288275;GMPS|0.496875;MTHFD1L|6.667175;CKB|0.486625;NARS|2.162;ALDH1L2|2.148;CNDP2|2.0938;ERO1L|2.370575;ALDOC|2.04725;PSME3|0.401575;ATG7|2.120975;MAPK14|0.483325;STAT3|0.4995;SLC2A1|5.081875;ASL|2.252975;ALB|0.23405;GCH1|4.01735;PLP1|3.936	carboxylic acid metabolic process	GO:0019752	8.91E-09	6.46E-07	20	6
3	HSPA8|0.3721;ENO1|2.1939;HSPD1|2.88995;SARS|2.2366;ASPH|2.59965;KRT1|0.4198;YARS|2.288275;ATP6V1B2|2.0575;GMPS|0.496875;MTHFD1L|6.667175;CKB|0.486625;NARS|2.162;DNMT1|0.172;ALDH1L2|2.148;HIST1H4A|0.23835;ANXA5|3.637725;SRRT|0.39875;GSN|2.4555;CNDP2|2.0938;AKR1B1|2.235325;LIG1|0.440425;SLC25A1|2.1121;ERO1L|2.370575;PRDX5|3.0143;BAZ1B|0.460625;ESD|2.155525;ISYNA1|0.49065;ALDOC|2.04725;PSME3|0.401575;RRM2|0.21675;ACAT2|2.108325;LAMTOR2|7.841025;SQSTM1|6.007525;ATG7|2.120975;PCNA|0.404975;MAPK14|0.483325;TOP2A|0.481675;SNW1|0.473175;STAT3|0.4995;SLC2A1|5.081875;VAT1|2.702925;PLEK|2.071125;HBA1|2.13735;VTN|2.01125;PLBD2|2.666725;TTK|0.488375;ASL|2.252975;ALB|0.23405;PLK1|0.4154	single-organism metabolic process	GO:0044710	1.10E-08	7.61E-07	53	3
5	MCM3|0.35205;MCM2|0.491625;SMC2|0.485025;YWHAG|2.359475;NCAPG|0.4528;LIG1|0.440425;PSME3|0.401575;RRM2|0.21675;MCMBP|0.4769;PCNA|0.404975;TOP2A|0.481675;TUBG1|0.4102;NDE1|0.446425;MYH10|5.579275;TTK|0.488375;PLK1|0.415475;CETN3|0.4171	mitotic cell cycle process	GO:1903047	1.11E-08	7.61E-07	17	5
5	LCP1|2.170475;HSPD1|2.88995;HIST1H2BN|0.481625;RPL3|3.30745;MCM2|0.491625;HIST1H4A|0.23835;ANXA5|3.637725;GSN|2.4555;RRM2|0.21675;SQSTM1|6.007525;HIST1H3A|0.455625;TUBG1|0.4102;SLC2A1|5.081875;PLEK|2.071125;NDE1|0.446425;HBA1|2.13735;RBBP7|0.218475;H3F3A|0.20985;PLK1|0.415475;GCH1|4.01735;SOD2|2.175425;TBCA|0.3515;CHAF1B|0.2723	macromolecular complex assembly	GO:0065003	1.20E-08	8.08E-07	23	5

5	MCM3|0.35205;MCM2|0.491625;SMC2|0.485025;HIST1H4A|0.23835;YWHAG|2.359475;NCAPG|0.4528;LIG1|0.440425;PSME3|0.401575;RRM2|0.21675;MCMBP|0.4769;PCNA|0.404975;TOP2A|0.481675;TUBG1|0.4102;NDE1|0.446425;MYH10|5.579275;TTK|0.488375;CDC123|0.29925;PLK1|0.415475;CETN3|0.4171	mitotic cell cycle	GO:0000278	1.25E-08	8.17E-07	19	5
3	HSPA8|0.3721;ACTG1|2.1999;HSP90AB1|0.458775;LCP1|2.170475;HSPD1|2.88995;RPL3|3.30745;VIM|2.241075;ASPH|2.59965;YARS|2.288275;ATP6V1B2|2.0575;MCM2|0.491625;DNMT1|0.172;HIST1H4A|0.23835;KPNA2|0.186675;YWHAG|2.359475;ANXA5|3.637725;SRRT|0.39875;GSN|2.4555;CNDP2|2.0938;AKR1B1|2.235325;LIG1|0.440425;G3BP1|0.3904;ERO1L|2.370575;PRDX5|3.0143;BAZ1B|0.460625;PSME3|0.401575;LAMTOR2|7.841025;SQSTM1|6.007525;ATG7|2.120975;PCNA|0.404975;MAPK14|0.483325;HIST1H3A|0.455625;TFRC|0.403125;RHOG|2.449775;TOP2A|0.481675;SNW1|0.473175;STAT3|0.4995;SLC2A1|5.081875;TXNIP|2.0553;IPO8|0.443925;PLEK|2.071125;NDE1|0.446425;S100A11|2.531875;NDRG1|10.0073;VTN|2.01125;RBBP7|0.218475;ARHGDIA|3.8215;ACTB|2.1999;H3F3A|0.20985;MYH10|5.579275;TTK|0.488375;SOS2|11.48525;ALB|0.23405;PLK1|0.415475;SOD2|2.175425 [illegible]	cellular response to stimulus	GO:0051716	1.30E-08	8.35E-07	59	3
2	HSPA8|0.3721;ACTG1|2.1999;HSP90AB1|0.458775;LCP1|2.170475;HSPD1|2.88995;VIM|2.241075;PABPC1|0.3285;ASPH|2.59965;KRT1|0.4198;NSF|2.017425;DNMT1|0.172;YWHAG|2.359475;ANXA5|3.637725;SRRT|0.39875;GSN|2.4555;AKR1B1|2.235325;PRDX5|3.0143;BAZ1B|0.460625;PSME3|0.401575;LAMTOR2|7.841025;SQSTM1|6.007525;ATG7|2.120975;PCNA|0.404975;MAPK14|0.483325;TFRC|0.403125;RHOG|2.449775;TOP2A|0.481675;SNW1|0.473175;STAT3|0.4995;TXNIP|2.0553;PLEK|2.071125;HBA1|2.13735;NDRG1|10.0073;VTN|2.01125;ARHGDIA|3.8215;ACTB|2.1999;H3F3A|0.20985;TTK|0.488375;SOS2|11.48525;CDC123|0.29925;PLK1|0.415475;GCH1|4.01735;	positive regulation of biological process	GO:0048518	1.50E-08	9.40E-07	44	2
4	HSPA8|0.3721;LCP1|2.170475;ENO1|2.1939;VIM|2.241075;ASPH|2.59965;DNMT1|0.172;VDAC3|2.015475;YWHAG|2.359475;ANXA5|3.637725;GSN|2.4555;SQSTM1|6.007525;ATG7|2.120975;MAPK14|0.483325;TFRC|0.403125;RHOG|2.449775;SNW1|0.473175;VAT1|2.702925;PLEK|2.071125;VTN|2.01125;RBBP7|0.218475;ARHGDIA|3.8215;H3F3A|0.20985;MYH10|5.579275;TTK|0.488375;PLK1|0.	regulation of cellular component organization	GO:0051128	1.58E-08	9.66E-07	26	4
6,8,9,10	DNMT1|0.172;HIST1H4A|0.23835;HIST1H3A|0.455625;H3F3A|0.20985	DNA methylation on cytosine	GO:0032776	1.69E-08	1.01E-06	4	10
4	HSPA8|0.3721;ENO1|2.1939;SARS|2.2366;YARS|2.288275;ATP6V1B2|2.0575;GMPS|0.496875;MTHFD1L|6.667175;CKB|0.486625;NARS|2.162;ALDH1L2|2.148;CNDP2|2.0938;AKR1B1|2.235325;SLC25A1|2.1121;ERO1L|2.370575;PRDX5|3.0143;ESD|2.155525;ISYNA1|0.49065;ALDOC|2.04725;PSME3|0.401575;RRM2|0.21675;ATG7|2.120975;MAPK14|0.483325;STAT3|0.4995;SLC2A1|5.081875;PLEK|2.071125;HBA1|2.13735;ASL|2.252975;ALB|0.23405;G	small molecule metabolic process	GO:0044281	2.65E-08	1.55E-06	30	4
5	ENO1|2.1939;SARS|2.2366;YARS|2.288275;GMPS|0.496875;MTHFD1L|6.667175;CKB|0.486625;NARS|2.162;ALDH1L2|2.148;CNDP2|2.0938;ERO1L|2.370575;ALDOC|2.04725;PSME3|0.401575;ATG7|2.120975;MAPK14|0.483325;STAT3|0.4995;SLC2A1|5.081875;ASL|2.252975;ALB|0.23405;GCH1|4.01735;PLP1|3.936	oxoacid metabolic process	GO:0043436	3.40E-08	1.95E-06	20	5
4	HSP90AB1|0.458775;RPL3|3.30745;ASPH|2.59965;ATP6V1B2|2.0575;MCM2|0.491625;DNMT1|0.172;KPNA2|0.186675;SRRT|0.39875;GSN|2.4555;CNDP2|2.0938;AKR1B1|2.235325;ERO1L|2.370575;PRDX5|3.0143;LAMTOR2|7.841025;SQSTM1|6.007525;ATG7|2.120975;MAPK14|0.483325;TFRC|0.403125;RHOG|2.449775;SNW1|0.473175;STAT3|0.4995;NDRG1|10.0073;VTN|2.01125;ARHGDIA|3.8215;SOS2|11.48525;SOD2|2.175425;PTP4A3|0.3433;IFIT	cellular response to chemical stimulus	GO:0070887	3.57E-08	2.01E-06	28	4
4	HSPA8|0.3721;MCM3|0.35205;MCM2|0.491625;SMC2|0.485025;HIST1H4A|0.23835;YWHAG|2.359475;NCAPG|0.4528;LIG1|0.440425;PSME3|0.401575;RRM2|0.21675;MCMBP|0.4769;PCNA|0.404975;MAPK14|0.483325;TOP2A|0.481675;TUBG1|0.4102;TXNIP|2.0553;NDE1|0.446425;NDRG1|10.0073;MYH10|5.579275;TTK|0.488375;CDC123|0.29925;PLK1|0.415475;CETN3|0.4171;CHAF1B|0.2723	cell cycle	GO:0007049	4.06E-08	2.20E-06	24	4
4	HSPA8|0.3721;LCP1|2.170475;RPL3|3.30745;VIM|2.241075;ASPH|2.59965;RPL4|2.063325;ANXA5|3.637725;GSN|2.4555;RPL27|2.15885;SQSTM1|6.007525;ATG7|2.120975;TOP2A|0.481675;PLEK|2.071125;PLK1|0.415475;CHAF1	cellular component disassembly	GO:0022411	4.07E-08	2.20E-06	15	4
4	ENO1|2.1939;SARS|2.2366;YARS|2.288275;GMPS|0.496875;MTHFD1L|6.667175;CKB|0.486625;NARS|2.162;ALDH1L2|2.148;CNDP2|2.0938;ERO1L|2.370575;ALDOC|2.04725;PSME3|0.401575;ATG7|2.120975;MAPK14|0.483325;STAT3|0.4995;SLC2A1|5.081875;ASL|2.252975;ALB|0.23405;GCH1|4.01735;PLP1|3.936	organic acid metabolic process	GO:0006082	4.43E-08	2.35E-06	20	4
4	MCM3|0.35205;MCM2|0.491625;SMC2|0.485025;YWHAG|2.359475;NCAPG|0.4528;LIG1|0.440425;PSME3|0.401575;RRM2|0.21675;MCMBP|0.4769;PCNA|0.404975;MAPK14|0.483325;TOP2A|0.481675;TUBG1|0.4102;NDE1|0.446425;NDRG1|10.0073;MYH10|5.579275;TTK|0.488375;CDC123|0.29925;PLK1|0.415475;CETN3|0.4171	cell cycle process	GO:0022402	4.65E-08	2.41E-06	20	4

4	ACTG1|2.1999;LCP1|2.170475;HSPD1|2.88995;KRT1|0.4198;ANXA5|3.637725;GSN|2.4555;PABPC4|0.26245;MAPK14|0.483325;HIST1H3A|0.455625;RHOG|2.449775;PLEK|2.071125;VTN|2.01125;ACTB|2.1999;H3F3A|0.20985;MYH10|5.579275;ALB|0.23405	response to wounding	GO:0009611	5.76E-08	2.93E-06	16	4
5	ACTG1|2.1999;KRT1|0.4198;ANXA5|3.637725;PABPC4|0.26245;MAPK14|0.483325;HIST1H3A|0.455625;RHOG|2.449775;PLEK|2.071125;VTN|2.01125;ACTB|2.1999;H3F3A|0.20985;ALB|0.23405	blood coagulation	GO:0007596	7.42E-08	3.71E-06	12	5
5	ACTG1|2.1999;KRT1|0.4198;ANXA5|3.637725;PABPC4|0.26245;MAPK14|0.483325;HIST1H3A|0.455625;RHOG|2.449775;PLEK|2.071125;VTN|2.01125;ACTB|2.1999;H3F3A|0.20985;ALB|0.23405	hemostasis	GO:0007599	8.13E-08	3.95E-06	12	5
4	ACTG1|2.1999;LCP1|2.170475;HSPD1|2.88995;HIST1H2BN|0.481625;RPL3|3.30745;MCM2|0.491625;HIST1H4A|0.23835;VDAC3|2.015475;ANXA5|3.637725;GSN|2.4555;RRM2|0.21675;SQSTM1|6.007525;HIST1H3A|0.455625;RHOG|2.449775;TUBG1|0.4102;SLC2A1|5.081875;PLEK|2.071125;NDE1|0.446425;HBA1|2.13735;RBBP7|0.218475;ACTB|2.1999;H3F3A|0.20985;MYH10|5.579275;PLK1|0.415475;GCH1|4.01735;SOD2|2.175425;TBCA|0.3515;CHAF	cellular component assembly	GO:0022607	8.17E-08	3.95E-06	28	4
4	ACTG1|2.1999;KRT1|0.4198;ANXA5|3.637725;PABPC4|0.26245;MAPK14|0.483325;HIST1H3A|0.455625;RHOG|2.449775;PLEK|2.071125;VTN|2.01125;ACTB|2.1999;H3F3A|0.20985;ALB|0.23405	coagulation	GO:0050817	8.59E-08	4.08E-06	12	4
3	HSPA8|0.3721;HSP90AB1|0.458775;ENO1|2.1939;RPL3|3.30745;PABPC1|0.3285;SARS|2.2366;ASPH|2.59965;YARS|2.288275;MCM3|0.35205;RPL4|2.063325;GMPS|0.496875;MTHFD1L|6.667175;NARS|2.162;MCM2|0.491625;DNMT1|0.172;ALDH1L2|2.148;HIST1H4A|0.23835;YWHAG|2.359475;SRRT|0.39875;CNDP2|2.0938;CBX3|0.485775;GTF2E1|0.300475;AKR1B1|2.235325;PABPC4|0.26245;LIG1|0.440425;SLC25A1|2.1121;PRDX5|3.0143;BAZ1B|0.460625;ISYNA1|0.49065;ALDOC|2.04725;RRM2|0.21675;EIF4H|0.275675;MCMBP|0.4769;RPL27|2.15885;SQSTM1|6.007525;ATG7|2.120975;PCNA|0.404975;MAPK14|0.483325;HIST1H3A|0.455625;BTF3|0.2449;RHOG|2.449775;TOP2A|0.481675;SNW1|0.473175;STAT3|0.4995;SLC2A1|5.081875;TXNIP|2.0553;PLEK|2.071125;S100A11|2.531875;RBBP7|0.218475;H3F3A|0.20985;ASL|2.252975;PLK1|0.415475;GCH1|4.01735;SOD2|2.175425;ZNF706|0.3974;CHA[illegible]	biosynthetic process	GO:0009058	8.90E-08	4.15E-06	57	3
5	HSPD1|2.88995;AKR1B1|2.235325;LIG1|0.440425;ERO1L|2.370575;PRDX5|3.0143;TXNIP|2.0553;HBA1|2.13735;SOD2|2.175425	response to reactive oxygen species	GO:0000302	1.12E-07	5.16E-06	8	5
2	ACTG1|2.1999;HSP90AB1|0.458775;LCP1|2.170475;HSPD1|2.88995;KRT1|0.4198;HIST1H4A|0.23835;ANXA5|3.637725;GSN|2.4555;LIG1|0.440425;PSME3|0.401575;SQSTM1|6.007525;ATG7|2.120975;MAPK14|0.483325;HIST1H3A|0.455625;TFRC|0.403125;TOP2A|0.481675;TXNIP|2.0553;PLEK|2.071125;NDRG1|10.0073;IK|0.480125;VTN|2.01125;ACTB|2.1999;SOS2|11.48525;GCH1|4.01735;HEATR9|2.53905;IFITM2|5.68315;CHAF1B|0.2723	immune system process	GO:0002376	1.28E-07	5.67E-06	27	2
4	ACTG1|2.1999;KRT1|0.4198;ANXA5|3.637725;PABPC4|0.26245;ATG7|2.120975;MAPK14|0.483325;HIST1H3A|0.455625;RHOG|2.449775;PLEK|2.071125;VTN|2.01125;ACTB|2.1999;H3F3A|0.20985;ALB|0.23405	regulation of body fluid levels	GO:0050878	1.28E-07	5.67E-06	13	4
7,8	HIST1H4A|0.23835;RRM2|0.21675;SQSTM1|6.007525;HIST1H3A|0.455625;HBA1|2.13735;GCH1|4.01735	protein heterooligomerization	GO:0051291	1.30E-07	5.67E-06	6	8
6	HSPA8|0.3721;ACTG1|2.1999;HSP90AB1|0.458775;VIM|2.241075;YWHAG|2.359475;SRRT|0.39875;GSN|2.4555;ATG7|2.120975;RHOG|2.449775;SNW1|0.473175;STAT3|0.4995;NCDN|0.4114;NDE1|0.446425;NDRG1|10.0073;VTN|2.01125;ARHGDIA|3.8215;ACTB|2.1999;MYH10|5.579275;SOS2|11.48525;PLP1|3.936	neurogenesis	GO:0022008	1.32E-07	5.67E-06	20	6
6	HIST1H2BN|0.481625;MCM2|0.491625;HIST1H4A|0.23835;BAZ1B|0.460625;HIST1H3A|0.455625;RBBP7|0.218475;H3F3A|0.20985;CHAF1B|0.2723	chromatin assembly or disassembly	GO:0006333	1.35E-07	5.71E-06	8	6
5,6	ACTG1|2.1999;KRT1|0.4198;AKR1B1|2.235325;PRDX5|3.0143;TFRC|0.403125;NCDN|0.4114;ACTB|2.1999;ALB|0.	tissue homeostasis	GO:0001894	1.40E-07	5.78E-06	8	6
4	SARS|2.2366;YARS|2.288275;GMPS|0.496875;MTHFD1L|6.667175;CKB|0.486625;NARS|2.162;ALDH1L2|2.148;CNDP2|2.0938;ERO1L|2.370575;PSME3|0.401575;ATG7|2.120975;ASL|2.252975;GCH1|4.01735	cellular amino acid metabolic process	GO:0006520	1.41E-07	5.78E-06	13	4
5	HSPA8|0.3721;ACTG1|2.1999;HSP90AB1|0.458775;VIM|2.241075;HIST1H4A|0.23835;YWHAG|2.359475;SRRT|0.39875;GSN|2.4555;ERO1L|2.370575;ALDOC|2.04725;SQSTM1|6.007525;ATG7|2.120975;PCNA|0.404975;MAPK14|0.483325;TFRC|0.403125;RHOG|2.449775;TOP2A|0.481675;SNW1|0.473175;STAT3|0.4995;NCDN|0.4114;TXNIP|2.0553;PLEK|2.071125;NDE1|0.446425;NDRG1|10.0073;VTN|2.01125;ARHGDIA|3.8215;ACTB|2.1999;MYH10|5.579275;SOS2|11.48525;HEATR9|2.53905;ZNF706|0.3974;	cell differentiation	GO:0030154	1.47E-07	5.95E-06	32	5

4,5	ACTG1|2.1999;LCP1|2.170475;VIM|2.241075;KRT9|0.268525;SMC2|0.485025;DNMT1|0.172;HIST1H4A|0.23835;VDAC3|2.015475;YWHAG|2.359475;ANXA5|3.637725;GSN|2.4555;NCAPG|0.4528;LIG1|0.440425;BAZ1B|0.460625;MCMBP|0.4769;SQSTM1|6.007525;ATG7|2.120975;PCNA|0.404975;RHOG|2.449775;TOP2A|0.481675;SNW1|0.473175;STAT3|0.4995;TUBG1|0.4102;VAT1|2.702925;PLEK|2.071125;NDE1|0.446425;MYH10|5.579275;TTK|0.488375;PLK1|0.415475;CETN3|0.4171;CHAF1B|0.2723	single-organism organelle organization	GO:1902589	1.53E-07	6.11E-06	31	5
4	HSPA8|0.3721;HSP90AB1|0.458775;ENO1|2.1939;RPL3|3.30745;PABPC1|0.3285;SARS|2.2366;ASPH|2.59965;YARS|2.288275;MCM3|0.35205;RPL4|2.063325;GMPS|0.496875;MTHFD1L|6.667175;NARS|2.162;MCM2|0.491625;DNMT1|0.172;HIST1H4A|0.23835;YWHAG|2.359475;SRRT|0.39875;CNDP2|2.0938;CBX3|0.485775;GTF2E1|0.300475;AKR1B1|2.235325;PABPC4|0.26245;LIG1|0.440425;SLC25A1|2.1121;PRDX5|3.0143;BAZ1B|0.460625;ISYNA1|0.49065;RRM2|0.21675;EIF4H|0.275675;MCMBP|0.4769;RPL27|2.15885;SQSTM1|6.007525;ATG7|2.120975;PCNA|0.404975;MAPK14|0.483325;HIST1H3A|0.455625;BTF3|0.2449;RHOG|2.449775;TOP2A|0.481675;SNW1|0.473175;STAT3|0.4995;SLC2A1|5.081875;TXNIP|2.0553;PLEK|2.071125;S100A11|2.531875;RBBP7|0.218475;H3F3A|0.20985;ASL|2.252975;PLK1|0.415475;GCH1|4.01735;SOD2|2.17 [illegible]	cellular biosynthetic process	GO:0044249	1.71E-07	6.71E-06	55	4
4	HSPD1|2.88995;ASPH|2.59965;ATP6V1B2|2.0575;DNMT1|0.172;GSN|2.4555;AKR1B1|2.235325;LIG1|0.440425;ERO1L|2.370575;PRDX5|3.0143;LAMTOR2|7.841025;MAPK14|0.483325;TFRC|0.403125;SNW1|0.473175;STAT3|0.4995;TXNIP|2.0553;HBA1|2.13735;GCH1|4.01735;SOD2|2.	response to oxygen-containing compound	GO:1901700	2.09E-07	8.12E-06	18	4
4	HSP90AB1|0.458775;HSPD1|2.88995;VIM|2.241075;YARS|2.288275;YWHAG|2.359475;ANXA5|3.637725;GSN|2.4555;ERO1L|2.370575;PRDX5|3.0143;PSME3|0.401575;SQSTM1|6.007525;ATG7|2.120975;MAPK14|0.483325;TOP2A|0.481675;SNW1|0.473175;STAT3|0.4995;TXNIP|2.0553;HBA1|2.13735;ARHGDIA|3.8215;SOS2|11.48525;ALB|0.23405;PLK1|0.415475;SOD2|2.175425	cell death	GO:0008219	2.44E-07	9.32E-06	23	4
3	HSP90AB1|0.458775;HSPD1|2.88995;VIM|2.241075;YARS|2.288275;YWHAG|2.359475;ANXA5|3.637725;GSN|2.4555;ERO1L|2.370575;PRDX5|3.0143;PSME3|0.401575;SQSTM1|6.007525;ATG7|2.120975;MAPK14|0.483325;TOP2A|0.481675;SNW1|0.473175;STAT3|0.4995;TXNIP|2.0553;HBA1|2.13735;ARHGDIA|3.8215;SOS2|11.48525;ALB|0.23405;PLK1|0.415475;SOD2|2.175425	death	GO:0016265	2.51E-07	9.45E-06	23	3
3	HSPD1|2.88995;ASPH|2.59965;DNMT1|0.172;SRRT|0.39875;AKR1B1|2.235325;PCNA|0.404975;MAPK14|0.483325;TFRC|0.403125;RHOG|2.449775;STAT3|0.4995;TXNIP|2.0553;NDE1|0.446425;S100A11|2.531875;NDRG1|10.0073;RBBP7|0.218475;MYH10|5.579275;TTK|0.488375;SOS2|11.48525;CDC123|0.29925;PLK1|0.415475;SOD2|2.175425	cell proliferation	GO:0008283	2.70E-07	1.00E-05	21	3
3	HSPA8|0.3721;HSP90AB1|0.458775;LCP1|2.170475;HSPD1|2.88995;PABPC1|0.3285;ASPH|2.59965;NSF|2.017425;DNMT1|0.172;YWHAG|2.359475;ANXA5|3.637725;SRRT|0.39875;GSN|2.4555;AKR1B1|2.235325;BAZ1B|0.460625;PSME3|0.401575;LAMTOR2|7.841025;SQSTM1|6.007525;ATG7|2.120975;PCNA|0.404975;MAPK14|0.483325;RHOG|2.449775;TOP2A|0.481675;SNW1|0.473175;STAT3|0.4995;TXNIP|2.0553;PLEK|2.071125;HBA1|2.13735;NDRG1|10.0073;VTN|2.01125;ARHGDIA|3.8215;H3F3A|0.20985;TTK|0.488375;SOS2|11.48525;CDC123|0.29925;PLK1	positive regulation of cellular process	GO:0048522	2.91E-07	1.07E-05	36	3
6,7	HIST1H2BN|0.481625;MCM2|0.491625;HIST1H4A|0.23835;HIST1H3A|0.455625;RBBP7|0.218475;H3F3A|0.20985;CHAF1B|0.2723	nucleosome assembly	GO:0006334	3.02E-07	1.10E-05	7	7
4	HSPA8|0.3721;ENO1|2.1939;RPL3|3.30745;PABPC1|0.3285;SARS|2.2366;ASPH|2.59965;YARS|2.288275;MCM3|0.35205;RPL4|2.063325;GMPS|0.496875;MTHFD1L|6.667175;NARS|2.162;MCM2|0.491625;DNMT1|0.172;HIST1H4A|0.23835;YWHAG|2.359475;SRRT|0.39875;CNDP2|2.0938;CBX3|0.485775;GTF2E1|0.300475;AKR1B1|2.235325;PABPC4|0.26245;LIG1|0.440425;SLC25A1|2.1121;PRDX5|3.0143;BAZ1B|0.460625;ISYNA1|0.49065;ALDOC|2.04725;RRM2|0.21675;EIF4H|0.275675;MCMBP|0.4769;RPL27|2.15885;SQSTM1|6.007525;ATG7|2.120975;PCNA|0.404975;MAPK14|0.483325;HIST1H3A|0.455625;BTF3|0.2449;RHOG|2.449775;TOP2A|0.481675;SNW1|0.473175;STAT3|0.4995;SLC2A1|5.081875;TXNIP|2.0553;PLEK|2.071125;S100A11|2.531875;RBBP7|0.218475;H3F3A|0.20985;ASL|2.252975;PLK1|0.415475;GCH1|4.01735;SOD2|2.175425; [illegible]	organic substance biosynthetic process	GO:1901576	3.14E-07	1.12E-05	55	4
4	HSPA8|0.3721;ENO1|2.1939;RPL3|3.30745;PABPC1|0.3285;SARS|2.2366;YARS|2.288275;ATP6V1B2|2.0575;RPL4|2.063325;GMPS|0.496875;MTHFD1L|6.667175;CKB|0.486625;NARS|2.162;ALDH1L2|2.148;CNDP2|2.0938;AKR1B1|2.235325;PABPC4|0.26245;ERO1L|2.370575;PRDX5|3.0143;ALDOC|2.04725;PSME3|0.401575;EIF4H|0.275675;RPL27|2.15885;ATG7|2.120975;STAT3|0.4995;DNPEP|2.077575;ASL|2.252975;GCH1|4.01735;ZNF706|0.3974	organonitrogen compound metabolic process	GO:1901564	3.20E-07	1.13E-05	28	4

6	HSP90AB1|0.458775;HSPD1|2.88995;VIM|2.241075;YARS|2.288275;YWHAG|2.359475;ANXA5|3.637725;GSN|2.4555;ERO1L|2.370575;PRDX5|3.0143;PSME3|0.401575;SQSTM1|6.007525;ATG7|2.120975;MAPK14|0.483325;TOP2A|0.481675;SNW1|0.473175;STAT3|0.4995;TXNIP|2.0553;ARHGDIA|3.8215;SOS2|11.48525;ALB|0.23405;PLK1|0.4	apoptotic process	GO:0006915	3.40E-07	1.18E-05	22	6
4,5	ACTG1|2.1999;KRT1|0.4198;AKR1B1|2.235325;PRDX5|3.0143;TFRC|0.403125;STAT3|0.4995;NCDN|0.4114;ACTB|2.1999;ALB|0.23405	multicellular organismal homeostasis	GO:0048871	3.60E-07	1.24E-05	9	5
5	HSP90AB1|0.458775;HSPD1|2.88995;VIM|2.241075;YARS|2.288275;YWHAG|2.359475;ANXA5|3.637725;GSN|2.4555;ERO1L|2.370575;PRDX5|3.0143;PSME3|0.401575;SQSTM1|6.007525;ATG7|2.120975;MAPK14|0.483325;TOP2A|0.481675;SNW1|0.473175;STAT3|0.4995;TXNIP|2.0553;ARHGDIA|3.8215;SOS2|11.48525;ALB|0.23405;PLK1|0.4	programmed cell death	GO:0012501	4.12E-07	1.40E-05	22	5
4	ACTG1|2.1999;ASPH|2.59965;KRT1|0.4198;ATP6V1B2|2.0575;CKB|0.486625;HIST1H4A|0.23835;AKR1B1|2.235325;LIG1|0.440425;ERO1L|2.370575;PRDX5|3.0143;PCNA|0.404975;MAPK14|0.483325;TFRC|0.403125;STAT3|0.4995;NCDN|0.4114;ACTB|2.1999;SOS2|11.48525;ALB|0.23	homeostatic process	GO:0042592	5.00E-07	1.68E-05	19	4
3	ACTG1|2.1999;HSP90AB1|0.458775;HSPD1|2.88995;KRT1|0.4198;YWHAG|2.359475;ANXA5|3.637725;GSN|2.4555;AKR1B1|2.235325;PSME3|0.401575;LAMTOR2|7.841025;SQSTM1|6.007525;ATG7|2.120975;PCNA|0.404975;MAPK14|0.483325;SNW1|0.473175;STAT3|0.4995;PLEK|2.071125;VTN|2.01125;ACTB|2.1999;TTK|0.488375;SOS2|1	positive regulation of response to stimulus	GO:0048584	5.45E-07	1.81E-05	22	3
4,5,6	HSPA8|0.3721;HSP90AB1|0.458775;HSPD1|2.88995;VIM|2.241075;PABPC1|0.3285;ASPH|2.59965;NSF|2.017425;DNMT1|0.172;GSN|2.4555;PRDX5|3.0143;BAZ1B|0.460625;PSME3|0.401575;LAMTOR2|7.841025;SQSTM1|6.007525;ATG7|2.120975;PCNA|0.404975;MAPK14|0.483325;RHOG|2.449775;TOP2A|0.481675;SNW1|0.473175;STAT3|0.4995;PLEK|2.071125;VTN|2.01125;TTK|0.488375;PLK1|0	positive regulation of macromolecule metabolic process	GO:0010604	5.67E-07	1.86E-05	26	6
4	HSPA8|0.3721;ACTG1|2.1999;HSP90AB1|0.458775;VIM|2.241075;HIST1H4A|0.23835;VDAC3|2.015475;YWHAG|2.359475;SRRT|0.39875;GSN|2.4555;ERO1L|2.370575;ALDOC|2.04725;SQSTM1|6.007525;ATG7|2.120975;PCNA|0.404975;MAPK14|0.483325;TFRC|0.403125;RHOG|2.449775;TOP2A|0.481675;SNW1|0.473175;STAT3|0.4995;NCDN|0.4114;TXNIP|2.0553;PLEK|2.071125;NDE1|0.446425;NDRG1|10.0073;VTN|2.01125;ARHGDIA|3.8215;ACTB|2.1999;MYH10|5.579275;SOS2|11.48525;HEATR9|2.53905	cellular developmental process	GO:0048869	5.86E-07	1.90E-05	33	4
3	ACTG1|2.1999;LCP1|2.170475;HSPD1|2.88995;HIST1H2BN|0.481625;RPL3|3.30745;MCM2|0.491625;HIST1H4A|0.23835;VDAC3|2.015475;ANXA5|3.637725;GSN|2.4555;RRM2|0.21675;SQSTM1|6.007525;HIST1H3A|0.455625;RHOG|2.449775;TUBG1|0.4102;SLC2A1|5.081875;PLEK|2.071125;NDE1|0.446425;HBA1|2.13735;RBBP7|0.218475;ACTB|2.1999;H3F3A|0.20985;MYH10|5.579275;PLK1|0.415475;GCH1|4.01735;SOD2|2.175425;TBCA|0.3515;CHAF	cellular component biogenesis	GO:0044085	6.11E-07		28	3
3	HSPA8|0.3721;HSP90AB1|0.458775;ENO1|2.1939;PABPC1|0.3285;DNMT1|0.172;HIST1H4A|0.23835;YWHAG|2.359475;ANXA5|3.637725;SRRT|0.39875;CBX3|0.485775;PRDX5|3.0143;PSME3|0.401575;ATG7|2.120975;HIST1H3A|0.455625;SNW1|0.473175;STAT3|0.4995;TXNIP|2.0553;PLEK|2.071125;S100A11|2.531875;VTN|2.01125;RBBP7|0.218475;H3F3A|0.20985;TTK|0.488375;PLK1|0.415475;ZN	negative regulation of metabolic process	GO:0009892	7.28E-07	1.96E-05	25	3
6	HIST1H2BN|0.481625;MCM2|0.491625;HIST1H4A|0.23835;HIST1H3A|0.455625;RBBP7|0.218475;H3F3A|0.20985;CHAF1B|0.2723	chromatin assembly	GO:0031497	7.76E-07	2.30E-05	7	6
6	HIST1H2BN|0.481625;MCM2|0.491625;HIST1H4A|0.23835;HIST1H3A|0.455625;RBBP7|0.218475;H3F3A|0.20985;CHAF1B|0.2723	nucleosome organization	GO:0034728	8.35E-07	2.43E-05	7	6
3	HSPA8|0.3721;HSP90AB1|0.458775;ENO1|2.1939;HSPD1|2.88995;RPL3|3.30745;PABPC1|0.3285;SARS|2.2366;ASPH|2.59965;YARS|2.288275;ATP6V1B2|2.0575;MCM3|0.35205;RPL4|2.063325;GMPS|0.496875;MTHFD1L|6.667175;CKB|0.486625;NARS|2.162;MCM2|0.491625;DNMT1|0.172;ALDH1L2|2.148;HIST1H4A|0.23835;KPNA2|0.186675;YWHAG|2.359475;SRRT|0.39875;CNDP2|2.0938;CBX3|0.485775;GTF2E1|0.300475;AKR1B1|2.235325;PABPC4|0.26245;LIG1|0.440425;G3BP1|0.3904;ERO1L|2.370575;PRDX5|3.0143;BAZ1B|0.460625;ALDOC|2.04725;PSME3|0.401575;RRM2|0.21675;EIF4H|0.275675;MCMBP|0.4769;RPL27|2.15885;SQSTM1|6.007525;ATG7|2.120975;PCNA|0.404975;MAPK14|0.483325;HIST1H3A|0.455625;BTF3|0.2449;RHOG|2.449775;TOP2A|0.481675;SNW1|0.473175;STAT3|0.4995;TXNIP|2.0553;S100A11|2.531875;RBBP7|0.218475;DNPEP|2.077575;H3F3A|0.20985;ASL|2.252975;PLK1|0.415475;GCH1|4.01735;SOD2|2.175425;ZNF706|0	nitrogen compound metabolic process	GO:0006807	8.86E-07	2.58E-05	60	3

3	HSPA8|0.3721;ACTG1|2.1999;HSP90AB1|0.458775;HSPD1|2.88995;ASPH|2.59965;KRT1|0.4198;YWHAG|2.359475;ANXA5|3.637725;GSN|2.4555;AKR1B1|2.235325;G3BP1|0.3904;BAZ1B|0.460625;PSME3|0.401575;LAMTOR2|7.841025;SQSTM1|6.007525;ATG7|2.120975;PCNA|0.404975;MAPK14|0.483325;HIST1H3A|0.455625;RHOG|2.449775;SNW1|0.473175;STAT3|0.4995;PLEK|2.071125;VTN|2.01125;ARHGDIA|3.8215;ACTB|2.1999;TTK|0.488375;SOS2|11.48525;SOD2|2.175425;PTP4A3|0.3433;CHAF1B|0.	regulation of response to stimulus	GO:0048583	9.51E-07	2.71E-05	31	3
4	HSPA8|0.3721;HSP90AB1|0.458775;ENO1|2.1939;HSPD1|2.88995;RPL3|3.30745;PABPC1|0.3285;SARS|2.2366;ASPH|2.59965;YARS|2.288275;ATP6V1B2|2.0575;MCM3|0.35205;RPL4|2.063325;GMPS|0.496875;MTHFD1L|6.667175;CKB|0.486625;NARS|2.162;MCM2|0.491625;DNMT1|0.172;ALDH1L2|2.148;HIST1H4A|0.23835;KPNA2|0.186675;YWHAG|2.359475;SRRT|0.39875;CNDP2|2.0938;CBX3|0.485775;GTF2E1|0.300475;PABPC4|0.26245;LIG1|0.440425;G3BP1|0.3904;PRDX5|3.0143;BAZ1B|0.460625;ALDOC|2.04725;PSME3|0.401575;RRM2|0.21675;EIF4H|0.275675;MCMBP|0.4769;RPL27|2.15885;SQSTM1|6.007525;ATG7|2.120975;PCNA|0.404975;MAPK14|0.483325;HIST1H3A|0.455625;BTF3|0.2449;RHOG|2.449775;TOP2A|0.481675;SNW1|0.473175;STAT3|0.4995;TXNIP|2.0553;S100A11|2.531875;RBBP7|0.218475;DNPEP|2.077575;H3F3A|0.20985;ASL|2.252975;PLK1|0.415475;GCH1|4.01735;SO[illegible]	cellular nitrogen compound metabolic process	GO:0034641	1.11E-06	2.88E-05	58	4
5	HSPA8|0.3721;ACTG1|2.1999;HSP90AB1|0.458775;VIM|2.241075;MTHFD1L|6.667175;CKB|0.486625;YWHAG|2.359475;SRRT|0.39875;GSN|2.4555;ATG7|2.120975;RHOG|2.449775;SNW1|0.473175;STAT3|0.4995;NCDN|0.4114;NDE1|0.446425;NDRG1|10.0073;VTN|2.01125;ARHGDIA|3.8215;ACTB|2.1999;H3F3A|0.20985;MYH10|5.579275;S	nervous system development	GO:0007399	1.25E-06	3.31E-05	23	5
6,7	MCM3|0.35205;MCM2|0.491625;LIG1|0.440425;RRM2|0.21675;MCMBP|0.4769;PCNA|0.404975;TOP2A|0.481675;S100A11|2.531875;RBBP7|0.218475;CHAF1B|0.2723	DNA replication	GO:0006260	1.26E-06	3.68E-05	10	7
4,5,6	HSPA8|0.3721;HSP90AB1|0.458775;ENO1|2.1939;PABPC1|0.3285;DNMT1|0.172;HIST1H4A|0.23835;YWHAG|2.359475;CBX3|0.485775;PRDX5|3.0143;PSME3|0.401575;ATG7|2.120975;HIST1H3A|0.455625;SNW1|0.473175;STAT3|0.4995;TXNIP|2.0553;PLEK|2.071125;S100A11|2.531875;VTN|2.01125;RBBP7|0.218475;H3F3A|0.20985;TTK|0.488375;PLK1|0.415475;ZNF706|0.3974	negative regulation of cellular metabolic process	GO:0031324	1.29E-06	3.68E-05	23	6
4,5,6	HSPA8|0.3721;HSP90AB1|0.458775;ENO1|2.1939;PABPC1|0.3285;DNMT1|0.172;HIST1H4A|0.23835;YWHAG|2.359475;SRRT|0.39875;CBX3|0.485775;PRDX5|3.0143;PSME3|0.401575;ATG7|2.120975;HIST1H3A|0.455625;SNW1|0.473175;STAT3|0.4995;TXNIP|2.0553;S100A11|2.531875;VTN|2.01125;RBBP7|0.218475;H3F3A|0.20985;TTK|0.488375;PLK1|0.415475;ZNF706|0.3974	negative regulation of macromolecule metabolic process	GO:0010605	1.34E-06	3.73E-05	23	6
3	HSPA8|0.3721;HSP90AB1|0.458775;HSPD1|2.88995;VIM|2.241075;PABPC1|0.3285;ASPH|2.59965;NSF|2.017425;DNMT1|0.172;ANXA5|3.637725;GSN|2.4555;PRDX5|3.0143;BAZ1B|0.460625;PSME3|0.401575;LAMTOR2|7.841025;SQSTM1|6.007525;ATG7|2.120975;PCNA|0.404975;MAPK14|0.483325;RHOG|2.449775;TOP2A|0.481675;SNW1|0.473175;STAT3|0.4995;PLEK|2.071125;VTN|2.01125;ARHGDIA|3.8215;TTK|0.488375;SOS2|11.48525;PLK1|0.415475;GCH1|4.01735;CHAF1B|0.2723;PLP1|3.936	positive regulation of metabolic process	GO:0009893	1.43E-06	3.83E-05	31	3
4	SMC2|0.485025;NCAPG|0.4528;LIG1|0.440425;MCMBP|0.4769;TOP2A|0.481675;TXNIP|2.0553;NDE1|0.446425;MYH10|5.579275;TTK|0.488375;CDC123|0.29925;PLK1|0.415475;CETN3|0.4171	cell division	GO:0051301	1.51E-06	4.07E-05	12	4
5	ACTG1|2.1999;ASPH|2.59965;ANXA5|3.637725;GSN|2.4555;PCNA|0.404975;TFRC|0.403125;TXNIP|2.0553;NDRG1|10.0073	response to metal ion	GO:0010038	1.66E-06	4.23E-05	8	5
4	HSPA8|0.3721;ACTG1|2.1999;HSP90AB1|0.458775;LCP1|2.170475;HSPD1|2.88995;VIM|2.241075;ASPH|2.59965;YARS|2.288275;ATP6V1B2|2.0575;NSF|2.017425;HIST1H4A|0.23835;KPNA2|0.186675;YWHAG|2.359475;ANXA5|3.637725;GSN|2.4555;AKR1B1|2.235325;G3BP1|0.3904;ERO1L|2.370575;BAZ1B|0.460625;PSME3|0.401575;LAMTOR2|7.841025;SQSTM1|6.007525;ATG7|2.120975;MAPK14|0.483325;HIST1H3A|0.455625;RHOG|2.449775;SNW1|0.473175;STAT3|0.4995;NCDN|0.4114;SLC2A1|5.081875;TXNIP|2.0553;IPO8|0.443925;PLEK|2.071125;NDE1|0.446425;S100A11|2.531875;NDRG1|10.0073;IK|0.480125;VTN|2.01125;ARHGDIA|3.8215;ACTB|2.1999;H3F3A|0.20985;MYH10|5.579275;TTK|0.488375;SOS2|11.48525;ALB|0.23405;PLK1|0.415475;SOD2|2.175425;PTP4A3|0.3433;I[illegible]	cell communication	GO:0007154	1.75E-06	4.61E-05	51	4
6	MCM3|0.35205;MCM2|0.491625;YWHAG|2.359475;PSME3|0.401575;RRM2|0.21675;PCNA|0.404975;TUBG1|0.4102;NDE1|0.446425;TTK|0.488375;PLK1|0.415475	mitotic cell cycle phase transition	GO:0044772	2.25E-06	4.82E-05	10	6
7	HIST1H4A|0.23835;HIST1H3A|0.455625;CHAF1B|0.2723	DNA replication-dependent nucleosome	GO:0034723	2.33E-06	6.14E-05	3	7
7,8	HIST1H4A|0.23835;HIST1H3A|0.455625;CHAF1B|0.2723	DNA replication-dependent nucleosome	GO:0006335	2.33E-06	6.17E-05	3	8
8,9	HSPA8|0.3721;HSPD1|2.88995;ERO1L|2.370575	chaperone mediated protein folding requiring	GO:0051085	2.33E-06	6.17E-05	3	9

4	HSPA8|0.3721;ACTG1|2.1999;RPL3|3.30745;RPL4|2.063325;NSF|2.017425;YWHAG|2.359475;GSN|2.4555;RPL27|2.15885;ATG7|2.120975;RHOG|2.449775;STAT3|0.4995;VAT1|2.702925;NDRG1|10.0073;ACTB|2.1999;MYH10|5.579275;PLK1|0.415475	membrane organization	GO:0061024	2.55E-06	6.17E-05	16	4
5,6	HSP90AB1|0.458775;RPL3|3.30745;RPL4|2.063325;GSN|2.4555;RPL27|2.15885;ATG7|2.120975;TOP2A|0.481675;SNW1|0.473175;IFITM2|5.68315	viral life cycle	GO:0019058	2.89E-06	6.68E-05	9	6
4,5	HSP90AB1|0.458775;VIM|2.241075;KRT1|0.4198;KRT9|0.268525;MTHFD1L|6.667175;CKB|0.486625;HIST1H4A|0.23835;AKR1B1|2.235325;BAZ1B|0.460625;ATG7|2.120975;PCNA|0.404975;MAPK14|0.483325;TFRC|0.403125;TOP2A|0.481675;SNW1|0.473175;STAT3|0.4995;TXNIP|2.0553;PLEK|2.071125;NDE1|0.446425;ACTB|2.1999;H3F3A|0.20985;MYH10|5.579275;SOS2|11.48525;HEATR9|2.5	organ development	GO:0048513	2.90E-06	7.46E-05	25	5
5	MCM3|0.35205;MCM2|0.491625;YWHAG|2.359475;PSME3|0.401575;RRM2|0.21675;PCNA|0.404975;TUBG1|0.4102;NDE1|0.446425;TTK|0.488375;PLK1|0.415475	cell cycle phase transition	GO:0044770	3.11E-06	7.46E-05	10	5
5,6	HSPD1|2.88995;MCM3|0.35205;MCM2|0.491625;DNMT1|0.172;HIST1H4A|0.23835;KPNA2|0.186675;LIG1|0.440425;BAZ1B|0.460625;RRM2|0.21675;MCMBP|0.4769;PCNA|0.404975;HIST1H3A|0.455625;TOP2A|0.481675;S100A11|2.531875;RBBP7|0.218475;H3F3A|0.20985;CHAF1B|0.2	DNA metabolic process	GO:0006259	3.33E-06	7.93E-05	17	6
4	HSPA8|0.3721;ENO1|2.1939;HSPD1|2.88995;RPL3|3.30745;PABPC1|0.3285;SARS|2.2366;ASPH|2.59965;YARS|2.288275;ATP6V1B2|2.0575;MCM3|0.35205;RPL4|2.063325;GMPS|0.496875;MTHFD1L|6.667175;NARS|2.162;MCM2|0.491625;DNMT1|0.172;ALDH1L2|2.148;HIST1H4A|0.23835;KPNA2|0.186675;YWHAG|2.359475;SRRT|0.39875;CBX3|0.485775;GTF2E1|0.300475;AKR1B1|2.235325;PABPC4|0.26245;LIG1|0.440425;G3BP1|0.3904;ERO1L|2.370575;PRDX5|3.0143;BAZ1B|0.460625;ALDOC|2.04725;RRM2|0.21675;MCMBP|0.4769;RPL27|2.15885;SQSTM1|6.007525;ATG7|2.120975;PCNA|0.404975;MAPK14|0.483325;HIST1H3A|0.455625;BTF3|0.2449;RHOG|2.449775;TOP2A|0.481675;SNW1|0.473175;STAT3|0.4995;TXNIP|2.0553;S100A11|2.531875;RBBP7|0.218475;H3F3A|0.20985;ALB|0.23405;PLK1|0.415475;GCH1|4.01735;SOD2|2.175425;[illegible]	organic cyclic compound metabolic process	GO:1901360	3.58E-06	8.42E-05	54	4
3	HSPA8|0.3721;HSP90AB1|0.458775;ENO1|2.1939;HSPD1|2.88995;RPL3|3.30745;CCT7|2.06135;PABPC1|0.3285;SARS|2.2366;ASPH|2.59965;YARS|2.288275;ATP6V1B2|2.0575;MCM3|0.35205;RPL4|2.063325;NSF|2.017425;GMPS|0.496875;MTHFD1L|6.667175;CKB|0.486625;NARS|2.162;MCM2|0.491625;HSPA4L|2.13105;DNMT1|0.172;ALDH1L2|2.148;HIST1H4A|0.23835;KPNA2|0.186675;YWHAG|2.359475;ANXA5|3.637725;SRRT|0.39875;GSN|2.4555;CNDP2|2.0938;CBX3|0.485775;GTF2E1|0.300475;AKR1B1|2.235325;PABPC4|0.26245;LIG1|0.440425;SLC25A1|2.1121;G3BP1|0.3904;ERO1L|2.370575;PRDX5|3.0143;BAZ1B|0.460625;ESD|2.155525;ISYNA1|0.49065;ALDOC|2.04725;PSME3|0.401575;RRM2|0.21675;EIF4H|0.275675;LAMTOR2|7.841025;MCMBP|0.4769;RPL27|2.15885;SQSTM1|6.007525;ATG7|2.120975;PCNA|0.404975;MAPK14|0.483325;HIST1H3A|0.455625;BTF3|0.2449;RHOG|2.449775;TOP2A|0.481675;SNW1|0.473175;STAT3|0.4995;SLC2A1|5.081875;TXNIP|2.0553;PLEK|2.071125;S100A11|2.531875;HBA1|2.13735;VTN|2.01125;RBBP7|0.218475;ACTB|2.1999;DNPEP|2.077575;H3F3A|0.20985;TTK|0.488375;ASL|2.252975;ALB|0.23405;PLK1|0.415475;GCH1|4.01735;SOD2|2.175425;PTP4A3|0.3433;TBCA|0.3515;ZNF706|0[illegible]	cellular metabolic process	GO:0044237	3.58E-06	8.87E-05	79	3
4	HSP90AB1|0.458775;LCP1|2.170475;RPL3|3.30745;ASPH|2.59965;ATP6V1B2|2.0575;RPL4|2.063325;NSF|2.017425;KPNA2|0.186675;YWHAG|2.359475;GSN|2.4555;LAMTOR2|7.841025;RPL27|2.15885;SQSTM1|6.007525;ATG7|2.120975;MAPK14|0.483325;BTF3|0.2449;TFRC|0.403125;RHOG|2.449775;STAT3|0.4995;SLC2A1|5.081875;TXNIP|2.0553;IPO8|0.443925;PLEK|2.071125;ARHGDIA|3.8215;	protein localization	GO:0008104	3.62E-06	8.87E-05	25	4
4,5	HSP90AB1|0.458775;HSPD1|2.88995;YWHAG|2.359475;ANXA5|3.637725;GSN|2.4555;PRDX5|3.0143;PSME3|0.401575;SQSTM1|6.007525;ATG7|2.120975;TOP2A|0.481675;STAT3|0.4995;TXNIP|2.0553;HBA1|2.13735;ARHGDIA|3.8215;SOS2|11.48525;ALB|0.23405;PLK1|0.415475;SO	regulation of cell death	GO:0010941	4.52E-06	8.89E-05	18	5

3	HSPA8|0.3721;HSP90AB1|0.458775;ENO1|2.1939;HSPD1|2.88995;RPL3|3.30745;VIM|2.241075;CCT7|2.06135;PABPC1|0.3285;SARS|2.2366;ASPH|2.59965;KRT1|0.4198;YARS|2.288275;ATP6V1B2|2.0575;MCM3|0.35205;RPL4|2.063325;NSF|2.017425;GMPS|0.496875;MTHFD1L|6.667175;CKB|0.486625;NARS|2.162;MCM2|0.491625;HSPA4L|2.13105;DNMT1|0.172;ALDH1L2|2.148;HIST1H4A|0.23835;KPNA2|0.186675;YWHAG|2.359475;SRRT|0.39875;GSN|2.4555;CNDP2|2.0938;CBX3|0.485775;GTF2E1|0.300475;AKR1B1|2.235325;PABPC4|0.26245;LIG1|0.440425;SLC25A1|2.1121;G3BP1|0.3904;ERO1L|2.370575;PRDX5|3.0143;BAZ1B|0.460625;ESD|2.155525;ISYNA1|0.49065;ALDOC|2.04725;PSME3|0.401575;RRM2|0.21675;EIF4H|0.275675;ACAT2|2.108325;LAMTOR2|7.841025;MCMBP|0.4769;RPL27|2.15885;SQSTM1|6.007525;ATG7|2.120975;PCNA|0.404975;MAPK14|0.483325;HIST1H3A|0.455625;BTF3|0.2449;RHOG|2.449775;TOP2A|0.481675;SNW1|0.473175;STAT3|0.4995;SLC2A1|5.081875;TXNIP|2.0553;IPO8|0.443925;PLEK|2.071125;S100A11|2.531875;VTN|2.01125;RBBP7|0.218475;PLBD2|2.666725;ACTB|2.1999;DNPEP|2.077575;H3F3A|0.20985;TTK|0.488375;ASL|2.252975;ALB|0.23405;PLK1|0.415475;GCH1|4.01735;SOD2|2.17[illegible]	organic substance metabolic process	GO:0071704	4.81E-06	1.10E-04	82	3
4	HSPA8|0.3721;HSP90AB1|0.458775;HSPD1|2.88995;KRT1|0.4198;ANXA5|3.637725;GSN|2.4555;PSME3|0.401575;SQSTM1|6.007525;ATG7|2.120975;PCNA|0.404975;MAPK14|0.483325;HIST1H3A|0.455625;PLEK|2.071125;VTN|2.01125;SOD2|2.175425;CHAF1B|0.2723	regulation of response to stress	GO:0080134	5.23E-06	1.16E-04	16	4
8,9	HIST1H4A|0.23835;RRM2|0.21675;HIST1H3A|0.455625	protein heterotetramerization	GO:0051290	5.52E-06	1.25E-04	3	9
7	HSPA8|0.3721;ACTG1|2.1999;HSP90AB1|0.458775;VIM|2.241075;YWHAG|2.359475;SRRT|0.39875;ATG7|2.120975;RHOG|2.449775;SNW1|0.473175;STAT3|0.4995;NCDN|0.4114;NDE1|0.446425;ARHGDIA|3.8215;ACTB|2.1999;MYH10|5.579275;SOS2|11.48525;PLP1|3.936	generation of neurons	GO:0048699	5.66E-06	1.31E-04	17	7
4,5,6	HSPA8|0.3721;HSP90AB1|0.458775;HSPD1|2.88995;PABPC1|0.3285;ASPH|2.59965;NSF|2.017425;DNMT1|0.172;ANXA5|3.637725;GSN|2.4555;BAZ1B|0.460625;PSME3|0.401575;LAMTOR2|7.841025;SQSTM1|6.007525;ATG7|2.120975;PCNA|0.404975;MAPK14|0.483325;RHOG|2.449775;TOP2A|0.481675;SNW1|0.473175;STAT3|0.4995;PLEK|2.071125;VTN|2.01125;TTK|0.488375;PLK1|0.415475;CH	positive regulation of cellular metabolic process	GO:0031325	5.74E-06	1.33E-04	25	6
4	HSPD1|2.88995;KRT1|0.4198;AKR1B1|2.235325;LIG1|0.440425;ERO1L|2.370575;PRDX5|3.0143;TXNIP|2.0553;HBA1|2.13735;SOD2|2.175425	response to oxidative stress	GO:0006979	5.83E-06	1.34E-04	9	4
5	HSP90AB1|0.458775;HSPD1|2.88995;PABPC1|0.3285;ASPH|2.59965;NSF|2.017425;DNMT1|0.172;GSN|2.4555;PSME3|0.401575;LAMTOR2|7.841025;SQSTM1|6.007525;ATG7|2.120975;MAPK14|0.483325;SNW1|0.473175;PLEK|2.071125;VTN|2.01125;TTK|0.488375;PLK1|0.415475	positive regulation of protein metabolic process	GO:0051247	6.00E-06	1.35E-04	17	5
6,7	ACTG1|2.1999;HIST1H4A|0.23835;YWHAG|2.359475;G3BP1|0.3904;SQSTM1|6.007525;MAPK14|0.483325;HIST1H3A|0.455625;RHOG|2.449775;NDE1|0.446425;ARHGDIA|3.8215;ACTB|2.1999;H3F3A|0.20985;MYH10|5.579275;SOS2|11.48525;PLK1|0.415475	small GTPase mediated signal transduction	GO:0007264	6.13E-06	1.38E-04	15	7
4,6,7	PABPC1|0.3285;DNMT1|0.172;HIST1H4A|0.23835;SRRT|0.39875;HIST1H3A|0.455625;H3F3A|0.20985	gene silencing	GO:0016458	6.17E-06	1.39E-04	6	7
4,5	HSP90AB1|0.458775;ASPH|2.59965;YWHAG|2.359475;ANXA5|3.637725;GSN|2.4555;AKR1B1|2.235325;G3BP1|0.3904;BAZ1B|0.460625;PSME3|0.401575;LAMTOR2|7.841025;SQSTM1|6.007525;ATG7|2.120975;MAPK14|0.483325;RHOG|2.449775;SNW1|0.473175;STAT3|0.4995;NCDN|0.4114;SLC2A1|5.081875;PLEK|2.071125;VTN|2.01125;ARHGDIA|3.8215;TTK|0.488375;SOS2|11.48525;SOD2|2.175425;PTP4A3|0.3433;CHAF1B|0.2723	regulation of cell communication	GO:0010646	6.26E-06	1.39E-04	26	5
5,6	HSPA8|0.3721;ENO1|2.1939;PABPC1|0.3285;DNMT1|0.172;HIST1H4A|0.23835;CBX3|0.485775;PRDX5|3.0143;HIST1H3A|0.455625;SNW1|0.473175;STAT3|0.4995;TXNIP|2.0553;S100A11|2.531875;RBBP7|0.218475;H3F3A|0.20985;PLK1|0.415475;ZNF706|0.3974	negative regulation of nucleobase-containing compound metabolic process	GO:0045934	6.48E-06	1.40E-04	16	6
4	HSP90AB1|0.458775;PRDX5|3.0143;MAPK14|0.483325;STAT3|0.4995;HBA1|2.13735;GCH1|4.01735;SOD2|2.1754	reactive oxygen species metabolic process	GO:0072593	6.90E-06	1.44E-04	7	4
5,6	ACTG1|2.1999;LCP1|2.170475;ASPH|2.59965;HIST1H4A|0.23835;YWHAG|2.359475;GSN|2.4555;AKR1B1|2.235325;G3BP1|0.3904;ERO1L|2.370575;PSME3|0.401575;LAMTOR2|7.841025;SQSTM1|6.007525;MAPK14|0.483325;HIST1H3A|0.455625;RHOG|2.449775;SNW1|0.473175;STAT3|0.4995;PLEK|2.071125;NDE1|0.446425;NDRG1|10.0073;ARHGDIA|3.8215;ACTB|2.1999;H3F3A|0.20985;MYH10|5.579275;SOS2|11.48525;PLK1|0.415475;SOD2|2.17542	intracellular signal transduction	GO:0035556	7.00E-06	1.52E-04	27	6

2	HSPA8|0.3721;ACTG1|2.1999;HSP90AB1|0.458775;LCP1|2.170475;ENO1|2.1939;HSPD1|2.88995;HIST1H2BN|0.481625;RPL3|3.30745;VIM|2.241075;CCT7|2.06135;PABPC1|0.3285;SARS|2.2366;ASPH|2.59965;YARS|2.288275;ATP6V1B2|2.0575;MCM3|0.35205;KRT9|0.268525;RPL4|2.063325;NSF|2.017425;GMPS|0.496875;MTHFD1L|6.667175;CKB|0.486625;NARS|2.162;MCM2|0.491625;HSPA4L|2.13105;SMC2|0.485025;DNMT1|0.172;ALDH1L2|2.148;HIST1H4A|0.23835;KPNA2|0.186675;VDAC3|2.015475;YWHAG|2.359475;ANXA5|3.637725;SRRT|0.39875;GSN|2.4555;CNDP2|2.0938;NCAPG|0.4528;CBX3|0.485775;GTF2E1|0.300475;AKR1B1|2.235325;PABPC4|0.26245;LIG1|0.440425;SLC25A1|2.1121;G3BP1|0.3904;ERO1L|2.370575;PRDX5|3.0143;BAZ1B|0.460625;ESD|2.155525;ISYNA1|0.49065;ALDOC|2.04725;PSME3|0.401575;RRM2|0.21675;EIF4H|0.275675;LAMTOR2|7.841025;MCMBP|0.4769;RPL27|2.15885;SQSTM1|6.007525;ATG7|2.120975;PCNA|0.404975;MAPK14|0.483325;HIST1H3A|0.455625;BTF3|0.2449;TFRC|0.403125;RHOG|2.449775;TOP2A|0.481675;SNW1|0.473175;STAT3|0.4995;TUBG1|0.4102;NCDN|0.4114;SLC2A1|5.081875;TXNIP|2.0553;VAT1|2.702925;IPO8|0.443925;PLEK|2.071125;NDE1|0.446425;S100A11|2.531875;HBA1|2.13735;NDRG1|10.0073;IK|0.480125;VTN|2.01125;RBBP7|0.218475;ARHGDIA|3.8215;ACTB|2.1999;DNPEP|2.077575;H3F3A|0.20985;MYH10|5.579275;TTK|0.488375;SOS2|11.48525;ASL|2.252975;ALB|0.23405;CDC123|0.29925;PLK1|0.415475;GCH1|4.01735;SOD2|2.175425;PTP4A3|0.3433;HEATR9|2.53905;TBCA|0.3515;CETN3|0.4171;IFITM2|5.68315;ZNF706|0.3974;CHAF1B|0.2723;PL[illegible]	cellular process	GO:0009987	7.60E-06	1.53E-04	102	2
6	HIST1H2BN|0.481625;MCM2|0.491625;HIST1H4A|0.23835;HIST1H3A|0.455625;RBBP7|0.218475;H3F3A|0.20985;CHAF1B|0.2723	protein-DNA complex assembly	GO:0065004	8.02E-06	1.65E-04	7	6
4	ACTG1|2.1999;HSP90AB1|0.458775;HSPD1|2.88995;KRT1|0.4198;ANXA5|3.637725;PRDX5|3.0143;PSME3|0.401575;ATG7|2.120975;MAPK14|0.483325;HIST1H3A|0.455625;TFRC|0.403125;STAT3|0.4995;TXNIP|2.0553;VTN|2.01125;ACTB|2.1999;GCH1|4.01735;IFITM2|5.68315;CHAF1B|0.2723;PLP1|3.936	defense response	GO:0006952	8.24E-06	1.73E-04	19	4
6,7	HSP90AB1|0.458775;HSPD1|2.88995;YWHAG|2.359475;ANXA5|3.637725;GSN|2.4555;PRDX5|3.0143;PSME3|0.401575;SQSTM1|6.007525;ATG7|2.120975;TOP2A|0.481675;STAT3|0.4995;TXNIP|2.0553;ARHGDIA|3.8215;SOS2|11.48525;ALB|0.23405;PLK1|0.415475;SOD2|2.175425	regulation of apoptotic process	GO:0042981	8.76E-06	1.76E-04	17	7
3	HSPA8|0.3721;HSP90AB1|0.458775;HSPD1|2.88995;AKR1B1|2.235325;ERO1L|2.370575;ATG7|2.120975;PCNA|0.404975;MAPK14|0.483325;TFRC|0.403125;SLC2A1|5.081875;TXNIP|2.0553;NDRG1|10.0073;RBBP7|0.218475;ARH	response to abiotic stimulus	GO:0009628	9.28E-06	1.86E-04	14	3
6,7	HSP90AB1|0.458775;HSPD1|2.88995;ANXA5|3.637725;PRDX5|3.0143;PSME3|0.401575;SQSTM1|6.007525;ATG7|2.120975;STAT3|0.4995;ARHGDIA|3.8215;ALB|0.23405;PLK1|0.415475;SOD2|2.175425	negative regulation of apoptotic process	GO:0043066	9.32E-06	1.94E-04	12	7
5,6	HSP90AB1|0.458775;HSPD1|2.88995;YWHAG|2.359475;ANXA5|3.637725;GSN|2.4555;PRDX5|3.0143;PSME3|0.401575;SQSTM1|6.007525;ATG7|2.120975;TOP2A|0.481675;STAT3|0.4995;TXNIP|2.0553;ARHGDIA|3.8215;SOS2|11.48525;ALB|0.23405;PLK1|0.415475;SOD2|2.175425	regulation of programmed cell death	GO:0043067	9.54E-06	1.94E-04	17	6
3	HSPA8|0.3721;ACTG1|2.1999;HSP90AB1|0.458775;LCP1|2.170475;HSPD1|2.88995;VIM|2.241075;ASPH|2.59965;YARS|2.288275;ATP6V1B2|2.0575;NSF|2.017425;HIST1H4A|0.23835;KPNA2|0.186675;YWHAG|2.359475;ANXA5|3.637725;GSN|2.4555;AKR1B1|2.235325;G3BP1|0.3904;ERO1L|2.370575;BAZ1B|0.460625;PSME3|0.401575;LAMTOR2|7.841025;SQSTM1|6.007525;MAPK14|0.483325;HIST1H3A|0.455625;RHOG|2.449775;SNW1|0.473175;STAT3|0.4995;NCDN|0.4114;SLC2A1|5.081875;TXNIP|2.0553;IPO8|0.443925;PLEK|2.071125;NDE1|0.446425;S100A11|2.531875;NDRG1|10.0073;IK|0.480125;VTN|2.01125;ARHGDIA|3.8215;ACTB|2.1999;H3F3A|0.20985;MYH10|5.579275;TTK|0.488375;SOS2|11.48525;PLK1|0.415475;SOD2|2.175425;PTP4A3|0.3433;IFITM2|5.68315;PLP1|3.936	single organism signaling	GO:0044700	9.56E-06	1.95E-04	48	3
4	HSPA8|0.3721;ENO1|2.1939;HSPD1|2.88995;RPL3|3.30745;PABPC1|0.3285;SARS|2.2366;ASPH|2.59965;YARS|2.288275;ATP6V1B2|2.0575;MCM3|0.35205;RPL4|2.063325;GMPS|0.496875;MTHFD1L|6.667175;NARS|2.162;MCM2|0.491625;DNMT1|0.172;ALDH1L2|2.148;HIST1H4A|0.23835;KPNA2|0.186675;YWHAG|2.359475;SRRT|0.39875;CBX3|0.485775;GTF2E1|0.300475;PABPC4|0.26245;LIG1|0.440425;G3BP1|0.3904;ERO1L|2.370575;PRDX5|3.0143;BAZ1B|0.460625;ALDOC|2.04725;RRM2|0.21675;MCMBP|0.4769;RPL27|2.15885;SQSTM1|6.007525;ATG7|2.120975;PCNA|0.404975;MAPK14|0.483325;HIST1H3A|0.455625;BTF3|0.2449;RHOG|2.449775;TOP2A|0.481675;SNW1|0.473175;STAT3|0.4995;TXNIP|2.0553;S100A11|2.531875;RBBP7|0.218475;H3F3A|0.20985;PLK1|0.415475;GCH1|4[illegible]	heterocycle metabolic process	GO:0046483	9.58E-06	1.95E-04	52	4
5,6,7	HSPA8|0.3721;ENO1|2.1939;PABPC1|0.3285;DNMT1|0.172;HIST1H4A|0.23835;CBX3|0.485775;PRDX5|3.0143;HIST1H3A|0.455625;SNW1|0.473175;STAT3|0.4995;TXNIP|2.0553;RBBP7|0.218475;H3F3A|0.20985;PLK1|0.415475;	negative regulation of RNA metabolic process	GO:0051253	9.70E-06	1.95E-04	15	7

5	ENO1|2.1939;AKR1B1|2.235325;SLC25A1|2.1121;ISYNA1|0.49065;ALDOC|2.04725;SLC2A1|5.081875;PLEK|2.071	carbohydrate biosynthetic process	GO:0016051	9.75E-06	1.95E-04	7	5
4	HSPA8|0.3721;ENO1|2.1939;HSPD1|2.88995;RPL3|3.30745;PABPC1|0.3285;SARS|2.2366;ASPH|2.59965;YARS|2.288275;ATP6V1B2|2.0575;MCM3|0.35205;RPL4|2.063325;GMPS|0.496875;MTHFD1L|6.667175;NARS|2.162;MCM2|0.491625;DNMT1|0.172;ALDH1L2|2.148;HIST1H4A|0.23835;KPNA2|0.186675;YWHAG|2.359475;SRRT|0.39875;CBX3|0.485775;GTF2E1|0.300475;AKR1B1|2.235325;PABPC4|0.26245;LIG1|0.440425;G3BP1|0.3904;PRDX5|3.0143;BAZ1B|0.460625;ALDOC|2.04725;RRM2|0.21675;MCMBP|0.4769;RPL27|2.15885;SQSTM1|6.007525;ATG7|2.120975;PCNA|0.404975;MAPK14|0.483325;HIST1H3A|0.455625;BTF3|0.2449;RHOG|2.449775;TOP2A|0.481675;SNW1|0.473175;STAT3|0.4995;TXNIP|2.0553;S100A11|2.531875;RBBP7|0.218475;H3F3A|0.20985;PLK1|0.415475;G [illegible]	cellular aromatic compound metabolic process	GO:0006725	9.77E-06	1.95E-04	52	4
5	HSP90AB1|0.458775;PABPC1|0.3285;ATG7|2.120975;STAT3|0.4995;TTK|0.488375;PLK1|0.415475	negative regulation of cellular catabolic process	GO:0031330	9.86E-06	1.95E-04	6	5
5	HSPA8|0.3721;HSP90AB1|0.458775;ENO1|2.1939;RPL3|3.30745;PABPC1|0.3285;SARS|2.2366;ASPH|2.59965;YARS|2.288275;RPL4|2.063325;GMPS|0.496875;MTHFD1L|6.667175;NARS|2.162;DNMT1|0.172;HIST1H4A|0.23835;YWHAG|2.359475;SRRT|0.39875;CNDP2|2.0938;CBX3|0.485775;GTF2E1|0.300475;PABPC4|0.26245;LIG1|0.440425;PRDX5|3.0143;BAZ1B|0.460625;RRM2|0.21675;EIF4H|0.275675;RPL27|2.15885;SQSTM1|6.007525;ATG7|2.120975;PCNA|0.404975;MAPK14|0.483325;HIST1H3A|0.455625;BTF3|0.2449;RHOG|2.449775;TOP2A|0.481675;SNW1|0.473175;STAT3|0.4995;TXNIP|2.0553;RBBP7|0.218475;H3F3A|0.20985;ASL|2.252975;PLK1|0.415475;GCH1|4.01735;SOD2|2.175425;ZNF706|0.3974;CHAF1B|0.2723	cellular nitrogen compound biosynthetic process	GO:0044271	1.02E-05	1.95E-04	45	5
2	HSPA8|0.3721;ACTG1|2.1999;HSP90AB1|0.458775;LCP1|2.170475;HSPD1|2.88995;VIM|2.241075;ASPH|2.59965;YARS|2.288275;ATP6V1B2|2.0575;NSF|2.017425;HIST1H4A|0.23835;KPNA2|0.186675;YWHAG|2.359475;ANXA5|3.637725;GSN|2.4555;AKR1B1|2.235325;G3BP1|0.3904;ERO1L|2.370575;BAZ1B|0.460625;PSME3|0.401575;LAMTOR2|7.841025;SQSTM1|6.007525;MAPK14|0.483325;HIST1H3A|0.455625;RHOG|2.449775;SNW1|0.473175;STAT3|0.4995;NCDN|0.4114;SLC2A1|5.081875;TXNIP|2.0553;IPO8|0.443925;PLEK|2.071125;NDE1|0.446425;S100A11|2.531875;NDRG1|10.0073;IK|0.480125;VTN|2.01125;ARHGDIA|3.8215;ACTB|2.1999;H3F3A|0.20985;MYH10|5.579275;TTK|0.488375;SOS2|11.48525;PLK1|0.415475;SOD2|2.175425;PTP4A3|0.3433;IFITM2|5.68315;PLP1|3.936	signaling	GO:0023052	1.04E-05	2.01E-04	48	2
5,6	HSP90AB1|0.458775;HSPD1|2.88995;ANXA5|3.637725;PRDX5|3.0143;PSME3|0.401575;SQSTM1|6.007525;ATG7|2.120975;STAT3|0.4995;ARHGDIA|3.8215;ALB|0.23405;PLK1|0.415475;SOD2|2.175425	negative regulation of programmed cell death	GO:0043069	1.05E-05	2.03E-04	12	6
3	ACTG1|2.1999;HSP90AB1|0.458775;LCP1|2.170475;HSPD1|2.88995;KRT1|0.4198;ANXA5|3.637725;ATG7|2.120975;HIST1H3A|0.455625;VTN|2.01125;ACTB|2.1999;IFITM2|5.68315;CHAF1B|0.2723	immune effector process	GO:0002252	1.06E-05	2.03E-04	12	3
5	HSPD1|2.88995;AKR1B1|2.235325;LIG1|0.440425;TXNIP|2.0553;HBA1|2.13735	response to hydrogen peroxide	GO:0042542	1.17E-05	2.04E-04	5	5
7,8	MCM3|0.35205;MCM2|0.491625;LIG1|0.440425;PCNA|0.404975	DNA strand elongation involved in DNA	GO:0006271	1.18E-05	2.23E-04	4	8
4	HSPA8|0.3721;HSP90AB1|0.458775;ANXA5|3.637725;AKR1B1|2.235325;LIG1|0.440425;ERO1L|2.370575;PRDX5|3.0143;BAZ1B|0.460625;PSME3|0.401575;SQSTM1|6.007525;ATG7|2.120975;PCNA|0.404975;MAPK14|0.483325;TOP2A|0.481675;SNW1|0.473175;SLC2A1|5.081875;NDRG1|10.0073;RBBP7|0.218475;ALB|0.23405;PLK1|0.415475;SOD2|2.175425;CHAF1B|0.2723	cellular response to stress	GO:0033554	1.23E-05	2.24E-04	22	4
5,6	HSP90AB1|0.458775;HSPD1|2.88995;PABPC1|0.3285;ASPH|2.59965;DNMT1|0.172;GSN|2.4555;PSME3|0.401575;LAMTOR2|7.841025;SQSTM1|6.007525;ATG7|2.120975;MAPK14|0.483325;SNW1|0.473175;PLEK|2.071125;VTN|2.01125;TTK|0.488375;PLK1|0.415475	positive regulation of cellular protein metabolic process	GO:0032270	1.26E-05	2.32E-04	16	6
5	RPL3|3.30745;PABPC1|0.3285;SARS|2.2366;YARS|2.288275;RPL4|2.063325;MTHFD1L|6.667175;NARS|2.162;ALDH1L2|2.148;CNDP2|2.0938;PABPC4|0.26245;EIF4H|0.275675;RPL27|2.15885;DNPEP|2.077575;ASL|2.252975;GCH1|4.01735;ZNF706|0.3974	cellular amide metabolic process	GO:0043603	1.26E-05	2.34E-04	16	5
6	VIM|2.241075;GSN|2.4555;STAT3|0.4995;NDRG1|10.0073;VTN|2.01125;PLP1|3.936	glial cell differentiation	GO:0010001	1.27E-05	2.34E-04	6	6
5	HSPA8|0.3721;ENO1|2.1939;DNMT1|0.172;HIST1H4A|0.23835;CBX3|0.485775;PRDX5|3.0143;HIST1H3A|0.455625;SNW1|0.473175;STAT3|0.4995;TXNIP|2.0553;PLEK|2.071125;S100A11|2.531875;RBBP7|0.218475;H3F3A|0.20985;PLK1|0.415475;ZNF706|0.3974	negative regulation of cellular biosynthetic process	GO:0031327	1.27E-05	2.34E-04	16	5
4,5	LCP1|2.170475;DNMT1|0.172;YWHAG|2.359475;ANXA5|3.637725;GSN|2.4555;SQSTM1|6.007525;MAPK14|0.483325;RHOG|2.449775;SNW1|0.473175;PLEK|2.071125;VTN|2.01125;ARHGDIA|3.8215;PLK1|0.415475;CHAF1B|0.2	positive regulation of cellular component organization	GO:0051130	1.44E-05	2.34E-04	14	5

4	ENO1|2.1939;ALDH1L2|2.148;ANXA5|3.637725;LIG1|0.440425;PRDX5|3.0143;ESD|2.155525;ALDOC|2.04725;SQSTM1|6.007525;ATG7|2.120975;STAT3|0.4995;HBA1|2.13735;PLBD2|2.666725;TTK|0.488375;ASL|2.252975;PLK1|0.415475;CHAF1B|0.2723	single-organism catabolic process	GO:0044712	1.46E-05	2.63E-04	16	4
3	HSP90AB1|0.458775;ENO1|2.1939;RPL3|3.30745;PABPC1|0.3285;RPL4|2.063325;NSF|2.017425;ALDH1L2|2.148;ANXA5|3.637725;PABPC4|0.26245;LIG1|0.440425;PRDX5|3.0143;ESD|2.155525;ALDOC|2.04725;PSME3|0.401575;RPL27|2.15885;SQSTM1|6.007525;ATG7|2.120975;STAT3|0.4995;HBA1|2.13735;PLBD2|2.666725;TTK|0.488375;ASL|2.252975;PLK1|0.415475;CHAF1B|0.2723	catabolic process	GO:0009056	1.47E-05	2.64E-04	24	3
5	HIST1H2BN|0.481625;MCM2|0.491625;HIST1H4A|0.23835;HIST1H3A|0.455625;RBBP7|0.218475;H3F3A|0.20985;CHAF1B|0.2723	protein-DNA complex subunit organization	GO:0071824	1.48E-05	2.64E-04	7	5
6,7	MCM3|0.35205;MCM2|0.491625;LIG1|0.440425;PCNA|0.404975	DNA strand elongation	GO:0022616	1.48E-05	2.64E-04	4	7
4	HSPA8|0.3721;ENO1|2.1939;DNMT1|0.172;HIST1H4A|0.23835;CBX3|0.485775;PRDX5|3.0143;HIST1H3A|0.455625;SNW1|0.473175;STAT3|0.4995;TXNIP|2.0553;PLEK|2.071125;S100A11|2.531875;RBBP7|0.218475;H3F3A|0.20985;PLK1|0.415475;ZNF706|0.3974	negative regulation of biosynthetic process	GO:0009890	1.50E-05	2.64E-04	16	4
6	HSPA8|0.3721;ENO1|2.1939;DNMT1|0.172;HIST1H4A|0.23835;CBX3|0.485775;PRDX5|3.0143;HIST1H3A|0.455625;SNW1|0.473175;STAT3|0.4995;TXNIP|2.0553;S100A11|2.531875;RBBP7|0.218475;H3F3A|0.20985;PLK1|0.415475;ZNF706|0.3974	negative regulation of cellular macromolecule biosynthetic process	GO:2000113	1.61E-05	2.64E-04	15	6
6,7,8	HSPA8|0.3721;ENO1|2.1939;DNMT1|0.172;HIST1H4A|0.23835;CBX3|0.485775;PRDX5|3.0143;HIST1H3A|0.455625;SNW1|0.473175;STAT3|0.4995;TXNIP|2.0553;RBBP7|0.218475;H3F3A|0.20985;PLK1|0.415475;ZNF706|0.3974	negative regulation of transcription, DNA-templated	GO:0045892	1.69E-05	2.82E-04	14	8
6,7	DNMT1|0.172;HIST1H4A|0.23835;SRRT|0.39875;BAZ1B|0.460625;HIST1H3A|0.455625;RBBP7|0.218475;H3F3A|0.	regulation of gene expression, epigenetic	GO:0040029	1.73E-05	2.94E-04	7	7
6,7,8	HSPA8|0.3721;ACTG1|2.1999;HSP90AB1|0.458775;ATG7|2.120975;RHOG|2.449775;ARHGDIA|3.8215;ACTB|2.1999;MYH10|5.579275;SOS2|11.48525;PLP1|3.936	axon development	GO:0061564	1.76E-05	2.99E-04	10	8
6,7	ACTG1|2.1999;VIM|2.241075;ATP6V1B2|2.0575;SQSTM1|6.007525;MAPK14|0.483325;SNW1|0.473175;STAT3|0.4995;TXNIP|2.0553;VTN|2.01125;ARHGDIA|3.8215;ACTB|2.1999;MYH10|5.579275;TTK|0.488375;SOS2|11.48525;	enzyme linked receptor protein signaling pathway	GO:0007167	1.78E-05	3.03E-04	15	7
4	HSPA8|0.3721;ENO1|2.1939;PABPC1|0.3285;DNMT1|0.172;HIST1H4A|0.23835;CBX3|0.485775;PRDX5|3.0143;HIST1H3A|0.455625;SNW1|0.473175;STAT3|0.4995;TXNIP|2.0553;S100A11|2.531875;RBBP7|0.218475;H3F3A|0.20985;PLK1|0.415475;ZNF706|0.3974	negative regulation of nitrogen compound metabolic process	GO:0051172	1.79E-05	3.04E-04	16	4
3	HSPA8|0.3721;HSP90AB1|0.458775;ENO1|2.1939;HSPD1|2.88995;RPL3|3.30745;CCT7|2.06135;PABPC1|0.3285;SARS|2.2366;ASPH|2.59965;KRT1|0.4198;YARS|2.288275;ATP6V1B2|2.0575;MCM3|0.35205;RPL4|2.063325;NSF|2.017425;GMPS|0.496875;MTHFD1L|6.667175;CKB|0.486625;NARS|2.162;MCM2|0.491625;HSPA4L|2.13105;DNMT1|0.172;ALDH1L2|2.148;HIST1H4A|0.23835;KPNA2|0.186675;YWHAG|2.359475;SRRT|0.39875;GSN|2.4555;CNDP2|2.0938;CBX3|0.485775;GTF2E1|0.300475;AKR1B1|2.235325;PABPC4|0.26245;LIG1|0.440425;SLC25A1|2.1121;G3BP1|0.3904;ERO1L|2.370575;PRDX5|3.0143;BAZ1B|0.460625;ISYNA1|0.49065;ALDOC|2.04725;PSME3|0.401575;RRM2|0.21675;EIF4H|0.275675;ACAT2|2.108325;LAMTOR2|7.841025;MCMBP|0.4769;RPL27|2.15885;SQSTM1|6.007525;ATG7|2.120975;PCNA|0.404975;MAPK14|0.483325;HIST1H3A|0.455625;BTF3|0.2449;RHOG|2.449775;TOP2A|0.481675;SNW1|0.473175;STAT3|0.4995;SLC2A1|5.081875;TXNIP|2.0553;PLEK|2.071125;S100A11|2.531875;VTN|2.01125;RBBP7|0.218475;PLBD2|2.666725;ACTB|2.1999;DNPEP|2.077575;H3F3A|0.20985;TTK|0.488375;ASL|2.252975;ALB|0.23405;PLK1|0.415475;GCH1|4.01735;SOD2|2.175425;PTP4A3|0.3433;TBCA|0.3515;ZNF706|0.3974;CHAF1B|0.2723;PLP1|3.936	primary metabolic process	GO:0044238	1.81E-05	3.04E-04	79	3
6,8,9	HIST1H4A|0.23835;HIST1H3A|0.455625;H3F3A|0.20985	chromatin silencing at rDNA	GO:0000183	1.85E-05	3.06E-04	3	9
6	HSPA8|0.3721;HSP90AB1|0.458775;GSN|2.4555	fibril organization	GO:0097435	1.85E-05	3.08E-04	3	6
5,6	HSPA8|0.3721;ENO1|2.1939;PABPC1|0.3285;DNMT1|0.172;HIST1H4A|0.23835;SRRT|0.39875;CBX3|0.485775;PRDX5|3.0143;HIST1H3A|0.455625;SNW1|0.473175;STAT3|0.4995;TXNIP|2.0553;RBBP7|0.218475;H3F3A|0.20985;PLK1|0.415475;ZNF706|0.3974	negative regulation of gene expression	GO:0010629	1.86E-05	3.08E-04	16	6
3	HSP90AB1|0.458775;LCP1|2.170475;RPL3|3.30745;ASPH|2.59965;ATP6V1B2|2.0575;RPL4|2.063325;NSF|2.017425;KPNA2|0.186675;YWHAG|2.359475;GSN|2.4555;LAMTOR2|7.841025;RPL27|2.15885;SQSTM1|6.007525;ATG7|2.120975;MAPK14|0.483325;BTF3|0.2449;TFRC|0.403125;RHOG|2.449775;STAT3|0.4995;SLC2A1|5.081875;TXNIP|2.0553;IPO8|0.443925;PLEK|2.071125;ARHGDIA|3.8215;ALB|0.23405;PLK1|0.415475	macromolecule localization	GO:0033036	1.97E-05	3.09E-04	26	3
4,5	HSP90AB1|0.458775;HSPD1|2.88995;ANXA5|3.637725;PRDX5|3.0143;PSME3|0.401575;SQSTM1|6.007525;ATG7|2.120975;STAT3|0.4995;ARHGDIA|3.8215;ALB|0.23405;PLK1|0.415475;SOD2|2.175425	negative regulation of cell death	GO:0060548	2.02E-05	3.25E-04	12	5

5	HIST1H2BN|0.481625;MCM2|0.491625;DNMT1|0.172;HIST1H4A|0.23835;CBX3|0.485775;BAZ1B|0.460625;ATG7|2.120975;HIST1H3A|0.455625;SNW1|0.473175;RBBP7|0.218475;ACTB|2.1999;H3F3A|0.20985;CHAF1B|0.2723	chromatin organization	GO:0006325	2.13E-05	3.32E-04	13	5
6,7	DNMT1|0.172;HIST1H4A|0.23835;HIST1H3A|0.455625;RBBP7|0.218475;H3F3A|0.20985	negative regulation of gene expression,	GO:0045814	2.20E-05	3.46E-04	5	7
4,5	HSPD1|2.88995;YWHAG|2.359475;ANXA5|3.637725;GSN|2.4555;SQSTM1|6.007525;ATG7|2.120975;TOP2A|0.481675;TXNIP|2.0553;HBA1|2.13735;SOS2|11.48525	positive regulation of cell death	GO:0010942	2.20E-05	3.54E-04	10	5
4,5	HSP90AB1|0.458775;LCP1|2.170475;RPL3|3.30745;ASPH|2.59965;ATP6V1B2|2.0575;RPL4|2.063325;NSF|2.017425;KPNA2|0.186675;YWHAG|2.359475;RPL27|2.15885;ATG7|2.120975;MAPK14|0.483325;BTF3|0.2449;TFRC|0.403125;RHOG|2.449775;STAT3|0.4995;SLC2A1|5.081875;TXNIP|2.0553;IPO8|0.443925;PLEK|2.071125;PLK1|0.415475	establishment of protein localization	GO:0045184	2.36E-05	3.54E-04	21	5
7	HSPA8|0.3721;ENO1|2.1939;DNMT1|0.172;HIST1H4A|0.23835;CBX3|0.485775;PRDX5|3.0143;HIST1H3A|0.455625;SNW1|0.473175;STAT3|0.4995;TXNIP|2.0553;RBBP7|0.218475;H3F3A|0.20985;PLK1|0.415475;ZNF706|0.3974	negative regulation of nucleic acid-templated transcription	GO:1903507	2.47E-05	3.78E-04	14	7
4,5	HSPA8|0.3721;ACTG1|2.1999;HSP90AB1|0.458775;VIM|2.241075;YWHAG|2.359475;SRRT|0.39875;GSN|2.4555;ATG7|2.120975;RHOG|2.449775;SNW1|0.473175;STAT3|0.4995;NCDN|0.4114;NDRG1|10.0073;ARHGDIA|3.8215;ACTB|2.1999;MYH10|5.579275;SOS2|11.48525;ZNF706|0	cell development	GO:0048468	2.53E-05	3.93E-04	19	5
6	RPL3|3.30745;PABPC1|0.3285;SARS|2.2366;YARS|2.288275;RPL4|2.063325;MTHFD1L|6.667175;NARS|2.162;CNDP2|2.0938;PABPC4|0.26245;EIF4H|0.275675;RPL27|2.15885;ASL|2.252975;GCH1|4.01735;ZNF706|0.3974	amide biosynthetic process	GO:0043604	2.55E-05	4.00E-04	14	6
6	KRT1|0.4198;ANXA5|3.637725;GSN|2.4555;PLEK|2.071125;VTN|2.01125	regulation of wound healing	GO:0061041	2.77E-05	4.01E-04	5	6
6	HSPA8|0.3721;ENO1|2.1939;DNMT1|0.172;HIST1H4A|0.23835;CBX3|0.485775;PRDX5|3.0143;HIST1H3A|0.455625;SNW1|0.473175;STAT3|0.4995;TXNIP|2.0553;RBBP7|0.218475;H3F3A|0.20985;PLK1|0.415475;ZNF706|0.3974	negative regulation of RNA biosynthetic process	GO:1902679	2.78E-05	4.32E-04	14	6
6,7	MTHFD1L|6.667175;ALDH1L2|2.148;GCH1|4.01735	tetrahydrofolate metabolic process	GO:0046653	2.92E-05	4.32E-04	3	7
6	HSPA8|0.3721;HSP90AB1|0.458775;HSPD1|2.88995;CCT7|2.06135;HSPA4L|2.13105;ERO1L|2.370575;ACTB|2.1999;TBCA|0.3515	protein folding	GO:0006457	2.98E-05	4.51E-04	8	6
5,6	HSPA8|0.3721;ENO1|2.1939;DNMT1|0.172;HIST1H4A|0.23835;CBX3|0.485775;PRDX5|3.0143;HIST1H3A|0.455625;SNW1|0.473175;STAT3|0.4995;TXNIP|2.0553;S100A11|2.531875;RBBP7|0.218475;H3F3A|0.20985;PLK1|0.415475;ZNF706|0.3974	negative regulation of macromolecule biosynthetic process	GO:0010558	3.15E-05	4.58E-04	15	6
3	HSP90AB1|0.458775;ASPH|2.59965;NSF|2.017425;YWHAG|2.359475;GSN|2.4555;AKR1B1|2.235325;G3BP1|0.3904;BAZ1B|0.460625;PSME3|0.401575;LAMTOR2|7.841025;SQSTM1|6.007525;MAPK14|0.483325;RHOG|2.449775;SNW1|0.473175;STAT3|0.4995;NCDN|0.4114;SLC2A1|5.081875;PLEK|2.071125;VTN|2.01125;ARHGDIA|3.8215;TTK|0.488375;SOS2|11.48525;SOD2|2.175425;PTP4A3|0.3	regulation of signaling	GO:0023051	3.41E-05	4.82E-04	24	3
5,6,7	HSPA8|0.3721;ACTG1|2.1999;HSP90AB1|0.458775;VIM|2.241075;ATG7|2.120975;RHOG|2.449775;NCDN|0.4114;ARHGDIA|3.8215;ACTB|2.1999;MYH10|5.579275;SOS2|11.48525;PLP1|3.936	neuron projection development	GO:0031175	3.62E-05	5.18E-04	12	7
7,8	MCM3|0.35205;MCM2|0.491625;LIG1|0.440425;MCMBP|0.4769;PCNA|0.404975;TOP2A|0.481675	DNA-dependent DNA replication	GO:0006261	3.69E-05	5.47E-04	6	8
4	HSP90AB1|0.458775;PABPC1|0.3285;ATG7|2.120975;STAT3|0.4995;TTK|0.488375;PLK1|0.415475	negative regulation of catabolic process	GO:0009895	3.69E-05	5.47E-04	6	4
5,7,8,9	DNMT1|0.172;HIST1H4A|0.23835;HIST1H3A|0.455625;H3F3A|0.20985	DNA methylation	GO:0006306	3.71E-05	5.47E-04	4	9
7,8	DNMT1|0.172;HIST1H4A|0.23835;HIST1H3A|0.455625;H3F3A|0.20985	DNA alkylation	GO:0006305	3.71E-05	5.47E-04	4	8
4	HSPD1|2.88995;KRT1|0.4198;ANXA5|3.637725;SQSTM1|6.007525;ATG7|2.120975;MAPK14|0.483325;HIST1H3A|0.455625;SNW1|0.473175;PLEK|2.071125;VTN|2.01125;CHAF1B|0.2723	regulation of response to external stimulus	GO:0032101	3.72E-05	5.47E-04	11	4
2	HSPA8|0.3721;ACTG1|2.1999;HSP90AB1|0.458775;LCP1|2.170475;ANXA5|3.637725;GSN|2.4555;MAPK14|0.483325;RHOG|2.449775;STAT3|0.4995;NDE1|0.446425;VTN|2.01125;ARHGDIA|3.8215;ACTB|2.1999;MYH10|5.579275;SOS2|11.48525;PTP4A3|0.3433;IFITM2|5.68315	locomotion	GO:0040011	3.82E-05	5.47E-04	17	2
4,5	RPL3|3.30745;RPL4|2.063325;NSF|2.017425;YWHAG|2.359475;GSN|2.4555;RPL27|2.15885;ATG7|2.120975;RHOG|2.449775;STAT3|0.4995;VAT1|2.702925;NDRG1|10.0073;MYH10|5.579275;PLK1|0.415475	single-organism membrane organization	GO:0044802	4.20E-05	5.59E-04	13	5
6,7	HIST1H4A|0.23835;ANXA5|3.637725;RRM2|0.21675;SQSTM1|6.007525;HIST1H3A|0.455625;HBA1|2.13735;GCH1|4.01735;SOD2|2.175425	protein oligomerization	GO:0051259	4.39E-05	6.12E-04	8	7
3	RPL3|3.30745;RPL4|2.063325;RPL27|2.15885;ATG7|2.120975;TOP2A|0.481675;SNW1|0.473175	multi-organism metabolic process	GO:0044033	4.59E-05	6.36E-04	6	3
4	HSPA8|0.3721;ACTG1|2.1999;HSP90AB1|0.458775;VIM|2.241075;VDAC3|2.015475;GSN|2.4555;ATG7|2.120975;RHOG|2.449775;NCDN|0.4114;PLEK|2.071125;ARHGDIA|3.8215;ACTB|2.1999;MYH10|5.579275;SOS2|11.48525;	cell projection organization	GO:0030030	4.62E-05	6.62E-04	15	4
3,4	ANXA5|3.637725;GSN|2.4555;SQSTM1|6.007525;HIST1H3A|0.455625;TOP2A|0.481675;SNW1|0.473175;IFITM2|5.68315;CHAF1B|0.2723	regulation of multi-organism process	GO:0043900	4.66E-05	6.62E-04	8	4

5	LCP1|2.170475;DNMT1|0.172;YWHAG|2.359475;ANXA5|3.637725;GSN|2.4555;SQSTM1|6.007525;SNW1|0.473175;PLEK|2.071125;PLK1|0.415475;CHAF1B|0.2723	positive regulation of organelle organization	GO:0010638	4.71E-05	6.65E-04	10	5
4	ENO1|2.1939;MTHFD1L|6.667175;ALDH1L2|2.148;AKR1B1|2.235325;SLC25A1|2.1121;PRDX5|3.0143;ALDOC|2.04725;STAT3|0.4995;GCH1|4.01735	cofactor metabolic process	GO:0051186	4.72E-05	6.67E-04	9	4
7	VIM|2.241075;GSN|2.4555;STAT3|0.4995;NDRG1|10.0073;VTN|2.01125;PLP1|3.936	gliogenesis	GO:0042063	4.82E-05	6.67E-04	6	7
5	LCP1|2.170475;DNMT1|0.172;VDAC3|2.015475;YWHAG|2.359475;ANXA5|3.637725;GSN|2.4555;SQSTM1|6.007525;ATG7|2.120975;SNW1|0.473175;VAT1|2.702925;PLEK|2.071125;TTK|0.488375;PLK1|0.415475;CHAF1B|0.272	regulation of organelle organization	GO:0033043	4.82E-05	6.73E-04	14	5
6,7	ACTG1|2.1999;KRT1|0.4198;ACTB|2.1999;ALB|0.23405	retina homeostasis	GO:0001895	5.46E-05	6.73E-04	4	7
7	HSP90AB1|0.458775;RPL3|3.30745;MCM2|0.491625	cellular response to interleukin-4	GO:0071353	5.49E-05	7.59E-04	3	7
6	HIST1H4A|0.23835;TOP2A|0.481675;PLEK|2.071125;SOS2|11.48525;HEATR9|2.53905	hematopoietic progenitor cell differentiation	GO:0002244	5.73E-05	7.59E-04	5	6
7,8	ACTG1|2.1999;ATP6V1B2|2.0575;SQSTM1|6.007525;MAPK14|0.483325;STAT3|0.4995;TXNIP|2.0553;VTN|2.01125;ARHGDIA|3.8215;ACTB|2.1999;MYH10|5.579275;SOS2|11.48525;PTP4A3|0.3433	transmembrane receptor protein tyrosine kinase signaling pathway	GO:0007169	6.05E-05	7.88E-04	12	8
6	HSPD1|2.88995;HIST1H2BN|0.481625;RPL3|3.30745;MCM2|0.491625;HIST1H4A|0.23835;GSN|2.4555;HIST1H3A|0.455625;TUBG1|0.4102;NDE1|0.446425;RBBP7|0.218475;H3F3A|0.20985;TBCA|0.3515;CHAF1B|0.2723	cellular macromolecular complex assembly	GO:0034622	6.36E-05	8.29E-04	13	6
5	HSPA8|0.3721;ENO1|2.1939;RPL3|3.30745;PABPC1|0.3285;SARS|2.2366;ASPH|2.59965;YARS|2.288275;MCM3|0.35205;RPL4|2.063325;NARS|2.162;MCM2|0.491625;DNMT1|0.172;HIST1H4A|0.23835;YWHAG|2.359475;SRRT|0.39875;CBX3|0.485775;GTF2E1|0.300475;PABPC4|0.26245;LIG1|0.440425;PRDX5|3.0143;BAZ1B|0.460625;RRM2|0.21675;EIF4H|0.275675;MCMBP|0.4769;RPL27|2.15885;SQSTM1|6.007525;ATG7|2.120975;PCNA|0.404975;MAPK14|0.483325;HIST1H3A|0.455625;BTF3|0.2449;RHOG|2.449775;TOP2A|0.481675;SNW1|0.473175;STAT3|0.4995;TXNIP|2.0553;S100A11|2.531875;RBBP7|0.218475;H3F3A|0.20985;PLK1|0.415475;SOD2|2.175425;ZNF706|0.3974	cellular macromolecule biosynthetic process	GO:0034645	6.80E-05	8.67E-04	43	5
4	HSP90AB1|0.458775;ENO1|2.1939;RPL3|3.30745;PABPC1|0.3285;RPL4|2.063325;NSF|2.017425;ALDH1L2|2.148;PABPC4|0.26245;LIG1|0.440425;ESD|2.155525;ALDOC|2.04725;PSME3|0.401575;RPL27|2.15885;SQSTM1|6.007525;ATG7|2.120975;STAT3|0.4995;PLBD2|2.666725;TTK|0.488375;ASL|2.252975;PLK1|0.415475	organic substance catabolic process	GO:1901575	7.26E-05	9.23E-04	20	4
4	ATP6V1B2|2.0575;GSN|2.4555;ATG7|2.120975;ALB|0.23405;IFITM2|5.68315	interaction with host	GO:0051701	7.34E-05	9.76E-04	5	4
5,6	ANXA5|3.637725;SQSTM1|6.007525;ATG7|2.120975;SNW1|0.473175;CHAF1B|0.2723	positive regulation of response to extracellular stimulus	GO:0032106	7.34E-05	9.76E-04	5	6
6,7	ANXA5|3.637725;SQSTM1|6.007525;ATG7|2.120975;SNW1|0.473175;CHAF1B|0.2723	positive regulation of response to nutrient levels	GO:0032109	7.34E-05	9.76E-04	5	7
7,8	DNMT1|0.172;HIST1H4A|0.23835;HIST1H3A|0.455625;H3F3A|0.20985	DNA methylation or demethylation	GO:0044728	7.38E-05	9.76E-04	4	8
7	HSPA8|0.3721;RPL3|3.30745;ASPH|2.59965;RPL4|2.063325;GSN|2.4555;RPL27|2.15885;PLEK|2.071125	cellular protein complex disassembly	GO:0043624	7.61E-05	9.77E-04	7	7
7,8	HSPA8|0.3721;ACTG1|2.1999;HSP90AB1|0.458775;ATG7|2.120975;RHOG|2.449775;ARHGDIA|3.8215;ACTB|2.1999;MYH10|5.579275;SOS2|11.48525	axonogenesis	GO:0007409	7.75E-05	1.00E-03	9	8
4,5	ACTG1|2.1999;HSP90AB1|0.458775;LCP1|2.170475;HSPD1|2.88995;VIM|2.241075;ASPH|2.59965;YARS|2.288275;ATP6V1B2|2.0575;HIST1H4A|0.23835;KPNA2|0.186675;YWHAG|2.359475;ANXA5|3.637725;GSN|2.4555;AKR1B1|2.235325;G3BP1|0.3904;ERO1L|2.370575;BAZ1B|0.460625;PSME3|0.401575;LAMTOR2|7.841025;SQSTM1|6.007525;MAPK14|0.483325;HIST1H3A|0.455625;RHOG|2.449775;SNW1|0.473175;STAT3|0.4995;TXNIP|2.0553;IPO8|0.443925;PLEK|2.071125;NDE1|0.446425;S100A11|2.531875;NDRG1|10.0073;VTN|2.01125;ARHGDIA|3.8215;ACTB|2.1999;H3F3A|0.20985;MYH10|5.579275;TTK|0.488375;SOS2|11.48525;PLK1|0.415475;SOD2|2.175425;PTP4A3	signal transduction	GO:0007165	7.83E-05	1.02E-03	43	5
5	HSPA8|0.3721;ENO1|2.1939;HSPD1|2.88995;RPL3|3.30745;PABPC1|0.3285;SARS|2.2366;ASPH|2.59965;YARS|2.288275;MCM3|0.35205;RPL4|2.063325;NARS|2.162;MCM2|0.491625;DNMT1|0.172;HIST1H4A|0.23835;KPNA2|0.186675;YWHAG|2.359475;SRRT|0.39875;CBX3|0.485775;GTF2E1|0.300475;PABPC4|0.26245;LIG1|0.440425;G3BP1|0.3904;PRDX5|3.0143;BAZ1B|0.460625;RRM2|0.21675;MCMBP|0.4769;RPL27|2.15885;SQSTM1|6.007525;ATG7|2.120975;PCNA|0.404975;MAPK14|0.483325;HIST1H3A|0.455625;BTF3|0.2449;RHOG|2.449775;TOP2A|0.481675;SNW1|0.473175;STAT3|0.4995;TXNIP|2.0553;S100A11|2.531875;RBBP7|0.218475;H3F3A|0.20985;PLK1|0.415475;	nucleic acid metabolic process	GO:0090304	8.05E-05	1.02E-03	45	5

5	HSPA8|0.3721;ENO1|2.1939;HSPD1|2.88995;RPL3|3.30745;VIM|2.241075;PABPC1|0.3285;SARS|2.2366;ASPH|2.59965;YARS|2.288275;RPL4|2.063325;NARS|2.162;DNMT1|0.172;HIST1H4A|0.23835;YWHAG|2.359475;SRRT|0.39875;GSN|2.4555;CBX3|0.485775;GTF2E1|0.300475;PABPC4|0.26245;ERO1L|2.370575;PRDX5|3.0143;BAZ1B|0.460625;PSME3|0.401575;RRM2|0.21675;EIF4H|0.275675;RPL27|2.15885;SQSTM1|6.007525;PCNA|0.404975;MAPK14|0.483325;HIST1H3A|0.455625;BTF3|0.2449;RHOG|2.449775;TOP2A|0.481675;SNW1|0.473175;STAT3|0.4995;TXNIP|2.0553;IPO8|0.443925;VTN|2.01125;RBBP7|0.218475;H3F3A|0.20985;PLK1|0.415475;SOD2|2.175425;ZNF706|0	gene expression	GO:0010467	8.20E-05	1.05E-03	45	5
6,7	HSPD1|2.88995;YWHAG|2.359475;ANXA5|3.637725;GSN|2.4555;SQSTM1|6.007525;ATG7|2.120975;TOP2A|0.481675;TXNIP|2.0553;SOS2|11.48525	positive regulation of apoptotic process	GO:0043065	8.70E-05	1.06E-03	9	7
4,5	PABPC1|0.3285;NSF|2.017425;ANXA5|3.637725;PSME3|0.401575;SQSTM1|6.007525;ATG7|2.120975;PLK1|0.415475;CHAF1B|0.2723	positive regulation of catabolic process	GO:0009896	8.79E-05	1.12E-03	8	5
4	ENO1|2.1939;GMPS|0.496875;MTHFD1L|6.667175;CNDP2|2.0938;AKR1B1|2.235325;LIG1|0.440425;SLC25A1|2.1121;PRDX5|3.0143;ISYNA1|0.49065;ALDOC|2.04725;RRM2|0.21675;PCNA|0.404975;STAT3|0.4995;SLC2A1|5.081875;PLEK|2.071125;ASL|2.252975;GCH1|4.01735;PLP1|	single-organism biosynthetic process	GO:0044711	8.82E-05	1.12E-03	18	4
5,6	HSPD1|2.88995;YWHAG|2.359475;ANXA5|3.637725;GSN|2.4555;SQSTM1|6.007525;ATG7|2.120975;TOP2A|0.481675;TXNIP|2.0553;SOS2|11.48525	positive regulation of programmed cell death	GO:0043068	9.21E-05	1.12E-03	9	6
4	HSP90AB1|0.458775;RPL3|3.30745;PABPC1|0.3285;RPL4|2.063325;ALDH1L2|2.148;ANXA5|3.637725;PABPC4|0.26245;LIG1|0.440425;PRDX5|3.0143;ESD|2.155525;PSME3|0.401575;RPL27|2.15885;SQSTM1|6.007525;ATG7|2.120975;STAT3|0.4995;HBA1|2.13735;TTK|0.488375;ASL|2.252975;PLK1|0.415475;CHAF1B|0.2723	cellular catabolic process	GO:0044248	9.31E-05	1.17E-03	20	4
4	SMC2|0.485025;NCAPG|0.4528;MCMBP|0.4769;TOP2A|0.481675;NDE1|0.446425;TTK|0.488375;PLK1|0.415475	chromosome segregation	GO:0007059	9.57E-05	1.18E-03	7	4
5,6	VIM|2.241075;GSN|2.4555;NDRG1|10.0073;PLP1|3.936	glial cell development	GO:0021782	9.75E-05	1.20E-03	4	6
6	HSP90AB1|0.458775;RPL3|3.30745;MCM2|0.491625	response to interleukin-4	GO:0070670	1.01E-04	1.22E-03	3	6
4	HSPA8|0.3721;VIM|2.241075;DNMT1|0.172;GSN|2.4555;ATG7|2.120975;VAT1|2.702925;ARHGDIA|3.8215;TTK|0.488375;PLK1|0.415475	negative regulation of cellular component organization	GO:0051129	1.04E-04	1.26E-03	9	4
5	ACTG1|2.1999;MAPK14|0.483325;RHOG|2.449775;PLEK|2.071125;ACTB|2.1999;ALB|0.23405	platelet activation	GO:0030168	1.07E-04	1.29E-03	6	5
2	HSPA8|0.3721;ACTG1|2.1999;HSP90AB1|0.458775;LCP1|2.170475;ENO1|2.1939;HSPD1|2.88995;VIM|2.241075;PABPC1|0.3285;ASPH|2.59965;KRT1|0.4198;YARS|2.288275;ATP6V1B2|2.0575;NSF|2.017425;CKB|0.486625;DNMT1|0.172;HIST1H4A|0.23835;KPNA2|0.186675;VDAC3|2.015475;YWHAG|2.359475;ANXA5|3.637725;SRRT|0.39875;GSN|2.4555;CBX3|0.485775;GTF2E1|0.300475;AKR1B1|2.235325;PABPC4|0.26245;LIG1|0.440425;G3BP1|0.3904;ERO1L|2.370575;PRDX5|3.0143;BAZ1B|0.460625;PSME3|0.401575;RRM2|0.21675;EIF4H|0.275675;LAMTOR2|7.841025;SQSTM1|6.007525;ATG7|2.120975;PCNA|0.404975;MAPK14|0.483325;HIST1H3A|0.455625;BTF3|0.2449;TFRC|0.403125;RHOG|2.449775;TOP2A|0.481675;SNW1|0.473175;STAT3|0.4995;NCDN|0.4114;SLC2A1|5.081875;TXNIP|2.0553;VAT1|2.702925;IPO8|0.443925;PLEK|2.071125;NDE1|0.446425;S100A11|2.531875;HBA1|2.13735;NDRG1|10.0073;VTN|2.01125;RBBP7|0.218475;ARHGDIA|3.8215;ACTB|2.1999;H3F3A|0.20985;MYH10|5.579275;TTK|0.488375;SOS2|11.48525;ALB|0.23405;CDC123|0.29925;PLK1|0.415475;GCH1|4.01735;SOD2|2.175425;PTP4A[illegible]	biological regulation	GO:0065007	1.08E-04	1.33E-03	74	2
4,5	HSPA8|0.3721;HSP90AB1|0.458775;ENO1|2.1939;HSPD1|2.88995;VIM|2.241075;PABPC1|0.3285;ASPH|2.59965;NSF|2.017425;DNMT1|0.172;HIST1H4A|0.23835;KPNA2|0.186675;YWHAG|2.359475;SRRT|0.39875;GSN|2.4555;CBX3|0.485775;GTF2E1|0.300475;PRDX5|3.0143;BAZ1B|0.460625;PSME3|0.401575;RRM2|0.21675;EIF4H|0.275675;LAMTOR2|7.841025;SQSTM1|6.007525;ATG7|2.120975;PCNA|0.404975;MAPK14|0.483325;HIST1H3A|0.455625;BTF3|0.2449;RHOG|2.449775;TOP2A|0.481675;SNW1|0.473175;STAT3|0.4995;TXNIP|2.0553;PLEK|2.071125;S100A11|2.531875;VTN|2.01125;RBBP7|0.218475;H3F3A|0.20985;TTK|0.488375;PLK1|0.415475;SOD2|2.175425;ZNF706	regulation of macromolecule metabolic process	GO:0060255	1.10E-04	1.33E-03	44	5
4,6	KRT1|0.4198;ANXA5|3.637725;PLEK|2.071125;VTN|2.01	regulation of hemostasis	GO:1900046	1.11E-04	1.34E-03	4	6
5,6,7	KRT1|0.4198;ANXA5|3.637725;PLEK|2.071125;VTN|2.01125	regulation of blood coagulation	GO:0030193	1.11E-04	1.34E-03	4	7
5	ENO1|2.1939;MTHFD1L|6.667175;ALDH1L2|2.148;SLC25A1|2.1121;PRDX5|3.0143;ALDOC|2.04725;STAT3|0.4995;GCH1|4.01735	coenzyme metabolic process	GO:0006732	1.11E-04	1.34E-03	8	5
6	HSPA8|0.3721;RPL3|3.30745;ASPH|2.59965;RPL4|2.063325;GSN|2.4555;RPL27|2.15885;PLEK|2.071125	protein complex disassembly	GO:0043241	1.12E-04	1.34E-03	7	6
5	HSP90AB1|0.458775;RPL3|3.30745;ATP6V1B2|2.0575;MCM2|0.491625;DNMT1|0.172;KPNA2|0.186675;SRRT|0.39875;AKR1B1|2.235325;ERO1L|2.370575;LAMTOR2|7.841025;SQSTM1|6.007525;MAPK14|0.483325;SNW1|0.473175;STAT3|0.4995;VTN|2.01125;ARHGDIA|3.8215;SOS2|11.48525;PTP4A3|0.3433;IFITM2|5.68315	cellular response to organic substance	GO:0071310	1.16E-04	1.35E-03	19	5

4,5	YWHAG|2.359475;ANXA5|3.637725;GSN|2.4555;AKR1B1|2.235325;PSME3|0.401575;LAMTOR2|7.841025;SQSTM1|6.007525;ATG7|2.120975;MAPK14|0.483325;SNW1|0.473175;STAT3|0.4995;VTN|2.01125;TTK|0.488375;SOS2|11.48525;CHAF1B|0.2723	positive regulation of cell communication	GO:0010647	1.19E-04	1.39E-03	15	5
3	HSPA8|0.3721;ACTG1|2.1999;HSP90AB1|0.458775;LCP1|2.170475;ENO1|2.1939;HSPD1|2.88995;VIM|2.241075;PABPC1|0.3285;ASPH|2.59965;YARS|2.288275;ATP6V1B2|2.0575;NSF|2.017425;DNMT1|0.172;HIST1H4A|0.23835;KPNA2|0.186675;VDAC3|2.015475;YWHAG|2.359475;ANXA5|3.637725;SRRT|0.39875;GSN|2.4555;CBX3|0.485775;GTF2E1|0.300475;AKR1B1|2.235325;G3BP1|0.3904;ERO1L|2.370575;PRDX5|3.0143;BAZ1B|0.460625;PSME3|0.401575;RRM2|0.21675;EIF4H|0.275675;LAMTOR2|7.841025;SQSTM1|6.007525;ATG7|2.120975;PCNA|0.404975;MAPK14|0.483325;HIST1H3A|0.455625;BTF3|0.2449;TFRC|0.403125;RHOG|2.449775;TOP2A|0.481675;SNW1|0.473175;STAT3|0.4995;NCDN|0.4114;SLC2A1|5.081875;TXNIP|2.0553;VAT1|2.702925;IPO8|0.443925;PLEK|2.071125;NDE1|0.446425;S100A11|2.531875;HBA1|2.13735;NDRG1|10.0073;VTN|2.01125;RBBP7|0.218475;ARHGDIA|3.8215;ACTB|2.1999;H3F3A|0.20985;MYH10|5.579275;TTK|0.488375;SOS2|11.48525;ALB|0.23405;CDC123|0.29925;PLK1|0.415475;SOD2|2.175425;PTP4A3|0.3433;IFITM2|5.6[illegible]	regulation of cellular process	GO:0050794	1.21E-04	1.42E-03	69	3
6,8	HSPA8|0.3721;ACTG1|2.1999;HSP90AB1|0.458775;VIM|2.241075;YWHAG|2.359475;ATG7|2.120975;RHOG|2.449775;STAT3|0.4995;NCDN|0.4114;ARHGDIA|3.8215;ACTB|2.1999;MYH10|5.579275;SOS2|11.48525;PLP1|3.936	neuron differentiation	GO:0030182	1.22E-04	1.44E-03	14	8
5,6	HSP90AB1|0.458775;LCP1|2.170475;RPL3|3.30745;ASPH|2.59965;ATP6V1B2|2.0575;RPL4|2.063325;NSF|2.017425;KPNA2|0.186675;YWHAG|2.359475;RPL27|2.15885;ATG7|2.120975;MAPK14|0.483325;BTF3|0.2449;TFRC|0.403125;STAT3|0.4995;SLC2A1|5.081875;TXNIP|2.0553;IPO8|0.443925;PLEK|2.071125	protein transport	GO:0015031	1.23E-04	1.44E-03	19	6
5	HSPA8|0.3721;ENO1|2.1939;RPL3|3.30745;PABPC1|0.3285;SARS|2.2366;ASPH|2.59965;YARS|2.288275;MCM3|0.35205;RPL4|2.063325;NARS|2.162;MCM2|0.491625;DNMT1|0.172;HIST1H4A|0.23835;YWHAG|2.359475;SRRT|0.39875;CBX3|0.485775;GTF2E1|0.300475;PABPC4|0.26245;LIG1|0.440425;PRDX5|3.0143;BAZ1B|0.460625;RRM2|0.21675;EIF4H|0.275675;MCMBP|0.4769;RPL27|2.15885;SQSTM1|6.007525;ATG7|2.120975;PCNA|0.404975;MAPK14|0.483325;HIST1H3A|0.455625;BTF3|0.2449;RHOG|2.449775;TOP2A|0.481675;SNW1|0.473175;STAT3|0.4995;TXNIP|2.0553;S100A11|2.531875;RBBP7|0.218475;H3F3A|0.20985;PLK1|0.415475;SOD2|2.175425;ZNF706|0.3974	macromolecule biosynthetic process	GO:0009059	1.24E-04	1.44E-03	43	5
4	HSPA8|0.3721;ENO1|2.1939;HSPD1|2.88995;RPL3|3.30745;PABPC1|0.3285;SARS|2.2366;ASPH|2.59965;YARS|2.288275;ATP6V1B2|2.0575;MCM3|0.35205;RPL4|2.063325;GMPS|0.496875;NARS|2.162;MCM2|0.491625;DNMT1|0.172;HIST1H4A|0.23835;KPNA2|0.186675;YWHAG|2.359475;SRRT|0.39875;CBX3|0.485775;GTF2E1|0.300475;PABPC4|0.26245;LIG1|0.440425;G3BP1|0.3904;PRDX5|3.0143;BAZ1B|0.460625;ALDOC|2.04725;RRM2|0.21675;MCMBP|0.4769;RPL27|2.15885;SQSTM1|6.007525;ATG7|2.120975;PCNA|0.404975;MAPK14|0.483325;HIST1H3A|0.455625;BTF3|0.2449;RHOG|2.449775;TOP2A|0.481675;SNW1|0.473175;STAT3|0.4995;TXNIP|2.0553;S100A11|2.531875;RBBP7|0.218475;H3F3A|0.20985;PLK1|0.415475;SO	nucleobase-containing compound metabolic process	GO:0006139	1.28E-04	1.45E-03	48	4
5	VIM|2.241075;CKB|0.486625;GSN|2.4555;ATG7|2.120975;STAT3|0.4995;NDE1|0.446425;VTN|2.01125;ACTB|2.1999;H3F3A|0.20985;MYH10|5.579275;PLP1|3.936	central nervous system development	GO:0007417	1.29E-04	1.49E-03	11	5
3	HSPA8|0.3721;ACTG1|2.1999;HSP90AB1|0.458775;LCP1|2.170475;ASPH|2.59965;KRT1|0.4198;MTHFD1L|6.667175;VDAC3|2.015475;GSN|2.4555;LIG1|0.440425;BAZ1B|0.460625;ATG7|2.120975;MAPK14|0.483325;RHOG|2.449775;STAT3|0.4995;VAT1|2.702925;VTN|2.01125;ARHGDIA|3.8215;ACTB|2.1999;MYH10|5.579275;SOS2|11.48525	anatomical structure morphogenesis	GO:0009653	1.29E-04	1.50E-03	21	3
5	HSPA8|0.3721;RPL3|3.30745;ASPH|2.59965;RPL4|2.063325;GSN|2.4555;RPL27|2.15885;PLEK|2.071125	macromolecular complex disassembly	GO:0032984	1.31E-04	1.50E-03	7	5
3	HSPD1|2.88995;VIM|2.241075;ASPH|2.59965;KRT1|0.4198;HIST1H4A|0.23835;YWHAG|2.359475;ANXA5|3.637725;SRRT|0.39875;PRDX5|3.0143;ATG7|2.120975;MAPK14|0.483325;TFRC|0.403125;SNW1|0.473175;STAT3|0.4995;PLEK|2.071125;VTN|2.01125;ARHGDIA|3.8215;SOS2|11.48525;ZNF706|0.3974	regulation of multicellular organismal process	GO:0051239	1.31E-04	1.50E-03	19	3
6,7,8,9,10	HSPA8|0.3721;PABPC1|0.3285;SNW1|0.473175	positive regulation of mRNA processing	GO:0050685	1.32E-04	1.50E-03	3	10
4,5,6	HSP90AB1|0.458775;ASPH|2.59965;YWHAG|2.359475;GSN|2.4555;AKR1B1|2.235325;G3BP1|0.3904;BAZ1B|0.460625;PSME3|0.401575;LAMTOR2|7.841025;SQSTM1|6.007525;MAPK14|0.483325;RHOG|2.449775;SNW1|0.473175;STAT3|0.4995;PLEK|2.071125;VTN|2.01125;ARHGDIA|3.8215;TTK|0.488375;SOS2|11.48525;SOD2|2.175425;PT	regulation of signal transduction	GO:0009966	1.33E-04	1.50E-03	21	6
5,6	HSPA8|0.3721;ACTG1|2.1999;HSP90AB1|0.458775;VIM|2.241075;ATG7|2.120975;RHOG|2.449775;NCDN|0.4114;ARHGDIA|3.8215;ACTB|2.1999;MYH10|5.579275;SOS2|11.48525;PLP1|3.936	neuron development	GO:0048666	1.35E-04	1.51E-03	12	6

4,5	KRT1|0.4198;ANXA5|3.637725;PLEK|2.071125;VTN|2.01	regulation of coagulation	GO:0050818	1.49E-04	1.53E-03	4	5
6,7	RPL3|3.30745;PABPC1|0.3285;RPL4|2.063325;PABPC4|0.26245;LIG1|0.440425;RPL27|2.15885	RNA catabolic process	GO:0006401	1.58E-04	1.68E-03	6	7
2,3	HSPA8|0.3721;ACTG1|2.1999;HSP90AB1|0.458775;LCP1|2.170475;ENO1|2.1939;HSPD1|2.88995;VIM|2.241075;PABPC1|0.3285;ASPH|2.59965;KRT1|0.4198;YARS|2.288275;ATP6V1B2|2.0575;NSF|2.017425;DNMT1|0.172;HIST1H4A|0.23835;KPNA2|0.186675;VDAC3|2.015475;YWHAG|2.359475;ANXA5|3.637725;SRRT|0.39875;GSN|2.4555;CBX3|0.485775;GTF2E1|0.300475;AKR1B1|2.235325;G3BP1|0.3904;ERO1L|2.370575;PRDX5|3.0143;BAZ1B|0.460625;PSME3|0.401575;RRM2|0.21675;EIF4H|0.275675;LAMTOR2|7.841025;SQSTM1|6.007525;ATG7|2.120975;PCNA|0.404975;MAPK14|0.483325;HIST1H3A|0.455625;BTF3|0.2449;TFRC|0.403125;RHOG|2.449775;TOP2A|0.481675;SNW1|0.473175;STAT3|0.4995;NCDN|0.4114;SLC2A1|5.081875;TXNIP|2.0553;VAT1|2.702925;IPO8|0.443925;PLEK|2.071125;NDE1|0.446425;S100A11|2.531875;HBA1|2.13735;NDRG1|10.0073;VTN|2.01125;RBBP7|0.218475;ARHGDIA|3.8215;ACTB|2.1999;H3F3A|0.20985;MYH10|5.579275;TTK|0.488375;SOS2|11.48525;ALB|0.23405;CDC123|0.29925;PLK1|0.415475;GCH1|4.01735;SOD2|2.175425;PTP4A3|0.3433;IFITM2|5.68315;ZNF706|0.3974;C[illegible]	regulation of biological process	GO:0050789	1.60E-04	1.77E-03	71	3
5	HSPA8|0.3721;HSP90AB1|0.458775;HSPD1|2.88995;HSPA4L|2.13105;ERO1L|2.370575	response to unfolded protein	GO:0006986	1.63E-04	1.79E-03	5	5
4	HSP90AB1|0.458775;RPL3|3.30745;RPL4|2.063325;NSF|2.017425;KPNA2|0.186675;YWHAG|2.359475;GSN|2.4555;RPL27|2.15885;MAPK14|0.483325;RHOG|2.449775;STAT3|0.4995;TXNIP|2.0553;NDE1|0.446425;ALB|0.23405	single-organism cellular localization	GO:1902580	1.70E-04	1.81E-03	14	4
5	HSPD1|2.88995;KRT1|0.4198;ANXA5|3.637725;GSN|2.4555;MAPK14|0.483325;PLEK|2.071125;VTN|2.01125	regulation of response to wounding	GO:1903034	1.70E-04	1.88E-03	7	5
5	GMPS|0.496875;MTHFD1L|6.667175;CKB|0.486625;ALDH1L2|2.148;ERO1L|2.370575;ASL|2.252975;GCH1|4.0173	alpha-amino acid metabolic process	GO:1901605	1.70E-04	1.88E-03	7	5
4	ASPH|2.59965;DNMT1|0.172;AKR1B1|2.235325;MAPK14|0.483325;TFRC|0.403125;RHOG|2.449775;STAT3|0.4995;TXNIP|2.0553;S100A11|2.531875;NDRG1|10.0073;TTK|0.488375;SOS2|11.48525;CDC123|0.29925;SOD2|2.17542	regulation of cell proliferation	GO:0042127	1.72E-04	1.88E-03	14	4
6,7	DNMT1|0.172;HIST1H4A|0.23835;HIST1H3A|0.455625;H3F3A|0.20985	DNA modification	GO:0006304	1.74E-04	1.88E-03	4	7
4	ACTG1|2.1999;LCP1|2.170475;HSPD1|2.88995;GSN|2.4555;MAPK14|0.483325;RHOG|2.449775;PLEK|2.071125;NDRG1|10.0073;ACTB|2.1999;SOS2|11.48525;ALB|0.2340	cell activation	GO:0001775	1.82E-04	1.90E-03	11	4
4	HSP90AB1|0.458775;PABPC1|0.3285;NSF|2.017425;ANXA5|3.637725;PSME3|0.401575;SQSTM1|6.007525;ATG7|2.120975;STAT3|0.4995;TTK|0.488375;PLK1|0.415475;CHAF1B|0.2723	regulation of catabolic process	GO:0009894	1.91E-04	1.98E-03	11	4
7	HIST1H4A|0.23835;RBBP7|0.218475;H3F3A|0.20985	DNA replication-independent nucleosome organization	GO:0034724	1.95E-04	2.07E-03	3	7
7,8	HIST1H4A|0.23835;RBBP7|0.218475;H3F3A|0.20985	DNA replication-independent nucleosome assembly	GO:0006336	1.95E-04	2.09E-03	3	8
4	DNMT1|0.172;GSN|2.4555;AKR1B1|2.235325;LAMTOR2|7.841025;TFRC|0.403125;SNW1|0.473175	response to acid chemical	GO:0001101	1.96E-04	2.09E-03	6	4
2	HSPA8|0.3721;HSP90AB1|0.458775;ENO1|2.1939;HSPD1|2.88995;RPL3|3.30745;VIM|2.241075;CCT7|2.06135;PABPC1|0.3285;SARS|2.2366;ASPH|2.59965;KRT1|0.4198;YARS|2.288275;ATP6V1B2|2.0575;MCM3|0.35205;RPL4|2.063325;NSF|2.017425;GMPS|0.496875;MTHFD1L|6.667175;CKB|0.486625;NARS|2.162;MCM2|0.491625;HSPA4L|2.13105;DNMT1|0.172;ALDH1L2|2.148;HIST1H4A|0.23835;KPNA2|0.186675;YWHAG|2.359475;ANXA5|3.637725;SRRT|0.39875;GSN|2.4555;CNDP2|2.0938;CBX3|0.485775;GTF2E1|0.300475;AKR1B1|2.235325;PABPC4|0.26245;LIG1|0.440425;SLC25A1|2.1121;G3BP1|0.3904;ERO1L|2.370575;PRDX5|3.0143;BAZ1B|0.460625;ESD|2.155525;ISYNA1|0.49065;ALDOC|2.04725;PSME3|0.401575;RRM2|0.21675;EIF4H|0.275675;ACAT2|2.108325;LAMTOR2|7.841025;MCMBP|0.4769;RPL27|2.15885;SQSTM1|6.007525;ATG7|2.120975;PCNA|0.404975;MAPK14|0.483325;HIST1H3A|0.455625;BTF3|0.2449;RHOG|2.449775;TOP2A|0.481675;SNW1|0.473175;STAT3|0.4995;TUBG1|0.4102;SLC2A1|5.081875;TXNIP|2.0553;VAT1|2.702925;IPO8|0.443925;PLEK|2.071125;S100A11|2.531875;HBA1|2.13735;VTN|2.01125;RBBP7|0.218475;ARHGDIA|3.8215;PLBD2|2.666725;ACTB|2.1999;DNPEP|2.077575;H3F3A|0.20985;MYH10|5.579275;TTK|0.488375;SOS2|11.48525;ASL|2.252975;ALB|0.23405;PLK1|0.415475;GCH1|4.01735;SOD2|2.[illegible]	metabolic process	GO:0008152	1.99E-04	2.10E-03	89	2
4,6	ENO1|2.1939;ANXA5|3.637725;ATG7|2.120975;MAPK14|0.483325;HIST1H3A|0.455625;IFITM2|5.68315;CHAF1B|	response to virus	GO:0009615	2.05E-04	2.12E-03	7	6
4,5	MTHFD1L|6.667175;CKB|0.486625;ALDH1L2|2.148;CNDP2|2.0938;ERO1L|2.370575;GCH1|4.01735	cellular modified amino acid metabolic process	GO:0006575	2.06E-04	2.18E-03	6	5
4	HSPA8|0.3721;HSP90AB1|0.458775;HSPD1|2.88995;HSPA4L|2.13105;ERO1L|2.370575	response to topologically incorrect protein	GO:0035966	2.07E-04	2.18E-03	5	4
6,7	HSPA8|0.3721;ACTG1|2.1999;HSP90AB1|0.458775;ATG7|2.120975;RHOG|2.449775;ARHGDIA|3.8215;ACTB|2.1999;MYH10|5.579275;SOS2|11.48525	cell morphogenesis involved in neuron differentiation	GO:0048667	2.16E-04	2.19E-03	9	7

5,6	SMC2|0.485025;NCAPG|0.4528;TOP2A|0.481675;TTK|0.488375;PLK1|0.415475	sister chromatid segregation	GO:0000819	2.17E-04	2.27E-03	5	6
5	PRDX5|3.0143;STAT3|0.4995;HBA1|2.13735	hydrogen peroxide metabolic process	GO:0042743	2.24E-04	2.27E-03	3	5
7	AKR1B1|2.235325;ISYNA1|0.49065;PLEK|2.071125	polyol biosynthetic	GO:0046173	2.24E-04	2.33E-03	3	7
8	MCM3|0.35205;MCM2|0.491625;G3BP1|0.3904;TOP2A|0.481675	DNA duplex unwinding	GO:0032508	2.33E-04	2.33E-03	4	8
5	HSP90AB1|0.458775;LCP1|2.170475;RPL3|3.30745;ASPH|2.59965;RPL4|2.063325;NSF|2.017425;KPNA2|0.186675;YWHAG|2.359475;GSN|2.4555;LAMTOR2|7.841025;RPL27|2.15885;MAPK14|0.483325;RHOG|2.449775;STAT3|0.4995;TXNIP|2.0553;IPO8|0.443925;PLK1|0.415475	cellular protein localization	GO:0034613	2.37E-04	2.41E-03	17	5
5	HSPA8|0.3721;ENO1|2.1939;RPL3|3.30745;SARS|2.2366;ASPH|2.59965;RPL4|2.063325;GMPS|0.496875;MTHFD1L|6.667175;DNMT1|0.172;HIST1H4A|0.23835;YWHAG|2.359475;SRRT|0.39875;CBX3|0.485775;GTF2E1|0.300475;AKR1B1|2.235325;LIG1|0.440425;PRDX5|3.0143;BAZ1B|0.460625;RRM2|0.21675;RPL27|2.15885;SQSTM1|6.007525;ATG7|2.120975;PCNA|0.404975;MAPK14|0.483325;HIST1H3A|0.455625;BTF3|0.2449;RHOG|2.449775;TOP2A|0.481675;SNW1|0.473175;STAT3|0.4995;TXNIP|2.0553;RBBP7|0.218475;H3F3A|0.20985;PLK1|0.415475;GCH1|4.01735;SOD2|2.175425;ZNF706|0.3974;CHAF1B|0.2723	organic cyclic compound biosynthetic process	GO:1901362	2.46E-04	2.45E-03	38	5
5	HSP90AB1|0.458775;HSPD1|2.88995;ASPH|2.59965;SRRT|0.39875;GSN|2.4555;SNW1|0.473175;STAT3|0.4995;TXNIP|2.0553;RBBP7|0.218475;ARHGDIA|3.8215	response to organic cyclic compound	GO:0014070	2.48E-04	2.53E-03	10	5
6	ACTG1|2.1999;ASPH|2.59965;ANXA5|3.637725;TXNIP|2.0553	response to calcium ion	GO:0051592	2.50E-04	2.54E-03	4	6
4	HSP90AB1|0.458775;LCP1|2.170475;RPL3|3.30745;ASPH|2.59965;RPL4|2.063325;NSF|2.017425;KPNA2|0.186675;YWHAG|2.359475;GSN|2.4555;LAMTOR2|7.841025;RPL27|2.15885;MAPK14|0.483325;RHOG|2.449775;STAT3|0.4995;TXNIP|2.0553;IPO8|0.443925;PLK1|0.415475	cellular macromolecule localization	GO:0070727	2.50E-04	2.54E-03	17	4
4	HSPA8|0.3721;ACTG1|2.1999;HSP90AB1|0.458775;LCP1|2.170475;VIM|2.241075;ANXA5|3.637725;GSN|2.4555;MAPK14|0.483325;RHOG|2.449775;STAT3|0.4995;NDE1|0.446425;VTN|2.01125;ARHGDIA|3.8215;ACTB|2.1999;MYH10|5.579275;SOS2|11.48525;PTP4A3|0.3433	movement of cell or subcellular component	GO:0006928	2.52E-04	2.54E-03	17	4
5,6,7	HSP90AB1|0.458775;RPL3|3.30745;RPL4|2.063325;KPNA2|0.186675;YWHAG|2.359475;RPL27|2.15885;MAPK14|0.483325;STAT3|0.4995;TXNIP|2.0553	establishment of protein localization to organelle	GO:0072594	2.55E-04	2.55E-03	9	7
5	CKB|0.486625;ACTB|2.1999;PLP1|3.936	substantia nigra development	GO:0021762	2.57E-04	2.57E-03	3	5
6,7,8	HSPA8|0.3721;PABPC1|0.3285;SNW1|0.473175	positive regulation of mRNA metabolic process	GO:1903313	2.57E-04	2.57E-03	3	8
6,8	HSPA8|0.3721;ACTG1|2.1999;HSP90AB1|0.458775;RHOG|2.449775;ACTB|2.1999;MYH10|5.579275;SOS2|11.4852	axon guidance	GO:0007411	2.68E-04	2.57E-03	7	8
5	PSME3|0.401575;MAPK14|0.483325;TOP2A|0.481675;NDRG1|10.0073;TTK|0.488375;PLK1|0.415475	cell cycle checkpoint	GO:0000075	2.71E-04	2.68E-03	6	5
5,7	HSPA8|0.3721;ACTG1|2.1999;HSP90AB1|0.458775;RHOG|2.449775;ACTB|2.1999;MYH10|5.579275;SOS2|11.4852	neuron projection guidance	GO:0097485	2.72E-04	2.69E-03	7	7
6,7	HSP90AB1|0.458775;DNMT1|0.172;PSME3|0.401575;LAMTOR2|7.841025;SQSTM1|6.007525;ATG7|2.120975;MAPK14|0.483325;SNW1|0.473175;PLEK|2.071125;VTN|2.01125;TTK|0.488375;PLK1|0.415475	positive regulation of protein modification process	GO:0031401	2.73E-04	2.69E-03	12	7
3	ACTG1|2.1999;HSP90AB1|0.458775;HSPD1|2.88995;KRT1|0.4198;HIST1H4A|0.23835;ANXA5|3.637725;GSN|2.4555;PSME3|0.401575;MAPK14|0.483325;HIST1H3A|0.455625;VTN|2.01125;ACTB|2.1999;SOS2|11.48525;CHAF1B|0	regulation of immune system process	GO:0002682	2.76E-04	2.70E-03	14	3
7,8	HIST1H4A|0.23835;RRM2|0.21675;HIST1H3A|0.455625;SOD2|2.175425	protein tetramerization	GO:0051262	2.76E-04	2.70E-03	4	8
7	MCM3|0.35205;MCM2|0.491625;G3BP1|0.3904;TOP2A|0.481675	DNA geometric change	GO:0032392	2.76E-04	2.70E-03	4	7
5	PABPC1|0.3285;ANXA5|3.637725;PSME3|0.401575;SQSTM1|6.007525;ATG7|2.120975;PLK1|0.415475;CHAF1B|0	positive regulation of cellular catabolic process	GO:0031331	2.79E-04	2.70E-03	7	5
5	RPL3|3.30745;PABPC1|0.3285;RPL4|2.063325;ALDH1L2|2.148;PABPC4|0.26245;LIG1|0.440425;RPL27|2.15885;STAT3|0.4995	cellular nitrogen compound catabolic process	GO:0044270	2.80E-04	2.71E-03	8	5
3	HSPA8|0.3721;HSP90AB1|0.458775;LCP1|2.170475;RPL3|3.30745;ASPH|2.59965;RPL4|2.063325;NSF|2.017425;KPNA2|0.186675;YWHAG|2.359475;GSN|2.4555;ERO1L|2.370575;LAMTOR2|7.841025;RPL27|2.15885;SQSTM1|6.007525;MAPK14|0.483325;RHOG|2.449775;STAT3|0.4995;SLC2A1|5.081875;TXNIP|2.0553;IPO8|0.443925;PLEK|2.071125;NDE1|0.446425;MYH10|5.579275;ALB|0.23405;PL	cellular localization	GO:0051641	2.86E-04	2.72E-03	25	3
5	RPL3|3.30745;PABPC1|0.3285;RPL4|2.063325;ALDH1L2|2.148;PABPC4|0.26245;LIG1|0.440425;RPL27|2.15885;STAT3|0.4995	heterocycle catabolic process	GO:0046700	2.99E-04	2.76E-03	8	5
3	ENO1|2.1939;HSPD1|2.88995;ANXA5|3.637725;ATG7|2.120975;MAPK14|0.483325;HIST1H3A|0.455625;TXNIP|2.0553;GCH1|4.01735;IFITM2|5.68315;CHAF1B|0.2723	response to biotic stimulus	GO:0009607	3.04E-04	2.88E-03	10	3
5	RPL3|3.30745;PABPC1|0.3285;RPL4|2.063325;ALDH1L2|2.148;PABPC4|0.26245;LIG1|0.440425;RPL27|2.15885;STAT3|0.4995	aromatic compound catabolic process	GO:0019439	3.26E-04	2.91E-03	8	5
5,6,7,8	LCP1|2.170475;GSN|2.4555	regulation of podosome assembly	GO:0071801	3.26E-04	3.11E-03	2	8
6	YWHAG|2.359475;ANXA5|3.637725;SQSTM1|6.007525;ATG7|2.120975;VAT1|2.702925;CHAF1B|0.2723	regulation of mitochondrion	GO:0010821	3.39E-04	3.11E-03	6	6

5	HSPA8|0.3721;ENO1|2.1939;RPL3|3.30745;SARS|2.2366;ASPH|2.59965;RPL4|2.063325;GMPS|0.496875;MTHFD1L|6.667175;DNMT1|0.172;HIST1H4A|0.23835;YWHAG|2.359475;SRRT|0.39875;CBX3|0.485775;GTF2E1|0.300475;LIG1|0.440425;PRDX5|3.0143;BAZ1B|0.460625;RRM2|0.21675;RPL27|2.15885;SQSTM1|6.007525;ATG7|2.120975;PCNA|0.404975;MAPK14|0.483325;HIST1H3A|0.455625;BTF3|0.2449;RHOG|2.449775;TOP2A|0.481675;SNW1|0.473175;STAT3|0.4995;TXNIP|2.0553;RBBP7|0.218475;H3F3A|0.20985;PLK1|0.415475;GCH1|4.01735;SOD2|2.1754	aromatic compound biosynthetic process	GO:0019438	3.41E-04	3.22E-03	37	5
6	HSP90AB1|0.458775;RPL3|3.30745;RPL4|2.063325;KPNA2|0.186675;YWHAG|2.359475;RPL27|2.15885;MAPK14|0.483325;STAT3|0.4995;TXNIP|2.0553;PLK1|0.415475	protein localization to organelle	GO:0033365	3.43E-04	3.23E-03	10	6
3,4	HSPA8|0.3721;HSP90AB1|0.458775;ENO1|2.1939;HSPD1|2.88995;VIM|2.241075;PABPC1|0.3285;ASPH|2.59965;NSF|2.017425;DNMT1|0.172;HIST1H4A|0.23835;KPNA2|0.186675;YWHAG|2.359475;ANXA5|3.637725;SRRT|0.39875;GSN|2.4555;CBX3|0.485775;GTF2E1|0.300475;PRDX5|3.0143;BAZ1B|0.460625;PSME3|0.401575;RRM2|0.21675;EIF4H|0.275675;LAMTOR2|7.841025;SQSTM1|6.007525;ATG7|2.120975;PCNA|0.404975;MAPK14|0.483325;HIST1H3A|0.455625;BTF3|0.2449;RHOG|2.449775;TOP2A|0.481675;SNW1|0.473175;STAT3|0.4995;TXNIP|2.0553;PLEK|2.071125;S100A11|2.531875;VTN|2.01125;RBBP7|0.218475;ARHGDIA|3.8215;H3F3A|0.20985;TTK|0.488375;SOS2|11.48525;PLK1|0.415475;GCH1|4.01735;SOD2|2.175	regulation of metabolic process	GO:0019222	3.46E-04	3.23E-03	48	4
4,5,7	KRT1|0.4198;ANXA5|3.637725;VTN|2.01125	negative regulation of hemostasis	GO:1900047	3.51E-04	3.25E-03	3	7
5,6,7,8	KRT1|0.4198;ANXA5|3.637725;VTN|2.01125	negative regulation of blood coagulation	GO:0030195	3.51E-04	3.26E-03	3	8
5,6	SMC2|0.485025;NCAPG|0.4528;TOP2A|0.481675	chromosome condensation	GO:0030261	3.51E-04	3.26E-03	3	6
5,6	MTHFD1L|6.667175;ALDH1L2|2.148;GCH1|4.01735	folic acid-containing compound metabolic process	GO:0006760	3.51E-04	3.26E-03	3	6
5	HSP90AB1|0.458775;PABPC1|0.3285;ANXA5|3.637725;PSME3|0.401575;SQSTM1|6.007525;ATG7|2.120975;STAT3|0.4995;TTK|0.488375;PLK1|0.415475;CHAF1B|0.2723	regulation of cellular catabolic process	GO:0031329	3.54E-04	3.26E-03	10	5
7	ENO1|2.1939;AKR1B1|2.235325;SLC25A1|2.1121;ALDOC|2.04725	hexose biosynthetic process	GO:0019319	3.58E-04	3.27E-03	4	7
5	ANXA5|3.637725;GSN|2.4555;SQSTM1|6.007525;ATG7|2.120975;TFRC|0.403125;SNW1|0.473175;SLC2A1|5.081875;ALB|0.23405;CHAF1B|0.2723	response to nutrient levels	GO:0031667	3.66E-04	3.29E-03	9	5
9,10	RPL3|3.30745;PABPC1|0.3285;RPL4|2.063325;RPL27|2.15885	nuclear-transcribed mRNA catabolic process, nonsense-mediated decay	GO:0000184	3.69E-04	3.36E-03	4	10
5	HSPA8|0.3721;ENO1|2.1939;RPL3|3.30745;SARS|2.2366;ASPH|2.59965;RPL4|2.063325;GMPS|0.496875;MTHFD1L|6.667175;DNMT1|0.172;HIST1H4A|0.23835;YWHAG|2.359475;SRRT|0.39875;CBX3|0.485775;GTF2E1|0.300475;LIG1|0.440425;PRDX5|3.0143;BAZ1B|0.460625;RRM2|0.21675;RPL27|2.15885;SQSTM1|6.007525;ATG7|2.120975;PCNA|0.404975;MAPK14|0.483325;HIST1H3A|0.455625;BTF3|0.2449;RHOG|2.449775;TOP2A|0.481675;SNW1|0.473175;STAT3|0.4995;TXNIP|2.0553;RBBP7|0.218475;H3F3A|0.20985;PLK1|0.415475;GCH1|4.01735;SOD2|2.1754	heterocycle biosynthetic process	GO:0018130	3.71E-04	3.37E-03	37	5
4,5	GSN|2.4555;SQSTM1|6.007525;TOP2A|0.481675;SNW1|0.473175;IFITM2|5.68315	regulation of symbiosis, encompassing mutualism through parasitism	GO:0043903	3.83E-04	3.38E-03	5	5
6	HSPA8|0.3721;ACTG1|2.1999;HSP90AB1|0.458775;ATG7|2.120975;RHOG|2.449775;ARHGDIA|3.8215;ACTB|2.1999;MYH10|5.579275;SOS2|11.48525	neuron projection morphogenesis	GO:0048812	3.97E-04	3.48E-03	9	6
5	HSP90AB1|0.458775;HSPD1|2.88995;KRT1|0.4198;ANXA5|3.637725;PSME3|0.401575;MAPK14|0.483325;HIST1H3A|0.455625;VTN|2.01125;CHAF1B|0.2723	regulation of defense response	GO:0031347	3.97E-04	3.58E-03	9	5
5	HSP90AB1|0.458775;HSPD1|2.88995;PABPC1|0.3285;ASPH|2.59965;NSF|2.017425;DNMT1|0.172;YWHAG|2.359475;GSN|2.4555;PRDX5|3.0143;PSME3|0.401575;EIF4H|0.275675;LAMTOR2|7.841025;SQSTM1|6.007525;ATG7|2.120975;MAPK14|0.483325;SNW1|0.473175;PLEK|2.071125;VTN|2.01125;TTK|0.488375;PLK1|0.415475;ZNF706|0.	regulation of protein metabolic process	GO:0051246	4.05E-04	3.58E-03	21	5
4,5	HSPA8|0.3721;HSP90AB1|0.458775;ENO1|2.1939;HSPD1|2.88995;PABPC1|0.3285;ASPH|2.59965;NSF|2.017425;DNMT1|0.172;HIST1H4A|0.23835;KPNA2|0.186675;YWHAG|2.359475;ANXA5|3.637725;SRRT|0.39875;GSN|2.4555;CBX3|0.485775;GTF2E1|0.300475;PRDX5|3.0143;BAZ1B|0.460625;PSME3|0.401575;RRM2|0.21675;EIF4H|0.275675;LAMTOR2|7.841025;SQSTM1|6.007525;ATG7|2.120975;PCNA|0.404975;MAPK14|0.483325;HIST1H3A|0.455625;BTF3|0.2449;RHOG|2.449775;TOP2A|0.481675;SNW1|0.473175;STAT3|0.4995;TXNIP|2.0553;PLEK|2.071125;S100A11|2.531875;VTN|2.01125;RBBP7|0.218475;H3F3A|0.20985;TTK|0.488375;PLK1|0.415475;SOD2|2.175425;Z	regulation of cellular metabolic process	GO:0031323	4.28E-04	3.64E-03	43	5
4	ANXA5|3.637725;SQSTM1|6.007525;ATG7|2.120975;SNW1|0.473175;SLC2A1|5.081875;ARHGDIA|3.8215;ALB|0.23405;CHAF1B|0.2723	cellular response to external stimulus	GO:0071496	4.35E-04	3.84E-03	8	4
5	ANXA5|3.637725;SQSTM1|6.007525;ATG7|2.120975;SNW1|0.473175;CHAF1B|0.2723	regulation of response to extracellular stimulus	GO:0032104	4.41E-04	3.89E-03	5	5

6	ANXA5|3.637725;SQSTM1|6.007525;ATG7|2.120975;SNW1|0.473175;CHAF1B|0.2723	regulation of response to nutrient levels	GO:0032107	4.41E-04	3.90E-03	5	6
6,7	VIM|2.241075;STAT3|0.4995;PLP1|3.936	astrocyte differentiation	GO:0048708	4.41E-04	3.90E-03	3	7
6	ENO1|2.1939;AKR1B1|2.235325;SLC25A1|2.1121;ALDOC|2.04725	monosaccharide biosynthetic process	GO:0046364	4.42E-04	3.90E-03	4	6
5,6	HSP90AB1|0.458775;LCP1|2.170475;ASPH|2.59965;YWHAG|2.359475;GSN|2.4555;MAPK14|0.483325;RHOG|2.44	regulation of cellular protein localization	GO:1903827	4.64E-04	3.90E-03	7	6
4	KRT1|0.4198;ANXA5|3.637725;VTN|2.01125	negative regulation of coagulation	GO:0050819	4.66E-04	4.08E-03	3	4
4	ANXA5|3.637725;GSN|2.4555;SQSTM1|6.007525;ATG7|2.120975;TFRC|0.403125;SNW1|0.473175;SLC2A1|5.081875;ALB|0.23405;CHAF1B|0.2723	response to extracellular stimulus	GO:0009991	4.77E-04	4.08E-03	9	4
5	HSPA8|0.3721;ACTG1|2.1999;HSP90AB1|0.458775;VDAC3|2.015475;GSN|2.4555;ATG7|2.120975;RHOG|2.449775;ARHGDIA|3.8215;ACTB|2.1999;MYH10|5.579275;SOS2|	cell projection morphogenesis	GO:0048858	4.83E-04	4.17E-03	11	5
5	RPL3|3.30745;PABPC1|0.3285;RPL4|2.063325;ALDH1L2|2.148;PABPC4|0.26245;LIG1|0.440425;RPL27|2.15885;STAT3|0.4995	organic cyclic compound catabolic process	GO:1901361	4.95E-04	4.21E-03	8	5
5	HSP90AB1|0.458775;LCP1|2.170475;RPL3|3.30745;ASPH|2.59965;ATP6V1B2|2.0575;RPL4|2.063325;NSF|2.017425;KPNA2|0.186675;VDAC3|2.015475;YWHAG|2.359475;SLC25A1|2.1121;RPL27|2.15885;ATG7|2.120975;MAPK14|0.483325;BTF3|0.2449;TFRC|0.403125;STAT3|0.4995;SLC2A1|5.081875;TXNIP|2.0553;IPO8|0.443925;PLEK|2.071125;HBA1|2.13735;ALB|0.23405	organic substance transport	GO:0071702	5.06E-04	4.30E-03	23	5
4	HSPA8|0.3721;HSP90AB1|0.458775;ENO1|2.1939;HSPD1|2.88995;RPL3|3.30745;VIM|2.241075;CCT7|2.06135;PABPC1|0.3285;SARS|2.2366;ASPH|2.59965;KRT1|0.4198;YARS|2.288275;MCM3|0.35205;RPL4|2.063325;NSF|2.017425;NARS|2.162;MCM2|0.491625;HSPA4L|2.13105;DNMT1|0.172;HIST1H4A|0.23835;KPNA2|0.186675;YWHAG|2.359475;SRRT|0.39875;GSN|2.4555;CNDP2|2.0938;CBX3|0.485775;GTF2E1|0.300475;PABPC4|0.26245;LIG1|0.440425;G3BP1|0.3904;ERO1L|2.370575;PRDX5|3.0143;BAZ1B|0.460625;PSME3|0.401575;RRM2|0.21675;EIF4H|0.275675;LAMTOR2|7.841025;MCMBP|0.4769;RPL27|2.15885;SQSTM1|6.007525;ATG7|2.120975;PCNA|0.404975;MAPK14|0.483325;HIST1H3A|0.455625;BTF3|0.2449;RHOG|2.449775;TOP2A|0.481675;SNW1|0.473175;STAT3|0.4995;TXNIP|2.0553;IPO8|0.443925;PLEK|2.071125;S100A11|2.531875;VTN|2.01125;RBBP7|0.218475;ACTB|2.1999;DNPEP|2.077575;H3F3A|0.20985;TTK|0.488375;ASL|2.252975;ALB|0.23405;PLK1|0.415475;SOD2|2.175425;PTP4A3|0.[illegible]	macromolecule metabolic process	GO:0043170	5.22E-04	4.38E-03	68	4
4,5	LCP1|2.170475;VDAC3|2.015475;GSN|2.4555;PLK1|0.415475	regulation of organelle assembly	GO:1902115	5.39E-04	4.50E-03	4	5
4	HSPA8|0.3721;HSP90AB1|0.458775;ENO1|2.1939;HSPD1|2.88995;PABPC1|0.3285;ASPH|2.59965;NSF|2.017425;DNMT1|0.172;HIST1H4A|0.23835;KPNA2|0.186675;YWHAG|2.359475;SRRT|0.39875;GSN|2.4555;CBX3|0.485775;GTF2E1|0.300475;PRDX5|3.0143;BAZ1B|0.460625;PSME3|0.401575;RRM2|0.21675;EIF4H|0.275675;LAMTOR2|7.841025;SQSTM1|6.007525;ATG7|2.120975;PCNA|0.404975;MAPK14|0.483325;HIST1H3A|0.455625;BTF3|0.2449;RHOG|2.449775;TOP2A|0.481675;SNW1|0.473175;STAT3|0.4995;TXNIP|2.0553;PLEK|2.071125;S100A11|2.531875;VTN|2.01125;RBBP7|0.218475;H3F3A|0.20985;TTK|0.488375;PLK1|0.415475;SOD2|2.175425;ZNF706|0.3974;CH	regulation of primary metabolic process	GO:0080090	5.40E-04	4.63E-03	42	4
2	ENO1|2.1939;GSN|2.4555;EIF4H|0.275675;LAMTOR2|7.841025;SQSTM1|6.007525;MAPK14|0.483325;TFRC|0.403125;STAT3|0.4995;RBBP7|0.218475;H3F3A|0.20985	growth	GO:0040007	5.42E-04	4.63E-03	10	2
5,6	HSP90AB1|0.458775;HSPD1|2.88995;PABPC1|0.3285;ASPH|2.59965;DNMT1|0.172;YWHAG|2.359475;GSN|2.4555;PRDX5|3.0143;PSME3|0.401575;EIF4H|0.275675;LAMTOR2|7.841025;SQSTM1|6.007525;ATG7|2.120975;MAPK14|0.483325;SNW1|0.473175;PLEK|2.071125;VTN|2.01125;TTK|0.488375;PLK1|0.415475;ZNF706|0.3974	regulation of cellular protein metabolic process	GO:0032268	5.44E-04	4.63E-03	20	6
6,7	ACTG1|2.1999;PLEK|2.071125;ACTB|2.1999	platelet aggregation	GO:0070527	5.45E-04	4.63E-03	3	7
5,6	HSPA8|0.3721;ACTG1|2.1999;HSP90AB1|0.458775;VDAC3|2.015475;GSN|2.4555;ATG7|2.120975;RHOG|2.449775;ARHGDIA|3.8215;ACTB|2.1999;MYH10|5.579275;SOS2|	cell part morphogenesis	GO:0032990	5.53E-04	4.63E-03	11	6
4	HSPD1|2.88995;ASPH|2.59965;ATP6V1B2|2.0575;DNMT1|0.172;SRRT|0.39875;GSN|2.4555;AKR1B1|2.235325;LAMTOR2|7.841025;MAPK14|0.483325;STAT3|0.4995	response to nitrogen compound	GO:1901698	5.58E-04	4.68E-03	10	4
4	HSPA8|0.3721;HSP90AB1|0.458775;HSPD1|2.88995;ERO1L|2.370575;RBBP7|0.218475	response to temperature stimulus	GO:0009266	5.65E-04	4.71E-03	5	4
5,6	ACTG1|2.1999;HSP90AB1|0.458775;VIM|2.241075;ATP6V1B2|2.0575;KPNA2|0.186675;G3BP1|0.3904;PSME3|0.401575;SQSTM1|6.007525;MAPK14|0.483325;SNW1|0.473175;STAT3|0.4995;TXNIP|2.0553;PLEK|2.071125;VTN|2.01125;ARHGDIA|3.8215;ACTB|2.1999;MYH10|5.579275;TTK|0.488375;SOS2|11.48525;PTP4A3|0.3433;IFITM2|5.6	cell surface receptor signaling pathway	GO:0007166	5.67E-04	4.75E-03	22	6
5	RPL3|3.30745;PABPC1|0.3285;SARS|2.2366;YARS|2.288275;RPL4|2.063325;NARS|2.162;CNDP2|2.0938;PABPC4|0.26245;EIF4H|0.275675;RPL27|2.15885;DNPEP|2.077575;ZNF706|0.3974	peptide metabolic process	GO:0006518	5.81E-04	4.76E-03	12	5
6,7	HSP90AB1|0.458775;RPL3|3.30745;RPL4|2.063325;KPNA2|0.186675;YWHAG|2.359475;RPL27|2.15885;MAPK14|0.483325;STAT3|0.4995;TXNIP|2.0553	protein targeting	GO:0006605	6.04E-04	4.86E-03	9	7

4	HSPD1|2.88995;ANXA5|3.637725;SQSTM1|6.007525;ATG7|2.120975;SNW1|0.473175;CHAF1B|0.2723	positive regulation of response to external stimulus	GO:0032103	6.11E-04	5.04E-03	6	4
8,9	ACTG1|2.1999;MAPK14|0.483325;VTN|2.01125;ACTB|2.1999	vascular endothelial growth factor receptor signaling pathway	GO:0048010	6.18E-04	5.08E-03	4	9
5	HSPD1|2.88995;PCNA|0.404975;MAPK14|0.483325;TFRC|0.403125;SNW1|0.473175;STAT3|0.4995;TXNIP|2.0553;RBBP7|0.218475;GCH1|4.01735	response to lipid	GO:0033993	6.24E-04	5.12E-03	9	5
6,7	DNMT1|0.172;ATG7|2.120975;TTK|0.488375;PLK1|0.415475	negative regulation of chromosome organization	GO:2001251	6.51E-04	5.16E-03	4	7
8,9	MCM2|0.491625;TOP2A|0.481675	DNA unwinding involved in DNA replication	GO:0006268	6.56E-04	5.37E-03	2	9
6,7,8	VIM|2.241075;PLP1|3.936	astrocyte development	GO:0014002	6.56E-04	5.37E-03	2	8
5	MTHFD1L|6.667175;ALDH1L2|2.148;GCH1|4.01735	pteridine-containing compound metabolic process	GO:0042558	6.62E-04	5.37E-03	3	5
5,7	KRT1|0.4198;ANXA5|3.637725;VTN|2.01125	negative regulation of wound healing	GO:0061045	6.62E-04	5.39E-03	3	7
5	RPL3|3.30745;PABPC1|0.3285;SARS|2.2366;YARS|2.288275;RPL4|2.063325;GMPS|0.496875;MTHFD1L|6.667175;NARS|2.162;CNDP2|2.0938;PABPC4|0.26245;EIF4H|0.275675;RPL27|2.15885;STAT3|0.4995;ASL|2.252975;GCH1|4.01735;ZNF706|0.3974	organonitrogen compound biosynthetic process	GO:1901566	7.07E-04	5.39E-03	16	5
5	YWHAG|2.359475;ANXA5|3.637725;SQSTM1|6.007525;ATG7|2.120975;MAPK14|0.483325;STAT3|0.4995;VAT1|2.702925;SOD2|2.175425;CHAF1B|0.2723	mitochondrion organization	GO:0007005	7.10E-04	5.74E-03	9	5
5,6	HSPA8|0.3721;HSP90AB1|0.458775;RPL3|3.30745;ASPH|2.59965;RPL4|2.063325;NSF|2.017425;KPNA2|0.186675;YWHAG|2.359475;ERO1L|2.370575;RPL27|2.15885;SQSTM1|6.007525;MAPK14|0.483325;STAT3|0.4995;TXNIP|2.0553;NDE1|0.446425	single-organism intracellular transport	GO:1902582	7.15E-04	5.75E-03	15	6
7,8,9,10,11	HSPA8|0.3721;SNW1|0.473175	positive regulation of mRNA splicing, via spliceosome	GO:0048026	7.55E-04	5.77E-03	2	11
7,8,9,11,12	DNMT1|0.172;SNW1|0.473175	positive regulation of histone H3-K4	GO:0051571	7.55E-04	6.04E-03	2	12
4	HSPD1|2.88995;TXNIP|2.0553	response to tumor cell	GO:0002347	7.55E-04	6.04E-03	2	4
5,6,7	YWHAG|2.359475;GSN|2.4555;ERO1L|2.370575;PSME3|0.401575;SQSTM1|6.007525;SNW1|0.473175;SOS2|11.48525;SOD2|2.175425	apoptotic signaling pathway	GO:0097190	7.58E-04	6.04E-03	8	7
6,7	RPL3|3.30745;PABPC1|0.3285;SARS|2.2366;YARS|2.288275;RPL4|2.063325;NARS|2.162;CNDP2|2.0938;PABPC4|0.26245;EIF4H|0.275675;RPL27|2.15885;ZNF706|0.3974	peptide biosynthetic process	GO:0043043	7.62E-04	6.05E-03	11	7
6,7	HSPA8|0.3721;ENO1|2.1939;RPL3|3.30745;SARS|2.2366;ASPH|2.59965;RPL4|2.063325;DNMT1|0.172;HIST1H4A|0.23835;YWHAG|2.359475;SRRT|0.39875;CBX3|0.485775;GTF2E1|0.300475;PRDX5|3.0143;BAZ1B|0.460625;RRM2|0.21675;RPL27|2.15885;SQSTM1|6.007525;ATG7|2.120975;PCNA|0.404975;MAPK14|0.483325;HIST1H3A|0.455625;BTF3|0.2449;RHOG|2.449775;TOP2A|0.481675;SNW1|0.473175;STAT3|0.4995;TXNIP|2.0553;RBBP7|0.218475;H3F3A|0.20985;PLK1|0.415475;SOD2|2.175425;ZNF7	RNA biosynthetic process	GO:0032774	8.00E-04	6.06E-03	33	7
5,6	RPL3|3.30745;PABPC1|0.3285;RPL4|2.063325;PABPC4|0.26245;LIG1|0.440425;RPL27|2.15885;STAT3|0.4995	nucleobase-containing compound catabolic process	GO:0034655	8.03E-04	6.35E-03	7	6
5,6,7	ANXA5|3.637725;SQSTM1|6.007525;ATG7|2.120975;CHAF1B|0.2723	positive regulation of macroautophagy	GO:0016239	8.18E-04	6.35E-03	4	7
5,6	HSPA8|0.3721;VIM|2.241075;PABPC1|0.3285;ASPH|2.59965;DNMT1|0.172;GSN|2.4555;BAZ1B|0.460625;SQSTM1|6.007525;MAPK14|0.483325;RHOG|2.449775;TOP2A|0.481675;SNW1|0.473175;STAT3|0.4995;PLP1|3.936	positive regulation of gene expression	GO:0010628	8.38E-04	6.45E-03	14	6
5	HSPD1|2.88995;KRT1|0.4198;PRDX5|3.0143;MAPK14|0.483325;TFRC|0.403125;STAT3|0.4995;VTN|2.01125;PLP	inflammatory response	GO:0006954	8.44E-04	6.59E-03	8	5
5,6	ANXA5|3.637725;SQSTM1|6.007525;ATG7|2.120975;SNW1|0.473175;SLC2A1|5.081875;ALB|0.23405;CHAF1B|0.2723	cellular response to nutrient levels	GO:0031669	8.46E-04	6.62E-03	7	6
6,7	LCP1|2.170475;GSN|2.4555	podosome assembly	GO:0071800	8.61E-04	6.62E-03	2	7
3	HSPD1|2.88995;HIST1H4A|0.23835;LIG1|0.440425;MAPK14|0.483325;TFRC|0.403125;TOP2A|0.481675;PLEK|2.071125;SOS2|11.48525;HEATR9|2.53905	immune system development	GO:0002520	8.64E-04	6.72E-03	9	3
4	CKB|0.486625;ACTB|2.1999;PLP1|3.936	neural nucleus	GO:0048857	8.67E-04	6.72E-03	3	4
5,6	HSPA8|0.3721;ENO1|2.1939;RPL3|3.30745;PABPC1|0.3285;SARS|2.2366;ASPH|2.59965;YARS|2.288275;RPL4|2.063325;NARS|2.162;DNMT1|0.172;HIST1H4A|0.23835;YWHAG|2.359475;SRRT|0.39875;CBX3|0.485775;GTF2E1|0.300475;PABPC4|0.26245;LIG1|0.440425;PRDX5|3.0143;BAZ1B|0.460625;RRM2|0.21675;RPL27|2.15885;SQSTM1|6.007525;ATG7|2.120975;PCNA|0.404975;MAPK14|0.483325;HIST1H3A|0.455625;BTF3|0.2449;RHOG|2.449775;TOP2A|0.481675;SNW1|0.473175;STAT3|0.4995;TXNIP|2.0553;RBBP7|0.218475;H3F3A|0.20985;PLK1|0.415475;SOD2|2.175425;ZNF706|0.3974;CHAF1B|0.2723	RNA metabolic process	GO:0016070	8.87E-04	6.72E-03	38	6
4	ENO1|2.1939;HSPD1|2.88995;ANXA5|3.637725;ATG7|2.120975;MAPK14|0.483325;HIST1H3A|0.455625;GCH1|4.01735;IFITM2|5.68315;CHAF1B|0.2723	response to external biotic stimulus	GO:0043207	9.05E-04	6.86E-03	9	4
3,5	ENO1|2.1939;HSPD1|2.88995;ANXA5|3.637725;ATG7|2.120975;MAPK14|0.483325;HIST1H3A|0.455625;GCH1|4.01735;IFITM2|5.68315;CHAF1B|0.2723	response to other organism	GO:0051707	9.05E-04	6.96E-03	9	5

5	DNMT1|0.172;GSN|2.4555;ATG7|2.120975;VAT1|2.702925;TTK|0.488375;PLK1|0.415475	negative regulation of organelle organization	GO:0010639	9.18E-04	6.96E-03	6	5
7	MCM3|0.35205;MCM2|0.491625;PSME3|0.401575;RRM2|0.21675;PCNA|0.404975	G1/S transition of mitotic cell cycle	GO:0000082	9.19E-04	7.03E-03	5	7
5,6	ACTG1|2.1999;LCP1|2.170475;HSPD1|2.88995;GSN|2.4555;PLEK|2.071125;ACTB|2.1999;SOS2|11.48525	homotypic cell-cell adhesion	GO:0034109	9.38E-04	7.03E-03	7	6
8	HSPA8|0.3721;ENO1|2.1939;ATP6V1B2|2.0575;GMPS|0.496875;ALDOC|2.04725;STAT3|0.4995	purine ribonucleoside monophosphate metabolic process	GO:0009167	9.54E-04	7.16E-03	6	8
4	GSN|2.4555;MAPK14|0.483325;TFRC|0.403125;STAT3|0.4995;SOD2|2.175425	aging	GO:0007568	9.65E-04	7.25E-03	5	4
7	HSPA8|0.3721;ENO1|2.1939;ATP6V1B2|2.0575;GMPS|0.496875;ALDOC|2.04725;STAT3|0.4995	purine nucleoside monophosphate metabolic process	GO:0009126	9.66E-04	7.30E-03	6	7
6,7	HSP90AB1|0.458775;LCP1|2.170475;RPL3|3.30745;ASPH|2.59965;RPL4|2.063325;NSF|2.017425;KPNA2|0.186675;YWHAG|2.359475;RPL27|2.15885;MAPK14|0.483325;STAT3|0.4995;TXNIP|2.0553;IPO8|0.443925	intracellular protein transport	GO:0006886	9.68E-04	7.30E-03	13	7
3	ACTG1|2.1999;HSP90AB1|0.458775;HSPD1|2.88995;KRT1|0.4198;PSME3|0.401575;MAPK14|0.483325;VTN|2.01125;ACTB|2.1999	activation of immune response	GO:0002253	9.71E-04	7.30E-03	8	3
5	SMC2|0.485025;NCAPG|0.4528;TOP2A|0.481675;TTK|0.488375;PLK1|0.415475	nuclear chromosome segregation	GO:0098813	9.81E-04	7.30E-03	5	5
6	MCM3|0.35205;MCM2|0.491625;PSME3|0.401575;RRM2|0.21675;PCNA|0.404975	cell cycle G1/S phase transition	GO:0044843	1.03E-03	7.36E-03	5	6
5	ENO1|2.1939;AKR1B1|2.235325;SLC25A1|2.1121;ALDOC|2.04725;MAPK14|0.483325;SLC2A1|5.081875	monosaccharide metabolic process	GO:0005996	1.07E-03	7.70E-03	6	5
7,8,9	SQSTM1|6.007525;MAPK14|0.483325;STAT3|0.4995;ARHGDIA|3.8215;SOS2|11.48525	neurotrophin TRK receptor signaling	GO:0048011	1.08E-03	7.95E-03	5	9
5	HSPA8|0.3721;HSP90AB1|0.458775;LCP1|2.170475;GSN|2.4555;ERO1L|2.370575;VTN|2.01125	extracellular matrix organization	GO:0030198	1.09E-03	8.01E-03	6	5
4	HSP90AB1|0.458775;HSPD1|2.88995;ASPH|2.59965;YWHAG|2.359475;ANXA5|3.637725;GSN|2.4555;PRDX5|3.0143;PSME3|0.401575;LAMTOR2|7.841025;PCNA|0.404975;MAPK14|0.483325;RHOG|2.449775;TXNIP|2.0553;PLEK|2.071125;VTN|2.01125;ARHGDIA|3.8215;TTK|0.488375;SOS2|11.48525;PLK1|0.415475;GCH1|4.01735	regulation of catalytic activity	GO:0050790	1.09E-03	8.01E-03	20	4
5,6,7,8	HSP90AB1|0.458775;KPNA2|0.186675;MAPK14|0.483325;STAT3|0.4995;TXNIP|2.0553	protein import into nucleus	GO:0006606	1.10E-03	8.01E-03	5	8
5,6,7,8	HSP90AB1|0.458775;KPNA2|0.186675;MAPK14|0.483325;STAT3|0.4995;TXNIP|2.0553	protein targeting to nucleus	GO:0044744	1.10E-03	8.01E-03	5	8
6,7	HSP90AB1|0.458775;KPNA2|0.186675;MAPK14|0.483325;STAT3|0.4995;TXNIP|2.0553	single-organism nuclear import	GO:1902593	1.10E-03	8.01E-03	5	7
5	HSPA8|0.3721;ENO1|2.1939;RPL3|3.30745;SARS|2.2366;ASPH|2.59965;RPL4|2.063325;GMPS|0.496875;DNMT1|0.172;HIST1H4A|0.23835;YWHAG|2.359475;SRRT|0.39875;CBX3|0.485775;GTF2E1|0.300475;LIG1|0.440425;PRDX5|3.0143;BAZ1B|0.460625;RRM2|0.21675;RPL27|2.15885;SQSTM1|6.007525;ATG7|2.120975;PCNA|0.404975;MAPK14|0.483325;HIST1H3A|0.455625;BTF3|0.2449;RHOG|2.449775;TOP2A|0.481675;SNW1|0.473175;STAT3|0.4995;TXNIP|2.0553;RBBP7|0.218475;H3F3A|0.20985;PLK1|0.415475;SOD2|2.175425;ZNF706|0.3974;CHAF1B|0.2723	nucleobase-containing compound biosynthetic process	GO:0034654	1.10E-03	8.01E-03	35	5
4	HSPA8|0.3721;HSP90AB1|0.458775;LCP1|2.170475;GSN|2.4555;ERO1L|2.370575;VTN|2.01125	extracellular structure organization	GO:0043062	1.10E-03	8.01E-03	6	4
4	HSPD1|2.88995;ASPH|2.59965;ATP6V1B2|2.0575;DNMT1|0.172;GSN|2.4555;AKR1B1|2.235325;LAMTOR2|7.841025;MAPK14|0.483325;STAT3|0.4995	response to organonitrogen compound	GO:0010243	1.11E-03	8.01E-03	9	4
5,7,8	HIST1H4A|0.23835;HIST1H3A|0.455625;H3F3A|0.20985	chromatin silencing	GO:0006342	1.11E-03	8.01E-03	3	8
8	HSPA8|0.3721;ASPH|2.59965;GSN|2.4555;PLEK|2.07112	protein depolymerization	GO:0051261	1.11E-03	8.01E-03	4	8
4	HSPA8|0.3721;HSP90AB1|0.458775;HSPD1|2.88995;RBBP7|0.218475	response to heat	GO:0009408	1.11E-03	8.01E-03	4	4
5,6	HSP90AB1|0.458775;HSPD1|2.88995;STAT3|0.4995;SOD2|2.175425	negative regulation of neuron death	GO:1901215	1.13E-03	8.01E-03	4	6
4,5	ANXA5|3.637725;SQSTM1|6.007525;ATG7|2.120975;SNW1|0.473175;SLC2A1|5.081875;ALB|0.23405;CHAF1B|0.	cellular response to extracellular stimulus	GO:0031668	1.14E-03	8.16E-03	7	5
4,5	ATG7|2.120975;SNW1|0.473175;ALB|0.23405	modification of morphology or physiology of other organism involved in symbiotic	GO:0051817	1.15E-03	8.16E-03	3	5
8	HSP90AB1|0.458775;KPNA2|0.186675;MAPK14|0.483325;STAT3|0.4995;TXNIP|2.0553	nuclear import	GO:0051170	1.17E-03	8.26E-03	5	8
6	SMC2|0.485025;NCAPG|0.4528;MCMBP|0.4769;TOP2A|0.481675;NDE1|0.446425;TTK|0.488375;PLK1|0.415475;CETN3|0.4171	nuclear division	GO:0000280	1.18E-03	8.34E-03	8	6
5,6	SMC2|0.485025;NCAPG|0.4528;MCMBP|0.4769;NDE1|0.446425;TTK|0.488375;PLK1|0.415475;CETN3|0.4171	mitotic nuclear division	GO:0007067	1.18E-03	8.40E-03	7	6
5,6	RPL3|3.30745;RPL4|2.063325;NSF|2.017425;YWHAG|2.359475;RPL27|2.15885;RHOG|2.449775	establishment of protein localization to membrane	GO:0090150	1.20E-03	8.40E-03	6	6
6,7	ANXA5|3.637725;SQSTM1|6.007525;ATG7|2.120975;CHAF1B|0.2723	regulation of mitophagy	GO:1903146	1.24E-03	8.52E-03	4	7
7	ENO1|2.1939;MTHFD1L|6.667175;ERO1L|2.370575;ALDOC|2.04725;MAPK14|0.483325;STAT3|0.4995;ALB|0.23405;PLP1|3.936	monocarboxylic acid metabolic process	GO:0032787	1.25E-03	8.76E-03	8	7
4	HSP90AB1|0.458775;LCP1|2.170475;ASPH|2.59965;YWHAG|2.359475;GSN|2.4555;MAPK14|0.483325;RHOG|2.449775;SLC2A1|5.081875;ARHGDIA|3.8215	regulation of protein localization	GO:0032880	1.26E-03	8.82E-03	9	4

4	HSP90AB1|0.458775;HSPD1|2.88995;ASPH|2.59965;GSN|2.4555;BAZ1B|0.460625;PSME3|0.401575;LAMTOR2|7.841025;PCNA|0.404975;MAPK14|0.483325;RHOG|2.449775;PLEK|2.071125;VTN|2.01125;ARHGDIA|3.8215;SOS2|11.48525;PLK1|0.415475;GCH1|4.01735	positive regulation of molecular function	GO:0044093	1.27E-03	8.90E-03	16	4
7	HSPA8|0.3721;ENO1|2.1939;ATP6V1B2|2.0575;GMPS|0.496875;ALDOC|2.04725;STAT3|0.4995	ribonucleoside monophosphate metabolic process	GO:0009161	1.27E-03	8.92E-03	6	7
6	HSP90AB1|0.458775;RPL3|3.30745;ASPH|2.59965;RPL4|2.063325;NSF|2.017425;KPNA2|0.186675;ERO1L|2.370575;RPL27|2.15885;MAPK14|0.483325;STAT3|0.4995;TXNI	cytoplasmic transport	GO:0016482	1.29E-03	8.92E-03	11	6
6	NDRG1|10.0073;TTK|0.488375;PLK1|0.415475	spindle checkpoint	GO:0031577	1.29E-03	8.98E-03	3	6
6,7	SQSTM1|6.007525;MAPK14|0.483325;STAT3|0.4995;ARHGDIA|3.8215;SOS2|11.48525	neurotrophin signaling pathway	GO:0038179	1.30E-03	9.00E-03	5	7
7	HIST1H4A|0.23835;CBX3|0.485775;BAZ1B|0.460625;RBBP7|0.218475;ACTB|2.1999	chromatin remodeling	GO:0006338	1.32E-03	9.02E-03	5	7
5,6	HSPA8|0.3721;ACTG1|2.1999;HSP90AB1|0.458775;ATG7|2.120975;RHOG|2.449775;ARHGDIA|3.8215;ACTB|2.1999;MYH10|5.579275;SOS2|11.48525	cell morphogenesis involved in differentiation	GO:0000904	1.32E-03	9.12E-03	9	6
6,7	GSN|2.4555;VTN|2.01125;PLP1|3.936	oligodendrocyte differentiation	GO:0048709	1.34E-03	9.12E-03	3	7
5,8	VIM|2.241075;GSN|2.4555;TOP2A|0.481675	cellular component disassembly involved in execution phase of apoptosis	GO:0006921	1.34E-03	9.22E-03	3	8
6,7	VIM|2.241075;KRT9|0.268525	intermediate filament organization	GO:0045109	1.35E-03	9.22E-03	2	7
4,5,7,8	GSN|2.4555;IFITM2|5.68315	negative regulation of viral entry into host cell	GO:0046597	1.35E-03	9.25E-03	2	8
6,7	LIG1|0.440425;PCNA|0.404975	nucleotide-excision repair, DNA gap filling	GO:0006297	1.35E-03	9.25E-03	2	7
4	HSP90AB1|0.458775;PRDX5|3.0143;GCH1|4.01735	reactive nitrogen species metabolic process	GO:2001057	1.49E-03	9.25E-03	3	4
5,6,7,8	SMC2|0.485025;TOP2A|0.481675;PLK1|0.415475	meiotic chromosome segregation	GO:0045132	1.49E-03	1.01E-02	3	8
6	ANXA5|3.637725;SQSTM1|6.007525;ATG7|2.120975;CHAF1B|0.2723	positive regulation of autophagy	GO:0010508	1.50E-03	1.01E-02	4	6
4	HSPA8|0.3721;ENO1|2.1939;ATP6V1B2|2.0575;GMPS|0.496875;AKR1B1|2.235325;ALDOC|2.04725;STAT3|0.499	glycosyl compound metabolic process	GO:1901657	1.51E-03	1.01E-02	7	4
5,6	HSPA8|0.3721;ENO1|2.1939;VIM|2.241075;PABPC1|0.3285;ASPH|2.59965;DNMT1|0.172;HIST1H4A|0.23835;SRRT|0.39875;GSN|2.4555;CBX3|0.485775;GTF2E1|0.300475;PRDX5|3.0143;BAZ1B|0.460625;RRM2|0.21675;EIF4H|0.275675;SQSTM1|6.007525;PCNA|0.404975;MAPK14|0.483325;HIST1H3A|0.455625;BTF3|0.2449;RHOG|2.449775;TOP2A|0.481675;SNW1|0.473175;STAT3|0.4995;TXNIP|2.0553;VTN|2.01125;RBBP7|0.218475;H3F3A|0.20985;PLK1|0.415475;SOD2|2.175425;ZNF706|0.3974;CHAF1B|0.27	regulation of gene expression	GO:0010468	1.54E-03	1.02E-02	33	6
4,5	HSPA8|0.3721;HSP90AB1|0.458775;ANXA5|3.637725;SQSTM1|6.007525;ATG7|2.120975;PCNA|0.404975;SOD2|2.175425;CHAF1B|0.2723	regulation of cellular response to stress	GO:0080135	1.55E-03	1.04E-02	8	5
7,8	HSPD1|2.88995;ASPH|2.59965;GSN|2.4555;PSME3|0.401575	positive regulation of endopeptidase activity	GO:0010950	1.59E-03	1.04E-02	4	8
5	ATP6V1B2|2.0575;DNMT1|0.172;AKR1B1|2.235325;PRDX5|3.0143;LAMTOR2|7.841025;MAPK14|0.483325;SNW1|0.473175;STAT3|0.4995;SOD2|2.175425	cellular response to oxygen-containing compound	GO:1901701	1.62E-03	1.07E-02	9	5
4,5	PRDX5|3.0143;HBA1|2.13735	hydrogen peroxide catabolic process	GO:0042744	1.64E-03	1.08E-02	2	5
5	HSPA8|0.3721;HSP90AB1|0.458775;LCP1|2.170475;RPL3|3.30745;ASPH|2.59965;RPL4|2.063325;NSF|2.017425;KPNA2|0.186675;YWHAG|2.359475;ERO1L|2.370575;RPL27|2.15885;SQSTM1|6.007525;MAPK14|0.483325;STAT3|0.4995;TXNIP|2.0553;IPO8|0.443925;NDE1|0.446425	intracellular transport	GO:0046907	1.65E-03	1.09E-02	17	5
6	HSPA8|0.3721;ENO1|2.1939;ATP6V1B2|2.0575;GMPS|0.496875;ALDOC|2.04725;STAT3|0.4995	nucleoside monophosphate metabolic	GO:0009123	1.65E-03	1.09E-02	6	6
4	CKB|0.486625;ACTB|2.1999;PLP1|3.936	midbrain development	GO:0030901	1.65E-03	1.09E-02	3	4
6,7	SMC2|0.485025;NCAPG|0.4528;TTK|0.488375;PLK1|0.415475	mitotic sister chromatid segregation	GO:0000070	1.65E-03	1.09E-02	4	7
4	ASPH|2.59965;CKB|0.486625;ATG7|2.120975;NDE1|0.446425;ACTB|2.1999;H3F3A|0.20985;MYH10|5.579275;PLP	head development	GO:0060322	1.68E-03	1.09E-02	8	4
7	ENO1|2.1939;DNMT1|0.172;YWHAG|2.359475;GTF2E1|0.300475;PRDX5|3.0143;RRM2|0.21675;SQSTM1|6.007525;PCNA|0.404975;MAPK14|0.483325;BTF3|0.2449;TOP2A|0.481675;SNW1|0.473175;STAT3|0.4995;TXNIP|2.0553;RBBP7|0.218475;PLK1|0.415475;SOD2|2.175425	transcription from RNA polymerase II promoter	GO:0006366	1.69E-03	1.11E-02	17	7
6	DNMT1|0.172;ATG7|2.120975;SNW1|0.473175;TTK|0.488375;PLK1|0.415475	regulation of chromosome organization	GO:0033044	1.71E-03	1.11E-02	5	6
8	HIST1H4A|0.23835;RBBP7|0.218475;ACTB|2.1999	ATP-dependent chromatin remodeling	GO:0043044	1.71E-03	1.12E-02	3	8
4,5	ACTG1|2.1999;HSP90AB1|0.458775;HSPD1|2.88995;KRT1|0.4198;PSME3|0.401575;MAPK14|0.483325;TXNIP|2.0553;VTN|2.01125;ACTB|2.1999;GCH1|4.01735;IFITM2|5.6	innate immune response	GO:0045087	1.72E-03	1.12E-02	11	5
5,6,7	GSN|2.4555;TOP2A|0.481675;SNW1|0.473175;IFITM2|5.68315	regulation of viral life cycle	GO:1903900	1.72E-03	1.12E-02	4	7
3	HSP90AB1|0.458775;ASPH|2.59965;YWHAG|2.359475;MAPK14|0.483325;RHOG|2.449775	positive regulation of cellular protein	GO:1903829	1.73E-03	1.12E-02	5	3

8,9,10,11	HSP90AB1|0.458775;TTK|0.488375;PLK1|0.415475	negative regulation of proteasomal ubiquitin-dependent protein catabolic process	GO:0032435	1.77E-03	1.13E-02	3	11
4	HSP90AB1|0.458775;STAT3|0.4995;GCH1|4.01735	reactive oxygen species biosynthetic process	GO:1903409	1.77E-03	1.14E-02	3	4
5,6	RPL3|3.30745;RPL4|2.063325;NSF|2.017425;YWHAG|2.359475;GSN|2.4555;RPL27|2.15885;RHOG|2.449775	protein localization to membrane	GO:0072657	1.79E-03	1.14E-02	7	6
4	HSPA8|0.3721;HSP90AB1|0.458775;LCP1|2.170475;RPL3|3.30745;ASPH|2.59965;RPL4|2.063325;NSF|2.017425;KPNA2|0.186675;YWHAG|2.359475;ERO1L|2.370575;RPL27|2.15885;SQSTM1|6.007525;MAPK14|0.483325;STAT3|0.4995;SLC2A1|5.081875;TXNIP|2.0553;IPO8|0.443925;PLEK|2.071125;NDE1|0.446425;MYH10|5.579275;ALB|0.23	establishment of localization in cell	GO:0051649	1.87E-03	1.16E-02	21	4
4	HSPA8|0.3721;HSP90AB1|0.458775;ENO1|2.1939;PABPC1|0.3285;ASPH|2.59965;DNMT1|0.172;HIST1H4A|0.23835;KPNA2|0.186675;SRRT|0.39875;CBX3|0.485775;GTF2E1|0.300475;PRDX5|3.0143;BAZ1B|0.460625;PSME3|0.401575;RRM2|0.21675;EIF4H|0.275675;SQSTM1|6.007525;PCNA|0.404975;MAPK14|0.483325;HIST1H3A|0.455625;BTF3|0.2449;RHOG|2.449775;TOP2A|0.481675;SNW1|0.473175;STAT3|0.4995;TXNIP|2.0553;S100A11|2.531875;RBBP7|0.218475;H3F3A|0.20985;PLK1|0.415475;SOD2|2.175425;ZNF706|0.3974;CHAF1B|0.2723	regulation of nitrogen compound metabolic process	GO:0051171	1.88E-03	1.21E-02	33	4
5	MTHFD1L|6.667175;AKR1B1|2.235325;ISYNA1|0.49065;PLEK|2.071125;ASL|2.252975;GCH1|4.01735;PLP1|3.936	small molecule biosynthetic process	GO:0044283	1.89E-03	1.21E-02	7	5
6,7	LIG1|0.440425;TOP2A|0.481675	DNA ligation	GO:0006266	1.95E-03	1.21E-02	2	7
6,7,8,9	HSPA8|0.3721;SNW1|0.473175	positive regulation of RNA splicing	GO:0033120	1.95E-03	1.24E-02	2	9
7	AKR1B1|2.235325;ALDOC|2.04725	fructose metabolic process	GO:0006000	1.95E-03	1.24E-02	2	7
5	SMC2|0.485025;NCAPG|0.4528;MCMBP|0.4769;TOP2A|0.481675;NDE1|0.446425;TTK|0.488375;PLK1|0.415475;CETN3|0.4171	organelle fission	GO:0048285	1.95E-03	1.24E-02	8	5
6,7	YWHAG|2.359475;TUBG1|0.4102;NDE1|0.446425;PLK1|0.415475	G2/M transition of mitotic cell cycle	GO:0000086	2.01E-03	1.24E-02	4	7
7,8,9,10	HSP90AB1|0.458775;TTK|0.488375;PLK1|0.415475	negative regulation of proteasomal protein catabolic process	GO:1901799	2.01E-03	1.27E-02	3	10
7	HSPA8|0.3721;HSPD1|2.88995;ERO1L|2.370575	chaperone-mediated protein folding	GO:0061077	2.01E-03	1.27E-02	3	7
3	HSPA8|0.3721;ACTG1|2.1999;HSP90AB1|0.458775;MAPK14|0.483325;RHOG|2.449775;ACTB|2.1999;MYH10|5.579275;SOS2|11.48525	taxis	GO:0042330	2.01E-03	1.27E-02	8	3
4	HSPA8|0.3721;ACTG1|2.1999;HSP90AB1|0.458775;MAPK14|0.483325;RHOG|2.449775;ACTB|2.1999;MYH10|5.579275;SOS2|11.48525	chemotaxis	GO:0006935	2.01E-03	1.27E-02	8	4
6	YWHAG|2.359475;TUBG1|0.4102;NDE1|0.446425;PLK1|0.415475	cell cycle G2/M phase transition	GO:0044839	2.08E-03	1.27E-02	4	6
3	HSPD1|2.88995;ASPH|2.59965;ATP6V1B2|2.0575;DNMT1|0.172;GSN|2.4555;AKR1B1|2.235325;LAMTOR2|7.841025;MAPK14|0.483325;SNW1|0.473175;STAT3|0.4995;TXNIP|2.0553;RBBP7|0.218475;H3F3A|0.20985	response to endogenous stimulus	GO:0009719	2.10E-03	1.31E-02	13	3
5,6	LIG1|0.440425;PCNA|0.404975	telomere maintenance via semi-conservative replication	GO:0032201	2.12E-03	1.31E-02	2	6
7,8,10,11	DNMT1|0.172;SNW1|0.473175	regulation of histone H3-K4 methylation	GO:0051569	2.12E-03	1.31E-02	2	11
4	HSPD1|2.88995;ASPH|2.59965;GSN|2.4555;PLK1|0.415475	regulation of protein stability	GO:0031647	2.12E-03	1.31E-02	4	4
5,7	RPL3|3.30745;RPL4|2.063325;RPL27|2.15885;SNW1|0.47	viral transcription	GO:0019083	2.12E-03	1.31E-02	4	7
6	RPL3|3.30745;PABPC1|0.3285;SARS|2.2366;YARS|2.288275;RPL4|2.063325;NARS|2.162;PABPC4|0.26245;EIF4H|0.275675;RPL27|2.15885;ZNF706|0.3974	translation	GO:0006412	2.12E-03	1.31E-02	10	6
5,6	MTHFD1L|6.667175;CNDP2|2.0938;GCH1|4.01735	cellular modified amino acid biosynthetic process	GO:0042398	2.14E-03	1.31E-02	3	6
3,4	ATG7|2.120975;SNW1|0.473175;ALB|0.23405	modification of morphology or physiology of other organism	GO:0035821	2.14E-03	1.32E-02	3	4
4	LCP1|2.170475;VIM|2.241075;KRT9|0.268525;MTHFD1L|6.667175;GSN|2.4555;ALDOC|2.04725;ATG7|2.120975;PCNA|0.404975;MAPK14|0.483325;SNW1|0.473175;TXNIP|2.0553;VTN|2.01125;MYH10|5.579275	tissue development	GO:0009888	2.15E-03	1.32E-02	13	4
4,5,6	GSN|2.4555;TOP2A|0.481675;SNW1|0.473175;IFITM2|5.68315	regulation of viral process	GO:0050792	2.16E-03	1.32E-02	4	6
3	ENO1|2.1939;SQSTM1|6.007525;MAPK14|0.483325;TFRC|0.403125;STAT3|0.4995;RBBP7|0.218475;H3F3A|0.209	regulation of growth	GO:0040008	2.17E-03	1.32E-02	7	3
5,6	HSPA8|0.3721;ACTG1|2.1999;HSP90AB1|0.458775;VDAC3|2.015475;GSN|2.4555;ATG7|2.120975;MAPK14|0.483325;RHOG|2.449775;ARHGDIA|3.8215;ACTB|2.1999;MYH10|5.579275;SOS2|11.48525	cell morphogenesis	GO:0000902	2.18E-03	1.33E-02	12	6
3	NSF|2.017425;YWHAG|2.359475;GSN|2.4555;AKR1B1|2.235325;PSME3|0.401575;LAMTOR2|7.841025;MAPK14|0.483325;SNW1|0.473175;STAT3|0.4995;VTN|2.01125;TTK|0.488375;SOS2|11.48525	positive regulation of signaling	GO:0023056	2.18E-03	1.33E-02	12	3
5	HSP90AB1|0.458775;RPL3|3.30745;PABPC1|0.3285;RPL4|2.063325;NSF|2.017425;PABPC4|0.26245;LIG1|0.440425;PSME3|0.401575;RPL27|2.15885;SQSTM1|6.007525;ATG7|2.120975;TTK|0.488375;PLK1|0.415475	macromolecule catabolic process	GO:0009057	2.22E-03	1.33E-02	13	5

6,7	HSPD1|2.88995;ASPH|2.59965;GSN|2.4555;PSME3|0.401575	positive regulation of peptidase activity	GO:0010952	2.24E-03	1.35E-02	4	7
6,8,9	RRM2|0.21675;PCNA|0.404975	regulation of transcription involved in G1/S transition of mitotic cell	GO:0000083	2.29E-03	1.36E-02	2	9
6,8,9	LCP1|2.170475;GSN|2.4555	epiboly involved in wound healing	GO:0090505	2.29E-03	1.38E-02	2	9
5	LCP1|2.170475;GSN|2.4555	wound healing, spreading of cells	GO:0044319	2.29E-03	1.38E-02	2	5
5,6,7	ANXA5|3.637725;SQSTM1|6.007525;ATG7|2.120975;CHAF1B|0.2723	regulation of macroautophagy	GO:0016241	2.45E-03	1.38E-02	4	7
5	ENO1|2.1939;ALDOC|2.04725	NADH regeneration	GO:0006735	2.47E-03	1.46E-02	2	5
6	ENO1|2.1939;ALDOC|2.04725	glycolytic process through fructose-6-phosphate	GO:0061615	2.47E-03	1.46E-02	2	6
5,6,7,8	LIG1|0.440425;PCNA|0.404975	telomere maintenance via recombination	GO:0000722	2.47E-03	1.46E-02	2	8
7,8	LCP1|2.170475;GSN|2.4555	epiboly	GO:0090504	2.47E-03	1.46E-02	2	8
9	ENO1|2.1939;ALDOC|2.04725	glucose catabolic process to pyruvate	GO:0061718	2.47E-03	1.46E-02	2	9
6,8	ENO1|2.1939;ALDOC|2.04725	canonical glycolysis	GO:0061621	2.47E-03	1.46E-02	2	8
7	ENO1|2.1939;ALDOC|2.04725	glycolytic process through glucose-6-phosphate	GO:0061620	2.47E-03	1.46E-02	2	7
7,8	HSP90AB1|0.458775;TTK|0.488375;PLK1|0.415475	negative regulation of proteolysis involved in cellular protein catabolic process	GO:1903051	2.48E-03	1.46E-02	3	8
4,5	AKR1B1|2.235325;ISYNA1|0.49065;SLC2A1|5.081875	cellular carbohydrate biosynthetic process	GO:0034637	2.48E-03	1.46E-02	3	5
4,6	RPL3|3.30745;RPL4|2.063325;RPL27|2.15885;SNW1|0.47	viral gene expression	GO:0019080	2.54E-03	1.46E-02	4	6
5,6,7	HSP90AB1|0.458775;KPNA2|0.186675;MAPK14|0.483325;STAT3|0.4995;TXNIP|2.0553	protein import	GO:0017038	2.54E-03	1.49E-02	5	7
7	HSP90AB1|0.458775;KPNA2|0.186675;MAPK14|0.483325;STAT3|0.4995;TXNIP|2.0553	protein localization to nucleus	GO:0034504	2.57E-03	1.49E-02	5	7
6	ENO1|2.1939;AKR1B1|2.235325;SLC25A1|2.1121;ALDOC|2.04725;MAPK14|0.483325	hexose metabolic process	GO:0019318	2.64E-03	1.49E-02	5	6
4,5	HSP90AB1|0.458775;HSPD1|2.88995;ASPH|2.59965;GSN|2.4555;PSME3|0.401575;LAMTOR2|7.841025;PCNA|0.404975;MAPK14|0.483325;RHOG|2.449775;PLEK|2.071125;ARHGDIA|3.8215;SOS2|11.48525;PLK1|0.415475;GCH1|	positive regulation of catalytic activity	GO:0043085	2.66E-03	1.49E-02	14	5
6	PSME3|0.401575;MAPK14|0.483325;TOP2A|0.481675;PLK1|0.415475	DNA integrity checkpoint	GO:0031570	2.67E-03	1.49E-02	4	6
5	HSPA8|0.3721;HSP90AB1|0.458775;ENO1|2.1939;PABPC1|0.3285;ASPH|2.59965;DNMT1|0.172;HIST1H4A|0.23835;SRRT|0.39875;CBX3|0.485775;GTF2E1|0.300475;PRDX5|3.0143;BAZ1B|0.460625;RRM2|0.21675;EIF4H|0.275675;SQSTM1|6.007525;PCNA|0.404975;MAPK14|0.483325;HIST1H3A|0.455625;BTF3|0.2449;RHOG|2.449775;TOP2A|0.481675;SNW1|0.473175;STAT3|0.4995;TXNIP|2.0553;PLEK|2.071125;S100A11|2.531875;RBBP7|0.218475;H3F3A|0.20985;PLK1|0.415475;SOD2|2.175425;ZNF706|0.397	regulation of cellular biosynthetic process	GO:0031326	2.70E-03	1.49E-02	32	5
5,6	HSPD1|2.88995	detection of misfolded protein	GO:0002236	2.73E-03	1.49E-02	1	6
6,7	GCH1|4.01735	7,8-dihydroneopterin 3'-triphosphate biosynthetic process	GO:0035998	2.73E-03	1.49E-02	1	7
6	GSN|2.4555	plasma membrane raft distribution	GO:0044855	2.73E-03	1.49E-02	1	6
6	PLEK|2.071125	regulation of cell diameter	GO:0060305	2.73E-03	1.49E-02	1	6
6,7,8	TOP2A|0.481675	positive regulation of single stranded viral RNA replication via double stranded DNA	GO:0045870	2.73E-03	1.49E-02	1	8
6,7	AKR1B1|2.235325	hexitol biosynthetic	GO:0019406	2.73E-03	1.49E-02	1	7
6,7	ATG7|2.120975	modulation by virus of host autophagy	GO:0039519	2.73E-03	1.49E-02	1	7
6,7,8	PRDX5|3.0143	regulation of apoptosis involved in tissue homeostasis	GO:0060785	2.73E-03	1.49E-02	1	8
7,8	AKR1B1|2.235325	sorbitol biosynthetic	GO:0006061	2.73E-03	1.49E-02	1	8
7	GSN|2.4555	plasma membrane raft polarization	GO:0044858	2.73E-03	1.49E-02	1	7
5	TXNIP|2.0553	cellular response to tumor cell	GO:0071228	2.73E-03	1.49E-02	1	5
7,9,12	SOD2|2.175425	vasodilation by acetylcholine involved in regulation of systemic arterial blood pressure	GO:0003069	2.73E-03	1.49E-02	1	12
6,7	STAT3|0.4995	positive regulation of satellite cell proliferation	GO:1902724	2.73E-03	1.49E-02	1	7
7	ATG7|2.120975	suppression by virus of host autophagy	GO:0039521	2.73E-03	1.49E-02	1	7
5,8	GSN|2.4555	regulation of plasma membrane raft	GO:1903906	2.73E-03	1.49E-02	1	8
6,7	HSPA8|0.3721	negative regulation of fibril organization	GO:1902904	2.73E-03	1.49E-02	1	7
6	GSN|2.4555	plasma membrane raft localization	GO:0044856	2.73E-03	1.49E-02	1	6

7	PLEK|2.071125	regulation of inositol-polyphosphate 5-phosphatase activity	GO:0010924	2.73E-03	1.49E-02	1	7
6	SOD2|2.175425	age-dependent response to reactive oxygen species	GO:0001315	2.73E-03	1.49E-02	1	6
8,11	SOD2|2.175425	regulation of systemic arterial blood pressure by acetylcholine	GO:0003068	2.73E-03	1.49E-02	1	11
7,8	STAT3|0.4995	positive regulation of growth factor dependent skeletal muscle satellite cell proliferation	GO:1902728	2.73E-03	1.49E-02	1	8
5,6,7	GSN|2.4555	regulation of receptor clustering	GO:1903909	2.73E-03	1.49E-02	1	7
6,7,8	PCNA|0.404975	positive regulation of deoxyribonuclease activity	GO:0032077	2.73E-03	1.49E-02	1	8
6	AKR1B1|2.235325	cellular response to methylglyoxal	GO:0097238	2.73E-03	1.49E-02	1	6
7,8	PLEK|2.071125	positive regulation of inositol-polyphosphate 5-phosphatase activity	GO:0010925	2.73E-03	1.49E-02	1	8
6,7	PLEK|2.071125	negative regulation of inositol phosphate biosynthetic process	GO:0010920	2.73E-03	1.49E-02	1	7
7,10	SOD2|2.175425	regulation of systemic arterial blood pressure by neurotransmitter	GO:0003070	2.73E-03	1.49E-02	1	10
6,7	PLEK|2.071125	phospholipase C-inhibiting G-protein coupled receptor signaling	GO:0030845	2.73E-03	1.49E-02	1	7
4,5,7	ANXA5|3.637725;ATG7|2.120975;HIST1H3A|0.455625;IFITM2|5.68315;CHAF1B|0.2723	defense response to virus	GO:0051607	2.73E-03	1.49E-02	5	7
4	ACTG1|2.1999;HSP90AB1|0.458775;HSPD1|2.88995;KRT1|0.4198;PSME3|0.401575;MAPK14|0.483325;VTN|2.01125;ACTB|2.1999	positive regulation of immune response	GO:0050778	2.76E-03	1.50E-02	8	4
3	HSP90AB1|0.458775;HSPD1|2.88995;ASPH|2.59965;YWHAG|2.359475;ANXA5|3.637725;GSN|2.4555;PRDX5|3.0143;BAZ1B|0.460625;PSME3|0.401575;LAMTOR2|7.841025;PCNA|0.404975;MAPK14|0.483325;RHOG|2.449775;TXNIP|2.0553;PLEK|2.071125;VTN|2.01125;ARHGDIA|3.8215;TTK|0.488375;SOS2|11.48525;PLK1|0.415475;GCH1|	regulation of molecular function	GO:0065009	2.83E-03	1.51E-02	21	3
4	ATG7|2.120975;MYH10|5.579275	cerebellar Purkinje cell layer development	GO:0021680	2.85E-03	1.54E-02	2	4
4,7,8	GSN|2.4555;IFITM2|5.68315	regulation of viral entry into host cell	GO:0046596	2.85E-03	1.54E-02	2	8
7	HSPA8|0.3721;HSPD1|2.88995	protein refolding	GO:0042026	2.85E-03	1.54E-02	2	7
6,7,8,10,11	DNMT1|0.172;SNW1|0.473175	positive regulation of histone methylation	GO:0031062	2.85E-03	1.54E-02	2	11
6,7	HSP90AB1|0.458775;TTK|0.488375;PLK1|0.415475	negative regulation of cellular protein catabolic process	GO:1903363	2.86E-03	1.54E-02	3	7
5,6,7	ANXA5|3.637725;SQSTM1|6.007525;ATG7|2.120975;SLC2A1|5.081875;ALB|0.23405;CHAF1B|0.2723	cellular response to starvation	GO:0009267	2.87E-03	1.54E-02	6	7
4	HSPA8|0.3721;HSP90AB1|0.458775;ENO1|2.1939;PABPC1|0.3285;ASPH|2.59965;DNMT1|0.172;HIST1H4A|0.23835;SRRT|0.39875;CBX3|0.485775;GTF2E1|0.300475;PRDX5|3.0143;BAZ1B|0.460625;RRM2|0.21675;EIF4H|0.275675;SQSTM1|6.007525;PCNA|0.404975;MAPK14|0.483325;HIST1H3A|0.455625;BTF3|0.2449;RHOG|2.449775;TOP2A|0.481675;SNW1|0.473175;STAT3|0.4995;TXNIP|2.0553;PLEK|2.071125;S100A11|2.531875;RBBP7|0.218475;H3F3A|0.20985;PLK1|0.415475;SOD2|2.175425;ZNF706|0.397	regulation of biosynthetic process	GO:0009889	2.98E-03	1.55E-02	32	4
4	HSPD1|2.88995;ANXA5|3.637725;HIST1H3A|0.455625;CHAF1B|0.2723	regulation of response to biotic stimulus	GO:0002831	3.00E-03	1.60E-02	4	4
4	ENO1|2.1939;ASPH|2.59965;MTHFD1L|6.667175;ALDH1L2|2.148;AKR1B1|2.235325;ERO1L|2.370575;PRDX5|3.0143;ALDOC|2.04725;RRM2|0.21675;MAPK14|0.483325;SLC2A1|5.081875;VAT1|2.702925;HBA1|2.13735;SOD2|2.	oxidation-reduction process	GO:0055114	3.07E-03	1.61E-02	14	4
2	CCT7|2.06135;KRT9|0.268525;SMC2|0.485025;AKR1B1|2.235325;EIF4H|0.275675;TOP2A|0.481675;STAT3|0.4995;TUBG1|0.4102;PLK1|0.415475	reproduction	GO:0000003	3.09E-03	1.64E-02	9	2
8,9	HSPA8|0.3721;ENO1|2.1939;ATP6V1B2|2.0575;ALDOC|2.04725;STAT3|0.4995	ATP metabolic process	GO:0046034	3.16E-03	1.65E-02	5	9
4,5,6,7	YWHAG|2.359475;GSN|2.4555;AKR1B1|2.235325;PSME3|0.401575;LAMTOR2|7.841025;MAPK14|0.483325;SNW1|0.473175;STAT3|0.4995;VTN|2.01125;TTK|0.488375;SO	positive regulation of signal transduction	GO:0009967	3.16E-03	1.68E-02	11	7
5	HSPA8|0.3721;HSP90AB1|0.458775;RBBP7|0.218475	cellular response to heat	GO:0034605	3.18E-03	1.68E-02	3	5
4,5	SMC2|0.485025;TOP2A|0.481675;TUBG1|0.4102;PLK1|0.415475	meiotic cell cycle process	GO:1903046	3.21E-03	1.69E-02	4	5
7	HSPA8|0.3721;ENO1|2.1939;ATP6V1B2|2.0575;GMPS|0.496875;ALDOC|2.04725;STAT3|0.4995	purine ribonucleoside metabolic process	GO:0046128	3.22E-03	1.70E-02	6	7
3	VIM|2.241075;KRT1|0.4198;HIST1H4A|0.23835;VDAC3|2.015475;YWHAG|2.359475;SRRT|0.39875;GSN|2.4555;MAPK14|0.483325;SNW1|0.473175;STAT3|0.4995;VAT1|2.702925;ARHGDIA|3.8215;MYH10|5.579275;SOS2|11.48525;ZNF706|0.3974	regulation of developmental process	GO:0050793	3.25E-03	1.70E-02	15	3

6,7	MTHFD1L|6.667175;GCH1|4.01735	folic acid-containing compound biosynthetic process	GO:0009396	3.25E-03	1.70E-02	2	7
6	HIST1H4A|0.23835;RBBP7|0.218475	CENP-A containing chromatin organization	GO:0061641	3.25E-03	1.70E-02	2	6
4,6,7,8	ATG7|2.120975;TOP2A|0.481675	viral RNA genome replication	GO:0039694	3.25E-03	1.70E-02	2	8
7,8,9	HIST1H4A|0.23835;RBBP7|0.218475	CENP-A containing nucleosome assembly	GO:0034080	3.25E-03	1.70E-02	2	9
6,7	ATG7|2.120975;TOP2A|0.481675	RNA replication	GO:0039703	3.25E-03	1.70E-02	2	7
5,7	HIST1H4A|0.23835;SOS2|11.48525	regulation of hematopoietic progenitor cell differentiation	GO:1901532	3.25E-03	1.70E-02	2	7
8	ENO1|2.1939;SLC25A1|2.1121;ALDOC|2.04725	gluconeogenesis	GO:0006094	3.26E-03	1.70E-02	3	8
4	TXNIP|2.0553;TTK|0.488375;PLK1|0.415475	negative regulation of cell division	GO:0051782	3.26E-03	1.70E-02	3	4
6	HSPA8|0.3721;ENO1|2.1939;ATP6V1B2|2.0575;GMPS|0.496875;ALDOC|2.04725;STAT3|0.4995	purine nucleoside metabolic process	GO:0042278	3.31E-03	1.70E-02	6	6
6	SQSTM1|6.007525;MAPK14|0.483325;SNW1|0.473175;STAT3|0.4995;VTN|2.01125;ARHGDIA|3.8215;SOS2|11.48525;PTP4A3|0.3433	cellular response to growth factor stimulus	GO:0071363	3.35E-03	1.72E-02	8	6
5,6,7	ATG7|2.120975;TOP2A|0.481675;IFITM2|5.68315	viral genome replication	GO:0019079	3.35E-03	1.73E-02	3	7
6,7,8,10,11,12	PSME3|0.401575;TTK|0.488375;PLK1|0.415475	negative regulation of ubiquitin-protein ligase activity involved in mitotic cell cycle	GO:0051436	3.35E-03	1.73E-02	3	12
3	HSP90AB1|0.458775;LCP1|2.170475;ASPH|2.59965;NSF|2.017425;VDAC3|2.015475;YWHAG|2.359475;ANXA5|3.637725;GSN|2.4555;ERO1L|2.370575;ATG7|2.120975;MAPK14|0.483325;RHOG|2.449775;STAT3|0.4995;SLC2A1|5.081875;VTN|2.01125;ARHGDIA|3.8215	regulation of localization	GO:0032879	3.44E-03	1.73E-02	16	3
6,9,10	PABPC1|0.3285;MAPK14|0.483325	mRNA stabilization	GO:0048255	3.46E-03	1.77E-02	2	10
6,7	SMC2|0.485025;NCAPG|0.4528	mitotic chromosome condensation	GO:0007076	3.46E-03	1.77E-02	2	7
5,8,9	PABPC1|0.3285;MAPK14|0.483325	RNA stabilization	GO:0043489	3.46E-03	1.77E-02	2	9
5	DNMT1|0.172;GSN|2.4555;LAMTOR2|7.841025	response to amino acid	GO:0043200	3.53E-03	1.77E-02	3	5
8,9	RPL3|3.30745;PABPC1|0.3285;RPL4|2.063325;RPL27|2.15885	nuclear-transcribed mRNA catabolic process	GO:0000956	3.53E-03	1.80E-02	4	9
5	LIG1|0.440425;BAZ1B|0.460625;PSME3|0.401575;PCNA|0.404975;MAPK14|0.483325;TOP2A|0.481675;SNW1|0.473175;NDRG1|10.0073;PLK1|0.415475;CHAF1B|0.2723	cellular response to DNA damage stimulus	GO:0006974	3.54E-03	1.80E-02	10	5
4,5	HSPA8|0.3721;ACTG1|2.1999;HSP90AB1|0.458775;VDAC3|2.015475;GSN|2.4555;ATG7|2.120975;MAPK14|0.483325;RHOG|2.449775;ARHGDIA|3.8215;ACTB|2.1999;MYH10|5.579275;SOS2|11.48525	cellular component morphogenesis	GO:0032989	3.55E-03	1.80E-02	12	5
5	GSN|2.4555;PLK1|0.415475	protein destabilization	GO:0031648	3.68E-03	1.81E-02	2	5
8	ENO1|2.1939;ALDOC|2.04725	glucose catabolic process	GO:0006007	3.68E-03	1.86E-02	2	8
5,6,7	HSPD1|2.88995;MAPK14|0.483325	positive regulation of interleukin-12 production	GO:0032735	3.68E-03	1.86E-02	2	7
6,7,8,10	ANXA5|3.637725;HIST1H3A|0.455625;CHAF1B|0.2723	positive regulation of defense response to virus by host	GO:0002230	3.71E-03	1.86E-02	3	10
6,7,8,9	HSPA8|0.3721;PABPC1|0.3285;SNW1|0.473175	regulation of mRNA processing	GO:0050684	3.71E-03	1.87E-02	3	9
6	ENO1|2.1939;ALDOC|2.04725;RRM2|0.21675;STAT3|0.4995	nucleoside diphosphate metabolic process	GO:0009132	3.75E-03	1.87E-02	4	6
5	SQSTM1|6.007525;MAPK14|0.483325;SNW1|0.473175;STAT3|0.4995;VTN|2.01125;ARHGDIA|3.8215;SOS2|11.48525;PTP4A3|0.3433	response to growth factor	GO:0070848	3.82E-03	1.89E-02	8	5
5	ACTG1|2.1999;LCP1|2.170475;VIM|2.241075;KRT9|0.268525;GSN|2.4555;RHOG|2.449775;TUBG1|0.4102;PLEK|2.071125;NDE1|0.446425;MYH10|5.579275;TTK|0.488375;PLK1|0.415475;CETN3|0.4171	cytoskeleton organization	GO:0007010	3.87E-03	1.92E-02	13	5
6,7	DNMT1|0.172;SNW1|0.473175;PLK1|0.415475	positive regulation of chromosome organization	GO:2001252	3.89E-03	1.94E-02	3	7
8	HIST1H4A|0.23835;RBBP7|0.218475	chromatin remodeling at centromere	GO:0031055	3.90E-03	1.95E-02	2	8
5,6	HSPD1|2.88995;ASPH|2.59965;GSN|2.4555;PSME3|0.401575;LAMTOR2|7.841025;PCNA|0.404975;RHOG|2.449775;PLEK|2.071125;ARHGDIA|3.8215;SOS2|11.48525	positive regulation of hydrolase activity	GO:0051345	3.91E-03	1.95E-02	10	6
3	HSPA8|0.3721;ACTG1|2.1999;HSP90AB1|0.458775;LCP1|2.170475;RPL3|3.30745;CCT7|2.06135;ASPH|2.59965;ATP6V1B2|2.0575;RPL4|2.063325;NSF|2.017425;KPNA2|0.186675;VDAC3|2.015475;YWHAG|2.359475;GSN|2.4555;SLC25A1|2.1121;G3BP1|0.3904;ERO1L|2.370575;RPL27|2.15885;SQSTM1|6.007525;ATG7|2.120975;MAPK14|0.483325;BTF3|0.2449;TFRC|0.403125;RHOG|2.449775;STAT3|0.4995;SLC2A1|5.081875;TXNIP|2.0553;IPO8|0.443925;PLEK|2.071125;NDE1|0.446425;HBA1|2.13735;VTN|2.01125;ACTB|2.1999;MYH10|5.579275;ALB|0.23405;PLK1|0.	establishment of localization	GO:0051234	3.91E-03	1.95E-02	36	3
6	PSME3|0.401575;TOP2A|0.481675;TTK|0.488375;PLK1|0.415475	mitotic cell cycle checkpoint	GO:0007093	3.93E-03	1.95E-02	4	6

5	HSPA8|0.3721;HSP90AB1|0.458775;HSPD1|2.88995;RPL3|3.30745;CCT7|2.06135;PABPC1|0.3285;SARS|2.2366;ASPH|2.59965;YARS|2.288275;RPL4|2.063325;NARS|2.162;HSPA4L|2.13105;DNMT1|0.172;HIST1H4A|0.23835;YWHAG|2.359475;GSN|2.4555;PABPC4|0.26245;ERO1L|2.370575;PRDX5|3.0143;BAZ1B|0.460625;PSME3|0.401575;EIF4H|0.275675;LAMTOR2|7.841025;RPL27|2.15885;SQSTM1|6.007525;ATG7|2.120975;MAPK14|0.483325;SNW1|0.473175;PLEK|2.071125;VTN|2.01125;ACTB|2.1999;TTK|0.488375;ASL|2.252975;PLK1|0.415475;PTP4A3|0.3433	cellular protein metabolic process	GO:0044267	3.93E-03	1.95E-02	37	5
4	HSPA8|0.3721;HSP90AB1|0.458775;ENO1|2.1939;HSPD1|2.88995;RPL3|3.30745;CCT7|2.06135;PABPC1|0.3285;SARS|2.2366;ASPH|2.59965;YARS|2.288275;MCM3|0.35205;RPL4|2.063325;NSF|2.017425;NARS|2.162;MCM2|0.491625;HSPA4L|2.13105;DNMT1|0.172;HIST1H4A|0.23835;KPNA2|0.186675;YWHAG|2.359475;SRRT|0.39875;GSN|2.4555;CBX3|0.485775;GTF2E1|0.300475;PABPC4|0.26245;LIG1|0.440425;ERO1L|2.370575;PRDX5|3.0143;BAZ1B|0.460625;PSME3|0.401575;RRM2|0.21675;EIF4H|0.275675;LAMTOR2|7.841025;MCMBP|0.4769;RPL27|2.15885;SQSTM1|6.007525;ATG7|2.120975;PCNA|0.404975;MAPK14|0.483325;HIST1H3A|0.455625;BTF3|0.2449;RHOG|2.449775;TOP2A|0.481675;SNW1|0.473175;STAT3|0.4995;TXNIP|2.0553;PLEK|2.071125;S100A11|2.531875;VTN|2.01125;RBBP7|0.218475;ACTB|2.1999;H3F3A|0.20985;TTK|0.488375;ASL|2.252975;PLK1|0.415475;SOD2|2.175425;PT[illegible]	cellular macromolecule metabolic process	GO:0044260	3.98E-03	1.95E-02	60	4
6,7,9,10,11	PSME3|0.401575;TTK|0.488375;PLK1|0.415475	negative regulation of ubiquitin-protein transferase activity	GO:0051444	3.99E-03	1.97E-02	3	11
4	HSPA8|0.3721;HSP90AB1|0.458775;HSPD1|2.88995;RPL3|3.30745;CCT7|2.06135;PABPC1|0.3285;SARS|2.2366;ASPH|2.59965;KRT1|0.4198;YARS|2.288275;RPL4|2.063325;NSF|2.017425;NARS|2.162;HSPA4L|2.13105;DNMT1|0.172;HIST1H4A|0.23835;YWHAG|2.359475;GSN|2.4555;CNDP2|2.0938;PABPC4|0.26245;ERO1L|2.370575;PRDX5|3.0143;BAZ1B|0.460625;PSME3|0.401575;EIF4H|0.275675;LAMTOR2|7.841025;RPL27|2.15885;SQSTM1|6.007525;ATG7|2.120975;MAPK14|0.483325;SNW1|0.473175;PLEK|2.071125;VTN|2.01125;ACTB|2.1999;DNPEP|2.077575;TTK|0.488375;ASL|2.252975;ALB|0.23405;PLK1|0.41547	protein metabolic process	GO:0019538	4.02E-03	1.97E-02	42	4
8,9	ACTG1|2.1999;ACTB|2.1999;MYH10|5.579275	ephrin receptor signaling pathway	GO:0048013	4.09E-03	1.98E-02	3	9
5,6	PSME3|0.401575;TTK|0.488375;PLK1|0.415475	negative regulation of ligase activity	GO:0051352	4.09E-03	2.01E-02	3	6
3	LCP1|2.170475;ANXA5|3.637725;GSN|2.4555;MAPK14|0.483325;RHOG|2.449775;STAT3|0.4995;NDE1|0.446425;VTN|2.01125;ARHGDIA|3.8215;MYH10|5.579275;PTP4A	localization of cell	GO:0051674	4.12E-03	2.01E-02	11	3
3	LCP1|2.170475;ANXA5|3.637725;GSN|2.4555;MAPK14|0.483325;RHOG|2.449775;STAT3|0.4995;NDE1|0.446425;VTN|2.01125;ARHGDIA|3.8215;MYH10|5.579275;PTP4A	cell motility	GO:0048870	4.12E-03	2.02E-02	11	3
6,7,8	GSN|2.4555;PLP1|3.936	oligodendrocyte development	GO:0014003	4.13E-03	2.02E-02	2	8
6	PSME3|0.401575;TTK|0.488375;PLK1|0.415475	regulation of ubiquitin-protein ligase activity involved in mitotic cell cycle	GO:0051439	4.19E-03	2.02E-02	3	6
5	TOP2A|0.481675;TTK|0.488375;PLK1|0.415475	chromosome separation	GO:0051304	4.19E-03	2.04E-02	3	5
4,5,6,7	HIST1H4A|0.23835;LIG1|0.440425;PCNA|0.404975	telomere maintenance	GO:0000723	4.19E-03	2.04E-02	3	7
6	HSPA8|0.3721;ENO1|2.1939;ATP6V1B2|2.0575;GMPS|0.496875;ALDOC|2.04725;STAT3|0.4995	ribonucleoside metabolic process	GO:0009119	4.25E-03	2.04E-02	6	6
3	ANXA5|3.637725;GSN|2.4555;MAPK14|0.483325;STAT3|0.4995;VTN|2.01125;ARHGDIA|3.8215;IFITM2|5.68315	regulation of locomotion	GO:0040012	4.34E-03	2.06E-02	7	3
4	ANXA5|3.637725;SQSTM1|6.007525;ATG7|2.120975;SLC2A1|5.081875;ALB|0.23405;CHAF1B|0.2723	response to starvation	GO:0042594	4.36E-03	2.10E-02	6	4
7,8	LIG1|0.440425;PCNA|0.404975	mitotic recombination	GO:0006312	4.36E-03	2.11E-02	2	8
7	HSPD1|2.88995;ASPH|2.59965;GSN|2.4555;PRDX5|3.0143;PSME3|0.401575;VTN|2.01125	regulation of endopeptidase activity	GO:0052548	4.48E-03	2.11E-02	6	7
6,8,9	RPL3|3.30745;RPL4|2.063325;RPL27|2.15885	SRP-dependent cotranslational protein targeting to membrane	GO:0006614	4.49E-03	2.16E-02	3	9
4,7	VIM|2.241075;GSN|2.4555;TOP2A|0.481675	execution phase of apoptosis	GO:0097194	4.49E-03	2.16E-02	3	7
6	AKR1B1|2.235325;PRDX5|3.0143;SOD2|2.175425	cellular response to reactive oxygen species	GO:0034614	4.59E-03	2.16E-02	3	6
4	ENO1|2.1939;AKR1B1|2.235325;SLC25A1|2.1121;ISYNA1|0.49065;ALDOC|2.04725;MAPK14|0.483325;STAT3|0.4995;SLC2A1|5.081875;PLEK|2.071125	single-organism carbohydrate metabolic process	GO:0044723	4.60E-03	2.19E-02	9	4
5	ATG7|2.120975;ALB|0.23405	modification by symbiont of host morphology or physiology	GO:0044003	4.61E-03	2.19E-02	2	5
6,7	ATP6V1B2|2.0575;TFRC|0.403125	transferrin transport	GO:0033572	4.61E-03	2.19E-02	2	7

6	HSPA8|0.3721;ENO1|2.1939;PABPC1|0.3285;ASPH|2.59965;DNMT1|0.172;HIST1H4A|0.23835;SRRT|0.39875;CBX3|0.485775;GTF2E1|0.300475;PRDX5|3.0143;BAZ1B|0.460625;RRM2|0.21675;EIF4H|0.275675;SQSTM1|6.007525;PCNA|0.404975;MAPK14|0.483325;HIST1H3A|0.455625;BTF3|0.2449;RHOG|2.449775;TOP2A|0.481675;SNW1|0.473175;STAT3|0.4995;TXNIP|2.0553;S100A11|2.531875;RBBP7|0.218475;H3F3A|0.20985;PLK1|0.415475;SOD2|2.175425;ZNF706|0.3974;CHAF1B|0.2723	regulation of cellular macromolecule biosynthetic process	GO:2000112	4.61E-03	2.19E-02	30	6
4,6	DNMT1|0.172;HIST1H4A|0.23835;HIST1H3A|0.455625;SNW1|0.473175;H3F3A|0.20985	macromolecule methylation	GO:0043414	4.62E-03	2.19E-02	5	6
3	GSN|2.4555;STAT3|0.4995;ARHGDIA|3.8215;IFITM2|5.68315	negative regulation of locomotion	GO:0040013	4.62E-03	2.19E-02	4	3
5	HSP90AB1|0.458775;RPL3|3.30745;PABPC1|0.3285;RPL4|2.063325;PABPC4|0.26245;LIG1|0.440425;PSME3|0.401575;RPL27|2.15885;SQSTM1|6.007525;TTK|0.488375;PL	cellular macromolecule catabolic process	GO:0044265	4.63E-03	2.19E-02	11	5
4,7	RPL3|3.30745;PABPC1|0.3285;RPL4|2.063325;EIF4H|0.275675;RPL27|2.15885	translational initiation	GO:0006413	4.67E-03	2.19E-02	5	7
5	AKR1B1|2.235325;ISYNA1|0.49065;PLEK|2.071125;GCH1|4.01735	organic hydroxy compound biosynthetic	GO:1901617	4.69E-03	2.21E-02	4	5
6,7	HSP90AB1|0.458775;DNMT1|0.172;YWHAG|2.359475;PSME3|0.401575;LAMTOR2|7.841025;SQSTM1|6.007525;ATG7|2.120975;MAPK14|0.483325;SNW1|0.473175;PLEK|2.071125;VTN|2.01125;TTK|0.488375;PLK1|0.415475	regulation of protein modification process	GO:0031399	4.69E-03	2.21E-02	13	7
2	HSPA8|0.3721;ACTG1|2.1999;HSP90AB1|0.458775;LCP1|2.170475;RPL3|3.30745;CCT7|2.06135;ASPH|2.59965;ATP6V1B2|2.0575;RPL4|2.063325;NSF|2.017425;KPNA2|0.186675;VDAC3|2.015475;YWHAG|2.359475;ANXA5|3.637725;GSN|2.4555;SLC25A1|2.1121;G3BP1|0.3904;ERO1L|2.370575;LAMTOR2|7.841025;RPL27|2.15885;SQSTM1|6.007525;ATG7|2.120975;MAPK14|0.483325;BTF3|0.2449;TFRC|0.403125;RHOG|2.449775;STAT3|0.4995;SLC2A1|5.081875;TXNIP|2.0553;IPO8|0.443925;PLEK|2.071125;NDE1|0.446425;HBA1|2.13735;VTN|2.01125;ARHGDIA|3.8215;ACTB|2.1999;MYH10|5.579275;ALB|0.23405;PLK1	localization	GO:0051179	4.70E-03	2.21E-02	40	2
6	HIST1H4A|0.23835;LIG1|0.440425;PCNA|0.404975	telomere organization	GO:0032200	4.70E-03	2.21E-02	3	6
5,6,7,9	ANXA5|3.637725;HIST1H3A|0.455625;CHAF1B|0.2723	regulation of defense response to virus by host	GO:0050691	4.81E-03	2.21E-02	3	9
6,8	ASPH|2.59965;GSN|2.4555;PLEK|2.071125	regulation of protein depolymerization	GO:1901879	4.81E-03	2.25E-02	3	8
7,8	RPL3|3.30745;PABPC1|0.3285;RPL4|2.063325;RPL27|2.1	mRNA catabolic process	GO:0006402	4.89E-03	2.25E-02	4	8
6,7,8	HSPA8|0.3721;PABPC1|0.3285;SNW1|0.473175	regulation of mRNA metabolic process	GO:1903311	4.91E-03	2.29E-02	3	8
8	HSPA8|0.3721;ENO1|2.1939;ATP6V1B2|2.0575;ALDOC|2.04725;STAT3|0.4995	purine ribonucleoside triphosphate metabolic process	GO:0009205	5.02E-03	2.29E-02	5	8
7,8	RPL3|3.30745;RPL4|2.063325;RPL27|2.15885	cotranslational protein targeting to membrane	GO:0006613	5.02E-03	2.34E-02	3	8
3	DNMT1|0.172;ALDH1L2|2.148;HIST1H4A|0.23835;HIST1H3A|0.455625;SNW1|0.473175;H3F3A|0.20985	methylation	GO:0032259	5.04E-03	2.34E-02	6	3
7	ERO1L|2.370575;MAPK14|0.483325	brown fat cell differentiation	GO:0050873	5.11E-03	2.34E-02	2	7
6,7	HSPD1|2.88995;KPNA2|0.186675;LIG1|0.440425;PCNA|0.404975;TOP2A|0.481675	DNA recombination	GO:0006310	5.13E-03	2.37E-02	5	7
4	CKB|0.486625;ATG7|2.120975;NDE1|0.446425;ACTB|2.1999;H3F3A|0.20985;MYH10|5.579275;PLP1|3.936	brain development	GO:0007420	5.14E-03	2.37E-02	7	4
7,8	ENO1|2.1939;PRDX5|3.0143;ALDOC|2.04725;STAT3|0.4995	nicotinamide nucleotide metabolic process	GO:0046496	5.17E-03	2.38E-02	4	8
6,7	ENO1|2.1939;PRDX5|3.0143;ALDOC|2.04725;STAT3|0.4995	pyridine nucleotide metabolic process	GO:0019362	5.17E-03	2.38E-02	4	7
7	HSPA8|0.3721;ENO1|2.1939;ATP6V1B2|2.0575;ALDOC|2.04725;STAT3|0.4995	ribonucleoside triphosphate metabolic	GO:0009199	5.34E-03	2.38E-02	5	7
6	MTHFD1L|6.667175;GCH1|4.01735	pteridine-containing compound biosynthetic process	GO:0042559	5.37E-03	2.38E-02	2	6
6	GSN|2.4555;PCNA|0.404975	response to cadmium ion	GO:0046686	5.37E-03	2.38E-02	2	6
6,7,9	ALDH1L2|2.148	folic acid-containing compound catabolic process	GO:0009397	5.44E-03	2.38E-02	1	9
7	HSPA8|0.3721	clathrin coat disassembly	GO:0072318	5.44E-03	2.38E-02	1	7
6,7	AKR1B1|2.235325	sorbitol metabolic process	GO:0006060	5.44E-03	2.38E-02	1	7
5,6,7	HSPD1|2.88995	T cell mediated immune response to tumor cell	GO:0002424	5.44E-03	2.38E-02	1	7
5,6,7	PLK1|0.415475	synaptonemal complex disassembly	GO:0070194	5.44E-03	2.38E-02	1	7
6,7,8	HSPD1|2.88995	positive regulation of T cell mediated immune response to tumor cell	GO:0002842	5.44E-03	2.38E-02	1	8
6,7	PCNA|0.404975	leading strand elongation	GO:0006272	5.44E-03	2.38E-02	1	7
5	SNW1|0.473175	positive regulation of vitamin D receptor signaling pathway	GO:0070564	5.44E-03	2.38E-02	1	5
5,6,7	HSPD1|2.88995	B cell cytokine production	GO:0002368	5.44E-03	2.38E-02	1	7
6,7,8,9	PLK1|0.415475	activation of APC-Cdc20 complex activity	GO:0007092	5.44E-03	2.38E-02	1	9
6	AKR1B1|2.235325	naphthalene metabolic process	GO:0018931	5.44E-03	2.38E-02	1	6

4	MYH10|5.579275	fourth ventricle development	GO:0021592	5.44E-03	2.38E-02	1	4
5,8	GCH1|4.01735	regulation of lung blood pressure	GO:0014916	5.44E-03	2.38E-02	1	8
6	STAT3|0.4995	negative regulation of hydrogen peroxide biosynthetic process	GO:0010730	5.44E-03	2.38E-02	1	6
8	AKR1B1|2.235325	fructose biosynthetic process	GO:0046370	5.44E-03	2.38E-02	1	8
7,8,9	ALDH1L2|2.148	10-formyltetrahydrofolate catabolic process	GO:0009258	5.44E-03	2.38E-02	1	9
8,9	ASPH|2.59965	peptidyl-aspartic acid hydroxylation	GO:0042264	5.44E-03	2.38E-02	1	9
5	AKR1B1|2.235325	naphthalene-containing compound metabolic process	GO:0090420	5.44E-03	2.38E-02	1	5
6,7,8	PLEK|2.071125	protein secretion by	GO:0070560	5.44E-03	2.38E-02	1	8
5,6	ALB|0.23405	maintenance of mitochondrion location	GO:0051659	5.44E-03	2.38E-02	1	6
6,7	GSN|2.4555	regulation of establishment of T cell	GO:1903903	5.44E-03	2.38E-02	1	7
8	GSN|2.4555	regulation of protein processing in phagocytic vesicle	GO:1903921	5.44E-03	2.38E-02	1	8
8	GSN|2.4555	positive regulation of protein processing in phagocytic vesicle	GO:1903923	5.44E-03	2.38E-02	1	8
6	GCH1|4.01735	dihydrobiopterin metabolic process	GO:0051066	5.44E-03	2.38E-02	1	6
8	ASPH|2.59965	peptidyl-aspartic acid modification	GO:0018197	5.44E-03	2.38E-02	1	8
6,7	HSPD1|2.88995	regulation of T cell mediated immune response to tumor cell	GO:0002840	5.44E-03	2.38E-02	1	7
7	GSN|2.4555	protein processing in phagocytic vesicle	GO:1900756	5.44E-03	2.38E-02	1	7
6	ALDH1L2|2.148	pteridine-containing compound catabolic process	GO:0042560	5.44E-03	2.38E-02	1	6
7	HSPA8|0.3721;ENO1|2.1939;ATP6V1B2|2.0575;ALDOC|2.04725;STAT3|0.4995	purine nucleoside triphosphate metabolic process	GO:0009144	5.45E-03	2.38E-02	5	7
5,6	HSP90AB1|0.458775;HSPD1|2.88995;STAT3|0.4995;SOD2|2.175425	regulation of neuron death	GO:1901214	5.47E-03	2.38E-02	4	6
5,7,8	RPL3|3.30745;RPL4|2.063325;RPL27|2.15885	protein targeting to ER	GO:0045047	5.60E-03	2.38E-02	3	8
7	ENO1|2.1939;SLC25A1|2.1121;ALDOC|2.04725;MAPK14|0.483325	glucose metabolic process	GO:0006006	5.62E-03	2.44E-02	4	7
7	HIST1H4A|0.23835;RBBP7|0.218475	histone exchange	GO:0043486	5.63E-03	2.44E-02	2	7
6,7,8,9,10	TUBG1|0.4102;NDE1|0.446425	microtubule nucleation	GO:0007020	5.63E-03	2.44E-02	2	10
3	VIM|2.241075;KRT1|0.4198;HIST1H4A|0.23835;ANXA5|3.637725;STAT3|0.4995;VTN|2.01125;ARHGDIA|3.8215;ZNF706|0.3974	negative regulation of multicellular organismal process	GO:0051241	5.77E-03	2.44E-02	8	3
6	HSP90AB1|0.458775;HSPD1|2.88995;ASPH|2.59965;GSN|2.4555;PRDX5|3.0143;PSME3|0.401575;VTN|2.01125;TTK|0.488375;PLK1|0.415475	regulation of proteolysis	GO:0030162	5.86E-03	2.50E-02	9	6
5	HSPA8|0.3721;ENO1|2.1939;ATP6V1B2|2.0575;GMPS|0.496875;ALDOC|2.04725;STAT3|0.4995	nucleoside metabolic process	GO:0009116	5.88E-03	2.53E-02	6	5
6	MYH10|5.579275;PLK1|0.415475	mitotic cytokinesis	GO:0000281	5.90E-03	2.54E-02	2	6
9,10	ENO1|2.1939;ALDOC|2.04725	NADH metabolic process	GO:0006734	5.90E-03	2.54E-02	2	10
3	SMC2|0.485025;TOP2A|0.481675;TUBG1|0.4102;PLK1|0.415475	meiotic cell cycle	GO:0051321	5.92E-03	2.54E-02	4	3
6	HSPD1|2.88995;ASPH|2.59965;GSN|2.4555;PRDX5|3.0143;PSME3|0.401575;VTN|2.01125	regulation of peptidase activity	GO:0052547	5.97E-03	2.54E-02	6	6
6	ANXA5|3.637725;SQSTM1|6.007525;CHAF1B|0.2723	activation of mitophagy in response to mitochondrial depolarization	GO:0098779	6.08E-03	2.56E-02	3	6
6,7,8	RPL3|3.30745;RPL4|2.063325;RPL27|2.15885	establishment of protein localization to endoplasmic reticulum	GO:0072599	6.08E-03	2.60E-02	3	8
5	ANXA5|3.637725;SQSTM1|6.007525;CHAF1B|0.2723	response to mitochondrial depolarisation	GO:0098780	6.08E-03	2.60E-02	3	5
5	HIST1H4A|0.23835;MAPK14|0.483325;TFRC|0.403125;TOP2A|0.481675;PLEK|2.071125;SOS2|11.48525;HEATR9|	hemopoiesis	GO:0030097	6.14E-03	2.60E-02	7	5
5,6	HSP90AB1|0.458775;DNMT1|0.172;YWHAG|2.359475;PRDX5|3.0143;PSME3|0.401575;ATG7|2.120975;VTN|2.01125;TTK|0.488375;PLK1|0.415475	negative regulation of cellular protein metabolic process	GO:0032269	6.21E-03	2.62E-02	9	6
5	HSPA8|0.3721;ENO1|2.1939;PABPC1|0.3285;ASPH|2.59965;DNMT1|0.172;HIST1H4A|0.23835;KPNA2|0.186675;SRRT|0.39875;CBX3|0.485775;GTF2E1|0.300475;PRDX5|3.0143;BAZ1B|0.460625;RRM2|0.21675;SQSTM1|6.007525;PCNA|0.404975;MAPK14|0.483325;HIST1H3A|0.455625;BTF3|0.2449;RHOG|2.449775;TOP2A|0.481675;SNW1|0.473175;STAT3|0.4995;TXNIP|2.0553;S100A11|2.531875;RBBP7|0.218475;H3F3A|0.20985;PLK1|0.415475;SOD2|2.175425;ZNF706|0.3974;CHAF1B|0.2723	regulation of nucleobase-containing compound metabolic process	GO:0019219	6.21E-03	2.64E-02	30	5
5	ENO1|2.1939;PRDX5|3.0143;ALDOC|2.04725;STAT3|0.4995	pyridine-containing compound metabolic process	GO:0072524	6.24E-03	2.64E-02	4	5

4	AKR1B1|2.235325;MAPK14|0.483325;STAT3|0.4995	muscle cell proliferation	GO:0033002	6.33E-03	2.65E-02	3	4
5,8	STAT3|0.4995;NDE1|0.446425;MYH10|5.579275	neuron migration	GO:0001764	6.33E-03	2.68E-02	3	8
4	HSPD1|2.88995;ANXA5|3.637725;HIST1H3A|0.455625;VTN|2.01125;CHAF1B|0.2723	regulation of immune effector process	GO:0002697	6.45E-03	2.68E-02	5	4
5,7	PLEK|2.071125;VTN|2.01125	positive regulation of wound healing	GO:0090303	6.46E-03	2.71E-02	2	7
5,6	VIM|2.241075;KRT9|0.268525	intermediate filament cytoskeleton organization	GO:0045104	6.46E-03	2.71E-02	2	6
6,7,8	HSP90AB1|0.458775;HSPD1|2.88995;SOD2|2.175425	negative regulation of neuron apoptotic process	GO:0043524	6.46E-03	2.71E-02	3	8
6,7,8	ANXA5|3.637725;SQSTM1|6.007525;CHAF1B|0.2723	positive regulation of mitophagy	GO:1903599	6.46E-03	2.71E-02	3	8
6	PSME3|0.401575;MAPK14|0.483325;NDRG1|10.0073	signal transduction in response to DNA damage	GO:0042770	6.46E-03	2.71E-02	3	6
5,6	HSPA8|0.3721;ENO1|2.1939;PABPC1|0.3285;ASPH|2.59965;DNMT1|0.172;HIST1H4A|0.23835;SRRT|0.39875;CBX3|0.485775;GTF2E1|0.300475;PRDX5|3.0143;BAZ1B|0.460625;RRM2|0.21675;EIF4H|0.275675;SQSTM1|6.007525;PCNA|0.404975;MAPK14|0.483325;HIST1H3A|0.455625;BTF3|0.2449;RHOG|2.449775;TOP2A|0.481675;SNW1|0.473175;STAT3|0.4995;TXNIP|2.0553;S100A11|2.531875;RBBP7|0.218475;H3F3A|0.20985;PLK1|0.415475;SOD2|2.175425;ZNF706|0.3974;CHAF1B|0.2723	regulation of macromolecule biosynthetic process	GO:0010556	6.48E-03	2.71E-02	30	6
3	ACTG1|2.1999;HSP90AB1|0.458775;LCP1|2.170475;HSPD1|2.88995;KRT1|0.4198;PSME3|0.401575;MAPK14|0.483325;TXNIP|2.0553;IK|0.480125;VTN|2.01125;ACTB|2.1999;GCH1|4.01735;IFITM2|5.68315	immune response	GO:0006955	6.64E-03	2.71E-02	13	3
8,9	PSME3|0.401575;ATG7|2.120975;TTK|0.488375;PLK1|0.415475	regulation of protein ubiquitination	GO:0031396	6.66E-03	2.77E-02	4	9
6	HSPD1|2.88995;ASPH|2.59965;GSN|2.4555;PSME3|0.401575;PLK1|0.415475	positive regulation of proteolysis	GO:0045862	6.70E-03	2.78E-02	5	6
7,8,10,11	DNMT1|0.172;SNW1|0.473175	histone H3-K4	GO:0051568	6.75E-03	2.79E-02	2	11
5,7	STAT3|0.4995;PLEK|2.071125	negative regulation of cellular carbohydrate metabolic process	GO:0010677	6.75E-03	2.80E-02	2	7
4	VIM|2.241075;KRT9|0.268525	intermediate filament-based process	GO:0045103	6.75E-03	2.80E-02	2	4
6	DNMT1|0.172;HIST1H4A|0.23835;CBX3|0.485775;BAZ1B|0.460625;ATG7|2.120975;SNW1|0.473175;RBBP7|0.218475;ACTB|2.1999	chromatin modification	GO:0016568	6.87E-03	2.80E-02	8	6
5	VIM|2.241075;STAT3|0.4995;ARHGDIA|3.8215;ZNF706|0.3974	negative regulation of cell development	GO:0010721	6.91E-03	2.84E-02	4	5
8	HSPD1|2.88995;ASPH|2.59965;GSN|2.4555;PRDX5|3.0143	regulation of cysteine-type endopeptidase activity	GO:2000116	6.91E-03	2.85E-02	4	8
5,7	ASPH|2.59965;GSN|2.4555;PLEK|2.071125	regulation of protein complex disassembly	GO:0043244	6.99E-03	2.85E-02	3	7
4	LCP1|2.170475;GSN|2.4555;MAPK14|0.483325;RHOG|2.449775;STAT3|0.4995;NDE1|0.446425;VTN|2.01125;ARHGDIA|3.8215;MYH10|5.579275;PTP4A3|0.3433	cell migration	GO:0016477	7.16E-03	2.88E-02	10	4
6	TFRC|0.403125;STAT3|0.4995;VTN|2.01125	acute inflammatory response	GO:0002526	7.26E-03	2.95E-02	3	6
5,6	NSF|2.017425;PSME3|0.401575;ATG7|2.120975;PLK1|0.415475	positive regulation of protein catabolic process	GO:0045732	7.45E-03	2.98E-02	4	6
6	HSPA8|0.3721;ENO1|2.1939;ATP6V1B2|2.0575;ALDOC|2.04725;STAT3|0.4995	nucleoside triphosphate metabolic process	GO:0009141	7.49E-03	3.06E-02	5	6
5,6	HSP90AB1|0.458775;MAPK14|0.483325;STAT3|0.4995	regulation of reactive oxygen species metabolic process	GO:2000377	7.54E-03	3.07E-02	3	6
4	HSPD1|2.88995;PLEK|2.071125;VTN|2.01125	positive regulation of response to wounding	GO:1903036	7.54E-03	3.08E-02	3	4
5	HSPD1|2.88995;ASPH|2.59965;GSN|2.4555;PRDX5|3.0143;PSME3|0.401575;LAMTOR2|7.841025;PCNA|0.404975;RHOG|2.449775;PLEK|2.071125;VTN|2.01125;ARHGDIA|3.8215;SOS2|11.48525	regulation of hydrolase activity	GO:0051336	7.69E-03	3.08E-02	12	5
6	ACTG1|2.1999;ATG7|2.120975;MAPK14|0.483325;MYH10|5.579275	striated muscle cell differentiation	GO:0051146	7.73E-03	3.14E-02	4	6
8,9,10,11	PSME3|0.401575;TTK|0.488375;PLK1|0.415475	anaphase-promoting complex-dependent proteasomal ubiquitin-dependent protein catabolic process	GO:0031145	7.83E-03	3.15E-02	3	11
6,9,10	PSME3|0.401575;TTK|0.488375;PLK1|0.415475	regulation of ubiquitin-protein transferase activity	GO:0051438	7.83E-03	3.15E-02	3	10
4	HIST1H4A|0.23835;MAPK14|0.483325;TFRC|0.403125;TOP2A|0.481675;PLEK|2.071125;SOS2|11.48525;HEATR9|	hematopoietic or lymphoid organ	GO:0048534	7.94E-03	3.15E-02	7	4
5	HSPD1|2.88995;ASPH|2.59965	response to ATP	GO:0033198	7.96E-03	3.15E-02	2	5
6,7,9,10	DNMT1|0.172;SNW1|0.473175	regulation of histone methylation	GO:0031060	7.96E-03	3.15E-02	2	10
7,8	ATP6V1B2|2.0575;TFRC|0.403125	trivalent inorganic cation transport	GO:0072512	7.96E-03	3.15E-02	2	8
8,9,10,11	ATP6V1B2|2.0575;TFRC|0.403125	ferric iron transport	GO:0015682	7.96E-03	3.15E-02	2	11
5,6,7,8	LIG1|0.440425;PCNA|0.404975	telomere maintenance via telomere lengthening	GO:0010833	7.96E-03	3.15E-02	2	8
5,6	HSP90AB1|0.458775;TTK|0.488375;PLK1|0.415475	negative regulation of protein catabolic process	GO:0042177	7.97E-03	3.15E-02	3	6
3	GSN|2.4555;SQSTM1|6.007525;IFITM2|5.68315	negative regulation of multi-organism process	GO:0043901	8.12E-03	3.15E-02	3	3
4	MYH10|5.579275	third ventricle	GO:0021678	8.16E-03	3.15E-02	1	4

8,9,12,13,14	ATG7|2.120975	negative regulation of histone H4-K16	GO:2000619	8.16E-03	3.15E-02	1	14
6,7,9,10	ALB|0.23405	hemolysis by symbiont of host erythrocytes	GO:0019836	8.16E-03	3.15E-02	1	10
7,8	HIST1H4A|0.23835	histone H4-K20 demethylation	GO:0035574	8.16E-03	3.15E-02	1	8
6,7,8,9	PLK1|0.415475	female meiosis chromosome segregation	GO:0016321	8.16E-03	3.15E-02	1	9
6	STAT3|0.4995	regulation of hydrogen peroxide biosynthetic process	GO:0010728	8.16E-03	3.15E-02	1	6
5	ALB|0.23405	cytolysis in other organism involved in symbiotic interaction	GO:0051801	8.16E-03	3.15E-02	1	5
5	AKR1B1|2.235325	inner medullary collecting duct development	GO:0072061	8.16E-03	3.15E-02	1	5
4	SNW1|0.473175	positive regulation of response to alcohol	GO:1901421	8.16E-03	3.15E-02	1	4
5,6	AKR1B1|2.235325	hexitol metabolic process	GO:0006059	8.16E-03	3.15E-02	1	6
6,7,8,9	SLC2A1|5.081875	dehydroascorbic acid transport	GO:0070837	8.16E-03	3.15E-02	1	9
6,7	HSPA8|0.3721	regulation of fibril organization	GO:1902903	8.16E-03	3.15E-02	1	7
4	ALB|0.23405	cytolysis in other	GO:0051715	8.16E-03	3.15E-02	1	4
6,8,9	ALB|0.23405	cytolysis by symbiont of host cells	GO:0001897	8.16E-03	3.15E-02	1	9
6	AKR1B1|2.235325	response to methylglyoxal	GO:0051595	8.16E-03	3.15E-02	1	6
4	ARHGDIA|3.8215	cellular response to redox state	GO:0071461	8.16E-03	3.15E-02	1	4
5,6	STAT3|0.4995	positive regulation of skeletal muscle cell proliferation	GO:0014858	8.16E-03	3.15E-02	1	6
8,9,10,11	GSN|2.4555	positive regulation of keratinocyte apoptotic process	GO:1902174	8.16E-03	3.15E-02	1	11
5	ALB|0.23405	hemolysis in other organism	GO:0044179	8.16E-03	3.15E-02	1	5
5	ALB|0.23405	hemolysis in other organism involved in symbiotic interaction	GO:0052331	8.16E-03	3.15E-02	1	5
7	GSN|2.4555	renal protein absorption	GO:0097017	8.16E-03	3.15E-02	1	7
6,7	MAPK14|0.483325	myoblast differentiation involved in skeletal muscle regeneration	GO:0014835	8.16E-03	3.15E-02	1	7
5	SOD2|2.175425	age-dependent response to oxidative stress	GO:0001306	8.16E-03	3.15E-02	1	5
4	SOD2|2.175425	age-dependent general metabolic decline	GO:0007571	8.16E-03	3.15E-02	1	4
7,8	VDAC3|2.015475	adenine transport	GO:0015853	8.16E-03	3.15E-02	1	8
6,7,8	MAPK14|0.483325	positive regulation of interleukin-12 secretion	GO:2001184	8.16E-03	3.15E-02	1	8
5,6	HSP90AB1|0.458775;LCP1|2.170475;ASPH|2.59965;YWHAG|2.359475;MAPK14|0.483325;RHOG|2.449775;SLC2A1|5.081875	regulation of establishment of protein localization	GO:0070201	8.19E-03	3.15E-02	7	6
7,8	PSME3|0.401575;ATG7|2.120975;TTK|0.488375;PLK1|0.415475	regulation of protein modification by small protein conjugation or removal	GO:1903320	8.20E-03	3.16E-02	4	8
6,7	YWHAG|2.359475;ANXA5|3.637725;SQSTM1|6.007525;CHAF1B|0.2723	positive regulation of mitochondrion	GO:0010822	8.20E-03	3.16E-02	4	7
4	ATG7|2.120975;MYH10|5.579275	cerebellar cortex development	GO:0021695	8.28E-03	3.16E-02	2	4
5,8	GCH1|4.01735;SOD2|2.175425	negative regulation of blood pressure	GO:0045776	8.28E-03	3.18E-02	2	8
5,6,7	HSP90AB1|0.458775;LAMTOR2|7.841025;SQSTM1|6.007525;MAPK14|0.483325;STAT3|0.4995;PLEK|2.071125;VTN|2.01125;TTK|0.488375;PLK1|0.415475	positive regulation of phosphorus metabolic process	GO:0010562	8.33E-03	3.18E-02	9	7
6	HSP90AB1|0.458775;LAMTOR2|7.841025;SQSTM1|6.007525;MAPK14|0.483325;STAT3|0.4995;PLEK|2.071125;VTN|2.01125;TTK|0.488375;PLK1|0.415475	positive regulation of phosphate metabolic process	GO:0045937	8.33E-03	3.19E-02	9	6
7,8,9	ENO1|2.1939;DNMT1|0.172;SNW1|0.473175;STAT3|0.4995;TXNIP|2.0553;RBBP7|0.218475;PLK1|0.415475	negative regulation of transcription from RNA polymerase II promoter	GO:0000122	8.40E-03	3.19E-02	7	9
6	AKR1B1|2.235325;ISYNA1|0.49065;PLEK|2.071125	polyol metabolic process	GO:0019751	8.42E-03	3.21E-02	3	6
5	HSP90AB1|0.458775;HSPD1|2.88995;STAT3|0.4995;SOD2|2.175425	neuron death	GO:0070997	8.50E-03	3.21E-02	4	5
4,5	AKR1B1|2.235325;ISYNA1|0.49065;STAT3|0.4995;SLC2A1|5.081875;PLEK|2.071125	cellular carbohydrate metabolic process	GO:0044262	8.50E-03	3.24E-02	5	5
5	ENO1|2.1939;ALDOC|2.04725;STAT3|0.4995	glycolytic process	GO:0006096	8.57E-03	3.24E-02	3	5
6,7,8	ANXA5|3.637725;SQSTM1|6.007525;ATG7|2.120975;CHAF1B|0.2723	mitophagy	GO:0000422	8.60E-03	3.24E-02	4	8
6,7	ANXA5|3.637725;SQSTM1|6.007525;ATG7|2.120975;CHAF1B|0.2723	mitochondrion disassembly	GO:0061726	8.60E-03	3.24E-02	4	7
6	MYH10|5.579275;PLK1|0.415475	cytoskeleton-dependent cytokinesis	GO:0061640	8.60E-03	3.24E-02	2	6
6,8,9	LIG1|0.440425;PCNA|0.404975	transcription-coupled nucleotide-excision repair	GO:0006283	8.60E-03	3.24E-02	2	9

5,7	AKR1B1|2.235325;GCH1|4.01735	catecholamine metabolic process	GO:0006584	8.60E-03	3.24E-02	2	7
5,6	HSPD1|2.88995;MAPK14|0.483325	regulation of interleukin-12 production	GO:0032655	8.60E-03	3.24E-02	2	6
5	LIG1|0.440425;PCNA|0.404975	nuclear DNA replication	GO:0033260	8.60E-03	3.24E-02	2	5
6	AKR1B1|2.235325;GCH1|4.01735	catechol-containing compound metabolic process	GO:0009712	8.60E-03	3.24E-02	2	6
5	HSP90AB1|0.458775;DNMT1|0.172;YWHAG|2.359475;PRDX5|3.0143;PSME3|0.401575;ATG7|2.120975;VTN|2.01125;TTK|0.488375;PLK1|0.415475	negative regulation of protein metabolic process	GO:0051248	8.70E-03	3.24E-02	9	5
8,9,10	ENO1|2.1939;ALDOC|2.04725;STAT3|0.4995	ATP generation from	GO:0006757	8.72E-03	3.27E-02	3	10
5	HSP90AB1|0.458775;VTN|2.01125;PLK1|0.415475	regulation of protein	GO:0043393	8.72E-03	3.27E-02	3	5
5	PSME3|0.401575;TTK|0.488375;PLK1|0.415475	regulation of ligase	GO:0051340	8.88E-03	3.27E-02	3	5
5	HSPD1|2.88995;MAPK14|0.483325	interleukin-12 production	GO:0032615	8.93E-03	3.32E-02	2	5
6,7	TFRC|0.403125;NCDN|0.4114	bone resorption	GO:0045453	8.93E-03	3.32E-02	2	7
5,7,8	DNMT1|0.172;ATG7|2.120975	negative regulation of histone modification	GO:0031057	8.93E-03	3.32E-02	2	8
7	MAPK14|0.483325;PTP4A3|0.3433	cellular response to vascular endothelial growth factor stimulus	GO:0035924	8.93E-03	3.32E-02	2	7
6,7	LCP1|2.170475;GSN|2.4555	morphogenesis of an epithelial sheet	GO:0002011	8.93E-03	3.32E-02	2	7
6,7	DNMT1|0.172;YWHAG|2.359475;PSME3|0.401575;ATG7|2.120975;TTK|0.488375;PLK1|0.415475	negative regulation of protein modification process	GO:0031400	8.95E-03	3.32E-02	6	7
7	RPL3|3.30745;RPL4|2.063325;RPL27|2.15885	protein localization to endoplasmic reticulum	GO:0070972	9.03E-03	3.32E-02	3	7
8,9,10	PSME3|0.401575;TTK|0.488375;PLK1|0.415475	negative regulation of protein ubiquitination	GO:0031397	9.03E-03	3.34E-02	3	10
5	ATP6V1B2|2.0575;DNMT1|0.172;SRRT|0.39875;AKR1B1|2.235325;LAMTOR2|7.841025;STAT3|0.4995	cellular response to nitrogen compound	GO:1901699	9.08E-03	3.34E-02	6	5
5,6,7	PSME3|0.401575;TOP2A|0.481675;TTK|0.488375;PLK1|0.415475	negative regulation of mitotic cell cycle	GO:0045930	9.11E-03	3.36E-02	4	7
4,5	ACTG1|2.1999;LCP1|2.170475;HSPD1|2.88995;GSN|2.4555;PLEK|2.071125;ACTB|2.1999;SOS2|11.48525	single organismal cell-cell adhesion	GO:0016337	9.25E-03	3.36E-02	7	5
7	TFRC|0.403125;STAT3|0.4995	acute-phase response	GO:0006953	9.26E-03	3.41E-02	2	7
4,6	STAT3|0.4995;PLEK|2.071125	negative regulation of carbohydrate metabolic process	GO:0045912	9.26E-03	3.41E-02	2	6
6	ENO1|2.1939;PRDX5|3.0143;ALDOC|2.04725;STAT3|0.4995	oxidoreduction coenzyme metabolic process	GO:0006733	9.32E-03	3.41E-02	4	6
5,6	HSPD1|2.88995;KRT1|0.4198;MAPK14|0.483325;VTN|2.01125	regulation of inflammatory response	GO:0050727	9.32E-03	3.41E-02	4	6
4	HSPD1|2.88995;ATG7|2.120975;TFRC|0.403125;NDRG1|10.0073	response to oxygen levels	GO:0070482	9.32E-03	3.41E-02	4	4
5	DNMT1|0.172;ATG7|2.120975;SNW1|0.473175	regulation of histone modification	GO:0031056	9.35E-03	3.41E-02	3	5
6	DNMT1|0.172;LAMTOR2|7.841025	cellular response to amino acid stimulus	GO:0071230	9.60E-03	3.42E-02	2	6
6,7	HSP90AB1|0.458775;TXNIP|2.0553	nucleotide-binding domain, leucine rich repeat containing receptor signaling pathway	GO:0035872	9.60E-03	3.50E-02	2	7
6	AKR1B1|2.235325;ISYNA1|0.49065;PLEK|2.071125	alcohol biosynthetic	GO:0046165	9.68E-03	3.50E-02	3	6
6,7	PABPC1|0.3285;SRRT|0.39875;EIF4H|0.275675;MAPK14|0.483325;ZNF706|0.3974	posttranscriptional regulation of gene expression	GO:0010608	9.76E-03	3.52E-02	5	7
3,5	ASPH|2.59965;MAPK14|0.483325;TFRC|0.403125	positive regulation of homeostatic process	GO:0032846	9.84E-03	3.55E-02	3	5
4	KRT1|0.4198;ANXA5|3.637725;VTN|2.01125	negative regulation of response to wounding	GO:1903035	9.84E-03	3.56E-02	3	4
6,7	ACTG1|2.1999;ATG7|2.120975;MYH10|5.579275	striated muscle cell development	GO:0055002	9.84E-03	3.56E-02	3	7
4,5	YWHAG|2.359475;ANXA5|3.637725;PRDX5|3.0143;PSME3|0.401575;TXNIP|2.0553;VTN|2.01125;TTK|0.488375;PLK1|0.415475	negative regulation of catalytic activity	GO:0043086	9.92E-03	3.56E-02	8	5
6,7,8	ATG7|2.120975;MYH10|5.579275	cardiac muscle cell development	GO:0055013	9.95E-03	3.59E-02	2	8
5	MAPK14|0.483325;STAT3|0.4995	striated muscle cell proliferation	GO:0014855	9.95E-03	3.59E-02	2	5
7,8,9	PSME3|0.401575;TTK|0.488375;PLK1|0.415475	negative regulation of protein modification by small protein conjugation or removal	GO:1903321	1.00E-02	3.59E-02	3	9
4	HSPA8|0.3721;ENO1|2.1939;ATP6V1B2|2.0575;GMPS|0.496875;PRDX5|3.0143;ISYNA1|0.49065;ALDOC|2.04725;RRM2|0.21675;STAT3|0.4995;PLEK|2.071125;GCH1|4.01	organophosphate metabolic process	GO:0019637	1.01E-02	3.61E-02	11	4
5,6	ANXA5|3.637725;SQSTM1|6.007525;ATG7|2.120975;CHAF1B|0.2723	organelle disassembly	GO:1903008	1.01E-02	3.62E-02	4	6

4	HSPA8|0.3721;ACTG1|2.1999;HSP90AB1|0.458775;LCP1|2.170475;RPL3|3.30745;CCT7|2.06135;ASPH|2.59965;ATP6V1B2|2.0575;RPL4|2.063325;NSF|2.017425;KPNA2|0.186675;VDAC3|2.015475;YWHAG|2.359475;GSN|2.4555;SLC25A1|2.1121;G3BP1|0.3904;ERO1L|2.370575;RPL27|2.15885;SQSTM1|6.007525;ATG7|2.120975;MAPK14|0.483325;BTF3|0.2449;TFRC|0.403125;STAT3|0.4995;SLC2A1|5.081875;TXNIP|2.0553;IPO8|0.443925;PLEK|2.071125;NDE1|0.446425;HBA1|2.13735;VTN|2.01125;ACTB|2.1999;	transport	GO:0006810	1.02E-02	3.62E-02	34	4
4	HSPA8|0.3721;HSP90AB1|0.458775;PABPC1|0.3285;ASPH|2.59965;BAZ1B|0.460625;SQSTM1|6.007525;PCNA|0.404975;MAPK14|0.483325;RHOG|2.449775;TOP2A|0.481675;SNW1|0.473175;STAT3|0.4995	positive regulation of nitrogen compound metabolic process	GO:0051173	1.03E-02	3.67E-02	12	4
5	RHOG|2.449775;PLEK|2.071125	ruffle organization	GO:0031529	1.03E-02	3.68E-02	2	5
7	ENO1|2.1939;ALDOC|2.04725	hexose catabolic process	GO:0019320	1.03E-02	3.68E-02	2	7
6,8	MTHFD1L|6.667175;ASL|2.252975;GCH1|4.01735	alpha-amino acid biosynthetic process	GO:1901607	1.03E-02	3.68E-02	3	8
4	SRRT|0.39875;STAT3|0.4995;ZNF706|0.3974	stem cell maintenance	GO:0019827	1.03E-02	3.68E-02	3	4
8,9	ENO1|2.1939;ALDOC|2.04725;STAT3|0.4995	ADP metabolic process	GO:0046031	1.03E-02	3.68E-02	3	9
4,6	ACTG1|2.1999;HSP90AB1|0.458775;HSPD1|2.88995;PSME3|0.401575;MAPK14|0.483325;ACTB|2.1999	immune response-activating signal transduction	GO:0002757	1.05E-02	3.68E-02	6	6
4	HSP90AB1|0.458775;PABPC1|0.3285;ASPH|2.59965;PRDX5|3.0143;BAZ1B|0.460625;SQSTM1|6.007525;PCNA|0.404975;MAPK14|0.483325;RHOG|2.449775;TOP2A|0.481675;SNW1|0.473175;STAT3|0.4995	positive regulation of biosynthetic process	GO:0009891	1.06E-02	3.72E-02	12	4
5,6,7	HSPA8|0.3721;ENO1|2.1939;PABPC1|0.3285;ASPH|2.59965;DNMT1|0.172;HIST1H4A|0.23835;SRRT|0.39875;CBX3|0.485775;GTF2E1|0.300475;PRDX5|3.0143;BAZ1B|0.460625;RRM2|0.21675;SQSTM1|6.007525;PCNA|0.404975;MAPK14|0.483325;HIST1H3A|0.455625;BTF3|0.2449;RHOG|2.449775;TOP2A|0.481675;SNW1|0.473175;STAT3|0.4995;TXNIP|2.0553;RBBP7|0.218475;H3F3A|0.20985;PLK1|0.415475;SOD2|2.175425;ZNF706|0.3974;CHAF1B|0.2	regulation of RNA metabolic process	GO:0051252	1.06E-02	3.72E-02	28	7
3	SRRT|0.39875;STAT3|0.4995;ZNF706|0.3974	maintenance of cell number	GO:0098727	1.07E-02	3.72E-02	3	3
7,9,10	SARS|2.2366;YARS|2.288275;NARS|2.162	tRNA aminoacylation for protein translation	GO:0006418	1.07E-02	3.72E-02	3	10
5	HSP90AB1|0.458775;NSF|2.017425;PSME3|0.401575;ATG7|2.120975;TTK|0.488375;PLK1|0.415475	regulation of protein catabolic process	GO:0042176	1.07E-02	3.72E-02	6	5
4	ANXA5|3.637725;GSN|2.4555;MAPK14|0.483325;STAT3|0.4995;VTN|2.01125;ARHGDIA|3.8215	regulation of cell motility	GO:2000145	1.08E-02	3.72E-02	6	4
5,6	AKR1B1|2.235325	alditol biosynthetic	GO:0019401	1.09E-02	3.72E-02	1	6
8,9,10,11	STAT3|0.4995	positive regulation of ATP biosynthetic process	GO:2001171	1.09E-02	3.72E-02	1	11
5,6,7	SLC2A1|5.081875	lactose metabolic process	GO:0005988	1.09E-02	3.72E-02	1	7
8,9,10,11	PABPC1|0.3285	negative regulation of nuclear-transcribed mRNA catabolic process, nonsense-mediated decay	GO:2000623	1.09E-02	3.72E-02	1	11
8,9,10	GSN|2.4555	regulation of keratinocyte apoptotic process	GO:1902172	1.09E-02	3.72E-02	1	10
9,10	CKB|0.486625	cellular chloride ion homeostasis	GO:0030644	1.09E-02	3.72E-02	1	10
5,6	SLC2A1|5.081875	disaccharide biosynthetic process	GO:0046351	1.09E-02	3.72E-02	1	6
8,9	SLC25A1|2.1121	citrate transport	GO:0015746	1.09E-02	3.72E-02	1	9
7,8	ALDH1L2|2.148	10-formyltetrahydrofolate metabolic process	GO:0009256	1.09E-02	3.72E-02	1	8
6	GSN|2.4555	membrane raft	GO:0001766	1.09E-02	3.72E-02	1	6
5	GSN|2.4555	membrane raft distribution	GO:0031580	1.09E-02	3.72E-02	1	5
7	ASL|2.252975	arginine biosynthetic process via ornithine	GO:0042450	1.09E-02	3.72E-02	1	7
8,10,11	YARS|2.288275	tyrosyl-tRNA aminoacylation	GO:0006437	1.09E-02	3.72E-02	1	11
4,5	GSN|2.4555	amyloid fibril formation	GO:1990000	1.09E-02	3.72E-02	1	5
7,8	SLC25A1|2.1121	tricarboxylic acid	GO:0006842	1.09E-02	3.72E-02	1	8
7,9,10,11	DNMT1|0.172	C-5 methylation of	GO:0090116	1.09E-02	3.72E-02	1	11
9	GSN|2.4555	keratinocyte apoptotic process	GO:0097283	1.09E-02	3.72E-02	1	9
6	RBBP7|0.218475	cellular heat acclimation	GO:0070370	1.09E-02	3.72E-02	1	6
5,6,7	PLEK|2.071125	positive regulation of platelet activation	GO:0010572	1.09E-02	3.72E-02	1	7
7,8,9,10	PABPC1|0.3285	regulation of nuclear-transcribed mRNA catabolic process, nonsense-mediated decay	GO:2000622	1.09E-02	3.72E-02	1	10
6,7	PCNA|0.404975	regulation of deoxyribonuclease activity	GO:0032070	1.09E-02	3.72E-02	1	7
6,7,8	SLC2A1|5.081875	lactose biosynthetic	GO:0005989	1.09E-02	3.72E-02	1	8
6,7,8,9	G3BP1|0.3904;PSME3|0.401575;MAPK14|0.483325	negative regulation of canonical Wnt signaling pathway	GO:0090090	1.09E-02	3.72E-02	3	9
6	ACTG1|2.1999;HSP90AB1|0.458775;GSN|2.4555;TFRC|0.403125;HBA1|2.13735;VTN|2.01125;ACTB|2.1999;ALB|0.	endocytosis	GO:0006897	1.09E-02	3.72E-02	8	6
5	VIM|2.241075;YWHAG|2.359475;SRRT|0.39875;SNW1|0.473175;STAT3|0.4995;ARHGDIA|3.8215;ZNF706|0.3974	regulation of cell development	GO:0060284	1.10E-02	3.74E-02	7	5

6	ANXA5|3.637725;SQSTM1|6.007525;ATG7|2.120975;CHAF1B|0.2723	regulation of autophagy	GO:0010506	1.10E-02	3.75E-02	4	6
5,6	ATG7|2.120975;MYH10|5.579275	cardiac cell development	GO:0055006	1.10E-02	3.76E-02	2	6
8	ENO1|2.1939;ALDOC|2.04725;STAT3|0.4995	purine ribonucleoside diphosphate metabolic process	GO:0009179	1.12E-02	3.76E-02	3	8
7	ENO1|2.1939;ALDOC|2.04725;STAT3|0.4995	purine nucleoside diphosphate metabolic process	GO:0009135	1.12E-02	3.82E-02	3	7
4	BAZ1B|0.460625;ATG7|2.120975;PCNA|0.404975;MAPK14|0.483325;MYH10|5.579275	heart development	GO:0007507	1.12E-02	3.82E-02	5	4
7	HSP90AB1|0.458775;KPNA2|0.186675;MAPK14|0.483325;STAT3|0.4995;TXNIP|2.0553	nucleocytoplasmic transport	GO:0006913	1.13E-02	3.82E-02	5	7
5,6,7	KPNA2|0.186675;PCNA|0.404975;TOP2A|0.481675;S100A11|2.531875	regulation of DNA metabolic process	GO:0051052	1.14E-02	3.85E-02	4	7
7,8,9,10	HSPA8|0.3721;SNW1|0.473175	regulation of mRNA splicing, via spliceosome	GO:0048024	1.14E-02	3.85E-02	2	10
4,5	GSN|2.4555;MAPK14|0.483325	tissue regeneration	GO:0042246	1.14E-02	3.85E-02	2	5
8,9	HSPD1|2.88995;ASPH|2.59965;GSN|2.4555	positive regulation of cysteine-type endopeptidase activity	GO:2001056	1.14E-02	3.85E-02	3	9
3,4	ENO1|2.1939;LAMTOR2|7.841025;TFRC|0.403125;RBBP7|0.218475;H3F3A|0.20985	cell growth	GO:0016049	1.15E-02	3.85E-02	5	4
7	ENO1|2.1939;ALDOC|2.04725;STAT3|0.4995	ribonucleoside diphosphate metabolic	GO:0009185	1.16E-02	3.89E-02	3	7
6	NDE1|0.446425;PLK1|0.415475;CETN3|0.4171	centrosome organization	GO:0051297	1.16E-02	3.90E-02	3	6
8	YWHAG|2.359475;GTF2E1|0.300475;PRDX5|3.0143;SNW1|0.473175	transcription initiation from RNA polymerase II promoter	GO:0006367	1.16E-02	3.90E-02	4	8
6,7	MCM3|0.35205;MCM2|0.491625	DNA replication initiation	GO:0006270	1.18E-02	3.90E-02	2	7
5	LIG1|0.440425;PCNA|0.404975	cell cycle DNA replication	GO:0044786	1.18E-02	3.94E-02	2	5
5	DNMT1|0.172;LAMTOR2|7.841025;SNW1|0.473175	cellular response to acid chemical	GO:0071229	1.18E-02	3.94E-02	3	5
6	HSP90AB1|0.458775;KPNA2|0.186675;MAPK14|0.483325;STAT3|0.4995;TXNIP|2.0553	nuclear transport	GO:0051169	1.18E-02	3.94E-02	5	6
6,8,9	SARS|2.2366;YARS|2.288275;NARS|2.162	tRNA aminoacylation	GO:0043039	1.19E-02	3.95E-02	3	9
5	SARS|2.2366;YARS|2.288275;NARS|2.162	amino acid activation	GO:0043038	1.19E-02	3.99E-02	3	5
3,4	ASPH|2.59965;GSN|2.4555;ERO1L|2.370575;ALB|0.2340	maintenance of location	GO:0051235	1.20E-02	3.99E-02	4	4
3	VIM|2.241075;HIST1H4A|0.23835;SRRT|0.39875;STAT3|0.4995;VAT1|2.702925;ARHGDIA|3.8215;ZNF706|0.3974	negative regulation of developmental process	GO:0051093	1.21E-02	3.99E-02	7	3
5	GSN|2.4555;SNW1|0.473175;STAT3|0.4995;TXNIP|2.055	response to alcohol	GO:0097305	1.25E-02	4.04E-02	4	5
4	VIM|2.241075;HIST1H4A|0.23835;SRRT|0.39875;STAT3|0.4995;ARHGDIA|3.8215;ZNF706|0.3974	negative regulation of cell differentiation	GO:0045596	1.25E-02	4.13E-02	6	4
3,4	ACTG1|2.1999;LCP1|2.170475;HSPD1|2.88995;GSN|2.4555;PLEK|2.071125;ACTB|2.1999;SOS2|11.48525	single organism cell adhesion	GO:0098602	1.25E-02	4.13E-02	7	4
5,6	ACTG1|2.1999;ATG7|2.120975;MYH10|5.579275	muscle cell development	GO:0055001	1.25E-02	4.13E-02	3	6
6	HIST1H4A|0.23835;RBBP7|0.218475	centromere complex assembly	GO:0034508	1.25E-02	4.13E-02	2	6
5	GSN|2.4555;ATG7|2.120975	membrane invagination	GO:0010324	1.25E-02	4.13E-02	2	5
6,7,8,9,10,11,12,13,14	TTK|0.488375;PLK1|0.415475	mitotic spindle assembly checkpoint	GO:0007094	1.25E-02	4.13E-02	2	14
6,7	YWHAG|2.359475;ERO1L|2.370575;SNW1|0.473175;SOD2|2.175425	intrinsic apoptotic signaling pathway	GO:0097193	1.27E-02	4.13E-02	4	7
6	HSPA8|0.3721;ENO1|2.1939;ATP6V1B2|2.0575;GMPS|0.496875;PRDX5|3.0143;ALDOC|2.04725;RRM2|0.21675;STAT3|0.4995	nucleotide metabolic process	GO:0009117	1.27E-02	4.19E-02	8	6
5,7	MTHFD1L|6.667175;ASL|2.252975;GCH1|4.01735	cellular amino acid biosynthetic process	GO:0008652	1.29E-02	4.19E-02	3	7
5	HSP90AB1|0.458775;STAT3|0.4995	regulation of reactive oxygen species biosynthetic process	GO:1903426	1.29E-02	4.23E-02	2	5
7	TTK|0.488375;PLK1|0.415475	spindle assembly checkpoint	GO:0071173	1.29E-02	4.23E-02	2	7
6,7	HSPA8|0.3721;ENO1|2.1939;ASPH|2.59965;DNMT1|0.172;HIST1H4A|0.23835;SRRT|0.39875;CBX3|0.485775;GTF2E1|0.300475;PRDX5|3.0143;BAZ1B|0.460625;RRM2|0.21675;SQSTM1|6.007525;PCNA|0.404975;MAPK14|0.483325;HIST1H3A|0.455625;BTF3|0.2449;RHOG|2.449775;TOP2A|0.481675;SNW1|0.473175;STAT3|0.4995;TXNIP|2.0553;RBBP7|0.218475;H3F3A|0.20985;PLK1|0.415475;SOD2|2.175425;ZNF706|0.3974;CHAF1B|0.2723	regulation of transcription, DNA-templated	GO:0006355	1.30E-02	4.23E-02	27	7
3	HSPA8|0.3721;ACTG1|2.1999;HSP90AB1|0.458775;RPL3|3.30745;ASPH|2.59965;ATP6V1B2|2.0575;RPL4|2.063325;NSF|2.017425;KPNA2|0.186675;VDAC3|2.015475;YWHAG|2.359475;GSN|2.4555;SLC25A1|2.1121;ERO1L|2.370575;RPL27|2.15885;SQSTM1|6.007525;ATG7|2.120975;MAPK14|0.483325;TFRC|0.403125;RHOG|2.449775;STAT3|0.4995;SLC2A1|5.081875;TXNIP|2.0553;PLEK|2.071125;NDE1|0.446425;HBA1|2.13735;ACTB|2.1999;MYH10|5.5	single-organism localization	GO:1902578	1.31E-02	4.26E-02	29	3
6	HSP90AB1|0.458775;VTN|2.01125	positive regulation of protein binding	GO:0032092	1.33E-02	4.28E-02	2	6
6,7,8,9,10,11,12,13	TTK|0.488375;PLK1|0.415475	mitotic spindle checkpoint	GO:0071174	1.33E-02	4.28E-02	2	13
6,7,8,9,10	TTK|0.488375;PLK1|0.415475	negative regulation of mitotic sister chromatid segregation	GO:0033048	1.33E-02	4.28E-02	2	10
5,6	TTK|0.488375;PLK1|0.415475	negative regulation of sister chromatid	GO:0033046	1.33E-02	4.28E-02	2	6

6,7,8,9,10,11,12	TTK|0.488375;PLK1|0.415475	negative regulation of mitotic metaphase/anaphase transition	GO:0045841	1.33E-02	4.28E-02	2	12
7,8,9	TTK|0.488375;PLK1|0.415475	negative regulation of mitotic sister chromatid separation	GO:2000816	1.33E-02	4.28E-02	2	9
5	HSPD1|2.88995;ASPH|2.59965;GSN|2.4555;ERO1L|2.370575;VTN|2.01125	protein maturation	GO:0051604	1.35E-02	4.28E-02	5	5
6	GCH1|4.01735;SOD2|2.175425;PTP4A3|0.3433	vascular process in circulatory system	GO:0003018	1.35E-02	4.28E-02	3	6
4	VAT1|2.702925	negative regulation of mitochondrial fusion	GO:0010637	1.36E-02	4.28E-02	1	4
7,8,11,12,13	ATG7|2.120975	negative regulation of histone H4 acetylation	GO:0090241	1.36E-02	4.28E-02	1	13
7,8,9	PABPC1|0.3285	negative regulation of mRNA catabolic process	GO:1902373	1.36E-02	4.28E-02	1	9
6,7	ALB|0.23405	disruption by symbiont of host cell	GO:0044004	1.36E-02	4.28E-02	1	7
7,8,9	SARS|2.2366	selenocysteinyl-tRNA(Sec) biosynthetic	GO:0097056	1.36E-02	4.28E-02	1	9
5,7,8	ALB|0.23405	killing by symbiont of host cells	GO:0001907	1.36E-02	4.28E-02	1	8
5	RBBP7|0.218475	heat acclimation	GO:0010286	1.36E-02	4.28E-02	1	5
5,6,7	GSN|2.4555	regulation of wound healing, spreading of epidermal cells	GO:1903689	1.36E-02	4.28E-02	1	7
8,11,12,13	ATG7|2.120975	regulation of histone H4-K16 acetylation	GO:2000618	1.36E-02	4.28E-02	1	13
6	HIST1H4A|0.23835	negative regulation of megakaryocyte differentiation	GO:0045653	1.36E-02	4.28E-02	1	6
6,7	VDAC3|2.015475	purine nucleobase	GO:0006863	1.36E-02	4.28E-02	1	7
6,7	TOP2A|0.481675	apoptotic chromosome condensation	GO:0030263	1.36E-02	4.28E-02	1	7
5,6,7,8	PLEK|2.071125	positive regulation of integrin activation	GO:0033625	1.36E-02	4.28E-02	1	8
5,9,10	PRDX5|3.0143	NADPH oxidation	GO:0070995	1.36E-02	4.28E-02	1	10
4,6,7	SMC2|0.485025	meiotic chromosome condensation	GO:0010032	1.36E-02	4.28E-02	1	7
6	VTN|2.01125	smooth muscle cell-matrix adhesion	GO:0061302	1.36E-02	4.28E-02	1	6
8,10,11	STAT3|0.4995	regulation of ATP biosynthetic process	GO:2001169	1.36E-02	4.28E-02	1	11
7	TBCA|0.3515	post-chaperonin tubulin folding pathway	GO:0007023	1.36E-02	4.28E-02	1	7
6	ESD|2.155525	formaldehyde catabolic process	GO:0046294	1.36E-02	4.28E-02	1	6
5	GSN|2.4555	membrane raft	GO:0051665	1.36E-02	4.28E-02	1	5
5,6,7,8,9	ASPH|2.59965	regulation of inositol 1,4,5-trisphosphate-sensitive calcium-release channel activity	GO:0031585	1.36E-02	4.28E-02	1	9
6	HSPA8|0.3721	vesicle uncoating	GO:0072319	1.36E-02	4.28E-02	1	6
4,5,6	ZNF706|0.3974	negative regulation of stem cell maintenance	GO:1902455	1.36E-02	4.28E-02	1	6
4	GSN|2.4555;TFRC|0.403125;SNW1|0.473175	response to nutrient	GO:0007584	1.37E-02	4.28E-02	3	4
6,7	PSME3|0.401575;SNW1|0.473175;NDRG1|10.0073	signal transduction by p53 class mediator	GO:0072331	1.37E-02	4.30E-02	3	7
7,8	TTK|0.488375;PLK1|0.415475	negative regulation of metaphase/anaphase transition of cell cycle	GO:1902100	1.37E-02	4.30E-02	2	8
5	HSP90AB1|0.458775;GCH1|4.01735	nitric oxide biosynthetic process	GO:0006809	1.37E-02	4.30E-02	2	5
5	HSPA8|0.3721;ENO1|2.1939;ATP6V1B2|2.0575;GMPS|0.496875;PRDX5|3.0143;ALDOC|2.04725;RRM2|0.21675;STAT3|0.4995	nucleoside phosphate metabolic process	GO:0006753	1.37E-02	4.30E-02	8	5
4,5,6,8	ANXA5|3.637725;HIST1H3A|0.455625;CHAF1B|0.2723	regulation of defense response to virus	GO:0050688	1.39E-02	4.32E-02	3	8
7	DNMT1|0.172;ATG7|2.120975;SNW1|0.473175	regulation of chromatin modification	GO:1903308	1.41E-02	4.35E-02	3	7
5	ATG7|2.120975;MAPK14|0.483325;MYH10|5.579275	cardiac muscle tissue development	GO:0048738	1.41E-02	4.40E-02	3	5
5	NDE1|0.446425;PLK1|0.415475;CETN3|0.4171	microtubule organizing center organization	GO:0031023	1.41E-02	4.40E-02	3	5
4,5	TTK|0.488375;PLK1|0.415475	negative regulation of chromosome segregation	GO:0051985	1.41E-02	4.40E-02	2	5
3	ACTG1|2.1999;HSP90AB1|0.458775;HSPD1|2.88995;KRT1|0.4198;PSME3|0.401575;MAPK14|0.483325;VTN|2.01125;ACTB|2.1999	positive regulation of immune system process	GO:0002684	1.41E-02	4.40E-02	8	3
4	HSP90AB1|0.458775;LCP1|2.170475;ASPH|2.59965;NSF|2.017425;YWHAG|2.359475;GSN|2.4555;MAPK14|0.483325;RHOG|2.449775;SLC2A1|5.081875	regulation of cellular localization	GO:0060341	1.42E-02	4.41E-02	9	4
5,6,7	PABPC1|0.3285;ASPH|2.59965;PRDX5|3.0143;BAZ1B|0.460625;SQSTM1|6.007525;PCNA|0.404975;MAPK14|0.483325;RHOG|2.449775;TOP2A|0.481675;SNW1|0.473175;STAT3|0.4995	positive regulation of macromolecule biosynthetic process	GO:0010557	1.42E-02	4.41E-02	11	7

6,8	VIM|2.241075;YWHAG|2.359475;SRRT|0.39875;SNW1|0.473175;STAT3|0.4995;ARHGDIA|3.8215	regulation of neurogenesis	GO:0050767	1.43E-02	4.41E-02	6	8
4,6	HSP90AB1|0.458775;ASPH|2.59965;YWHAG|2.359475;MAPK14|0.483325	positive regulation of intracellular transport	GO:0032388	1.43E-02	4.45E-02	4	6
5	HSP90AB1|0.458775;RPL3|3.30745;MCM2|0.491625;KPNA2|0.186675;STAT3|0.4995;GCH1|4.01735;IFITM2|5.6831	response to cytokine	GO:0034097	1.44E-02	4.45E-02	7	5
8	ENO1|2.1939;ALDOC|2.04725;STAT3|0.4995	pyruvate metabolic	GO:0006090	1.45E-02	4.48E-02	3	8
7,8	DNMT1|0.172;ATG7|2.120975	negative regulation of chromatin modification	GO:1903309	1.45E-02	4.49E-02	2	8
7,8,9	HSPA8|0.3721;ENO1|2.1939;ASPH|2.59965;DNMT1|0.172;HIST1H4A|0.23835;SRRT|0.39875;CBX3|0.485775;GTF2E1|0.300475;PRDX5|3.0143;BAZ1B|0.460625;RRM2|0.21675;SQSTM1|6.007525;PCNA|0.404975;MAPK14|0.483325;HIST1H3A|0.455625;BTF3|0.2449;RHOG|2.449775;TOP2A|0.481675;SNW1|0.473175;STAT3|0.4995;TXNIP|2.0553;RBBP7|0.218475;H3F3A|0.20985;PLK1|0.415475;SOD2|2.175425;ZNF706|0.3974;CHAF1B|0.2723	regulation of nucleic acid-templated transcription	GO:1903506	1.46E-02	4.49E-02	27	9
4,5	HSPA8|0.3721;ACTG1|2.1999;HSP90AB1|0.458775;RPL3|3.30745;ASPH|2.59965;ATP6V1B2|2.0575;RPL4|2.063325;NSF|2.017425;KPNA2|0.186675;VDAC3|2.015475;YWHAG|2.359475;GSN|2.4555;SLC25A1|2.1121;ERO1L|2.370575;RPL27|2.15885;SQSTM1|6.007525;ATG7|2.120975;MAPK14|0.483325;TFRC|0.403125;STAT3|0.4995;SLC2A1|5.081875;TXNIP|2.0553;PLEK|2.071125;NDE1|0.446425;HBA1|2.13735;ACTB|2.1999;MYH10|5.579275;ALB|0.234	single-organism transport	GO:0044765	1.47E-02	4.50E-02	28	5
6	HSPA8|0.3721;ENO1|2.1939;ASPH|2.59965;DNMT1|0.172;HIST1H4A|0.23835;YWHAG|2.359475;SRRT|0.39875;CBX3|0.485775;GTF2E1|0.300475;PRDX5|3.0143;BAZ1B|0.460625;RRM2|0.21675;SQSTM1|6.007525;PCNA|0.404975;MAPK14|0.483325;HIST1H3A|0.455625;BTF3|0.2449;RHOG|2.449775;TOP2A|0.481675;SNW1|0.473175;STAT3|0.4995;TXNIP|2.0553;RBBP7|0.218475;H3F3A|0.20985;PLK1|0.415475;SOD2|2.175425;ZNF706|0.3974;CHAF1B|0.	transcription, DNA-templated	GO:0006351	1.47E-02	4.54E-02	28	6
5,7,8	YWHAG|2.359475;STAT3|0.4995	regulation of mitochondrial membrane	GO:0046902	1.49E-02	4.54E-02	2	8
7,8,9	TUBG1|0.4102;NDE1|0.446425	microtubule	GO:0046785	1.49E-02	4.59E-02	2	9
5	LCP1|2.170475;VIM|2.241075;KRT9|0.268525;MTHFD1L|6.667175;GSN|2.4555;ALDOC|2.04725;PCNA|0.404975;TXNIP|2.0553	epithelium development	GO:0060429	1.50E-02	4.59E-02	8	5
5	ASPH|2.59965;KRT1|0.4198;ATP6V1B2|2.0575;CKB|0.486625;ERO1L|2.370575;TFRC|0.403125;STAT3|0.4995;SOD2|2.175425	chemical homeostasis	GO:0048878	1.51E-02	4.60E-02	8	5
8,9,10,11	HSP90AB1|0.458775;TTK|0.488375;PLK1|0.415475	regulation of proteasomal ubiquitin-dependent protein catabolic process	GO:0032434	1.53E-02	4.63E-02	3	11
6	DNMT1|0.172;ATG7|2.120975;SNW1|0.473175	regulation of chromatin organization	GO:1902275	1.53E-02	4.68E-02	3	6
6,7,8	TOP2A|0.481675;IFITM2|5.68315	regulation of viral genome replication	GO:0045069	1.53E-02	4.68E-02	2	8
7,8	GCH1|4.01735;SOD2|2.175425	vasodilation	GO:0042311	1.53E-02	4.68E-02	2	8
6	HSPA8|0.3721;ENO1|2.1939;ASPH|2.59965;DNMT1|0.172;HIST1H4A|0.23835;SRRT|0.39875;CBX3|0.485775;GTF2E1|0.300475;PRDX5|3.0143;BAZ1B|0.460625;RRM2|0.21675;SQSTM1|6.007525;PCNA|0.404975;MAPK14|0.483325;HIST1H3A|0.455625;BTF3|0.2449;RHOG|2.449775;TOP2A|0.481675;SNW1|0.473175;STAT3|0.4995;TXNIP|2.0553;RBBP7|0.218475;H3F3A|0.20985;PLK1|0.415475;SOD2|2.175425;ZNF706|0.3974;CHAF1B|0.2723	regulation of RNA biosynthetic process	GO:2001141	1.54E-02	4.68E-02	27	6
5,6	HSPA8|0.3721;HSP90AB1|0.458775	regulation of cellular response to heat	GO:1900034	1.58E-02	4.70E-02	2	6
4	MTHFD1L|6.667175;ALDH1L2|2.148	one-carbon metabolic process	GO:0006730	1.58E-02	4.80E-02	2	4
7	HSPA8|0.3721;ENO1|2.1939;ATP6V1B2|2.0575;GMPS|0.496875;ALDOC|2.04725;STAT3|0.4995	purine ribonucleotide metabolic process	GO:0009150	1.59E-02	4.80E-02	6	7
7,8	RPL3|3.30745;RPL4|2.063325;RPL27|2.15885	translational termination	GO:0006415	1.60E-02	4.83E-02	3	8
6	ENO1|2.1939;ALDOC|2.04725	monosaccharide catabolic process	GO:0046365	1.62E-02	4.85E-02	2	6
7,8	HSPA8|0.3721;ENO1|2.1939;ASPH|2.59965;DNMT1|0.172;HIST1H4A|0.23835;YWHAG|2.359475;SRRT|0.39875;CBX3|0.485775;GTF2E1|0.300475;PRDX5|3.0143;BAZ1B|0.460625;RRM2|0.21675;SQSTM1|6.007525;PCNA|0.404975;MAPK14|0.483325;HIST1H3A|0.455625;BTF3|0.2449;RHOG|2.449775;TOP2A|0.481675;SNW1|0.473175;STAT3|0.4995;TXNIP|2.0553;RBBP7|0.218475;H3F3A|0.20985;PLK1|0.415475;SOD2|2.175425;ZNF706|0.3974;CHAF1B|0.	nucleic acid-templated transcription	GO:0097659	1.62E-02	4.85E-02	28	8
5,6,7	SNW1|0.473175	regulation of vitamin D receptor signaling	GO:0070562	1.62E-02	4.85E-02	1	7
9,10	GMPS|0.496875	GMP biosynthetic process	GO:0006177	1.62E-02	4.85E-02	1	10
5	ANXA5|3.637725	regulation of sperm	GO:1901317	1.62E-02	4.85E-02	1	5
8	MTHFD1L|6.667175	formate metabolic process	GO:0015942	1.62E-02	4.85E-02	1	8
5	ESD|2.155525	formaldehyde metabolic process	GO:0046292	1.62E-02	4.85E-02	1	5
7,8	VIM|2.241075	Bergmann glial cell differentiation	GO:0060020	1.62E-02	4.85E-02	1	8
7,8,9,11,12	DNMT1|0.172	negative regulation of histone H3-K9	GO:0051573	1.62E-02	4.85E-02	1	12

6,7,8	STAT3|0.4995	negative regulation of hydrogen peroxide metabolic process	GO:0010727	1.62E-02	4.85E-02	1	8
5,6,7	HSPA8|0.3721	negative regulation of extracellular matrix organization	GO:1903054	1.62E-02	4.85E-02	1	7
7	SOD2|2.175425	oxygen homeostasis	GO:0032364	1.62E-02	4.85E-02	1	7
5,6	SLC2A1|5.081875	xenobiotic transport	GO:0042908	1.62E-02	4.85E-02	1	6
7,8	GCH1|4.01735	tetrahydrofolate biosynthetic process	GO:0046654	1.62E-02	4.85E-02	1	8
5	ALDOC|2.04725	fructose 1,6-bisphosphate metabolic process	GO:0030388	1.62E-02	4.85E-02	1	5
7,8	GSN|2.4555	actin filament reorganization	GO:0090527	1.62E-02	4.85E-02	1	8
6,7	MAPK14|0.483325	regulation of interleukin-12 secretion	GO:2001182	1.62E-02	4.85E-02	1	7
6	PSME3|0.401575;MAPK14|0.483325;PLK1|0.415475	DNA damage checkpoint	GO:0000077	1.64E-02	4.85E-02	3	6
6	ATG7|2.120975;MAPK14|0.483325;SNW1|0.473175;MYH10|5.579275	striated muscle tissue development	GO:0014706	1.65E-02	4.89E-02	4	6
4	ACTG1|2.1999;HSP90AB1|0.458775;HSPD1|2.88995;KRT1|0.4198;PSME3|0.401575;MAPK14|0.483325;VTN|2.01125;ACTB|2.1999	regulation of immune response	GO:0050776	1.65E-02	4.92E-02	8	4
6	HSP90AB1|0.458775;PRDX5|3.0143;VTN|2.01125;TTK|0.488375;PLK1|0.415475	negative regulation of proteolysis	GO:0045861	1.66E-02	4.92E-02	5	6
5,8,9	PABPC1|0.3285;MAPK14|0.483325	regulation of mRNA stability	GO:0043488	1.66E-02	4.92E-02	2	9
4	YWHAG|2.359475;STAT3|0.4995	regulation of membrane permeability	GO:0090559	1.66E-02	4.92E-02	2	4
7,8	PSME3|0.401575;PLK1|0.415475	positive regulation of ubiquitin-protein ligase activity involved in regulation of mitotic cell cycle transition	GO:0051437	1.66E-02	4.92E-02	2	8
6	HSPD1|2.88995;STAT3|0.4995;TXNIP|2.0553	response to estrogen	GO:0043627	1.69E-02	4.92E-02	3	6
6,7	HSP90AB1|0.458775;LCP1|2.170475;ASPH|2.59965;MAPK14|0.483325	regulation of intracellular protein transport	GO:0033157	1.71E-02	4.99E-02	4	7
4	ANXA5|3.637725;GSN|2.4555;MAPK14|0.483325;STAT3|0.4995;VTN|2.01125;ARHGDIA|3.8215	regulation of cellular component movement	GO:0051270	1.75E-02	5.06E-02	6	4
6,7	HSPA8|0.3721;ENO1|2.1939;ATP6V1B2|2.0575;GMPS|0.496875;ALDOC|2.04725;STAT3|0.4995	ribonucleotide metabolic process	GO:0009259	1.75E-02	5.14E-02	6	7
5	ACTG1|2.1999;LCP1|2.170475;RPL3|3.30745;VDAC3|2.015475;GSN|2.4555;MYH10|5.579275;PLK1|0.415475	organelle assembly	GO:0070925	1.75E-02	5.14E-02	7	5
4	HSP90AB1|0.458775;SLC2A1|5.081875	response to osmotic stress	GO:0006970	1.75E-02	5.14E-02	2	4
5,6,7,8	ACTG1|2.1999;MYH10|5.579275	myofibril assembly	GO:0030239	1.75E-02	5.14E-02	2	8
4,5	ISYNA1|0.49065;PLEK|2.071125	inositol phosphate metabolic process	GO:0043647	1.75E-02	5.14E-02	2	5
4,7,8	PABPC1|0.3285;MAPK14|0.483325	regulation of RNA	GO:0043487	1.79E-02	5.14E-02	2	8
5	TFRC|0.403125;NCDN|0.4114	bone remodeling	GO:0046849	1.79E-02	5.25E-02	2	5
6,7,8	TTK|0.488375;PLK1|0.415475	negative regulation of mitotic nuclear division	GO:0045839	1.79E-02	5.25E-02	2	8
4	VIM|2.241075;HIST1H4A|0.23835;YWHAG|2.359475;SRRT|0.39875;MAPK14|0.483325;SNW1|0.473175;STAT3|0.4995;ARHGDIA|3.8215;SOS2|11.48525;ZNF706|0.3974	regulation of cell differentiation	GO:0045595	1.80E-02	5.25E-02	10	4
4,6,7	ACTG1|2.1999;HSP90AB1|0.458775;ACTB|2.1999	immune response-regulating cell surface receptor signaling pathway involved in	GO:0002433	1.82E-02	5.26E-02	3	7
5,7,9	ACTG1|2.1999;HSP90AB1|0.458775;ACTB|2.1999	Fc-gamma receptor signaling pathway involved in phagocytosis	GO:0038096	1.82E-02	5.32E-02	3	9
5,6	ACTG1|2.1999;HSP90AB1|0.458775;GSN|2.4555;ACTB|2.1999	phagocytosis	GO:0006909	1.83E-02	5.32E-02	4	6
7	ENO1|2.1939;ALDOC|2.04725;STAT3|0.4995	nucleoside diphosphate phosphorylation	GO:0006165	1.85E-02	5.32E-02	3	7
8	ACTG1|2.1999;HSP90AB1|0.458775;ACTB|2.1999	Fc-gamma receptor signaling pathway	GO:0038094	1.85E-02	5.37E-02	3	8
5,6	HSPA8|0.3721;PABPC1|0.3285;ASPH|2.59965;BAZ1B|0.460625;SQSTM1|6.007525;PCNA|0.404975;MAPK14|0.483325;RHOG|2.449775;TOP2A|0.481675;SNW1|0.473175;STAT3|0.4995	positive regulation of nucleobase-containing compound metabolic process	GO:0045935	1.85E-02	5.37E-02	11	6
6,7,8	PSME3|0.401575;TTK|0.488375;PLK1|0.415475	negative regulation of mitotic cell cycle phase transition	GO:1901991	1.87E-02	5.38E-02	3	8
5	ATG7|2.120975;MAPK14|0.483325;SNW1|0.473175;MYH10|5.579275	muscle tissue development	GO:0060537	1.88E-02	5.39E-02	4	5
7	NDRG1|10.0073	positive regulation of spindle checkpoint	GO:0090232	1.89E-02	5.39E-02	1	7
5	MTHFD1L|6.667175	embryonic neurocranium morphogenesis	GO:0048702	1.89E-02	5.39E-02	1	5
7,9,11,12	SRRT|0.39875	primary miRNA	GO:0031053	1.89E-02	5.39E-02	1	12
6	PABPC1|0.3285	negative regulation of RNA catabolic process	GO:1902369	1.89E-02	5.39E-02	1	6
4	AKR1B1|2.235325	response to water deprivation	GO:0009414	1.89E-02	5.39E-02	1	4
6,8,9	SOS2|11.48525	regulation of pro-B cell differentiation	GO:2000973	1.89E-02	5.39E-02	1	9
6	MYH10|5.579275	substrate-dependent cell migration, cell extension	GO:0006930	1.89E-02	5.39E-02	1	6

7,8,9	PRDX5|3.0143	negative regulation of transcription from RNA polymerase III promoter	GO:0016480	1.89E-02	5.39E-02	1	9
6	STAT3|0.4995	negative regulation of neuron migration	GO:2001223	1.89E-02	5.39E-02	1	6
6,7,8	PLEK|2.071125	negative regulation of calcium-mediated	GO:0050849	1.89E-02	5.39E-02	1	8
5,6,7,8	LCP1|2.170475	positive regulation of podosome assembly	GO:0071803	1.89E-02	5.39E-02	1	8
7,8,9,10	ASPH|2.59965	positive regulation of ryanodine-sensitive calcium-release channel activity	GO:0060316	1.89E-02	5.39E-02	1	10
5,6	ALB|0.23405	maintenance of organelle location	GO:0051657	1.89E-02	5.39E-02	1	6
4	ATG7|2.120975	positive regulation of mucus secretion	GO:0070257	1.89E-02	5.39E-02	1	4
5	TOP2A|0.481675	embryonic cleavage	GO:0040016	1.89E-02	5.39E-02	1	5
5,6	VDAC3|2.015475	nucleobase transport	GO:0015851	1.89E-02	5.39E-02	1	6
5,6	HSP90AB1|0.458775;SNW1|0.473175;STAT3|0.4995;TXNIP|2.0553	intracellular receptor signaling pathway	GO:0030522	1.91E-02	5.39E-02	4	6
6,7	GSN|2.4555;IFITM2|5.68315	viral entry into host cell	GO:0046718	1.93E-02	5.43E-02	2	7
4	HSPD1|2.88995;LIG1|0.440425	somatic diversification of immune receptors via germline recombination within a single locus	GO:0002562	1.93E-02	5.48E-02	2	4
5	HSP90AB1|0.458775;GCH1|4.01735	nitric oxide metabolic process	GO:0046209	1.93E-02	5.48E-02	2	5
7,8	HSPD1|2.88995;LIG1|0.440425	somatic cell DNA recombination	GO:0016444	1.93E-02	5.48E-02	2	8
4,5,6,7	SMC2|0.485025;TOP2A|0.481675;PLK1|0.415475	meiotic nuclear division	GO:0007126	1.94E-02	5.48E-02	3	7
4,5	ANXA5|3.637725;ATG7|2.120975;HIST1H3A|0.455625;IFITM2|5.68315;CHAF1B|0.2723	defense response to other organism	GO:0098542	1.95E-02	5.50E-02	5	5
5	ACTG1|2.1999;HSP90AB1|0.458775;HSPD1|2.88995;PSME3|0.401575;MAPK14|0.483325;ACTB|2.1999	immune response-regulating signaling pathway	GO:0002764	1.96E-02	5.50E-02	6	5
3,4,5	ASPH|2.59965;ERO1L|2.370575;MAPK14|0.483325;TFRC|0.403125	regulation of homeostatic process	GO:0032844	1.96E-02	5.53E-02	4	5
6,7	HSP90AB1|0.458775;HSPD1|2.88995;SOD2|2.175425	regulation of neuron apoptotic process	GO:0043523	1.97E-02	5.53E-02	3	7
4,5	ASPH|2.59965;ATP6V1B2|2.0575;CKB|0.486625;LIG1|0.440425;ERO1L|2.370575;PCNA|0.404975;TFRC|0.403125	cellular homeostasis	GO:0019725	1.98E-02	5.56E-02	7	5
6	HSPA8|0.3721;ENO1|2.1939;ATP6V1B2|2.0575;GMPS|0.496875;ALDOC|2.04725;STAT3|0.4995	purine nucleotide metabolic process	GO:0006163	1.98E-02	5.57E-02	6	6
3	HSPD1|2.88995;SRRT|0.39875;PRDX5|3.0143;ATG7|2.120975;MAPK14|0.483325;TFRC|0.403125;SNW1|0.473175;PLEK|2.071125;ARHGDIA|3.8215	positive regulation of multicellular organismal process	GO:0051240	2.02E-02	5.57E-02	9	3
5,7,8,9	DNMT1|0.172;SNW1|0.473175	positive regulation of histone modification	GO:0031058	2.03E-02	5.69E-02	2	9
6,8	ACTG1|2.1999;HSP90AB1|0.458775;ACTB|2.1999	Fc receptor mediated stimulatory signaling pathway	GO:0002431	2.04E-02	5.69E-02	3	8
5,6	NSF|2.017425;RHOG|2.449775;NDRG1|10.0073;MYH10|5.579275	plasma membrane organization	GO:0007009	2.05E-02	5.73E-02	4	6
5	HSP90AB1|0.458775;LCP1|2.170475;ASPH|2.59965;YWHAG|2.359475;MAPK14|0.483325	regulation of intracellular transport	GO:0032386	2.07E-02	5.73E-02	5	5
5,6,7	HSPA8|0.3721;PABPC1|0.3285;ASPH|2.59965;BAZ1B|0.460625;SQSTM1|6.007525;MAPK14|0.483325;RHOG|2.449775;TOP2A|0.481675;SNW1|0.473175;STAT3|0.4995	positive regulation of RNA metabolic process	GO:0051254	2.07E-02	5.79E-02	10	7
5,6	YWHAG|2.359475;PSME3|0.401575;TTK|0.488375;PLK1|0.415475	negative regulation of transferase activity	GO:0051348	2.08E-02	5.79E-02	4	6
7,8	ENO1|2.1939;DNMT1|0.172;RRM2|0.21675;SQSTM1|6.007525;PCNA|0.404975;MAPK14|0.483325;TOP2A|0.481675;SNW1|0.473175;STAT3|0.4995;TXNIP|2.0553;RBBP7|0.218475;PLK1|0.415475;SOD2|2.175425	regulation of transcription from RNA polymerase II promoter	GO:0006357	2.09E-02	5.81E-02	13	8
5	HSPA8|0.3721;ENO1|2.1939;ATP6V1B2|2.0575;GMPS|0.496875;ALDOC|2.04725;STAT3|0.4995	ribose phosphate metabolic process	GO:0019693	2.09E-02	5.84E-02	6	5
4	VIM|2.241075;KRT1|0.4198;HIST1H4A|0.23835;YWHAG|2.359475;SRRT|0.39875;MAPK14|0.483325;SNW1|0.473175;STAT3|0.4995;ARHGDIA|3.8215;SOS2|11.48525	regulation of multicellular organismal development	GO:2000026	2.11E-02	5.84E-02	10	4
6	PSME3|0.401575;TTK|0.488375;PLK1|0.415475	negative regulation of cell cycle phase transition	GO:1901988	2.15E-02	5.87E-02	3	6
5	ATG7|2.120975	cellular response to hyperoxia	GO:0071455	2.16E-02	5.89E-02	1	5
6	MAPK14|0.483325	interleukin-12 secretion	GO:0072610	2.16E-02	5.89E-02	1	6
7,8	TBCA|0.3515	tubulin complex assembly	GO:0007021	2.16E-02	5.89E-02	1	8
6	GSN|2.4555	establishment of T cell polarity	GO:0001768	2.16E-02	5.89E-02	1	6
8,9,11	ATG7|2.120975	pyramidal neuron development	GO:0021860	2.16E-02	5.89E-02	1	11
6,7,8,9	TOP2A|0.481675	regulation of single stranded viral RNA replication via double stranded DNA	GO:0045091	2.16E-02	5.89E-02	1	9
6	MAPK14|0.483325	stress-induced premature senescence	GO:0090400	2.16E-02	5.89E-02	1	6

5,7	LIG1|0.440425	removal of RNA primer involved in mitotic DNA replication	GO:1903469	2.16E-02	5.89E-02	1	7
6	ATG7|2.120975	cellular response to increased oxygen levels	GO:0036295	2.16E-02	5.89E-02	1	6
5,6,7	VAT1|2.702925	regulation of mitochondrial fusion	GO:0010635	2.16E-02	5.89E-02	1	7
6,7	MYH10|5.579275	plasma membrane repair	GO:0001778	2.16E-02	5.89E-02	1	7
6,7	MAPK14|0.483325	positive regulation of brown fat cell	GO:0090336	2.16E-02	5.89E-02	1	7
7,8	PLK1|0.415475	activation of anaphase-promoting complex	GO:0051488	2.16E-02	5.89E-02	1	8
6	NSF|2.017425	Golgi vesicle docking	GO:0048211	2.16E-02	5.89E-02	1	6
7,8	BAZ1B|0.460625	chromatin-mediated maintenance of transcription	GO:0048096	2.16E-02	5.89E-02	1	8
5	AKR1B1|2.235325	doxorubicin metabolic process	GO:0044598	2.16E-02	5.89E-02	1	5
5,7	AKR1B1|2.235325	daunorubicin metabolic process	GO:0044597	2.16E-02	5.89E-02	1	7
5,8	GSN|2.4555	striated muscle atrophy	GO:0014891	2.16E-02	5.89E-02	1	8
4	SNW1|0.473175	regulation of response to alcohol	GO:1901419	2.16E-02	5.89E-02	1	4
4,7	LIG1|0.440425	Okazaki fragment processing involved in mitotic DNA replication	GO:1903461	2.16E-02	5.89E-02	1	7
7,8	G3BP1|0.3904;SQSTM1|6.007525;MAPK14|0.483325;RHOG|2.449775;ARHGDIA|3.8215;SOS2|11.48525	Ras protein signal transduction	GO:0007265	2.16E-02	5.89E-02	6	8
5,6,7	HSP90AB1|0.458775;MAPK14|0.483325	positive regulation of reactive oxygen species metabolic process	GO:2000379	2.17E-02	5.90E-02	2	7
5,6,7,8	GSN|2.4555;IFITM2|5.68315	negative regulation of viral life cycle	GO:1903901	2.17E-02	5.90E-02	2	8
7,8	DNMT1|0.172;SNW1|0.473175	positive regulation of chromatin modification	GO:1903310	2.17E-02	5.90E-02	2	8
5,7,9	VIM|2.241075;STAT3|0.4995;ARHGDIA|3.8215	negative regulation of neurogenesis	GO:0050768	2.17E-02	5.90E-02	3	9
4	ENO1|2.1939;TFRC|0.403125;RBBP7|0.218475;H3F3A|0.20985	regulation of cell growth	GO:0001558	2.19E-02	5.90E-02	4	4
4,5	HSPA8|0.3721;ENO1|2.1939;ATP6V1B2|2.0575;GMPS|0.496875;PRDX5|3.0143;ALDOC|2.04725;RRM2|0.21675;STAT3|0.4995	nucleobase-containing small molecule metabolic process	GO:0055086	2.20E-02	5.93E-02	8	5
3	GSN|2.4555;IFITM2|5.68315	movement in environment of other organism involved in symbiotic	GO:0052192	2.22E-02	5.97E-02	2	3
5,6	GSN|2.4555;IFITM2|5.68315	entry into host	GO:0044409	2.22E-02	5.97E-02	2	6
6	GSN|2.4555;IFITM2|5.68315	entry into host cell	GO:0030260	2.22E-02	5.97E-02	2	6
5	GSN|2.4555;IFITM2|5.68315	entry into cell of other organism involved in symbiotic interaction	GO:0051806	2.22E-02	5.97E-02	2	5
4	GSN|2.4555;IFITM2|5.68315	entry into other organism involved in symbiotic interaction	GO:0051828	2.22E-02	5.97E-02	2	4
5,7,8	LIG1|0.440425;PCNA|0.404975	mismatch repair	GO:0006298	2.22E-02	5.97E-02	2	8
4,5	GSN|2.4555;IFITM2|5.68315	movement in host environment	GO:0052126	2.22E-02	5.97E-02	2	5
5,7,8	PABPC1|0.3285;SRRT|0.39875	gene silencing by RNA	GO:0031047	2.22E-02	5.97E-02	2	8
4	KRT1|0.4198;VDAC3|2.015475;GSN|2.4555;MAPK14|0.483325;VAT1|2.702925;ARHGDIA|3.8215;MYH10|5.57927	regulation of anatomical structure morphogenesis	GO:0022603	2.26E-02	5.97E-02	7	4
5,6	HSP90AB1|0.458775;PABPC1|0.3285;ASPH|2.59965;BAZ1B|0.460625;SQSTM1|6.007525;PCNA|0.404975;MAPK14|0.483325;RHOG|2.449775;TOP2A|0.481675;SNW1|0.473175;STAT3|0.4995	positive regulation of cellular biosynthetic process	GO:0031328	2.28E-02	6.07E-02	11	6
5,6,7,8	G3BP1|0.3904;PSME3|0.401575;MAPK14|0.483325	negative regulation of Wnt signaling pathway	GO:0030178	2.30E-02	6.13E-02	3	8
4,5,6,7	GSN|2.4555;IFITM2|5.68315	negative regulation of viral process	GO:0048525	2.32E-02	6.17E-02	2	7
6,7,8,9	TTK|0.488375;PLK1|0.415475	regulation of mitotic metaphase/anaphase transition	GO:0030071	2.32E-02	6.20E-02	2	9
6,8,9	PLEK|2.071125;ALB|0.23405	platelet degranulation	GO:0002576	2.32E-02	6.20E-02	2	9
5	ACTG1|2.1999;ATG7|2.120975;MAPK14|0.483325;MYH10|5.579275	muscle cell differentiation	GO:0042692	2.34E-02	6.20E-02	4	5
5	ATP6V1B2|2.0575;DNMT1|0.172;AKR1B1|2.235325;LAMTOR2|7.841025;STAT3|0.4995	cellular response to organonitrogen compound	GO:0071417	2.34E-02	6.24E-02	5	5
3	ENO1|2.1939;SQSTM1|6.007525;RBBP7|0.218475	negative regulation of growth	GO:0045926	2.36E-02	6.24E-02	3	3
8,9	PSME3|0.401575;PLK1|0.415475	positive regulation of protein ubiquitination involved in ubiquitin-dependent protein catabolic process	GO:2000060	2.37E-02	6.28E-02	2	9
7	TTK|0.488375;PLK1|0.415475	regulation of metaphase/anaphase transition of cell cycle	GO:1902099	2.37E-02	6.30E-02	2	7
2	ACTG1|2.1999;HSP90AB1|0.458775;LCP1|2.170475;HSPD1|2.88995;GSN|2.4555;PLEK|2.071125;VTN|2.01125;ARHGDIA|3.8215;ACTB|2.1999;MYH10|5.579275;SOS2|11.4	biological adhesion	GO:0022610	2.39E-02	6.30E-02	11	2

7,9	GSN|2.4555;PLEK|2.071125	regulation of actin filament depolymerization	GO:0030834	2.42E-02	6.30E-02	2	9
5	TTK|0.488375;PLK1|0.415475	metaphase/anaphase transition of mitotic cell cycle	GO:0007091	2.42E-02	6.30E-02	2	5
6	SOD2|2.175425	gas homeostasis	GO:0033483	2.43E-02	6.30E-02	1	6
5	GSN|2.4555	establishment of lymphocyte polarity	GO:0001767	2.43E-02	6.30E-02	1	5
4,5,6	SLC2A1|5.081875	disaccharide metabolic process	GO:0005984	2.43E-02	6.30E-02	1	6
6,8,9,10,11	HSP90AB1|0.458775	positive regulation of protein import into nucleus, translocation	GO:0033160	2.43E-02	6.30E-02	1	11
7	STAT3|0.4995	radial glial cell differentiation	GO:0060019	2.43E-02	6.30E-02	1	7
5,7	HSP90AB1|0.458775	virion attachment to host cell	GO:0019062	2.43E-02	6.30E-02	1	7
8,10,11	YARS|2.288275	isoleucyl-tRNA aminoacylation	GO:0006428	2.43E-02	6.30E-02	1	11
8,10	ASL|2.252975	arginine catabolic process	GO:0006527	2.43E-02	6.30E-02	1	10
7,8,10	ATG7|2.120975	pyramidal neuron differentiation	GO:0021859	2.43E-02	6.30E-02	1	10
5,6	KRT1|0.4198	complement activation, lectin pathway	GO:0001867	2.43E-02	6.30E-02	1	6
6,7	NDRG1|10.0073	positive regulation of cell cycle checkpoint	GO:1901978	2.43E-02	6.30E-02	1	7
4	AKR1B1|2.235325	polyketide metabolic process	GO:0030638	2.43E-02	6.30E-02	1	4
8,10,11	SARS|2.2366	seryl-tRNA	GO:0006434	2.43E-02	6.30E-02	1	11
7,8	STAT3|0.4995	interleukin-6-mediated signaling pathway	GO:0070102	2.43E-02	6.30E-02	1	8
4,6	HSP90AB1|0.458775	adhesion of symbiont to host cell	GO:0044650	2.43E-02	6.30E-02	1	6
6	AKR1B1|2.235325	aminoglycoside antibiotic metabolic process	GO:0030647	2.43E-02	6.30E-02	1	6
5,6	ISYNA1|0.49065	inositol biosynthetic	GO:0006021	2.43E-02	6.30E-02	1	6
5,6,7	PTP4A3|0.3433	regulation of vascular endothelial growth factor signaling pathway	GO:1900746	2.43E-02	6.30E-02	1	7
5,8	PTP4A3|0.3433	positive regulation of vascular permeability	GO:0043117	2.43E-02	6.30E-02	1	8
6,7	DNMT1|0.172	maintenance of DNA methylation	GO:0010216	2.43E-02	6.30E-02	1	7
6,8	MAPK14|0.483325	regulation of brown fat cell differentiation	GO:0090335	2.43E-02	6.30E-02	1	8
6,7	ERO1L|2.370575	protein maturation by protein folding	GO:0022417	2.43E-02	6.30E-02	1	7
4	PSME3|0.401575;TOP2A|0.481675;TTK|0.488375;CDC123|0.29925;PLK1|0.415475	negative regulation of cell cycle	GO:0045786	2.44E-02	6.30E-02	5	4
6,7	PSME3|0.401575;PLK1|0.415475	positive regulation of ubiquitin-protein transferase activity	GO:0051443	2.47E-02	6.34E-02	2	7
6	TTK|0.488375;PLK1|0.415475	metaphase/anaphase transition of cell cycle	GO:0044784	2.47E-02	6.40E-02	2	6
8,9	ENO1|2.1939;ALDOC|2.04725	NAD metabolic process	GO:0019674	2.52E-02	6.40E-02	2	9
6	MTHFD1L|6.667175;SLC25A1|2.1121;GCH1|4.01735	coenzyme biosynthetic process	GO:0009108	2.55E-02	6.53E-02	3	6
5,7,8,9,10	HSP90AB1|0.458775;MAPK14|0.483325	positive regulation of protein import into	GO:0042307	2.57E-02	6.60E-02	2	10
7,8,9	TTK|0.488375;PLK1|0.415475	regulation of mitotic sister chromatid separation	GO:0010965	2.57E-02	6.64E-02	2	9
6	HSP90AB1|0.458775;RPL3|3.30745;MCM2|0.491625;KPNA2|0.186675;STAT3|0.4995;IFITM2|5.68315	cellular response to cytokine stimulus	GO:0071345	2.58E-02	6.64E-02	6	6
6	HSP90AB1|0.458775;HSPD1|2.88995;SOD2|2.175425	neuron apoptotic process	GO:0051402	2.58E-02	6.64E-02	3	6
6,7,8,9	TTK|0.488375;PLK1|0.415475	regulation of mitotic sister chromatid segregation	GO:0033047	2.63E-02	6.64E-02	2	9
5,6,7	TTK|0.488375;PLK1|0.415475	regulation of sister chromatid segregation	GO:0033045	2.63E-02	6.75E-02	2	7
5,6,7	YWHAG|2.359475;GSN|2.4555;PSME3|0.401575;SOD2|2.175425	regulation of apoptotic signaling pathway	GO:2001233	2.63E-02	6.75E-02	4	7
5	ENO1|2.1939;ALDOC|2.04725;STAT3|0.4995	single-organism carbohydrate catabolic process	GO:0044724	2.67E-02	6.76E-02	3	5
6,7,8	TTK|0.488375;PLK1|0.415475	mitotic sister chromatid separation	GO:0051306	2.68E-02	6.79E-02	2	8
8,10,11	HSPD1|2.88995;MAPK14|0.483325	MyD88-dependent toll-like receptor signaling	GO:0002755	2.68E-02	6.79E-02	2	11
7	PLP1|3.936	long-chain fatty acid biosynthetic process	GO:0042759	2.69E-02	6.79E-02	1	7
8,9	GMPS|0.496875	GMP metabolic process	GO:0046037	2.69E-02	6.79E-02	1	9
4,6,8,9	ATG7|2.120975	regulation of mucus secretion	GO:0070255	2.69E-02	6.79E-02	1	9
5	MTHFD1L|6.667175	embryonic viscerocranium morphogenesis	GO:0048703	2.69E-02	6.79E-02	1	5
7	GCH1|4.01735	tetrahydrobiopterin biosynthetic process	GO:0006729	2.69E-02	6.79E-02	1	7
7,10,11,12	ATG7|2.120975	regulation of histone H4 acetylation	GO:0090239	2.69E-02	6.79E-02	1	12

5,6,7	TOP2A|0.481675	single stranded viral RNA replication via double stranded DNA	GO:0039692	2.69E-02	6.79E-02	1	7
5,6	CKB|0.486625	creatine metabolic process	GO:0006600	2.69E-02	6.79E-02	1	6
5	HSPD1|2.88995	positive regulation of response to tumor cell	GO:0002836	2.69E-02	6.79E-02	1	5
8,10,11	NARS|2.162	asparaginyl-tRNA aminoacylation	GO:0006421	2.69E-02	6.79E-02	1	11
5	HSPD1|2.88995	regulation of response to tumor cell	GO:0002834	2.69E-02	6.79E-02	1	5
5,6	HSPD1|2.88995	regulation of immune response to tumor cell	GO:0002837	2.69E-02	6.79E-02	1	6
5,6,7	HSPD1|2.88995	positive regulation of immune response to tumor cell	GO:0002839	2.69E-02	6.79E-02	1	7
5	PTP4A3|0.3433	regulation of cellular response to vascular endothelial growth factor stimulus	GO:1902547	2.69E-02	6.79E-02	1	5
5,6,7,8	PLEK|2.071125	regulation of integrin activation	GO:0033623	2.69E-02	6.79E-02	1	8
4	NSF|2.017425	positive regulation of receptor recycling	GO:0001921	2.69E-02	6.79E-02	1	4
5,6	HSP90AB1|0.458775;GCH1|4.01735;IFITM2|5.68315	response to interferon-gamma	GO:0034341	2.70E-02	6.79E-02	3	6
4	MAPK14|0.483325;TXNIP|2.0553;ARHGDIA|3.8215	response to mechanical stimulus	GO:0009612	2.70E-02	6.79E-02	3	4
5	VIM|2.241075;YWHAG|2.359475;SRRT|0.39875;SNW1|0.473175;STAT3|0.4995;ARHGDIA|3.8215	regulation of nervous system development	GO:0051960	2.71E-02	6.79E-02	6	5
6,7	HSPA8|0.3721;RPL3|3.30745;PABPC1|0.3285;RPL4|2.063325;RPL27|2.15885;SNW1|0.473175	mRNA metabolic process	GO:0016071	2.72E-02	6.82E-02	6	7
9,10	ATP6V1B2|2.0575;TFRC|0.403125	iron ion transport	GO:0006826	2.73E-02	6.84E-02	2	10
5	PRDX5|3.0143;GCH1|4.01735	regulation of oxidoreductase activity	GO:0051341	2.73E-02	6.84E-02	2	5
5	GSN|2.4555;SNW1|0.473175	response to vitamin	GO:0033273	2.73E-02	6.84E-02	2	5
5,6	PSME3|0.401575;PLK1|0.415475	positive regulation of ligase activity	GO:0051351	2.73E-02	6.84E-02	2	6
5,6	YWHAG|2.359475;STAT3|0.4995	mitochondrial membrane organization	GO:0007006	2.73E-02	6.84E-02	2	6
5,6,7,8	TTK|0.488375;PLK1|0.415475	negative regulation of nuclear division	GO:0051784	2.73E-02	6.84E-02	2	8
7	ENO1|2.1939;ALDOC|2.04725;STAT3|0.4995	nucleotide	GO:0046939	2.75E-02	6.84E-02	3	7
4	ATG7|2.120975;MYH10|5.579275	cerebellum development	GO:0021549	2.79E-02	6.88E-02	2	4
4,5,7,8	HSP90AB1|0.458775;ASPH|2.59965;MAPK14|0.483325	positive regulation of intracellular protein transport	GO:0090316	2.81E-02	6.96E-02	3	8
7	YWHAG|2.359475;GTF2E1|0.300475;PRDX5|3.0143;SNW1|0.473175	DNA-templated transcription, initiation	GO:0006352	2.82E-02	7.02E-02	4	7
4	HSPA8|0.3721;HSP90AB1|0.458775;ENO1|2.1939;ATP6V1B2|2.0575;GMPS|0.496875;CKB|0.486625;YWHAG|2.359475;SLC25A1|2.1121;PRDX5|3.0143;BAZ1B|0.460625;ISYNA1|0.49065;ALDOC|2.04725;RRM2|0.21675;LAMTOR2|7.841025;SQSTM1|6.007525;MAPK14|0.483325;STAT3|0.4995;PLEK|2.071125;VTN|2.01125;TTK|0.488375;PLK1|0.415475;GCH1|4.01735;PTP4A3|0.3433	phosphorus metabolic process	GO:0006793	2.83E-02	7.02E-02	23	4
8	MAPK14|0.483325;TFRC|0.403125	osteoclast differentiation	GO:0030316	2.84E-02	7.04E-02	2	8
8,9,10	PSME3|0.401575;PLK1|0.415475	regulation of protein ubiquitination involved in ubiquitin-dependent protein catabolic process	GO:2000058	2.84E-02	7.06E-02	2	10
6	ATG7|2.120975;MYH10|5.579275	cardiac muscle cell differentiation	GO:0055007	2.84E-02	7.06E-02	2	6
4	ENO1|2.1939;AKR1B1|2.235325;SLC25A1|2.1121;ISYNA1|0.49065;ALDOC|2.04725;MAPK14|0.483325;STAT3|0.4995;SLC2A1|5.081875;PLEK|2.071125	carbohydrate metabolic process	GO:0005975	2.85E-02	7.06E-02	9	4
4	YWHAG|2.359475;ANXA5|3.637725;PRDX5|3.0143;PSME3|0.401575;TXNIP|2.0553;VTN|2.01125;TTK|0.488375;PLK1|0.415475	negative regulation of molecular function	GO:0044092	2.86E-02	7.06E-02	8	4
4	ACTG1|2.1999;ATG7|2.120975;MAPK14|0.483325;SNW1|0.473175;MYH10|5.579275	muscle structure development	GO:0061061	2.88E-02	7.08E-02	5	4
6,7	ASPH|2.59965;ATP6V1B2|2.0575;CKB|0.486625;ERO1L|2.370575;TFRC|0.403125	cellular ion homeostasis	GO:0006873	2.88E-02	7.12E-02	5	7
5,7,8	LIG1|0.440425;PCNA|0.404975	base-excision repair	GO:0006284	2.90E-02	7.12E-02	2	8
4	ASPH|2.59965;ERO1L|2.370575	negative regulation of sequestering of calcium	GO:0051283	2.90E-02	7.13E-02	2	4
3	HSPD1|2.88995;LIG1|0.440425	somatic diversification of immune receptors	GO:0002200	2.90E-02	7.13E-02	2	3
5,7,8	ASPH|2.59965;ERO1L|2.370575	release of sequestered calcium ion into cytosol	GO:0051209	2.90E-02	7.13E-02	2	8
5	AKR1B1|2.235325;PRDX5|3.0143;SOD2|2.175425	cellular response to oxidative stress	GO:0034599	2.90E-02	7.13E-02	3	5
7,8	HSP90AB1|0.458775;LAMTOR2|7.841025;SQSTM1|6.007525;MAPK14|0.483325;VTN|2.01125;TTK|0.488375;PLK1|0.415475	positive regulation of protein phosphorylation	GO:0001934	2.93E-02	7.13E-02	7	8
4	VIM|2.241075;STAT3|0.4995;ARHGDIA|3.8215	negative regulation of nervous system development	GO:0051961	2.93E-02	7.13E-02	3	4

5,6	HSP90AB1|0.458775;HSPD1|2.88995;PSME3|0.401575;MAPK14|0.483325	regulation of innate immune response	GO:0045088	2.95E-02	7.13E-02	4	6
4,5	AKR1B1|2.235325;MAPK14|0.483325;RHOG|2.449775;STAT3|0.4995;TTK|0.488375;CDC123|0.29925	positive regulation of cell proliferation	GO:0008284	2.95E-02	7.13E-02	6	5
4	ASPH|2.59965;ERO1L|2.370575	regulation of sequestering of calcium ion	GO:0051282	2.95E-02	7.13E-02	2	4
5	TOP2A|0.481675;SNW1|0.473175	positive regulation of viral life cycle	GO:1903902	2.95E-02	7.13E-02	2	5
5	STAT3|0.4995	hydrogen peroxide biosynthetic process	GO:0050665	2.96E-02	7.13E-02	1	5
6	GCH1|4.01735	dopamine biosynthetic process	GO:0042416	2.96E-02	7.13E-02	1	6
8	SOS2|11.48525	pro-B cell differentiation	GO:0002328	2.96E-02	7.13E-02	1	8
5	HIST1H4A|0.23835	negative regulation of hematopoietic progenitor cell differentiation	GO:1901533	2.96E-02	7.13E-02	1	5
5,6,7	PCNA|0.404975	positive regulation of nuclease activity	GO:0032075	2.96E-02	7.13E-02	1	7
6	HSP90AB1|0.458775	positive regulation of cell size	GO:0045793	2.96E-02	7.13E-02	1	6
5	MTHFD1L|6.667175	tetrahydrofolate interconversion	GO:0035999	2.96E-02	7.13E-02	1	5
5	HSPD1|2.88995	isotype switching to IgG isotypes	GO:0048291	2.96E-02	7.13E-02	1	5
4,7	GSN|2.4555	muscle atrophy	GO:0014889	2.96E-02	7.13E-02	1	7
8,9	CKB|0.486625	cellular monovalent inorganic anion	GO:0030320	2.96E-02	7.13E-02	1	9
6,7,8,9	STAT3|0.4995	negative regulation of glycolytic process	GO:0045820	2.96E-02	7.13E-02	1	9
6	ALDH1L2|2.148	coenzyme catabolic process	GO:0009109	2.96E-02	7.13E-02	1	6
6,8	STAT3|0.4995	regulation of satellite cell proliferation	GO:0014842	2.96E-02	7.13E-02	1	8
5,6	ASPH|2.59965	regulation of cell communication by electrical coupling	GO:0010649	2.96E-02	7.13E-02	1	6
6	GCH1|4.01735	tetrahydrobiopterin metabolic process	GO:0046146	2.96E-02	7.13E-02	1	6
6	SLC2A1|5.081875	L-ascorbic acid metabolic process	GO:0019852	2.96E-02	7.13E-02	1	6
6,7	GSN|2.4555	sequestering of actin monomers	GO:0042989	2.96E-02	7.13E-02	1	7
7,8	CKB|0.486625	cellular anion homeostasis	GO:0030002	2.96E-02	7.13E-02	1	8
5,6	MAPK14|0.483325	regulation of cytokine production involved in inflammatory response	GO:1900015	2.96E-02	7.13E-02	1	6
5	ANXA5|3.637725;SQSTM1|6.007525;ATG7|2.120975;CHAF1B|0.2723	macroautophagy	GO:0016236	2.97E-02	7.13E-02	4	5
5	HSPA8|0.3721;ENO1|2.1939;ATP6V1B2|2.0575;GMPS|0.496875;ALDOC|2.04725;STAT3|0.4995	purine-containing compound metabolic process	GO:0072521	2.98E-02	7.14E-02	6	5
5	PRDX5|3.0143;MAPK14|0.483325;SOS2|11.48525	homeostasis of number of cells	GO:0048872	3.00E-02	7.16E-02	3	5
6,7,8	HSPA8|0.3721;SNW1|0.473175	regulation of RNA	GO:0043484	3.01E-02	7.20E-02	2	8
6	MTHFD1L|6.667175;ASL|2.252975;GCH1|4.01735;PLP1|3.936	carboxylic acid biosynthetic process	GO:0046394	3.03E-02	7.22E-02	4	6
5	MTHFD1L|6.667175;ASL|2.252975;GCH1|4.01735;PLP1|3.936	organic acid biosynthetic process	GO:0016053	3.03E-02	7.27E-02	4	5
7,8	VIM|2.241075;MAPK14|0.483325;SNW1|0.473175;TTK|0.488375	transmembrane receptor protein serine/threonine kinase signaling pathway	GO:0007178	3.06E-02	7.27E-02	4	8
4,5,6,7	ASPH|2.59965;BAZ1B|0.460625	regulation of receptor activity	GO:0010469	3.06E-02	7.32E-02	2	7
4,7,8	HSP90AB1|0.458775;MAPK14|0.483325	positive regulation of protein localization to nucleus	GO:1900182	3.12E-02	7.33E-02	2	8
5,6,10,11	ASPH|2.59965;ERO1L|2.370575	sequestering of calcium	GO:0051208	3.12E-02	7.44E-02	2	11
4	KRT1|0.4198;ANXA5|3.637725;VTN|2.01125	negative regulation of response to external stimulus	GO:0032102	3.12E-02	7.44E-02	3	4
6,7,8,9	G3BP1|0.3904;PSME3|0.401575;MAPK14|0.483325	regulation of canonical Wnt signaling pathway	GO:0060828	3.12E-02	7.44E-02	3	9
5	GSN|2.4555;MAPK14|0.483325;STAT3|0.4995;VTN|2.01125;ARHGDIA|3.8215	regulation of cell migration	GO:0030334	3.16E-02	7.44E-02	5	5
5,6	KRT1|0.4198;KRT9|0.268525;TXNIP|2.0553	skin development	GO:0043588	3.18E-02	7.52E-02	3	6
5	ENO1|2.1939;ALDOC|2.04725;STAT3|0.4995	carbohydrate catabolic process	GO:0016052	3.18E-02	7.53E-02	3	5
4	DNMT1|0.172;HIST1H4A|0.23835;BAZ1B|0.460625;ATG7|2.120975;SNW1|0.473175	histone modification	GO:0016570	3.21E-02	7.53E-02	5	4
6,7	RPL3|3.30745;RPL4|2.063325;RPL27|2.15885	protein targeting to membrane	GO:0006612	3.22E-02	7.53E-02	3	7
5	STAT3|0.4995	negative regulation of cofactor metabolic process	GO:0051195	3.22E-02	7.53E-02	1	5
7	PLEK|2.071125	positive regulation of actin filament depolymerization	GO:0030836	3.22E-02	7.53E-02	1	7
6,7,8,9	PLK1|0.415475	regulation of mitotic spindle assembly	GO:1901673	3.22E-02	7.53E-02	1	9

3,5	HSP90AB1|0.458775	adhesion of symbiont to host	GO:0044406	3.22E-02	7.53E-02	1	5
6	STAT3|0.4995	negative regulation of coenzyme metabolic process	GO:0051198	3.22E-02	7.53E-02	1	6
7,8,9	SNW1|0.473175	positive regulation by host of viral transcription	GO:0043923	3.22E-02	7.53E-02	1	9
6,7,8	PLEK|2.071125	regulation of inositol phosphate biosynthetic process	GO:0010919	3.22E-02	7.53E-02	1	8
7	STAT3|0.4995	skeletal muscle satellite cell proliferation	GO:0014841	3.22E-02	7.53E-02	1	7
7,8,9	MYH10|5.579275	ventricular cardiac muscle cell development	GO:0055015	3.22E-02	7.53E-02	1	9
7,10,11	MAPK14|0.483325	3'-UTR-mediated mRNA stabilization	GO:0070935	3.22E-02	7.53E-02	1	11
4,5	AKR1B1|2.235325	response to water	GO:0009415	3.22E-02	7.53E-02	1	5
4,5	HSPD1|2.88995	immune response to tumor cell	GO:0002418	3.22E-02	7.53E-02	1	5
5,7	ASPH|2.59965	detection of calcium ion	GO:0005513	3.22E-02	7.53E-02	1	7
6,7	MYH10|5.579275	nuclear migration	GO:0007097	3.22E-02	7.53E-02	1	7
6,8	AKR1B1|2.235325	norepinephrine metabolic process	GO:0042415	3.22E-02	7.53E-02	1	8
4,5	MYH10|5.579275	lateral ventricle development	GO:0021670	3.22E-02	7.53E-02	1	5
6,7	PLEK|2.071125	thrombin receptor signaling pathway	GO:0070493	3.22E-02	7.53E-02	1	7
5,6	HSPD1|2.88995	positive regulation of macrophage activation	GO:0043032	3.22E-02	7.53E-02	1	6
6,7	SNW1|0.473175	vitamin D receptor signaling pathway	GO:0070561	3.22E-02	7.53E-02	1	7
6,7,9,11	VIM|2.241075;ARHGDIA|3.8215	negative regulation of neuron projection development	GO:0010977	3.23E-02	7.53E-02	2	11
4	TOP2A|0.481675;SNW1|0.473175	positive regulation of viral process	GO:0048524	3.23E-02	7.54E-02	2	4
6,7	RPL3|3.30745;RPL4|2.063325;RPL27|2.15885	translational elongation	GO:0006414	3.25E-02	7.54E-02	3	7
5,7	HSP90AB1|0.458775;ASPH|2.59965;MAPK14|0.483325	positive regulation of cytoplasmic transport	GO:1903651	3.25E-02	7.56E-02	3	7
4	AKR1B1|2.235325;ISYNA1|0.49065;PLEK|2.071125;ALB|0.23405;GCH1|4.01735	organic hydroxy compound metabolic	GO:1901615	3.32E-02	7.56E-02	5	4
4	HSPD1|2.88995;ATP6V1B2|2.0575;AKR1B1|2.235325;STAT3|0.4995;TXNIP|2.0553;RBBP7|0.218475;H3F3A|0.209	response to hormone	GO:0009725	3.33E-02	7.73E-02	7	4
4	HSP90AB1|0.458775;VTN|2.01125;PLK1|0.415475	regulation of binding	GO:0051098	3.35E-02	7.75E-02	3	4
8	GSN|2.4555;PLEK|2.071125	actin filament depolymerization	GO:0030042	3.35E-02	7.77E-02	2	8
4	ACTG1|2.1999;LCP1|2.170475;VIM|2.241075;GSN|2.4555;RHOG|2.449775;PLEK|2.071125;MYH10|5.579275	actin filament-based process	GO:0030029	3.36E-02	7.77E-02	7	4
4	HSP90AB1|0.458775;GSN|2.4555;PLEK|2.071125;GCH1|4.01735;SOD2|2.175425	regulation of anatomical structure size	GO:0090066	3.38E-02	7.79E-02	5	4
7	DNMT1|0.172;HIST1H4A|0.23835;BAZ1B|0.460625;ATG7|2.120975;SNW1|0.473175	covalent chromatin modification	GO:0016569	3.40E-02	7.83E-02	5	7
7	GSN|2.4555;VTN|2.01125	regulation of protein processing	GO:0070613	3.41E-02	7.86E-02	2	7
6	GSN|2.4555;VTN|2.01125	regulation of protein maturation	GO:1903317	3.41E-02	7.87E-02	2	6
7,8	HSP90AB1|0.458775;LAMTOR2|7.841025;SQSTM1|6.007525;MAPK14|0.483325;VTN|2.01125;TTK|0.488375;PLK1|0.415475	positive regulation of phosphorylation	GO:0042327	3.46E-02	7.87E-02	7	8
6,8	HSP90AB1|0.458775;MAPK14|0.483325	positive regulation of nucleocytoplasmic transport	GO:0046824	3.47E-02	7.92E-02	2	8
4	ATG7|2.120975;MYH10|5.579275	metencephalon development	GO:0022037	3.47E-02	7.92E-02	2	4
10,11	ATP6V1B2|2.0575;TFRC|0.403125	cellular iron ion homeostasis	GO:0006879	3.47E-02	7.92E-02	2	11
6	STAT3|0.4995	regulation of hydrogen peroxide metabolic	GO:0010310	3.49E-02	7.92E-02	1	6
4,6,7	ALB|0.23405	killing of cells in other organism involved in symbiotic interaction	GO:0051883	3.49E-02	7.92E-02	1	7
6	GSN|2.4555	response to folic acid	GO:0051593	3.49E-02	7.92E-02	1	6
7,8,9,11,12	DNMT1|0.172	regulation of histone H3-K9 methylation	GO:0051570	3.49E-02	7.92E-02	1	12
5	ERO1L|2.370575	4-hydroxyproline metabolic process	GO:0019471	3.49E-02	7.92E-02	1	5
4	AKR1B1|2.235325	collecting duct	GO:0072044	3.49E-02	7.92E-02	1	4
7,8	PLK1|0.415475	positive regulation of metaphase/anaphase transition of cell cycle	GO:1902101	3.49E-02	7.92E-02	1	8
6,7,8,9,10,11,12	PLK1|0.415475	positive regulation of mitotic metaphase/anaphase transition	GO:0045842	3.49E-02	7.92E-02	1	12
5	ESD|2.155525	aldehyde catabolic process	GO:0046185	3.49E-02	7.92E-02	1	5
5	STAT3|0.4995	regulation of skeletal muscle cell proliferation	GO:0014857	3.49E-02	7.92E-02	1	5

6,7,8,10,11	DNMT1|0.172	negative regulation of histone methylation	GO:0031061	3.49E-02	7.92E-02	1	11
5	MAPK14|0.483325	cytokine production involved in inflammatory response	GO:0002534	3.49E-02	7.92E-02	1	5
5,6	ALB|0.23405	disruption of cells of other organism involved in symbiotic interaction	GO:0051818	3.49E-02	7.92E-02	1	6
5,6,7	TFRC|0.403125	positive regulation of bone remodeling	GO:0046852	3.49E-02	7.92E-02	1	7
4,5,6,7,8	TFRC|0.403125	positive regulation of bone resorption	GO:0045780	3.49E-02	7.92E-02	1	8
3	ARHGDIA|3.8215	response to redox state	GO:0051775	3.49E-02	7.92E-02	1	3
5	TUBG1|0.4102;TTK|0.488375;PLK1|0.415475	spindle organization	GO:0007051	3.51E-02	7.92E-02	3	5
4	NSF|2.017425;RHOG|2.449775;NDRG1|10.0073;MYH10|5.579275;PLK1|0.415475	endomembrane system organization	GO:0010256	3.51E-02	7.96E-02	5	4
4,5	ASPH|2.59965;STAT3|0.4995;S100A11|2.531875;NDRG1|10.0073;SOD2|2.175425	negative regulation of cell proliferation	GO:0008285	3.51E-02	7.96E-02	5	5
5,6,7,8	SNW1|0.473175;TTK|0.488375	positive regulation of transmembrane receptor protein serine/threonine kinase signaling pathway	GO:0090100	3.53E-02	7.96E-02	2	8
5	AKR1B1|2.235325;GCH1|4.01735	phenol-containing compound metabolic process	GO:0018958	3.53E-02	7.97E-02	2	5
5	HSP90AB1|0.458775;VTN|2.01125	positive regulation of binding	GO:0051099	3.53E-02	7.97E-02	2	5
7,9	HSPD1|2.88995;GSN|2.4555;PRDX5|3.0143	regulation of cysteine-type endopeptidase activity involved in apoptotic process	GO:0043281	3.55E-02	7.97E-02	3	9
3	HSP90AB1|0.458775;ASPH|2.59965;YWHAG|2.359475;ATG7|2.120975;MAPK14|0.483325;VTN|2.01125	positive regulation of transport	GO:0051050	3.55E-02	8.01E-02	6	3
4	HSPD1|2.88995;TFRC|0.403125;STAT3|0.4995;TXNIP|2.0	response to drug	GO:0042493	3.59E-02	8.02E-02	4	4
7	HSP90AB1|0.458775;HSPD1|2.88995;GSN|2.4555;SOD2|2.175425	cell-type specific apoptotic process	GO:0097285	3.64E-02	8.10E-02	4	7
6	MYH10|5.579275;GCH1|4.01735	neuromuscular process	GO:0050905	3.65E-02	8.20E-02	2	6
5	TFRC|0.403125;SNW1|0.473175	response to retinoic acid	GO:0032526	3.65E-02	8.20E-02	2	5
5,6	PSME3|0.401575;NDRG1|10.0073;PLK1|0.415475	positive regulation of cell cycle process	GO:0090068	3.65E-02	8.20E-02	3	6
4,5	ASPH|2.59965;ERO1L|2.370575	sequestering of metal ion	GO:0051238	3.71E-02	8.20E-02	2	5
4	ATG7|2.120975;NDE1|0.446425;MYH10|5.579275	telencephalon	GO:0021537	3.72E-02	8.30E-02	3	4
4	SNW1|0.473175;VTN|2.01125;PTP4A3|0.3433	regulation of cellular response to growth factor stimulus	GO:0090287	3.72E-02	8.30E-02	3	4
6,7,8,9	KPNA2|0.186675	NLS-bearing protein import into nucleus	GO:0006607	3.75E-02	8.30E-02	1	9
8	PLK1|0.415475	protein localization to chromatin	GO:0071168	3.75E-02	8.30E-02	1	8
6	PLP1|3.936	axon ensheathment in central nervous system	GO:0032291	3.75E-02	8.30E-02	1	6
4,6	TUBG1|0.4102	meiotic spindle	GO:0000212	3.75E-02	8.30E-02	1	6
7,8,9	PLP1|3.936	central nervous system myelination	GO:0022010	3.75E-02	8.30E-02	1	9
5,6	PLK1|0.415475	regulation of spindle assembly	GO:0090169	3.75E-02	8.30E-02	1	6
6	MAPK14|0.483325	response to muramyl dipeptide	GO:0032495	3.75E-02	8.30E-02	1	6
5	HSPD1|2.88995	response to misfolded protein	GO:0051788	3.75E-02	8.30E-02	1	5
6	ASL|2.252975	ornithine metabolic	GO:0006591	3.75E-02	8.30E-02	1	6
9	GSN|2.4555	hepatocyte apoptotic process	GO:0097284	3.75E-02	8.30E-02	1	9
5	TFRC|0.403125	response to manganese ion	GO:0010042	3.75E-02	8.30E-02	1	5
6,7	LIG1|0.440425	DNA replication, removal of RNA primer	GO:0043137	3.75E-02	8.30E-02	1	7
6,7,8	LIG1|0.440425	DNA replication, Okazaki fragment processing	GO:0033567	3.75E-02	8.30E-02	1	8
5,7,8	ATG7|2.120975	mucus secretion	GO:0070254	3.75E-02	8.30E-02	1	8
6	VIM|2.241075	lens fiber cell	GO:0070307	3.75E-02	8.30E-02	1	6
9	CKB|0.486625	chloride ion homeostasis	GO:0055064	3.75E-02	8.30E-02	1	9
5	MTHFD1L|6.667175;SLC25A1|2.1121;GCH1|4.01735	cofactor biosynthetic process	GO:0051188	3.75E-02	8.30E-02	3	5
5	VIM|2.241075;MYH10|5.579275	actin filament-based movement	GO:0030048	3.77E-02	8.30E-02	2	5
6	ASPH|2.59965;GCH1|4.01735;SOD2|2.175425;PTP4A3|0.	blood circulation	GO:0008015	3.84E-02	8.32E-02	4	6
6	HSP90AB1|0.458775;SRRT|0.39875;SNW1|0.473175;ARHGDIA|3.8215	cellular response to organic cyclic compound	GO:0071407	3.87E-02	8.49E-02	4	6
4	ACTG1|2.1999;LCP1|2.170475;HSPD1|2.88995;GSN|2.4555;PLEK|2.071125;ACTB|2.1999;SOS2|11.48525	cell-cell adhesion	GO:0098609	3.88E-02	8.54E-02	7	4
4	HSPD1|2.88995;GSN|2.4555;VTN|2.01125;ARHGDIA|3.8215;SOS2|11.48525	regulation of cell adhesion	GO:0030155	3.89E-02	8.56E-02	5	4
5	ASPH|2.59965;GCH1|4.01735;SOD2|2.175425;PTP4A3|0.	circulatory system process	GO:0003013	3.90E-02	8.57E-02	4	5
4,6	HSPD1|2.88995;TFRC|0.403125;NDRG1|10.0073	response to hypoxia	GO:0001666	3.93E-02	8.57E-02	3	6
5,7	NDE1|0.446425;CETN3|0.4171	centrosome cycle	GO:0007098	3.95E-02	8.64E-02	2	7
5,6,7	ASPH|2.59965;AKR1B1|2.235325;LAMTOR2|7.841025;SQSTM1|6.007525;MAPK14|0.483325;RHOG|2.449775;PLEK|2.071125;ARHGDIA|3.8215;SOS2|11.48525;SOD2|2.1	regulation of intracellular signal transduction	GO:1902531	3.97E-02	8.68E-02	10	7

6	SLC2A1|5.081875	oligosaccharide biosynthetic process	GO:0009312	4.01E-02	8.70E-02	1	6
6,7	STAT3|0.4995	negative regulation of nucleotide catabolic	GO:0030812	4.01E-02	8.70E-02	1	7
5,7	ATG7|2.120975	modulation by virus of host process	GO:0019054	4.01E-02	8.70E-02	1	7
8,9,10,11	PABPC1|0.3285	regulation of nuclear-transcribed mRNA poly(A) tail shortening	GO:0060211	4.01E-02	8.70E-02	1	11
5	MYH10|5.579275	adult heart development	GO:0007512	4.01E-02	8.70E-02	1	5
8,9,10,11,12	PABPC1|0.3285	positive regulation of nuclear-transcribed mRNA poly(A) tail	GO:0060213	4.01E-02	8.70E-02	1	12
7	RRM2|0.21675	deoxyribonucleoside diphosphate metabolic process	GO:0009186	4.01E-02	8.70E-02	1	7
7,8	ALDH1L2|2.148	dicarboxylic acid catabolic process	GO:0043649	4.01E-02	8.70E-02	1	8
7	GCH1|4.01735	neuromuscular process controlling posture	GO:0050884	4.01E-02	8.70E-02	1	7
6	STAT3|0.4995	skeletal muscle cell proliferation	GO:0014856	4.01E-02	8.70E-02	1	6
6,7,8	HSPD1|2.88995	positive regulation of interferon-alpha	GO:0032727	4.01E-02	8.70E-02	1	8
7,8,9,10,12	GSN|2.4555	positive regulation of actin nucleation	GO:0051127	4.01E-02	8.70E-02	1	12
6,8	HIST1H4A|0.23835	regulation of megakaryocyte differentiation	GO:0045652	4.01E-02	8.70E-02	1	8
5,6,7,8,9	PLK1|0.415475	positive regulation of mitotic sister chromatid separation	GO:1901970	4.01E-02	8.70E-02	1	9
6	GSN|2.4555	renal absorption	GO:0070293	4.01E-02	8.70E-02	1	6
5	ATG7|2.120975;MYH10|5.579275	cardiocyte differentiation	GO:0035051	4.01E-02	8.70E-02	2	5
3,4	LCP1|2.170475;VDAC3|2.015475;GSN|2.4555;RHOG|2.449775;PLEK|2.071125;PLK1|0.415475	regulation of cellular component biogenesis	GO:0044087	4.05E-02	8.70E-02	6	4
5	HSPD1|2.88995;TFRC|0.403125;NDRG1|10.0073	response to decreased oxygen levels	GO:0036293	4.11E-02	8.77E-02	3	5
7,8,9	HSP90AB1|0.458775;PSME3|0.401575;TTK|0.488375;PLK1|0.415475	regulation of proteasomal protein catabolic process	GO:0061136	4.13E-02	8.89E-02	4	9
6,7,9,10	DNMT1|0.172;SNW1|0.473175	histone lysine methylation	GO:0034968	4.14E-02	8.93E-02	2	10
4,5	TTK|0.488375;PLK1|0.415475	regulation of chromosome segregation	GO:0051983	4.20E-02	8.95E-02	2	5
6	ASPH|2.59965;GSN|2.4555	cellular response to metal ion	GO:0071248	4.20E-02	9.07E-02	2	6
5	HSPD1|2.88995;MAPK14|0.483325;GCH1|4.01735	response to lipopolysaccharide	GO:0032496	4.22E-02	9.07E-02	3	5
5	GSN|2.4555;NDE1|0.446425	establishment of cell polarity	GO:0030010	4.27E-02	9.09E-02	2	5
6,9	SOD2|2.175425	negative regulation of systemic arterial blood pressure	GO:0003085	4.27E-02	9.13E-02	1	9
5,7,8	LIG1|0.440425	DNA ligation involved in DNA repair	GO:0051103	4.27E-02	9.13E-02	1	8
6,7	PLEK|2.071125	integrin activation	GO:0033622	4.27E-02	9.13E-02	1	7
6	LIG1|0.440425	mitotic DNA replication	GO:1902969	4.27E-02	9.13E-02	1	6
6,7,8,9	MYH10|5.579275	cardiac myofibril	GO:0055003	4.27E-02	9.13E-02	1	9
6,9	SOD2|2.175425	neurological system process involved in regulation of systemic arterial blood pressure	GO:0001976	4.27E-02	9.13E-02	1	9
7	GSN|2.4555	cellular response to cadmium ion	GO:0071276	4.27E-02	9.13E-02	1	7
6	IFITM2|5.68315	response to interferon-beta	GO:0035456	4.27E-02	9.13E-02	1	6
7,8,9,10	HSP90AB1|0.458775	regulation of protein import into nucleus,	GO:0033158	4.27E-02	9.13E-02	1	10
5,7,8,9,10	TOP2A|0.481675	resolution of meiotic recombination intermediates	GO:0000712	4.27E-02	9.13E-02	1	10
5,6,7,8,9,10	VTN|2.01125	positive regulation of vascular endothelial growth factor receptor signaling pathway	GO:0030949	4.27E-02	9.13E-02	1	10
5,6	PSME3|0.401575;TOP2A|0.481675;TTK|0.488375;CDC123|0.29925;PLK1|0.415475	regulation of mitotic cell cycle	GO:0007346	4.32E-02	9.13E-02	5	6
6,8	ALDH1L2|2.148;ASL|2.252975	alpha-amino acid catabolic process	GO:1901606	4.33E-02	9.21E-02	2	8
6,7	YWHAG|2.359475;SOD2|2.175425	apoptotic mitochondrial changes	GO:0008637	4.33E-02	9.22E-02	2	7
6	GSN|2.4555;STAT3|0.4995	response to ethanol	GO:0045471	4.33E-02	9.22E-02	2	6
7,8	PSME3|0.401575;NDRG1|10.0073	DNA damage response, signal transduction by p53 class mediator	GO:0030330	4.40E-02	9.22E-02	2	8
6,7	TUBG1|0.4102;NDE1|0.446425	microtubule polymerization or	GO:0031109	4.40E-02	9.35E-02	2	7
5	HSPD1|2.88995;STAT3|0.4995;TXNIP|2.0553;RBBP7|0.218475	response to steroid hormone	GO:0048545	4.40E-02	9.35E-02	4	5

5	HSPA8|0.3721;HSP90AB1|0.458775;ENO1|2.1939;ATP6V1B2|2.0575;GMPS|0.496875;CKB|0.486625;YWHAG|2.359475;PRDX5|3.0143;BAZ1B|0.460625;ISYNA1|0.49065;ALDOC|2.04725;RRM2|0.21675;LAMTOR2|7.841025;SQSTM1|6.007525;MAPK14|0.483325;STAT3|0.4995;PLEK|2.071125;VTN|2.01125;TTK|0.488375;PLK1|0.415475;GCH1|4.01735;PTP4A3|0.3433	phosphate-containing compound metabolic process	GO:0006796	4.41E-02	9.35E-02	22	5
6,7	STAT3|0.4995;TXNIP|2.0553	response to estradiol	GO:0032355	4.46E-02	9.35E-02	2	7
8,9	GMPS|0.496875;STAT3|0.4995	purine ribonucleoside monophosphate biosynthetic process	GO:0009168	4.46E-02	9.43E-02	2	9
5,7,8	LIG1|0.440425;PCNA|0.404975	nucleotide-excision repair	GO:0006289	4.46E-02	9.43E-02	2	8
5	VIM|2.241075;ARHGDIA|3.8215	negative regulation of cell projection organization	GO:0031345	4.46E-02	9.43E-02	2	5
7,8	GMPS|0.496875;STAT3|0.4995	purine nucleoside monophosphate biosynthetic process	GO:0009127	4.46E-02	9.43E-02	2	8
5	HSPA8|0.3721;ACTG1|2.1999;HSP90AB1|0.458775;NSF|2.017425;GSN|2.4555;TFRC|0.403125;PLEK|2.071125;HBA1|2.13735;VTN|2.01125;ACTB|2.1999;MYH10|5.579275;	vesicle-mediated transport	GO:0016192	4.49E-02	9.43E-02	12	5
6	HSPD1|2.88995;ASPH|2.59965;GSN|2.4555;VTN|2.01125	protein processing	GO:0016485	4.51E-02	9.50E-02	4	6
8,9,10,11	ASPH|2.59965	activation of store-operated calcium channel	GO:0032237	4.54E-02	9.50E-02	1	11
5,6,7	SQSTM1|6.007525	negative regulation of growth of symbiont in	GO:0044130	4.54E-02	9.50E-02	1	7
5,6,7	PLEK|2.071125	negative regulation of alcohol biosynthetic	GO:1902931	4.54E-02	9.50E-02	1	7
6,7	HSPD1|2.88995	regulation of interferon-alpha production	GO:0032647	4.54E-02	9.50E-02	1	7
7,8	GCH1|4.01735	positive regulation of nitric-oxide synthase	GO:0051000	4.54E-02	9.50E-02	1	8
6,7	NSF|2.017425	plasma membrane fusion	GO:0045026	4.54E-02	9.50E-02	1	7
8,10	ASL|2.252975	arginine biosynthetic process	GO:0006526	4.54E-02	9.50E-02	1	10
6	GSN|2.4555	wound healing, spreading of epidermal cells	GO:0035313	4.54E-02	9.50E-02	1	6
5,6	MYH10|5.579275	establishment of nucleus localization	GO:0040023	4.54E-02	9.50E-02	1	6
5	LCP1|2.170475;HSPD1|2.88995;GSN|2.4555;SOS2|11.485	T cell activation	GO:0042110	4.54E-02	9.50E-02	4	5
4	LCP1|2.170475;HSPD1|2.88995;GSN|2.4555;SOS2|11.485	T cell aggregation	GO:0070489	4.54E-02	9.50E-02	4	4
7,8	LCP1|2.170475;HSPD1|2.88995;GSN|2.4555;SOS2|11.485	lymphocyte aggregation	GO:0071593	4.57E-02	9.50E-02	4	8
6,7,8	SQSTM1|6.007525;RHOG|2.449775;ARHGDIA|3.8215;SOS2|11.48525	regulation of small GTPase mediated signal transduction	GO:0051056	4.57E-02	9.55E-02	4	8
6	NDRG1|10.0073;PLP1|3.936	myelination	GO:0042552	4.59E-02	9.55E-02	2	6
6,7	ASPH|2.59965;ERO1L|2.370575	calcium ion transport into cytosol	GO:0060402	4.59E-02	9.58E-02	2	7
6,8,9	DNMT1|0.172;SNW1|0.473175	peptidyl-lysine	GO:0018022	4.59E-02	9.58E-02	2	9
4	PCNA|0.404975;MAPK14|0.483325;ARHGDIA|3.8215	cellular response to abiotic stimulus	GO:0071214	4.60E-02	9.58E-02	3	4
7	HSP90AB1|0.458775;YWHAG|2.359475;LAMTOR2|7.841025;SQSTM1|6.007525;MAPK14|0.483325;STAT3|0.4995;VTN|2.01125;TTK|0.488375;PLK1|0.415475	regulation of phosphorylation	GO:0042325	4.62E-02	9.58E-02	9	7
10,11	ASPH|2.59965;ERO1L|2.370575	cytosolic calcium ion transport	GO:0060401	4.66E-02	9.63E-02	2	11
6,7	LCP1|2.170475;HSPD1|2.88995;GSN|2.4555;SOS2|11.485	leukocyte aggregation	GO:0070486	4.77E-02	9.69E-02	4	7
5	NDRG1|10.0073;PLP1|3.936	axon ensheathment	GO:0008366	4.79E-02	9.83E-02	2	5
4	NDRG1|10.0073;PLP1|3.936	ensheathment of neurons	GO:0007272	4.79E-02	9.83E-02	2	4
6,7	GSN|2.4555	positive regulation of protein maturation	GO:1903319	4.80E-02	9.83E-02	1	7
5,6	PCNA|0.404975	regulation of nuclease activity	GO:0032069	4.80E-02	9.83E-02	1	6
4	SQSTM1|6.007525	negative regulation of growth of symbiont involved in interaction with host	GO:0044146	4.80E-02	9.83E-02	1	4
5	AKR1B1|2.235325	response to thyroid hormone	GO:0097066	4.80E-02	9.83E-02	1	5
5	AKR1B1|2.235325	glycoside metabolic process	GO:0016137	4.80E-02	9.83E-02	1	5
5,6	RRM2|0.21675	nucleobase-containing small molecule interconversion	GO:0015949	4.80E-02	9.83E-02	1	6
7,8	NDRG1|10.0073	peripheral nervous system myelin maintenance	GO:0032287	4.80E-02	9.83E-02	1	8
7,8,9,10	ASPH|2.59965	positive regulation of store-operated calcium channel activity	GO:1901341	4.80E-02	9.83E-02	1	10
6,7	STAT3|0.4995	negative regulation of nucleoside metabolic process	GO:0045978	4.80E-02	9.83E-02	1	7
6	HSPD1|2.88995	interferon-alpha	GO:0032607	4.80E-02	9.83E-02	1	6
7	GSN|2.4555	positive regulation of protein processing	GO:0010954	4.80E-02	9.83E-02	1	7
4,6	ATG7|2.120975	modulation by symbiont of host cellular process	GO:0044068	4.80E-02	9.83E-02	1	6

5	STAT3|0.4995	negative regulation of reactive oxygen species biosynthetic process	GO:1903427	4.80E-02	9.83E-02	1	5
7,8,9,10	STAT3|0.4995	negative regulation of ATP metabolic process	GO:1903579	4.80E-02	9.83E-02	1	10
4,5,6	SQSTM1|6.007525	regulation of growth of symbiont in host	GO:0044126	4.80E-02	9.83E-02	1	6
6,7,9	KRT1|0.4198	establishment of skin	GO:0061436	4.80E-02	9.83E-02	1	9
5,6,8	PLEK|2.071125	inositol phosphate biosynthetic process	GO:0032958	4.80E-02	9.83E-02	1	8
4,5	HSPD1|2.88995;GSN|2.4555;PLEK|2.071125;SOS2|11.485	regulation of cell	GO:0050865	4.85E-02	9.83E-02	4	5
5	GMPS|0.496875;ISYNA1|0.49065;RRM2|0.21675;STAT3|0.4995;PLEK|2.071125;GCH1|4.01735	organophosphate biosynthetic process	GO:0090407	4.92E-02	9.94E-02	6	5
9,10	MAPK14|0.483325;SLC2A1|5.081875	glucose import	GO:0046323	4.93E-02	1.01E-01	2	10
6,7	GSN|2.4555;PLEK|2.071125	actin cytoskeleton reorganization	GO:0031532	4.93E-02	1.01E-01	2	7
5,7	ACTG1|2.1999;HSP90AB1|0.458775;PSME3|0.401575;ACTB|2.1999	immune response-activating cell surface receptor signaling	GO:0002429	4.94E-02	1.01E-01	4	7
6,7	HSP90AB1|0.458775;YWHAG|2.359475;LAMTOR2|7.841025;SQSTM1|6.007525;MAPK14|0.483325;STAT3|0.4995;PLEK|2.071125;VTN|2.01125;TTK|0.488375;PLK1|0.415	regulation of phosphate metabolic process	GO:0019220	4.94E-02	1.01E-01	10	7
5,7	HSPD1|2.88995;MAPK14|0.483325;GCH1|4.01735	response to molecule of bacterial origin	GO:0002237	4.95E-02	1.01E-01	3	7
5,7,8	HSPD1|2.88995;PSME3|0.401575;MAPK14|0.483325	innate immune response-activating signal transduction	GO:0002758	4.95E-02	1.01E-01	3	8
4,5	LCP1|2.170475;GSN|2.4555;PLEK|2.071125;PLK1|0.415475	regulation of protein complex assembly	GO:0043254	4.97E-02	1.01E-01	4	5
7,8	ANXA5|3.637725;GCH1|4.01735;SOD2|2.175425	protein homooligomerization	GO:0051260	4.99E-02	1.01E-01	3	8
5,6	LCP1|2.170475;GSN|2.4555	extracellular matrix disassembly	GO:0022617	4.99E-02	1.01E-01	2	6
5,6,7,8	TOP2A|0.481675;PLK1|0.415475	meiosis I	GO:0007127	4.99E-02	1.01E-01	2	8
6,7	HSPD1|2.88995;GSN|2.4555;SOS2|11.48525	regulation of T cell activation	GO:0050863	5.03E-02	1.01E-01	3	7
8,9	ASPH|2.59965	regulation of store-operated calcium channel	GO:1901339	5.06E-02	1.02E-01	1	9
7	SOS2|11.48525	lymphoid progenitor cell differentiation	GO:0002320	5.06E-02	1.02E-01	1	7
6	IFITM2|5.68315	response to interferon-	GO:0035455	5.06E-02	1.02E-01	1	6
4,5,6	SQSTM1|6.007525	modulation of growth of symbiont involved in interaction with host	GO:0044144	5.06E-02	1.02E-01	1	6
5,7	ASL|2.252975	urea cycle	GO:0000050	5.06E-02	1.02E-01	1	7
6,7	PLK1|0.415475	regulation of mitotic spindle organization	GO:0060236	5.06E-02	1.02E-01	1	7
7	SNW1|0.473175	cellular response to vitamin D	GO:0071305	5.06E-02	1.02E-01	1	7
5,6,7,9	MAPK14|0.483325	positive regulation of myoblast fusion	GO:1901741	5.06E-02	1.02E-01	1	9
7	MYH10|5.579275	ventricular cardiac muscle cell differentiation	GO:0055012	5.06E-02	1.02E-01	1	7
6,7,9	PLEK|2.071125	positive regulation of protein depolymerization	GO:1901881	5.06E-02	1.02E-01	1	9
4,5	ZNF706|0.3974	regulation of stem cell maintenance	GO:2000036	5.06E-02	1.02E-01	1	5
6	PCNA|0.404975	error-free translesion synthesis	GO:0070987	5.06E-02	1.02E-01	1	6
5,6	HSPA8|0.3721;NSF|2.017425;ATG7|2.120975;MAPK14|0.483325;SLC2A1|5.081875;PLEK|2.071125;MYH10|5.579275;ALB|0.23405	secretion	GO:0046903	5.06E-02	1.02E-01	8	6
10	ATP6V1B2|2.0575;TFRC|0.403125	iron ion homeostasis	GO:0055072	5.06E-02	1.02E-01	2	10
5,6,7	SRRT|0.39875;STAT3|0.4995;ZNF706|0.3974	stem cell development	GO:0048864	5.11E-02	1.02E-01	3	7
7	TFRC|0.403125;HBA1|2.13735;VTN|2.01125;ALB|0.23405	receptor-mediated endocytosis	GO:0006898	5.12E-02	1.03E-01	4	7
5,6	HSP90AB1|0.458775;YWHAG|2.359475;LAMTOR2|7.841025;SQSTM1|6.007525;MAPK14|0.483325;STAT3|0.4995;PLEK|2.071125;VTN|2.01125;TTK|0.488375;PLK1|0.415	regulation of phosphorus metabolic process	GO:0051174	5.12E-02	1.03E-01	10	6
4,5,6	LCP1|2.170475;GSN|2.4555;PLEK|2.071125	positive regulation of protein complex assembly	GO:0031334	5.15E-02	1.03E-01	3	6
3	ACTG1|2.1999;LCP1|2.170475;HSPD1|2.88995;GSN|2.4555;PLEK|2.071125;VTN|2.01125;ARHGDIA|3.8215;ACTB|2.1999;MYH10|5.579275;SOS2|11.48525	cell adhesion	GO:0007155	5.19E-02	1.03E-01	10	3
4,6,7	HSPD1|2.88995;PSME3|0.401575;MAPK14|0.483325	activation of innate immune response	GO:0002218	5.23E-02	1.04E-01	3	7
6,7	PCNA|0.404975;S100A11|2.531875	regulation of DNA replication	GO:0006275	5.27E-02	1.05E-01	2	7
5,6	PSME3|0.401575;TTK|0.488375;PLK1|0.415475	negative regulation of cell cycle process	GO:0010948	5.27E-02	1.05E-01	3	6
7,8	GSN|2.4555	positive regulation of epithelial cell apoptotic process	GO:1904037	5.31E-02	1.05E-01	1	8
5,6,7	RHOG|2.449775	regulation of ruffle assembly	GO:1900027	5.31E-02	1.05E-01	1	7
5,6	MAPK14|0.483325	positive regulation of cardiac muscle cell proliferation	GO:0060045	5.31E-02	1.05E-01	1	6

6,7	MAPK14|0.483325	positive regulation of myoblast differentiation	GO:0045663	5.31E-02	1.05E-01	1	7
6,8	GCH1|4.01735	catecholamine biosynthetic process	GO:0042423	5.31E-02	1.05E-01	1	8
7,8,9	ALB|0.23405	sodium-independent organic anion transport	GO:0043252	5.31E-02	1.05E-01	1	9
7	NDRG1|10.0073	regulation of spindle checkpoint	GO:0090231	5.31E-02	1.05E-01	1	7
6,7,8	SOD2|2.175425	negative regulation of oxidative stress-induced intrinsic apoptotic signaling pathway	GO:1902176	5.31E-02	1.05E-01	1	8
5	MAPK14|0.483325	response to muscle stretch	GO:0035994	5.31E-02	1.05E-01	1	5
4,6,7,8,9	TOP2A|0.481675	meiotic chromosome separation	GO:0051307	5.31E-02	1.05E-01	1	9
7,8,9,10	STAT3|0.4995	positive regulation of ATP metabolic process	GO:1903580	5.31E-02	1.05E-01	1	10
6,8	KRT1|0.4198	regulation of water loss via skin	GO:0033561	5.31E-02	1.05E-01	1	8
7	GCH1|4.01735	catechol-containing compound biosynthetic process	GO:0009713	5.31E-02	1.05E-01	1	7
7,8,9,10	PABPC1|0.3285	regulation of nuclear-transcribed mRNA catabolic process, deadenylation-dependent decay	GO:1900151	5.31E-02	1.05E-01	1	10
6,7	STAT3|0.4995	positive regulation of nucleoside metabolic process	GO:0045979	5.31E-02	1.05E-01	1	7
7	PABPC1|0.3285	positive regulation of nuclear-transcribed mRNA catabolic process, deadenylation-dependent decay	GO:1900153	5.31E-02	1.05E-01	1	7
5,6	GSN|2.4555	regulation of establishment of cell	GO:2000114	5.31E-02	1.05E-01	1	6
6,7	PSME3|0.401575;TTK|0.488375;PLK1|0.415475	regulation of mitotic cell cycle phase transition	GO:1901990	5.32E-02	1.05E-01	3	7
5	ASPH|2.59965;GSN|2.4555	cellular response to inorganic substance	GO:0071241	5.34E-02	1.05E-01	2	5
6	ACTG1|2.1999;ACTB|2.1999	adherens junction organization	GO:0034332	5.34E-02	1.05E-01	2	6
6,7	NSF|2.017425;RHOG|2.449775	establishment of protein localization to plasma membrane	GO:0090002	5.34E-02	1.05E-01	2	7
8	HSPD1|2.88995;ASPH|2.59965	activation of cysteine-type endopeptidase activity	GO:0097202	5.34E-02	1.05E-01	2	8
6	HSPD1|2.88995;GSN|2.4555;SOS2|11.48525	regulation of leukocyte cell-cell adhesion	GO:1903037	5.36E-02	1.05E-01	3	6
5	KRT1|0.4198;BAZ1B|0.460625;ATG7|2.120975;PCNA|0.404975;MAPK14|0.483325;MYH10|5.579275	cardiovascular system development	GO:0072358	5.42E-02	1.05E-01	6	5
5	KRT1|0.4198;BAZ1B|0.460625;ATG7|2.120975;PCNA|0.404975;MAPK14|0.483325;MYH10|5.579275	circulatory system development	GO:0072359	5.42E-02	1.06E-01	6	5
5	NSF|2.017425;ATG7|2.120975;VAT1|2.702925	membrane fusion	GO:0061025	5.44E-02	1.06E-01	3	5
5	ATG7|2.120975;NDRG1|10.0073	cellular response to oxygen levels	GO:0071453	5.48E-02	1.07E-01	2	5
8,9	YWHAG|2.359475;PLK1|0.415475	negative regulation of protein serine/threonine kinase activity	GO:0071901	5.48E-02	1.07E-01	2	9
7	ASPH|2.59965;ATP6V1B2|2.0575;CKB|0.486625;ERO1L|2.370575;TFRC|0.403125	inorganic ion homeostasis	GO:0098771	5.49E-02	1.07E-01	5	7
8,9	ASPH|2.59965;ATP6V1B2|2.0575;ERO1L|2.370575;TFRC|0.403125	cellular metal ion homeostasis	GO:0006875	5.52E-02	1.07E-01	4	9
5	HSP90AB1|0.458775;YWHAG|2.359475;PSME3|0.401575;LAMTOR2|7.841025;MAPK14|0.483325;TTK|0.488375;PLK1|0.415475	regulation of transferase activity	GO:0051338	5.54E-02	1.08E-01	7	5
9,10	ATP6V1B2|2.0575;TFRC|0.403125	cellular transition metal ion homeostasis	GO:0046916	5.55E-02	1.08E-01	2	10
6	GMPS|0.496875;ASL|2.252975	glutamine family amino acid metabolic process	GO:0009064	5.55E-02	1.08E-01	2	6
6	PSME3|0.401575;TOP2A|0.481675	mitotic DNA integrity checkpoint	GO:0044774	5.55E-02	1.08E-01	2	6
8,9,10	ASPH|2.59965	regulation of cardiac muscle contraction by regulation of the release of sequestered calcium ion	GO:0010881	5.57E-02	1.08E-01	1	10
7,9	SNW1|0.473175	modulation by host of viral transcription	GO:0043921	5.57E-02	1.08E-01	1	9
7,8,9,10,11	PABPC1|0.3285	positive regulation of mRNA 3'-end processing	GO:0031442	5.57E-02	1.08E-01	1	11
8,9	PLK1|0.415475	positive regulation of peptidyl-threonine phosphorylation	GO:0010800	5.57E-02	1.08E-01	1	9
5,7,8,9	ATG7|2.120975	negative stranded viral RNA replication	GO:0039689	5.57E-02	1.08E-01	1	9

6,7,8	VTN|2.01125	positive regulation of smooth muscle cell migration	GO:0014911	5.57E-02	1.08E-01	1	8
8,9	SLC25A1|2.1121	long-chain fatty-acyl-CoA biosynthetic process	GO:0035338	5.57E-02	1.08E-01	1	9
4,5	PLK1|0.415475	positive regulation of chromosome segregation	GO:0051984	5.57E-02	1.08E-01	1	5
6,7	SNW1|0.473175	modulation by host of symbiont transcription	GO:0052472	5.57E-02	1.08E-01	1	7
4	ATG7|2.120975	multi-organism biosynthetic process	GO:0044034	5.57E-02	1.08E-01	1	4
4,5	ASL|2.252975	urea metabolic process	GO:0019627	5.57E-02	1.08E-01	1	5
5	SRRT|0.39875	neuronal stem cell maintenance	GO:0097150	5.57E-02	1.08E-01	1	5
3	KRT1|0.4198;ANXA5|3.637725;G3BP1|0.3904;PSME3|0.401575;MAPK14|0.483325;PLEK|2.071125;VTN|2.01125;SOD2|2.175425	negative regulation of response to stimulus	GO:0048585	5.59E-02	1.08E-01	8	3
4,5	GSN|2.4555;ALB|0.23405	maintenance of location in cell	GO:0051651	5.62E-02	1.08E-01	2	5
5	AKR1B1|2.235325;TXNIP|2.0553	response to ketone	GO:1901654	5.69E-02	1.08E-01	2	5
5,6	LCP1|2.170475;HSPD1|2.88995;GSN|2.4555;SOS2|11.485	leukocyte cell-cell	GO:0007159	5.70E-02	1.10E-01	4	6
6,7	HSPD1|2.88995;GSN|2.4555;SOS2|11.48525	regulation of homotypic cell-cell adhesion	GO:0034110	5.74E-02	1.10E-01	3	7
7	ASPH|2.59965;DNMT1|0.172;BAZ1B|0.460625;ATG7|2.120975;MAPK14|0.483325;SNW1|0.473175;VTN|2.01125;TTK|0.488375;PLK1|0.415475	peptidyl-amino acid modification	GO:0018193	5.75E-02	1.11E-01	9	7
4	ATG7|2.120975;NDE1|0.446425	cerebral cortex development	GO:0021987	5.76E-02	1.11E-01	2	4
5,6	SNW1|0.473175	modulation of transcription in other organism involved in	GO:0052312	5.83E-02	1.11E-01	1	6
6	RPL3|3.30745	ribosomal large subunit assembly	GO:0000027	5.83E-02	1.11E-01	1	6
5,7	SQSTM1|6.007525	growth of symbiont in	GO:0044117	5.83E-02	1.11E-01	1	7
7,8	HSPD1|2.88995	chaperone-mediated protein complex assembly	GO:0051131	5.83E-02	1.11E-01	1	8
5	AKR1B1|2.235325	C21-steroid hormone biosynthetic process	GO:0006700	5.83E-02	1.11E-01	1	5
4,5,6	NSF|2.017425	regulation of receptor recycling	GO:0001919	5.83E-02	1.11E-01	1	6
6,7,9	MAPK14|0.483325	regulation of myoblast fusion	GO:1901739	5.83E-02	1.11E-01	1	9
6	RRM2|0.21675	deoxyribonucleotide biosynthetic process	GO:0009263	5.83E-02	1.11E-01	1	6
10,11	TFRC|0.403125	iron ion import	GO:0097286	5.83E-02	1.11E-01	1	11
5,6,7	MAPK14|0.483325	positive regulation of cardiac muscle tissue growth	GO:0055023	5.83E-02	1.11E-01	1	7
7,8	ASPH|2.59965;PLEK|2.071125	calcium-mediated	GO:0019722	5.83E-02	1.11E-01	2	8
4	TFRC|0.403125;NCDN|0.4114	tissue remodeling	GO:0048771	5.90E-02	1.11E-01	2	4
6,7	HSP90AB1|0.458775;IFITM2|5.68315	cellular response to type I interferon	GO:0071357	5.98E-02	1.13E-01	2	7
7,8	HSP90AB1|0.458775;IFITM2|5.68315	type I interferon signaling pathway	GO:0060337	5.98E-02	1.14E-01	2	8
4,5,6	PSME3|0.401575;NDRG1|10.0073;PLK1|0.415475	positive regulation of cell cycle	GO:0045787	6.00E-02	1.14E-01	3	6
5	VIM|2.241075;VDAC3|2.015475;RHOG|2.449775;ARHGDIA|3.8215	regulation of cell projection organization	GO:0031344	6.03E-02	1.14E-01	4	5
7,8	HSP90AB1|0.458775;PSME3|0.401575;TTK|0.488375;PLK1|0.415475	regulation of proteolysis involved in cellular protein catabolic process	GO:1903050	6.03E-02	1.14E-01	4	8
6	PSME3|0.401575;TTK|0.488375;PLK1|0.415475	regulation of cell cycle phase transition	GO:1901987	6.04E-02	1.14E-01	3	6
5	YWHAG|2.359475;NCDN|0.4114	regulation of synaptic plasticity	GO:0048167	6.05E-02	1.14E-01	2	5
5,6	HSP90AB1|0.458775;IFITM2|5.68315	response to type I interferon	GO:0034340	6.05E-02	1.14E-01	2	6
4,6	HIST1H4A|0.23835;MAPK14|0.483325;SOS2|11.48525	regulation of hemopoiesis	GO:1903706	6.09E-02	1.14E-01	3	6
4,5	GSN|2.4555	regulation of establishment or maintenance of cell	GO:0032878	6.09E-02	1.14E-01	1	5
5,6,7	PRDX5|3.0143	positive regulation of collagen metabolic	GO:0010714	6.09E-02	1.14E-01	1	7
5	LIG1|0.440425	V(D)J recombination	GO:0033151	6.09E-02	1.14E-01	1	5
4,5,6	MAPK14|0.483325	positive regulation of erythrocyte differentiation	GO:0045648	6.09E-02	1.14E-01	1	6
6,7	LIG1|0.440425	lagging strand elongation	GO:0006273	6.09E-02	1.14E-01	1	7
3,6,7	MAPK14|0.483325	cartilage condensation	GO:0001502	6.09E-02	1.14E-01	1	7
6,7	NDRG1|10.0073	myelin maintenance	GO:0043217	6.09E-02	1.14E-01	1	7
2	MAPK14|0.483325	cell aggregation	GO:0098743	6.09E-02	1.14E-01	1	2
5,6,7,8	PLEK|2.071125	positive regulation of blood coagulation	GO:0030194	6.09E-02	1.14E-01	1	8
5,6	HSPD1|2.88995	positive regulation of interleukin-10 production	GO:0032733	6.09E-02	1.14E-01	1	6
6,7,9,10	MAPK14|0.483325	positive regulation of blood vessel endothelial cell migration	GO:0043536	6.09E-02	1.14E-01	1	10

7	STAT3|0.4995	cellular response to interleukin-6	GO:0071354	6.09E-02	1.14E-01	1	7
6	PLK1|0.415475	regulation of spindle organization	GO:0090224	6.09E-02	1.14E-01	1	6
4,6	SQSTM1|6.007525	growth of symbiont involved in interaction with host	GO:0044116	6.09E-02	1.14E-01	1	6
6,7	PLEK|2.071125	positive regulation of phosphatase activity	GO:0010922	6.09E-02	1.14E-01	1	7
6	PRDX5|3.0143	positive regulation of collagen biosynthetic process	GO:0032967	6.09E-02	1.14E-01	1	6
7,8	SLC25A1|2.1121	fatty-acyl-CoA biosynthetic process	GO:0046949	6.09E-02	1.14E-01	1	8
4	ASL|2.252975	nitrogen cycle metabolic process	GO:0071941	6.09E-02	1.14E-01	1	4
4	PLEK|2.071125	positive regulation of hemostasis	GO:1900048	6.09E-02	1.14E-01	1	4
3,5	SQSTM1|6.007525	growth involved in symbiotic interaction	GO:0044110	6.09E-02	1.14E-01	1	5
5,6,7	HSP90AB1|0.458775;LCP1|2.170475;ASPH|2.59965;MAPK14|0.483325;SLC2A1|5.081875	regulation of protein transport	GO:0051223	6.12E-02	1.14E-01	5	7
7,8	GMPS|0.496875;STAT3|0.4995	ribonucleoside monophosphate biosynthetic process	GO:0009156	6.12E-02	1.14E-01	2	8
5,6,7	PCNA|0.404975;TOP2A|0.481675	positive regulation of DNA metabolic process	GO:0051054	6.19E-02	1.14E-01	2	7
5,7	ALDH1L2|2.148;ASL|2.252975	cellular amino acid catabolic process	GO:0009063	6.27E-02	1.16E-01	2	7
5,6,7	YWHAG|2.359475;STAT3|0.4995;PLEK|2.071125;PLK1|0.415475	negative regulation of phosphorus metabolic process	GO:0010563	6.29E-02	1.17E-01	4	7
6	YWHAG|2.359475;STAT3|0.4995;PLEK|2.071125;PLK1|0.415475	negative regulation of phosphate metabolic process	GO:0045936	6.29E-02	1.17E-01	4	6
5,6	HSP90AB1|0.458775;PSME3|0.401575;LAMTOR2|7.841025;MAPK14|0.483325;PLK1|0.415475	positive regulation of transferase activity	GO:0051347	6.31E-02	1.17E-01	5	6
4	ENO1|2.1939;RBBP7|0.218475	negative regulation of cell growth	GO:0030308	6.34E-02	1.17E-01	2	4
6,7	PLEK|2.071125;PLP1|3.936	integrin-mediated signaling pathway	GO:0007229	6.34E-02	1.17E-01	2	7
6	PABPC1|0.3285	positive regulation of mRNA catabolic process	GO:0061014	6.34E-02	1.17E-01	1	6
6,7	GCH1|4.01735	positive regulation of monooxygenase activity	GO:0032770	6.34E-02	1.17E-01	1	7
6,7,8,10,11	DNMT1|0.172	histone H3-K9	GO:0051567	6.34E-02	1.17E-01	1	11
5,6	ALDH1L2|2.148	cellular modified amino acid catabolic process	GO:0042219	6.34E-02	1.17E-01	1	6
5,6	GSN|2.4555	membrane raft	GO:0031579	6.34E-02	1.17E-01	1	6
5	GSN|2.4555	actin filament severing	GO:0051014	6.34E-02	1.17E-01	1	5
7,8,9	STAT3|0.4995	JAK-STAT cascade involved in growth hormone signaling	GO:0060397	6.34E-02	1.17E-01	1	9
7,8,9	MAPK14|0.483325	p38MAPK cascade	GO:0038066	6.34E-02	1.17E-01	1	9
6,8,9	HSP90AB1|0.458775	regulation of interferon-gamma-mediated signaling pathway	GO:0060334	6.34E-02	1.17E-01	1	9
6,7,8,9,11	GSN|2.4555	positive regulation of cysteine-type endopeptidase activity involved in apoptotic signaling pathway	GO:2001269	6.34E-02	1.17E-01	1	11
5,6	ACTG1|2.1999;LCP1|2.170475;GSN|2.4555;RHOG|2.449775;PLEK|2.071125;MYH10|5.579275	actin cytoskeleton organization	GO:0030036	6.36E-02	1.17E-01	6	6
5,6	ASPH|2.59965;ATP6V1B2|2.0575;CKB|0.486625;ERO1L|2.370575;TFRC|0.403125	cellular chemical homeostasis	GO:0055082	6.39E-02	1.17E-01	5	6
4,5	LCP1|2.170475;HSPD1|2.88995	lymphocyte activation involved in immune response	GO:0002285	6.42E-02	1.18E-01	2	5
7	MAPK14|0.483325;PTP4A3|0.3433	endothelial cell migration	GO:0043542	6.49E-02	1.18E-01	2	7
4	ATG7|2.120975;MYH10|5.579275	hindbrain development	GO:0030902	6.49E-02	1.19E-01	2	4
8,9	RHOG|2.449775;ARHGDIA|3.8215;SOS2|11.48525	Rho protein signal transduction	GO:0007266	6.49E-02	1.19E-01	3	9
2	HSP90AB1|0.458775;CCT7|2.06135;KRT9|0.268525;AKR1B1|2.235325;EIF4H|0.275675;MAPK14|0.483325;STAT3|	reproductive process	GO:0022414	6.53E-02	1.19E-01	7	2
3	TOP2A|0.481675;SNW1|0.473175	positive regulation of multi-organism process	GO:0043902	6.56E-02	1.20E-01	2	3
4	HSP90AB1|0.458775;MAPK14|0.483325	placenta development	GO:0001890	6.56E-02	1.20E-01	2	4
6,7	HSP90AB1|0.458775;PSME3|0.401575;TTK|0.488375;PLK1|0.415475	regulation of cellular protein catabolic process	GO:1903362	6.60E-02	1.20E-01	4	7
5	ATG7|2.120975	microautophagy	GO:0016237	6.60E-02	1.20E-01	1	5
5,6	ATG7|2.120975	single-organism membrane invagination	GO:1902534	6.60E-02	1.20E-01	1	6
6,7	ATG7|2.120975	piecemeal microautophagy of nucleus	GO:0034727	6.60E-02	1.20E-01	1	7
4	TFRC|0.403125	positive regulation of tissue remodeling	GO:0034105	6.60E-02	1.20E-01	1	4

5	HSP90AB1|0.458775	regulation of response to interferon-gamma	GO:0060330	6.60E-02	1.20E-01	1	5
5	ASPH|2.59965	cell communication by electrical coupling	GO:0010644	6.60E-02	1.20E-01	1	5
4,5,6	MAPK14|0.483325	positive regulation of syncytium formation by plasma membrane fusion	GO:0060143	6.60E-02	1.20E-01	1	6
7	ATG7|2.120975	late nucleophagy	GO:0044805	6.60E-02	1.20E-01	1	7
8,9	ASPH|2.59965	regulation of cardiac muscle contraction by calcium ion signaling	GO:0010882	6.60E-02	1.20E-01	1	9
5	TFRC|0.403125	response to iron ion	GO:0010039	6.60E-02	1.20E-01	1	5
5,6,7,8,9	SNW1|0.473175	positive regulation of transforming growth factor beta receptor signaling pathway	GO:0030511	6.60E-02	1.20E-01	1	9
6	PCNA|0.404975	error-prone translesion synthesis	GO:0042276	6.60E-02	1.20E-01	1	6
4,6	SNW1|0.473175	positive regulation of cellular response to transforming growth factor beta stimulus	GO:1903846	6.60E-02	1.20E-01	1	6
4	PLEK|2.071125	positive regulation of coagulation	GO:0050820	6.60E-02	1.20E-01	1	4
5	ALDH1L2|2.148;ESD|2.155525;ASL|2.252975	small molecule catabolic process	GO:0044282	6.63E-02	1.20E-01	3	5
6	TTK|0.488375;PLK1|0.415475	mitotic spindle	GO:0007052	6.71E-02	1.20E-01	2	6
5	HSPD1|2.88995;ASPH|2.59965	response to organophosphorus	GO:0046683	6.71E-02	1.22E-01	2	5
3,5	ASPH|2.59965;ERO1L|2.370575	negative regulation of homeostatic process	GO:0032845	6.71E-02	1.22E-01	2	5
4	AKR1B1|2.235325;SLC25A1|2.1121;ISYNA1|0.49065;ACAT2|2.108325;MAPK14|0.483325;PLEK|2.071125;PLBD2|2.666725;ALB|0.23405;PLP1|3.936	lipid metabolic process	GO:0006629	6.78E-02	1.22E-01	9	4
5,6,7	G3BP1|0.3904;PSME3|0.401575;MAPK14|0.483325	regulation of Wnt signaling pathway	GO:0030111	6.81E-02	1.23E-01	3	7
5,6	HSPD1|2.88995	regulation of macrophage activation	GO:0043030	6.85E-02	1.23E-01	1	6
5,6	VAT1|2.702925	mitochondrial fusion	GO:0008053	6.85E-02	1.23E-01	1	6
4,5	ISYNA1|0.49065	inositol metabolic process	GO:0006020	6.85E-02	1.23E-01	1	5
5,7	MAPK14|0.483325	cellular response to virus	GO:0098586	6.85E-02	1.23E-01	1	7
6	PLK1|0.415475	synaptonemal complex organization	GO:0070193	6.85E-02	1.23E-01	1	6
8,9	ASPH|2.59965	regulation of ryanodine-sensitive calcium-release channel activity	GO:0060314	6.85E-02	1.23E-01	1	9
6,7,9	STAT3|0.4995	regulation of neuron migration	GO:2001222	6.85E-02	1.23E-01	1	9
6,7	BAZ1B|0.460625	positive regulation of gene expression, epigenetic	GO:0045815	6.85E-02	1.23E-01	1	7
5,6	MAPK14|0.483325	positive regulation of heart growth	GO:0060421	6.85E-02	1.23E-01	1	6
6,7	GMPS|0.496875	purine nucleobase biosynthetic process	GO:0009113	6.85E-02	1.23E-01	1	7
7,8	G3BP1|0.3904;PSME3|0.401575;MAPK14|0.483325	canonical Wnt signaling pathway	GO:0060070	6.86E-02	1.23E-01	3	8
7,8	HSPA8|0.3721;NSF|2.017425	post-Golgi vesicle-mediated transport	GO:0006892	6.87E-02	1.23E-01	2	8
5,6	TUBG1|0.4102;NDE1|0.446425;TTK|0.488375;PLK1|0.415475;CETN3|0.4171	microtubule cytoskeleton organization	GO:0000226	6.87E-02	1.23E-01	5	6
5	ATP6V1B2|2.0575;AKR1B1|2.235325;MAPK14|0.483325;STAT3|0.4995	response to peptide	GO:1901652	6.88E-02	1.23E-01	4	5
6	HIST1H4A|0.23835;MAPK14|0.483325;TFRC|0.403125	myeloid cell	GO:0030099	6.95E-02	1.23E-01	3	6
5,6	STAT3|0.4995;PLEK|2.071125	regulation of cellular carbohydrate metabolic process	GO:0010675	7.02E-02	1.25E-01	2	6
4,5	TXNIP|2.0553;TTK|0.488375;PLK1|0.415475	regulation of cell division	GO:0051302	7.05E-02	1.25E-01	3	5
8,9	ATP6V1B2|2.0575;TFRC|0.403125	transition metal ion transport	GO:0000041	7.09E-02	1.25E-01	2	9
6,7	GCH1|4.01735;SOD2|2.175425	regulation of blood vessel size	GO:0050880	7.09E-02	1.25E-01	2	7
7,8	HSPD1|2.88995;GSN|2.4555	positive regulation of cysteine-type endopeptidase activity involved in apoptotic process	GO:0043280	7.09E-02	1.25E-01	2	8
9,10	HSPA8|0.3721;PABPC1|0.3285;SNW1|0.473175	RNA splicing, via transesterification reactions with bulged adenosine as nucleophile	GO:0000377	7.10E-02	1.25E-01	3	10
8,9,10,11	HSPA8|0.3721;PABPC1|0.3285;SNW1|0.473175	mRNA splicing, via spliceosome	GO:0000398	7.10E-02	1.25E-01	3	11
7,8	SLC25A1|2.1121	long-chain fatty-acyl-CoA metabolic process	GO:0035336	7.11E-02	1.25E-01	1	8
5,6,7,8	PLK1|0.415475	female meiotic division	GO:0007143	7.11E-02	1.25E-01	1	8
5	VIM|2.241075	lens fiber cell differentiation	GO:0070306	7.11E-02	1.25E-01	1	5

4	CNDP2|2.0938	glutathione derivative metabolic process	GO:1901685	7.11E-02	1.25E-01	1	4
5,8,9	YWHAG|2.359475	positive regulation of protein insertion into mitochondrial membrane involved in apoptotic signaling pathway	GO:1900740	7.11E-02	1.25E-01	1	9
6,7,8	TOP2A|0.481675	positive regulation of viral genome replication	GO:0045070	7.11E-02	1.25E-01	1	8
5,6,7	SOS2|11.48525	regulation of thymocyte aggregation	GO:2000398	7.11E-02	1.25E-01	1	7
5	ALDH1L2|2.148	cofactor catabolic process	GO:0051187	7.11E-02	1.25E-01	1	5
6	SNW1|0.473175	cellular response to	GO:0071295	7.11E-02	1.25E-01	1	6
5	CNDP2|2.0938	glutathione derivative biosynthetic process	GO:1901687	7.11E-02	1.25E-01	1	5
6,7,8,9	PABPC1|0.3285	regulation of mRNA catabolic process	GO:0061013	7.11E-02	1.25E-01	1	9
5,6	ATG7|2.120975	modulation by virus of host morphology or	GO:0019048	7.11E-02	1.25E-01	1	6
5,6,7,8	STAT3|0.4995	positive regulation of Notch signaling pathway	GO:0045747	7.11E-02	1.25E-01	1	8
5	MYH10|5.579275	nucleus localization	GO:0051647	7.11E-02	1.25E-01	1	5
7,8,9,10,12,13	ASPH|2.59965	regulation of release of sequestered calcium ion into cytosol by sarcoplasmic reticulum	GO:0010880	7.11E-02	1.25E-01	1	13
6,7,8	YWHAG|2.359475	regulation of protein insertion into mitochondrial membrane involved in apoptotic	GO:1900739	7.11E-02	1.25E-01	1	8
6,7,8	SOS2|11.48525	regulation of T cell differentiation in thymus	GO:0033081	7.11E-02	1.25E-01	1	8
6,7	LCP1|2.170475	protein kinase A signaling	GO:0010737	7.11E-02	1.25E-01	1	7
6	ASPH|2.59965;ATP6V1B2|2.0575;CKB|0.486625;ERO1L|2.370575;TFRC|0.403125	ion homeostasis	GO:0050801	7.16E-02	1.25E-01	5	6
5	GCH1|4.01735;SOD2|2.175425	regulation of tube size	GO:0035150	7.17E-02	1.26E-01	2	5
6,7	HSPD1|2.88995;GSN|2.4555;TUBG1|0.4102;NDE1|0.446425;TBCA|0.3515	cellular protein complex assembly	GO:0043623	7.19E-02	1.26E-01	5	7
8,9	HSPA8|0.3721;PABPC1|0.3285;SNW1|0.473175	RNA splicing, via transesterification	GO:0000375	7.24E-02	1.27E-01	3	9
4	HSP90AB1|0.458775;LCP1|2.170475;ASPH|2.59965;NSF|2.017425;VDAC3|2.015475;YWHAG|2.359475;ATG7|2.120975;MAPK14|0.483325;SLC2A1|5.081875;VTN|2.01125	regulation of transport	GO:0051049	7.24E-02	1.27E-01	10	4
6,7	AKR1B1|2.235325;STAT3|0.4995	JAK-STAT cascade	GO:0007259	7.25E-02	1.27E-01	2	7
3	LCP1|2.170475;HSPD1|2.88995;GSN|2.4555;NDRG1|10.0073;SOS2|11.48525	leukocyte activation	GO:0045321	7.25E-02	1.27E-01	5	3
7,8	HSP90AB1|0.458775;YWHAG|2.359475;LAMTOR2|7.841025;SQSTM1|6.007525;MAPK14|0.483325;VTN|2.01125;TTK|0.488375;PLK1|0.415475	regulation of protein phosphorylation	GO:0001932	7.25E-02	1.27E-01	8	8
3,4	GSN|2.4555;EIF4H|0.275675;MAPK14|0.483325;STAT3|0.4995	developmental growth	GO:0048589	7.30E-02	1.27E-01	4	4
4	MAPK14|0.483325;TXNIP|2.0553	cellular response to biotic stimulus	GO:0071216	7.33E-02	1.28E-01	2	4
5,6,7,8	PLEK|2.071125	regulation of platelet activation	GO:0010543	7.36E-02	1.28E-01	1	8
6,8,9	LIG1|0.440425	double-strand break repair via nonhomologous end joining	GO:0006303	7.36E-02	1.28E-01	1	9
4,7	PTP4A3|0.3433	regulation of vascular permeability	GO:0043114	7.36E-02	1.28E-01	1	7
5	TFRC|0.403125	response to copper ion	GO:0046688	7.36E-02	1.28E-01	1	5
4	SRRT|0.39875	response to arsenic-containing substance	GO:0046685	7.36E-02	1.28E-01	1	4
5,6	PRDX5|3.0143	negative regulation of oxidoreductase activity	GO:0051354	7.36E-02	1.28E-01	1	6
5	HSP90AB1|0.458775	response to salt stress	GO:0009651	7.36E-02	1.28E-01	1	5
6	STAT3|0.4995	response to interleukin-6	GO:0070741	7.36E-02	1.28E-01	1	6
7,13	ATG7|2.120975	histone H4-K16	GO:0043984	7.36E-02	1.28E-01	1	13
5	ANXA5|3.637725;SQSTM1|6.007525;ATG7|2.120975;CHAF1B|0.2723	autophagy	GO:0006914	7.38E-02	1.28E-01	4	5
5,6	HSPD1|2.88995;PSME3|0.401575;MAPK14|0.483325	positive regulation of innate immune response	GO:0045089	7.38E-02	1.28E-01	3	6
7	ACTG1|2.1999;HSP90AB1|0.458775;ACTB|2.1999	Fc receptor signaling pathway	GO:0038093	7.38E-02	1.28E-01	3	7
6,7,8,9	HSP90AB1|0.458775;MAPK14|0.483325	regulation of protein import into nucleus	GO:0042306	7.40E-02	1.28E-01	2	9
5	VIM|2.241075;STAT3|0.4995;MYH10|5.579275	eye development	GO:0001654	7.43E-02	1.29E-01	3	5
8,9,10	PSME3|0.401575;PLK1|0.415475	positive regulation of protein ubiquitination	GO:0031398	7.48E-02	1.29E-01	2	10
6,7	GMPS|0.496875;STAT3|0.4995	nucleoside monophosphate	GO:0009124	7.48E-02	1.30E-01	2	7
8,9,10	HSP90AB1|0.458775;YWHAG|2.359475;MAPK14|0.483325;PLK1|0.415475	regulation of protein serine/threonine kinase activity	GO:0071900	7.49E-02	1.30E-01	4	10
5	LCP1|2.170475;PLEK|2.071125	actin filament bundle assembly	GO:0051017	7.56E-02	1.30E-01	2	5

5	MAPK14|0.483325	regulation of syncytium formation by plasma membrane fusion	GO:0060142	7.61E-02	1.31E-01	1	5
7	ASPH|2.59965	protein hydroxylation	GO:0018126	7.61E-02	1.31E-01	1	7
6,7,8,9	KRT1|0.4198	fibrinolysis	GO:0042730	7.61E-02	1.31E-01	1	9
5	MYH10|5.579275	substrate-dependent cell migration	GO:0006929	7.61E-02	1.31E-01	1	5
7,9	ASL|2.252975	glutamine family amino acid catabolic process	GO:0009065	7.61E-02	1.31E-01	1	9
6,7	PRDX5|3.0143	homeostasis of number of cells within a tissue	GO:0048873	7.61E-02	1.31E-01	1	7
5,6	HSPA8|0.3721	regulation of extracellular matrix organization	GO:1903053	7.61E-02	1.31E-01	1	6
6,7	PLEK|2.071125	protein kinase C signaling	GO:0070528	7.61E-02	1.31E-01	1	7
6,7,8,10	GSN|2.4555	regulation of cysteine-type endopeptidase activity involved in apoptotic signaling pathway	GO:2001267	7.61E-02	1.31E-01	1	10
5,6,7,8,9	YWHAG|2.359475	protein insertion into mitochondrial membrane involved in apoptotic signaling pathway	GO:0001844	7.61E-02	1.31E-01	1	9
5	STAT3|0.4995;PLEK|2.071125	regulation of carbohydrate metabolic process	GO:0006109	7.64E-02	1.31E-01	2	5
7	HSPD1|2.88995;ASPH|2.59965	zymogen activation	GO:0031638	7.64E-02	1.31E-01	2	7
4,6,7	HSPA8|0.3721;NSF|2.017425;MAPK14|0.483325;SLC2A1|5.081875;PLEK|2.071125;MYH10|5.579275;ALB|0.23405	secretion by cell	GO:0032940	7.64E-02	1.31E-01	7	7
7	LCP1|2.170475;PLEK|2.071125	actin filament bundle organization	GO:0061572	7.72E-02	1.31E-01	2	7
5	PCNA|0.404975;MAPK14|0.483325	cellular response to radiation	GO:0071478	7.80E-02	1.33E-01	2	5
5	HSPD1|2.88995;ASPH|2.59965	response to purine-containing compound	GO:0014074	7.80E-02	1.33E-01	2	5
6,8,10	VIM|2.241075;ARHGDIA|3.8215	negative regulation of neuron differentiation	GO:0045665	7.80E-02	1.33E-01	2	10
7	MAPK14|0.483325;SNW1|0.473175	skeletal muscle tissue development	GO:0007519	7.80E-02	1.33E-01	2	7
6,7	HBA1|2.13735	oxygen transport	GO:0015671	7.87E-02	1.33E-01	1	7
4,5,6,7,8	YWHAG|2.359475	protein insertion into mitochondrial membrane	GO:0051204	7.87E-02	1.33E-01	1	8
6,7	NDE1|0.446425	mitotic centrosome separation	GO:0007100	7.87E-02	1.33E-01	1	7
6,7	CNDP2|2.0938	glutathione biosynthetic process	GO:0006750	7.87E-02	1.33E-01	1	7
4,5	ALB|0.23405	disruption of cells of other organism	GO:0044364	7.87E-02	1.33E-01	1	5
6	PRDX5|3.0143	regulation of collagen biosynthetic process	GO:0032965	7.87E-02	1.33E-01	1	6
3	ALB|0.23405	killing of cells of other organism	GO:0031640	7.87E-02	1.33E-01	1	3
4,5	HSPD1|2.88995	positive regulation of response to biotic stimulus	GO:0002833	7.87E-02	1.33E-01	1	5
5	ATG7|2.120975	response to increased oxygen levels	GO:0036296	7.87E-02	1.33E-01	1	5
4	STAT3|0.4995	eating behavior	GO:0042755	7.87E-02	1.33E-01	1	4
6,7	NDE1|0.446425	establishment of mitotic spindle orientation	GO:0000132	7.87E-02	1.33E-01	1	7
5,6,7	SOD2|2.175425	negative regulation of oxidative stress-induced cell death	GO:1903202	7.87E-02	1.33E-01	1	7
6	SMC2|0.485025	kinetochore organization	GO:0051383	7.87E-02	1.33E-01	1	6
6	MAPK14|0.483325	positive regulation of cardiac muscle tissue development	GO:0055025	7.87E-02	1.33E-01	1	6
4	ATG7|2.120975	response to hyperoxia	GO:0055093	7.87E-02	1.33E-01	1	4
6,7,8,9	LAMTOR2|7.841025	positive regulation of TOR signaling	GO:0032008	7.87E-02	1.33E-01	1	9
4,6	NSF|2.017425	receptor recycling	GO:0001881	7.87E-02	1.33E-01	1	6
5,6,7,9,10	PLK1|0.415475	negative regulation of cyclin-dependent protein serine/threonine kinase activity	GO:0045736	7.87E-02	1.33E-01	1	10
5	HSP90AB1|0.458775;PLEK|2.071125	regulation of cell size	GO:0008361	7.88E-02	1.33E-01	2	5
5,6,8,9	DNMT1|0.172;SNW1|0.473175	histone methylation	GO:0016571	7.88E-02	1.33E-01	2	9
9	ATP6V1B2|2.0575;TFRC|0.403125	transition metal ion homeostasis	GO:0055076	8.03E-02	1.33E-01	2	9
4,5	HSPA8|0.3721;PSME3|0.401575;TOP2A|0.481675;NDRG1|10.0073;TTK|0.488375;CDC123|0.29925;PLK1|0.415475	regulation of cell cycle	GO:0051726	8.11E-02	1.36E-01	7	5
7,9,10	HSPD1|2.88995;MAPK14|0.483325	toll-like receptor signaling pathway	GO:0002224	8.11E-02	1.36E-01	2	10
5	GCH1|4.01735	dopamine metabolic process	GO:0042417	8.12E-02	1.36E-01	1	5
5	MYH10|5.579275	ventricular system development	GO:0021591	8.12E-02	1.36E-01	1	5
8,9	PLK1|0.415475	regulation of peptidyl-threonine phosphorylation	GO:0010799	8.12E-02	1.36E-01	1	9

6,7	RHOG|2.449775	positive regulation of establishment of protein localization to plasma membrane	GO:0090004	8.12E-02	1.36E-01	1	7
8,9	RHOG|2.449775	Rac protein signal transduction	GO:0016601	8.12E-02	1.36E-01	1	9
5,6	PLEK|2.071125	positive regulation of protein complex disassembly	GO:0043243	8.12E-02	1.36E-01	1	6
6,7	GSN|2.4555	phagocytosis, engulfment	GO:0006911	8.12E-02	1.36E-01	1	7
5	NDE1|0.446425	centrosome separation	GO:0051299	8.12E-02	1.36E-01	1	5
7,8	MAPK14|0.483325	positive regulation of myotube differentiation	GO:0010831	8.12E-02	1.36E-01	1	8
4	HSPD1|2.88995;NDRG1|10.0073	myeloid leukocyte activation	GO:0002274	8.19E-02	1.36E-01	2	4
8	MAPK14|0.483325;TXNIP|2.0553	response to glucose	GO:0009749	8.19E-02	1.37E-01	2	8
6,7,8	PABPC1|0.3285;EIF4H|0.275675;ZNF706|0.3974	regulation of translation	GO:0006417	8.23E-02	1.37E-01	3	8
6	SRRT|0.39875;STAT3|0.4995;ZNF706|0.3974	stem cell differentiation	GO:0048863	8.33E-02	1.38E-01	3	6
6,7,8	STAT3|0.4995	regulation of glycolytic process	GO:0006110	8.37E-02	1.39E-01	1	8
6	ASPH|2.59965	calcium ion transport from endoplasmic reticulum to cytosol	GO:1903514	8.37E-02	1.39E-01	1	6
8	STAT3|0.4995	growth hormone receptor signaling pathway	GO:0060396	8.37E-02	1.39E-01	1	8
5	MAPK14|0.483325	regulation of cardiac muscle cell proliferation	GO:0060043	8.37E-02	1.39E-01	1	5
5,6	MAPK14|0.483325	skeletal muscle tissue regeneration	GO:0043403	8.37E-02	1.39E-01	1	6
4	SOD2|2.175425	negative regulation of response to oxidative	GO:1902883	8.37E-02	1.39E-01	1	4
4	PRDX5|3.0143	positive regulation of multicellular organismal metabolic process	GO:0044253	8.37E-02	1.39E-01	1	4
5,6,7	VTN|2.01125	regulation of vascular endothelial growth factor receptor signaling	GO:0030947	8.37E-02	1.39E-01	1	7
4	AKR1B1|2.235325	C21-steroid hormone metabolic process	GO:0008207	8.37E-02	1.39E-01	1	4
6,7,8	SOD2|2.175425	regulation of oxidative stress-induced intrinsic apoptotic signaling	GO:1902175	8.37E-02	1.39E-01	1	8
4,5,6	SOD2|2.175425	negative regulation of cellular response to oxidative stress	GO:1900408	8.37E-02	1.39E-01	1	6
7,8	CNDP2|2.0938	nonribosomal peptide biosynthetic process	GO:0019184	8.37E-02	1.39E-01	1	8
6,7,8	ASPH|2.59965	release of sequestered calcium ion into cytosol by sarcoplasmic reticulum	GO:0014808	8.37E-02	1.39E-01	1	8
8	ASPH|2.59965;ATP6V1B2|2.0575;ERO1L|2.370575;TFRC|0.403125	metal ion homeostasis	GO:0055065	8.47E-02	1.39E-01	4	8
7,8,9	PSME3|0.401575;PLK1|0.415475	positive regulation of protein modification by small protein conjugation or removal	GO:1903322	8.52E-02	1.41E-01	2	9
5	HIST1H4A|0.23835;MAPK14|0.483325	regulation of myeloid cell differentiation	GO:0045637	8.60E-02	1.41E-01	2	5
4,5,6	VDAC3|2.015475;RHOG|2.449775	regulation of cell projection assembly	GO:0060491	8.60E-02	1.42E-01	2	6
6,7	SLC25A1|2.1121	fatty-acyl-CoA metabolic process	GO:0035337	8.62E-02	1.42E-01	1	7
3	ALB|0.23405	cytolysis	GO:0019835	8.62E-02	1.42E-01	1	3
6,8,9,10,11	ATG7|2.120975	negative regulation of histone acetylation	GO:0035067	8.62E-02	1.42E-01	1	11
5,6,7,8	BAZ1B|0.460625	positive regulation of receptor activity	GO:2000273	8.62E-02	1.42E-01	1	8
6,7,8	SLC2A1|5.081875	cellular response to glucose starvation	GO:0042149	8.62E-02	1.42E-01	1	8
6,8,10,11	SRRT|0.39875	production of miRNAs involved in gene silencing by miRNA	GO:0035196	8.62E-02	1.42E-01	1	11
4,8	SOD2|2.175425	removal of superoxide radicals	GO:0019430	8.62E-02	1.42E-01	1	8
4,5,6,7	TFRC|0.403125	regulation of bone resorption	GO:0045124	8.62E-02	1.42E-01	1	7
7	STAT3|0.4995	cellular response to growth hormone stimulus	GO:0071378	8.62E-02	1.42E-01	1	7
4	GSN|2.4555;MAPK14|0.483325	regeneration	GO:0031099	8.68E-02	1.42E-01	2	4
7	MAPK14|0.483325;TXNIP|2.0553	response to hexose	GO:0009746	8.68E-02	1.43E-01	2	7
6,7	SNW1|0.473175;STAT3|0.4995	Notch signaling pathway	GO:0007219	8.68E-02	1.43E-01	2	7
6,7,8	TTK|0.488375;PLK1|0.415475	regulation of mitotic nuclear division	GO:0007088	8.68E-02	1.43E-01	2	8
7,8	ASPH|2.59965;BAZ1B|0.460625;SQSTM1|6.007525;MAPK14|0.483325;RHOG|2.449775;TOP2A|0.481675;SNW1|0.473175;STAT3|0.4995	positive regulation of nucleic acid-templated transcription	GO:1903508	8.72E-02	1.43E-01	8	8

6,7,8,9	ASPH|2.59965;BAZ1B|0.460625;SQSTM1|6.007525;MAPK14|0.483325;RHOG|2.449775;TOP2A|0.481675;SNW1|0.473175;STAT3|0.4995	positive regulation of transcription, DNA-templated	GO:0045893	8.72E-02	1.43E-01	8	9
3	ACTG1|2.1999;KRT1|0.4198;MTHFD1L|6.667175;VDAC3|2.015475;MAPK14|0.483325;VTN|2.01125;MYH10|5.579275	anatomical structure formation involved in morphogenesis	GO:0048646	8.73E-02	1.43E-01	7	3
6	VIM|2.241075;ALDOC|2.04725;PCNA|0.404975;TXNIP|2.0553	epithelial cell differentiation	GO:0030855	8.75E-02	1.43E-01	4	6
4,5,6,7	ASPH|2.59965;ERO1L|2.370575	regulation of ion homeostasis	GO:2000021	8.76E-02	1.44E-01	2	7
5	HSP90AB1|0.458775;NSF|2.017425;PSME3|0.401575;SQSTM1|6.007525;ATG7|2.120975;TTK|0.488375;PLK1|0.41	protein catabolic process	GO:0030163	8.80E-02	1.44E-01	7	5
6	ACTG1|2.1999;HSP90AB1|0.458775;PSME3|0.401575;ACTB|2.1999	immune response-regulating cell surface receptor signaling	GO:0002768	8.83E-02	1.44E-01	4	6
7,8	ASPH|2.59965;ATP6V1B2|2.0575;ERO1L|2.370575;TFRC|0.403125	cellular cation homeostasis	GO:0030003	8.87E-02	1.44E-01	4	8
7,8	ATG7|2.120975	forebrain neuron development	GO:0021884	8.87E-02	1.44E-01	1	8
6	SNW1|0.473175	response to vitamin D	GO:0033280	8.87E-02	1.44E-01	1	6
5	SRRT|0.39875	production of small RNA involved in gene silencing by RNA	GO:0070918	8.87E-02	1.44E-01	1	5
7	HSPD1|2.88995	positive regulation of T cell mediated immunity	GO:0002711	8.87E-02	1.44E-01	1	7
6	NDE1|0.446425	establishment of mitotic spindle localization	GO:0040001	8.87E-02	1.44E-01	1	6
5	DNMT1|0.172	histone H3-K9	GO:0061647	8.87E-02	1.44E-01	1	5
4	SRRT|0.39875	dsRNA fragmentation	GO:0031050	8.87E-02	1.44E-01	1	4
5,6	PRDX5|3.0143	regulation of collagen metabolic process	GO:0010712	8.87E-02	1.44E-01	1	6
5,6	HBA1|2.13735	gas transport	GO:0015669	8.87E-02	1.44E-01	1	6
5,6,7,8,9	YWHAG|2.359475	positive regulation of mitochondrial outer membrane permeabilization involved in apoptotic signaling	GO:1901030	8.87E-02	1.44E-01	1	9
7	PLK1|0.415475	G2 DNA damage checkpoint	GO:0031572	8.87E-02	1.44E-01	1	7
6	ATG7|2.120975	negative regulation of autophagy	GO:0010507	8.87E-02	1.44E-01	1	6
6	NDE1|0.446425	establishment of spindle orientation	GO:0051294	8.87E-02	1.44E-01	1	6
5,6	RHOG|2.449775	positive regulation of protein localization to plasma membrane	GO:1903078	8.87E-02	1.44E-01	1	6
4,7	RHOG|2.449775	positive regulation of protein localization to cell periphery	GO:1904377	8.87E-02	1.44E-01	1	7
6,7,8	HSP90AB1|0.458775;MAPK14|0.483325	regulation of protein localization to nucleus	GO:1900180	8.93E-02	1.44E-01	2	8
6	MAPK14|0.483325;SNW1|0.473175	skeletal muscle organ development	GO:0060538	8.93E-02	1.45E-01	2	6
6,7	ACTG1|2.1999;MYH10|5.579275	actomyosin structure organization	GO:0031032	9.01E-02	1.45E-01	2	7
7	GMPS|0.496875;STAT3|0.4995	purine nucleoside biosynthetic process	GO:0042451	9.01E-02	1.46E-01	2	7
8	GMPS|0.496875;STAT3|0.4995	purine ribonucleoside biosynthetic process	GO:0046129	9.01E-02	1.46E-01	2	8
6	MAPK14|0.483325;TXNIP|2.0553	response to monosaccharide	GO:0034284	9.09E-02	1.46E-01	2	6
4,7	GCH1|4.01735;SOD2|2.175425	regulation of blood	GO:0008217	9.09E-02	1.47E-01	2	7
7	NDRG1|10.0073	myelination in peripheral nervous system	GO:0022011	9.12E-02	1.47E-01	1	7
6	NDRG1|10.0073	regulation of cell cycle checkpoint	GO:1901976	9.12E-02	1.47E-01	1	6
6,7	ALB|0.23405	bile acid and bile salt transport	GO:0015721	9.12E-02	1.47E-01	1	7
4,6	PCNA|0.404975	DNA damage response, detection of DNA damage	GO:0042769	9.12E-02	1.47E-01	1	6
6	NDRG1|10.0073	peripheral nervous system axon ensheathment	GO:0032292	9.12E-02	1.47E-01	1	6
5	GCH1|4.01735	response to pain	GO:0048265	9.12E-02	1.47E-01	1	5
6,7	ASPH|2.59965;BAZ1B|0.460625;SQSTM1|6.007525;MAPK14|0.483325;RHOG|2.449775;TOP2A|0.481675;SNW1|0.473175;STAT3|0.4995	positive regulation of RNA biosynthetic process	GO:1902680	9.35E-02	1.47E-01	8	7
5	NDE1|0.446425	vesicle transport along microtubule	GO:0047496	9.36E-02	1.50E-01	1	5
7,8	ATG7|2.120975	central nervous system neuron axonogenesis	GO:0021955	9.36E-02	1.50E-01	1	8
6,7	ASPH|2.59965	sarcoplasmic reticulum calcium ion transport	GO:0070296	9.36E-02	1.50E-01	1	7
4,5,6	LCP1|2.170475	positive regulation of organelle assembly	GO:1902117	9.36E-02	1.50E-01	1	6
5	NDE1|0.446425	centrosome localization	GO:0051642	9.36E-02	1.50E-01	1	5
6	SOD2|2.175425	cellular response to oxygen radical	GO:0071450	9.36E-02	1.50E-01	1	6

7	PLK1|0.415475	protein localization to chromosome	GO:0034502	9.36E-02	1.50E-01	1	7
8,9,10	ATG7|2.120975	negative regulation of peptidyl-lysine acetylation	GO:2000757	9.36E-02	1.50E-01	1	10
7	SOD2|2.175425	cellular response to superoxide	GO:0071451	9.36E-02	1.50E-01	1	7
9,10	GSN|2.4555	barbed-end actin filament capping	GO:0051016	9.36E-02	1.50E-01	1	10
5	PRDX5|3.0143	collagen biosynthetic process	GO:0032964	9.36E-02	1.50E-01	1	5
5	ALB|0.23405	mitochondrion	GO:0051646	9.36E-02	1.50E-01	1	5
5,6	PABPC1|0.3285;EIF4H|0.275675;ZNF706|0.3974	regulation of cellular amide metabolic process	GO:0034248	9.37E-02	1.50E-01	3	6
6,7	LIG1|0.440425;PCNA|0.404975	DNA biosynthetic process	GO:0071897	9.43E-02	1.50E-01	2	7
5	HSPD1|2.88995;GSN|2.4555;SOS2|11.48525	regulation of cell-cell adhesion	GO:0022407	9.43E-02	1.50E-01	3	5
5	NSF|2.017425;PLEK|2.071125	vesicle docking	GO:0048278	9.51E-02	1.50E-01	2	5
4,5	ATG7|2.120975;NDE1|0.446425	pallium development	GO:0021543	9.60E-02	1.52E-01	2	5
4	AKR1B1|2.235325;GCH1|4.01735	ammonium ion metabolic process	GO:0097164	9.60E-02	1.53E-01	2	4
5,6	HSP90AB1|0.458775	positive regulation of nitric oxide metabolic	GO:1904407	9.61E-02	1.53E-01	1	6
6,7	VTN|2.01125	regulation of smooth muscle cell migration	GO:0014910	9.61E-02	1.53E-01	1	7
6,7	STAT3|0.4995	regulation of nucleotide catabolic process	GO:0030811	9.61E-02	1.53E-01	1	7
4,5,6	MAPK14|0.483325	regulation of erythrocyte differentiation	GO:0045646	9.61E-02	1.53E-01	1	6
6,7	HSP90AB1|0.458775	positive regulation of nitric oxide biosynthetic	GO:0045429	9.61E-02	1.53E-01	1	7
9,10	PABPC1|0.3285	nuclear-transcribed mRNA poly(A) tail	GO:0000289	9.61E-02	1.53E-01	1	10
4	ATG7|2.120975;NDE1|0.446425;MYH10|5.579275	forebrain development	GO:0030900	9.70E-02	1.53E-01	3	4
5	PSME3|0.401575;NDRG1|10.0073;TTK|0.488375;PLK1|0.415475	regulation of cell cycle process	GO:0010564	9.72E-02	1.54E-01	4	5
8,9	MAPK14|0.483325;SLC2A1|5.081875	glucose transport	GO:0015758	9.76E-02	1.54E-01	2	9
5	MYH10|5.579275;PLK1|0.415475	cytokinesis	GO:0000910	9.85E-02	1.55E-01	2	5
5,6,7,8	YWHAG|2.359475;GSN|2.4555	positive regulation of apoptotic signaling	GO:2001235	9.85E-02	1.55E-01	2	8
4,5	KRT1|0.4198;VTN|2.01125	complement activation	GO:0006956	9.85E-02	1.55E-01	2	5
5,6,7	MAPK14|0.483325	regulation of cardiac muscle tissue growth	GO:0055021	9.86E-02	1.55E-01	1	7
6,7	VAT1|2.702925	negative regulation of mitochondrion	GO:0010823	9.86E-02	1.55E-01	1	7
5,7,8	LIG1|0.440425	non-recombinational	GO:0000726	9.86E-02	1.55E-01	1	8
8,9	PTP4A3|0.3433	vascular endothelial growth factor signaling	GO:0038084	9.86E-02	1.55E-01	1	9
5,6	STAT3|0.4995	temperature homeostasis	GO:0001659	9.86E-02	1.55E-01	1	6
4,5,6	MAPK14|0.483325	positive regulation of organ growth	GO:0046622	9.86E-02	1.55E-01	1	6
5	ATG7|2.120975	adult walking behavior	GO:0007628	9.86E-02	1.55E-01	1	5
7,8,9	ATG7|2.120975	negative regulation of protein acetylation	GO:1901984	9.86E-02	1.55E-01	1	9
5,6	TFRC|0.403125	regulation of bone remodeling	GO:0046850	9.86E-02	1.55E-01	1	6
5,7,10,11	MAPK14|0.483325	positive regulation of glucose import	GO:0046326	9.86E-02	1.55E-01	1	11
4	ENO1|2.1939;ALDOC|2.04725;STAT3|0.4995;SLC2A1|5.081875	generation of precursor metabolites and energy	GO:0006091	9.93E-02	1.55E-01	4	4
7,8	SARS|2.2366;YARS|2.288275;NARS|2.162	tRNA metabolic process	GO:0006399	1.00E-01	1.56E-01	3	8
7,8	VIM|2.241075	muscle filament sliding	GO:0030049	1.01E-01	1.57E-01	1	8
7	CCT7|2.06135	binding of sperm to zona pellucida	GO:0007339	1.01E-01	1.57E-01	1	7
7,8	PRDX5|3.0143	regulation of transcription from RNA polymerase III promoter	GO:0006359	1.01E-01	1.57E-01	1	8
5,7,8	VTN|2.01125	regulation of complement activation	GO:0030449	1.01E-01	1.57E-01	1	8
6	TXNIP|2.0553	response to progesterone	GO:0032570	1.01E-01	1.57E-01	1	6
4,7	GSN|2.4555	striated muscle adaptation	GO:0014888	1.01E-01	1.57E-01	1	7
7	VIM|2.241075	actin-myosin filament sliding	GO:0033275	1.01E-01	1.57E-01	1	7
6,7	ASPH|2.59965	positive regulation of calcium ion transmembrane transporter activity	GO:1901021	1.01E-01	1.57E-01	1	7
7	HIST1H4A|0.23835	megakaryocyte differentiation	GO:0030219	1.01E-01	1.57E-01	1	7
4	MAPK14|0.483325	production of molecular mediator involved in inflammatory response	GO:0002532	1.01E-01	1.57E-01	1	4
6,7,8,9	HSP90AB1|0.458775	regulation of type I interferon-mediated signaling pathway	GO:0060338	1.01E-01	1.57E-01	1	9
6	SOD2|2.175425	response to superoxide	GO:0000303	1.01E-01	1.57E-01	1	6
6,7	ASPH|2.59965	positive regulation of calcium ion transmembrane transport	GO:1904427	1.01E-01	1.57E-01	1	7

6,7,8	NDRG1|10.0073	Schwann cell	GO:0014044	1.01E-01	1.57E-01	1	8
5,6,7	HSPD1|2.88995;GSN|2.4555;SOS2|11.48525	regulation of lymphocyte activation	GO:0051249	1.02E-01	1.57E-01	3	7
9	ATG7|2.120975;ASL|2.252975	internal protein amino acid acetylation	GO:0006475	1.03E-01	1.59E-01	2	9
5	STAT3|0.4995;ARHGDIA|3.8215	negative regulation of cell migration	GO:0030336	1.03E-01	1.60E-01	2	5
6	ERO1L|2.370575;MAPK14|0.483325	fat cell differentiation	GO:0045444	1.03E-01	1.60E-01	2	6
6,9	TOP2A|0.481675	apoptotic nuclear changes	GO:0030262	1.03E-01	1.60E-01	1	9
6,7,8,9	SOS2|11.48525	positive regulation of small GTPase mediated signal transduction	GO:0051057	1.03E-01	1.60E-01	1	9
7,8	RHOG|2.449775	activation of GTPase activity	GO:0090630	1.03E-01	1.60E-01	1	8
6,7	TOP2A|0.481675	DNA topological change	GO:0006265	1.03E-01	1.60E-01	1	7
5	SOD2|2.175425	response to oxygen radical	GO:0000305	1.03E-01	1.60E-01	1	5
5,6	HSPD1|2.88995	regulation of interleukin-10 production	GO:0032653	1.03E-01	1.60E-01	1	6
6,7	HIST1H4A|0.23835	histone lysine demethylation	GO:0070076	1.03E-01	1.60E-01	1	7
6,7	PLEK|2.071125	cortical actin cytoskeleton organization	GO:0030866	1.03E-01	1.60E-01	1	7
7	ASL|2.252975	arginine metabolic process	GO:0006525	1.03E-01	1.60E-01	1	7
5,6	GMPS|0.496875	nucleobase biosynthetic process	GO:0046112	1.03E-01	1.60E-01	1	6
7,8	PSME3|0.401575;PLK1|0.415475	positive regulation of proteolysis involved in cellular protein catabolic process	GO:1903052	1.04E-01	1.60E-01	2	8
7,8	HSP90AB1|0.458775;MAPK14|0.483325	regulation of nucleocytoplasmic transport	GO:0046822	1.04E-01	1.60E-01	2	8
7,8	MAPK14|0.483325;SLC2A1|5.081875	hexose transport	GO:0008645	1.05E-01	1.61E-01	2	8
7	MAPK14|0.483325;TFRC|0.403125	myeloid leukocyte differentiation	GO:0002573	1.05E-01	1.63E-01	2	7
6,7,8,9	YWHAG|2.359475	regulation of mitochondrial outer membrane permeabilization involved	GO:1901028	1.06E-01	1.63E-01	1	9
5	SNW1|0.473175	cellular response to	GO:0031670	1.06E-01	1.63E-01	1	5
4	ASPH|2.59965	face morphogenesis	GO:0060325	1.06E-01	1.63E-01	1	4
5	ZNF706|0.3974	negative regulation of stem cell differentiation	GO:2000737	1.06E-01	1.63E-01	1	5
5,6,7,9	SRRT|0.39875;SNW1|0.473175;ARHGDIA|3.8215	positive regulation of neurogenesis	GO:0050769	1.07E-01	1.63E-01	3	9
7,8	YWHAG|2.359475;STAT3|0.4995;PLK1|0.415475	negative regulation of phosphorylation	GO:0042326	1.07E-01	1.65E-01	3	8
4,6,7	HSP90AB1|0.458775;ASPH|2.59965;MAPK14|0.483325	positive regulation of protein transport	GO:0051222	1.08E-01	1.65E-01	3	7
7	GMPS|0.496875;STAT3|0.4995	ribonucleoside biosynthetic process	GO:0042455	1.08E-01	1.65E-01	2	7
5,6	HIST1H4A|0.23835	histone demethylation	GO:0016577	1.08E-01	1.65E-01	1	6
6,7	SLC25A1|2.1121	acyl-CoA biosynthetic process	GO:0071616	1.08E-01	1.65E-01	1	7
5	SLC25A1|2.1121	thioester biosynthetic process	GO:0035384	1.08E-01	1.65E-01	1	5
6	STAT3|0.4995	response to growth hormone	GO:0060416	1.08E-01	1.65E-01	1	6
5,6,7	MAPK14|0.483325	cardiac muscle cell proliferation	GO:0060038	1.08E-01	1.65E-01	1	7
4,6	VTN|2.01125	regulation of protein activation cascade	GO:2000257	1.08E-01	1.65E-01	1	6
6	PLK1|0.415475	mitotic nuclear envelope disassembly	GO:0007077	1.08E-01	1.65E-01	1	6
6,7	HSP90AB1|0.458775	protein import into nucleus, translocation	GO:0000060	1.08E-01	1.65E-01	1	7
5	CCT7|2.06135	toxin transport	GO:1901998	1.08E-01	1.65E-01	1	5
5,6	GCH1|4.01735	positive regulation of oxidoreductase activity	GO:0051353	1.08E-01	1.65E-01	1	6
4,6,9,10	MAPK14|0.483325	positive regulation of glucose transport	GO:0010828	1.08E-01	1.65E-01	1	10
6,8,9	HSPD1|2.88995;MAPK14|0.483325	pattern recognition receptor signaling	GO:0002221	1.10E-01	1.65E-01	2	9
4	NSF|2.017425;PLEK|2.071125	membrane docking	GO:0022406	1.10E-01	1.67E-01	2	4
6,7	MAPK14|0.483325;SLC2A1|5.081875	monosaccharide transport	GO:0015749	1.10E-01	1.67E-01	2	7
5	MAPK14|0.483325;TXNIP|2.0553	response to carbohydrate	GO:0009743	1.11E-01	1.67E-01	2	5
6,7,8	ACTG1|2.1999	sarcomere organization	GO:0045214	1.11E-01	1.68E-01	1	8
4	PRDX5|3.0143	regulation of multicellular organismal metabolic process	GO:0044246	1.11E-01	1.68E-01	1	4
5	NDE1|0.446425	establishment of spindle localization	GO:0051293	1.11E-01	1.68E-01	1	5
5,6	STAT3|0.4995	regulation of carbohydrate catabolic process	GO:0043470	1.11E-01	1.68E-01	1	6
5,6,7	STAT3|0.4995	negative regulation of reactive oxygen species metabolic process	GO:2000378	1.11E-01	1.68E-01	1	7
5	HSPD1|2.88995	interleukin-10 production	GO:0032613	1.11E-01	1.68E-01	1	5

5,6	STAT3|0.4995	regulation of cellular carbohydrate catabolic process	GO:0043471	1.11E-01	1.68E-01	1	6
6	GCH1|4.01735	phenol-containing compound biosynthetic process	GO:0046189	1.11E-01	1.68E-01	1	6
6	VTN|2.01125	smooth muscle cell migration	GO:0014909	1.11E-01	1.68E-01	1	6
5	SOS2|11.48525	B cell homeostasis	GO:0001782	1.11E-01	1.68E-01	1	5
6	RHOG|2.449775	ruffle assembly	GO:0097178	1.11E-01	1.68E-01	1	6
5	HSP90AB1|0.458775	positive regulation of reactive oxygen species biosynthetic process	GO:1903428	1.11E-01	1.68E-01	1	5
4,5	AKR1B1|2.235325	alditol metabolic process	GO:0019400	1.11E-01	1.68E-01	1	5
5,7	PCNA|0.404975	positive regulation of DNA repair	GO:0045739	1.11E-01	1.68E-01	1	7
7,8	GSN|2.4555;TUBG1|0.4102;NDE1|0.446425	protein polymerization	GO:0051258	1.11E-01	1.68E-01	3	8
4	STAT3|0.4995;ARHGDIA|3.8215	negative regulation of cell motility	GO:2000146	1.11E-01	1.68E-01	2	4
4,6	ARHGDIA|3.8215;MYH10|5.579275	regulation of cell shape	GO:0008360	1.11E-01	1.68E-01	2	6
5	VIM|2.241075;ASPH|2.59965;GSN|2.4555	muscle system process	GO:0003012	1.12E-01	1.68E-01	3	5
6	ATP6V1B2|2.0575;AKR1B1|2.235325;STAT3|0.4995	cellular response to	GO:1901653	1.13E-01	1.69E-01	3	6
4,5,7,8	HIST1H3A|0.455625	regulation of gene	GO:0060968	1.13E-01	1.70E-01	1	8
7	HIST1H4A|0.23835	protein dealkylation	GO:0008214	1.13E-01	1.70E-01	1	7
4	HSPD1|2.88995	response to cold	GO:0009409	1.13E-01	1.70E-01	1	4
4,5	HIST1H4A|0.23835	protein demethylation	GO:0006482	1.13E-01	1.70E-01	1	5
7	ASPH|2.59965	cellular response to calcium ion	GO:0071277	1.13E-01	1.70E-01	1	7
6,7	PLK1|0.415475	nuclear envelope disassembly	GO:0051081	1.13E-01	1.70E-01	1	7
4,6,7,8	ASPH|2.59965	positive regulation of calcium ion transport into cytosol	GO:0010524	1.13E-01	1.70E-01	1	8
5,7	PLEK|2.071125	regulation of alcohol biosynthetic process	GO:1902930	1.13E-01	1.70E-01	1	7
5,6	PLK1|0.415475	membrane disassembly	GO:0030397	1.13E-01	1.70E-01	1	6
6,7	PSME3|0.401575;PLK1|0.415475	positive regulation of cellular protein catabolic process	GO:1903364	1.14E-01	1.70E-01	2	7
5,6,7	TTK|0.488375;PLK1|0.415475	regulation of nuclear division	GO:0051783	1.15E-01	1.71E-01	2	7
5	SNW1|0.473175	modification by host of symbiont morphology or physiology	GO:0051851	1.16E-01	1.72E-01	1	5
9	GMPS|0.496875	guanosine-containing compound biosynthetic process	GO:1901070	1.16E-01	1.72E-01	1	9
6,7,8	IFITM2|5.68315	negative regulation of viral genome replication	GO:0045071	1.16E-01	1.72E-01	1	8
8	CKB|0.486625	monovalent inorganic anion homeostasis	GO:0055083	1.16E-01	1.72E-01	1	8
4,5,6	PLEK|2.071125	positive regulation of actin filament bundle assembly	GO:0032233	1.16E-01	1.72E-01	1	6
6,7,8,9,11	GSN|2.4555	regulation of actin nucleation	GO:0051125	1.16E-01	1.72E-01	1	11
6,7	ASPH|2.59965	positive regulation of cation channel activity	GO:2001259	1.16E-01	1.72E-01	1	7
7,9,10	STAT3|0.4995	regulation of ATP metabolic process	GO:1903578	1.16E-01	1.72E-01	1	10
6	STAT3|0.4995	regulation of nucleoside metabolic process	GO:0009118	1.16E-01	1.72E-01	1	6
7,8	PLEK|2.071125	positive regulation of protein dephosphorylation	GO:0035307	1.16E-01	1.72E-01	1	8
6	GMPS|0.496875;STAT3|0.4995	nucleoside biosynthetic process	GO:0009163	1.16E-01	1.72E-01	2	6
6	HSP90AB1|0.458775;ASPH|2.59965;MAPK14|0.483325	regulation of cytoplasmic transport	GO:1903649	1.17E-01	1.73E-01	3	6
4	LCP1|2.170475;HSPD1|2.88995	leukocyte activation involved in immune response	GO:0002366	1.18E-01	1.75E-01	2	4
5,6	GMPS|0.496875;STAT3|0.4995	glycosyl compound biosynthetic process	GO:1901659	1.18E-01	1.75E-01	2	6
4,5	HSPD1|2.88995;PSME3|0.401575;MAPK14|0.483325	positive regulation of defense response	GO:0031349	1.18E-01	1.75E-01	3	5
5,6,7	SOD2|2.175425	regulation of oxidative stress-induced cell death	GO:1903201	1.18E-01	1.75E-01	1	7
6,7,8	ARHGDIA|3.8215	negative regulation of axonogenesis	GO:0050771	1.18E-01	1.75E-01	1	8
5,6	MAPK14|0.483325	regulation of heart growth	GO:0060420	1.18E-01	1.75E-01	1	6
7	PLEK|2.071125	positive regulation of dephosphorylation	GO:0035306	1.18E-01	1.75E-01	1	7
6,8,9	MAPK14|0.483325	regulation of blood vessel endothelial cell migration	GO:0043535	1.18E-01	1.75E-01	1	9
7,8,9,10	PABPC1|0.3285	regulation of mRNA 3'-end processing	GO:0031440	1.18E-01	1.75E-01	1	10
5	MCMBP|0.4769	sister chromatid cohesion	GO:0007062	1.18E-01	1.75E-01	1	5
4,5	LCP1|2.170475;HSPD1|2.88995	cell activation involved in immune response	GO:0002263	1.19E-01	1.75E-01	2	5

4,5	ACTG1|2.1999;VDAC3|2.015475;MYH10|5.579275	cellular component assembly involved in morphogenesis	GO:0010927	1.19E-01	1.75E-01	3	5
6,7	HSP90AB1|0.458775;KPNA2|0.186675;STAT3|0.4995;IFITM2|5.68315	cytokine-mediated signaling pathway	GO:0019221	1.19E-01	1.76E-01	4	7
5,6,7,8	PSME3|0.401575;SOD2|2.175425	negative regulation of apoptotic signaling	GO:2001234	1.19E-01	1.76E-01	2	8
6	MAPK14|0.483325;PTP4A3|0.3433	epithelial cell migration	GO:0010631	1.19E-01	1.76E-01	2	6
3,4,5	KRT1|0.4198;VTN|2.01125	protein activation cascade	GO:0072376	1.19E-01	1.76E-01	2	5
4	LCP1|2.170475;HSPD1|2.88995;GSN|2.4555;SOS2|11.485	lymphocyte activation	GO:0046649	1.20E-01	1.76E-01	4	4
6,7	NDRG1|10.0073	Schwann cell	GO:0014037	1.20E-01	1.76E-01	1	7
6,8	MAPK14|0.483325	myoblast fusion	GO:0007520	1.20E-01	1.77E-01	1	8
6	VTN|2.01125	endodermal cell differentiation	GO:0035987	1.20E-01	1.77E-01	1	6
5,6,7	VTN|2.01125	positive regulation of receptor-mediated endocytosis	GO:0048260	1.20E-01	1.77E-01	1	7
4	ASPH|2.59965	head morphogenesis	GO:0060323	1.20E-01	1.77E-01	1	4
5	HSPD1|2.88995	response to cocaine	GO:0042220	1.20E-01	1.77E-01	1	5
6,7	MAPK14|0.483325	regulation of myoblast differentiation	GO:0045661	1.20E-01	1.77E-01	1	7
4	SNW1|0.473175	interaction with symbiont	GO:0051702	1.20E-01	1.77E-01	1	4
5	NDE1|0.446425	spindle localization	GO:0051653	1.20E-01	1.77E-01	1	5
8	DNMT1|0.172;ATG7|2.120975;SNW1|0.473175	peptidyl-lysine modification	GO:0018205	1.22E-01	1.77E-01	3	8
6,7,8	SLC25A1|2.1121;SLC2A1|5.081875;HBA1|2.13735;ALB|0.23405	organic anion transport	GO:0015711	1.22E-01	1.78E-01	4	8
5	MAPK14|0.483325;PTP4A3|0.3433	epithelium migration	GO:0090132	1.22E-01	1.79E-01	2	5
6,7	MAPK14|0.483325	lipopolysaccharide-mediated signaling	GO:0031663	1.23E-01	1.79E-01	1	7
7,8,9,10	NSF|2.017425	Golgi to plasma membrane protein	GO:0043001	1.23E-01	1.80E-01	1	10
5,6	SLC2A1|5.081875	vitamin transport	GO:0051180	1.23E-01	1.80E-01	1	6
7	GCH1|4.01735	regulation of nitric-oxide synthase activity	GO:0050999	1.23E-01	1.80E-01	1	7
6,7	SOD2|2.175425	intrinsic apoptotic signaling pathway in response to oxidative	GO:0008631	1.23E-01	1.80E-01	1	7
4,5	STAT3|0.4995;ARHGDIA|3.8215	negative regulation of cellular component movement	GO:0051271	1.24E-01	1.80E-01	2	5
4	GSN|2.4555;NDE1|0.446425	establishment or maintenance of cell polarity	GO:0007163	1.25E-01	1.81E-01	2	4
5,7,8	NSF|2.017425;PLEK|2.071125;MYH10|5.579275;ALB|0.2	exocytosis	GO:0006887	1.25E-01	1.82E-01	4	8
6,7,8,9	RHOG|2.449775	regulation of establishment of protein localization to plasma	GO:0090003	1.25E-01	1.82E-01	1	9
6,7,8	PABPC1|0.3285	negative regulation of mRNA metabolic process	GO:1903312	1.25E-01	1.82E-01	1	8
6,7	SRRT|0.39875	cellular response to	GO:0071359	1.25E-01	1.82E-01	1	7
5,6,7	SNW1|0.473175	regulation of retinoic acid receptor signaling	GO:0048385	1.25E-01	1.82E-01	1	7
8	ATG7|2.120975;ASL|2.252975	protein acetylation	GO:0006473	1.27E-01	1.82E-01	2	8
6,7	ASPH|2.59965;PLEK|2.071125	second-messenger-mediated signaling	GO:0019932	1.27E-01	1.84E-01	2	7
5,6	MTHFD1L|6.667175;MAPK14|0.483325	skeletal system morphogenesis	GO:0048705	1.28E-01	1.84E-01	2	6
4	MAPK14|0.483325;PTP4A3|0.3433	tissue migration	GO:0090130	1.28E-01	1.85E-01	2	4
7	GMPS|0.496875	glutamine metabolic process	GO:0006541	1.28E-01	1.85E-01	1	7
8,9	TXNIP|2.0553	platelet-derived growth factor receptor signaling pathway	GO:0048008	1.28E-01	1.85E-01	1	9
5	STAT3|0.4995	regulation of cofactor metabolic process	GO:0051193	1.28E-01	1.85E-01	1	5
5,6	VDAC3|2.015475	regulation of cilium assembly	GO:1902017	1.28E-01	1.85E-01	1	6
5	HSPD1|2.88995	macrophage activation	GO:0042116	1.28E-01	1.85E-01	1	5
7	RPL4|2.063325	cytoplasmic translation	GO:0002181	1.28E-01	1.85E-01	1	7
6,7,8	S100A11|2.531875	negative regulation of DNA replication	GO:0008156	1.28E-01	1.85E-01	1	8
7	SNW1|0.473175	intrinsic apoptotic signaling pathway in response to DNA damage by p53 class mediator	GO:0042771	1.28E-01	1.85E-01	1	7
6	STAT3|0.4995	regulation of coenzyme metabolic process	GO:0051196	1.28E-01	1.85E-01	1	6
7,8	MTHFD1L|6.667175	embryonic cranial skeleton morphogenesis	GO:0048701	1.30E-01	1.85E-01	1	8
5,6,7	YWHAG|2.359475	protein insertion into membrane	GO:0051205	1.30E-01	1.87E-01	1	7
6,7,8	PLK1|0.415475	positive regulation of mitotic nuclear division	GO:0045840	1.30E-01	1.87E-01	1	8
4	VTN|2.01125	cell adhesion mediated by integrin	GO:0033627	1.30E-01	1.87E-01	1	4
5,6	HSP90AB1|0.458775	regulation of nitric oxide biosynthetic process	GO:0045428	1.30E-01	1.87E-01	1	6

5	ALB|0.23405	bile acid metabolic	GO:0008206	1.30E-01	1.87E-01	1	5
5	MAPK14|0.483325	cellular senescence	GO:0090398	1.30E-01	1.87E-01	1	5
3	MAPK14|0.483325;H3F3A|0.20985	positive regulation of growth	GO:0045927	1.31E-01	1.87E-01	2	3
3	CCT7|2.06135;KRT9|0.268525;AKR1B1|2.235325;EIF4H|0.275675;STAT3|0.4995	multi-organism reproductive process	GO:0044703	1.31E-01	1.89E-01	5	3
6,7,8	YWHAG|2.359475	mitochondrial outer membrane permeabilization	GO:0097345	1.32E-01	1.89E-01	1	8
7,8,9	GSN|2.4555	regulation of epithelial cell apoptotic process	GO:1904035	1.32E-01	1.90E-01	1	9
6	MAPK14|0.483325	regulation of cardiac muscle tissue	GO:0055024	1.32E-01	1.90E-01	1	6
6	CCT7|2.06135	sperm-egg recognition	GO:0035036	1.32E-01	1.90E-01	1	6
6	NCDN|0.4114	regulation of neuronal synaptic plasticity	GO:0048168	1.32E-01	1.90E-01	1	6
6,7,8,9	TTK|0.488375	positive regulation of pathway-restricted SMAD protein phosphorylation	GO:0010862	1.32E-01	1.90E-01	1	9
5,6	AKR1B1|2.235325	positive regulation of smooth muscle cell proliferation	GO:0048661	1.32E-01	1.90E-01	1	6
11,12	ASPH|2.59965;ERO1L|2.370575	positive regulation of cytosolic calcium ion concentration	GO:0007204	1.33E-01	1.90E-01	2	12
7	ASPH|2.59965;ATP6V1B2|2.0575;ERO1L|2.370575;TFRC|0.403125	cation homeostasis	GO:0055080	1.34E-01	1.91E-01	4	7
6,7,8,9	VDAC3|2.015475	regulation of anion transmembrane transport	GO:1903959	1.35E-01	1.92E-01	1	9
5,6	HSPD1|2.88995	somatic diversification of immunoglobulins involved in immune	GO:0002208	1.35E-01	1.92E-01	1	6
4	HSPD1|2.88995	isotype switching	GO:0045190	1.35E-01	1.92E-01	1	4
7,9	ASL|2.252975	glutamine family amino acid biosynthetic process	GO:0009084	1.35E-01	1.92E-01	1	9
7	YWHAG|2.359475	positive regulation of mitochondrial membrane permeability involved in apoptotic process	GO:1902110	1.35E-01	1.92E-01	1	7
6,7	HSPD1|2.88995	somatic recombination of immunoglobulin genes involved in immune response	GO:0002204	1.35E-01	1.92E-01	1	7
6	YWHAG|2.359475	mitochondrial outer membrane permeabilization involved in programmed cell death	GO:1902686	1.35E-01	1.92E-01	1	6
7,8	YWHAG|2.359475;PLK1|0.415475	negative regulation of protein kinase activity	GO:0006469	1.35E-01	1.92E-01	2	8
5,6,7	SNW1|0.473175;TTK|0.488375	regulation of transmembrane receptor protein serine/threonine kinase signaling pathway	GO:0090092	1.36E-01	1.93E-01	2	7
7	DNMT1|0.172;SNW1|0.473175	protein alkylation	GO:0008213	1.37E-01	1.94E-01	2	7
5,7,8	DNMT1|0.172;SNW1|0.473175	protein methylation	GO:0006479	1.37E-01	1.94E-01	2	8
7,8,10	STAT3|0.4995	eye photoreceptor cell differentiation	GO:0001754	1.37E-01	1.94E-01	1	10
6,7	ARHGDIA|3.8215	semaphorin-plexin signaling pathway	GO:0071526	1.37E-01	1.94E-01	1	7
4,6	MAPK14|0.483325	positive regulation of muscle organ	GO:0048636	1.37E-01	1.94E-01	1	6
5	VTN|2.01125	muscle cell migration	GO:0014812	1.37E-01	1.94E-01	1	5
4	ASPH|2.59965	face development	GO:0060324	1.37E-01	1.94E-01	1	4
5	RPL3|3.30745	ribosomal large subunit biogenesis	GO:0042273	1.37E-01	1.94E-01	1	5
4	GSN|2.4555	localization within membrane	GO:0051668	1.37E-01	1.94E-01	1	4
5	PCNA|0.404975	translesion synthesis	GO:0019985	1.37E-01	1.94E-01	1	5
7,9,10	SRRT|0.39875	gene silencing by miRNA	GO:0035195	1.37E-01	1.94E-01	1	10
6,7	MAPK14|0.483325	positive regulation of striated muscle cell differentiation	GO:0051155	1.37E-01	1.94E-01	1	7
5,6	SOD2|2.175425	regulation of cellular response to oxidative	GO:1900407	1.37E-01	1.94E-01	1	6
5,6	MAPK14|0.483325	positive regulation of striated muscle tissue development	GO:0045844	1.37E-01	1.94E-01	1	6
4,5,6	HSPD1|2.88995;GSN|2.4555;SOS2|11.48525	regulation of leukocyte activation	GO:0002694	1.37E-01	1.94E-01	3	6
4	YWHAG|2.359475;NCDN|0.4114	regulation of synapse structure or activity	GO:0050803	1.38E-01	1.94E-01	2	4
6	AKR1B1|2.235325	cellular response to ketone	GO:1901655	1.39E-01	1.95E-01	1	6
6,8,9	YWHAG|2.359475	positive regulation of mitochondrial membrane permeability	GO:0035794	1.39E-01	1.97E-01	1	9
6,7,8	PLEK|2.071125	regulation of calcium-mediated signaling	GO:0050848	1.39E-01	1.97E-01	1	8
7,8,9	HBA1|2.13735	bicarbonate transport	GO:0015701	1.39E-01	1.97E-01	1	9

6,7,8	SNW1|0.473175	positive regulation of viral transcription	GO:0050434	1.39E-01	1.97E-01	1	8
4,5	MAPK14|0.483325	positive regulation of muscle tissue	GO:1901863	1.39E-01	1.97E-01	1	5
6,7,8,9	PLK1|0.415475	homologous chromosome segregation	GO:0045143	1.39E-01	1.97E-01	1	9
5	AKR1B1|2.235325;ISYNA1|0.49065;PLEK|2.071125	alcohol metabolic process	GO:0006066	1.40E-01	1.97E-01	3	5
5,6,7	STAT3|0.4995	regulation of Notch signaling pathway	GO:0008593	1.42E-01	1.97E-01	1	7
6	PLEK|2.071125	cortical cytoskeleton organization	GO:0030865	1.42E-01	1.99E-01	1	6
5,6	HSPD1|2.88995	positive regulation of interferon-gamma production	GO:0032729	1.42E-01	1.99E-01	1	6
4,5,6	MAPK14|0.483325	cardiac muscle tissue growth	GO:0055017	1.42E-01	1.99E-01	1	6
5,6	MAPK14|0.483325	positive regulation of fat cell differentiation	GO:0045600	1.42E-01	1.99E-01	1	6
6,9	SOD2|2.175425	regulation of systemic arterial blood pressure mediated by a chemical signal	GO:0003044	1.42E-01	1.99E-01	1	9
6,7,8	SOD2|2.175425	release of cytochrome c from mitochondria	GO:0001836	1.42E-01	1.99E-01	1	8
7,8,9	HSP90AB1|0.458775;YWHAG|2.359475;LAMTOR2|7.841025;MAPK14|0.483325;PLK1|0.415475	regulation of protein kinase activity	GO:0045859	1.43E-01	1.99E-01	5	9
5	CNDP2|2.0938;SLC25A1|2.1121	sulfur compound biosynthetic process	GO:0044272	1.44E-01	2.00E-01	2	5
5	ACTG1|2.1999;ACTB|2.1999	cell-cell junction organization	GO:0045216	1.44E-01	2.01E-01	2	5
7,8	MAPK14|0.483325	regulation of myotube differentiation	GO:0010830	1.44E-01	2.01E-01	1	8
6,7,8	ERO1L|2.370575	intrinsic apoptotic signaling pathway in response to endoplasmic reticulum stress	GO:0070059	1.44E-01	2.01E-01	1	8
8	GMPS|0.496875	guanosine-containing compound metabolic process	GO:1901068	1.44E-01	2.01E-01	1	8
5	VTN|2.01125	regulation of humoral immune response	GO:0002920	1.44E-01	2.01E-01	1	5
7	HSPD1|2.88995	regulation of T cell mediated immunity	GO:0002709	1.44E-01	2.01E-01	1	7
5	MAPK14|0.483325	syncytium formation by plasma membrane fusion	GO:0000768	1.44E-01	2.01E-01	1	5
5	ATP6V1B2|2.0575	phagosome maturation	GO:0090382	1.44E-01	2.01E-01	1	5
6,7,8	YWHAG|2.359475	regulation of mitochondrial membrane permeability involved in apoptotic process	GO:1902108	1.47E-01	2.01E-01	1	8
6	MAPK14|0.483325	cellular response to ionizing radiation	GO:0071479	1.47E-01	2.04E-01	1	6
5,6	VTN|2.01125	endoderm formation	GO:0001706	1.47E-01	2.04E-01	1	6
4,5	AKR1B1|2.235325	maternal process involved in female pregnancy	GO:0060135	1.47E-01	2.04E-01	1	5
4	SRRT|0.39875;SNW1|0.473175;ARHGDIA|3.8215	positive regulation of nervous system development	GO:0051962	1.47E-01	2.04E-01	3	4
5,7	KRT1|0.4198	multicellular organismal water homeostasis	GO:0050891	1.49E-01	2.05E-01	1	7
5	TFRC|0.403125	cellular response to drug	GO:0035690	1.49E-01	2.07E-01	1	5
4,5	TFRC|0.403125	regulation of tissue remodeling	GO:0034103	1.49E-01	2.07E-01	1	5
6	GMPS|0.496875	purine nucleobase metabolic process	GO:0006144	1.49E-01	2.07E-01	1	6
7	MYH10|5.579275	neuromuscular process controlling balance	GO:0050885	1.49E-01	2.07E-01	1	7
6,7	VDAC3|2.015475;SLC25A1|2.1121;SLC2A1|5.081875;HBA1|2.13735;ALB|0.23405	anion transport	GO:0006820	1.49E-01	2.07E-01	5	7
5	ACTG1|2.1999;ACTB|2.1999	cell junction assembly	GO:0034329	1.50E-01	2.08E-01	2	5
6,7	NSF|2.017425;RHOG|2.449775	protein localization to plasma membrane	GO:0072659	1.50E-01	2.08E-01	2	7
6,7	YWHAG|2.359475;PLK1|0.415475	negative regulation of kinase activity	GO:0033673	1.51E-01	2.08E-01	2	7
6	NSF|2.017425;RHOG|2.449775	protein localization to cell periphery	GO:1990778	1.51E-01	2.09E-01	2	6
5	SOD2|2.175425	regulation of response to oxidative stress	GO:1902882	1.51E-01	2.09E-01	1	5
5,8	NDE1|0.446425	neuroblast proliferation	GO:0007405	1.51E-01	2.09E-01	1	8
8,9	PRDX5|3.0143	NADP metabolic process	GO:0006739	1.51E-01	2.09E-01	1	9
6,10,11	MAPK14|0.483325	regulation of glucose	GO:0046324	1.51E-01	2.09E-01	1	11
6,8,9	SRRT|0.39875	posttranscriptional gene silencing by RNA	GO:0035194	1.51E-01	2.09E-01	1	9
6	GCH1|4.01735	regulation of monooxygenase activity	GO:0032768	1.51E-01	2.09E-01	1	6
6,7	AKR1B1|2.235325	cellular response to hydrogen peroxide	GO:0070301	1.51E-01	2.09E-01	1	7

5	ASPH|2.59965	endoplasmic reticulum to cytosol transport	GO:1903513	1.51E-01	2.09E-01	1	5
6,7	TUBG1|0.4102	cytoplasmic microtubule organization	GO:0031122	1.51E-01	2.09E-01	1	7
5,6	NSF|2.017425;VAT1|2.702925	single-organism membrane fusion	GO:0044801	1.53E-01	2.09E-01	2	6
7	NDE1|0.446425	organelle transport along microtubule	GO:0072384	1.53E-01	2.11E-01	1	7
4	MAPK14|0.483325	syncytium formation	GO:0006949	1.53E-01	2.11E-01	1	4
5	HSPD1|2.88995	somatic recombination of immunoglobulin gene segments	GO:0016447	1.53E-01	2.11E-01	1	5
5,7,8	SRRT|0.39875	posttranscriptional gene silencing	GO:0016441	1.53E-01	2.11E-01	1	8
5,7,8	LIG1|0.440425;BAZ1B|0.460625	double-strand break repair	GO:0006302	1.55E-01	2.11E-01	2	8
4,5	HSPA8|0.3721;NSF|2.017425;YWHAG|2.359475;NCDN|0.4114;SLC2A1|5.081875;IK|0.480125;PLP1|3.936	cell-cell signaling	GO:0007267	1.55E-01	2.13E-01	7	5
6	MAPK14|0.483325;TFRC|0.403125;SOS2|11.48525	leukocyte differentiation	GO:0002521	1.56E-01	2.13E-01	3	6
5,7,8	MAPK14|0.483325	positive regulation of endothelial cell migration	GO:0010595	1.56E-01	2.14E-01	1	8
4	ASPH|2.59965	body morphogenesis	GO:0010171	1.56E-01	2.14E-01	1	4
5,6	SMC2|0.485025	chromosome organization involved in meiosis	GO:0070192	1.56E-01	2.14E-01	1	6
5,6	SOD2|2.175425	cell death in response to oxidative stress	GO:0036473	1.56E-01	2.14E-01	1	6
6,7	ARHGDIA|3.8215	positive regulation of axonogenesis	GO:0050772	1.56E-01	2.14E-01	1	7
6,7,8	PLK1|0.415475	positive regulation of mitotic cell cycle phase transition	GO:1901992	1.56E-01	2.14E-01	1	8
4	HSP90AB1|0.458775;GSN|2.4555;PLEK|2.071125	regulation of cellular component size	GO:0032535	1.56E-01	2.14E-01	3	4
9,10	PSME3|0.401575;PLK1|0.415475	protein ubiquitination involved in ubiquitin-dependent protein catabolic process	GO:0042787	1.57E-01	2.14E-01	2	10
6,7	HSPA8|0.3721;PABPC1|0.3285;SARS|2.2366;SRRT|0.39875;PABPC4|0.26245;SNW1|0.473175	RNA processing	GO:0006396	1.58E-01	2.15E-01	6	7
6,7,8,9	SLC2A1|5.081875	hexose transmembrane transport	GO:0035428	1.58E-01	2.16E-01	1	9
5,6	HSPD1|2.88995	positive regulation of interleukin-6 production	GO:0032755	1.58E-01	2.16E-01	1	6
5	RRM2|0.21675	deoxyribonucleotide metabolic process	GO:0009262	1.58E-01	2.16E-01	1	5
6	ALDH1L2|2.148;ASL|2.252975	carboxylic acid catabolic process	GO:0046395	1.58E-01	2.16E-01	2	6
5	ALDH1L2|2.148;ASL|2.252975	organic acid catabolic process	GO:0016054	1.58E-01	2.16E-01	2	5
4,6	HSPD1|2.88995;MAPK14|0.483325;GCH1|4.01735	response to bacterium	GO:0009617	1.58E-01	2.16E-01	3	6
3	LCP1|2.170475;GSN|2.4555;PLEK|2.071125	positive regulation of cellular component biogenesis	GO:0044089	1.60E-01	2.16E-01	3	3
5,8	BAZ1B|0.460625	histone phosphorylation	GO:0016572	1.60E-01	2.18E-01	1	8
6,7,8	RHOG|2.449775	regulation of protein localization to plasma membrane	GO:1903076	1.60E-01	2.18E-01	1	8
5	NDE1|0.446425	establishment of chromosome localization	GO:0051303	1.60E-01	2.18E-01	1	5
6,7	RHOG|2.449775	regulation of protein localization to cell periphery	GO:1904375	1.60E-01	2.18E-01	1	7
5	AKR1B1|2.235325	quinone metabolic process	GO:1901661	1.60E-01	2.18E-01	1	5
6,7,8,9	TTK|0.488375	regulation of pathway-restricted SMAD protein phosphorylation	GO:0060393	1.60E-01	2.18E-01	1	9
5	NDE1|0.446425	chromosome localization	GO:0050000	1.60E-01	2.18E-01	1	5
7,8,9,10	HSP90AB1|0.458775;PSME3|0.401575;TTK|0.488375;PLK1|0.415475	proteasome-mediated ubiquitin-dependent protein catabolic process	GO:0043161	1.61E-01	2.18E-01	4	10
10,11	ASPH|2.59965;ERO1L|2.370575	cytosolic calcium ion homeostasis	GO:0051480	1.62E-01	2.18E-01	2	11
7,8	HSPA8|0.3721;PABPC1|0.3285;SNW1|0.473175	RNA splicing	GO:0008380	1.62E-01	2.20E-01	3	8
5,6,7,8	PLK1|0.415475	positive regulation of nuclear division	GO:0051785	1.63E-01	2.20E-01	1	8
5	HSPD1|2.88995	immunoglobulin production involved in immunoglobulin mediated	GO:0002381	1.63E-01	2.20E-01	1	5
6	PLK1|0.415475	positive regulation of cell cycle phase transition	GO:1901989	1.63E-01	2.20E-01	1	6
6,7,8	KPNA2|0.186675	regulation of DNA recombination	GO:0000018	1.63E-01	2.20E-01	1	8
6,9	ATG7|2.120975	forebrain neuron differentiation	GO:0021879	1.63E-01	2.20E-01	1	9
5,6	SRRT|0.39875;SNW1|0.473175;ARHGDIA|3.8215	positive regulation of cell development	GO:0010720	1.64E-01	2.20E-01	3	6
4	HSPA8|0.3721;ENO1|2.1939;ATP6V1B2|2.0575;GMPS|0.496875;AKR1B1|2.235325;ALDOC|2.04725;RRM2|0.21675;STAT3|0.4995	carbohydrate derivative metabolic process	GO:1901135	1.64E-01	2.21E-01	8	4

8,10,11	MAPK14|0.483325	toll-like receptor 10 signaling pathway	GO:0034166	1.65E-01	2.22E-01	1	11
8,9	SOD2|2.175425	protein homotetramerization	GO:0051289	1.65E-01	2.22E-01	1	9
5,6	VIM|2.241075	SMAD protein signal transduction	GO:0060395	1.65E-01	2.22E-01	1	6
7	PSME3|0.401575	DNA damage response, signal transduction by p53 class mediator resulting in cell cycle arrest	GO:0006977	1.65E-01	2.22E-01	1	7
5	MAPK14|0.483325	heart growth	GO:0060419	1.65E-01	2.22E-01	1	5
7	STAT3|0.4995	negative regulation of purine nucleotide metabolic process	GO:1900543	1.65E-01	2.22E-01	1	7
7,9	STAT3|0.4995	photoreceptor cell differentiation	GO:0046530	1.65E-01	2.22E-01	1	9
8,10,11	MAPK14|0.483325	toll-like receptor 5 signaling pathway	GO:0034146	1.65E-01	2.22E-01	1	11
7	ATG7|2.120975;ASL|2.252975	protein acylation	GO:0043543	1.65E-01	2.22E-01	2	7
4	VIM|2.241075	lens development in camera-type eye	GO:0002088	1.67E-01	2.22E-01	1	4
5	CCT7|2.06135	cell-cell recognition	GO:0009988	1.67E-01	2.24E-01	1	5
8	TTK|0.488375	pathway-restricted SMAD protein phosphorylation	GO:0060389	1.67E-01	2.24E-01	1	8
6,7,8	PCNA|0.404975	positive regulation of DNA replication	GO:0045740	1.67E-01	2.24E-01	1	8
8,9	PSME3|0.401575	intracellular signal transduction involved in G1 DNA damage	GO:1902400	1.67E-01	2.24E-01	1	9
7,8,9	NSF|2.017425	Golgi to plasma membrane transport	GO:0006893	1.67E-01	2.24E-01	1	9
8,9,10,11	PSME3|0.401575	signal transduction involved in mitotic G1 DNA damage checkpoint	GO:0072431	1.67E-01	2.24E-01	1	11
5,6	MAPK14|0.483325;SLC2A1|5.081875	carbohydrate transport	GO:0008643	1.69E-01	2.24E-01	2	6
7	CKB|0.486625	anion homeostasis	GO:0055081	1.70E-01	2.26E-01	1	7
8	MAPK14|0.483325	blood vessel endothelial cell migration	GO:0043534	1.70E-01	2.27E-01	1	8
6,9,10,11	ATG7|2.120975	regulation of histone acetylation	GO:0035065	1.70E-01	2.27E-01	1	11
8,9	GSN|2.4555	actin filament capping	GO:0051693	1.70E-01	2.27E-01	1	9
6,7	SNW1|0.473175	retinoic acid receptor signaling pathway	GO:0048384	1.70E-01	2.27E-01	1	7
5	SOD2|2.175425	superoxide metabolic process	GO:0006801	1.70E-01	2.27E-01	1	5
6	SNW1|0.473175	cellular response to retinoic acid	GO:0071300	1.72E-01	2.27E-01	1	6
4,5,6	HSPD1|2.88995	B cell activation involved in immune response	GO:0002312	1.72E-01	2.29E-01	1	6
7,8,9	PSME3|0.401575	signal transduction involved in mitotic DNA integrity checkpoint	GO:1902403	1.72E-01	2.29E-01	1	9
7,8,9,10	PSME3|0.401575	signal transduction involved in mitotic DNA damage checkpoint	GO:1902402	1.72E-01	2.29E-01	1	10
6,7,8	PSME3|0.401575	signal transduction involved in mitotic cell cycle checkpoint	GO:0072413	1.72E-01	2.29E-01	1	8
6,12	ATG7|2.120975	histone H4 acetylation	GO:0043967	1.72E-01	2.29E-01	1	12
5,6	LCP1|2.170475;MTHFD1L|6.667175;GSN|2.4555	morphogenesis of an epithelium	GO:0002009	1.74E-01	2.29E-01	3	6
5	ATG7|2.120975;ALB|0.23405	lipoprotein metabolic process	GO:0042157	1.74E-01	2.31E-01	2	5
6,7	HSPD1|2.88995	positive regulation of adaptive immune response based on somatic recombination of immune receptors built from immunoglobulin superfamily domains	GO:0002824	1.74E-01	2.31E-01	1	7
6,7	STAT3|0.4995	negative regulation of nucleotide metabolic process	GO:0045980	1.74E-01	2.31E-01	1	7
6	SNW1|0.473175	skeletal muscle cell differentiation	GO:0035914	1.74E-01	2.31E-01	1	6
6,7,8,9,11,12	ASPH|2.59965	regulation of release of sequestered calcium ion into cytosol	GO:0051279	1.74E-01	2.31E-01	1	12
5	HIST1H4A|0.23835	negative regulation of myeloid cell	GO:0045638	1.74E-01	2.31E-01	1	5
4	AKR1B1|2.235325	secondary metabolic process	GO:0019748	1.74E-01	2.31E-01	1	4
6,8	HSP90AB1|0.458775;YWHAG|2.359475;LAMTOR2|7.841025;MAPK14|0.483325;PLK1|0.415475	regulation of kinase activity	GO:0043549	1.75E-01	2.31E-01	5	8
6,7,8	HSP90AB1|0.458775;PSME3|0.401575;TTK|0.488375;PLK1|0.415475	proteasomal protein catabolic process	GO:0010498	1.75E-01	2.32E-01	4	8
5,6	HSPD1|2.88995;SOS2|11.48525	lymphocyte proliferation	GO:0046651	1.76E-01	2.32E-01	2	6

5	AKR1B1|2.235325;SLC25A1|2.1121;ISYNA1|0.49065;PLP1|3.936	lipid biosynthetic process	GO:0008610	1.76E-01	2.32E-01	4	5
5,6	HSPA8|0.3721;NSF|2.017425;YWHAG|2.359475;NCDN|0.4114;PLP1|3.936	synaptic transmission	GO:0007268	1.76E-01	2.32E-01	5	6
8,9	PLK1|0.415475	negative regulation of cyclin-dependent protein kinase activity	GO:1904030	1.76E-01	2.32E-01	1	9
8,10,11	MAPK14|0.483325	toll-like receptor TLR1:TLR2 signaling pathway	GO:0038123	1.76E-01	2.32E-01	1	11
8,10,11	MAPK14|0.483325	toll-like receptor TLR6:TLR2 signaling pathway	GO:0038124	1.76E-01	2.32E-01	1	11
5,6,7	RHOG|2.449775	regulation of plasma membrane organization	GO:1903729	1.76E-01	2.32E-01	1	7
6,7,8	PSME3|0.401575	signal transduction involved in DNA integrity	GO:0072401	1.76E-01	2.32E-01	1	8
5,6,7	SLC2A1|5.081875	carbohydrate transmembrane transport	GO:0034219	1.76E-01	2.32E-01	1	7
7	PSME3|0.401575	signal transduction involved in DNA damage	GO:0072422	1.76E-01	2.32E-01	1	7
7	SLC25A1|2.1121	triglyceride biosynthetic process	GO:0019432	1.76E-01	2.32E-01	1	7
4	HIST1H4A|0.23835	demethylation	GO:0070988	1.76E-01	2.32E-01	1	4
6	HSPD1|2.88995	positive regulation of lymphocyte mediated immunity	GO:0002708	1.76E-01	2.32E-01	1	6
4,5	HSPD1|2.88995	cytokine production involved in immune response	GO:0002367	1.76E-01	2.32E-01	1	5
8	BAZ1B|0.460625;VTN|2.01125;TTK|0.488375	peptidyl-tyrosine phosphorylation	GO:0018108	1.77E-01	2.32E-01	3	8
4	MTHFD1L|6.667175;BTF3|0.2449;TOP2A|0.481675;VTN|2.01125;MYH10|5.579275	embryo development	GO:0009790	1.77E-01	2.33E-01	5	4
5	HSPD1|2.88995;SOS2|11.48525	mononuclear cell proliferation	GO:0032943	1.78E-01	2.33E-01	2	5
4	MAPK14|0.483325;STAT3|0.4995	regulation of developmental growth	GO:0048638	1.78E-01	2.34E-01	2	4
8,9	HSP90AB1|0.458775;PSME3|0.401575;SQSTM1|6.007525;TTK|0.488375;PLK1|0.415475	ubiquitin-dependent protein catabolic process	GO:0006511	1.79E-01	2.34E-01	5	9
8,9,10	ATG7|2.120975	regulation of peptidyl-lysine acetylation	GO:2000756	1.79E-01	2.34E-01	1	10
8	PLK1|0.415475	peptidyl-threonine phosphorylation	GO:0018107	1.79E-01	2.34E-01	1	8
4	ANXA5|3.637725	sperm motility	GO:0030317	1.79E-01	2.34E-01	1	4
6	RPL3|3.30745	ribosome assembly	GO:0042255	1.79E-01	2.34E-01	1	6
5,8	ATG7|2.120975	forebrain generation of neurons	GO:0021872	1.79E-01	2.34E-01	1	8
6,7	PLK1|0.415475	microtubule bundle formation	GO:0001578	1.79E-01	2.34E-01	1	7
7	PCNA|0.404975	cellular response to UV	GO:0034644	1.79E-01	2.34E-01	1	7
5	ARHGDIA|3.8215	cellular response to mechanical stimulus	GO:0071260	1.79E-01	2.34E-01	1	5
5,6,7	PSME3|0.401575	signal transduction involved in cell cycle	GO:0072395	1.79E-01	2.34E-01	1	7
5	PSME3|0.401575;CDC123|0.29925	cell cycle arrest	GO:0007050	1.79E-01	2.34E-01	2	5
4	ACTG1|2.1999;ACTB|2.1999	cell junction organization	GO:0034330	1.80E-01	2.34E-01	2	4
5	NDRG1|10.0073	mast cell activation	GO:0045576	1.81E-01	2.35E-01	1	5
5	HSPD1|2.88995	positive regulation of adaptive immune response	GO:0002821	1.81E-01	2.36E-01	1	5
5	SLC25A1|2.1121	neutral lipid biosynthetic process	GO:0046460	1.81E-01	2.36E-01	1	5
6	SLC25A1|2.1121	acylglycerol biosynthetic process	GO:0046463	1.81E-01	2.36E-01	1	6
3,4	CCT7|2.06135;KRT9|0.268525;EIF4H|0.275675;STAT3|0.	sexual reproduction	GO:0019953	1.81E-01	2.36E-01	4	4
6	GSN|2.4555;PLEK|2.071125	positive regulation of cytoskeleton organization	GO:0051495	1.82E-01	2.37E-01	2	6
6,7,8,9	AKR1B1|2.235325	positive regulation of JAK-STAT cascade	GO:0046427	1.83E-01	2.37E-01	1	9
7,8	SNW1|0.473175	intrinsic apoptotic signaling pathway by p53 class mediator	GO:0072332	1.83E-01	2.38E-01	1	8
5,6	ASPH|2.59965	positive regulation of ion transmembrane transporter activity	GO:0032414	1.83E-01	2.38E-01	1	6
8	GSN|2.4555	epithelial cell apoptotic process	GO:1904019	1.83E-01	2.38E-01	1	8
8	BAZ1B|0.460625;VTN|2.01125;TTK|0.488375	peptidyl-tyrosine modification	GO:0018212	1.84E-01	2.38E-01	3	8
8	ATG7|2.120975	C-terminal protein	GO:0006501	1.85E-01	2.39E-01	1	8
8,10,11	MAPK14|0.483325	toll-like receptor 9 signaling pathway	GO:0034162	1.85E-01	2.41E-01	1	11
4	AKR1B1|2.235325	hormone biosynthetic process	GO:0042446	1.85E-01	2.41E-01	1	4
5,6,7	PSME3|0.401575	regulation of cellular amino acid metabolic	GO:0006521	1.85E-01	2.41E-01	1	7

5	HSP90AB1|0.458775;HSPD1|2.88995;ASPH|2.59965;GSN|2.4555;CNDP2|2.0938;PRDX5|3.0143;PSME3|0.401575;SQSTM1|6.007525;VTN|2.01125;DNPEP|2.077575;TTK|0.488375;PLK1|0.415475	proteolysis	GO:0006508	1.86E-01	2.41E-01	12	5
7,8	HSP90AB1|0.458775;MAPK14|0.483325	regulation of protein targeting	GO:1903533	1.86E-01	2.41E-01	2	8
7,8	HSP90AB1|0.458775;PSME3|0.401575;SQSTM1|6.007525;TTK|0.488375;PLK1|0.415475	modification-dependent protein catabolic process	GO:0019941	1.87E-01	2.42E-01	5	8
8	PLK1|0.415475	peptidyl-threonine modification	GO:0018210	1.88E-01	2.42E-01	1	8
4,5	STAT3|0.4995	regulation of multicellular organism growth	GO:0040014	1.88E-01	2.43E-01	1	5
9,10	STAT3|0.4995	ATP biosynthetic process	GO:0006754	1.88E-01	2.43E-01	1	10
4	HSPD1|2.88995;SOS2|11.48525	leukocyte proliferation	GO:0070661	1.89E-01	2.43E-01	2	4
5	SOS2|11.48525	thymocyte aggregation	GO:0071594	1.90E-01	2.45E-01	1	5
5	STAT3|0.4995	somatic stem cell maintenance	GO:0035019	1.90E-01	2.45E-01	1	5
6,7,8	VTN|2.01125	regulation of receptor-mediated endocytosis	GO:0048259	1.90E-01	2.45E-01	1	8
5	STAT3|0.4995	cellular carbohydrate catabolic process	GO:0044275	1.90E-01	2.45E-01	1	5
6	SOS2|11.48525	T cell differentiation in thymus	GO:0033077	1.90E-01	2.45E-01	1	6
7,8,9	ATG7|2.120975	regulation of protein acetylation	GO:1901983	1.90E-01	2.45E-01	1	9
7,8	GSN|2.4555	negative regulation of actin filament	GO:0030835	1.90E-01	2.45E-01	1	8
5	CNDP2|2.0938	sulfur amino acid metabolic process	GO:0000096	1.90E-01	2.45E-01	1	5
9,11,12	MAPK14|0.483325	TRIF-dependent toll-like receptor signaling	GO:0035666	1.90E-01	2.45E-01	1	12
6	HSP90AB1|0.458775;PSME3|0.401575;SQSTM1|6.007525;TTK|0.488375;PLK1|0.415475	modification-dependent macromolecule catabolic process	GO:0043632	1.91E-01	2.45E-01	5	6
5	GMPS|0.496875	pigment biosynthetic process	GO:0046148	1.92E-01	2.46E-01	1	5
4,5	PLEK|2.071125	regulation of actin filament bundle assembly	GO:0032231	1.92E-01	2.47E-01	1	5
8,10,11	MAPK14|0.483325	toll-like receptor 2 signaling pathway	GO:0034134	1.92E-01	2.47E-01	1	11
6,7	VTN|2.01125	regulation of acute inflammatory response	GO:0002673	1.92E-01	2.47E-01	1	7
4,6	PCNA|0.404975	positive regulation of response to DNA damage stimulus	GO:2001022	1.92E-01	2.47E-01	1	6
4	GCH1|4.01735	multicellular organismal response to stress	GO:0033555	1.92E-01	2.47E-01	1	4
6,7	SARS|2.2366;YARS|2.288275;NARS|2.162;SRRT|0.39875	ncRNA metabolic process	GO:0034660	1.92E-01	2.47E-01	4	7
5	VDAC3|2.015475;RHOG|2.449775;MYH10|5.579275	cell projection assembly	GO:0030031	1.94E-01	2.47E-01	3	5
5	NDE1|0.446425	centrosome duplication	GO:0051298	1.94E-01	2.49E-01	1	5
7,8,9,10	PABPC1|0.3285	mRNA polyadenylation	GO:0006378	1.94E-01	2.49E-01	1	10
3,6	GSN|2.4555	muscle adaptation	GO:0043500	1.94E-01	2.49E-01	1	6
5,6	PLK1|0.415475	nuclear envelope organization	GO:0006998	1.94E-01	2.49E-01	1	6
4,5	HSPD1|2.88995	somatic diversification of immunoglobulins	GO:0016445	1.94E-01	2.49E-01	1	5
6	MAPK14|0.483325	myoblast differentiation	GO:0045445	1.96E-01	2.49E-01	1	6
4,5,6	LCP1|2.170475	T cell activation involved in immune response	GO:0002286	1.96E-01	2.51E-01	1	6
6,7	PABPC1|0.3285	RNA polyadenylation	GO:0043631	1.96E-01	2.51E-01	1	7
4	SOS2|11.48525	lymphocyte homeostasis	GO:0002260	1.96E-01	2.51E-01	1	4
5,6,7	PCNA|0.404975	regulation of DNA repair	GO:0006282	1.96E-01	2.51E-01	1	7
4	VIM|2.241075;STAT3|0.4995;MYH10|5.579275	sensory organ	GO:0007423	1.97E-01	2.51E-01	3	4
6	VIM|2.241075;MYH10|5.579275	camera-type eye development	GO:0043010	1.98E-01	2.52E-01	2	6
7,8,9,10,11	PSME3|0.401575	mitotic G1 DNA damage checkpoint	GO:0031571	1.99E-01	2.52E-01	1	11
9,10	PABPC1|0.3285	nuclear-transcribed mRNA catabolic process, deadenylation-dependent decay	GO:0000288	1.99E-01	2.52E-01	1	10
5	SLC2A1|5.081875	oligosaccharide metabolic process	GO:0009311	1.99E-01	2.52E-01	1	5
6	ASPH|2.59965	positive regulation of cation transmembrane	GO:1904064	1.99E-01	2.52E-01	1	6
7,8	TOP2A|0.481675	reciprocal DNA recombination	GO:0035825	1.99E-01	2.52E-01	1	8
4,8,9	TOP2A|0.481675	reciprocal meiotic recombination	GO:0007131	1.99E-01	2.52E-01	1	9
8,10,11	MAPK14|0.483325	MyD88-independent toll-like receptor signaling pathway	GO:0002756	1.99E-01	2.52E-01	1	11
5	VTN|2.01125	endoderm development	GO:0007492	1.99E-01	2.52E-01	1	5
8	ATG7|2.120975	C-terminal protein amino acid modification	GO:0018410	1.99E-01	2.52E-01	1	8
6	HSPD1|2.88995	T cell mediated immunity	GO:0002456	2.01E-01	2.52E-01	1	6
7,8,9,10	PSME3|0.401575	mitotic G1/S transition checkpoint	GO:0044819	2.01E-01	2.54E-01	1	10

4,5,6	ASPH|2.59965	positive regulation of transporter activity	GO:0032411	2.01E-01	2.54E-01	1	6
7,8,9	ASPH|2.59965	regulation of cardiac muscle contraction	GO:0055117	2.01E-01	2.54E-01	1	9
5,6	MAPK14|0.483325	positive regulation of myeloid cell	GO:0045639	2.01E-01	2.54E-01	1	6
7	PSME3|0.401575	G1 DNA damage checkpoint	GO:0044783	2.01E-01	2.54E-01	1	7
4,5	MAPK14|0.483325	regulation of organ growth	GO:0046620	2.03E-01	2.54E-01	1	5
6,7	PLK1|0.415475	mitotic spindle assembly	GO:0090307	2.03E-01	2.57E-01	1	7
5,6	MAPK14|0.483325	positive regulation of cytokine secretion	GO:0050715	2.03E-01	2.57E-01	1	6
5	PLEK|2.071125	regulation of carbohydrate biosynthetic process	GO:0043255	2.03E-01	2.57E-01	1	5
6,7,8	LAMTOR2|7.841025	regulation of TOR	GO:0032006	2.03E-01	2.57E-01	1	8
5	MAPK14|0.483325;PTP4A3|0.3433	ameboidal-type cell migration	GO:0001667	2.03E-01	2.57E-01	2	5
4	AKR1B1|2.235325;PSME3|0.401575	cellular ketone metabolic process	GO:0042180	2.03E-01	2.57E-01	2	4
5	AKR1B1|2.235325	regulation of smooth muscle cell proliferation	GO:0048660	2.05E-01	2.57E-01	1	5
5	STAT3|0.4995	regulation of generation of precursor metabolites and energy	GO:0043467	2.05E-01	2.58E-01	1	5
5,7,8	PCNA|0.404975	postreplication repair	GO:0006301	2.05E-01	2.58E-01	1	8
6,7	ATG7|2.120975	central nervous system neuron development	GO:0021954	2.05E-01	2.58E-01	1	7
5,6	CNDP2|2.0938	glutathione metabolic process	GO:0006749	2.05E-01	2.58E-01	1	6
8,9,10,11	PLK1|0.415475	positive regulation of proteasomal ubiquitin-dependent protein catabolic process	GO:0032436	2.05E-01	2.58E-01	1	11
6,7	YWHAG|2.359475;STAT3|0.4995	mitochondrial transport	GO:0006839	2.06E-01	2.58E-01	2	7
4	ATP6V1B2|2.0575;DNMT1|0.172;AKR1B1|2.235325;LAMTOR2|7.841025;SNW1|0.473175;STAT3|0.4995	cellular response to endogenous stimulus	GO:0071495	2.06E-01	2.60E-01	6	4
4	YWHAG|2.359475;NCDN|0.4114	modulation of synaptic transmission	GO:0050804	2.07E-01	2.60E-01	2	4
6,7	GSN|2.4555	receptor clustering	GO:0043113	2.07E-01	2.61E-01	1	7
5,6,7	HSPD1|2.88995	positive regulation of type I interferon production	GO:0032481	2.07E-01	2.61E-01	1	7
3	HSP90AB1|0.458775;CCT7|2.06135;KRT9|0.268525;AKR1B1|2.235325;MAPK14|0.483325	single organism reproductive process	GO:0044702	2.08E-01	2.61E-01	5	3
5	AKR1B1|2.235325	smooth muscle cell proliferation	GO:0048659	2.09E-01	2.61E-01	1	5
5,6	MAPK14|0.483325	positive regulation of muscle cell differentiation	GO:0051149	2.09E-01	2.63E-01	1	6
6,8	SNW1|0.473175	regulation of viral transcription	GO:0046782	2.09E-01	2.63E-01	1	8
4	ESD|2.155525	cellular aldehyde metabolic process	GO:0006081	2.12E-01	2.63E-01	1	4
4	ASPH|2.59965	palate development	GO:0060021	2.12E-01	2.65E-01	1	4
6	PSME3|0.401575	positive regulation of cell cycle arrest	GO:0071158	2.12E-01	2.65E-01	1	6
5,6,7	MAPK14|0.483325;SLC2A1|5.081875;PLEK|2.071125	protein secretion	GO:0009306	2.12E-01	2.65E-01	3	7
5	NSF|2.017425;ATG7|2.120975;MAPK14|0.483325;SLC2A1|5.081875	regulation of secretion	GO:0051046	2.13E-01	2.66E-01	4	5
9,10	ATP6V1B2|2.0575	ATP hydrolysis coupled proton transport	GO:0015991	2.14E-01	2.67E-01	1	10
4	SLC25A1|2.1121	thioester metabolic	GO:0035383	2.14E-01	2.67E-01	1	4
6	MAPK14|0.483325	regulation of striated muscle cell differentiation	GO:0051153	2.14E-01	2.67E-01	1	6
5,6	HSPD1|2.88995	positive regulation of leukocyte mediated immunity	GO:0002705	2.14E-01	2.67E-01	1	6
8,9	ATP6V1B2|2.0575	energy coupled proton transmembrane transport, against electrochemical gradient	GO:0015988	2.14E-01	2.67E-01	1	9
5,6	SLC25A1|2.1121	acyl-CoA metabolic process	GO:0006637	2.14E-01	2.67E-01	1	6
6,7	GMPS|0.496875;RRM2|0.21675;STAT3|0.4995	nucleotide biosynthetic process	GO:0009165	2.16E-01	2.67E-01	3	7
5,6,7,8,10,11	ASPH|2.59965	regulation of calcium ion transport into cytosol	GO:0010522	2.16E-01	2.69E-01	1	11
5	SRRT|0.39875	response to dsRNA	GO:0043331	2.16E-01	2.69E-01	1	5
4,6,7	ATG7|2.120975	secretion by tissue	GO:0032941	2.16E-01	2.69E-01	1	7
4,5,6	HSPD1|2.88995;PLEK|2.071125	positive regulation of cell activation	GO:0050867	2.17E-01	2.69E-01	2	6
5,6	HSPD1|2.88995	regulation of interferon-gamma production	GO:0032649	2.18E-01	2.70E-01	1	6
6	KRT9|0.268525;TXNIP|2.0553	epidermis development	GO:0008544	2.18E-01	2.71E-01	2	6
6,8	GSN|2.4555;PLEK|2.071125	regulation of actin polymerization or depolymerization	GO:0008064	2.18E-01	2.71E-01	2	8
8	MAPK14|0.483325;PLK1|0.415475	peptidyl-serine phosphorylation	GO:0018105	2.18E-01	2.71E-01	2	8

5	GSN|2.4555;PLEK|2.071125	regulation of actin filament length	GO:0030832	2.19E-01	2.71E-01	2	5
7,9	VIM|2.241075;YWHAG|2.359475;ARHGDIA|3.8215	regulation of neuron differentiation	GO:0045664	2.20E-01	2.72E-01	3	9
6,7	PLK1|0.415475	microtubule cytoskeleton organization involved in mitosis	GO:1902850	2.20E-01	2.73E-01	1	7
7,8,9	GSN|2.4555	negative regulation of actin filament	GO:0030837	2.20E-01	2.73E-01	1	9
4	ATG7|2.120975	adult locomotory behavior	GO:0008344	2.20E-01	2.73E-01	1	4
5	HSPD1|2.88995	response to ammonium	GO:0060359	2.20E-01	2.73E-01	1	5
5,6	GMPS|0.496875;RRM2|0.21675;STAT3|0.4995	nucleoside phosphate biosynthetic process	GO:1901293	2.22E-01	2.73E-01	3	6
4	KRT1|0.4198;VTN|2.01125	humoral immune response	GO:0006959	2.22E-01	2.75E-01	2	4
6	KRT1|0.4198	water homeostasis	GO:0030104	2.22E-01	2.75E-01	1	6
6,7	MAPK14|0.483325	fatty acid oxidation	GO:0019395	2.22E-01	2.75E-01	1	7
6,7,8	PABPC1|0.3285	positive regulation of translation	GO:0045727	2.22E-01	2.75E-01	1	8
8,10,11	MAPK14|0.483325	toll-like receptor 3 signaling pathway	GO:0034138	2.24E-01	2.75E-01	1	11
5,8	SOD2|2.175425	regulation of systemic arterial blood pressure	GO:0003073	2.24E-01	2.77E-01	1	8
3	SOS2|11.48525	leukocyte homeostasis	GO:0001776	2.24E-01	2.77E-01	1	3
6	VIM|2.241075	actin-mediated cell contraction	GO:0070252	2.24E-01	2.77E-01	1	6
5,6	MAPK14|0.483325	positive regulation of cyclase activity	GO:0031281	2.24E-01	2.77E-01	1	6
7,8	ASPH|2.59965	regulation of calcium ion transmembrane transporter activity	GO:1901019	2.24E-01	2.77E-01	1	8
7,8,9	SQSTM1|6.007525;MAPK14|0.483325;TOP2A|0.481675;SNW1|0.473175;STAT3|0.4995	positive regulation of transcription from RNA polymerase II promoter	GO:0045944	2.26E-01	2.77E-01	5	9
5	VDAC3|2.015475;ARHGDIA|3.8215;MYH10|5.579275	regulation of cell morphogenesis	GO:0022604	2.26E-01	2.79E-01	3	5
5,6	MAPK14|0.483325	lipid oxidation	GO:0034440	2.27E-01	2.79E-01	1	6
5	GMPS|0.496875	nucleobase metabolic process	GO:0009112	2.27E-01	2.79E-01	1	5
6,7	HSP90AB1|0.458775;PSME3|0.401575;SQSTM1|6.007525;TTK|0.488375;PLK1|0.415475	proteolysis involved in cellular protein catabolic process	GO:0051603	2.27E-01	2.79E-01	5	7
5	AKR1B1|2.235325;ALB|0.23405	steroid metabolic process	GO:0008202	2.27E-01	2.79E-01	2	5
4,5	SRRT|0.39875;MAPK14|0.483325;SNW1|0.473175;ARHGDIA|3.8215	positive regulation of cell differentiation	GO:0045597	2.28E-01	2.80E-01	4	5
7,8,9,10	ASPH|2.59965	regulation of calcium ion transmembrane transport	GO:1903169	2.29E-01	2.80E-01	1	10
5,6,7	ARHGDIA|3.8215	negative regulation of cell morphogenesis involved in differentiation	GO:0010771	2.29E-01	2.81E-01	1	7
7,8	NSF|2.017425	intra-Golgi vesicle-mediated transport	GO:0006891	2.29E-01	2.81E-01	1	8
2	CBX3|0.485775;TOP2A|0.481675	rhythmic process	GO:0048511	2.30E-01	2.81E-01	2	2
5	NDRG1|10.0073	peripheral nervous system development	GO:0007422	2.31E-01	2.83E-01	1	5
5,6	KRT1|0.4198	negative regulation of inflammatory response	GO:0050728	2.31E-01	2.83E-01	1	6
6,7	LAMTOR2|7.841025	TOR signaling	GO:0031929	2.33E-01	2.83E-01	1	7
4	MAPK14|0.483325	cell aging	GO:0007569	2.33E-01	2.85E-01	1	4
5,6	HSPD1|2.88995	regulation of interleukin-6 production	GO:0032675	2.33E-01	2.85E-01	1	6
5,6	PSME3|0.401575	regulation of cellular amine metabolic process	GO:0033238	2.33E-01	2.85E-01	1	6
6	LCP1|2.170475;GSN|2.4555;PLEK|2.071125	actin filament	GO:0007015	2.34E-01	2.85E-01	3	6
7,8	ASPH|2.59965	regulation of striated muscle contraction	GO:0006942	2.35E-01	2.87E-01	1	8
6,7,8	SOD2|2.175425	negative regulation of intrinsic apoptotic signaling pathway	GO:2001243	2.35E-01	2.87E-01	1	8
5,9,10	MAPK14|0.483325	regulation of glucose transport	GO:0010827	2.35E-01	2.87E-01	1	10
4,6	MAPK14|0.483325	positive regulation of epithelial cell migration	GO:0010634	2.35E-01	2.87E-01	1	6
7,8,9,10	LAMTOR2|7.841025	activation of MAPKK activity	GO:0000186	2.35E-01	2.87E-01	1	10
4	NDE1|0.446425;MYH10|5.579275;ALB|0.23405	organelle localization	GO:0051640	2.36E-01	2.87E-01	3	4
8	MAPK14|0.483325;PLK1|0.415475	peptidyl-serine modification	GO:0018209	2.36E-01	2.88E-01	2	8
3,4	TOP2A|0.481675	regulation of circadian rhythm	GO:0042752	2.37E-01	2.88E-01	1	4
5,6,7	ATG7|2.120975	body fluid secretion	GO:0007589	2.37E-01	2.89E-01	1	7
6	GSN|2.4555;PLEK|2.071125;PLK1|0.415475	regulation of cytoskeleton organization	GO:0051493	2.39E-01	2.89E-01	3	6
5,6	VTN|2.01125	positive regulation of cell-substrate adhesion	GO:0010811	2.39E-01	2.91E-01	1	6
6	SNW1|0.473175	cellular response to	GO:0097306	2.39E-01	2.91E-01	1	6
5,7,8,9	EIF4H|0.275675	regulation of translational initiation	GO:0006446	2.39E-01	2.91E-01	1	9

4,6,7	ATG7|2.120975;MAPK14|0.483325	positive regulation of secretion	GO:0051047	2.39E-01	2.91E-01	2	7
7,8	HSPA8|0.3721;PABPC1|0.3285;SNW1|0.473175	mRNA processing	GO:0006397	2.39E-01	2.91E-01	3	8
6	HSP90AB1|0.458775;PSME3|0.401575;SQSTM1|6.007525;TTK|0.488375;PLK1|0.415475	cellular protein catabolic process	GO:0044257	2.40E-01	2.91E-01	5	6
5	HSPD1|2.88995	interleukin-6 production	GO:0032635	2.41E-01	2.92E-01	1	5
4	GMPS|0.496875	pigment metabolic process	GO:0042440	2.41E-01	2.92E-01	1	4
6,7	ATG7|2.120975	cellular response to nitrogen levels	GO:0043562	2.41E-01	2.92E-01	1	7
5,6	HSPD1|2.88995	positive regulation of inflammatory response	GO:0050729	2.41E-01	2.92E-01	1	6
6,7,8	ATG7|2.120975	cellular response to nitrogen starvation	GO:0006995	2.41E-01	2.92E-01	1	8
5	HSPD1|2.88995	interferon-gamma production	GO:0032609	2.41E-01	2.92E-01	1	5
6,7,8,9	GSN|2.4555	negative regulation of protein polymerization	GO:0032272	2.41E-01	2.92E-01	1	9
5,6,7	PABPC1|0.3285	positive regulation of cellular amide metabolic process	GO:0034250	2.43E-01	2.92E-01	1	7
7	ALDH1L2|2.148	dicarboxylic acid metabolic process	GO:0043648	2.43E-01	2.95E-01	1	7
4,5,6,7	G3BP1|0.3904;PSME3|0.401575;MAPK14|0.483325;PLEK|2.071125;SOD2|2.175425	negative regulation of signal transduction	GO:0009968	2.45E-01	2.95E-01	5	7
5	MAPK14|0.483325;VTN|2.01125	positive regulation of cell migration	GO:0030335	2.45E-01	2.96E-01	2	5
6,7	MTHFD1L|6.667175	embryonic skeletal system morphogenesis	GO:0048704	2.45E-01	2.97E-01	1	7
5,6,7	S100A11|2.531875	negative regulation of DNA metabolic process	GO:0051053	2.45E-01	2.97E-01	1	7
5,7,8	MAPK14|0.483325	regulation of endothelial cell migration	GO:0010594	2.47E-01	2.97E-01	1	8
6	SLC2A1|5.081875	water-soluble vitamin metabolic process	GO:0006767	2.47E-01	2.99E-01	1	6
6,7,8,9	PSME3|0.401575	negative regulation of extrinsic apoptotic signaling pathway	GO:2001237	2.47E-01	2.99E-01	1	9
6,7	G3BP1|0.3904;PSME3|0.401575;MAPK14|0.483325	Wnt signaling pathway	GO:0016055	2.48E-01	2.99E-01	3	7
6	MTHFD1L|6.667175	neural tube closure	GO:0001843	2.49E-01	2.99E-01	1	6
6,7	HSPD1|2.88995	B cell proliferation	GO:0042100	2.52E-01	3.01E-01	1	7
5	MTHFD1L|6.667175	tube closure	GO:0060606	2.52E-01	3.03E-01	1	5
6,7,9	GSN|2.4555	negative regulation of protein depolymerization	GO:1901880	2.54E-01	3.03E-01	1	9
5	ASPH|2.59965	positive regulation of ion transmembrane transport	GO:0034767	2.54E-01	3.05E-01	1	5
4,5	MAPK14|0.483325;VTN|2.01125	positive regulation of cell motility	GO:2000147	2.54E-01	3.05E-01	2	5
4	ATG7|2.120975	post-embryonic development	GO:0009791	2.56E-01	3.06E-01	1	4
5	MAPK14|0.483325	erythrocyte differentiation	GO:0030218	2.56E-01	3.07E-01	1	5
3	STAT3|0.4995	feeding behavior	GO:0007631	2.56E-01	3.07E-01	1	3
8,9,10,11	HSP90AB1|0.458775;MAPK14|0.483325	positive regulation of protein serine/threonine kinase activity	GO:0071902	2.57E-01	3.07E-01	2	11
7	SOS2|11.48525	regulation of T cell differentiation	GO:0045580	2.58E-01	3.09E-01	1	7
5	MAPK14|0.483325	regulation of striated muscle tissue	GO:0016202	2.60E-01	3.09E-01	1	5
6,8	MTHFD1L|6.667175	primary neural tube formation	GO:0014020	2.60E-01	3.11E-01	1	8
6	MAPK14|0.483325	chondrocyte	GO:0002062	2.60E-01	3.11E-01	1	6
5,6,8	GSN|2.4555	negative regulation of protein complex disassembly	GO:0043242	2.60E-01	3.11E-01	1	8
8	ASPH|2.59965	cardiac muscle contraction	GO:0060048	2.60E-01	3.11E-01	1	8
6	HSP90AB1|0.458775;ENO1|2.1939;CKB|0.486625;YWHAG|2.359475;BAZ1B|0.460625;ALDOC|2.04725;LAMTOR2|7.841025;SQSTM1|6.007525;MAPK14|0.483325;STAT3|0.4995;VTN|2.01125;TTK|0.488375;PLK1|0.415475	phosphorylation	GO:0016310	2.60E-01	3.11E-01	13	6
4	VIM|2.241075;ASPH|2.59965;GSN|2.4555;ATG7|2.120975;MYH10|5.579275;GCH1|4.01735;SOD2|2.175425;PTP4A3|0.3433	system process	GO:0003008	2.60E-01	3.11E-01	8	4
7,8,10	PRDX5|3.0143	negative regulation of cysteine-type endopeptidase activity involved in apoptotic process	GO:0043154	2.62E-01	3.12E-01	1	10
4	AKR1B1|2.235325	cellular hormone metabolic process	GO:0034754	2.62E-01	3.13E-01	1	4
4	MAPK14|0.483325	regulation of muscle tissue development	GO:1901861	2.62E-01	3.13E-01	1	4
5,7	MAPK14|0.483325	regulation of fat cell differentiation	GO:0045598	2.62E-01	3.13E-01	1	7
7,8,9,10	HSP90AB1|0.458775;LAMTOR2|7.841025;MAPK14|0.483325	positive regulation of protein kinase activity	GO:0045860	2.62E-01	3.13E-01	3	10
4	MAPK14|0.483325;VTN|2.01125	positive regulation of cellular component movement	GO:0051272	2.62E-01	3.13E-01	2	4

6,7	ATP6V1B2|2.0575;SLC2A1|5.081875	proton transport	GO:0015992	2.63E-01	3.13E-01	2	7
7,8,9,10	ASPH|2.59965	regulation of cation channel activity	GO:2001257	2.64E-01	3.14E-01	1	10
5,6	SNW1|0.473175	regulation of cellular response to transforming growth factor beta	GO:1903844	2.64E-01	3.14E-01	1	6
6,7,8	SNW1|0.473175	regulation of transforming growth factor beta receptor signaling	GO:0017015	2.64E-01	3.14E-01	1	8
4,5,6	VTN|2.01125	positive regulation of endocytosis	GO:0045807	2.64E-01	3.14E-01	1	6
5,6	MAPK14|0.483325	regulation of muscle organ development	GO:0048634	2.64E-01	3.14E-01	1	6
7,8,9	SQSTM1|6.007525;SOS2|11.48525	regulation of Ras protein signal transduction	GO:0046578	2.64E-01	3.14E-01	2	9
5,6	ATP6V1B2|2.0575;SLC2A1|5.081875	hydrogen transport	GO:0006818	2.65E-01	3.15E-01	2	6
6	VIM|2.241075;ASPH|2.59965	muscle contraction	GO:0006936	2.65E-01	3.16E-01	2	6
4	ASPH|2.59965	positive regulation of transmembrane transport	GO:0034764	2.66E-01	3.16E-01	1	4
4	HIST1H4A|0.23835	negative regulation of hemopoiesis	GO:1903707	2.66E-01	3.16E-01	1	4
4,7,8	YWHAG|2.359475	positive regulation of establishment of protein localization to mitochondrion	GO:1903749	2.66E-01	3.16E-01	1	8
9,10	ASPH|2.59965;ERO1L|2.370575	cellular calcium ion homeostasis	GO:0006874	2.66E-01	3.16E-01	2	10
8,9	PRDX5|3.0143	negative regulation of cysteine-type endopeptidase activity	GO:2000117	2.68E-01	3.16E-01	1	9
7	MAPK14|0.483325	myotube differentiation	GO:0014902	2.68E-01	3.17E-01	1	7
5	ZNF706|0.3974	regulation of stem cell differentiation	GO:2000736	2.68E-01	3.17E-01	1	5
6	HSPD1|2.88995	regulation of adaptive immune response based on somatic recombination of immune receptors built from immunoglobulin superfamily domains	GO:0002822	2.68E-01	3.17E-01	1	6
8,9	STAT3|0.4995	purine ribonucleoside triphosphate biosynthetic process	GO:0009206	2.68E-01	3.17E-01	1	9
4	LCP1|2.170475;MTHFD1L|6.667175;GSN|2.4555	tissue morphogenesis	GO:0048729	2.68E-01	3.17E-01	3	4
4	TUBG1|0.4102;NDE1|0.446425;TTK|0.488375;PLK1|0.415475;CETN3|0.4171	microtubule-based process	GO:0007017	2.69E-01	3.17E-01	5	4
7,8	STAT3|0.4995	purine nucleoside triphosphate biosynthetic process	GO:0009145	2.70E-01	3.19E-01	1	8
5	GSN|2.4555	renal system process	GO:0003014	2.70E-01	3.19E-01	1	5
5,6,7	HSP90AB1|0.458775	regulation of cytokine-mediated signaling	GO:0001959	2.70E-01	3.19E-01	1	7
4,6,7	LIG1|0.440425;BAZ1B|0.460625;PCNA|0.404975;CHAF1B|0.2723	DNA repair	GO:0006281	2.70E-01	3.19E-01	4	7
5	MAPK14|0.483325;SNW1|0.473175	muscle organ	GO:0007517	2.71E-01	3.19E-01	2	5
3	MAPK14|0.483325;VTN|2.01125	positive regulation of locomotion	GO:0040017	2.71E-01	3.20E-01	2	3
7,8,9	PSME3|0.401575	negative regulation of G1/S transition of mitotic cell cycle	GO:2000134	2.72E-01	3.20E-01	1	9
5,7,8	ASPH|2.59965	positive regulation of calcium ion transport	GO:0051928	2.72E-01	3.21E-01	1	8
5,6,7,8	PLEK|2.071125	negative regulation of G-protein coupled receptor protein signaling pathway	GO:0045744	2.74E-01	3.21E-01	1	8
4	TFRC|0.403125	response to transition metal nanoparticle	GO:1990267	2.74E-01	3.22E-01	1	4
6	PCNA|0.404975	cellular response to light stimulus	GO:0071482	2.74E-01	3.22E-01	1	6
5	MAPK14|0.483325	regulation of cyclase activity	GO:0031279	2.74E-01	3.22E-01	1	5
4	MAPK14|0.483325	erythrocyte homeostasis	GO:0034101	2.74E-01	3.22E-01	1	4
8,10,11	MAPK14|0.483325	toll-like receptor 4 signaling pathway	GO:0034142	2.74E-01	3.22E-01	1	11
7	BTF3|0.2449;MYH10|5.579275	in utero embryonic development	GO:0001701	2.74E-01	3.22E-01	2	7
5	HSPD1|2.88995	protein stabilization	GO:0050821	2.76E-01	3.23E-01	1	5
8	GTF2E1|0.300475	transcription elongation from RNA polymerase II promoter	GO:0006368	2.76E-01	3.24E-01	1	8
4,5	HSPD1|2.88995;MAPK14|0.483325	positive regulation of cytokine production	GO:0001819	2.77E-01	3.24E-01	2	5
6,8,10	VIM|2.241075;ARHGDIA|3.8215	regulation of neuron projection development	GO:0010975	2.77E-01	3.26E-01	2	10
7,8	PSME3|0.401575	negative regulation of cell cycle G1/S phase	GO:1902807	2.78E-01	3.26E-01	1	8
7,9	PSME3|0.401575	stimulatory C-type lectin receptor signaling	GO:0002223	2.78E-01	3.26E-01	1	9

7,8	YWHAG|2.359475;PLK1|0.415475	negative regulation of protein phosphorylation	GO:0001933	2.80E-01	3.26E-01	2	8
7,8	STAT3|0.4995	ribonucleoside triphosphate biosynthetic	GO:0009201	2.80E-01	3.28E-01	1	8
5,6	HSPD1|2.88995	regulation of type I interferon production	GO:0032479	2.80E-01	3.28E-01	1	6
6	PRDX5|3.0143	collagen metabolic	GO:0032963	2.80E-01	3.28E-01	1	6
6	MTHFD1L|6.667175;BTF3|0.2449;MYH10|5.579275	chordate embryonic development	GO:0043009	2.81E-01	3.28E-01	3	6
8,9	GMPS|0.496875;STAT3|0.4995	purine ribonucleotide biosynthetic process	GO:0009152	2.82E-01	3.29E-01	2	9
6	PSME3|0.401575	regulation of cell cycle arrest	GO:0071156	2.82E-01	3.29E-01	1	6
4	HSP90AB1|0.458775	regulation of response to cytokine stimulus	GO:0060759	2.82E-01	3.29E-01	1	4
2	ALB|0.23405	cell killing	GO:0001906	2.82E-01	3.29E-01	1	2
6,7	LAMTOR2|7.841025;RHOG|2.449775;ARHGDIA|3.8215;SOS2|11.48525	positive regulation of GTPase activity	GO:0043547	2.82E-01	3.29E-01	4	7
4	HSPD1|2.88995;VTN|2.01125	positive regulation of cell adhesion	GO:0045785	2.83E-01	3.30E-01	2	4
9	ASPH|2.59965;ERO1L|2.370575	calcium ion homeostasis	GO:0055074	2.84E-01	3.30E-01	2	9
6,8	PSME3|0.401575	innate immune response activating cell surface receptor signaling	GO:0002220	2.84E-01	3.30E-01	1	8
5,7	MTHFD1L|6.667175	neural tube formation	GO:0001841	2.84E-01	3.30E-01	1	7
4	HSPD1|2.88995	immunoglobulin	GO:0002377	2.84E-01	3.30E-01	1	4
6	HSPD1|2.88995	regulation of lymphocyte mediated immunity	GO:0002706	2.84E-01	3.30E-01	1	6
4	VTN|2.01125	formation of primary germ layer	GO:0001704	2.84E-01	3.30E-01	1	4
4	MTHFD1L|6.667175;BAZ1B|0.460625;MAPK14|0.483325;STAT3|0.4995	organ morphogenesis	GO:0009887	2.84E-01	3.30E-01	4	4
6,7,8,9	HSP90AB1|0.458775;LAMTOR2|7.841025;MAPK14|0.483325	positive regulation of kinase activity	GO:0033674	2.86E-01	3.31E-01	3	9
5,6,7	PLK1|0.415475	positive regulation of mitotic cell cycle	GO:0045931	2.86E-01	3.32E-01	1	7
5	HSPD1|2.88995	type I interferon	GO:0032606	2.86E-01	3.32E-01	1	5
7,8,9	PLK1|0.415475	positive regulation of proteasomal protein catabolic process	GO:1901800	2.86E-01	3.32E-01	1	9
5	HSPD1|2.88995	regulation of adaptive immune response	GO:0002819	2.86E-01	3.32E-01	1	5
5	MTHFD1L|6.667175;BTF3|0.2449;MYH10|5.579275	embryo development ending in birth or egg hatching	GO:0009792	2.87E-01	3.32E-01	3	5
7,8,10	GSN|2.4555	actin nucleation	GO:0045010	2.88E-01	3.33E-01	1	10
7	PRDX5|3.0143	transcription from RNA polymerase III promoter	GO:0006383	2.88E-01	3.33E-01	1	7
8,9	ASPH|2.59965;ERO1L|2.370575	cellular divalent inorganic cation homeostasis	GO:0072503	2.88E-01	3.33E-01	2	9
7	GSN|2.4555;PLEK|2.071125	actin polymerization or depolymerization	GO:0008154	2.89E-01	3.33E-01	2	7
6	PLEK|2.071125	vesicle docking involved in exocytosis	GO:0006904	2.89E-01	3.35E-01	1	6
6,7	YWHAG|2.359475	regulation of establishment of protein localization to	GO:1903747	2.89E-01	3.35E-01	1	7
5,6,7,8	AKR1B1|2.235325;LAMTOR2|7.841025;MAPK14|0.483325;SOS2|11.48525	positive regulation of intracellular signal transduction	GO:1902533	2.89E-01	3.35E-01	4	8
5,7	NDRG1|10.0073	cellular response to	GO:0071456	2.91E-01	3.35E-01	1	7
6,7,8	TXNIP|2.0553	keratinocyte	GO:0030216	2.91E-01	3.37E-01	1	8
6,7,8	AKR1B1|2.235325	regulation of JAK-STAT cascade	GO:0046425	2.91E-01	3.37E-01	1	8
5	PRDX5|3.0143	multicellular organismal macromolecule metabolic process	GO:0044259	2.91E-01	3.37E-01	1	5
5	ATG7|2.120975	liver development	GO:0001889	2.91E-01	3.37E-01	1	5
6	VDAC3|2.015475;GSN|2.4555	cilium morphogenesis	GO:0060271	2.92E-01	3.37E-01	2	6
7,8	PRDX5|3.0143;VTN|2.01125	negative regulation of endopeptidase activity	GO:0010951	2.92E-01	3.37E-01	2	8
8,9	PABPC1|0.3285	mRNA 3'-end processing	GO:0031124	2.93E-01	3.37E-01	1	9
6,7	PSME3|0.401575	mitotic DNA damage checkpoint	GO:0044773	2.95E-01	3.38E-01	1	7
7,8	GMPS|0.496875;STAT3|0.4995	ribonucleotide biosynthetic process	GO:0009260	2.96E-01	3.41E-01	2	8
5	HSP90AB1|0.458775;ASPH|2.59965;DNMT1|0.172;HIST1H4A|0.23835;YWHAG|2.359475;ERO1L|2.370575;BAZ1B|0.460625;PSME3|0.401575;LAMTOR2|7.841025;SQSTM1|6.007525;ATG7|2.120975;MAPK14|0.483325;HIST1H3A|0.455625;SNW1|0.473175;PLEK|2.071125;VTN|2.01125;H3F3A|0.20985;TTK|0.488375;ASL|2.252975;PLK1|0.415	macromolecule modification	GO:0043412	2.97E-01	3.41E-01	21	5
7,8,9	HSPD1|2.88995	activation of cysteine-type endopeptidase activity involved in apoptotic process	GO:0006919	2.97E-01	3.42E-01	1	9
5,6	GSN|2.4555	maintenance of protein location in cell	GO:0032507	2.97E-01	3.42E-01	1	6

5	ATG7|2.120975	hepaticobiliary system development	GO:0061008	2.97E-01	3.42E-01	1	5
6	NDRG1|10.0073	cellular response to decreased oxygen levels	GO:0036294	2.97E-01	3.42E-01	1	6
7	GMPS|0.496875;STAT3|0.4995	purine nucleotide biosynthetic process	GO:0006164	2.99E-01	3.42E-01	2	7
6	STAT3|0.4995	nucleotide catabolic	GO:0009166	2.99E-01	3.43E-01	1	6
6	GMPS|0.496875;STAT3|0.4995	ribose phosphate biosynthetic process	GO:0046390	3.00E-01	3.44E-01	2	6
6,7,8	STAT3|0.4995	positive regulation of nucleotide biosynthetic process	GO:0030810	3.01E-01	3.44E-01	1	8
7,8,9	STAT3|0.4995	positive regulation of purine nucleotide biosynthetic process	GO:1900373	3.01E-01	3.46E-01	1	9
3,4	G3BP1|0.3904;PSME3|0.401575;MAPK14|0.483325;PLEK|2.071125;SOD2|2.175425	negative regulation of signaling	GO:0023057	3.04E-01	3.46E-01	5	4
6	ATG7|2.120975	nucleophagy	GO:0044804	3.05E-01	3.49E-01	1	6
5,6	ASPH|2.59965;ATP6V1B2|2.0575;NSF|2.017425;VDAC3|2.015475;SLC25A1|2.1121;ERO1L|2.370575;TFRC|0.403125;SLC2A1|5.081875;HBA1|2.13735;ALB|0.23405	ion transport	GO:0006811	3.07E-01	3.50E-01	10	6
6	PLP1|3.936	long-chain fatty acid metabolic process	GO:0001676	3.09E-01	3.52E-01	1	6
3	MAPK14|0.483325	myeloid cell homeostasis	GO:0002262	3.09E-01	3.53E-01	1	3
5,6,9,10,11	PLK1|0.415475	regulation of cyclin-dependent protein serine/threonine kinase activity	GO:0000079	3.09E-01	3.53E-01	1	11
6,7,8,9	PSME3|0.401575	positive regulation of canonical Wnt signaling pathway	GO:0090263	3.09E-01	3.53E-01	1	9
4,5	G3BP1|0.3904;PSME3|0.401575;MAPK14|0.483325;PLEK|2.071125;SOD2|2.175425	negative regulation of cell communication	GO:0010648	3.09E-01	3.53E-01	5	5
6,7	PRDX5|3.0143;VTN|2.01125	negative regulation of peptidase activity	GO:0010466	3.10E-01	3.53E-01	2	7
4,5	GSN|2.4555	maintenance of protein location	GO:0045185	3.11E-01	3.54E-01	1	5
4	CCT7|2.06135	cell recognition	GO:0008037	3.11E-01	3.55E-01	1	4
4,5	H3F3A|0.20985	positive regulation of cell growth	GO:0030307	3.11E-01	3.55E-01	1	5
4	NDE1|0.446425	neural precursor cell proliferation	GO:0061351	3.11E-01	3.55E-01	1	4
8	ASPH|2.59965;ERO1L|2.370575	divalent inorganic cation homeostasis	GO:0072507	3.12E-01	3.55E-01	2	8
6,7	STAT3|0.4995	nucleoside triphosphate biosynthetic process	GO:0009142	3.12E-01	3.56E-01	1	7
6	SNW1|0.473175	intrinsic apoptotic signaling pathway in response to DNA damage	GO:0008630	3.12E-01	3.56E-01	1	6
6	ATP6V1B2|2.0575;STAT3|0.4995	cellular response to peptide hormone stimulus	GO:0071375	3.13E-01	3.56E-01	2	6
6	MTHFD1L|6.667175	embryonic skeletal system development	GO:0048706	3.14E-01	3.56E-01	1	6
5	HSPD1|2.88995	response to alkaloid	GO:0043279	3.14E-01	3.58E-01	1	5
5	CCT7|2.06135	single fertilization	GO:0007338	3.16E-01	3.58E-01	1	5
6	MAPK14|0.483325	cellular response to lipopolysaccharide	GO:0071222	3.16E-01	3.60E-01	1	6
5	STAT3|0.4995	nucleoside phosphate catabolic process	GO:1901292	3.16E-01	3.60E-01	1	5
7,8	ASPH|2.59965;ATP6V1B2|2.0575;NSF|2.017425;ERO1L|2.370575;TFRC|0.403125	metal ion transport	GO:0030001	3.18E-01	3.60E-01	5	8
4	PRDX5|3.0143	multicellular organismal metabolic process	GO:0044236	3.18E-01	3.61E-01	1	4
4,5	KRT1|0.4198	negative regulation of defense response	GO:0031348	3.20E-01	3.62E-01	1	5
6	MTHFD1L|6.667175	embryonic epithelial tube formation	GO:0001838	3.22E-01	3.64E-01	1	6
5,6	MAPK14|0.483325	regulation of cytokine secretion	GO:0050707	3.22E-01	3.65E-01	1	6
5	PLK1|0.415475	nucleus organization	GO:0006997	3.22E-01	3.65E-01	1	5
7	PSME3|0.401575	antigen processing and presentation of exogenous peptide antigen via MHC class I, TAP-dependent	GO:0002479	3.22E-01	3.65E-01	1	7
5,6,7,8	MTHFD1L|6.667175	epithelial tube formation	GO:0072175	3.24E-01	3.65E-01	1	8
4,5,6	ERO1L|2.370575	cell redox homeostasis	GO:0045454	3.24E-01	3.67E-01	1	6
4,5	MAPK14|0.483325	organ growth	GO:0035265	3.24E-01	3.67E-01	1	5
6	LAMTOR2|7.841025;RHOG|2.449775;ARHGDIA|3.8215;SOS2|11.48525	regulation of GTPase activity	GO:0043087	3.24E-01	3.67E-01	4	6
4	SLC25A1|2.1121;ISYNA1|0.49065;MAPK14|0.483325;PLEK|2.071125;PLP1|3.936	cellular lipid metabolic process	GO:0044255	3.25E-01	3.67E-01	5	4
5,7	MYH10|5.579275	retina development in camera-type eye	GO:0060041	3.26E-01	3.68E-01	1	7
6,7,8	MAPK14|0.483325;SLC2A1|5.081875	regulation of protein secretion	GO:0050708	3.26E-01	3.68E-01	2	8
5	SLC2A1|5.081875	vitamin metabolic process	GO:0006766	3.27E-01	3.68E-01	1	5
7	SLC25A1|2.1121	triglyceride metabolic process	GO:0006641	3.27E-01	3.70E-01	1	7

5,6	ERO1L|2.370575	endoplasmic reticulum unfolded protein response	GO:0030968	3.27E-01	3.70E-01	1	6
5	MAPK14|0.483325	cellular response to molecule of bacterial	GO:0071219	3.29E-01	3.70E-01	1	5
7	ARHGDIA|3.8215	regulation of	GO:0050770	3.29E-01	3.71E-01	1	7
6	PSME3|0.401575	antigen processing and presentation of exogenous peptide antigen via MHC class I	GO:0042590	3.29E-01	3.71E-01	1	6
7,8	SOS2|11.48525	regulation of T cell proliferation	GO:0042129	3.29E-01	3.71E-01	1	8
6,7	ERO1L|2.370575	cellular response to unfolded protein	GO:0034620	3.33E-01	3.71E-01	1	7
5,6	GSN|2.4555	negative regulation of protein complex assembly	GO:0031333	3.35E-01	3.75E-01	1	6
5,6	ARHGDIA|3.8215	positive regulation of cell morphogenesis involved in differentiation	GO:0010770	3.37E-01	3.77E-01	1	6
6	GMPS|0.496875;STAT3|0.4995	purine-containing compound biosynthetic process	GO:0072522	3.37E-01	3.79E-01	2	6
6,7	HSPA8|0.3721;NSF|2.017425	Golgi vesicle transport	GO:0048193	3.38E-01	3.79E-01	2	7
6	SOS2|11.48525	regulation of lymphocyte differentiation	GO:0045619	3.38E-01	3.80E-01	1	6
7	ASPH|2.59965	striated muscle	GO:0006941	3.38E-01	3.80E-01	1	7
4,6	HSP90AB1|0.458775;MAPK14|0.483325	reproductive structure development	GO:0048608	3.39E-01	3.80E-01	2	6
5,6	ASPH|2.59965	limb morphogenesis	GO:0035108	3.40E-01	3.81E-01	1	6
4	NDE1|0.446425	stem cell proliferation	GO:0072089	3.40E-01	3.82E-01	1	4
4	ASPH|2.59965	appendage morphogenesis	GO:0035107	3.40E-01	3.82E-01	1	4
6	PLEK|2.071125	regulation of phosphatase activity	GO:0010921	3.40E-01	3.82E-01	1	6
5	NSF|2.017425	receptor metabolic process	GO:0043112	3.42E-01	3.82E-01	1	5
4,5	MAPK14|0.483325	positive regulation of developmental growth	GO:0048639	3.42E-01	3.83E-01	1	5
7,8	PSME3|0.401575	regulation of G1/S transition of mitotic cell	GO:2000045	3.42E-01	3.83E-01	1	8
5	MAPK14|0.483325	regulation of muscle cell differentiation	GO:0051147	3.42E-01	3.83E-01	1	5
6	SLC25A1|2.1121	acylglycerol metabolic process	GO:0006639	3.42E-01	3.83E-01	1	6
5	HSP90AB1|0.458775;MAPK14|0.483325	reproductive system development	GO:0061458	3.43E-01	3.83E-01	2	5
5	SLC25A1|2.1121	neutral lipid metabolic process	GO:0006638	3.44E-01	3.84E-01	1	5
5,6	PLK1|0.415475	regulation of microtubule cytoskeleton organization	GO:0070507	3.44E-01	3.85E-01	1	6
4	ATG7|2.120975	adult behavior	GO:0030534	3.46E-01	3.85E-01	1	4
4,6	MAPK14|0.483325	regulation of epithelial cell migration	GO:0010632	3.46E-01	3.86E-01	1	6
5	PLP1|3.936	cell maturation	GO:0048469	3.46E-01	3.86E-01	1	5
7	STAT3|0.4995	positive regulation of purine nucleotide metabolic process	GO:1900544	3.47E-01	3.86E-01	1	7
4	HSPD1|2.88995	positive regulation of immune effector process	GO:0002699	3.47E-01	3.87E-01	1	4
6,7,8	VDAC3|2.015475	regulation of anion	GO:0044070	3.47E-01	3.87E-01	1	8
4,5	STAT3|0.4995	multicellular organism growth	GO:0035264	3.47E-01	3.87E-01	1	5
5,6	ERO1L|2.370575	cellular response to topologically incorrect protein	GO:0035967	3.47E-01	3.87E-01	1	6
5	MAPK14|0.483325;PLP1|3.936	fatty acid metabolic process	GO:0006631	3.48E-01	3.87E-01	2	5
5	ALDH1L2|2.148;ASL|2.252975	organonitrogen compound catabolic process	GO:1901565	3.48E-01	3.87E-01	2	5
6,7	STAT3|0.4995	positive regulation of nucleotide metabolic process	GO:0045981	3.49E-01	3.87E-01	1	7
4	MTHFD1L|6.667175	tube formation	GO:0035148	3.49E-01	3.88E-01	1	4
4,5,6	PLK1|0.415475	positive regulation of cell division	GO:0051781	3.49E-01	3.88E-01	1	6
8,9,10	VTN|2.01125	positive regulation of peptidyl-tyrosine phosphorylation	GO:0050731	3.51E-01	3.88E-01	1	10
6	PCNA|0.404975	response to UV	GO:0009411	3.51E-01	3.90E-01	1	6
6,7,8	SOD2|2.175425	regulation of intrinsic apoptotic signaling	GO:2001242	3.51E-01	3.90E-01	1	8
5,6	PCNA|0.404975	regulation of response to DNA damage stimulus	GO:2001020	3.53E-01	3.90E-01	1	6
5,6	GSN|2.4555;PLEK|2.071125	regulation of actin cytoskeleton organization	GO:0032956	3.53E-01	3.92E-01	2	6
7	PSME3|0.401575	regulation of cell cycle G1/S phase transition	GO:1902806	3.54E-01	3.93E-01	1	7
5,6,7	AKR1B1|2.235325;MAPK14|0.483325	stress-activated protein kinase signaling cascade	GO:0031098	3.55E-01	3.93E-01	2	7
5	MAPK14|0.483325	cytokine secretion	GO:0050663	3.58E-01	3.94E-01	1	5
6	STAT3|0.4995	eye morphogenesis	GO:0048592	3.58E-01	3.97E-01	1	6

6	MAPK14|0.483325;SNW1|0.473175	cellular response to lipid	GO:0071396	3.58E-01	3.97E-01	2	6
7	GTF2E1|0.300475	DNA-templated transcription, elongation	GO:0006354	3.60E-01	3.97E-01	1	7
7,8	HSP90AB1|0.458775	interferon-gamma-mediated signaling	GO:0060333	3.63E-01	3.99E-01	1	8
6	PLK1|0.415475	spindle assembly	GO:0051225	3.63E-01	4.02E-01	1	6
6,7	STAT3|0.4995	regulation of nucleotide biosynthetic process	GO:0030808	3.67E-01	4.02E-01	1	7
5,6,7,8	PSME3|0.401575	positive regulation of Wnt signaling pathway	GO:0030177	3.67E-01	4.05E-01	1	8
7,8	STAT3|0.4995	regulation of purine nucleotide biosynthetic process	GO:1900371	3.67E-01	4.05E-01	1	8
5	HSPD1|2.88995	regulation of leukocyte mediated immunity	GO:0002703	3.68E-01	4.05E-01	1	5
5,6,7	PLEK|2.071125;SOD2|2.175425	negative regulation of intracellular signal transduction	GO:1902532	3.69E-01	4.07E-01	2	7
6,7	SLC25A1|2.1121;ALB|0.23405	carboxylic acid transport	GO:0046942	3.69E-01	4.08E-01	2	7
5	VTN|2.01125	regulation of cell-substrate adhesion	GO:0010810	3.70E-01	4.08E-01	1	5
6,8,9	LIG1|0.440425	double-strand break repair via homologous recombination	GO:0000724	3.70E-01	4.08E-01	1	9
6,7,8,9	PSME3|0.401575	regulation of extrinsic apoptotic signaling	GO:2001236	3.72E-01	4.08E-01	1	9
5,7,8	LIG1|0.440425	recombinational repair	GO:0000725	3.72E-01	4.10E-01	1	8
4	KRT1|0.4198;MAPK14|0.483325	angiogenesis	GO:0001525	3.72E-01	4.10E-01	2	4
4	CNDP2|2.0938;SLC25A1|2.1121	sulfur compound metabolic process	GO:0006790	3.73E-01	4.10E-01	2	4
6	NDE1|0.446425	microtubule-based	GO:0010970	3.74E-01	4.11E-01	1	6
6	AKR1B1|2.235325	steroid biosynthetic	GO:0006694	3.74E-01	4.11E-01	1	6
5,6	SLC25A1|2.1121;ALB|0.23405	organic acid transport	GO:0015849	3.74E-01	4.11E-01	2	6
5	MTHFD1L|6.667175	morphogenesis of embryonic epithelium	GO:0016331	3.75E-01	4.12E-01	1	5
5	ASPH|2.59965	limb development	GO:0060173	3.77E-01	4.13E-01	1	5
5	MAPK14|0.483325	response to ionizing radiation	GO:0010212	3.77E-01	4.14E-01	1	5
4	ASPH|2.59965	appendage development	GO:0048736	3.77E-01	4.14E-01	1	4
6,8	ASPH|2.59965	regulation of heart contraction	GO:0008016	3.79E-01	4.14E-01	1	8
6	GCH1|4.01735	response to tumor necrosis factor	GO:0034612	3.80E-01	4.16E-01	1	6
4,6	MAPK14|0.483325	positive regulation of hemopoiesis	GO:1903708	3.84E-01	4.18E-01	1	6
6,7	SOS2|11.48525	T cell proliferation	GO:0042098	3.84E-01	4.21E-01	1	7
7,8	PLEK|2.071125	regulation of protein dephosphorylation	GO:0035304	3.84E-01	4.21E-01	1	8
8,9,10	PLK1|0.415475	regulation of cyclin-dependent protein kinase activity	GO:1904029	3.84E-01	4.21E-01	1	10
4	MTHFD1L|6.667175	neural tube development	GO:0021915	3.86E-01	4.21E-01	1	4
5,7,8	NSF|2.017425;MAPK14|0.483325;SLC2A1|5.081875	regulation of secretion by cell	GO:1903530	3.86E-01	4.22E-01	3	8
3	HSPD1|2.88995;ASPH|2.59965;PCNA|0.404975	detection of stimulus	GO:0051606	3.87E-01	4.23E-01	3	3
9	PSME3|0.401575;ATG7|2.120975;TTK|0.488375;PLK1|0.415475	protein ubiquitination	GO:0016567	3.88E-01	4.23E-01	4	9
4	CCT7|2.06135	fertilization	GO:0009566	3.89E-01	4.25E-01	1	4
4,5	PLK1|0.415475	regulation of microtubule-based process	GO:0032886	3.91E-01	4.25E-01	1	5
3	HSPD1|2.88995	production of molecular mediator of immune response	GO:0002440	3.94E-01	4.27E-01	1	3
7	PLEK|2.071125	regulation of dephosphorylation	GO:0035303	3.94E-01	4.30E-01	1	7
6,7	ASPH|2.59965	regulation of muscle contraction	GO:0006937	3.96E-01	4.30E-01	1	7
6	PLP1|3.936	fatty acid biosynthetic process	GO:0006633	3.96E-01	4.32E-01	1	6
4	NDE1|0.446425;MYH10|5.579275	establishment of organelle localization	GO:0051656	3.96E-01	4.32E-01	2	4
8,9,10,11	LAMTOR2|7.841025;MAPK14|0.483325	activation of protein kinase activity	GO:0032147	3.98E-01	4.32E-01	2	11
5,11	ATG7|2.120975	histone acetylation	GO:0016573	4.01E-01	4.34E-01	1	11
7	TXNIP|2.0553	epidermal cell differentiation	GO:0009913	4.02E-01	4.37E-01	1	7
4	AKR1B1|2.235325	female pregnancy	GO:0007565	4.02E-01	4.38E-01	1	4
6,7	NDE1|0.446425	cytoskeleton-dependent intracellular transport	GO:0030705	4.02E-01	4.38E-01	1	7
5	PSME3|0.401575	antigen processing and presentation of peptide antigen via MHC class I	GO:0002474	4.02E-01	4.38E-01	1	5
5	HSP90AB1|0.458775;ASPH|2.59965;DNMT1|0.172;HIST1H4A|0.23835;YWHAG|2.359475;ERO1L|2.370575;BAZ1B|0.460625;PSME3|0.401575;LAMTOR2|7.841025;SQSTM1|6.007525;ATG7|2.120975;MAPK14|0.483325;SNW1|0.473175;PLEK|2.071125;VTN|2.01125;TTK|0.488375;ASL|2.252975;PLK1|0.415475;PTP4A3|0.3433	protein modification process	GO:0036211	4.03E-01	4.38E-01	19	5

6	HSP90AB1|0.458775;ASPH|2.59965;DNMT1|0.172;HIST1H4A|0.23835;YWHAG|2.359475;ERO1L|2.370575;BAZ1B|0.460625;PSME3|0.401575;LAMTOR2|7.841025;SQSTM1|6.007525;ATG7|2.120975;MAPK14|0.483325;SNW1|0.473175;PLEK|2.071125;VTN|2.01125;TTK|0.488375;ASL|2.252975;PLK1|0.415475;PTP4A3|0.3433	cellular protein modification process	GO:0006464	4.03E-01	4.38E-01	19	6
4,5	GSN|2.4555;PLEK|2.071125	regulation of actin filament-based process	GO:0032970	4.03E-01	4.38E-01	2	5
6,7	GSN|2.4555	negative regulation of cytoskeleton organization	GO:0051494	4.04E-01	4.38E-01	1	7
7,8	ALB|0.23405	monocarboxylic acid transport	GO:0015718	4.04E-01	4.39E-01	1	8
5	VTN|2.01125	gastrulation	GO:0007369	4.07E-01	4.39E-01	1	5
10	ATG7|2.120975	internal peptidyl-lysine acetylation	GO:0018393	4.09E-01	4.42E-01	1	10
6,7,9	GSN|2.4555	positive regulation of actin filament polymerization	GO:0030838	4.09E-01	4.43E-01	1	9
6,7,9	ATG7|2.120975	central nervous system neuron differentiation	GO:0021953	4.10E-01	4.43E-01	1	9
5,6,7,8	MAPK14|0.483325	positive regulation of protein secretion	GO:0050714	4.10E-01	4.45E-01	1	8
8,9,10,11	MAPK14|0.483325	activation of MAPK activity	GO:0000187	4.12E-01	4.45E-01	1	11
7	ATG7|2.120975	protein lipidation	GO:0006497	4.12E-01	4.46E-01	1	7
5	ATP6V1B2|2.0575;STAT3|0.4995	response to peptide hormone	GO:0043434	4.16E-01	4.46E-01	2	5
9	ATG7|2.120975	peptidyl-lysine acetylation	GO:0018394	4.17E-01	4.51E-01	1	9
7	PLEK|2.071125;PTP4A3|0.3433	protein dephosphorylation	GO:0006470	4.18E-01	4.51E-01	2	7
5,6	SLC2A1|5.081875	energy reserve metabolic process	GO:0006112	4.20E-01	4.53E-01	1	6
6	ATG7|2.120975	lipoprotein biosynthetic process	GO:0042158	4.20E-01	4.54E-01	1	6
5	STAT3|0.4995	organophosphate catabolic process	GO:0046434	4.22E-01	4.54E-01	1	5
6,7,8,9,11	ARHGDIA|3.8215	positive regulation of neuron projection development	GO:0010976	4.22E-01	4.55E-01	1	11
7,8	PABPC1|0.3285	RNA 3'-end processing	GO:0031123	4.25E-01	4.55E-01	1	8
3	SRRT|0.39875;MAPK14|0.483325;SNW1|0.473175;ARHGDIA|3.8215	positive regulation of developmental process	GO:0051094	4.25E-01	4.59E-01	4	3
6,7,8	YWHAG|2.359475	establishment of protein localization to mitochondrion	GO:0072655	4.26E-01	4.59E-01	1	8
5,6,7	PLEK|2.071125	regulation of G-protein coupled receptor protein signaling pathway	GO:0008277	4.26E-01	4.60E-01	1	7
4,6	CNDP2|2.0938	xenobiotic metabolic process	GO:0006805	4.28E-01	4.60E-01	1	6
5,6,7,8	ASPH|2.59965;VDAC3|2.015475	regulation of ion transmembrane transport	GO:0034765	4.28E-01	4.61E-01	2	8
4	HSPD1|2.88995;ASPH|2.59965	detection of chemical stimulus	GO:0009593	4.28E-01	4.62E-01	2	4
5,6,7,8,9	GSN|2.4555	positive regulation of protein polymerization	GO:0032273	4.29E-01	4.62E-01	1	9
6,7	SOS2|11.48525	regulation of lymphocyte proliferation	GO:0050670	4.29E-01	4.62E-01	1	7
7	ASPH|2.59965	heart contraction	GO:0060047	4.29E-01	4.62E-01	1	7
3	TOP2A|0.481675	circadian rhythm	GO:0007623	4.31E-01	4.62E-01	1	3
6	SOS2|11.48525	regulation of mononuclear cell proliferation	GO:0032944	4.31E-01	4.63E-01	1	6
5,6	PSME3|0.401575	regulation of cellular ketone metabolic process	GO:0010565	4.31E-01	4.63E-01	1	6
5,6	MAPK14|0.483325	cartilage development	GO:0051216	4.31E-01	4.63E-01	1	6
4	PCNA|0.404975;MAPK14|0.483325	response to radiation	GO:0009314	4.31E-01	4.63E-01	2	4
5	SLC25A1|2.1121;PLEK|2.071125	glycerolipid metabolic process	GO:0046486	4.31E-01	4.63E-01	2	5
6	ASPH|2.59965	heart process	GO:0003015	4.33E-01	4.63E-01	1	6
5	CNDP2|2.0938	cellular response to xenobiotic stimulus	GO:0071466	4.34E-01	4.64E-01	1	5
7	YWHAG|2.359475	protein localization to mitochondrion	GO:0070585	4.36E-01	4.66E-01	1	7
6,7,8,9,10,11	SLC2A1|5.081875	regulation of insulin secretion	GO:0050796	4.36E-01	4.67E-01	1	11
4	AKR1B1|2.235325;SLC2A1|5.081875	regulation of hormone levels	GO:0010817	4.37E-01	4.67E-01	2	4
6,7,8,9	ASPH|2.59965	regulation of cation transmembrane transport	GO:1904062	4.39E-01	4.68E-01	1	9
3	ATG7|2.120975	locomotory behavior	GO:0007626	4.40E-01	4.70E-01	1	3
5	SOS2|11.48525	regulation of leukocyte proliferation	GO:0070663	4.42E-01	4.71E-01	1	5
3	AKR1B1|2.235325	hormone metabolic	GO:0042445	4.43E-01	4.73E-01	1	3
4,5	ASPH|2.59965;VDAC3|2.015475	regulation of transmembrane transport	GO:0034762	4.43E-01	4.74E-01	2	5
5	ISYNA1|0.49065;PLEK|2.071125	phospholipid metabolic process	GO:0006644	4.46E-01	4.74E-01	2	5
6,7,8,9	HSPD1|2.88995	positive regulation of T cell activation	GO:0050870	4.46E-01	4.77E-01	1	9

8,9	VTN|2.01125	regulation of peptidyl-tyrosine phosphorylation	GO:0050730	4.46E-01	4.77E-01	1	9
6,7	HSP90AB1|0.458775	cellular response to interferon-gamma	GO:0071346	4.46E-01	4.77E-01	1	7
4	KRT1|0.4198;MAPK14|0.483325	blood vessel	GO:0048514	4.48E-01	4.77E-01	2	4
5,6	VIM|2.241075	epithelial cell	GO:0002064	4.49E-01	4.78E-01	1	6
5,6	KRT1|0.4198	regulation of angiogenesis	GO:0045765	4.49E-01	4.79E-01	1	6
4	ARHGDIA|3.8215	negative regulation of cell adhesion	GO:0007162	4.49E-01	4.79E-01	1	4
6,7	ASPH|2.59965;ATP6V1B2|2.0575;NSF|2.017425;ERO1L|2.370575;TFRC|0.403125;SLC2A1|5.081875	cation transport	GO:0006812	4.51E-01	4.79E-01	6	7
7	STAT3|0.4995	glucose homeostasis	GO:0042593	4.51E-01	4.80E-01	1	7
8	ATP6V1B2|2.0575	insulin receptor signaling pathway	GO:0008286	4.51E-01	4.80E-01	1	8
6	STAT3|0.4995	carbohydrate homeostasis	GO:0033500	4.51E-01	4.80E-01	1	6
6,7	HSPD1|2.88995	positive regulation of homotypic cell-cell adhesion	GO:0034112	4.52E-01	4.80E-01	1	7
4	CNDP2|2.0938	response to xenobiotic stimulus	GO:0009410	4.54E-01	4.81E-01	1	4
6,7	HSPD1|2.88995	positive regulation of leukocyte cell-cell	GO:1903039	4.54E-01	4.83E-01	1	7
5,6	PRDX5|3.0143;VTN|2.01125	negative regulation of hydrolase activity	GO:0051346	4.54E-01	4.83E-01	2	6
5	MTHFD1L|6.667175;MAPK14|0.483325	skeletal system development	GO:0001501	4.54E-01	4.83E-01	2	5
5	BAZ1B|0.460625	heart morphogenesis	GO:0003007	4.55E-01	4.83E-01	1	5
6	SOS2|11.48525	T cell differentiation	GO:0030217	4.57E-01	4.84E-01	1	6
8	PSME3|0.401575;ATG7|2.120975;TTK|0.488375;PLK1|0.415475	protein modification by small protein conjugation	GO:0032446	4.57E-01	4.85E-01	4	8
7,8	SARS|2.2366;SRRT|0.39875	ncRNA processing	GO:0034470	4.57E-01	4.85E-01	2	8
8,9	SARS|2.2366	tRNA processing	GO:0008033	4.60E-01	4.85E-01	1	9
5,6	ASPH|2.59965	regulation of muscle system process	GO:0090257	4.60E-01	4.87E-01	1	6
8,9,10	SOS2|11.48525	regulation of Rho protein signal transduction	GO:0035023	4.63E-01	4.87E-01	1	10
6,7	ASPH|2.59965	regulation of ion transmembrane transporter activity	GO:0032412	4.63E-01	4.90E-01	1	7
7	STAT3|0.4995	regulation of purine nucleotide metabolic process	GO:1900542	4.66E-01	4.90E-01	1	7
8	PTP4A3|0.3433	peptidyl-tyrosine dephosphorylation	GO:0035335	4.66E-01	4.93E-01	1	8
5,6	ASPH|2.59965	regulation of transmembrane transporter activity	GO:0022898	4.69E-01	4.93E-01	1	6
4,5	HSPD1|2.88995;MAPK14|0.483325	regulation of cytokine production	GO:0001817	4.70E-01	4.96E-01	2	5
5,6,7,8,9,10	SLC2A1|5.081875	regulation of peptide hormone secretion	GO:0090276	4.72E-01	4.97E-01	1	10
10	PSME3|0.401575	protein polyubiquitination	GO:0000209	4.73E-01	4.99E-01	1	10
4,6,7	ASPH|2.59965	positive regulation of ion transport	GO:0043270	4.75E-01	5.00E-01	1	7
5,6	VTN|2.01125	regulation of endocytosis	GO:0030100	4.75E-01	5.01E-01	1	6
7	HSP90AB1|0.458775;YWHAG|2.359475;BAZ1B|0.460625;LAMTOR2|7.841025;SQSTM1|6.007525;MAPK14|0.483325;VTN|2.01125;TTK|0.488375;PLK1|0.415475	protein phosphorylation	GO:0006468	4.75E-01	5.01E-01	9	7
3	AKR1B1|2.235325	multi-multicellular organism process	GO:0044706	4.76E-01	5.01E-01	1	3
5	KRT1|0.4198	regulation of vasculature development	GO:1901342	4.76E-01	5.02E-01	1	5
7,8	ATP6V1B2|2.0575	hydrogen ion transmembrane transport	GO:1902600	4.76E-01	5.02E-01	1	8
6,7,8	SQSTM1|6.007525	regulation of I-kappaB kinase/NF-kappaB	GO:0043122	4.76E-01	5.02E-01	1	8
6	STAT3|0.4995	regulation of nucleotide metabolic process	GO:0006140	4.77E-01	5.02E-01	1	6
6,7,8,9	SLC2A1|5.081875	regulation of peptide secretion	GO:0002791	4.79E-01	5.03E-01	1	9
7,8	GSN|2.4555	phosphatidylinositol-mediated signaling	GO:0048015	4.80E-01	5.04E-01	1	8
6,7,8	SLC2A1|5.081875	insulin secretion	GO:0030073	4.80E-01	5.05E-01	1	8
5	VAT1|2.702925	organelle fusion	GO:0048284	4.82E-01	5.05E-01	1	5
5	VTN|2.01125	cell-matrix adhesion	GO:0007160	4.82E-01	5.07E-01	1	5
6,7	GSN|2.4555	inositol lipid-mediated signaling	GO:0048017	4.83E-01	5.07E-01	1	7
5	PSME3|0.401575	cellular amine metabolic process	GO:0044106	4.83E-01	5.08E-01	1	5
6,7,8,9	LAMTOR2|7.841025;MAPK14|0.483325	positive regulation of MAPK cascade	GO:0043410	4.85E-01	5.08E-01	2	9
6	SNW1|0.473175	transforming growth factor beta receptor signaling pathway	GO:0007179	4.86E-01	5.09E-01	1	6
4,5	ASPH|2.59965	regulation of transporter activity	GO:0032409	4.87E-01	5.10E-01	1	5
9,10	ASPH|2.59965;ERO1L|2.370575	calcium ion transport	GO:0006816	4.91E-01	5.11E-01	2	10

7	PLP1|3.936	monocarboxylic acid biosynthetic process	GO:0072330	4.93E-01	5.15E-01	1	7
4,5	NSF|2.017425;VTN|2.01125	regulation of vesicle-mediated transport	GO:0060627	4.94E-01	5.17E-01	2	5
7	HSPD1|2.88995	immunoglobulin mediated immune response	GO:0016064	4.98E-01	5.18E-01	1	7
5	PSME3|0.401575	antigen processing and presentation of exogenous peptide antigen	GO:0002478	4.98E-01	5.22E-01	1	5
4	PLP1|3.936	developmental maturation	GO:0021700	5.01E-01	5.22E-01	1	4
6	HSPD1|2.88995	B cell mediated immunity	GO:0019724	5.03E-01	5.25E-01	1	6
5,6	HSPD1|2.88995	positive regulation of cell-cell adhesion	GO:0022409	5.04E-01	5.26E-01	1	6
5	SOS2|11.48525	regulation of leukocyte differentiation	GO:1902105	5.04E-01	5.27E-01	1	5
5,7	ASPH|2.59965	regulation of blood circulation	GO:1903522	5.05E-01	5.27E-01	1	7
5	PSME3|0.401575	amine metabolic process	GO:0009308	5.05E-01	5.28E-01	1	5
5	HSPD1|2.88995	B cell activation	GO:0042113	5.05E-01	5.28E-01	1	5
4	MTHFD1L|6.667175;VTN|2.01125	embryonic morphogenesis	GO:0048598	5.07E-01	5.28E-01	2	4
6,7,8	PSME3|0.401575	extrinsic apoptotic signaling pathway	GO:0097191	5.07E-01	5.29E-01	1	8
4	PSME3|0.401575	antigen processing and presentation of exogenous antigen	GO:0019884	5.08E-01	5.29E-01	1	4
5	MAPK14|0.483325	connective tissue development	GO:0061448	5.08E-01	5.30E-01	1	5
7,9,10	ASPH|2.59965	regulation of calcium ion transport	GO:0051924	5.09E-01	5.30E-01	1	10
5,6	HSPA8|0.3721;SLC2A1|5.081875	signal release	GO:0023061	5.11E-01	5.31E-01	2	6
4	KRT1|0.4198;MAPK14|0.483325	blood vessel development	GO:0001568	5.12E-01	5.32E-01	2	4
4	MTHFD1L|6.667175;AKR1B1|2.235325	tube development	GO:0035295	5.12E-01	5.33E-01	2	4
7,8,9,10	MAPK14|0.483325	positive regulation of MAP kinase activity	GO:0043406	5.19E-01	5.33E-01	1	10
4	HSPD1|2.88995;MAPK14|0.483325	cytokine production	GO:0001816	5.25E-01	5.40E-01	2	4
5	RPL3|3.30745	ribonucleoprotein complex assembly	GO:0022618	5.25E-01	5.47E-01	1	5
5	ARHGDIA|3.8215	positive regulation of cell projection organization	GO:0031346	5.27E-01	5.47E-01	1	5
5	KRT1|0.4198;MAPK14|0.483325	vasculature development	GO:0001944	5.30E-01	5.48E-01	2	5
7,8,9	SLC2A1|5.081875	peptide hormone secretion	GO:0030072	5.30E-01	5.51E-01	1	9
4	AKR1B1|2.235325	kidney development	GO:0001822	5.32E-01	5.51E-01	1	4
5	STAT3|0.4995	sensory organ morphogenesis	GO:0090596	5.32E-01	5.52E-01	1	5
5	SNW1|0.473175	cellular response to transforming growth factor beta stimulus	GO:0071560	5.37E-01	5.52E-01	1	5
5	RPL3|3.30745	ribonucleoprotein complex subunit	GO:0071826	5.37E-01	5.57E-01	1	5
6,7,9	GSN|2.4555	regulation of actin filament polymerization	GO:0030833	5.38E-01	5.57E-01	1	9
5	SLC2A1|5.081875	regulation of peptide transport	GO:0090087	5.38E-01	5.58E-01	1	5
4	SNW1|0.473175	response to transforming growth factor beta	GO:0071559	5.38E-01	5.58E-01	1	4
6,7,8	SLC2A1|5.081875	peptide secretion	GO:0002790	5.39E-01	5.58E-01	1	8
5	RHOG|2.449775	cell chemotaxis	GO:0060326	5.41E-01	5.59E-01	1	5
8,9	ASPH|2.59965;ERO1L|2.370575	divalent metal ion	GO:0070838	5.41E-01	5.60E-01	2	9
4,5,6,7,8	SLC2A1|5.081875	regulation of hormone secretion	GO:0046883	5.42E-01	5.60E-01	1	8
7,8	ASPH|2.59965;ERO1L|2.370575	divalent inorganic cation transport	GO:0072511	5.43E-01	5.61E-01	2	8
4	PSME3|0.401575	antigen processing and presentation of peptide antigen	GO:0048002	5.46E-01	5.62E-01	1	4
5,6,7,8	HSPD1|2.88995	positive regulation of lymphocyte activation	GO:0051251	5.46E-01	5.64E-01	1	8
3	HSP90AB1|0.458775;MAPK14|0.483325	developmental process involved in reproduction	GO:0003006	5.47E-01	5.64E-01	2	3
7	ATP6V1B2|2.0575	cellular response to insulin stimulus	GO:0032869	5.47E-01	5.65E-01	1	7
6,7	SQSTM1|6.007525	I-kappaB kinase/NF-kappaB signaling	GO:0007249	5.52E-01	5.65E-01	1	7
5	AKR1B1|2.235325	renal system development	GO:0072001	5.52E-01	5.70E-01	1	5
7,8	ATP6V1B2|2.0575;NSF|2.017425;SLC2A1|5.081875	monovalent inorganic cation transport	GO:0015672	5.55E-01	5.70E-01	3	8
7	PLEK|2.071125	phosphatidylinositol metabolic process	GO:0046488	5.58E-01	5.73E-01	1	7
7	PSME3|0.401575;ATG7|2.120975;TTK|0.488375;PLK1|0.415475	protein modification by small protein conjugation or removal	GO:0070647	5.64E-01	5.76E-01	4	7
5	MAPK14|0.483325	lipid modification	GO:0030258	5.68E-01	5.82E-01	1	5
5	MTHFD1L|6.667175	embryonic organ morphogenesis	GO:0048562	5.68E-01	5.85E-01	1	5
5,6,8,9	GSN|2.4555	regulation of protein polymerization	GO:0032271	5.68E-01	5.85E-01	1	9
8	GSN|2.4555	actin filament polymerization	GO:0030041	5.69E-01	5.85E-01	1	8

4,5,7,8	MAPK14|0.483325	positive regulation of secretion by cell	GO:1903532	5.72E-01	5.86E-01	1	8
6,7,8	MAPK14|0.483325	stress-activated MAPK cascade	GO:0051403	5.74E-01	5.89E-01	1	8
5,6	ALB|0.23405	organic hydroxy compound transport	GO:0015850	5.74E-01	5.90E-01	1	6
4	HSPD1|2.88995	positive regulation of leukocyte activation	GO:0002696	5.74E-01	5.90E-01	1	4
5,6,8,9	NSF|2.017425	regulation of exocytosis	GO:0017157	5.78E-01	5.90E-01	1	9
5	ATG7|2.120975;MYH10|5.579275;GCH1|4.01735;SOD2|2.175425	neurological system process	GO:0050877	5.79E-01	5.94E-01	4	5
5,6,7	HSPA8|0.3721	neurotransmitter secretion	GO:0007269	5.79E-01	5.95E-01	1	7
6	ARHGDIA|3.8215	regulation of cell morphogenesis involved in differentiation	GO:0010769	5.81E-01	5.95E-01	1	6
5	ISYNA1|0.49065	phospholipid biosynthetic process	GO:0008654	5.83E-01	5.96E-01	1	5
5	SLC25A1|2.1121	glycerolipid biosynthetic process	GO:0045017	5.85E-01	5.98E-01	1	5
2	ATG7|2.120975;STAT3|0.4995	behavior	GO:0007610	5.90E-01	6.00E-01	2	2
5	ERO1L|2.370575	response to endoplasmic reticulum stress	GO:0034976	5.93E-01	6.05E-01	1	5
5,6	VDAC3|2.015475	cilium assembly	GO:0042384	5.96E-01	6.08E-01	1	6
5	AKR1B1|2.235325	urogenital system development	GO:0001655	5.98E-01	6.11E-01	1	5
5	SOS2|11.48525	lymphocyte differentiation	GO:0030098	6.00E-01	6.12E-01	1	5
6,7	SLC2A1|5.081875	hormone secretion	GO:0046879	6.03E-01	6.14E-01	1	7
6	ATP6V1B2|2.0575	response to insulin	GO:0032868	6.05E-01	6.17E-01	1	6
6,7,8,10	ARHGDIA|3.8215	positive regulation of neuron differentiation	GO:0045666	6.11E-01	6.19E-01	1	10
5,6,7	MTHFD1L|6.667175	epithelial tube morphogenesis	GO:0060562	6.13E-01	6.24E-01	1	7
7,8,9,10,11	MAPK14|0.483325	regulation of MAP kinase activity	GO:0043405	6.17E-01	6.26E-01	1	11
4	VTN|2.01125	cell-substrate adhesion	GO:0031589	6.20E-01	6.31E-01	1	4
5,6	SLC2A1|5.081875	hormone transport	GO:0009914	6.22E-01	6.33E-01	1	6
6,7	SLC2A1|5.081875	peptide transport	GO:0015833	6.25E-01	6.35E-01	1	7
5	ASPH|2.59965;VDAC3|2.015475	regulation of ion transport	GO:0043269	6.26E-01	6.38E-01	2	5
5	PLBD2|2.666725	lipid catabolic process	GO:0016042	6.26E-01	6.39E-01	1	5
8,9	NSF|2.017425	potassium ion transport	GO:0006813	6.28E-01	6.39E-01	1	9
5	ATP6V1B2|2.0575;STAT3|0.4995	cellular response to hormone stimulus	GO:0032870	6.30E-01	6.41E-01	2	5
5	VDAC3|2.015475	cilium organization	GO:0044782	6.31E-01	6.43E-01	1	5
4	HSPA8|0.3721	regulation of neurotransmitter levels	GO:0001505	6.33E-01	6.43E-01	1	4
5	GMPS|0.496875;RRM2|0.21675;STAT3|0.4995	carbohydrate derivative biosynthetic process	GO:1901137	6.34E-01	6.44E-01	3	5
5	HSPD1|2.88995	adaptive immune response based on somatic recombination of immune receptors built from immunoglobulin superfamily domains	GO:0002460	6.35E-01	6.46E-01	1	5
3	HIST1H4A|0.23835	negative regulation of immune system process	GO:0002683	6.40E-01	6.46E-01	1	3
5	HSPD1|2.88995	lymphocyte mediated immunity	GO:0002449	6.41E-01	6.51E-01	1	5
7	ATG7|2.120975	post-translational protein modification	GO:0043687	6.41E-01	6.51E-01	1	7
8	MAPK14|0.483325	protein	GO:0046777	6.42E-01	6.51E-01	1	8
5	NDE1|0.446425	establishment of vesicle localization	GO:0051650	6.42E-01	6.52E-01	1	5
3	KRT9|0.268525;AKR1B1|2.235325	multicellular organismal reproductive process	GO:0048609	6.43E-01	6.53E-01	2	3
3	PSME3|0.401575	antigen processing and presentation	GO:0019882	6.44E-01	6.53E-01	1	3
8,9,10,11	ASPH|2.59965	calcium ion transmembrane transport	GO:0070588	6.44E-01	6.54E-01	1	11
6,7	SQSTM1|6.007525	endosomal transport	GO:0016197	6.46E-01	6.54E-01	1	7
5	PCNA|0.404975	response to light stimulus	GO:0009416	6.52E-01	6.56E-01	1	5
5	NDE1|0.446425	vesicle localization	GO:0051648	6.53E-01	6.61E-01	1	5
4	MTHFD1L|6.667175	tube morphogenesis	GO:0035239	6.53E-01	6.62E-01	1	4
6	PLEK|2.071125;PTP4A3|0.3433	dephosphorylation	GO:0016311	6.56E-01	6.62E-01	2	6
3	KRT9|0.268525;AKR1B1|2.235325	multicellular organism reproduction	GO:0032504	6.59E-01	6.65E-01	2	3
6,8,9	ASPH|2.59965	regulation of metal ion transport	GO:0010959	6.63E-01	6.67E-01	1	9
4,5,6	ASPH|2.59965;ATP6V1B2|2.0575;VDAC3|2.015475;SLC25A1|2.1121;TFRC|0.403125;SLC2A1|5.081875;ALB|0.234	transmembrane transport	GO:0055085	6.63E-01	6.71E-01	7	6
5,6	HSPA8|0.3721	neurotransmitter transport	GO:0006836	6.65E-01	6.71E-01	1	6
4,7,8	MAPK14|0.483325	regulation of sequence-specific DNA binding transcription factor	GO:0051090	6.65E-01	6.73E-01	1	8
6,7,8,9	LAMTOR2|7.841025;MAPK14|0.483325	regulation of MAPK cascade	GO:0043408	6.73E-01	6.73E-01	2	9
5	RPL3|3.30745	ribosome biogenesis	GO:0042254	6.79E-01	6.80E-01	1	5
3	ATG7|2.120975	single-organism behavior	GO:0044708	6.82E-01	6.86E-01	1	3
5,6	SLC2A1|5.081875	amide transport	GO:0042886	6.83E-01	6.89E-01	1	6

6	PLEK|2.071125	glycerophospholipid metabolic process	GO:0006650	6.86E-01	6.90E-01	1	6
4	ATG7|2.120975	gland development	GO:0048732	7.00E-01	6.92E-01	1	4
4	HSPD1|2.88995	adaptive immune response	GO:0002250	7.04E-01	7.06E-01	1	4
4	HSPD1|2.88995	leukocyte mediated immunity	GO:0002443	7.09E-01	7.10E-01	1	4
5	NDE1|0.446425	microtubule-based movement	GO:0007018	7.09E-01	7.14E-01	1	5
4	MTHFD1L|6.667175	embryonic organ development	GO:0048568	7.13E-01	7.14E-01	1	4
4,5	SLC2A1|5.081875	energy derivation by oxidation of organic compounds	GO:0015980	7.17E-01	7.19E-01	1	5
4	ASPH|2.59965	pattern specification process	GO:0007389	7.26E-01	7.22E-01	1	4
6	G3BP1|0.3904	nucleic acid phosphodiester bond	GO:0090305	7.28E-01	7.31E-01	1	6
4	ASPH|2.59965	regulation of system process	GO:0044057	7.32E-01	7.32E-01	1	4
5,6,7	LAMTOR2|7.841025;MAPK14|0.483325	MAPK cascade	GO:0000165	7.40E-01	7.37E-01	2	7
5,6	ALB|0.23405	lipid transport	GO:0006869	7.40E-01	7.44E-01	1	6
6	KRT9|0.268525	spermatogenesis	GO:0007283	7.57E-01	7.44E-01	1	6
5	KRT9|0.268525	male gamete generation	GO:0048232	7.58E-01	7.61E-01	1	5
6,7,8	VDAC3|2.015475	anion transmembrane transport	GO:0098656	7.68E-01	7.61E-01	1	8
4	ALB|0.23405	lipid localization	GO:0010876	7.68E-01	7.71E-01	1	4
4,8	LAMTOR2|7.841025;MAPK14|0.483325	signal transduction by protein phosphorylation	GO:0023014	7.78E-01	7.71E-01	2	8
5	VDAC3|2.015475;SLC2A1|5.081875	nitrogen compound transport	GO:0071705	7.93E-01	7.81E-01	2	5
4	RPL3|3.30745	ribonucleoprotein complex biogenesis	GO:0022613	8.13E-01	7.95E-01	1	4
7,8,9	ASPH|2.59965;ATP6V1B2|2.0575	inorganic cation transmembrane transport	GO:0098662	8.16E-01	8.15E-01	2	9
4	KRT9|0.268525	gamete generation	GO:0007276	8.31E-01	8.18E-01	1	4
6,7,8	ASPH|2.59965;ATP6V1B2|2.0575	cation transmembrane transport	GO:0098655	8.72E-01	8.33E-01	2	8
6,7,8	ASPH|2.59965;ATP6V1B2|2.0575	inorganic ion transmembrane transport	GO:0098660	8.74E-01	8.73E-01	2	8
5,6,7	ASPH|2.59965;ATP6V1B2|2.0575;VDAC3|2.015475	ion transmembrane transport	GO:0034220	9.20E-01	8.75E-01	3	7
5,6	PLEK|2.071125	G-protein coupled receptor signaling	GO:0007186	9.93E-01	9.20E-01	1	6
1	HSPA8|0.3721;ACTG1|2.1999;HSP90AB1|0.458775;LCP1|2.170475;ENO1|2.1939;HSPD1|2.88995;HIST1H2BN|0.481625;RPL3|3.30745;VIM|2.241075;CCT7|2.06135;PABPC1|0.3285;SARS|2.2366;ASPH|2.59965;KRT1|0.4198;YARS|2.288275;ATP6V1B2|2.0575;MCM3|0.35205;KRT9|0.268525;RPL4|2.063325;NSF|2.017425;GMPS|0.496875;MTHFD1L|6.667175;CKB|0.486625;NARS|2.162;MCM2|0.491625;HSPA4L|2.13105;SMC2|0.485025;DNMT1|0.172;ALDH1L2|2.148;HIST1H4A|0.23835;KPNA2|0.186675;VDAC3|2.015475;YWHAG|2.359475;ANXA5|3.637725;SRRT|0.39875;GSN|2.4555;CNDP2|2.0938;NCAPG|0.4528;CBX3|0.485775;GTF2E1|0.300475;AKR1B1|2.235325;PABPC4|0.26245;LIG1|0.440425;SLC25A1|2.1121;G3BP1|0.3904;ERO1L|2.370575;PRDX5|3.0143;BAZ1B|0.460625;ESD|2.155525;ISYNA1|0.49065;ALDOC|2.04725;PSME3|0.401575;RRM2|0.21675;EIF4H|0.275675;ACAT2|2.108325;LAMTOR2|7.841025;MCMBP|0.4769;RPL27|2.15885;SQSTM1|6.007525;ATG7|2.120975;PCNA|0.404975;MAPK14|0.483325;HIST1H3A|0.455625;BTF3|0.2449;TFRC|0.403125;RHOG|2.449775;TOP2A|0.481675;SNW1|0.473175;STAT3|0.4995;TUBG1|0.4102;NCDN|0.4114;SLC2A1|5.081875;TXNIP|2.0553;VAT1|2.702925;IPO8|0.443925;PLEK|2.071125;NDE1|0.446425;S100A11|2.531875;HBA1|2.13735;NDRG1|10.0073;IK|0.480125;VTN|2.01125;RBBP7|0.218475;ARHGDIA|3.8215;PLBD2|2.666725;ACTB|2.1999;DNPEP|2.077575;H3F3A|0.20985;MYH10|5.579275;TTK|0.488375;SOS2|11.48525;ASL|2.252975;ALB|0.23405;CDC123|0.29925;PLK1|0.415475;GCH1|4.01735;SOD2|2.175425;PTP4A3|0.3433;HEATR9|2.53905;TBCA|0.3515;CETN3|0.4171;IFITM2|5.68315;ZNF706|0.3974;CHAF1B|0.2723;PLP1|3.936	biological_process	GO:0008150	1.00E+00	9.93E-01	105	1

Table S6 CC enrichment analysis of differentially expressed proteins

<table>
<tr><th>levels</th><th>Genes</th><th>GO_Name</th><th>GO_ID</th><th>Pvalue</th><th>Pvalue_adjusted</th><th>Count</th><th>maxLevel</th></tr>
<tr><td>4</td><td>HSPA8|0.3721;ACTG1|2.1999;HSP90AB1|0.458775;LCP1|2.170475;ENO1|2.1939;HSPD1|2.88995;HIST1H2BN|0.481625;RPL3|3.30745;VIM|2.241075;CCT7|2.06135;PABPC1|0.3285;SARS|2.2366;KRT1|0.4198;ATP6V1B2|2.0575;KRT9|0.268525;RPL4|2.063325;NSF|2.017425;CKB|0.486625;NARS|2.162;SMC2|0.485025;ALDH1L2|2.148;HIST1H4A|0.23835;VDAC3|2.015475;YWHAG|2.359475;ANXA5|3.637725;GSN|2.4555;CNDP2|2.0938;AKR1B1|2.235325;SLC25A1|2.1121;PRDX5|3.0143;ESD|2.155525;ALDOC|2.04725;ACAT2|2.108325;LAMTOR2|7.841025;RPL27|2.15885;SQSTM1|6.007525;PCNA|0.404975;MAPK14|0.483325;HIST1H3A|0.455625;TFRC|0.403125;RHOG|2.449775;SLC2A1|5.081875;VAT1|2.702925;S100A11|2.531875;HBA1|2.13735;NDRG1|10.0073;VTN|2.01125;ARHGDIA|3.8215;PLBD2|2.666725;ACTB|2.1999;HIST2H2AA3|0.29265;H3F3A|0.20985;MYH10|5.579275;ASL|2.252975;ALB|0.23405;SOD2|2.175425;TB</td><td>extracellular membrane-bounded organelle</td><td>GO:0065010</td><td>1.43E-33</td><td>1.84E-31</td><td>57</td><td>4</td></tr>
<tr><td>4</td><td>HSPA8|0.3721;ACTG1|2.1999;HSP90AB1|0.458775;LCP1|2.170475;ENO1|2.1939;HSPD1|2.88995;HIST1H2BN|0.481625;RPL3|3.30745;VIM|2.241075;CCT7|2.06135;PABPC1|0.3285;SARS|2.2366;KRT1|0.4198;ATP6V1B2|2.0575;KRT9|0.268525;RPL4|2.063325;NSF|2.017425;CKB|0.486625;NARS|2.162;SMC2|0.485025;ALDH1L2|2.148;HIST1H4A|0.23835;VDAC3|2.015475;YWHAG|2.359475;ANXA5|3.637725;GSN|2.4555;CNDP2|2.0938;AKR1B1|2.235325;SLC25A1|2.1121;PRDX5|3.0143;ESD|2.155525;ALDOC|2.04725;ACAT2|2.108325;LAMTOR2|7.841025;RPL27|2.15885;SQSTM1|6.007525;PCNA|0.404975;MAPK14|0.483325;HIST1H3A|0.455625;TFRC|0.403125;RHOG|2.449775;SLC2A1|5.081875;VAT1|2.702925;S100A11|2.531875;HBA1|2.13735;NDRG1|10.0073;VTN|2.01125;ARHGDIA|3.8215;PLBD2|2.666725;ACTB|2.1999;HIST2H2AA3|0.29265;H3F3A|0.20985;MYH10|5.579275;ASL|2.252975;ALB|0.23405;SOD2|2.175425;TB</td><td>extracellular exosome</td><td>GO:0070062</td><td>1.43E-33</td><td>1.84E-31</td><td>57</td><td>4</td></tr>
<tr><td>3</td><td>HSPA8|0.3721;ACTG1|2.1999;HSP90AB1|0.458775;LCP1|2.170475;ENO1|2.1939;HSPD1|2.88995;HIST1H2BN|0.481625;RPL3|3.30745;VIM|2.241075;CCT7|2.06135;PABPC1|0.3285;SARS|2.2366;KRT1|0.4198;ATP6V1B2|2.0575;KRT9|0.268525;RPL4|2.063325;NSF|2.017425;CKB|0.486625;NARS|2.162;SMC2|0.485025;ALDH1L2|2.148;HIST1H4A|0.23835;VDAC3|2.015475;YWHAG|2.359475;ANXA5|3.637725;GSN|2.4555;CNDP2|2.0938;AKR1B1|2.235325;SLC25A1|2.1121;PRDX5|3.0143;ESD|2.155525;ALDOC|2.04725;ACAT2|2.108325;LAMTOR2|7.841025;RPL27|2.15885;SQSTM1|6.007525;PCNA|0.404975;MAPK14|0.483325;HIST1H3A|0.455625;TFRC|0.403125;RHOG|2.449775;SLC2A1|5.081875;VAT1|2.702925;S100A11|2.531875;HBA1|2.13735;NDRG1|10.0073;VTN|2.01125;ARHGDIA|3.8215;PLBD2|2.666725;ACTB|2.1999;HIST2H2AA3|0.29265;H3F3A|0.20985;MYH10|5.579275;ASL|2.252975;ALB|0.23405;SOD2|2.175425;TB</td><td>extracellular organelle</td><td>GO:0043230</td><td>1.83E-33</td><td>1.84E-31</td><td>57</td><td>3</td></tr>
<tr><td>3</td><td>HSPA8|0.3721;ACTG1|2.1999;HSP90AB1|0.458775;LCP1|2.170475;ENO1|2.1939;HSPD1|2.88995;HIST1H2BN|0.481625;RPL3|3.30745;VIM|2.241075;CCT7|2.06135;PABPC1|0.3285;SARS|2.2366;KRT1|0.4198;ATP6V1B2|2.0575;KRT9|0.268525;RPL4|2.063325;NSF|2.017425;CKB|0.486625;NARS|2.162;SMC2|0.485025;ALDH1L2|2.148;HIST1H4A|0.23835;VDAC3|2.015475;YWHAG|2.359475;ANXA5|3.637725;GSN|2.4555;CNDP2|2.0938;AKR1B1|2.235325;SLC25A1|2.1121;PRDX5|3.0143;ESD|2.155525;ALDOC|2.04725;ACAT2|2.108325;LAMTOR2|7.841025;RPL27|2.15885;SQSTM1|6.007525;PCNA|0.404975;MAPK14|0.483325;HIST1H3A|0.455625;TFRC|0.403125;RHOG|2.449775;SLC2A1|5.081875;VAT1|2.702925;S100A11|2.531875;HBA1|2.13735;NDRG1|10.0073;VTN|2.01125;ARHGDIA|3.8215;PLBD2|2.666725;ACTB|2.1999;HIST2H2AA3|0.29265;H3F3A|0.20985;MYH10|5.579275;ASL|2.252975;ALB|0.23405;SOD2|2.175425;TB</td><td>extracellular vesicle</td><td>GO:1903561</td><td>1.83E-33</td><td>1.84E-31</td><td>57</td><td>3</td></tr>
</table>

5,6	HSPA8|0.3721;ACTG1|2.1999;HSP90AB1|0.458775;LCP1|2.170475;ENO1|2.1939;HSPD1|2.88995;RPL3|3.30745;VIM|2.241075;CCT7|2.06135;PABPC1|0.3285;SARS|2.2366;YARS|2.288275;ATP6V1B2|2.0575;RPL4|2.063325;NSF|2.017425;GMPS|0.496875;MTHFD1L|6.667175;CKB|0.486625;NARS|2.162;HSPA4L|2.13105;SMC2|0.485025;KPNA2|0.186675;YWHAG|2.359475;GSN|2.4555;CNDP2|2.0938;NCAPG|0.4528;AKR1B1|2.235325;G3BP1|0.3904;PRDX5|3.0143;ISYNA1|0.49065;ALDOC|2.04725;PSME3|0.401575;RRM2|0.21675;EIF4H|0.275675;RPL27|2.15885;SQSTM1|6.007525;ATG7|2.120975;MAPK14|0.483325;RHOG|2.449775;STAT3|0.4995;TUBG1|0.4102;NCDN|0.4114;SLC2A1|5.081875;TXNIP|2.0553;IPO8|0.443925;PLEK|2.071125;NDE1|0.446425;HBA1|2.13735;NDRG1|10.0073;ARHGDIA|3.8215;ACTB|2.1999;MYH10|5.579275;SOS2|11.48525;ASL|2.252975;PLK1|0.415475;GCH1|4.01735;CHAF1B|0.2723	cytosol	GO:0005829	2.44E-27	1.96E-25	57	6
2	HSPA8|0.3721;ACTG1|2.1999;HSP90AB1|0.458775;LCP1|2.170475;ENO1|2.1939;HSPD1|2.88995;HIST1H2BN|0.481625;RPL3|3.30745;VIM|2.241075;CCT7|2.06135;PABPC1|0.3285;SARS|2.2366;KRT1|0.4198;YARS|2.288275;ATP6V1B2|2.0575;KRT9|0.268525;RPL4|2.063325;NSF|2.017425;CKB|0.486625;NARS|2.162;SMC2|0.485025;ALDH1L2|2.148;HIST1H4A|0.23835;VDAC3|2.015475;YWHAG|2.359475;ANXA5|3.637725;GSN|2.4555;CNDP2|2.0938;AKR1B1|2.235325;SLC25A1|2.1121;PRDX5|3.0143;ESD|2.155525;ALDOC|2.04725;ACAT2|2.108325;LAMTOR2|7.841025;RPL27|2.15885;SQSTM1|6.007525;PCNA|0.404975;MAPK14|0.483325;HIST1H3A|0.455625;TFRC|0.403125;RHOG|2.449775;SLC2A1|5.081875;VAT1|2.702925;S100A11|2.531875;HBA1|2.13735;NDRG1|10.0073;IK|0.480125;VTN|2.01125;ARHGDIA|3.8215;PLBD2|2.666725;ACTB|2.1999;DNPEP|2.077575;HIST2H2AA3|0.29265;H3F3A|0.20985;MYH10|5.579275;ASL|2.252975;ALB|0.23405;SOD2|2.175425;TBCA|0.3515	extracellular region part	GO:0044421	4.96E-27	3.32E-25	60	2
3	HSPA8|0.3721;ACTG1|2.1999;HSP90AB1|0.458775;LCP1|2.170475;ENO1|2.1939;HSPD1|2.88995;HIST1H2BN|0.481625;RPL3|3.30745;VIM|2.241075;CCT7|2.06135;PABPC1|0.3285;SARS|2.2366;KRT1|0.4198;ATP6V1B2|2.0575;KRT9|0.268525;RPL4|2.063325;NSF|2.017425;CKB|0.486625;NARS|2.162;SMC2|0.485025;ALDH1L2|2.148;HIST1H4A|0.23835;VDAC3|2.015475;YWHAG|2.359475;ANXA5|3.637725;GSN|2.4555;CNDP2|2.0938;AKR1B1|2.235325;SLC25A1|2.1121;PRDX5|3.0143;ESD|2.155525;ALDOC|2.04725;ACAT2|2.108325;LAMTOR2|7.841025;RPL27|2.15885;SQSTM1|6.007525;PCNA|0.404975;MAPK14|0.483325;HIST1H3A|0.455625;TFRC|0.403125;RHOG|2.449775;SLC2A1|5.081875;VAT1|2.702925;S100A11|2.531875;HBA1|2.13735;NDRG1|10.0073;VTN|2.01125;ARHGDIA|3.8215;PLBD2|2.666725;ACTB|2.1999;HIST2H2AA3|0.29265;H3F3A|0.20985;MYH10|5.579275;ASL|2.252975;ALB|0.23405;GCH1|4.01735;SOD2|2.175425;TBCA|0.3515	vesicle	GO:0031982	2.64E-26	1.52E-24	58	3
4	HSPA8|0.3721;ACTG1|2.1999;HSP90AB1|0.458775;LCP1|2.170475;ENO1|2.1939;HSPD1|2.88995;HIST1H2BN|0.481625;RPL3|3.30745;VIM|2.241075;CCT7|2.06135;PABPC1|0.3285;SARS|2.2366;KRT1|0.4198;ATP6V1B2|2.0575;KRT9|0.268525;RPL4|2.063325;NSF|2.017425;CKB|0.486625;NARS|2.162;SMC2|0.485025;ALDH1L2|2.148;HIST1H4A|0.23835;VDAC3|2.015475;YWHAG|2.359475;ANXA5|3.637725;GSN|2.4555;CNDP2|2.0938;AKR1B1|2.235325;SLC25A1|2.1121;PRDX5|3.0143;ESD|2.155525;ALDOC|2.04725;ACAT2|2.108325;LAMTOR2|7.841025;RPL27|2.15885;SQSTM1|6.007525;PCNA|0.404975;MAPK14|0.483325;HIST1H3A|0.455625;TFRC|0.403125;RHOG|2.449775;SLC2A1|5.081875;VAT1|2.702925;S100A11|2.531875;HBA1|2.13735;NDRG1|10.0073;VTN|2.01125;ARHGDIA|3.8215;PLBD2|2.666725;ACTB|2.1999;HIST2H2AA3|0.29265;H3F3A|0.20985;MYH10|5.579275;ASL|2.252975;ALB|0.23405;SOD2|2.175425;TBCA|0.3515	membrane-bounded vesicle	GO:0031988	4.05E-26	2.03E-24	57	4

<table>
<tr><th></th><th></th><th></th><th></th><th></th><th></th><th></th><th></th></tr>
<tr><td>2</td><td>HSPA8|0.3721;ACTG1|2.1999;HSP90AB1|0.458775;LCP1|2.170475;ENO1|2.1939;HSPD1|2.88995;HIST1H2BN|0.481625;RPL3|3.30745;VIM|2.241075;CCT7|2.06135;PABPC1|0.3285;SARS|2.2366;KRT1|0.4198;YARS|2.288275;ATP6V1B2|2.0575;KRT9|0.268525;RPL4|2.063325;NSF|2.017425;CKB|0.486625;NARS|2.162;SMC2|0.485025;ALDH1L2|2.148;HIST1H4A|0.23835;VDAC3|2.015475;YWHAG|2.359475;ANXA5|3.637725;GSN|2.4555;CNDP2|2.0938;AKR1B1|2.235325;SLC25A1|2.1121;PRDX5|3.0143;ESD|2.155525;ALDOC|2.04725;ACAT2|2.108325;LAMTOR2|7.841025;RPL27|2.15885;SQSTM1|6.007525;PCNA|0.404975;MAPK14|0.483325;HIST1H3A|0.455625;TFRC|0.403125;RHOG|2.449775;SLC2A1|5.081875;VAT1|2.702925;PLEK|2.071125;S100A11|2.531875;HBA1|2.13735;NDRG1|10.0073;IK|0.480125;VTN|2.01125;ARHGDIA|3.8215;PLBD2|2.666725;ACTB|2.1999;DNPEP|2.077575;HIST2H2AA3|0.29265;H3F3A|0.20985;MYH10|5.579275;ASL|2.252975;ALB|0.23405;SOD2|2.175425;TBCA|0.3515</td><td>extracellular region</td><td>GO:0005576</td><td>2.23E-22</td><td>9.94E-21</td><td>61</td><td>2</td></tr>
<tr><td>2</td><td>HSPA8|0.3721;ACTG1|2.1999;HSP90AB1|0.458775;LCP1|2.170475;ENO1|2.1939;HSPD1|2.88995;HIST1H2BN|0.481625;RPL3|3.30745;VIM|2.241075;CCT7|2.06135;PABPC1|0.3285;ASPH|2.59965;KRT1|0.4198;ATP6V1B2|2.0575;MCM3|0.35205;KRT9|0.268525;RPL4|2.063325;NSF|2.017425;MCM2|0.491625;SMC2|0.485025;DNMT1|0.172;HIST1H4A|0.23835;KPNA2|0.186675;VDAC3|2.015475;YWHAG|2.359475;ANXA5|3.637725;SRRT|0.39875;GSN|2.4555;NCAPG|0.4528;CBX3|0.485775;GTF2E1|0.300475;AKR1B1|2.235325;LIG1|0.440425;SLC25A1|2.1121;ERO1L|2.370575;ANKRD28|3.3035;PRDX5|3.0143;BAZ1B|0.460625;ESD|2.155525;PSME3|0.401575;RRM2|0.21675;ACAT2|2.108325;LAMTOR2|7.841025;MCMBP|0.4769;RPL27|2.15885;SQSTM1|6.007525;ATG7|2.120975;PCNA|0.404975;MAPK14|0.483325;HIST1H3A|0.455625;TFRC|0.403125;RHOG|2.449775;TOP2A|0.481675;SNW1|0.473175;STAT3|0.4995;TUBG1|0.4102;SLC2A1|5.081875;TXNIP|2.0553;VAT1|2.702925;IPO8|0.443925;NDE1|0.446425;S100A11|2.531875;HBA1|2.13735;NDRG1|10.0073;IK|0.480125;RBBP7|0.218475;PLBD2|2.666725;ACTB|2.1999;DNPEP|2.077575;HIST2H2AA3|0.29265;H3F3A|0.20985;MYH10|5.579275;TTK|0.488375;ALB|0.23405;PLK1|0.415475;GCH1|4.01735;SOD2|2.175425;TBCA|0.3515;CETN3|0.4171;CHAF1B|0.2723</td><td>organelle part</td><td>GO:0044422</td><td>4.59E-15</td><td>1.85E-13</td><td>80</td><td>2</td></tr>
<tr><td>4,5</td><td>HSPA8|0.3721;ACTG1|2.1999;HSP90AB1|0.458775;LCP1|2.170475;ENO1|2.1939;HSPD1|2.88995;RPL3|3.30745;VIM|2.241075;CCT7|2.06135;PABPC1|0.3285;SARS|2.2366;ASPH|2.59965;YARS|2.288275;ATP6V1B2|2.0575;MCM3|0.35205;RPL4|2.063325;NSF|2.017425;GMPS|0.496875;MTHFD1L|6.667175;CKB|0.486625;NARS|2.162;HSPA4L|2.13105;SMC2|0.485025;ALDH1L2|2.148;KPNA2|0.186675;VDAC3|2.015475;YWHAG|2.359475;ANXA5|3.637725;GSN|2.4555;CNDP2|2.0938;NCAPG|0.4528;AKR1B1|2.235325;PABPC4|0.26245;LIG1|0.440425;SLC25A1|2.1121;G3BP1|0.3904;ERO1L|2.370575;PRDX5|3.0143;ESD|2.155525;ISYNA1|0.49065;ALDOC|2.04725;PSME3|0.401575;RRM2|0.21675;EIF4H|0.275675;LAMTOR2|7.841025;RPL27|2.15885;SQSTM1|6.007525;ATG7|2.120975;MAPK14|0.483325;TFRC|0.403125;RHOG|2.449775;TOP2A|0.481675;STAT3|0.4995;TUBG1|0.4102;NCDN|0.4114;SLC2A1|5.081875;TXNIP|2.0553;VAT1|2.702925;IPO8|0.443925;PLEK|2.071125;NDE1|0.446425;HBA1|2.13735;NDRG1|10.0073;ARHGDIA|3.8215;PLBD2|2.666725;ACTB|2.1999;MYH10|5.579275;SOS2|11.48525;ASL|2.252975;ALB|0.23405;PLK1|0.415475;GCH1|4.01735;SOD2|2.175425;PTP4A3|0.3433;CETN3|0.4171;CHAF1B|0.2723</td><td>cytoplasmic part</td><td>GO:0044444</td><td>5.33E-15</td><td>1.95E-13</td><td>76</td><td>5</td></tr>
<tr><td>3</td><td>HSPA8|0.3721;ACTG1|2.1999;HSPD1|2.88995;ATP6V1B2|2.0575;NSF|2.017425;CKB|0.486625;YWHAG|2.359475;GSN|2.4555;AKR1B1|2.235325;NDRG1|10.0073;ACTB|2.1999;ALB|0.23405;PLP1|3.936</td><td>myelin sheath</td><td>GO:0043209</td><td>9.09E-15</td><td>3.05E-13</td><td>13</td><td>3</td></tr>
</table>

3	HSPA8|0.3721;ACTG1|2.1999;HSP90AB1|0.458775;LCP1|2.170475;ENO1|2.1939;HSPD1|2.88995;HIST1H2BN|0.481625;RPL3|3.30745;VIM|2.241075;CCT7|2.06135;PABPC1|0.3285;SARS|2.2366;ASPH|2.59965;KRT1|0.4198;YARS|2.288275;ATP6V1B2|2.0575;MCM3|0.35205;KRT9|0.268525;RPL4|2.063325;NSF|2.017425;MTHFD1L|6.667175;CKB|0.486625;NARS|2.162;MCM2|0.491625;HSPA4L|2.13105;SMC2|0.485025;DNMT1|0.172;ALDH1L2|2.148;HIST1H4A|0.23835;KPNA2|0.186675;VDAC3|2.015475;YWHAG|2.359475;ANXA5|3.637725;SRRT|0.39875;GSN|2.4555;CNDP2|2.0938;NCAPG|0.4528;CBX3|0.485775;GTF2E1|0.300475;AKR1B1|2.235325;PABPC4|0.26245;LIG1|0.440425;SLC25A1|2.1121;G3BP1|0.3904;ERO1L|2.370575;ANKRD28|3.3035;PRDX5|3.0143;BAZ1B|0.460625;ESD|2.155525;ALDOC|2.04725;PSME3|0.401575;RRM2|0.21675;ACAT2|2.108325;LAMTOR2|7.841025;MCMBP|0.4769;RPL27|2.15885;SQSTM1|6.007525;PCNA|0.404975;MAPK14|0.483325;HIST1H3A|0.455625;BTF3|0.2449;TFRC|0.403125;RHOG|2.449775;TOP2A|0.481675;SNW1|0.473175;STAT3|0.4995;TUBG1|0.4102;NCDN|0.4114;SLC2A1|5.081875;TXNIP|2.0553;VAT1|2.702925;IPO8|0.443925;S100A11|2.531875;FAM192A|0.462425;HBA1|2.13735;NDRG1|10.0073;IK|0.480125;VTN|2.01125;RBBP7|0.218475;ARHGDIA|3.8215;PLBD2|2.666725;ACTB|2.1999;DNPEP|2.077575;HIST2H2AA3|0.29265;H3F3A|0.20985;MYH10|5.579275;ASL|2.252975;ALB|0.23405;PLK1|0.415475;GCH1|4.01735;SOD2|2.175425;PTP4A3|0.3433;TBCA|0.3515;CETN3|0.4171;	membrane-bounded organelle	GO:0043227	2.47E-14	7.63E-13	96	3
4,5	HSPA8|0.3721;ACTG1|2.1999;HSP90AB1|0.458775;ENO1|2.1939;HSPD1|2.88995;HIST1H2BN|0.481625;RPL3|3.30745;VIM|2.241075;CCT7|2.06135;PABPC1|0.3285;SARS|2.2366;ASPH|2.59965;KRT1|0.4198;YARS|2.288275;ATP6V1B2|2.0575;MCM3|0.35205;KRT9|0.268525;RPL4|2.063325;NSF|2.017425;MTHFD1L|6.667175;CKB|0.486625;NARS|2.162;MCM2|0.491625;HSPA4L|2.13105;SMC2|0.485025;DNMT1|0.172;ALDH1L2|2.148;HIST1H4A|0.23835;KPNA2|0.186675;VDAC3|2.015475;YWHAG|2.359475;ANXA5|3.637725;SRRT|0.39875;GSN|2.4555;NCAPG|0.4528;CBX3|0.485775;GTF2E1|0.300475;AKR1B1|2.235325;PABPC4|0.26245;LIG1|0.440425;SLC25A1|2.1121;G3BP1|0.3904;ERO1L|2.370575;ANKRD28|3.3035;PRDX5|3.0143;BAZ1B|0.460625;ESD|2.155525;ALDOC|2.04725;PSME3|0.401575;RRM2|0.21675;ACAT2|2.108325;LAMTOR2|7.841025;MCMBP|0.4769;RPL27|2.15885;SQSTM1|6.007525;PCNA|0.404975;MAPK14|0.483325;HIST1H3A|0.455625;BTF3|0.2449;TFRC|0.403125;RHOG|2.449775;TOP2A|0.481675;SNW1|0.473175;STAT3|0.4995;TUBG1|0.4102;NCDN|0.4114;SLC2A1|5.081875;TXNIP|2.0553;VAT1|2.702925;IPO8|0.443925;S100A11|2.531875;FAM192A|0.462425;HBA1|2.13735;NDRG1|10.0073;IK|0.480125;RBBP7|0.218475;ARHGDIA|3.8215;PLBD2|2.666725;ACTB|2.1999;DNPEP|2.077575;HIST2H2AA3|0.29265;H3F3A|0.20985;MYH10|5.579275;ALB|0.23405;PLK1|0.415475;GCH1|4.01735;SOD2|2.175425;PTP4A3|0.3433;TBCA|0.3515;CETN3|0.4171;ZNF706|0.3974;CHAF1B|0.2723	intracellular membrane-bounded organelle	GO:0043231	9.27E-14	2.54E-12	92	5

3,4	HSPA8|0.3721;ACTG1|2.1999;HSP90AB1|0.458775;LCP1|2.170475;HSPD1|2.88995;HIST1H2BN|0.481625;RPL3|3.30745;VIM|2.241075;CCT7|2.06135;PABPC1|0.3285;ASPH|2.59965;KRT1|0.4198;ATP6V1B2|2.0575;MCM3|0.35205;KRT9|0.268525;RPL4|2.063325;NSF|2.017425;MCM2|0.491625;SMC2|0.485025;DNMT1|0.172;HIST1H4A|0.23835;KPNA2|0.186675;VDAC3|2.015475;YWHAG|2.359475;SRRT|0.39875;GSN|2.4555;NCAPG|0.4528;CBX3|0.485775;GTF2E1|0.300475;AKR1B1|2.235325;LIG1|0.440425;SLC25A1|2.1121;ERO1L|2.370575;ANKRD28|3.3035;PRDX5|3.0143;BAZ1B|0.460625;ESD|2.155525;PSME3|0.401575;RRM2|0.21675;ACAT2|2.108325;LAMTOR2|7.841025;MCMBP|0.4769;RPL27|2.15885;SQSTM1|6.007525;PCNA|0.404975;MAPK14|0.483325;HIST1H3A|0.455625;TFRC|0.403125;RHOG|2.449775;TOP2A|0.481675;SNW1|0.473175;STAT3|0.4995;TUBG1|0.4102;SLC2A1|5.081875;TXNIP|2.0553;VAT1|2.702925;IPO8|0.443925;NDE1|0.446425;S100A11|2.531875;HBA1|2.13735;NDRG1|10.0073;IK|0.480125;RBBP7|0.218475;PLBD2|2.666725;ACTB|2.1999;DNPEP|2.077575;HIST2H2AA3|0.29265;H3F3A|0.20985;MYH10|5.579275;TTK|0.488375;ALB|0.23405;PLK1|0.415475;GCH1|4.01735;SOD2|2.175425;TBCA|0.3515;CETN3|0.4171;CH	intracellular organelle part	GO:0044446	9.49E-14	2.54E-12	77	4
3,4	HSPA8|0.3721;ACTG1|2.1999;KRT1|0.4198;ANXA5|3.637725;GSN|2.4555;TFRC|0.403125;SLC2A1|5.081875;HBA1|2.13735;VTN|2.01125;ACTB|2.1999;DNPEP|2.077575;ALB|0.23405	blood microparticle	GO:0072562	1.73E-13	4.35E-12	12	4
2	HSPA8|0.3721;HSP90AB1|0.458775;HSPD1|2.88995;HIST1H2BN|0.481625;RPL3|3.30745;ASPH|2.59965;MCM3|0.35205;RPL4|2.063325;MCM2|0.491625;SMC2|0.485025;DNMT1|0.172;HIST1H4A|0.23835;KPNA2|0.186675;SRRT|0.39875;CBX3|0.485775;GTF2E1|0.300475;AKR1B1|2.235325;LIG1|0.440425;ERO1L|2.370575;ANKRD28|3.3035;PRDX5|3.0143;BAZ1B|0.460625;ESD|2.155525;PSME3|0.401575;RRM2|0.21675;ACAT2|2.108325;MCMBP|0.4769;SQSTM1|6.007525;PCNA|0.404975;MAPK14|0.483325;HIST1H3A|0.455625;TOP2A|0.481675;SNW1|0.473175;STAT3|0.4995;TUBG1|0.4102;TXNIP|2.0553;IPO8|0.443925;S100A11|2.531875;HBA1|2.13735;IK|0.480125;RBBP7|0.218475;PLBD2|2.666725;ACTB|2.1999;DNPEP|2.077575;H3F3A|0.20985;ALB|0.23405;PLK1|0.415475;GCH1|4.01735;SOD2|2.175425;TBCA|0.3515;CETN3|0.417[illegible]	membrane-enclosed lumen	GO:0031974	1.55E-12	3.67E-11	52	2
4,5	HSPA8|0.3721;ACTG1|2.1999;LCP1|2.170475;ENO1|2.1939;HIST1H2BN|0.481625;RPL3|3.30745;VIM|2.241075;CCT7|2.06135;PABPC1|0.3285;KRT1|0.4198;MCM3|0.35205;KRT9|0.268525;RPL4|2.063325;MCM2|0.491625;SMC2|0.485025;DNMT1|0.172;HIST1H4A|0.23835;ANXA5|3.637725;GSN|2.4555;NCAPG|0.4528;CBX3|0.485775;GTF2E1|0.300475;PABPC4|0.26245;G3BP1|0.3904;BAZ1B|0.460625;ALDOC|2.04725;ACAT2|2.108325;RPL27|2.15885;SQSTM1|6.007525;PCNA|0.404975;MAPK14|0.483325;HIST1H3A|0.455625;TOP2A|0.481675;SNW1|0.473175;STAT3|0.4995;TUBG1|0.4102;SLC2A1|5.081875;NDE1|0.446425;HBA1|2.13735;NDRG1|10.0073;IK|0.480125;ARHGDIA|3.8215;ACTB|2.1999;HIST2H2AA3|0.29265;H3F3A|0.20985;MYH10|5.579275;TTK|0.488375;PLK1|0.415475;TBCA|0.3515;CETN3|0.4171;CHAF1B|0.2723	intracellular non-membrane-bounded organelle	GO:0043232	1.85E-12	3.91E-11	51	5
3	HSPA8|0.3721;ACTG1|2.1999;LCP1|2.170475;ENO1|2.1939;HIST1H2BN|0.481625;RPL3|3.30745;VIM|2.241075;CCT7|2.06135;PABPC1|0.3285;KRT1|0.4198;MCM3|0.35205;KRT9|0.268525;RPL4|2.063325;MCM2|0.491625;SMC2|0.485025;DNMT1|0.172;HIST1H4A|0.23835;ANXA5|3.637725;GSN|2.4555;NCAPG|0.4528;CBX3|0.485775;GTF2E1|0.300475;PABPC4|0.26245;G3BP1|0.3904;BAZ1B|0.460625;ALDOC|2.04725;ACAT2|2.108325;RPL27|2.15885;SQSTM1|6.007525;PCNA|0.404975;MAPK14|0.483325;HIST1H3A|0.455625;TOP2A|0.481675;SNW1|0.473175;STAT3|0.4995;TUBG1|0.4102;SLC2A1|5.081875;NDE1|0.446425;HBA1|2.13735;NDRG1|10.0073;IK|0.480125;ARHGDIA|3.8215;ACTB|2.1999;HIST2H2AA3|0.29265;H3F3A|0.20985;MYH10|5.579275;TTK|0.488375;PLK1|0.415475;TBCA|	non-membrane-bounded organelle	GO:0043228	1.85E-12	3.91E-11	51	3

5,6	HSPA8|0.3721;ACTG1|2.1999;HSP90AB1|0.458775;ENO1|2.1939;HSPD1|2.88995;HIST1H2BN|0.481625;RPL3|3.30745;PABPC1|0.3285;KRT1|0.4198;YARS|2.288275;MCM3|0.35205;KRT9|0.268525;RPL4|2.063325;MCM2|0.491625;HSPA4L|2.13105;SMC2|0.485025;DNMT1|0.172;HIST1H4A|0.23835;KPNA2|0.186675;VDAC3|2.015475;ANXA5|3.637725;SRRT|0.39875;GSN|2.4555;NCAPG|0.4528;CBX3|0.485775;GTF2E1|0.300475;AKR1B1|2.235325;PABPC4|0.26245;LIG1|0.440425;SLC25A1|2.1121;G3BP1|0.3904;ANKRD28|3.3035;PRDX5|3.0143;BAZ1B|0.460625;ESD|2.155525;PSME3|0.401575;RRM2|0.21675;ACAT2|2.108325;MCMBP|0.4769;RPL27|2.15885;SQSTM1|6.007525;PCNA|0.404975;MAPK14|0.483325;HIST1H3A|0.455625;BTF3|0.2449;TOP2A|0.481675;SNW1|0.473175;STAT3|0.4995;TUBG1|0.4102;NCDN|0.4114;SLC2A1|5.081875;TXNIP|2.0553;IPO8|0.443925;S100A11|2.531875;FAM192A|0.462425;NDRG1|10.0073;IK|0.480125;RBBP7|0.218475;ARHGDIA|3.8215;ACTB|2.1999;DNPEP|2.077575;HIST2H2AA3|0.29265;H3F3A|0.20985;MYH10|5.579275;ALB|0.23405;PLK1|0.415475;GCH1|4.01735;TBCA|0.3515;CETN3|0.4171;ZNF706|0.3974;CHAF1B	nucleus	GO:0005634	2.88E-12	5.78E-11	71	6
2	HSPA8|0.3721;ACTG1|2.1999;HSP90AB1|0.458775;LCP1|2.170475;ENO1|2.1939;HSPD1|2.88995;HIST1H2BN|0.481625;RPL3|3.30745;VIM|2.241075;CCT7|2.06135;PABPC1|0.3285;SARS|2.2366;ASPH|2.59965;KRT1|0.4198;YARS|2.288275;ATP6V1B2|2.0575;MCM3|0.35205;KRT9|0.268525;RPL4|2.063325;NSF|2.017425;MTHFD1L|6.667175;CKB|0.486625;NARS|2.162;MCM2|0.491625;HSPA4L|2.13105;SMC2|0.485025;DNMT1|0.172;ALDH1L2|2.148;HIST1H4A|0.23835;KPNA2|0.186675;VDAC3|2.015475;YWHAG|2.359475;ANXA5|3.637725;SRRT|0.39875;GSN|2.4555;CNDP2|2.0938;NCAPG|0.4528;CBX3|0.485775;GTF2E1|0.300475;AKR1B1|2.235325;PABPC4|0.26245;LIG1|0.440425;SLC25A1|2.1121;G3BP1|0.3904;ERO1L|2.370575;ANKRD28|3.3035;PRDX5|3.0143;BAZ1B|0.460625;ESD|2.155525;ALDOC|2.04725;PSME3|0.401575;RRM2|0.21675;ACAT2|2.108325;LAMTOR2|7.841025;MCMBP|0.4769;RPL27|2.15885;SQSTM1|6.007525;ATG7|2.120975;PCNA|0.404975;MAPK14|0.483325;HIST1H3A|0.455625;BTF3|0.2449;TFRC|0.403125;RHOG|2.449775;TOP2A|0.481675;SNW1|0.473175;STAT3|0.4995;TUBG1|0.4102;NCDN|0.4114;SLC2A1|5.081875;TXNIP|2.0553;VAT1|2.702925;IPO8|0.443925;NDE1|0.446425;S100A11|2.531875;FAM192A|0.462425;HBA1|2.13735;NDRG1|10.0073;IK|0.480125;VTN|2.01125;RBBP7|0.218475;ARHGDIA|3.8215;PLBD2|2.666725;ACTB|2.1999;DNPEP|2.077575;HIST2H2AA3|0.29265;H3F3A|0.20985;MYH10|5.579275;TTK|0.488375;ASL|2.252975;ALB|0.23405;PLK1|0.415475;GCH1|4.01735;SOD2|2.175425;PTP4A3|0.3433;TBCA|0.3515;CETN3|0.4171;Z	organelle	GO:0043226	3.58E-12	6.85E-11	99	2
3	HSPA8|0.3721;HSP90AB1|0.458775;HSPD1|2.88995;HIST1H2BN|0.481625;RPL3|3.30745;ASPH|2.59965;MCM3|0.35205;RPL4|2.063325;MCM2|0.491625;SMC2|0.485025;DNMT1|0.172;HIST1H4A|0.23835;KPNA2|0.186675;SRRT|0.39875;CBX3|0.485775;GTF2E1|0.300475;AKR1B1|2.235325;LIG1|0.440425;ERO1L|2.370575;ANKRD28|3.3035;PRDX5|3.0143;BAZ1B|0.460625;ESD|2.155525;PSME3|0.401575;RRM2|0.21675;ACAT2|2.108325;MCMBP|0.4769;SQSTM1|6.007525;PCNA|0.404975;MAPK14|0.483325;HIST1H3A|0.455625;TOP2A|0.481675;SNW1|0.473175;STAT3|0.4995;TUBG1|0.4102;IPO8|0.443925;S100A11|2.531875;HBA1|2.13735;IK|0.480125;RBBP7|0.218475;PLBD2|2.666725;ACTB|2.1999;DNPEP|2.077575;H3F3A|0.20985;ALB|0.23405;PLK1|0.415475;GCH1|4.01735;SOD2|2.175425;TBCA|0.3515;CETN3|0.4171;CHAF1B|0.2	organelle lumen	GO:0043233	4.24E-12	7.56E-11	51	3

<table>
<tr><td>4</td><td>HSPA8|0.3721;ACTG1|2.1999;HSP90AB1|0.458775;LCP1|2.170475;ENO1|2.1939;HSPD1|2.88995;RPL3|3.30745;VIM|2.241075;CCT7|2.06135;PABPC1|0.3285;SARS|2.2366;ASPH|2.59965;YARS|2.288275;ATP6V1B2|2.0575;MCM3|0.35205;RPL4|2.063325;NSF|2.017425;GMPS|0.496875;MTHFD1L|6.667175;CKB|0.486625;NARS|2.162;MCM2|0.491625;HSPA4L|2.13105;SMC2|0.485025;ALDH1L2|2.148;KPNA2|0.186675;VDAC3|2.015475;YWHAG|2.359475;ANXA5|3.637725;SRRT|0.39875;GSN|2.4555;CNDP2|2.0938;NCAPG|0.4528;AKR1B1|2.235325;PABPC4|0.26245;LIG1|0.440425;SLC25A1|2.1121;G3BP1|0.3904;ERO1L|2.370575;PRDX5|3.0143;ESD|2.155525;ISYNA1|0.49065;ALDOC|2.04725;PSME3|0.401575;RRM2|0.21675;EIF4H|0.275675;ACAT2|2.108325;LAMTOR2|7.841025;MCMBP|0.4769;RPL27|2.15885;SQSTM1|6.007525;ATG7|2.120975;PCNA|0.404975;MAPK14|0.483325;BTF3|0.2449;TFRC|0.403125;RHOG|2.449775;TOP2A|0.481675;STAT3|0.4995;TUBG1|0.4102;NCDN|0.4114;SLC2A1|5.081875;TXNIP|2.0553;VAT1|2.702925;IPO8|0.443925;PLEK|2.071125;NDE1|0.446425;S100A11|2.531875;HBA1|2.13735;NDRG1|10.0073;ARHGDIA|3.8215;PLBD2|2.666725;ACTB|2.1999;DNPEP|2.077575;MYH10|5.579275;SOS2|11.48525;ASL|2.252975;ALB|0.23405;CDC123|0.29925;PLK1|0.415475;GCH1|4.01735;SOD2|2.175425;PTP4A3|0.3433;TBCA|0.3515;CETN3|0.4171;ZNF706|0.3974;CH</td><td>cytoplasm</td><td>GO:0005737</td><td>4.33E-12</td><td>7.56E-11</td><td>87</td><td>4</td></tr>
<tr><td>3,4</td><td>HSPA8|0.3721;ACTG1|2.1999;HSP90AB1|0.458775;LCP1|2.170475;ENO1|2.1939;HSPD1|2.88995;HIST1H2BN|0.481625;RPL3|3.30745;VIM|2.241075;CCT7|2.06135;PABPC1|0.3285;SARS|2.2366;ASPH|2.59965;KRT1|0.4198;YARS|2.288275;ATP6V1B2|2.0575;MCM3|0.35205;KRT9|0.268525;RPL4|2.063325;NSF|2.017425;MTHFD1L|6.667175;CKB|0.486625;NARS|2.162;MCM2|0.491625;HSPA4L|2.13105;SMC2|0.485025;DNMT1|0.172;ALDH1L2|2.148;HIST1H4A|0.23835;KPNA2|0.186675;VDAC3|2.015475;YWHAG|2.359475;ANXA5|3.637725;SRRT|0.39875;GSN|2.4555;NCAPG|0.4528;CBX3|0.485775;GTF2E1|0.300475;AKR1B1|2.235325;PABPC4|0.26245;LIG1|0.440425;SLC25A1|2.1121;G3BP1|0.3904;ERO1L|2.370575;ANKRD28|3.3035;PRDX5|3.0143;BAZ1B|0.460625;ESD|2.155525;ALDOC|2.04725;PSME3|0.401575;RRM2|0.21675;ACAT2|2.108325;LAMTOR2|7.841025;MCMBP|0.4769;RPL27|2.15885;SQSTM1|6.007525;PCNA|0.404975;MAPK14|0.483325;HIST1H3A|0.455625;BTF3|0.2449;TFRC|0.403125;RHOG|2.449775;TOP2A|0.481675;SNW1|0.473175;STAT3|0.4995;TUBG1|0.4102;NCDN|0.4114;SLC2A1|5.081875;TXNIP|2.0553;VAT1|2.702925;IPO8|0.443925;NDE1|0.446425;S100A11|2.531875;FAM192A|0.462425;HBA1|2.13735;NDRG1|10.0073;IK|0.480125;RBBP7|0.218475;ARHGDIA|3.8215;PLBD2|2.666725;ACTB|2.1999;DNPEP|2.077575;HIST2H2AA3|0.29265;H3F3A|0.20985;MYH10|5.579275;TTK|0.488375;ALB|0.23405;PLK1|0.415475;GCH1|4.01735;SOD2|2.175425;PTP4A3|0.3433;TBCA|0.3515;CETN3|0.4171;ZNF706|0.</td><td>intracellular organelle</td><td>GO:0043229</td><td>2.19E-11</td><td>3.66E-10</td><td>95</td><td>4</td></tr>
</table>

3	HSPA8|0.3721;ACTG1|2.1999;HSP90AB1|0.458775;LCP1|2.170475;ENO1|2.1939;HSPD1|2.88995;HIST1H2BN|0.481625;RPL3|3.30745;VIM|2.241075;CCT7|2.06135;PABPC1|0.3285;SARS|2.2366;ASPH|2.59965;KRT1|0.4198;YARS|2.288275;ATP6V1B2|2.0575;MCM3|0.35205;KRT9|0.268525;RPL4|2.063325;NSF|2.017425;GMPS|0.496875;MTHFD1L|6.667175;CKB|0.486625;NARS|2.162;MCM2|0.491625;HSPA4L|2.13105;SMC2|0.485025;DNMT1|0.172;ALDH1L2|2.148;HIST1H4A|0.23835;KPNA2|0.186675;VDAC3|2.015475;YWHAG|2.359475;ANXA5|3.637725;SRRT|0.39875;GSN|2.4555;CNDP2|2.0938;NCAPG|0.4528;CBX3|0.485775;GTF2E1|0.300475;AKR1B1|2.235325;PABPC4|0.26245;LIG1|0.440425;SLC25A1|2.1121;G3BP1|0.3904;ERO1L|2.370575;ANKRD28|3.3035;PRDX5|3.0143;BAZ1B|0.460625;ESD|2.155525;ISYNA1|0.49065;ALDOC|2.04725;PSME3|0.401575;RRM2|0.21675;EIF4H|0.275675;ACAT2|2.108325;LAMTOR2|7.841025;MCMBP|0.4769;RPL27|2.15885;SQSTM1|6.007525;ATG7|2.120975;PCNA|0.404975;MAPK14|0.483325;HIST1H3A|0.455625;BTF3|0.2449;TFRC|0.403125;RHOG|2.449775;TOP2A|0.481675;SNW1|0.473175;STAT3|0.4995;TUBG1|0.4102;NCDN|0.4114;SLC2A1|5.081875;TXNIP|2.0553;VAT1|2.702925;IPO8|0.443925;PLEK|2.071125;NDE1|0.446425;S100A11|2.531875;FAM192A|0.462425;HBA1|2.13735;NDRG1|10.0073;IK|0.480125;RBBP7|0.218475;ARHGDIA|3.8215;PLBD2|2.666725;ACTB|2.1999;DNPEP|2.077575;HIST2H2AA3|0.29265;H3F3A|0.20985;MYH10|5.579275;TTK|0.488375;SOS2|11.48525;ASL|2.252975;ALB|0.23405;CDC123|0.29925;PLK1|0.415475;GCH1|4.01735;SOD2|2.175425;PTP4A3|0.3433;TBCA|0.3515;CETN3|0.4171;Z[illegible]	intracellular part	GO:0044424	3.31E-11	5.31E-10	104	3
5,6	HIST1H2BN|0.481625;MCM3|0.35205;MCM2|0.491625;SMC2|0.485025;DNMT1|0.172;HIST1H4A|0.23835;NCAPG|0.4528;CBX3|0.485775;BAZ1B|0.460625;PCNA|0.404975;HIST1H3A|0.455625;TOP2A|0.481675;SNW1|0.473175;STAT3|0.4995;TUBG1|0.4102;NDE1|0.446425;ACTB|2.1999;HIST2H2AA3|0.29265;H3F3A|0.20985;PLK1|0.415475;CHAF1B|0.2723	chromosome	GO:0005694	3.58E-11	5.54E-10	21	6
4,5	HSPA8|0.3721;HSP90AB1|0.458775;HSPD1|2.88995;HIST1H2BN|0.481625;RPL3|3.30745;ASPH|2.59965;MCM3|0.35205;RPL4|2.063325;MCM2|0.491625;SMC2|0.485025;DNMT1|0.172;HIST1H4A|0.23835;KPNA2|0.186675;SRRT|0.39875;CBX3|0.485775;GTF2E1|0.300475;AKR1B1|2.235325;LIG1|0.440425;ERO1L|2.370575;ANKRD28|3.3035;PRDX5|3.0143;BAZ1B|0.460625;ESD|2.155525;PSME3|0.401575;RRM2|0.21675;ACAT2|2.108325;MCMBP|0.4769;SQSTM1|6.007525;PCNA|0.404975;MAPK14|0.483325;HIST1H3A|0.455625;TOP2A|0.481675;SNW1|0.473175;STAT3|0.4995;TUBG1|0.4102;IPO8|0.443925;S100A11|2.531875;IK|0.480125;RBBP7|0.218475;PLBD2|2.666725;ACTB|2.1999;DNPEP|2.077575;H3F3A|0.20985;PLK1|0.415475;GCH1|4.01735;SOD2|2.175425;TBCA|0.3515;CETN3|0.4171;CHAF1B|0.2723	intracellular organelle lumen	GO:0070013	4.74E-11	7.06E-10	49	5
5,6	HIST1H2BN|0.481625;MCM3|0.35205;MCM2|0.491625;SMC2|0.485025;HIST1H4A|0.23835;CBX3|0.485775;BAZ1B|0.460625;PCNA|0.404975;HIST1H3A|0.455625;TOP2A|0.481675;STAT3|0.4995;TUBG1|0.4102;ACTB|2.1999;H3F3A|0.20985;PLK1|0.415475;CHAF1B|0.2723	nuclear chromosome	GO:0000228	6.39E-11	9.18E-10	16	6
5,6	HSPA8|0.3721;ACTG1|2.1999;LCP1|2.170475;RPL3|3.30745;VIM|2.241075;PABPC1|0.3285;RPL4|2.063325;YWHAG|2.359475;ANXA5|3.637725;GSN|2.4555;G3BP1|0.3904;RPL27|2.15885;RHOG|2.449775;ACTB|2.1999	focal adhesion	GO:0005925	8.66E-11	1.20E-09	14	6
4,5	HSPA8|0.3721;ACTG1|2.1999;LCP1|2.170475;RPL3|3.30745;VIM|2.241075;PABPC1|0.3285;RPL4|2.063325;YWHAG|2.359475;ANXA5|3.637725;GSN|2.4555;G3BP1|0.3904;RPL27|2.15885;RHOG|2.449775;ACTB|2.1999	cell-substrate adherens junction	GO:0005924	1.04E-10	1.39E-09	14	5

3	HSPA8|0.3721;ACTG1|2.1999;LCP1|2.170475;RPL3|3.30745;VIM|2.241075;PABPC1|0.3285;RPL4|2.063325;YWHAG|2.359475;ANXA5|3.637725;GSN|2.4555;G3BP1|0.3904;RPL27|2.15885;RHOG|2.449775;ACTB|2.1999	cell-substrate junction	GO:0030055	1.21E-10	1.56E-09	14	3
4,5	HIST1H2BN|0.481625;MCM3|0.35205;MCM2|0.491625;SMC2|0.485025;DNMT1|0.172;HIST1H4A|0.23835;NCAPG|0.4528;CBX3|0.485775;BAZ1B|0.460625;PCNA|0.404975;HIST1H3A|0.455625;SNW1|0.473175;STAT3|0.4995;NDE1|0.446425;ACTB|2.1999;HIST2H2AA3|0.29265;H3F3A|0.20985;PLK1|0.415475;CHAF1B|0.2723	chromosomal part	GO:0044427	2.42E-10	3.04E-09	19	5
4	HSPA8|0.3721;ACTG1|2.1999;LCP1|2.170475;RPL3|3.30745;VIM|2.241075;PABPC1|0.3285;RPL4|2.063325;YWHAG|2.359475;ANXA5|3.637725;GSN|2.4555;G3BP1|0.3904;RPL27|2.15885;RHOG|2.449775;NDRG1|10.0073;ACTB|2.1999	adherens junction	GO:0005912	3.74E-10	4.56E-09	15	4
3	HSPA8|0.3721;ACTG1|2.1999;LCP1|2.170475;RPL3|3.30745;VIM|2.241075;PABPC1|0.3285;RPL4|2.063325;YWHAG|2.359475;ANXA5|3.637725;GSN|2.4555;G3BP1|0.3904;RPL27|2.15885;RHOG|2.449775;NDRG1|10.0073;ACTB|2.1999	anchoring junction	GO:0070161	6.87E-10	8.12E-09	15	3
3,5,6	HIST1H2BN|0.481625;MCM2|0.491625;DNMT1|0.172;HIST1H4A|0.23835;CBX3|0.485775;BAZ1B|0.460625;HIST1H3A|0.455625;SNW1|0.473175;STAT3|0.4995;ACTB|2.1999;HIST2H2AA3|0.29265;H3F3A|0.20985;PLK1|0.415475;CHAF1B|0.2723	chromatin	GO:0000785	7.78E-10	8.94E-09	14	6
3	HSPA8|0.3721;ACTG1|2.1999;LCP1|2.170475;ENO1|2.1939;HSPD1|2.88995;KRT1|0.4198;YARS|2.288275;KRT9|0.268525;CKB|0.486625;ANXA5|3.637725;GSN|2.4555;AKR1B1|2.235325;PRDX5|3.0143;TFRC|0.403125;SLC2A1|5.081875;S100A11|2.531875;HBA1|2.13735;IK|0.480125;VTN|2.01125;ARHGDIA|3.8215;ACTB|2.1999;DNPEP|2.077575;ALB|0.23405	extracellular space	GO:0005615	1.07E-09	1.19E-08	23	3
5,6	HSPA8|0.3721;HSP90AB1|0.458775;HIST1H2BN|0.481625;RPL3|3.30745;MCM3|0.35205;MCM2|0.491625;SMC2|0.485025;DNMT1|0.172;HIST1H4A|0.23835;KPNA2|0.186675;SRRT|0.39875;CBX3|0.485775;GTF2E1|0.300475;AKR1B1|2.235325;LIG1|0.440425;ANKRD28|3.3035;ESD|2.155525;PSME3|0.401575;RRM2|0.21675;ACAT2|2.108325;MCMBP|0.4769;SQSTM1|6.007525;PCNA|0.404975;MAPK14|0.483325;HIST1H3A|0.455625;TOP2A|0.481675;SNW1|0.473175;STAT3|0.4995;IPO8|0.443925;S100A11|2.531875;IK|0.480125;RBBP7|0.218475;ACTB|2.1999;DNPEP|2.077575;H3F3A|0.20985;PLK1|0.415475;GCH1|4.01735;CHAF1B|0.2723	nucleoplasm	GO:0005654	1.14E-09	1.24E-08	38	6
5,6	HSPA8|0.3721;HSP90AB1|0.458775;HIST1H2BN|0.481625;RPL3|3.30745;MCM3|0.35205;RPL4|2.063325;MCM2|0.491625;SMC2|0.485025;DNMT1|0.172;HIST1H4A|0.23835;KPNA2|0.186675;SRRT|0.39875;CBX3|0.485775;GTF2E1|0.300475;AKR1B1|2.235325;LIG1|0.440425;ANKRD28|3.3035;BAZ1B|0.460625;ESD|2.155525;PSME3|0.401575;RRM2|0.21675;ACAT2|2.108325;MCMBP|0.4769;SQSTM1|6.007525;PCNA|0.404975;MAPK14|0.483325;HIST1H3A|0.455625;TOP2A|0.481675;SNW1|0.473175;STAT3|0.4995;TUBG1|0.4102;IPO8|0.443925;S100A11|2.531875;IK|0.480125;RBBP7|0.218475;ACTB|2.1999;DNPEP|2.077575;H3F3A|0.20985;PLK1|0.415475;GCH1|4.01735;TBCA|0.3515;CETN3|0.4171;CHAF1B|0.2723	nuclear lumen	GO:0031981	2.08E-09	2.20E-08	43	6

2	HSPA8|0.3721;ACTG1|2.1999;HSP90AB1|0.458775;LCP1|2.170475;ENO1|2.1939;HSPD1|2.88995;HIST1H2BN|0.481625;RPL3|3.30745;VIM|2.241075;CCT7|2.06135;PABPC1|0.3285;ASPH|2.59965;KRT1|0.4198;ATP6V1B2|2.0575;MCM3|0.35205;KRT9|0.268525;RPL4|2.063325;MCM2|0.491625;SMC2|0.485025;DNMT1|0.172;HIST1H4A|0.23835;VDAC3|2.015475;GSN|2.4555;NCAPG|0.4528;CBX3|0.485775;PABPC4|0.26245;G3BP1|0.3904;BAZ1B|0.460625;PSME3|0.401575;RRM2|0.21675;EIF4H|0.275675;LAMTOR2|7.841025;MCMBP|0.4769;RPL27|2.15885;SQSTM1|6.007525;PCNA|0.404975;HIST1H3A|0.455625;TFRC|0.403125;TOP2A|0.481675;SNW1|0.473175;STAT3|0.4995;TUBG1|0.4102;NDE1|0.446425;HBA1|2.13735;NDRG1|10.0073;VTN|2.01125;RBBP7|0.218475;ACTB|2.1999;HIST2H2AA3|0.29265;H3F3A|0.20985;MYH10|5.579275;ALB|0.23405;PLK1|0.415475;GCH1|4.01735;TBCA|0.3515;CHAF1B|0.2723	macromolecular complex	GO:0032991	2.30E-09	2.37E-08	56	2
4,5	HSPA8|0.3721;HSP90AB1|0.458775;HSPD1|2.88995;HIST1H2BN|0.481625;RPL3|3.30745;PABPC1|0.3285;MCM3|0.35205;RPL4|2.063325;MCM2|0.491625;SMC2|0.485025;DNMT1|0.172;HIST1H4A|0.23835;KPNA2|0.186675;SRRT|0.39875;CBX3|0.485775;GTF2E1|0.300475;AKR1B1|2.235325;LIG1|0.440425;ANKRD28|3.3035;BAZ1B|0.460625;ESD|2.155525;PSME3|0.401575;RRM2|0.21675;ACAT2|2.108325;MCMBP|0.4769;SQSTM1|6.007525;PCNA|0.404975;MAPK14|0.483325;HIST1H3A|0.455625;TOP2A|0.481675;SNW1|0.473175;STAT3|0.4995;TUBG1|0.4102;IPO8|0.443925;S100A11|2.531875;IK|0.480125;RBBP7|0.218475;ACTB|2.1999;DNPEP|2.077575;H3F3A|0.20985;PLK1|0.415475;GCH1|4.01735;TBCA|0.3515;CETN3|0.4171;CHAF1B|0.2	nuclear part	GO:0044428	1.03E-08	1.03E-07	45	5
4	HIST1H2BN|0.481625;SMC2|0.485025;HIST1H4A|0.23835;NCAPG|0.4528;HIST1H3A|0.455625;HIST2H2AA3|0.29265;H3F3A|0.20985	DNA packaging complex	GO:0044815	4.65E-08	4.56E-07	7	4
3	HSPA8|0.3721;ACTG1|2.1999;HSP90AB1|0.458775;LCP1|2.170475;ENO1|2.1939;HSPD1|2.88995;HIST1H2BN|0.481625;RPL3|3.30745;VIM|2.241075;CCT7|2.06135;PABPC1|0.3285;SARS|2.2366;ASPH|2.59965;KRT1|0.4198;YARS|2.288275;ATP6V1B2|2.0575;MCM3|0.35205;KRT9|0.268525;RPL4|2.063325;NSF|2.017425;GMPS|0.496875;MTHFD1L|6.667175;CKB|0.486625;NARS|2.162;MCM2|0.491625;HSPA4L|2.13105;SMC2|0.485025;DNMT1|0.172;ALDH1L2|2.148;HIST1H4A|0.23835;KPNA2|0.186675;VDAC3|2.015475;YWHAG|2.359475;ANXA5|3.637725;SRRT|0.39875;GSN|2.4555;CNDP2|2.0938;NCAPG|0.4528;CBX3|0.485775;GTF2E1|0.300475;AKR1B1|2.235325;PABPC4|0.26245;LIG1|0.440425;SLC25A1|2.1121;G3BP1|0.3904;ERO1L|2.370575;ANKRD28|3.3035;PRDX5|3.0143;BAZ1B|0.460625;ESD|2.155525;ISYNA1|0.49065;ALDOC|2.04725;PSME3|0.401575;RRM2|0.21675;EIF4H|0.275675;ACAT2|2.108325;LAMTOR2|7.841025;MCMBP|0.4769;RPL27|2.15885;SQSTM1|6.007525;ATG7|2.120975;PCNA|0.404975;MAPK14|0.483325;HIST1H3A|0.455625;BTF3|0.2449;TFRC|0.403125;RHOG|2.449775;TOP2A|0.481675;SNW1|0.473175;STAT3|0.4995;TUBG1|0.4102;NCDN|0.4114;SLC2A1|5.081875;TXNIP|2.0553;VAT1|2.702925;IPO8|0.443925;PLEK|2.071125;NDE1|0.446425;S100A11|2.531875;FAM192A|0.462425;HBA1|2.13735;NDRG1|10.0073;IK|0.480125;RBBP7|0.218475;ARHGDIA|3.8215;PLBD2|2.666725;ACTB|2.1999;DNPEP|2.077575;HIST2H2AA3|0.29265;H3F3A|0.20985;MYH10|5.579275;TTK|0.488375;SOS2|11.48525;ASL|2.252975;ALB|0.23405;CDC123|0.29925;PLK1|0.415475;GCH1|4.01735;SOD2|2.175425;PTP4A3|0.3433;TBCA|0.3515;CETN3|0.4171;Z	intracellular	GO:0005622	1.07E-07	1.02E-06	104	3
5,6,7	HIST1H2BN|0.481625;MCM3|0.35205;MCM2|0.491625;CBX3|0.485775;BAZ1B|0.460625;PCNA|0.404975;HIST1H3A|0.455625;STAT3|0.4995;ACTB|2.1999;H3F3A|0.20985;PLK1|0.415475;CHAF1B|0.2723	nuclear chromosome part	GO:0044454	1.31E-07	1.22E-06	12	7

3	HSPA8|0.3721;ACTG1|2.1999;HSP90AB1|0.458775;LCP1|2.170475;ENO1|2.1939;HSPD1|2.88995;HIST1H2BN|0.481625;VIM|2.241075;CCT7|2.06135;ASPH|2.59965;KRT1|0.4198;ATP6V1B2|2.0575;MCM3|0.35205;KRT9|0.268525;MCM2|0.491625;SMC2|0.485025;HIST1H4A|0.23835;VDAC3|2.015475;GSN|2.4555;NCAPG|0.4528;PSME3|0.401575;RRM2|0.21675;EIF4H|0.275675;LAMTOR2|7.841025;MCMBP|0.4769;PCNA|0.404975;HIST1H3A|0.455625;TFRC|0.403125;TOP2A|0.481675;SNW1|0.473175;STAT3|0.4995;TUBG1|0.4102;NDE1|0.446425;HBA1|2.13735;NDRG1|10.0073;VTN|2.01125;RBBP7|0.218475;ACTB|2.1999;HIST2H2AA3|0.29265;H3F3A|0.20985;MYH10|5.579275;ALB|0.23405;PLK1|0.415475;GCH1|4.01735;TBCA|0.3515;CHAF1B|0.2723	protein complex	GO:0043234	5.41E-07	4.94E-06	46	3
5,6	ACTG1|2.1999;LCP1|2.170475;VIM|2.241075;CCT7|2.06135;KRT1|0.4198;MCM3|0.35205;KRT9|0.268525;MCM2|0.491625;GSN|2.4555;NCAPG|0.4528;CBX3|0.485775;GTF2E1|0.300475;ALDOC|2.04725;PCNA|0.404975;MAPK14|0.483325;TOP2A|0.481675;TUBG1|0.4102;SLC2A1|5.081875;NDE1|0.446425;NDRG1|10.0073;ARHGDIA|3.8215;ACTB|2.1999;MYH10|5.579275;TTK|0.488375;PLK1|0.415475;TBCA|0.3515;CETN3|0.4171	cytoskeleton	GO:0005856	1.81E-06	1.62E-05	27	6
2	HSPA8|0.3721;ACTG1|2.1999;LCP1|2.170475;RPL3|3.30745;VIM|2.241075;PABPC1|0.3285;RPL4|2.063325;YWHAG|2.359475;ANXA5|3.637725;GSN|2.4555;AKR1B1|2.235325;G3BP1|0.3904;RPL27|2.15885;RHOG|2.449775;SLC2A1|5.081875;NDRG1|10.0073;ACTB|2.1999	cell junction	GO:0030054	2.12E-06	1.85E-05	17	2
4,5,6,7	ACTG1|2.1999;LCP1|2.170475;VIM|2.241075;CCT7|2.06135;KRT1|0.4198;MCM3|0.35205;KRT9|0.268525;GSN|2.4555;NCAPG|0.4528;CBX3|0.485775;PCNA|0.404975;MAPK14|0.483325;TOP2A|0.481675;TUBG1|0.4102;SLC2A1|5.081875;NDE1|0.446425;NDRG1|10.0073;MYH10|5.579275;TTK|0.488375;PLK1|0.415475;TBCA|0.3515;CETN3|0.4171	cytoskeletal part	GO:0044430	2.65E-06	2.27E-05	22	7
6,7	SMC2|0.485025;NCAPG|0.4528;CBX3|0.485775;BAZ1B|0.460625;TOP2A|0.481675;TUBG1|0.4102;NDE1|0.446425;PLK1|0.415475	condensed chromosome	GO:0000793	3.03E-06	2.54E-05	8	7
4,5	HIST1H2BN|0.481625;HIST1H4A|0.23835;HIST1H3A|0.455625;HIST2H2AA3|0.29265;H3F3A|0.20985	nucleosome	GO:0000786	1.04E-05	8.53E-05	5	5
3	LCP1|2.170475;VIM|2.241075;ATP6V1B2|2.0575;GSN|2.4555;TUBG1|0.4102;PLEK|2.071125;S100A11|2.531875;MYH10|5.579275	cell leading edge	GO:0031252	1.28E-05	1.03E-04	8	3
5,7,8	DNMT1|0.172;CBX3|0.485775;BAZ1B|0.460625	pericentric heterochromatin	GO:0005721	1.33E-05	1.05E-04	3	8
7	HSPA8|0.3721;HSP90AB1|0.458775;ATP6V1B2|2.0575;TFRC|0.403125;SLC2A1|5.081875	melanosome	GO:0042470	2.18E-05	1.66E-04	5	7
6	HSPA8|0.3721;HSP90AB1|0.458775;ATP6V1B2|2.0575;TFRC|0.403125;SLC2A1|5.081875	pigment granule	GO:0048770	2.18E-05	1.66E-04	5	6
3	HIST1H2BN|0.481625;MCM3|0.35205;HIST1H4A|0.23835;HIST1H3A|0.455625;HIST2H2AA3|0.29265;H3F3A|0.20985	protein-DNA complex	GO:0032993	2.47E-05	1.84E-04	6	3
4	MCM3|0.35205;MCM2|0.491625;MCMBP|0.47[illegible]	MCM complex	GO:0042555	3.29E-05	2.41E-04	3	4
4,6,7,8	HIST1H2BN|0.481625;CBX3|0.485775;HIST1H3A|0.455625;STAT3|0.4995;ACTB|2.1999;H3F3A|0.20985;CHAF1B|0.2723	nuclear chromatin	GO:0000790	4.40E-05	3.15E-04	7	8
5,6	HSP90AB1|0.458775;HSPD1|2.88995;CCT7|2.06135;SARS|2.2366;MTHFD1L|6.667175;CKB|0.486625;NARS|2.162;ALDH1L2|2.148;VDAC3|2.015475;LIG1|0.440425;SLC25A1|2.1121;PRDX5|3.0143;ALDOC|2.04725;MAPK14|0.483325;TFRC|0.403125;STAT3|0.4995;TXNIP|2.0553;VAT1|2.702925;MYH10|5.579275;SOD2|2.175425	mitochondrion	GO:0005739	4.46E-05	3.15E-04	20	6

2	HSPA8|0.3721;ACTG1|2.1999;HSP90AB1|0.458775;LCP1|2.170475;ENO1|2.1939;HSPD1|2.88995;HIST1H2BN|0.481625;RPL3|3.30745;VIM|2.241075;CCT7|2.06135;PABPC1|0.3285;SARS|2.2366;ASPH|2.59965;KRT1|0.4198;YARS|2.288275;ATP6V1B2|2.0575;MCM3|0.35205;KRT9|0.268525;RPL4|2.063325;NSF|2.017425;GMPS|0.496875;MTHFD1L|6.667175;CKB|0.486625;NARS|2.162;MCM2|0.491625;HSPA4L|2.13105;SMC2|0.485025;DNMT1|0.172;ALDH1L2|2.148;HIST1H4A|0.23835;KPNA2|0.186675;VDAC3|2.015475;YWHAG|2.359475;ANXA5|3.637725;SRRT|0.39875;GSN|2.4555;CNDP2|2.0938;NCAPG|0.4528;CBX3|0.485775;GTF2E1|0.300475;AKR1B1|2.235325;PABPC4|0.26245;LIG1|0.440425;SLC25A1|2.1121;G3BP1|0.3904;ERO1L|2.370575;ANKRD28|3.3035;PRDX5|3.0143;BAZ1B|0.460625;ESD|2.155525;ISYNA1|0.49065;ALDOC|2.04725;PSME3|0.401575;RRM2|0.21675;EIF4H|0.275675;ACAT2|2.108325;LAMTOR2|7.841025;MCMBP|0.4769;RPL27|2.15885;SQSTM1|6.007525;ATG7|2.120975;PCNA|0.404975;MAPK14|0.483325;HIST1H3A|0.455625;BTF3|0.2449;TFRC|0.403125;RHOG|2.449775;TOP2A|0.481675;SNW1|0.473175;STAT3|0.4995;TUBG1|0.4102;NCDN|0.4114;SLC2A1|5.081875;TXNIP|2.0553;VAT1|2.702925;IPO8|0.443925;PLEK|2.071125;NDE1|0.446425;S100A11|2.531875;FAM192A|0.462425;HBA1|2.13735;NDRG1|10.0073;IK|0.480125;VTN|2.01125;RBBP7|0.218475;ARHGDIA|3.8215;PLBD2|2.666725;ACTB|2.1999;DNPEP|2.077575;HIST2H2AA3|0.29265;H3F3A|0.20985;MYH10|5.579275;TTK|0.488375;SOS2|11.48525;ASL|2.252975;ALB|0.23405;CDC123|0.29925;PLK1|0.415475;GCH1|4.01735;SOD2|2.175425;PTP4A3|0.3433;TBCA|0.3515;CET	cell part	GO:0044464	4.56E-05	3.16E-04	107	2
5,6,7,8	HIST1H2BN|0.481625;HIST1H3A|0.455625;H3F3A|0.20985	nuclear nucleosome	GO:0000788	5.79E-05	3.95E-04	3	8
2	HSPA8|0.3721;ACTG1|2.1999;HSP90AB1|0.458775;LCP1|2.170475;ENO1|2.1939;HSPD1|2.88995;HIST1H2BN|0.481625;RPL3|3.30745;VIM|2.241075;CCT7|2.06135;PABPC1|0.3285;SARS|2.2366;ASPH|2.59965;KRT1|0.4198;YARS|2.288275;ATP6V1B2|2.0575;MCM3|0.35205;KRT9|0.268525;RPL4|2.063325;NSF|2.017425;GMPS|0.496875;MTHFD1L|6.667175;CKB|0.486625;NARS|2.162;MCM2|0.491625;HSPA4L|2.13105;SMC2|0.485025;DNMT1|0.172;ALDH1L2|2.148;HIST1H4A|0.23835;KPNA2|0.186675;VDAC3|2.015475;YWHAG|2.359475;ANXA5|3.637725;SRRT|0.39875;GSN|2.4555;CNDP2|2.0938;NCAPG|0.4528;CBX3|0.485775;GTF2E1|0.300475;AKR1B1|2.235325;PABPC4|0.26245;LIG1|0.440425;SLC25A1|2.1121;G3BP1|0.3904;ERO1L|2.370575;ANKRD28|3.3035;PRDX5|3.0143;BAZ1B|0.460625;ESD|2.155525;ISYNA1|0.49065;ALDOC|2.04725;PSME3|0.401575;RRM2|0.21675;EIF4H|0.275675;ACAT2|2.108325;LAMTOR2|7.841025;MCMBP|0.4769;RPL27|2.15885;SQSTM1|6.007525;ATG7|2.120975;PCNA|0.404975;MAPK14|0.483325;HIST1H3A|0.455625;BTF3|0.2449;TFRC|0.403125;RHOG|2.449775;TOP2A|0.481675;SNW1|0.473175;STAT3|0.4995;TUBG1|0.4102;NCDN|0.4114;SLC2A1|5.081875;TXNIP|2.0553;VAT1|2.702925;IPO8|0.443925;PLEK|2.071125;NDE1|0.446425;S100A11|2.531875;FAM192A|0.462425;HBA1|2.13735;NDRG1|10.0073;IK|0.480125;VTN|2.01125;RBBP7|0.218475;ARHGDIA|3.8215;PLBD2|2.666725;ACTB|2.1999;DNPEP|2.077575;HIST2H2AA3|0.29265;H3F3A|0.20985;MYH10|5.579275;TTK|0.488375;SOS2|11.48525;ASL|2.252975;ALB|0.23405;CDC123|0.29925;PLK1|0.415475;GCH1|4.01735;SOD2|2.175425;PTP4A3|0.3433;TBCA|0.3515;CET	cell	GO:0005623	9.65E-05	6.47E-04	107	2
6,7	CCT7|2.06135;MCM3|0.35205;MCM2|0.491625;NCAPG|0.4528;CBX3|0.485775;PCNA|0.404975;MAPK14|0.483325;TOP2A|0.481675;TUBG1|0.4102;NDE1|0.446425;NDRG1|10.0073;MYH10|5.579275;TTK|0.488375;PLK1|0.415475;TBCA|0.3515;CETN3|0.4171	microtubule cytoskeleton	GO:0015630	1.00E-04	6.62E-04	16	7
5	PABPC1|0.3285;PABPC4|0.26245;G3BP1|0.3904;SQSTM1|6.007525;ACTB|2.1999	cytoplasmic ribonucleoprotein granule	GO:0036464	1.29E-04	8.38E-04	5	5

4,5,6	PABPC1|0.3285;PABPC4|0.26245;G3BP1|0.3904;SQSTM1|6.007525;ACTB|2.1999	ribonucleoprotein granule	GO:0035770	1.37E-04	8.76E-04	5	6
4	LCP1|2.170475;ATP6V1B2|2.0575;GSN|2.4555;PLEK|2.071125;S100A11|2.531875	ruffle	GO:0001726	1.41E-04	8.89E-04	5	4
5,6	ACTG1|2.1999;ACTB|2.1999	dense body	GO:0097433	1.45E-04	8.97E-04	2	6
6	PABPC1|0.3285;PABPC4|0.26245;G3BP1|0.3904	cytoplasmic stress granule	GO:0010494	1.67E-04	1.02E-03	3	6
5,6,7,8	CBX3|0.485775;MAPK14|0.483325;TUBG1|0.4102;NDE1|0.446425;MYH10|5.579275;TTK|0.488375;PLK1|0.415475	spindle	GO:0005819	1.73E-04	1.04E-03	7	8
5,6,7	ENO1|2.1939;RPL3|3.30745;CCT7|2.06135;RPL4|2.063325;RPL27|2.15885;HBA1|2.13735	cytosolic part	GO:0044445	2.73E-04	1.61E-03	6	7
5,6	MCM3|0.35205;DNMT1|0.172;BAZ1B|0.460625;PCNA|0.404975	replication fork	GO:0005657	4.39E-04	2.56E-03	4	6
5,6	MCM3|0.35205;NCAPG|0.4528;PCNA|0.404975;TOP2A|0.481675;TUBG1|0.4102;NDE1|0.446425;NDRG1|10.0073;PLK1|0.415475;CETN3|0.4171	centrosome	GO:0005813	4.68E-04	2.69E-03	9	6
5,6,7	RPL3|3.30745;RPL4|2.063325;RPL27|2.15885;HBA1|2.13735	cytosolic ribosome	GO:0022626	5.51E-04	3.12E-03	4	7
6,7	DNMT1|0.172;CBX3|0.485775;BAZ1B|0.460625;NDE1|0.446425;PLK1|0.415475	chromosome, centromeric region	GO:0000775	6.94E-04	3.87E-03	5	7
4,5	HSPA8|0.3721;HSP90AB1|0.458775;HSPD1|2.88995;ATP6V1B2|2.0575;YWHAG|2.359475;PRDX5|3.0143;ESD|2.155525;SQSTM1|6.007525;TFRC|0.403125;SLC2A1|5.081875;HBA1|2.13735;ALB|0.23405;GCH1|4.01735	cytoplasmic vesicle	GO:0031410	8.12E-04	4.47E-03	13	5
6,7	RPL3|3.30745;RPL4|2.063325;RPL27|2.15885	cytosolic large ribosomal subunit	GO:0022625	9.02E-04	4.90E-03	3	7
5	SMC2|0.485025;NCAPG|0.4528	condensin complex	GO:0000796	9.97E-04	5.34E-03	2	5
5,6,7,8,9	MAPK14|0.483325;TUBG1|0.4102;NDE1|0.446425;PLK1|0.415475	spindle pole	GO:0000922	1.54E-03	8.17E-03	4	9
6,7,8	MCM3|0.35205;BAZ1B|0.460625;PCNA|0.404975	nuclear replication fork	GO:0043596	1.59E-03	8.31E-03	3	8
5,6	ASPH|2.59965;GSN|2.4555;SLC2A1|5.081875;ACTB|2.1999	cell cortex part	GO:0044448	1.73E-03	8.93E-03	4	6
4	LCP1|2.170475;GSN|2.4555	podosome	GO:0002102	1.79E-03	9.08E-03	2	4
4,6,7	DNMT1|0.172;CBX3|0.485775;BAZ1B|0.46062	heterochromatin	GO:0000792	1.86E-03	9.32E-03	3	7
5,6,7,8	MCM3|0.35205;NCAPG|0.4528;PCNA|0.404975;TOP2A|0.481675;TUBG1|0.4102;NDE1|0.446425;NDRG1|10.0073;PLK1|0.415475;CETN3|0.4	microtubule organizing center	GO:0005815	2.24E-03	1.11E-02	9	8
5,6,9	HSPD1|2.88995;SNW1|0.473175	carboxy-terminal domain protein kinase complex	GO:0032806	2.36E-03	1.15E-02	2	9
6,7	GSN|2.4555;SLC2A1|5.081875;ACTB|2.1999	cortical	GO:0030863	2.38E-03	1.15E-02	3	7
4,5,6	ASPH|2.59965;GSN|2.4555;SLC2A1|5.081875;ACTB|2.1999;MYH10|5.579275	cell cortex	GO:0005938	2.50E-03	1.20E-02	5	6
3,4	HSPA8|0.3721;RPL3|3.30745;PABPC1|0.3285;RPL4|2.063325;PABPC4|0.26245;G3BP1|0.3904;RPL27|2.15885;SQSTM1|6.007525;SNW1|0.473175;HBA1|2.13735;ACTB|2.1999	ribonucleoprotein complex	GO:0030529	2.63E-03	1.24E-02	11	4
5,6	DNMT1|0.172;CBX3|0.485775;BAZ1B|0.460625;NDE1|0.446425;PLK1|0.415475	chromosomal region	GO:0098687	2.80E-03	1.31E-02	5	6
4	PCNA|0.404975	DNA polymerase processivity factor complex	GO:0044796	3.14E-03	1.40E-02	1	4
4	TOP2A|0.481675	viral integration complex	GO:0019035	3.14E-03	1.40E-02	1	4
5	SNW1|0.473175	SMAD3-SMAD4 protein complex	GO:0071146	3.14E-03	1.40E-02	1	5
5	PCNA|0.404975	PCNA complex	GO:0043626	3.14E-03	1.40E-02	1	5
5,6	MCM3|0.35205;GSN|2.4555;AKR1B1|2.235325;PRDX5|3.0143;EIF4H|0.275675;TFRC|0.403125;NDRG1|10.0073	perinuclear region of cytoplasm	GO:0048471	4.37E-03	1.93E-02	7	6
3	HSP90AB1|0.458775;LCP1|2.170475;VIM|2.241075;ATP6V1B2|2.0575;NSF|2.017425;ANXA5|3.637725;GSN|2.4555;AKR1B1|2.235325;ATG7|2.120975;TUBG1|0.4102;NCDN|0.4114;PLEK|2.071125;S100A11|2.531875;MYH10|5.579275	cell projection	GO:0042995	4.55E-03	1.99E-02	14	3
5,6,7	TOP2A|0.481675;TUBG1|0.4102;CETN3|0.4171	centriole	GO:0005814	4.95E-03	2.14E-02	3	7
4,5	ANXA5|3.637725	endothelial microparticle	GO:0072563	6.26E-03	2.62E-02	1	5
4,5,6	RRM2|0.21675	ribonucleoside-diphosphate reductase complex	GO:0005971	6.26E-03	2.62E-02	1	6
4	PCNA|0.404975	PCNA-p21	GO:0070557	6.26E-03	2.62E-02	1	4
4,5	RPL3|3.30745;RPL4|2.063325;RPL27|2.15885;HBA1|2.13735	ribosomal subunit	GO:0044391	6.62E-03	2.74E-02	4	5
7,8	CBX3|0.485775;NDE1|0.446425;PLK1|0.415475	condensed chromosome, centromeric region	GO:0000779	7.07E-03	2.90E-02	3	8
3	SLC2A1|5.081875;MYH10|5.579275;PLK1|0.415475	midbody	GO:0030496	7.88E-03	3.20E-02	3	3

5,6	RPL3|3.30745;RPL4|2.063325;RPL27|2.15885	large ribosomal subunit	GO:0015934	8.05E-03	3.24E-02	3	6
4	CCT7|2.06135;TUBG1|0.4102;NDE1|0.446425;NDRG1|10.0073;PLK1|0.415475;TBCA|0.3515	microtubule	GO:0005874	8.49E-03	3.38E-02	6	4
3,4	NDE1|0.446425;MYH10|5.579275	cell surface furrow	GO:0097610	8.84E-03	3.45E-02	2	4
4,5	NDE1|0.446425;MYH10|5.579275	cleavage furrow	GO:0032154	8.84E-03	3.45E-02	2	5
4	HBA1|2.13735	haptoglobin-hemoglobin complex	GO:0031838	9.38E-03	3.49E-02	1	4
6	MYH10|5.579275	myosin II filament	GO:0097513	9.38E-03	3.49E-02	1	6
5,6	VTN|2.01125	alphav-beta3 integrin-vitronectin complex	GO:0071062	9.38E-03	3.49E-02	1	6
4	CHAF1B|0.2723	CAF-1 complex	GO:0033186	9.38E-03	3.49E-02	1	4
5	AKR1B1|2.235325	Schwann cell microvillus	GO:0097454	9.38E-03	3.49E-02	1	5
3	NDE1|0.446425;MYH10|5.579275	cell division site	GO:0032153	1.00E-02	3.66E-02	2	3
3,4	NDE1|0.446425;MYH10|5.579275	cell division site	GO:0032155	1.00E-02	3.66E-02	2	4
5,6,7	TFRC|0.403125;TUBG1|0.4102;NDRG1|10.007	recycling endosome	GO:0055037	1.06E-02	3.85E-02	3	7
4	HSPD1|2.88995;LAMTOR2|7.841025;EFHD2|2.281;SLC2A1|5.081875	membrane raft	GO:0045121	1.10E-02	3.96E-02	4	4
5,6	TOP2A|0.481675;TUBG1|0.4102;CETN3|0.4171	microtubule organizing center part	GO:0044450	1.12E-02	4.00E-02	3	6
3,4	HSP90AB1|0.458775;LCP1|2.170475;NSF|2.017425;AKR1B1|2.235325;ATG7|2.120975;TUBG1|0.4102;PLEK|2.071125;MYH10|5.579275	cell projection part	GO:0044463	1.15E-02	4.05E-02	8	4
7,8	ATP6V1B2|2.0575;NSF|2.017425;AKR1B1|2.235325;LAMTOR2|7.841025;SQSTM1|6.007525;PLBD2|2.666725	lysosome	GO:0005764	1.18E-02	4.10E-02	6	8
5	HSPA8|0.3721;HSP90AB1|0.458775;HSPD1|2.88995;ATP6V1B2|2.0575;YWHAG|2.359475;ESD|2.155525;TFRC|0.403125;SLC2A1|5.081875;HBA1|2.13735;ALB|0.23405	cytoplasmic membrane-bounded vesicle	GO:0016023	1.21E-02	4.10E-02	10	5
5,6,7,8	TFRC|0.403125;NDRG1|10.0073	recycling endosome membrane	GO:0055038	1.21E-02	4.10E-02	2	8
6,7	ATP6V1B2|2.0575;NSF|2.017425;AKR1B1|2.235325;LAMTOR2|7.841025;SQSTM1|6.007525;PLBD2|2.666725	lytic vacuole	GO:0000323	1.25E-02	4.10E-02	6	7
5,6,7,8,9,10,11	PLK1|0.415475	condensed nuclear chromosome outer kinetochore	GO:0000942	1.25E-02	4.10E-02	1	11
5,6,7,8	GSN|2.4555	actin cap	GO:0030478	1.25E-02	4.10E-02	1	8
4	TOP2A|0.481675	DNA topoisomerase complex (ATP-	GO:0009330	1.25E-02	4.10E-02	1	4
6,8,9	CBX3|0.485775	nuclear pericentric heterochromatin	GO:0031618	1.25E-02	4.10E-02	1	9
6,7,8,9	LCP1|2.170475;MYH10|5.579275	stress fiber	GO:0001725	1.25E-02	4.10E-02	2	9
5,6,7,8	GSN|2.4555;SLC2A1|5.081875	cortical actin cytoskeleton	GO:0030864	1.48E-02	4.77E-02	2	8
5,6,7,8	LCP1|2.170475;MYH10|5.579275	actin filament bundle	GO:0032432	1.48E-02	4.77E-02	2	8
6,7,8,9,10	TUBG1|0.4102	polar microtubule	GO:0005827	1.56E-02	4.86E-02	1	10
4,5	HSPD1|2.88995	lipopolysaccharide receptor complex	GO:0046696	1.56E-02	4.86E-02	1	5
5,6,7,10	SNW1|0.473175	positive transcription elongation factor	GO:0008024	1.56E-02	4.86E-02	1	10
4	AKR1B1|2.235325	paranodal junction	GO:0033010	1.56E-02	4.86E-02	1	4
5,6,7,8	LCP1|2.170475;MYH10|5.579275	actomyosin	GO:0042641	1.73E-02	5.36E-02	2	8
4	HSPA8|0.3721	Prp19 complex	GO:0000974	1.87E-02	5.69E-02	1	4
6,7,8	HSPD1|2.88995	cyclin-dependent protein kinase activating kinase holoenzyme complex	GO:0019907	1.87E-02	5.69E-02	1	8
5,6,7,8	RBBP7|0.218475;ACTB|2.1999	histone methyltransferase complex	GO:0035097	1.89E-02	5.71E-02	2	8
4	PCNA|0.404975	DNA replication factor C complex	GO:0005663	2.18E-02	6.48E-02	1	4
6,7,8	MCM3|0.35205	alpha DNA polymerase:primase complex	GO:0005658	2.18E-02	6.48E-02	1	8
5,6	HSPA8|0.3721;RPL3|3.30745;RPL4|2.063325;ACAT2|2.108325;TOP2A|0.481675;IK|0.480125;PLK1|0.415475;TBCA|0.3515;CETN3|0.4171	nucleolus	GO:0005730	2.39E-02	6.90E-02	9	6
9	HSPA8|0.3721	clathrin-sculpted gamma-aminobutyric acid transport vesicle	GO:0061200	2.48E-02	6.90E-02	1	9
7,8	SLC2A1|5.081875	female pronucleus	GO:0001939	2.48E-02	6.90E-02	1	8
6,7,8,9	ACTB|2.1999	MLL5-L complex	GO:0070688	2.48E-02	6.90E-02	1	9

6,7	ASPH|2.59965	sarcoplasmic reticulum lumen	GO:0033018	2.48E-02	6.90E-02	1	7
4,5,6,7	TFRC|0.403125	HFE-transferrin receptor complex	GO:1990712	2.48E-02	6.90E-02	1	7
4	SNW1|0.473175	SMAD protein complex	GO:0071141	2.48E-02	6.90E-02	1	4
6,7,8,9	HSPA8|0.3721	clathrin-sculpted gamma-aminobutyric acid transport vesicle membrane	GO:0061202	2.48E-02	6.90E-02	1	9
5,6,7	ASPH|2.59965	cortical endoplasmic	GO:0032541	2.48E-02	6.90E-02	1	7
5	ASPH|2.59965;GSN|2.4555	sarcoplasm	GO:0016528	2.51E-02	6.90E-02	2	5
5	LCP1|2.170475;PLEK|2.071125	ruffle membrane	GO:0032587	2.51E-02	6.90E-02	2	5
5,6	HBA1|2.13735;ALB|0.23405	cytoplasmic membrane-bounded vesicle lumen	GO:0060205	2.57E-02	7.02E-02	2	6
4	HBA1|2.13735;ALB|0.23405	vesicle lumen	GO:0031983	2.63E-02	7.14E-02	2	4
3	HSPA8|0.3721;HSP90AB1|0.458775;LCP1|2.170475;HSPD1|2.88995;ATP6V1B2|2.0575;NSF|2.017425;VDAC3|2.015475;LAMTOR2|7.841025;TFRC|0.403125;EFHD2|2.281;SLC2A1|5.081875;VAT1|2.702925;PLEK|2.071125;NDRG1|10.0	whole membrane	GO:0098805	2.65E-02	7.16E-02	14	3
4	HSP90AB1|0.458775;SQSTM1|6.007525	inclusion body	GO:0016234	2.75E-02	7.28E-02	2	4
5,6,7	HSPD1|2.88995	nuclear cyclin-dependent protein kinase holoenzyme complex	GO:0019908	2.79E-02	7.28E-02	1	7
4	EIF4H|0.275675	eukaryotic translation initiation factor 4F	GO:0016281	2.79E-02	7.28E-02	1	4
4	HSPD1|2.88995	mitochondrial	GO:0030061	2.79E-02	7.28E-02	1	4
4	CCT7|2.06135	chaperonin-containing T-complex	GO:0005832	2.79E-02	7.28E-02	1	4
4	ACTG1|2.1999;LCP1|2.170475	actin filament	GO:0005884	2.88E-02	7.43E-02	2	4
6,7	ACTG1|2.1999;LCP1|2.170475;GSN|2.4555;NCAPG|0.4528;SLC2A1|5.081875;MYH10|5.57927	actin cytoskeleton	GO:0015629	2.88E-02	7.43E-02	6	7
5,6,7,8	ASPH|2.59965	junctional sarcoplasmic reticulum membrane	GO:0014701	3.09E-02	7.82E-02	1	8
4,5	LAMTOR2|7.841025	Ragulator complex	GO:0071986	3.09E-02	7.82E-02	1	5
6,7,8,9,10	PLK1|0.415475	condensed nuclear chromosome kinetochore	GO:0000778	3.09E-02	7.82E-02	1	10
4,5	HSPD1|2.88995;VDAC3|2.015475;SLC25A1|2.1121;PRDX5|3.0143;STAT3|0.4995;TXNIP|2.0553;VAT1|2.702925;SOD2|2.175425	mitochondrial part	GO:0044429	3.12E-02	7.82E-02	8	5
5,6,7	PABPC1|0.3285;SNW1|0.473175	catalytic step 2 spliceosome	GO:0071013	3.13E-02	7.82E-02	2	7
5,6,7,8,9	TUBG1|0.4102;PLK1|0.415475	spindle microtubule	GO:0005876	3.26E-02	8.10E-02	2	9
4,5,6	HSPA8|0.3721;PABPC1|0.3285;SNW1|0.473175	spliceosomal complex	GO:0005681	3.33E-02	8.22E-02	3	6
4	CCT7|2.06135	zona pellucida receptor complex	GO:0002199	3.40E-02	8.33E-02	1	4
6,7	VIM|2.241075;KRT1|0.4198;KRT9|0.268525;GTF2E1|0.300475	intermediate filament cytoskeleton	GO:0045111	3.44E-02	8.38E-02	4	7
4	RBBP7|0.218475;ACTB|2.1999	methyltransferase complex	GO:0034708	3.60E-02	8.73E-02	2	4
5,6,7,8	MCM2|0.491625	nuclear origin of replication recognition	GO:0005664	3.70E-02	8.80E-02	1	8
3,4,5	AKR1B1|2.235325	Schmidt-Lanterman incisure	GO:0043220	3.70E-02	8.80E-02	1	5
8	HSPA8|0.3721	clathrin-sculpted vesicle	GO:0060198	3.70E-02	8.80E-02	1	8
3,4	HSPD1|2.88995;TFRC|0.403125	coated pit	GO:0005905	3.74E-02	8.85E-02	2	4
4	HSP90AB1|0.458775;MYH10|5.579275	brush border	GO:0005903	3.88E-02	9.13E-02	2	4
4	AKR1B1|2.235325	glial cell projection	GO:0097386	4.00E-02	9.30E-02	1	4
5,6	ATP6V1B2|2.0575;NSF|2.017425;AKR1B1|2.235325;LAMTOR2|7.841025;SQSTM1|6.007525;PLBD2|2.666725	vacuole	GO:0005773	4.03E-02	9.30E-02	6	6
8	HSPD1|2.88995;SNW1|0.473175	serine/threonine protein kinase complex	GO:1902554	4.03E-02	9.30E-02	2	8
5,8,9	NDE1|0.446425;PLK1|0.415475	condensed chromosome kinetochore	GO:0000777	4.24E-02	9.75E-02	2	9
3,4	AKR1B1|2.235325	compact myelin	GO:0043218	4.30E-02	9.77E-02	1	4
5,7	HSPD1|2.88995	zymogen granule	GO:0042588	4.30E-02	9.77E-02	1	7
4	ATP6V1B2|2.0575;AKR1B1|2.235325	microvillus	GO:0005902	4.47E-02	1.01E-01	2	4

4,5,6	HSPD1|2.88995;LAMTOR2|7.841025;SQSTM1|6.007525;TFRC|0.403125;TUBG1|0.4102;NDRG1|10.0073;PTP4A3|0.3433	endosome	GO:0005768	4.56E-02	1.02E-01	7	6
6,7	CBX3|0.485775	chromocenter	GO:0010369	4.60E-02	1.02E-01	1	7
4	MCM2|0.491625	origin recognition complex	GO:0000808	4.60E-02	1.02E-01	1	4
3	HSP90AB1|0.458775;HSPD1|2.88995;ANXA5|3.637725;TFRC|0.403125;NDE1|0.446425;MYH10|5.579275	cell surface	GO:0009986	4.83E-02	1.07E-01	6	3
6,7	TUBG1|0.4102	pericentriolar material	GO:0000242	4.90E-02	1.07E-01	1	7
6,7	HBA1|2.13735	endocytic vesicle lumen	GO:0071682	4.90E-02	1.07E-01	1	7
5	RBBP7|0.218475	ESC/E(Z) complex	GO:0035098	4.90E-02	1.07E-01	1	5
3	HSP90AB1|0.458775;LCP1|2.170475;HSPD1|2.88995;LAMTOR2|7.841025;TFRC|0.403125;EFHD2|2.281;SLC2A1|5.081875;PLEK|2.071125	membrane region	GO:0098589	4.94E-02	1.07E-01	8	3
4	HBA1|2.13735	hemoglobin complex	GO:0005833	5.20E-02	1.12E-01	1	4
6,7	ACTG1|2.1999;ENO1|2.1939;ANXA5|3.637725	myofibril	GO:0030016	5.39E-02	1.13E-01	3	7
7	HSPD1|2.88995;SNW1|0.473175	protein kinase complex	GO:1902911	5.48E-02	1.13E-01	2	7
4	PSME3|0.401575	proteasome activator complex	GO:0008537	5.50E-02	1.13E-01	1	4
4	TUBG1|0.4102	gamma-tubulin complex	GO:0000930	5.50E-02	1.13E-01	1	4
4	LCP1|2.170475	phagocytic cup	GO:0001891	5.50E-02	1.13E-01	1	4
6,7	SLC2A1|5.081875	pronucleus	GO:0045120	5.50E-02	1.13E-01	1	7
5,6,7	ASPH|2.59965	endoplasmic reticulum tubular network	GO:0071782	5.50E-02	1.13E-01	1	7
4	PLK1|0.415475	condensed chromosome outer kinetochore	GO:0000940	5.50E-02	1.13E-01	1	4
2	HSPA8|0.3721;ACTG1|2.1999;HSP90AB1|0.458775;LCP1|2.170475;ENO1|2.1939;HSPD1|2.88995;VIM|2.241075;PABPC1|0.3285;ASPH|2.59965;KRT1|0.4198;ATP6V1B2|2.0575;MCM3|0.35205;KRT9|0.268525;RPL4|2.063325;NSF|2.017425;MTHFD1L|6.667175;HIST1H4A|0.23835;KPNA2|0.186675;VDAC3|2.015475;YWHAG|2.359475;ANXA5|3.637725;GSN|2.4555;NCAPG|0.4528;CBX3|0.485775;SLC25A1|2.1121;G3BP1|0.3904;ERO1L|2.370575;ESD|2.155525;PSME3|0.401575;EIF4H|0.275675;LAMTOR2|7.841025;MCMBP|0.4769;RPL27|2.15885;TM9SF4|2.3766;HIST1H3A|0.455625;TFRC|0.403125;RHOG|2.449775;STAT3|0.4995;FNDC3B|2.328575;EFHD2|2.281;NCDN|0.4114;SLC2A1|5.081875;VAT1|2.702925;PLEK|2.071125;NDE1|0.446425;HBA1|2.13735;NDRG1|10.0073;VTN|2.01125;ARHGDIA|3.8215;ACTB|2.1999;MYH10|5.579275;TTK|0.488375;MPEG1|5.8062;GCH1|4.01735;PTP4A3|0.3433;IFITM2|5.68315;PLP1|3.936	membrane	GO:0016020	5.66E-02	1.16E-01	57	2
3	HSPA8|0.3721;ACTG1|2.1999;HSP90AB1|0.458775;LCP1|2.170475;ENO1|2.1939;HSPD1|2.88995;VIM|2.241075;ASPH|2.59965;KRT1|0.4198;ATP6V1B2|2.0575;NSF|2.017425;ANXA5|3.637725;GSN|2.4555;G3BP1|0.3904;ESD|2.155525;MCMBP|0.4769;TFRC|0.403125;RHOG|2.449775;STAT3|0.4995;SLC2A1|5.081875;PLEK|2.071125;NDRG1|10.0073;VTN|2.01125;ARHGDIA|3.8215;ACTB|2.1999;MYH10|5.579275;PTP4A3|0.3433;IFITM2|5.68315;PLP1|3.936	plasma membrane	GO:0005886	5.75E-02	1.17E-01	29	3
4,5	HSP90AB1|0.458775;LCP1|2.170475;PLEK|2.071125	cell projection membrane	GO:0031253	5.83E-02	1.18E-01	3	5
5,6	ACTG1|2.1999;ENO1|2.1939;ANXA5|3.637725	contractile fiber	GO:0043292	5.98E-02	1.21E-01	3	6
4	HSPD1|2.88995	cyclin-dependent protein kinase holoenzyme complex	GO:0000307	6.09E-02	1.22E-01	1	4
5,6,7,8	ENO1|2.1939	phosphopyruvate hydratase complex	GO:0000015	6.09E-02	1.22E-01	1	8
4,5,6	AKR1B1|2.235325;ATG7|2.120975	cell projection cytoplasm	GO:0032838	6.31E-02	1.26E-01	2	6
4	LCP1|2.170475;PLEK|2.071125	leading edge membrane	GO:0031256	6.39E-02	1.27E-01	2	4
4	VIM|2.241075;KRT1|0.4198;KRT9|0.268525	intermediate filament	GO:0005882	6.55E-02	1.28E-01	3	4
5,6	HSPD1|2.88995;VDAC3|2.015475;SLC25A1|2.1121;STAT3|0.4995;TXNIP|2.0553;VAT1|2.7029	mitochondrial envelope	GO:0005740	6.57E-02	1.28E-01	6	6
6,7	NDE1|0.446425	spindle pole centrosome	GO:0031616	6.68E-02	1.28E-01	1	7
4,5,6,8,9,10	ENO1|2.1939	M band	GO:0031430	6.68E-02	1.28E-01	1	10

5,6	ASPH|2.59965	endoplasmic reticulum subcompartment	GO:0098827	6.68E-02	1.28E-01	1	6
5	MYH10|5.579275	myosin filament	GO:0032982	6.68E-02	1.28E-01	1	5
8,9	AKR1B1|2.235325	mast cell granule	GO:0042629	6.68E-02	1.28E-01	1	9
6,7,8	TUBG1|0.4102;PLK1|0.415475	condensed nuclear chromosome	GO:0000794	6.91E-02	1.31E-01	2	8
5,6	ATP6V1B2|2.0575	proton-transporting V-type ATPase, V1 domain	GO:0033180	6.97E-02	1.31E-01	1	6
4	ACTG1|2.1999	filamentous actin	GO:0031941	6.97E-02	1.31E-01	1	4
3	ANXA5|3.637725;AKR1B1|2.235325;SLC2A1|5.081875;NDRG1|10.0073	cell-cell junction	GO:0005911	6.99E-02	1.31E-01	4	3
3	HSPA8|0.3721;ACTG1|2.1999;HSP90AB1|0.458775;LCP1|2.170475;ENO1|2.1939;HSPD1|2.88995;VIM|2.241075;ASPH|2.59965;KRT1|0.4198;ATP6V1B2|2.0575;NSF|2.017425;ANXA5|3.637725;GSN|2.4555;G3BP1|0.3904;ESD|2.155525;MCMBP|0.4769;TFRC|0.403125;RHOG|2.449775;STAT3|0.4995;SLC2A1|5.081875;PLEK|2.071125;NDRG1|10.0073;VTN|2.01125;ARHGDIA|3.8215;ACTB|2.1999;MYH10|5.579275;PTP4A3|0.3433;IFITM2|5.68315;PLP1|3.936	cell periphery	GO:0071944	7.10E-02	1.33E-01	29	3
5,6,7	CBX3|0.485775	nuclear	GO:0005719	7.26E-02	1.35E-01	1	7
5,6	SQSTM1|6.007525;ATG7|2.120975	pre-autophagosomal	GO:0000407	7.35E-02	1.36E-01	2	6
5	MYH10|5.579275	myosin II complex	GO:0016460	7.56E-02	1.39E-01	1	5
4	NDE1|0.446425;PLK1|0.415475	kinetochore	GO:0000776	7.62E-02	1.40E-01	2	4
8	ACTB|2.1999	NuA4 histone acetyltransferase complex	GO:0035267	7.85E-02	1.43E-01	1	8
5,6,8,9	ATP6V1B2|2.0575;NSF|2.017425;LAMTOR2|7.841025	lysosomal membrane	GO:0005765	8.10E-02	1.45E-01	3	9
5	RBBP7|0.218475	CHD-type complex	GO:0090545	8.13E-02	1.45E-01	1	5
6,7,8,9	RBBP7|0.218475	NuRD complex	GO:0016581	8.13E-02	1.45E-01	1	9
6	ACTB|2.1999	H4 histone acetyltransferase complex	GO:1902562	8.13E-02	1.45E-01	1	6
7	ACTB|2.1999	H4/H2A histone acetyltransferase complex	GO:0043189	8.13E-02	1.45E-01	1	7
3	HSPA8|0.3721;HSPD1|2.88995;ASPH|2.59965;ATP6V1B2|2.0575;NSF|2.017425;VDAC3|2.015475;YWHAG|2.359475;CBX3|0.485775;SLC25A1|2.1121;ERO1L|2.370575;LAMTOR2|7.841025;TFRC|0.403125;RHOG|2.449775;STAT3|0.4995;SLC2A1|5.081875;VAT1|2.702925;NDRG1|10.0073;GCH1|4.01735	organelle membrane	GO:0031090	8.72E-02	1.55E-01	18	3
4,5,6,7	ATP6V1B2|2.0575;NSF|2.017425;LAMTOR2|7.841025;PLBD2|2.666725	vacuolar part	GO:0044437	8.77E-02	1.55E-01	4	7
5,6,7	RBBP7|0.218475	nuclear transcriptional repressor complex	GO:0090568	9.00E-02	1.57E-01	1	7
6,7,8,9	PLK1|0.415475	condensed nuclear chromosome, centromeric region	GO:0000780	9.00E-02	1.57E-01	1	9
4,6,7	CBX3|0.485775	euchromatin	GO:0000791	9.00E-02	1.57E-01	1	7
4	GSN|2.4555;MYH10|5.579275	lamellipodium	GO:0030027	9.21E-02	1.60E-01	2	4
4,5	HSPD1|2.88995;VDAC3|2.015475;CBX3|0.485775;SLC25A1|2.1121;STAT3|0.4995;TXNIP|2.0553;VAT1|2.702925;GCH1|4.01735	organelle envelope	GO:0031967	9.28E-02	1.61E-01	8	5
3	HSPD1|2.88995;VDAC3|2.015475;CBX3|0.485775;SLC25A1|2.1121;STAT3|0.4995;TXNIP|2.0553;VAT1|2.702925;GCH1|4.01735	envelope	GO:0031975	9.39E-02	1.62E-01	8	3
4	ARHGDIA|3.8215	immunological synapse	GO:0001772	9.85E-02	1.69E-01	1	4
5	HSPA8|0.3721;HSPD1|2.88995;MCM3|0.35205;SNW1|0.473175;RBBP7|0.218475;ACTB|2.1999	transferase complex	GO:1990234	1.01E-01	1.73E-01	6	5
4,5,7,8,9	ENO1|2.1939	A band	GO:0031672	1.04E-01	1.77E-01	1	9
5,6	VIM|2.241075;PRDX5|3.0143	microbody	GO:0042579	1.05E-01	1.77E-01	2	6
6,7	VIM|2.241075;PRDX5|3.0143	peroxisome	GO:0005777	1.05E-01	1.77E-01	2	7
5,7,8	CBX3|0.485775	nuclear heterochromatin	GO:0005720	1.07E-01	1.80E-01	1	8
5,6,7,8,9	PLK1|0.415475	spindle midzone	GO:0051233	1.10E-01	1.83E-01	1	9
4,5	ATP6V1B2|2.0575	proton-transporting two-sector ATPase complex, catalytic domain	GO:0033178	1.10E-01	1.83E-01	1	5
4,5,6	RPL3|3.30745;RPL4|2.063325;RPL27|2.15885;HBA1|2.13735	ribosome	GO:0005840	1.12E-01	1.85E-01	4	6
6,7	PRDX5|3.0143	peroxisomal matrix	GO:0005782	1.13E-01	1.85E-01	1	7
5,6	PRDX5|3.0143	microbody lumen	GO:0031907	1.13E-01	1.85E-01	1	6
5,6,7,8	MCM3|0.35205	nuclear replisome	GO:0043601	1.18E-01	1.94E-01	1	8

5,6	ASPH|2.59965;ERO1L|2.370575	endoplasmic reticulum lumen	GO:0005788	1.25E-01	2.05E-01	2	6
4,5,6,7	MCM3|0.35205	replisome	GO:0030894	1.26E-01	2.06E-01	1	7
4	RBBP7|0.218475	PcG protein complex	GO:0031519	1.29E-01	2.09E-01	1	4
4,6,7	HSPD1|2.88995;VDAC3|2.015475;SLC25A1|2.1121;STAT3|0.4995;VAT1|2.702925	mitochondrial membrane	GO:0031966	1.29E-01	2.09E-01	5	7
5	VDAC3|2.015475;VAT1|2.702925	mitochondrial outer membrane	GO:0005741	1.33E-01	2.13E-01	2	5
4	HSP90AB1|0.458775	COP9 signalosome	GO:0008180	1.35E-01	2.14E-01	1	4
2	TOP2A|0.481675	nucleoid	GO:0009295	1.35E-01	2.14E-01	1	2
4,5,6,7	ASPH|2.59965	sarcoplasmic reticulum membrane	GO:0033017	1.35E-01	2.14E-01	1	7
4	HSPA8|0.3721;ENO1|2.1939;HSPD1|2.88995;MCM3|0.35205;RRM2|0.21675;SNW1|0.473175;RBBP7|0.218475;ACTB|2.1999	catalytic complex	GO:1902494	1.37E-01	2.16E-01	8	4
5	SQSTM1|6.007525	aggresome	GO:0016235	1.37E-01	2.16E-01	1	5
6,7,8	HBA1|2.13735	cytosolic small ribosomal subunit	GO:0022627	1.37E-01	2.16E-01	1	8
6,8	ALB|0.23405	platelet alpha granule lumen	GO:0031093	1.40E-01	2.19E-01	1	8
5	HSP90AB1|0.458775	brush border membrane	GO:0031526	1.43E-01	2.22E-01	1	5
5,6	HSPD1|2.88995;PRDX5|3.0143;SOD2|2.175425	mitochondrial matrix	GO:0005759	1.43E-01	2.22E-01	3	6
6	HSPD1|2.88995;MCM3|0.35205;SNW1|0.473175	transferase complex, transferring phosphorus-containing groups	GO:0061695	1.54E-01	2.38E-01	3	6
5	ANXA5|3.637725	intercalated disc	GO:0014704	1.56E-01	2.40E-01	1	5
4	VDAC3|2.015475;VAT1|2.702925	organelle outer membrane	GO:0031968	1.58E-01	2.43E-01	2	4
4	NSF|2.017425	dendritic shaft	GO:0043198	1.64E-01	2.51E-01	1	4
3	VDAC3|2.015475;VAT1|2.702925	outer membrane	GO:0019867	1.65E-01	2.51E-01	2	3
4	ANXA5|3.637725;TFRC|0.403125	external side of plasma membrane	GO:0009897	1.67E-01	2.54E-01	2	4
4,5	ATP6V1B2|2.0575;NSF|2.017425;LAMTOR2|7.841025	vacuolar membrane	GO:0005774	1.76E-01	2.66E-01	3	5
5,7	ALB|0.23405	secretory granule lumen	GO:0034774	1.77E-01	2.67E-01	1	7
4,5	HSP90AB1|0.458775;SLC2A1|5.081875	basolateral plasma membrane	GO:0016323	1.79E-01	2.68E-01	2	5
4,6,7,8	ENO1|2.1939;ANXA5|3.637725	sarcomere	GO:0030017	1.79E-01	2.68E-01	2	8
5,7	ALB|0.23405	platelet alpha granule	GO:0031091	1.80E-01	2.68E-01	1	7
6,7,8	PLK1|0.415475	synaptonemal complex	GO:0000795	1.82E-01	2.70E-01	1	8
5,6,7	LAMTOR2|7.841025;SQSTM1|6.007525	late endosome	GO:0005770	1.85E-01	2.74E-01	2	7
4	HSPD1|2.88995;CBX3|0.485775;SLC25A1|2.1121;STAT3|0.4995	organelle inner membrane	GO:0019866	1.86E-01	2.74E-01	4	4
5	NSF|2.017425;NCDN|0.4114;MYH10|5.579275	dendrite	GO:0030425	1.87E-01	2.74E-01	3	5
5	TUBG1|0.4102	cytoplasmic microtubule	GO:0005881	1.87E-01	2.74E-01	1	5
4	ANXA5|3.637725	cell-cell contact zone	GO:0044291	1.90E-01	2.77E-01	1	4
3	CCT7|2.06135;NCDN|0.4114;MYH10|5.579275	cell body	GO:0044297	1.95E-01	2.84E-01	3	3
4,5	NDRG1|10.0073	cell-cell adherens junction	GO:0005913	1.98E-01	2.86E-01	1	5
3,5,6,7	ENO1|2.1939;ANXA5|3.637725	contractile fiber	GO:0044449	2.00E-01	2.88E-01	2	7
5,6,7	HSPD1|2.88995	rough endoplasmic reticulum	GO:0005791	2.03E-01	2.91E-01	1	7
5,6	ASPH|2.59965	sarcoplasmic reticulum	GO:0016529	2.05E-01	2.92E-01	1	6
4	SNW1|0.473175	transcription elongation factor complex	GO:0008023	2.05E-01	2.92E-01	1	4
4,5	HSPA8|0.3721;YWHAG|2.359475;HBA1|2.13735;ALB|0.23405	cytoplasmic vesicle part	GO:0044433	2.06E-01	2.93E-01	4	5
6,7,8,9	PLBD2|2.666725	lysosomal lumen	GO:0043202	2.13E-01	3.01E-01	1	9
5,6,7,8	RBBP7|0.218475	histone deacetylase complex	GO:0000118	2.15E-01	3.03E-01	1	8
5,6	ATP6V1B2|2.0575	proton-transporting V-type ATPase complex	GO:0033176	2.18E-01	3.06E-01	1	6
6	SQSTM1|6.007525	cytoplasmic mRNA processing body	GO:0000932	2.20E-01	3.07E-01	1	6
5,6	SLC2A1|5.081875	caveola	GO:0005901	2.20E-01	3.07E-01	1	6
5,6,7	PLBD2|2.666725	vacuolar lumen	GO:0005775	2.22E-01	3.09E-01	1	7
4	PSME3|0.401575	proteasome accessory complex	GO:0022624	2.27E-01	3.15E-01	1	4

4,5,6,7	TXNIP|2.0553	mitochondrial intermembrane space	GO:0005758	2.30E-01	3.17E-01	1	7
4,5	LAMTOR2|7.841025;TFRC|0.403125;NDRG1|10.0073	endosome membrane	GO:0010008	2.34E-01	3.22E-01	3	5
4	LCP1|2.170475	filopodium	GO:0030175	2.35E-01	3.22E-01	1	4
4	RBBP7|0.218475	transcriptional repressor complex	GO:0017053	2.37E-01	3.24E-01	1	4
4,5	SLC2A1|5.081875	plasma membrane raft	GO:0044853	2.39E-01	3.26E-01	1	5
3	MYH10|5.579275	neuromuscular junction	GO:0031594	2.42E-01	3.28E-01	1	3
3,4,5	TXNIP|2.0553	organelle envelope lumen	GO:0031970	2.49E-01	3.37E-01	1	5
7,8,9	SQSTM1|6.007525	PML body	GO:0016605	2.54E-01	3.42E-01	1	9
4	HSPA8|0.3721;ASPH|2.59965;ATP6V1B2|2.0575;NSF|2.017425;VDAC3|2.015475;ERO1L|2.370575;LAMTOR2|7.841025;TFRC|0.403125;RHOG|2.449775;SLC2A1|5.081875;VAT1|2.702925;NDRG1|10.0073	bounding membrane of organelle	GO:0098588	2.58E-01	3.47E-01	12	4
4,5	LAMTOR2|7.841025;TFRC|0.403125;NDRG1|10.0073	endosomal part	GO:0044440	2.63E-01	3.52E-01	3	5
4,5	PRDX5|3.0143	microbody part	GO:0044438	2.65E-01	3.53E-01	1	5
5,6	PRDX5|3.0143	peroxisomal part	GO:0044439	2.65E-01	3.53E-01	1	6
5,6,7	SNW1|0.473175	nuclear matrix	GO:0016363	2.70E-01	3.58E-01	1	7
3	MYH10|5.579275	dendritic spine	GO:0043197	2.81E-01	3.71E-01	1	3
6,7	SQSTM1|6.007525	autophagosome	GO:0005776	2.84E-01	3.71E-01	1	7
4,5,6	TUBG1|0.4102	ciliary basal body	GO:0036064	2.84E-01	3.71E-01	1	6
5,6	HBA1|2.13735	small ribosomal subunit	GO:0015935	2.84E-01	3.71E-01	1	6
5	MYH10|5.579275	neuron spine	GO:0044309	2.86E-01	3.73E-01	1	5
5	HSPD1|2.88995;SLC25A1|2.1121;STAT3|0.4995	mitochondrial inner membrane	GO:0005743	2.91E-01	3.78E-01	3	5
4,5	ATG7|2.120975	ciliary cytoplasm	GO:0097014	2.93E-01	3.78E-01	1	5
4,5,6	ATG7|2.120975	axoneme	GO:0005930	2.93E-01	3.78E-01	1	6
5,6,7,8	CBX3|0.485775	nuclear inner membrane	GO:0005637	2.99E-01	3.86E-01	1	8
6,7,8	STAT3|0.4995	RNA polymerase II transcription factor complex	GO:0090575	3.01E-01	3.87E-01	1	8
4,6	HSPD1|2.88995;ALB|0.23405	secretory granule	GO:0030141	3.03E-01	3.88E-01	2	6
6	HSPA8|0.3721;HSPD1|2.88995	coated vesicle	GO:0030135	3.05E-01	3.89E-01	2	6
4	ANXA5|3.637725	sarcolemma	GO:0042383	3.06E-01	3.89E-01	1	4
4,5	ATP6V1B2|2.0575	proton-transporting two-sector ATPase complex	GO:0016469	3.10E-01	3.93E-01	1	5
5,6,7,8	HSPA8|0.3721	clathrin-coated vesicle membrane	GO:0030665	3.14E-01	3.98E-01	1	8
5,6	SNW1|0.473175	nuclear periphery	GO:0034399	3.17E-01	3.99E-01	1	6
3	HSP90AB1|0.458775;TUBG1|0.4102	apical part of cell	GO:0045177	3.20E-01	4.02E-01	2	3
5,6,7	SQSTM1|6.007525;SNW1|0.473175;RBBP7|0.218475;ACTB|2.1999	nucleoplasm part	GO:0044451	3.24E-01	4.06E-01	4	7
5,6,7	HSPD1|2.88995;PTP4A3|0.3433	early endosome	GO:0005769	3.27E-01	4.08E-01	2	7
3,4,5	ATG7|2.120975;TUBG1|0.4102	ciliary part	GO:0044441	3.34E-01	4.15E-01	2	5
5	ACTB|2.1999	histone acetyltransferase complex	GO:0000123	3.38E-01	4.18E-01	1	5
5	KRT1|0.4198	keratin filament	GO:0045095	3.38E-01	4.18E-01	1	5
5,6,7,8	LAMTOR2|7.841025	late endosome membrane	GO:0031902	3.40E-01	4.19E-01	1	8
4,5,6,8,9,10	ANXA5|3.637725	Z disc	GO:0030018	3.44E-01	4.23E-01	1	10
4,5	VDAC3|2.015475	pore complex	GO:0046930	3.46E-01	4.24E-01	1	5
5	NCDN|0.4114;MYH10|5.579275	axon	GO:0030424	3.49E-01	4.25E-01	2	5
3	RRM2|0.21675	oxidoreductase complex	GO:1990204	3.50E-01	4.25E-01	1	3
5,6,7	NSF|2.017425	Golgi stack	GO:0005795	3.50E-01	4.25E-01	1	7
4	MYH10|5.579275	growth cone	GO:0030426	3.54E-01	4.29E-01	1	4
7	ASPH|2.59965	calcium channel complex	GO:0034704	3.58E-01	4.33E-01	1	7
3	HSPD1|2.88995;ASPH|2.59965;ATP6V1B2|2.0575;NSF|2.017425;CBX3|0.485775;LIG1|0.440425;ERO1L|2.370575;ESD|2.155525;LAMTOR2|7.841025;SQSTM1|6.007525;TFRC|0.403125;RHOG|2.449775;TUBG1|0.4102;SLC2A1|5.081875;NDRG1|10.0073;ALB|0.23405;GCH1|4.01735;P	endomembrane system	GO:0012505	3.61E-01	4.33E-01	18	3
6	ACTB|2.1999	acetyltransferase complex	GO:1902493	3.62E-01	4.33E-01	1	6
4	ACTB|2.1999	protein acetyltransferase complex	GO:0031248	3.62E-01	4.33E-01	1	4
4,5	ASPH|2.59965;NSF|2.017425	organelle subcompartment	GO:0031984	3.64E-01	4.33E-01	2	5
3	MYH10|5.579275	site of polarized growth	GO:0030427	3.64E-01	4.33E-01	1	3

4	NSF|2.017425;NCDN|0.4114;MYH10|5.579275	somatodendritic compartment	GO:0036477	3.65E-01	4.33E-01	3	4
4,5,6,7	CBX3|0.485775;GCH1|4.01735	nuclear membrane	GO:0031965	3.67E-01	4.34E-01	2	7
4	MYH10|5.579275	myosin complex	GO:0016459	3.70E-01	4.37E-01	1	4
4,5,7,8,9	ANXA5|3.637725	I band	GO:0031674	3.74E-01	4.40E-01	1	9
4	VIM|2.241075;NSF|2.017425;NCDN|0.4114;MYH10|5.579275	neuron projection	GO:0043005	3.75E-01	4.40E-01	4	4
5,6	TUBG1|0.4102	nonmotile primary cilium	GO:0031513	3.82E-01	4.47E-01	1	6
5,6,7	STAT3|0.4995	nuclear transcription factor	GO:0044798	3.84E-01	4.48E-01	1	7
4	RBBP7|0.218475	SWI/SNF superfamily-type complex	GO:0070603	3.86E-01	4.49E-01	1	4
4	NCDN|0.4114;MYH10|5.579275	neuronal cell body	GO:0043025	3.95E-01	4.57E-01	2	4
5	NDE1|0.446425	kinesin complex	GO:0005871	4.18E-01	4.82E-01	1	5
3	NSF|2.017425;MYH10|5.579275	postsynapse	GO:0098794	4.20E-01	4.82E-01	2	3
4	HSPD1|2.88995;TFRC|0.403125	receptor complex	GO:0043235	4.20E-01	4.82E-01	2	4
4	HSP90AB1|0.458775;LCP1|2.170475;SLC2A1|5.081875;PLEK|2.071125	plasma membrane region	GO:0098590	4.26E-01	4.88E-01	4	4
4	SNW1|0.473175;STAT3|0.4995	transcription factor complex	GO:0005667	4.29E-01	4.90E-01	2	4
4	PSME3|0.401575	proteasome complex	GO:0000502	4.34E-01	4.95E-01	1	4
7	HSPA8|0.3721	clathrin-coated vesicle	GO:0030136	4.67E-01	5.31E-01	1	7
5	MCM3|0.35205	DNA-directed RNA polymerase complex	GO:0000428	4.74E-01	5.35E-01	1	5
5,6	MCM3|0.35205	nuclear DNA-directed RNA polymerase complex	GO:0055029	4.74E-01	5.35E-01	1	6
4	MCM3|0.35205	RNA polymerase complex	GO:0030880	4.77E-01	5.38E-01	1	4
3,4	HSPD1|2.88995;ASPH|2.59965;ATP6V1B2|2.0575;VDAC3|2.015475;LAMTOR2|7.841025;TFRC|0.403125;VTN|2.01125	membrane protein complex	GO:0098796	4.85E-01	5.44E-01	7	4
4,5	TUBG1|0.4102	primary cilium	GO:0072372	5.08E-01	5.69E-01	1	5
3	NSF|2.017425	postsynaptic	GO:0014069	5.22E-01	5.83E-01	1	3
5,6	HSPA8|0.3721;YWHAG|2.359475	cytoplasmic vesicle membrane	GO:0030659	5.37E-01	5.98E-01	2	6
4,5,6	ASPH|2.59965	integral component of endoplasmic reticulum membrane	GO:0030176	5.44E-01	6.04E-01	1	6
3	HSPD1|2.88995	mitochondrial membrane part	GO:0044455	5.47E-01	6.05E-01	1	3
4,5,6	ASPH|2.59965	intrinsic component of endoplasmic reticulum membrane	GO:0031227	5.51E-01	6.09E-01	1	6
4	HSPA8|0.3721;YWHAG|2.359475	vesicle membrane	GO:0012506	5.53E-01	6.09E-01	2	4
4,5,6,7	HSPA8|0.3721	coated vesicle membrane	GO:0030662	5.58E-01	6.11E-01	1	7
4,5,6	CBX3|0.485775;GCH1|4.01735	nuclear envelope	GO:0005635	5.58E-01	6.11E-01	2	6
2	KRT1|0.4198;VTN|2.01125	extracellular matrix	GO:0031012	5.60E-01	6.12E-01	2	2
3,4	ATG7|2.120975;TUBG1|0.4102	cilium	GO:0005929	5.85E-01	6.37E-01	2	4
4,5	HSP90AB1|0.458775	apical plasma membrane	GO:0016324	6.02E-01	6.54E-01	1	5
4,5,6	TFRC|0.403125	plasma membrane receptor complex	GO:0098802	6.13E-01	6.64E-01	1	6
6	ASPH|2.59965	cation channel complex	GO:0034703	6.24E-01	6.74E-01	1	6
4	HSPA8|0.3721	ubiquitin ligase complex	GO:0000151	6.30E-01	6.79E-01	1	4
3	ANXA5|3.637725;TFRC|0.403125	side of membrane	GO:0098552	6.40E-01	6.88E-01	2	3
6,7,8	SQSTM1|6.007525	nuclear body	GO:0016604	6.50E-01	6.97E-01	1	8
3	VIM|2.241075;NSF|2.017425;NCDN|0.4114;MYH10|5.579275	neuron part	GO:0097458	6.63E-01	7.09E-01	4	3
6	HBA1|2.13735	endocytic vesicle	GO:0030139	6.70E-01	7.14E-01	1	6
4	NDE1|0.446425	microtubule associated complex	GO:0005875	6.74E-01	7.17E-01	1	4
5,6,7	NSF|2.017425	Golgi subcompartment	GO:0098791	7.04E-01	7.46E-01	1	7
2	NSF|2.017425;MYH10|5.579275	synapse part	GO:0044456	7.10E-01	7.51E-01	2	2
3,4	ASPH|2.59965;ERO1L|2.370575;RHOG|2.449775	endoplasmic reticulum membrane	GO:0005789	7.59E-01	8.00E-01	3	4
3	ASPH|2.59965;ERO1L|2.370575;RHOG|2.449775	nuclear outer membrane-endoplasmic reticulum membrane network	GO:0042175	7.69E-01	8.10E-01	3	3

4,5,6	HSPD1|2.88995;NSF|2.017425;LIG1|0.440425;ESD|2.155525;SLC2A1|5.081875	Golgi apparatus	GO:0005794	7.82E-01	8.20E-01	5	6
4,5	ASPH|2.59965	integral component of organelle membrane	GO:0031301	7.83E-01	8.20E-01	1	5
3,4	ASPH|2.59965	intrinsic component of organelle membrane	GO:0031300	7.91E-01	8.25E-01	1	4
3	VTN|2.01125	proteinaceous extracellular matrix	GO:0005578	7.92E-01	8.25E-01	1	3
5	ASPH|2.59965	ion channel	GO:0034702	8.01E-01	8.32E-01	1	5
4,5,6	HSPD1|2.88995;ASPH|2.59965;ERO1L|2.370575;SQSTM1|6.007525;RHOG|2.449775	endoplasmic reticulum	GO:0005783	8.09E-01	8.39E-01	5	6
2	NSF|2.017425;MYH10|5.579275	synapse	GO:0045202	8.20E-01	8.47E-01	2	2
4,5	ASPH|2.59965	transmembrane transporter	GO:1902495	8.27E-01	8.51E-01	1	5
4,5	TFRC|0.403125;VTN|2.01125	plasma membrane protein complex	GO:0098797	8.28E-01	8.51E-01	2	5
4	ASPH|2.59965	transporter	GO:1990351	8.30E-01	8.51E-01	1	4
4,5	ASPH|2.59965;ERO1L|2.370575;RHOG|2.449775	endoplasmic reticulum part	GO:0044432	8.38E-01	8.57E-01	3	5
4,5	NSF|2.017425;SLC2A1|5.081875	Golgi apparatus	GO:0044431	8.90E-01	9.07E-01	2	5
3	HSP90AB1|0.458775;LCP1|2.170475;ANXA5|3.637725;TFRC|0.403125;SLC2A1|5.081875;PLEK|2.071125;VTN|2.01125;ARHGDIA|3.8215	plasma membrane part	GO:0044459	8.91E-01	9.07E-01	8	3
5,6	SLC2A1|5.081875	Golgi membrane	GO:0000139	9.38E-01	9.53E-01	1	6
4,5	TFRC|0.403125;SLC2A1|5.081875	integral component of plasma membrane	GO:0005887	9.92E-01	1.00E+00	2	5
4	TFRC|0.403125;SLC2A1|5.081875	intrinsic component of plasma membrane	GO:0031226	9.94E-01	1.00E+00	2	4
2	HSP90AB1|0.458775;LCP1|2.170475;HSPD1|2.88995;ASPH|2.59965;ATP6V1B2|2.0575;VDAC3|2.015475;ANXA5|3.637725;SLC25A1|2.1121;ERO1L|2.370575;LAMTOR2|7.841025;TM9SF4|2.3766;TFRC|0.403125;RHOG|2.449775;FNDC3B|2.328575;EFHD2|2.281;SLC2A1|5.081875;VAT1|2.702925;PLEK|2.071125;VTN|2.01125;ARHGDIA|3.8215;MPEG1|5.8062;IFITM2|5.6831	membrane part	GO:0044425	9.96E-01	1.00E+00	23	2
4	HSPD1|2.88995;ASPH|2.59965;ATP6V1B2|2.0575;VDAC3|2.015475;SLC25A1|2.1121;TM9SF4|2.3766;TFRC|0.403125;FNDC3B|2.328575;SLC2A1|5.081875;VAT1|2.702925;MPEG1|5.8062;IFITM2|5.68315;PLP1|3.936	integral component of membrane	GO:0016021	1.00E+00	1.00E+00	13	4
3	HSPD1|2.88995;ASPH|2.59965;ATP6V1B2|2.0575;VDAC3|2.015475;SLC25A1|2.1121;TM9SF4|2.3766;TFRC|0.403125;FNDC3B|2.328575;SLC2A1|5.081875;VAT1|2.702925;MPEG1|5.8062;IFITM2|5.68315;PLP1|3.936	intrinsic component of membrane	GO:0031224	1.00E+00	1.00E+00	13	3

1	HSPA8|0.3721;ACTG1|2.1999;HSP90AB1|0.458775;LCP1|2.170475;ENO1|2.1939;HSPD1|2.88995;HIST1H2BN|0.481625;RPL3|3.30745;VIM|2.241075;CCT7|2.06135;PABPC1|0.3285;SARS|2.2366;ASPH|2.59965;KRT1|0.4198;YARS|2.288275;ATP6V1B2|2.0575;MCM3|0.35205;KRT9|0.268525;RPL4|2.063325;NSF|2.017425;GMPS|0.496875;MTHFD1L|6.667175;CKB|0.486625;NARS|2.162;MCM2|0.491625;HSPA4L|2.13105;SMC2|0.485025;DNMT1|0.172;ALDH1L2|2.148;HIST1H4A|0.23835;KPNA2|0.186675;VDAC3|2.015475;YWHAG|2.359475;ANXA5|3.637725;SRRT|0.39875;GSN|2.4555;CNDP2|2.0938;NCAPG|0.4528;CBX3|0.485775;GTF2E1|0.300475;AKR1B1|2.235325;PABPC4|0.26245;LIG1|0.440425;SLC25A1|2.1121;G3BP1|0.3904;ERO1L|2.370575;ANKRD28|3.3035;PRDX5|3.0143;BAZ1B|0.460625;ESD|2.155525;ISYNA1|0.49065;ALDOC|2.04725;PSME3|0.401575;RRM2|0.21675;EIF4H|0.275675;ACAT2|2.108325;LAMTOR2|7.841025;MCMBP|0.4769;RPL27|2.15885;SQSTM1|6.007525;ATG7|2.120975;PCNA|0.404975;TM9SF4|2.3766;MAPK14|0.483325;HIST1H3A|0.455625;BTF3|0.2449;TFRC|0.403125;RHOG|2.449775;TOP2A|0.481675;SNW1|0.473175;STAT3|0.4995;FNDC3B|2.328575;TUBG1|0.4102;EFHD2|2.281;NCDN|0.4114;SLC2A1|5.081875;TXNIP|2.0553;VAT1|2.702925;IPO8|0.443925;PLEK|2.071125;NDE1|0.446425;S100A11|2.531875;FAM192A|0.462425;HBA1|2.13735;NDRG1|10.0073;IK|0.480125;VTN|2.01125;RBBP7|0.218475;ARHGDIA|3.8215;PLBD2|2.666725;ACTB|2.1999;DNPEP|2.077575;HIST2H2AA3|0.29265;H3F3A|0.20985;MYH10|5.579275;TTK|0.488375;SOS2|11.48525;ASL|2.252975;ALB|0.23405;MPEG1|5.8062;CDC123|0.29925;PLK1|0.415475;GCH1|4.01735;SOD2|2.175425;PTP4A3|0.3433;TBCA|0.3515;CETN3|0.4171;IFITM2|5.68315;ZNF706|0.3974;CHAF1B|0.2723;PLP1|3.936	cellular_component	GO:0005575	1.00E+00	1.00E+00	111	1

Table S7 MF enrichment analysis of differentially expressed proteins

levels	Genes	GO_Name	GO_ID	Pvalue	Pvalue_adjusted	Count	maxLevel
3	HSPA8|0.3721;ACTG1|2.1999;HSP90AB1|0.458775;LCP1|2.170475;ENO1|2.1939;HSPD1|2.88995;HIST1H2BN|0.481625;VIM|2.241075;CCT7|2.06135;PABPC1|0.3285;ASPH|2.59965;KRT1|0.4198;YARS|2.288275;ATP6V1B2|2.0575;MCM3|0.35205;RPL4|2.063325;NSF|2.017425;MTHFD1L|6.667175;CKB|0.486625;MCM2|0.491625;SMC2|0.485025;DNMT1|0.172;HIST1H4A|0.23835;KPNA2|0.186675;YWHAG|2.359475;ANXA5|3.637725;SRRT|0.39875;GSN|2.4555;NCAPG|0.4528;CBX3|0.485775;GTF2E1|0.300475;PABPC4|0.26245;G3BP1|0.3904;ERO1L|2.370575;ANKRD28|3.3035;PRDX5|3.0143;BAZ1B|0.460625;ESD|2.155525;ISYNA1|0.49065;ALDOC|2.04725;PSME3|0.401575;RRM2|0.21675;EIF4H|0.275675;ACAT2|2.108325;LAMTOR2|7.841025;MCMBP|0.4769;SQSTM1|6.007525;ATG7|2.120975;PCNA|0.404975;MAPK14|0.483325;HIST1H3A|0.455625;BTF3|0.2449;TFRC|0.403125;RHOG|2.449775;TOP2A|0.481675;SNW1|0.473175;STAT3|0.4995;FNDC3B|2.328575;TUBG1|0.4102;NCDN|0.4114;SLC2A1|5.081875;TXNIP|2.0553;IPO8|0.443925;PLEK|2.071125;NDE1|0.446425;S100A11|2.531875;FAM192A|0.462425;HBA1|2.13735;NDRG1|10.0073;IK|0.480125;VTN|2.01125;RBBP7|0.218475;ARHGDIA|3.8215;ACTB|2.1999;DNPEP|2.077575;HIST2H2AA3|0.29265;H3F3A|0.20985;MYH10|5.579275;TTK|0.488375;SOS2|11.48525;ASL|2.252975;ALB|0.23405;PLK1|0.415475;GCH1|4.01735;SOD2|2.175425;TBCA|0.3515	protein binding	GO:0005515	3.56E-24	1.84E-21	89	3
6	HSPA8|0.3721;HSP90AB1|0.458775;ENO1|2.1939;HSPD1|2.88995;RPL3|3.30745;PABPC1|0.3285;YARS|2.288275;RPL4|2.063325;HIST1H4A|0.23835;KPNA2|0.186675;YWHAG|2.359475;SRRT|0.39875;PABPC4|0.26245;G3BP1|0.3904;EIF4H|0.275675;RPL27|2.15885;BTF3|0.2449;TFRC|0.403125;TOP2A|0.481675;SNW1|0.473175;FNDC3B|2.328575;TBCA|0.3515	poly(A) RNA binding	GO:0044822	5.72E-12	1.47E-09	22	6
4	HSPA8|0.3721;ACTG1|2.1999;HSP90AB1|0.458775;LCP1|2.170475;ENO1|2.1939;HSPD1|2.88995;NSF|2.017425;CKB|0.486625;KPNA2|0.186675;YWHAG|2.359475;ANXA5|3.637725;CBX3|0.485775;SQSTM1|6.007525;PCNA|0.404975;MAPK14|0.483325;TOP2A|0.481675;STAT3|0.4995;SLC2A1|5.081875;TXNIP|2.0553;IPO8|0.443925;PLEK|2.071125;NDRG1|10.0073;ARHGDIA|3.8215;ACTB|2.1999;PLK1|0.415475	enzyme binding	GO:0019899	3.07E-10	5.26E-08	25	4
4	ACTG1|2.1999;LCP1|2.170475;VIM|2.241075;CCT7|2.06135;MTHFD1L|6.667175;CBX3|0.485775;PSME3|0.401575;SQSTM1|6.007525;ATG7|2.120975;PCNA|0.404975;TFRC|0.403125;TOP2A|0.481675;SLC2A1|5.081875;PLEK|2.071125;NDE1|0.446425;S100A11|2.531875;IK|0.480125;ACTB|2.1999;GCH1|4.01735;SOD2|2.175425	identical protein binding	GO:0042802	5.34E-10	6.87E-08	20	4

2	HSPA8|0.3721;ACTG1|2.1999;HSP90AB1|0.458775;LCP1|2.170475;ENO1|2.1939;HSPD1|2.88995;HIST1H2BN|0.481625;RPL3|3.30745;VIM|2.241075;CCT7|2.06135;PABPC1|0.3285;SARS|2.2366;ASPH|2.59965;KRT1|0.4198;YARS|2.288275;ATP6V1B2|2.0575;MCM3|0.35205;RPL4|2.063325;NSF|2.017425;GMPS|0.496875;MTHFD1L|6.667175;CKB|0.486625;NARS|2.162;MCM2|0.491625;HSPA4L|2.13105;SMC2|0.485025;DNMT1|0.172;HIST1H4A|0.23835;KPNA2|0.186675;VDAC3|2.015475;YWHAG|2.359475;ANXA5|3.637725;SRRT|0.39875;GSN|2.4555;CNDP2|2.0938;NCAPG|0.4528;CBX3|0.485775;GTF2E1|0.300475;PABPC4|0.26245;LIG1|0.440425;G3BP1|0.3904;ERO1L|2.370575;ANKRD28|3.3035;PRDX5|3.0143;BAZ1B|0.460625;ESD|2.155525;ISYNA1|0.49065;ALDOC|2.04725;PSME3|0.401575;RRM2|0.21675;EIF4H|0.275675;ACAT2|2.108325;LAMTOR2|7.841025;MCMBP|0.4769;RPL27|2.15885;SQSTM1|6.007525;ATG7|2.120975;PCNA|0.404975;MAPK14|0.483325;HIST1H3A|0.455625;BTF3|0.2449;TFRC|0.403125;RHOG|2.449775;TOP2A|0.481675;SNW1|0.473175;STAT3|0.4995;FNDC3B|2.328575;TUBG1|0.4102;EFHD2|2.281;NCDN|0.4114;SLC2A1|5.081875;TXNIP|2.0553;VAT1|2.702925;IPO8|0.443925;PLEK|2.071125;NDE1|0.446425;S100A11|2.531875;FAM192A|0.462425;HBA1|2.13735;NDRG1|10.0073;IK|0.480125;VTN|2.01125;RBBP7|0.218475;ARHGDIA|3.8215;ACTB|2.1999;DNPEP|2.077575;HIST2H2AA3|0.29265;H3F3A|0.20985;MYH10|5.579275;TTK|0.488375;SOS2|11.48525;ASL|2.252975;ALB|0.23405;PLK1|0.415475;GCH1|4.01735;SOD2|2.175425;TBCA|0.3515;CETN3|0.4171;ZN	binding	GO:0005488	7.82E-10	7.41E-08	101	2
5	HSPA8|0.3721;HSP90AB1|0.458775;ENO1|2.1939;HSPD1|2.88995;RPL3|3.30745;VIM|2.241075;PABPC1|0.3285;SARS|2.2366;YARS|2.288275;RPL4|2.063325;DNMT1|0.172;HIST1H4A|0.23835;KPNA2|0.186675;YWHAG|2.359475;SRRT|0.39875;PABPC4|0.26245;G3BP1|0.3904;EIF4H|0.275675;RPL27|2.15885;BTF3|0.2449;TFRC|0.403125;TOP2A|0.481675;SNW1|0.473175;FNDC3B|2.328575;RBBP7|0.218475;TBCA|0.3515	RNA binding	GO:0003723	8.63E-10	7.41E-08	26	5
4	HSPD1|2.88995;HIST1H2BN|0.481625;MTHFD1L|6.667175;SMC2|0.485025;HIST1H4A|0.23835;PRDX5|3.0143;SQSTM1|6.007525;ATG7|2.120975;HIST1H3A|0.455625;TFRC|0.403125;TOP2A|0.481675;STAT3|0.4995;PLEK|2.071125;S100A11|2.531875;HIST2H2AA3|0.29265;H3F3A|0.20985;SOS2|11.48525;GCH1|4.01735	protein dimerization activity	GO:0046983	1.05E-07	7.71E-06	18	4
5	HSP90AB1|0.458775;NSF|2.017425;YWHAG|2.359475;ANXA5|3.637725;SQSTM1|6.007525;PCNA|0.404975;TOP2A|0.481675;STAT3|0.4995;SLC2A1|5.081875;PLEK|2.071125;ARHGDIA|3.8215;PLK1|0.415475	kinase binding	GO:0019900	4.04E-07	2.60E-05	12	5
6	HSP90AB1|0.458775;NSF|2.017425;YWHAG|2.359475;ANXA5|3.637725;SQSTM1|6.007525;PCNA|0.404975;TOP2A|0.481675;STAT3|0.4995;PLEK|2.071125;ARHGDIA|3.8215;PLK1|0.415475	protein kinase binding	GO:0019901	1.06E-06	6.05E-05	11	6
3	HSPA8|0.3721;HSP90AB1|0.458775;LCP1|2.170475;HSPD1|2.88995;VIM|2.241075;NSF|2.017425;DNMT1|0.172;BAZ1B|0.460625;MCMBP|0.4769;PCNA|0.404975;TOP2A|0.481675;NDE1|0.446425;NDRG1|10.0073;VTN|2.01125;ACTB|2.1999;H3F3A|0.20985;MYH10|5.579275;PLK1|0.415475;CHAF1B|0.2723	macromolecular complex binding	GO:0044877	3.08E-06	1.47E-04	19	3
6	HSPA8|0.3721;ACTG1|2.1999;HSP90AB1|0.458775;HSPD1|2.88995;CCT7|2.06135;SARS|2.2366;YARS|2.288275;ATP6V1B2|2.0575;MCM3|0.35205;NSF|2.017425;GMPS|0.496875;MTHFD1L|6.667175;CKB|0.486625;NARS|2.162;MCM2|0.491625;HSPA4L|2.13105;SMC2|0.485025;LIG1|0.440425;G3BP1|0.3904;BAZ1B|0.460625;MAPK14|0.483325;TOP2A|0.481675;ACTB|2.1999;MYH10|5.579275;TTK|0.488375;PLK1|0.415475	ATP binding	GO:0005524	3.23E-06	1.47E-04	26	6

<table>
<tr><td>3</td><td>HSPA8|0.3721;ACTG1|2.1999;HSP90AB1|0.458775;ENO1|2.1939;HSPD1|2.88995;HIST1H2BN|0.481625;RPL3|3.30745;VIM|2.241075;CCT7|2.06135;PABPC1|0.3285;SARS|2.2366;YARS|2.288275;ATP6V1B2|2.0575;MCM3|0.35205;RPL4|2.063325;NSF|2.017425;GMPS|0.496875;MTHFD1L|6.667175;CKB|0.486625;NARS|2.162;MCM2|0.491625;HSPA4L|2.13105;SMC2|0.485025;DNMT1|0.172;HIST1H4A|0.23835;KPNA2|0.186675;VDAC3|2.015475;YWHAG|2.359475;SRRT|0.39875;GTF2E1|0.300475;PABPC4|0.26245;LIG1|0.440425;G3BP1|0.3904;PRDX5|3.0143;BAZ1B|0.460625;EIF4H|0.275675;RPL27|2.15885;PCNA|0.404975;MAPK14|0.483325;HIST1H3A|0.455625;BTF3|0.2449;TFRC|0.403125;RHOG|2.449775;TOP2A|0.481675;SNW1|0.473175;STAT3|0.4995;FNDC3B|2.328575;TUBG1|0.4102;HBA1|2.13735;RBBP7|0.218475;ACTB|2.1999;HIST2H2AA3|0.29265;H3F3A|0.20985;MYH10|5.579275;TTK|0.488375;SOS2|11.48525;ALB|0.23405;PLK1|0.415475;GCH1|4.01735;TBCA|0.3515</td><td>heterocyclic compound binding</td><td>GO:1901363</td><td>3.44E-06</td><td>1.47E-04</td><td>60</td><td>3</td></tr>
<tr><td>6,7</td><td>HSPA8|0.3721;ACTG1|2.1999;HSP90AB1|0.458775;HSPD1|2.88995;CCT7|2.06135;SARS|2.2366;YARS|2.288275;ATP6V1B2|2.0575;MCM3|0.35205;NSF|2.017425;GMPS|0.496875;MTHFD1L|6.667175;CKB|0.486625;NARS|2.162;MCM2|0.491625;HSPA4L|2.13105;SMC2|0.485025;LIG1|0.440425;G3BP1|0.3904;BAZ1B|0.460625;MAPK14|0.483325;TOP2A|0.481675;ACTB|2.1999;MYH10|5.579275;TTK|0.488375;PLK1|0.415475</td><td>adenyl ribonucleotide binding</td><td>GO:0032559</td><td>3.93E-06</td><td>1.48E-04</td><td>26</td><td>7</td></tr>
<tr><td>6</td><td>HSPA8|0.3721;ACTG1|2.1999;HSP90AB1|0.458775;HSPD1|2.88995;CCT7|2.06135;SARS|2.2366;YARS|2.288275;ATP6V1B2|2.0575;MCM3|0.35205;NSF|2.017425;GMPS|0.496875;MTHFD1L|6.667175;CKB|0.486625;NARS|2.162;MCM2|0.491625;HSPA4L|2.13105;SMC2|0.485025;LIG1|0.440425;G3BP1|0.3904;BAZ1B|0.460625;MAPK14|0.483325;TOP2A|0.481675;ACTB|2.1999;MYH10|5.579275;TTK|0.488375;PLK1|0.415475</td><td>adenyl nucleotide binding</td><td>GO:0030554</td><td>4.11E-06</td><td>1.48E-04</td><td>26</td><td>6</td></tr>
<tr><td>3</td><td>HSPA8|0.3721;ACTG1|2.1999;HSP90AB1|0.458775;HSPD1|2.88995;VIM|2.241075;CCT7|2.06135;SARS|2.2366;YARS|2.288275;ATP6V1B2|2.0575;MCM3|0.35205;NSF|2.017425;GMPS|0.496875;MTHFD1L|6.667175;CKB|0.486625;NARS|2.162;MCM2|0.491625;HSPA4L|2.13105;SMC2|0.485025;LIG1|0.440425;G3BP1|0.3904;BAZ1B|0.460625;MAPK14|0.483325;TFRC|0.403125;RHOG|2.449775;TOP2A|0.481675;TUBG1|0.4102;VTN|2.01125;ACTB|2.1999;MYH10|5.579275;TTK|0.488375;PLK1|0.415475;GCH1|4.01735</td><td>carbohydrate derivative binding</td><td>GO:0097367</td><td>4.35E-06</td><td>1.48E-04</td><td>32</td><td>3</td></tr>
<tr><td>3</td><td>HSPA8|0.3721;ACTG1|2.1999;HSP90AB1|0.458775;ENO1|2.1939;HSPD1|2.88995;HIST1H2BN|0.481625;RPL3|3.30745;VIM|2.241075;CCT7|2.06135;PABPC1|0.3285;SARS|2.2366;YARS|2.288275;ATP6V1B2|2.0575;MCM3|0.35205;RPL4|2.063325;NSF|2.017425;GMPS|0.496875;MTHFD1L|6.667175;CKB|0.486625;NARS|2.162;MCM2|0.491625;HSPA4L|2.13105;SMC2|0.485025;DNMT1|0.172;HIST1H4A|0.23835;KPNA2|0.186675;VDAC3|2.015475;YWHAG|2.359475;SRRT|0.39875;GTF2E1|0.300475;PABPC4|0.26245;LIG1|0.440425;G3BP1|0.3904;PRDX5|3.0143;BAZ1B|0.460625;EIF4H|0.275675;RPL27|2.15885;PCNA|0.404975;MAPK14|0.483325;HIST1H3A|0.455625;BTF3|0.2449;TFRC|0.403125;RHOG|2.449775;TOP2A|0.481675;SNW1|0.473175;STAT3|0.4995;FNDC3B|2.328575;TUBG1|0.4102;HBA1|2.13735;RBBP7|0.218475;ACTB|2.1999;HIST2H2AA3|0.29265;H3F3A|0.20985;MYH10|5.579275;TTK|0.488375;SOS2|11.48525;ALB|0.23405;PLK1|0.415475;GCH1|4.01735;TBCA|0.3515</td><td>organic cyclic compound binding</td><td>GO:0097159</td><td>4.59E-06</td><td>1.48E-04</td><td>60</td><td>3</td></tr>
<tr><td>3</td><td>HSPA8|0.3721;ACTG1|2.1999;HSP90AB1|0.458775;HSPD1|2.88995;CCT7|2.06135;PABPC1|0.3285;SARS|2.2366;YARS|2.288275;ATP6V1B2|2.0575;MCM3|0.35205;NSF|2.017425;GMPS|0.496875;MTHFD1L|6.667175;CKB|0.486625;NARS|2.162;MCM2|0.491625;HSPA4L|2.13105;SMC2|0.485025;DNMT1|0.172;VDAC3|2.015475;PABPC4|0.26245;LIG1|0.440425;G3BP1|0.3904;BAZ1B|0.460625;EIF4H|0.275675;MAPK14|0.483325;RHOG|2.449775;TOP2A|0.481675;TUBG1|0.4102;ARHGDIA|3.8215;ACTB|2.1999;MYH10|5.579275;TTK|0.488375;ALB|0.23405;PLK1|0.415475;GCH1|4.01735</td><td>small molecule binding</td><td>GO:0036094</td><td>7.41E-06</td><td>2.24E-04</td><td>36</td><td>3</td></tr>
</table>

4	MCM2|0.491625;HIST1H4A|0.23835;BAZ1B|0.460625;HIST1H3A|0.455625;H3F3A|0.20985;CHAF1B|0.2723	histone binding	GO:0042393	8.93E-06	2.36E-04	6	4
5	HSPA8|0.3721;ACTG1|2.1999;HSP90AB1|0.458775;HSPD1|2.88995;CCT7|2.06135;SARS|2.2366;YARS|2.288275;ATP6V1B2|2.0575;MCM3|0.35205;NSF|2.017425;GMPS|0.496875;MTHFD1L|6.667175;CKB|0.486625;NARS|2.162;MCM2|0.491625;HSPA4L|2.13105;SMC2|0.485025;LIG1|0.440425;G3BP1|0.3904;BAZ1B|0.460625;MAPK14|0.483325;RHOG|2.449775;TOP2A|0.481675;TUBG1|0.4102;ACTB|2.1999;MYH10|5.579275;TTK|0.488375;PLK1|0.415475;GCH1|4.01735	purine ribonucleoside triphosphate binding	GO:0035639	9.40E-06	2.36E-04	29	5
6	HSPA8|0.3721;ACTG1|2.1999;HSP90AB1|0.458775;HSPD1|2.88995;CCT7|2.06135;SARS|2.2366;YARS|2.288275;ATP6V1B2|2.0575;MCM3|0.35205;NSF|2.017425;GMPS|0.496875;MTHFD1L|6.667175;CKB|0.486625;NARS|2.162;MCM2|0.491625;HSPA4L|2.13105;SMC2|0.485025;LIG1|0.440425;G3BP1|0.3904;BAZ1B|0.460625;MAPK14|0.483325;RHOG|2.449775;TOP2A|0.481675;TUBG1|0.4102;ACTB|2.1999;MYH10|5.579275;TTK|0.488375;PLK1|0.415475;GCH1|4.01735	purine ribonucleoside binding	GO:0032550	9.86E-06	2.36E-04	29	6
5	HSPA8|0.3721;ACTG1|2.1999;HSP90AB1|0.458775;HSPD1|2.88995;CCT7|2.06135;SARS|2.2366;YARS|2.288275;ATP6V1B2|2.0575;MCM3|0.35205;NSF|2.017425;GMPS|0.496875;MTHFD1L|6.667175;CKB|0.486625;NARS|2.162;MCM2|0.491625;HSPA4L|2.13105;SMC2|0.485025;LIG1|0.440425;G3BP1|0.3904;BAZ1B|0.460625;MAPK14|0.483325;RHOG|2.449775;TOP2A|0.481675;TUBG1|0.4102;ACTB|2.1999;MYH10|5.579275;TTK|0.488375;PLK1|0.415475;GCH1|4.01735	purine nucleoside binding	GO:0001883	9.99E-06	2.36E-04	29	5
5	HSPA8|0.3721;ACTG1|2.1999;HSP90AB1|0.458775;HSPD1|2.88995;CCT7|2.06135;SARS|2.2366;YARS|2.288275;ATP6V1B2|2.0575;MCM3|0.35205;NSF|2.017425;GMPS|0.496875;MTHFD1L|6.667175;CKB|0.486625;NARS|2.162;MCM2|0.491625;HSPA4L|2.13105;SMC2|0.485025;LIG1|0.440425;G3BP1|0.3904;BAZ1B|0.460625;MAPK14|0.483325;RHOG|2.449775;TOP2A|0.481675;TUBG1|0.4102;ACTB|2.1999;MYH10|5.579275;TTK|0.488375;PLK1|0.415475;GCH1|4.01735	ribonucleoside binding	GO:0032549	1.07E-05	2.36E-04	29	5
4	HSPA8|0.3721;ACTG1|2.1999;HSP90AB1|0.458775;HSPD1|2.88995;CCT7|2.06135;SARS|2.2366;YARS|2.288275;ATP6V1B2|2.0575;MCM3|0.35205;NSF|2.017425;GMPS|0.496875;MTHFD1L|6.667175;CKB|0.486625;NARS|2.162;MCM2|0.491625;HSPA4L|2.13105;SMC2|0.485025;LIG1|0.440425;G3BP1|0.3904;BAZ1B|0.460625;MAPK14|0.483325;RHOG|2.449775;TOP2A|0.481675;TUBG1|0.4102;ACTB|2.1999;MYH10|5.579275;TTK|0.488375;PLK1|0.415475;GCH1|4.01735	nucleoside binding	GO:0001882	1.13E-05	2.36E-04	29	4
5,6	HSPA8|0.3721;ACTG1|2.1999;HSP90AB1|0.458775;HSPD1|2.88995;CCT7|2.06135;SARS|2.2366;YARS|2.288275;ATP6V1B2|2.0575;MCM3|0.35205;NSF|2.017425;GMPS|0.496875;MTHFD1L|6.667175;CKB|0.486625;NARS|2.162;MCM2|0.491625;HSPA4L|2.13105;SMC2|0.485025;LIG1|0.440425;G3BP1|0.3904;BAZ1B|0.460625;MAPK14|0.483325;RHOG|2.449775;TOP2A|0.481675;TUBG1|0.4102;ACTB|2.1999;MYH10|5.579275;TTK|0.488375;PLK1|0.415475;GCH1|4.01735	purine ribonucleotide binding	GO:0032555	1.15E-05	2.36E-04	29	6
4	HSPA8|0.3721;HSP90AB1|0.458775;HSPD1|2.88995;CCT7|2.06135;TBCA|0.3515;CHAF1B|0.2723	unfolded protein binding	GO:0051082	1.24E-05	2.36E-04	6	4
4	HSPA8|0.3721;ACTG1|2.1999;HSP90AB1|0.458775;HSPD1|2.88995;CCT7|2.06135;PABPC1|0.3285;SARS|2.2366;YARS|2.288275;ATP6V1B2|2.0575;MCM3|0.35205;NSF|2.017425;GMPS|0.496875;MTHFD1L|6.667175;CKB|0.486625;NARS|2.162;MCM2|0.491625;HSPA4L|2.13105;SMC2|0.485025;DNMT1|0.172;VDAC3|2.015475;PABPC4|0.26245;LIG1|0.440425;G3BP1|0.3904;BAZ1B|0.460625;EIF4H|0.275675;MAPK14|0.483325;RHOG|2.449775;TOP2A|0.481675;TUBG1|0.4102;ACTB|2.1999;MYH10|5.579275;TTK|0.488375;PLK1|0.415475;GCH1|4.01735	nucleotide binding	GO:0000166	1.25E-05	2.36E-04	34	4

4	HSPA8|0.3721;ACTG1|2.1999;HSP90AB1|0.458775;HSPD1|2.88995;CCT7|2.06135;PABPC1|0.3285;SARS|2.2366;YARS|2.288275;ATP6V1B2|2.0575;MCM3|0.35205;NSF|2.017425;GMPS|0.496875;MTHFD1L|6.667175;CKB|0.486625;NARS|2.162;MCM2|0.491625;HSPA4L|2.13105;SMC2|0.485025;DNMT1|0.172;VDAC3|2.015475;PABPC4|0.26245;LIG1|0.440425;G3BP1|0.3904;BAZ1B|0.460625;EIF4H|0.275675;MAPK14|0.483325;RHOG|2.449775;TOP2A|0.481675;TUBG1|0.4102;ACTB|2.1999;MYH10|5.579275;TTK|0.488375;PLK1|0.415475;GCH1|4.01735	nucleoside phosphate binding	GO:1901265	1.25E-05	2.36E-04	34	4
5	HSPA8|0.3721;ACTG1|2.1999;HSP90AB1|0.458775;HSPD1|2.88995;CCT7|2.06135;SARS|2.2366;YARS|2.288275;ATP6V1B2|2.0575;MCM3|0.35205;NSF|2.017425;GMPS|0.496875;MTHFD1L|6.667175;CKB|0.486625;NARS|2.162;MCM2|0.491625;HSPA4L|2.13105;SMC2|0.485025;LIG1|0.440425;G3BP1|0.3904;BAZ1B|0.460625;MAPK14|0.483325;RHOG|2.449775;TOP2A|0.481675;TUBG1|0.4102;ACTB|2.1999;MYH10|5.579275;TTK|0.488375;PLK1|0.415475;GCH1|4.01735	purine nucleotide binding	GO:0017076	1.29E-05	2.36E-04	29	5
4	HSPA8|0.3721;ACTG1|2.1999;HSP90AB1|0.458775;HSPD1|2.88995;CCT7|2.06135;SARS|2.2366;YARS|2.288275;ATP6V1B2|2.0575;MCM3|0.35205;NSF|2.017425;GMPS|0.496875;MTHFD1L|6.667175;CKB|0.486625;NARS|2.162;MCM2|0.491625;HSPA4L|2.13105;SMC2|0.485025;LIG1|0.440425;G3BP1|0.3904;BAZ1B|0.460625;MAPK14|0.483325;RHOG|2.449775;TOP2A|0.481675;TUBG1|0.4102;ACTB|2.1999;MYH10|5.579275;TTK|0.488375;PLK1|0.415475;GCH1|4.01735	ribonucleotide binding	GO:0032553	1.36E-05	2.41E-04	29	4
7	YWHAG|2.359475;SQSTM1|6.007525;TOP2A|0.481675;PLEK|2.071125	protein kinase C binding	GO:0005080	2.01E-05	3.46E-04	4	7
4	HSPA8|0.3721;ACTG1|2.1999;HSP90AB1|0.458775;HSPD1|2.88995;CCT7|2.06135;SARS|2.2366;YARS|2.288275;ATP6V1B2|2.0575;MCM3|0.35205;NSF|2.017425;GMPS|0.496875;MTHFD1L|6.667175;CKB|0.486625;NARS|2.162;MCM2|0.491625;HSPA4L|2.13105;SMC2|0.485025;ANXA5|3.637725;LIG1|0.440425;G3BP1|0.3904;BAZ1B|0.460625;MAPK14|0.483325;RHOG|2.449775;TOP2A|0.481675;TUBG1|0.4102;PLEK|2.071125;VTN|2.01125;ARHGDIA|3.8215;ACTB|2.1999;MYH10|5.579275;TTK|0.488375;ALB|0.23405;PLK1|0.415475;GCH1|4.01735	anion binding	GO:0043168	2.32E-05	3.85E-04	34	4
4	HSPA8|0.3721;HSP90AB1|0.458775;LCP1|2.170475;HSPD1|2.88995;VIM|2.241075;NSF|2.017425;PCNA|0.404975;NDE1|0.446425;NDRG1|10.0073;VTN|2.01125;ACTB|2.1999;H3F3A|0.20985;MYH10|5.579275;PLK1|0.415475	protein complex binding	GO:0032403	3.16E-05	5.08E-04	14	4
5	HSPD1|2.88995;HIST1H2BN|0.481625;SMC2|0.485025;HIST1H4A|0.23835;HIST1H3A|0.455625;TOP2A|0.481675;HIST2H2AA3|0.29265;H3F3A|0.20985;SOS2|11.48525	protein heterodimerization activity	GO:0046982	3.27E-05	5.10E-04	9	5
4	HSPA8|0.3721;HSP90AB1|0.458775;ENO1|2.1939;HSPD1|2.88995;HIST1H2BN|0.481625;RPL3|3.30745;VIM|2.241075;PABPC1|0.3285;SARS|2.2366;YARS|2.288275;MCM3|0.35205;RPL4|2.063325;NARS|2.162;MCM2|0.491625;DNMT1|0.172;HIST1H4A|0.23835;KPNA2|0.186675;YWHAG|2.359475;SRRT|0.39875;GTF2E1|0.300475;PABPC4|0.26245;LIG1|0.440425;G3BP1|0.3904;PRDX5|3.0143;EIF4H|0.275675;RPL27|2.15885;PCNA|0.404975;HIST1H3A|0.455625;BTF3|0.2449;TFRC|0.403125;TOP2A|0.481675;SNW1|0.473175;STAT3|0.4995;FNDC3B|2.328575;RBBP7|0.218475;ACTB|2.1999;HIST2H2AA3|0.29265;H3F3A|0.20985;SOS2|11.48525;ALB|0.23405;TBCA|0.3515	nucleic acid binding	GO:0003676	5.91E-05	8.95E-04	41	4
3	ACTG1|2.1999;VIM|2.241075;KRT9|0.268525;TUBG1|0.4102;ACTB|2.1999	structural constituent of	GO:0005200	9.71E-05	1.42E-03	5	3
6	HSP90AB1|0.458775;HSPD1|2.88995;VIM|2.241075;TFRC|0.403125	double-stranded RNA binding	GO:0003725	9.95E-05	1.42E-03	4	6
4	HSPD1|2.88995;TFRC|0.403125;ALB|0.23405;TBCA|0.3515	chaperone binding	GO:0051087	2.35E-04	3.28E-03	4	4
4	DNMT1|0.172;BAZ1B|0.460625;MCMBP|0.4769;PCNA|0.404975;TOP2A|0.481675;ACTB|2.1999;H3F3A|0.20985;CHAF1B|0.2723	chromatin binding	GO:0003682	3.16E-04	4.28E-03	8	4
2	ACTG1|2.1999;RPL3|3.30745;VIM|2.241075;ASPH|2.59965;KRT1|0.4198;KRT9|0.268525;RPL4|2.063325;LAMTOR2|7.841025;RPL27|2.15885;TUBG1|0.4102;ACTB|2.1999;PLP1|3.936	structural molecule activity	GO:0005198	4.15E-04	5.49E-03	12	2

8	PABPC1|0.3285;PABPC4|0.26245	poly(U) RNA binding	GO:0008266	6.10E-04	7.86E-03	2	8
4	HSPA8|0.3721;HSP90AB1|0.458775;NSF|2.017425;YWHAG|2.359475;GSN|2.4555;CBX3|0.485775;SQSTM1|6.007525;NDE1|0.446425	protein domain specific binding	GO:0019904	7.20E-04	8.92E-03	8	4
7	ANXA5|3.637725;SQSTM1|6.007525;PCNA|0.404975	protein tyrosine kinase binding	GO:1990782	7.45E-04	8.92E-03	3	7
5	ANXA5|3.637725;SQSTM1|6.007525;PCNA|0.404975	receptor tyrosine kinase binding	GO:0030971	7.45E-04	8.92E-03	3	5
7	PABPC1|0.3285;PABPC4|0.26245	poly-pyrimidine tract binding	GO:0008187	8.18E-04	9.58E-03	2	7
2	PRDX5|3.0143;HBA1|2.13735;ALB|0.23405;SOD2|2.175425	antioxidant activity	GO:0016209	8.40E-04	9.61E-03	4	2
7	HSPD1|2.88995;MCM2|0.491625	DNA replication origin binding	GO:0003688	9.34E-04	1.05E-02	2	7
5,6	HSPA8|0.3721;HSP90AB1|0.458775	MHC class II protein complex	GO:0023026	1.06E-03	1.16E-02	2	6
4,5	HSPA8|0.3721;HSP90AB1|0.458775	MHC protein complex binding	GO:0023023	1.47E-03	1.57E-02	2	5
3	HSPA8|0.3721;ACTG1|2.1999;HSP90AB1|0.458775;LCP1|2.170475;ENO1|2.1939;HSPD1|2.88995;CCT7|2.06135;SARS|2.2366;ASPH|2.59965;YARS|2.288275;ATP6V1B2|2.0575;MCM3|0.35205;NSF|2.017425;GMPS|0.496875;MTHFD1L|6.667175;CKB|0.486625;NARS|2.162;MCM2|0.491625;HSPA4L|2.13105;SMC2|0.485025;DNMT1|0.172;ANXA5|3.637725;GSN|2.4555;CNDP2|2.0938;GTF2E1|0.300475;LIG1|0.440425;G3BP1|0.3904;BAZ1B|0.460625;RRM2|0.21675;SQSTM1|6.007525;MAPK14|0.483325;RHOG|2.449775;TOP2A|0.481675;TUBG1|0.4102;EFHD2|2.281;VAT1|2.702925;PLEK|2.071125;S100A11|2.531875;HBA1|2.13735;VTN|2.01125;ARHGDIA|3.8215;ACTB|2.1999;DNPEP|2.077575;MYH10|5.579275;TTK|0.488375;ALB|0.23405;PLK1|0.415475;GCH1|4.01735;SOD2|2.175425;CETN3|0.4171;ZNF706|0.3974	ion binding	GO:0043167	1.55E-03	1.61E-02	51	3
4	HSPA8|0.3721;TFRC|0.403125;ARHGDIA|3.8215	heat shock protein binding	GO:0031072	1.56E-03	1.61E-02	3	4
5	LCP1|2.170475;ENO1|2.1939;NSF|2.017425;IPO8|0.443925;NDRG1|10.0073;ARHGDIA|3.8215	GTPase binding	GO:0051020	1.60E-03	1.62E-02	6	5
5	MTHFD1L|6.667175;SQSTM1|6.007525;ATG7|2.120975;TFRC|0.403125;TOP2A|0.481675;PLEK|2.071125;S100A11|2.531875;GCH1|4.01735	protein homodimerization activity	GO:0042803	1.91E-03	1.86E-02	8	5
4	VIM|2.241075;PABPC1|0.3285;MAPK14|0.483325;TOP2A|0.481675	protein C-terminus binding	GO:0008022	1.92E-03	1.86E-02	4	4
6	MTHFD1L|6.667175;GCH1|4.01735	cyclohydrolase activity	GO:0019238	2.12E-03	2.02E-02	2	6
8	PABPC1|0.3285;PABPC4|0.26245	poly(A) binding	GO:0008143	2.68E-03	2.18E-02	2	8
5	BAZ1B|0.460625	vitamin D receptor activator activity	GO:0071884	2.84E-03	2.18E-02	1	5
8	GCH1|4.01735	GTP cyclohydrolase I activity	GO:0003934	2.84E-03	2.18E-02	1	8
4	PRDX5|3.0143	peroxynitrite reductase activity	GO:0072541	2.84E-03	2.18E-02	1	4
6	GMPS|0.496875	GMP synthase (glutamine-hydrolyzing) activity	GO:0003922	2.84E-03	2.18E-02	1	6
8	PCNA|0.404975	purine-specific mismatch base pair DNA N-glycosylase activity	GO:0000701	2.84E-03	2.18E-02	1	8
5	AKR1B1|2.235325	glyceraldehyde oxidoreductase activity	GO:0043795	2.84E-03	2.18E-02	1	5
8	PABPC4|0.26245	poly(C) RNA binding	GO:0017130	2.84E-03	2.18E-02	1	8
7,8,10,12	TFRC|0.403125	transferrin transmembrane transporter activity	GO:0033570	2.84E-03	2.18E-02	1	12
5	HSP90AB1|0.458775	CTP binding	GO:0002135	2.84E-03	2.18E-02	1	5
7	GCH1|4.01735	GTP cyclohydrolase activity	GO:0003933	2.84E-03	2.18E-02	1	7
5	GMPS|0.496875	GMP synthase activity	GO:0003921	2.84E-03	2.18E-02	1	5
6	ASPH|2.59965	peptide-aspartate beta-dioxygenase activity	GO:0004597	2.84E-03	2.18E-02	1	6
6	HSPA8|0.3721;ACTG1|2.1999;HSPD1|2.88995;CKB|0.486625;TXNIP|2.0553	ubiquitin protein ligase binding	GO:0031625	2.88E-03	2.18E-02	5	6

9	MCM3|0.35205;MCM2|0.491625;G3BP1|0.3904	DNA helicase activity	GO:0003678	2.95E-03	2.20E-02	3	9
5	HSPA8|0.3721;ACTG1|2.1999;HSPD1|2.88995;CKB|0.486625;TXNIP|2.0553	ubiquitin-like protein ligase	GO:0044389	3.02E-03	2.22E-02	5	5
7	PABPC1|0.3285;PABPC4|0.26245	poly-purine tract binding	GO:0070717	3.30E-03	2.36E-02	2	7
4,7	ARHGDIA|3.8215;ALB|0.23405	fatty acid binding	GO:0005504	3.30E-03	2.36E-02	2	7
5	ENO1|2.1939;HSPD1|2.88995;HIST1H2BN|0.481625;MCM3|0.35205;MCM2|0.491625;DNMT1|0.172;HIST1H4A|0.23835;SRRT|0.39875;GTF2E1|0.300475;LIG1|0.440425;G3BP1|0.3904;PRDX5|3.0143;PCNA|0.404975;HIST1H3A|0.455625;TOP2A|0.481675;STAT3|0.4995;ACTB|2.1999;HIST2H2AA3|0.29265;H3F3A|0.20985;SOS2|11.48525;ALB|0.23405	DNA binding	GO:0003677	4.19E-03	2.95E-02	21	5
5	HSP90AB1|0.458775	pyrimidine nucleoside binding	GO:0001884	5.67E-03	3.52E-02	1	5
9,11	TFRC|0.403125	ferric iron transmembrane transporter activity	GO:0015091	5.67E-03	3.52E-02	1	11
4	HBA1|2.13735	haptoglobin binding	GO:0031720	5.67E-03	3.52E-02	1	4
5	HSP90AB1|0.458775	UTP binding	GO:0002134	5.67E-03	3.52E-02	1	5
5	CBX3|0.485775	histone methyltransferase binding	GO:1990226	5.67E-03	3.52E-02	1	5
5	HSP90AB1|0.458775	sulfonylurea receptor binding	GO:0017098	5.67E-03	3.52E-02	1	5
4	PCNA|0.404975	DNA polymerase processivity factor activity	GO:0030337	5.67E-03	3.52E-02	1	4
8	TFRC|0.403125	trivalent inorganic cation transmembrane transporter activity	GO:0072510	5.67E-03	3.52E-02	1	8
6	HSP90AB1|0.458775	pyrimidine ribonucleoside binding	GO:0032551	5.67E-03	3.52E-02	1	6
9	PTP4A3|0.3433	prenylated protein tyrosine phosphatase	GO:0004727	5.67E-03	3.52E-02	1	9
6	ACTB|2.1999;H3F3A|0.20985	nucleosomal DNA binding	GO:0031492	5.81E-03	3.56E-02	2	6
4,5	HSPD1|2.88995;ANXA5|3.637725	peptide hormone binding	GO:0017046	6.09E-03	3.69E-02	2	5
3	SARS|2.2366;YARS|2.288275;GMPS|0.496875;MTHFD1L|6.667175;NARS|2.162;LIG1|0.440425;ATG7|2.120975	ligase activity	GO:0016874	6.84E-03	4.10E-02	7	3
5	ESD|2.155525	methylumbelliferyl-acetate deacetylase activity	GO:0047374	8.49E-03	4.33E-02	1	5
9,10	SLC2A1|5.081875	D-glucose transmembrane transporter activity	GO:0055056	8.49E-03	4.33E-02	1	10
5	HIST1H4A|0.23835	histone demethylase activity (H4-K20	GO:0035575	8.49E-03	4.33E-02	1	5
7	CNDP2|2.0938	tripeptidase activity	GO:0034701	8.49E-03	4.33E-02	1	7
8	YWHAG|2.359475	protein kinase C inhibitor activity	GO:0008426	8.49E-03	4.33E-02	1	8
5	PLK1|0.415475	anaphase-promoting complex	GO:0010997	8.49E-03	4.33E-02	1	5
4	SLC2A1|5.081875	dehydroascorbic acid transporter activity	GO:0033300	8.49E-03	4.33E-02	1	4
10	PCNA|0.404975	dinucleotide insertion or deletion binding	GO:0032139	8.49E-03	4.33E-02	1	10
7	PCNA|0.404975	mismatch base pair DNA N-glycosylase activity	GO:0000700	8.49E-03	4.33E-02	1	7
8	YARS|2.288275	interleukin-8 receptor binding	GO:0005153	8.49E-03	4.33E-02	1	8
6,7	HSP90AB1|0.458775	adenyl deoxyribonucleotide binding	GO:0032558	8.49E-03	4.33E-02	1	7
5	HSP90AB1|0.458775	dATP binding	GO:0032564	8.49E-03	4.33E-02	1	5
8	ACAT2|2.108325	acetyl-CoA C-acetyltransferase activity	GO:0003985	8.49E-03	4.33E-02	1	8
6	ASL|2.252975	argininosuccinate lyase activity	GO:0004056	8.49E-03	4.33E-02	1	6

5,6	HSP90AB1|0.458775	pyrimidine ribonucleotide binding	GO:0032557	8.49E-03	4.33E-02	1	6
6	SARS|2.2366;YARS|2.288275;NARS|2.162	aminoacyl-tRNA ligase activity	GO:0004812	9.91E-03	5.00E-02	3	6
4	SARS|2.2366;YARS|2.288275;NARS|2.162	ligase activity, forming carbon-oxygen bonds	GO:0016875	1.04E-02	5.06E-02	3	4
5	SARS|2.2366;YARS|2.288275;NARS|2.162	ligase activity, forming aminoacyl-tRNA and related compounds	GO:0016876	1.04E-02	5.06E-02	3	5
7	YARS|2.288275	tyrosine-tRNA ligase activity	GO:0004831	1.13E-02	5.06E-02	1	7
6	RRM2|0.21675	ribonucleoside-diphosphate reductase activity, thioredoxin disulfide as	GO:0004748	1.13E-02	5.06E-02	1	6
7	ACAT2|2.108325	C-acetyltransferase activity	GO:0016453	1.13E-02	5.06E-02	1	7
8,10	SLC25A1|2.1121	citrate transmembrane transporter activity	GO:0015137	1.13E-02	5.06E-02	1	10
5,6	HSP90AB1|0.458775	purine deoxyribonucleotid e binding	GO:0032554	1.13E-02	5.06E-02	1	6
6	ESD|2.155525	S-formylglutathione	GO:0018738	1.13E-02	5.06E-02	1	6
7,9	SLC25A1|2.1121	tricarboxylic acid transmembrane transporter activity	GO:0015142	1.13E-02	5.06E-02	1	9
5	RRM2|0.21675	oxidoreductase activity, acting on CH or CH2 groups, disulfide as	GO:0016728	1.13E-02	5.06E-02	1	5
8	PRDX5|3.0143	RNA polymerase III regulatory region DNA	GO:0001016	1.13E-02	5.06E-02	1	8
7	DNMT1|0.172	DNA (cytosine-5-)-methyltransferase activity	GO:0003886	1.13E-02	5.06E-02	1	7
6	VIM|2.241075	keratin filament binding	GO:1990254	1.13E-02	5.06E-02	1	6
5	ACTB|2.1999;H3F3A|0.20985	nucleosome binding	GO:0031491	1.15E-02	5.11E-02	2	5
4	DNMT1|0.172;ATG7|2.120975;MAPK14|0.483325;SNW1|0.473175;STAT3|0.4995;ACTB|2.1999	transcription factor binding	GO:0008134	1.18E-02	5.20E-02	6	4
6	PCNA|0.404975;SNW1|0.473175;STAT3|0.4995	nuclear hormone receptor binding	GO:0035257	1.25E-02	5.46E-02	3	6
6	ARHGDIA|3.8215;ALB|0.23405	monocarboxylic acid binding	GO:0033293	1.35E-02	5.64E-02	2	6
6	CKB|0.486625	creatine kinase activity	GO:0004111	1.41E-02	5.64E-02	1	6
4	HSP90AB1|0.458775	deoxyribonucleotid e binding	GO:0032552	1.41E-02	5.64E-02	1	4
5	HSP90AB1|0.458775	TPR domain binding	GO:0030911	1.41E-02	5.64E-02	1	5
6	PSME3|0.401575	endopeptidase activator activity	GO:0061133	1.41E-02	5.64E-02	1	6
3	SLC2A1|5.081875	xenobiotic transporter activity	GO:0042910	1.41E-02	5.64E-02	1	3
5	HSP90AB1|0.458775	pyrimidine nucleotide binding	GO:0019103	1.41E-02	5.64E-02	1	5
5	MAPK14|0.483325	NFAT protein binding	GO:0051525	1.41E-02	5.64E-02	1	5
6	GSN|2.4555	myosin II binding	GO:0045159	1.41E-02	5.64E-02	1	6
5	TFRC|0.403125	transferrin receptor activity	GO:0004998	1.41E-02	5.64E-02	1	5
6	ATG7|2.120975	ubiquitin activating enzyme activity	GO:0004839	1.41E-02	5.64E-02	1	6
9,10	ACTB|2.1999;H3F3A|0.20985	RNA polymerase II distal enhancer sequence-specific DNA binding	GO:0000980	1.48E-02	5.86E-02	2	10
5	MTHFD1L|6.667175;ALDH1L2|2.148	oxidoreductase activity, acting on the CH-NH group of donors, NAD or NADP as acceptor	GO:0016646	1.57E-02	6.13E-02	2	5
6	HSPA8|0.3721;HSPD1|2.88995;ATP6V1B2|2.0575;MCM3|0.35205;NSF|2.017425;GMPS|0.496875;MCM2|0.491625;G3BP1|0.3904;RHOG|2.449775;TOP2A|0.481675;TUBG1|0.4102;MYH10|5.	pyrophosphatase activity	GO:0016462	1.64E-02	6.13E-02	12	6

5	ASL|2.252975	amidine-lyase activity	GO:0016842	1.69E-02	6.13E-02	1	5
5	HSPA8|0.3721	C3HC4-type RING finger domain binding	GO:0055131	1.69E-02	6.13E-02	1	5
7	VDAC3|2.015475	porin activity	GO:0015288	1.69E-02	6.13E-02	1	7
4	ISYNA1|0.49065	intramolecular lyase activity	GO:0016872	1.69E-02	6.13E-02	1	4
5	ISYNA1|0.49065	inositol-3-phosphate synthase	GO:0004512	1.69E-02	6.13E-02	1	5
4,5,6	HSPD1|2.88995	insulin binding	GO:0043559	1.69E-02	6.13E-02	1	6
4	PSME3|0.401575	MDM2/MDM4 family protein binding	GO:0097371	1.69E-02	6.13E-02	1	4
6	PCNA|0.404975	MutLalpha complex binding	GO:0032405	1.69E-02	6.13E-02	1	6
7	ACAT2|2.108325	acetyl-CoA C-acyltransferase activity	GO:0003988	1.69E-02	6.13E-02	1	7
4	HSP90AB1|0.458775	nitric-oxide synthase regulator	GO:0030235	1.69E-02	6.13E-02	1	4
5	HSPA8|0.3721;HSPD1|2.88995;ATP6V1B2|2.0575;MCM3|0.35205;NSF|2.017425;GMPS|0.496875;MCM2|0.491625;G3BP1|0.3904;RHOG|2.449775;TOP2A|0.481675;TUBG1|0.4102;MYH10|5.579275	hydrolase activity, acting on acid anhydrides, in phosphorus-containing anhydrides	GO:0016818	1.73E-02	6.24E-02	12	5
4	HSPA8|0.3721;HSPD1|2.88995;ATP6V1B2|2.0575;MCM3|0.35205;NSF|2.017425;GMPS|0.496875;MCM2|0.491625;G3BP1|0.3904;RHOG|2.449775;TOP2A|0.481675;TUBG1|0.4102;MYH10|5.[illegible]	hydrolase activity, acting on acid anhydrides	GO:0016817	1.80E-02	6.42E-02	12	4
3	HSPD1|2.88995;ANXA5|3.637725	hormone binding	GO:0042562	1.84E-02	6.49E-02	2	3
4	HSPD1|2.88995;PSME3|0.401575	p53 binding	GO:0002039	1.84E-02	6.49E-02	2	4
7	PCNA|0.404975;STAT3|0.4995	steroid hormone receptor binding	GO:0035258	1.89E-02	6.62E-02	2	7
6	YARS|2.288275;STAT3|0.4995	chemokine receptor binding	GO:0042379	1.94E-02	6.68E-02	2	6
6	DNMT1|0.172	DNA-methyltransferase activity	GO:0009008	1.97E-02	6.68E-02	1	6
8	STAT3|0.4995	CCR5 chemokine receptor binding	GO:0031730	1.97E-02	6.68E-02	1	8
5	ACTB|2.1999	Tat protein binding	GO:0030957	1.97E-02	6.68E-02	1	5
8	AKR1B1|2.235325	alditol:NADP+ 1-oxidoreductase activity	GO:0004032	1.97E-02	6.68E-02	1	8
3	HBA1|2.13735;ALB|0.23405	oxygen binding	GO:0019825	2.08E-02	6.97E-02	2	3
7	MAPK14|0.483325;TTK|0.488375	protein serine/threonine/tyrosine kinase	GO:0004712	2.08E-02	6.97E-02	2	7
8	ACTB|2.1999;H3F3A|0.20985	enhancer sequence-specific DNA binding	GO:0001158	2.24E-02	7.24E-02	2	8
6	PABPC1|0.3285;PABPC4|0.26245	single-stranded RNA binding	GO:0003727	2.24E-02	7.24E-02	2	6
4	PABPC1|0.3285	translation activator activity	GO:0008494	2.25E-02	7.24E-02	1	4
5	PCNA|0.404975	DNA polymerase binding	GO:0070182	2.25E-02	7.24E-02	1	5
6,7	TFRC|0.403125	P-P-bond-hydrolysis-driven protein transmembrane transporter activity	GO:0015450	2.25E-02	7.24E-02	1	7
5	PCNA|0.404975	mismatch repair complex binding	GO:0032404	2.25E-02	7.24E-02	1	5
7	NSF|2.017425;IPO8|0.443925;NDRG1|10.0073;ARHGDIA|3.8215	Ras GTPase binding	GO:0017016	2.33E-02	7.44E-02	4	7
4	MTHFD1L|6.667175;ALDH1L2|2.148	oxidoreductase activity, acting on the CH-NH group of donors	GO:0016645	2.34E-02	7.44E-02	2	4
4	HSPA8|0.3721;HSP90AB1|0.458775;YARS|2.288275;YWHAG|2.359475;ANXA5|3.637725;PRDX5|3.0143;SQSTM1|6.007525;PCNA|0.404975;SNW1|0.473175;STAT3|0.4995;VTN|2.01125	receptor binding	GO:0005102	2.38E-02	7.52E-02	11	4

5	MTHFD1L|6.667175;GCH1|4.01735	hydrolase activity, acting on carbon-nitrogen (but not peptide) bonds, in cyclic amidines	GO:0016814	2.45E-02	7.56E-02	2	5
5,7	ACTB|2.1999;H3F3A|0.20985	chromatin DNA binding	GO:0031490	2.50E-02	7.56E-02	2	7
7	NARS|2.162	asparagine-tRNA ligase activity	GO:0004816	2.53E-02	7.56E-02	1	7
3	PRDX5|3.0143	peroxiredoxin activity	GO:0051920	2.53E-02	7.56E-02	1	3
7	SARS|2.2366	serine-tRNA ligase activity	GO:0004828	2.53E-02	7.56E-02	1	7
6	ALDH1L2|2.148	formyltetrahydrofolate dehydrogenase activity	GO:0016155	2.53E-02	7.56E-02	1	6
10	TFRC|0.403125	iron ion transmembrane transporter activity	GO:0005381	2.53E-02	7.56E-02	1	10
7	YARS|2.288275	isoleucine-tRNA ligase activity	GO:0004822	2.53E-02	7.56E-02	1	7
6	KPNA2|0.186675	nuclear localization sequence binding	GO:0008139	2.53E-02	7.56E-02	1	6
3	PRDX5|3.0143;HBA1|2.13735	peroxidase activity	GO:0004601	2.55E-02	7.60E-02	2	3
5	PCNA|0.404975;SNW1|0.473175;STAT3|0.4995	hormone receptor binding	GO:0051427	2.68E-02	7.92E-02	3	5
4	PRDX5|3.0143;HBA1|2.13735	oxidoreductase activity, acting on peroxide as	GO:0016684	2.72E-02	7.97E-02	2	4
6	HSPD1|2.88995;PCNA|0.404975;ACTB|2.1999;H3F3A|0.20985	structure-specific DNA binding	GO:0043566	2.73E-02	7.97E-02	4	6
5	TOP2A|0.481675	DNA topoisomerase II	GO:0061505	2.80E-02	8.07E-02	1	5
8	STAT3|0.4995	glucocorticoid receptor binding	GO:0035259	2.80E-02	8.07E-02	1	8
6,11	TOP2A|0.481675	DNA topoisomerase type II (ATP-hydrolyzing)	GO:0003918	2.80E-02	8.07E-02	1	11
7	HSPA8|0.3721;HSPD1|2.88995;ATP6V1B2|2.0575;MCM3|0.35205;NSF|2.017425;MCM2|0.491625;G3BP1|0.3904;RHOG|2.449775;TOP2A|0.481675;TUBG1|0.4102;MYH10|5.579275	nucleoside-triphosphatase activity	GO:0017111	2.90E-02	8.31E-02	11	7
8	ACTB|2.1999;H3F3A|0.20985	enhancer binding	GO:0035326	2.95E-02	8.38E-02	2	8
6	NSF|2.017425;IPO8|0.443925;NDRG1|10.0073;ARHGDIA|3.8215	small GTPase binding	GO:0031267	2.97E-02	8.40E-02	4	6
5	KPNA2|0.186675;TOP2A|0.481675	histone deacetylase binding	GO:0042826	3.06E-02	8.57E-02	2	5
3	ALB|0.23405	toxic substance binding	GO:0015643	3.08E-02	8.57E-02	1	3
4	RRM2|0.21675	oxidoreductase activity, acting on CH or CH2 groups	GO:0016725	3.08E-02	8.57E-02	1	4
4	HSP90AB1|0.458775;ASPH|2.59965	ion channel binding	GO:0044325	3.18E-02	8.81E-02	2	4
10	MYH10|5.579275	actin-dependent ATPase activity	GO:0030898	3.35E-02	9.24E-02	1	10
4	LCP1|2.170475;GSN|2.4555;ALDOC|2.04725;NDE1|0.446425;NDRG1|10.0073;ACTB|2.1999;MYH10|5.579275;PLK1|0.415475;TBCA|0.3515	cytoskeletal protein binding	GO:0008092	3.49E-02	9.44E-02	9	4
2	HSP90AB1|0.458775;YWHAG|2.359475;ANXA5|3.637725;PRDX5|3.0143;BAZ1B|0.460625;PSME3|0.401575;LAMTOR2|7.841025;PCNA|0.404975;TXNIP|2.0553;ARHGDIA|3.8215;SOS2|11.	molecular function regulator	GO:0098772	3.63E-02	9.44E-02	11	2
5	LIG1|0.440425	DNA ligase activity	GO:0003909	3.63E-02	9.44E-02	1	5
4	HSPD1|2.88995	misfolded protein binding	GO:0051787	3.63E-02	9.44E-02	1	4
9	PCNA|0.404975	DNA insertion or deletion binding	GO:0032135	3.63E-02	9.44E-02	1	9
7	AKR1B1|2.235325	alcohol dehydrogenase (NADP+) activity	GO:0008106	3.63E-02	9.44E-02	1	7
6	LIG1|0.440425	DNA ligase (ATP) activity	GO:0003910	3.63E-02	9.44E-02	1	6
5	VIM|2.241075	intermediate filament binding	GO:0019215	3.63E-02	9.44E-02	1	5
6	ATG7|2.120975	Atg8 activating enzyme activity	GO:0019779	3.63E-02	9.44E-02	1	6
4	S100A11|2.531875	S100 protein binding	GO:0044548	3.63E-02	9.44E-02	1	4
6	ATG7|2.120975	Atg12 activating enzyme activity	GO:0019778	3.63E-02	9.44E-02	1	6

4	VIM|2.241075;TFRC|0.403125	glycoprotein binding	GO:0001948	3.67E-02	9.50E-02	2	4
9	HSPA8|0.3721;ATP6V1B2|2.0575;NSF|2.017425;G3BP1|0.3904;TOP2A|0.481675;MYH10|5.5792	ATPase activity, coupled	GO:0042623	3.92E-02	1.01E-01	6	9
3	ASPH|2.59965;MTHFD1L|6.667175;ALDH1L2|2.148;AKR1B1|2.235325;ERO1L|2.370575;PRDX5|3.0143;RRM2|0.21675;VAT1|2.702925;HBA1|2.13735;SOD2|2.175425	oxidoreductase activity	GO:0016491	4.06E-02	1.04E-01	10	3
5	SNW1|0.473175	Notch binding	GO:0005112	4.18E-02	1.05E-01	1	5
5	ACTB|2.1999	nitric-oxide synthase binding	GO:0050998	4.18E-02	1.05E-01	1	5
6	ACAT2|2.108325	C-acyltransferase activity	GO:0016408	4.18E-02	1.05E-01	1	6
7	ARHGDIA|3.8215	Rho GDP-dissociation inhibitor activity	GO:0005094	4.45E-02	1.10E-01	1	7
5	SNW1|0.473175	vitamin D receptor binding	GO:0042809	4.45E-02	1.10E-01	1	5
5	CKB|0.486625	phosphotransferase activity, nitrogenous group	GO:0016775	4.45E-02	1.10E-01	1	5
5	SQSTM1|6.007525;TOP2A|0.481675	ubiquitin binding	GO:0043130	4.45E-02	1.10E-01	2	5
5	NDE1|0.446425;NDRG1|10.0073;PLK1|0.415475;TBCA|0.3515	tubulin binding	GO:0015631	4.53E-02	1.12E-01	4	5
4	GCH1|4.01735	GTP-dependent protein binding	GO:0030742	4.72E-02	1.15E-01	1	4
5	BAZ1B|0.460625	lysine-acetylated histone binding	GO:0070577	4.72E-02	1.15E-01	1	5
5	TFRC|0.403125	protein transmembrane transporter activity	GO:0008320	4.72E-02	1.15E-01	1	5
5	MTHFD1L|6.667175	formate-tetrahydrofolate ligase activity	GO:0004329	4.99E-02	1.18E-01	1	5
3	PABPC1|0.3285	translation regulator activity, nucleic acid	GO:0090079	4.99E-02	1.18E-01	1	3
3,5	SOD2|2.175425	superoxide dismutase activity	GO:0004784	4.99E-02	1.18E-01	1	5
4	SOD2|2.175425	oxidoreductase activity, acting on superoxide radicals as acceptor	GO:0016721	4.99E-02	1.18E-01	1	4
4	HSPD1|2.88995	lipopolysaccharide binding	GO:0001530	4.99E-02	1.18E-01	1	4
6	ALDOC|2.04725	fructose-bisphosphate aldolase activity	GO:0004332	4.99E-02	1.18E-01	1	6
4	ALDH1L2|2.148;AKR1B1|2.235325	oxidoreductase activity, acting on the aldehyde or oxo group of donors	GO:0016903	5.01E-02	1.18E-01	2	4
4	SQSTM1|6.007525;TOP2A|0.481675	ubiquitin-like protein binding	GO:0032182	5.22E-02	1.21E-01	2	4
4	LIG1|0.440425	ligase activity, forming phosphoric ester bonds	GO:0016886	5.26E-02	1.21E-01	1	4
5,7	DNMT1|0.172	methyl-CpG binding	GO:0008327	5.26E-02	1.21E-01	1	7
7	YARS|2.288275	CXCR chemokine receptor binding	GO:0045236	5.26E-02	1.21E-01	1	7
7	MTHFD1L|6.667175	methenyltetrahydrofolate cyclohydrolase activity	GO:0004477	5.26E-02	1.21E-01	1	7
4	GMPS|0.496875;MTHFD1L|6.667175	ligase activity, forming carbon-nitrogen bonds	GO:0016879	5.29E-02	1.21E-01	2	4
8	NSF|2.017425;NDRG1|10.0073	Rab GTPase binding	GO:0017137	5.37E-02	1.22E-01	2	8
4	TFRC|0.403125;VTN|2.01125	cargo receptor activity	GO:0038024	5.51E-02	1.24E-01	2	4
6	ENO1|2.1939	phosphopyruvate hydratase activity	GO:0004634	5.53E-02	1.24E-01	1	6
8,9	PLEK|2.071125	phosphatidylinositol-3,4-bisphosphate binding	GO:0043325	5.53E-02	1.24E-01	1	9
5	ALDH1L2|2.148	hydroxymethyl-, formyl- and related transferase activity	GO:0016742	5.53E-02	1.24E-01	1	5
3	TOP2A|0.481675;ALB|0.23405	drug binding	GO:0008144	5.66E-02	1.26E-01	2	3

8	HSPA8|0.3721;HSPD1|2.88995;ATP6V1B2|2.0575;NSF|2.017425;G3BP1|0.3904;TOP2A|0.481675;MYH10|5.579275	ATPase activity	GO:0016887	5.71E-02	1.27E-01	7	8
6	AKR1B1|2.235325	aldo-keto reductase (NADP) activity	GO:0004033	5.80E-02	1.27E-01	1	6
5	TFRC|0.403125	Hsp70 protein binding	GO:0030544	5.80E-02	1.27E-01	1	5
7	SQSTM1|6.007525	K63-linked polyubiquitin	GO:0070530	5.80E-02	1.27E-01	1	7
4	YWHAG|2.359475;ANXA5|3.637725;PRDX5|3.0143;TXNIP|2.0553	enzyme inhibitor activity	GO:0004857	5.98E-02	1.30E-01	4	4
6	MTHFD1L|6.667175	methylenetetrahydrofolate dehydrogenase (NADP+) activity	GO:0004488	6.06E-02	1.31E-01	1	6
6	TOP2A|0.481675	DNA binding, bending	GO:0008301	6.06E-02	1.31E-01	1	6
9	MYH10|5.579275	microfilament motor activity	GO:0000146	6.06E-02	1.31E-01	1	9
6	VDAC3|2.015475	wide pore channel activity	GO:0022829	6.33E-02	1.36E-01	1	6
3	HSP90AB1|0.458775;YWHAG|2.359475;ANXA5|3.637725;PRDX5|3.0143;PSME3|0.401575;PCNA|0.404975;TXNIP|2.0553;ARHGDIA|3.8215	enzyme regulator activity	GO:0030234	6.38E-02	1.36E-01	8	3
2	ASPH|2.59965;AKR1B1|2.235325	electron carrier activity	GO:0009055	6.49E-02	1.38E-01	2	2
5	ARHGDIA|3.8215	Hsp90 protein binding	GO:0051879	6.60E-02	1.40E-01	1	5
5	PCNA|0.404975	histone acetyltransferase binding	GO:0035035	6.86E-02	1.44E-01	1	5
5	TFRC|0.403125	macromolecule transmembrane transporter activity	GO:0022884	6.86E-02	1.44E-01	1	5
5	HSPA8|0.3721;YARS|2.288275;STAT3|0.4995	G-protein coupled receptor binding	GO:0001664	6.95E-02	1.45E-01	3	5
6	LCP1|2.170475;ASPH|2.59965;ANXA5|3.637725;GSN|2.4555;EFHD2|2.281;S100A11|2.531875;GCH1|4.01735;CETN3|0.4171	calcium ion binding	GO:0005509	7.05E-02	1.47E-01	8	6
3	PLP1|3.936	structural constituent of	GO:0019911	7.13E-02	1.48E-01	1	3
4	KPNA2|0.186675;TFRC|0.403125	protein transporter activity	GO:0008565	7.20E-02	1.49E-01	2	4
6	STAT3|0.4995	RNA polymerase II repressing transcription factor binding	GO:0001103	7.39E-02	1.52E-01	1	6
4	HBA1|2.13735	oxygen transporter activity	GO:0005344	7.39E-02	1.52E-01	1	4
4	BAZ1B|0.460625	receptor activator activity	GO:0030546	7.92E-02	1.59E-01	1	4
5	ALDOC|2.04725	aldehyde-lyase activity	GO:0016832	7.92E-02	1.59E-01	1	5
4	ASL|2.252975	carbon-nitrogen lyase activity	GO:0016840	7.92E-02	1.59E-01	1	4
5	KPNA2|0.186675	signal sequence binding	GO:0005048	7.92E-02	1.59E-01	1	5
7	CNDP2|2.0938	dipeptidase activity	GO:0016805	7.92E-02	1.59E-01	1	7
4	VTN|2.01125	polysaccharide binding	GO:0030247	8.18E-02	1.63E-01	1	4
3	VTN|2.01125	pattern binding	GO:0001871	8.18E-02	1.63E-01	1	3
4	HSPD1|2.88995;KPNA2|0.186675;ANXA5|3.637725	peptide binding	GO:0042277	8.38E-02	1.67E-01	3	4
5	SQSTM1|6.007525	SH2 domain binding	GO:0042169	8.44E-02	1.67E-01	1	5
5	MYH10|5.579275	ADP binding	GO:0043531	8.44E-02	1.67E-01	1	5
5	NDE1|0.446425;NDRG1|10.0073;PLK1|0.415475	microtubule binding	GO:0008017	8.65E-02	1.70E-01	3	5
3	VIM|2.241075	structural constituent of eye	GO:0005212	8.70E-02	1.70E-01	1	3
5	GMPS|0.496875	carbon-nitrogen ligase activity, with glutamine as amido-N-donor	GO:0016884	8.96E-02	1.74E-01	1	5
6	PCNA|0.404975	DNA N-glycosylase activity	GO:0019104	8.96E-02	1.74E-01	1	6
6	NSF|2.017425	syntaxin-1 binding	GO:0017075	9.22E-02	1.77E-01	1	6
8	PCNA|0.404975	estrogen receptor binding	GO:0030331	9.22E-02	1.77E-01	1	8
9	TFRC|0.403125	transition metal ion transmembrane transporter activity	GO:0046915	9.22E-02	1.77E-01	1	9

5	YWHAG|2.359475	insulin-like growth factor receptor binding	GO:0005159	9.48E-02	1.81E-01	1	5
7	STAT3|0.4995	CCR chemokine receptor binding	GO:0048020	9.73E-02	1.85E-01	1	7
3	SLC2A1|5.081875	vitamin transporter activity	GO:0051183	9.73E-02	1.85E-01	1	3
3	HSPD1|2.88995;KPNA2|0.186675;ANXA5|3.637725	amide binding	GO:0033218	9.87E-02	1.87E-01	3	3
6,9	MAPK14|0.483325	MAP kinase activity	GO:0004707	9.99E-02	1.88E-01	1	9
5,8	PRDX5|3.0143	cysteine-type endopeptidase inhibitor activity involved in apoptotic process	GO:0043027	9.99E-02	1.88E-01	1	8
8	MCM3|0.35205;MCM2|0.491625;G3BP1|0.3904	helicase activity	GO:0004386	1.00E-01	1.88E-01	3	8
4	TOP2A|0.481675	DNA topoisomerase	GO:0003916	1.02E-01	1.90E-01	1	4
7	YWHAG|2.359475	protein serine/threonine kinase inhibitor activity	GO:0030291	1.02E-01	1.90E-01	1	7
3	ENO1|2.1939;ALDOC|2.04725;ASL|2.252975	lyase activity	GO:0016829	1.04E-01	1.92E-01	3	3
6	ARHGDIA|3.8215	GDP-dissociation inhibitor activity	GO:0005092	1.05E-01	1.94E-01	1	6
6	NDRG1|10.0073	gamma-tubulin binding	GO:0043015	1.08E-01	1.96E-01	1	6
2	PABPC1|0.3285	translation regulator activity	GO:0045182	1.08E-01	1.96E-01	1	2
4	HIST1H4A|0.23835	histone demethylase	GO:0032452	1.08E-01	1.96E-01	1	4
10	G3BP1|0.3904;TOP2A|0.481675	DNA-dependent ATPase activity	GO:0008094	1.08E-01	1.97E-01	2	10
6	ANXA5|3.637725	phospholipase inhibitor activity	GO:0004859	1.13E-01	2.02E-01	1	6
8,9	VDAC3|2.015475	voltage-gated anion channel activity	GO:0008308	1.13E-01	2.02E-01	1	9
6	SQSTM1|6.007525	polyubiquitin binding	GO:0031593	1.13E-01	2.02E-01	1	6
6	ERO1L|2.370575	protein disulfide oxidoreductase activity	GO:0015035	1.13E-01	2.02E-01	1	6
5	ERO1L|2.370575	oxidoreductase activity, acting on a sulfur group of donors, disulfide as acceptor	GO:0016671	1.15E-01	2.04E-01	1	5
8	PCNA|0.404975	mismatched DNA binding	GO:0030983	1.15E-01	2.04E-01	1	8
4	SLC2A1|5.081875	protein self-association	GO:0043621	1.15E-01	2.04E-01	1	4
5	ERO1L|2.370575	intramolecular oxidoreductase activity, transposing S-S	GO:0016864	1.18E-01	2.07E-01	1	5
6	ERO1L|2.370575	protein disulfide isomerase activity	GO:0003756	1.18E-01	2.07E-01	1	6
3	ASPH|2.59965	structural constituent of	GO:0008307	1.18E-01	2.07E-01	1	3
7	BAZ1B|0.460625	histone kinase activity	GO:0035173	1.20E-01	2.08E-01	1	7
3	BAZ1B|0.460625	receptor regulator activity	GO:0030545	1.20E-01	2.08E-01	1	3
4	VIM|2.241075	scaffold protein binding	GO:0097110	1.20E-01	2.08E-01	1	4
8	MAPK14|0.483325	MAP kinase kinase activity	GO:0004708	1.20E-01	2.08E-01	1	8
5	ARHGDIA|3.8215;ALB|0.23405	carboxylic acid binding	GO:0031406	1.22E-01	2.10E-01	2	5
5	PSME3|0.401575	peptidase activator activity	GO:0016504	1.23E-01	2.10E-01	1	5
8,9	SLC2A1|5.081875	glucose transmembrane transporter activity	GO:0005355	1.23E-01	2.10E-01	1	9
4	ARHGDIA|3.8215;ALB|0.23405	organic acid binding	GO:0043177	1.23E-01	2.10E-01	2	4
6	HSPD1|2.88995;MCM2|0.491625;DNMT1|0.172;GTF2E1|0.300475;STAT3|0.4995;ACTB|2.1999;H3F3A|0.20985	sequence-specific DNA binding	GO:0043565	1.25E-01	2.13E-01	7	6
5	ANXA5|3.637725	lipase inhibitor activity	GO:0055102	1.25E-01	2.13E-01	1	5

5	ERO1L|2.370575	disulfide oxidoreductase activity	GO:0015036	1.28E-01	2.16E-01	1	5
3	HIST1H4A|0.23835	demethylase activity	GO:0032451	1.28E-01	2.16E-01	1	3
5	ATG7|2.120975	small protein activating enzyme activity	GO:0008641	1.30E-01	2.19E-01	1	5
9,10	H3F3A|0.20985	RNA polymerase II core promoter sequence-specific DNA binding	GO:0000979	1.33E-01	2.22E-01	1	10
3	VTN|2.01125	extracellular matrix binding	GO:0050840	1.40E-01	2.34E-01	1	3
5	ESD|2.155525	thiolester hydrolase activity	GO:0016790	1.42E-01	2.37E-01	1	5
6	ALDH1L2|2.148	aldehyde dehydrogenase (NAD) activity	GO:0004029	1.42E-01	2.37E-01	1	6
6	YARS|2.288275	tRNA binding	GO:0000049	1.47E-01	2.44E-01	1	6
5	STAT3|0.4995	repressing transcription factor binding	GO:0070491	1.50E-01	2.47E-01	1	5
3	ERO1L|2.370575;ISYNA1|0.49065;TOP2A|0.481675	isomerase activity	GO:0016853	1.51E-01	2.48E-01	3	3
7,8	SLC2A1|5.081875	hexose transmembrane transporter activity	GO:0015149	1.52E-01	2.49E-01	1	8
7	SOD2|2.175425	manganese ion binding	GO:0030145	1.52E-01	2.49E-01	1	7
8,9	ATP6V1B2|2.0575	proton-transporting ATPase activity, rotational mechanism	GO:0046961	1.55E-01	2.51E-01	1	9
4	PRDX5|3.0143	cysteine-type endopeptidase regulator activity involved in apoptotic process	GO:0043028	1.55E-01	2.51E-01	1	4
5	SNW1|0.473175	retinoic acid receptor binding	GO:0042974	1.57E-01	2.54E-01	1	5
6	CNDP2|2.0938;DNPEP|2.077575	exopeptidase activity	GO:0008238	1.59E-01	2.56E-01	2	6
5	PCNA|0.404975	hydrolase activity, hydrolyzing N-glycosyl	GO:0016799	1.59E-01	2.56E-01	1	5
4	S100A11|2.531875	calcium-dependent protein binding	GO:0048306	1.62E-01	2.59E-01	1	4
3	RPL3|3.30745;RPL4|2.063325;RPL27|2.15885	structural constituent of	GO:0003735	1.63E-01	2.61E-01	3	3
6,7	SLC2A1|5.081875	monosaccharide transmembrane transporter activity	GO:0015145	1.64E-01	2.62E-01	1	7
4	NDRG1|10.0073;VTN|2.01125	cell adhesion molecule binding	GO:0050839	1.65E-01	2.62E-01	2	4
8	IPO8|0.443925	Ran GTPase binding	GO:0008536	1.66E-01	2.63E-01	1	8
8	ATP6V1B2|2.0575	hydrogen-exporting ATPase activity	GO:0036442	1.66E-01	2.63E-01	1	8
5	PRDX5|3.0143;PSME3|0.401575	endopeptidase regulator activity	GO:0061135	1.68E-01	2.64E-01	2	5
10	G3BP1|0.3904	ATP-dependent DNA helicase activity	GO:0004003	1.69E-01	2.65E-01	1	10
7	DNPEP|2.077575	aminopeptidase activity	GO:0004177	1.71E-01	2.67E-01	1	7
7	ATP6V1B2|2.0575	ATPase activity, coupled to transmembrane movement of ions, rotational mechanism	GO:0044769	1.71E-01	2.67E-01	1	7
5	ACTB|2.1999	kinesin binding	GO:0019894	1.74E-01	2.70E-01	1	5
5	GSN|2.4555	myosin binding	GO:0017022	1.78E-01	2.77E-01	1	5
4	ESD|2.155525	deacetylase activity	GO:0019213	1.85E-01	2.87E-01	1	4
5	ATP6V1B2|2.0575;TFRC|0.403125	primary active transmembrane transporter activity	GO:0015399	1.87E-01	2.87E-01	2	5
6	ATP6V1B2|2.0575;TFRC|0.403125	P-P-bond-hydrolysis-driven transmembrane transporter activity	GO:0015405	1.87E-01	2.87E-01	2	6

<table>
<tr><td>2</td><td>HSPA8|0.3721;ENO1|2.1939;HSPD1|2.88995;SARS|2.2366;ASPH|2.59965;YARS|2.288275;ATP6V1B2|2.0575;MCM3|0.35205;NSF|2.017425;GMPS|0.496875;MTHFD1L|6.667175;CKB|0.486625;NARS|2.162;MCM2|0.491625;DNMT1|0.172;ALDH1L2|2.148;HIST1H4A|0.23835;CNDP2|2.0938;AKR1B1|2.235325;LIG1|0.440425;G3BP1|0.3904;ERO1L|2.370575;PRDX5|3.0143;BAZ1B|0.460625;ESD|2.155525;ISYNA1|0.49065;ALDOC|2.04725;RRM2|0.21675;ACAT2|2.108325;SQSTM1|6.007525;ATG7|2.120975;PCNA|0.404975;MAPK14|0.483325;RHOG|2.449775;TOP2A|0.481675;TUBG1|0.4102;VAT1|2.702925;HBA1|2.13735;PLBD2|2.666725;DNPEP|2.077575;MYH10|5.579275;TTK|0.488375;ASL|2.252975;PLK1|0.415475;GCH1|4.01735;SOD2|2.175425;PTP4A3|0.3433</td><td>catalytic activity</td><td>GO:0003824</td><td>1.88E-01</td><td>2.88E-01</td><td>47</td><td>2</td></tr>
<tr><td>4</td><td>SNW1|0.473175</td><td>SMAD binding</td><td>GO:0046332</td><td>1.92E-01</td><td>2.93E-01</td><td>1</td><td>4</td></tr>
<tr><td>7</td><td>PRDX5|3.0143</td><td>cysteine-type endopeptidase inhibitor activity</td><td>GO:0004869</td><td>1.92E-01</td><td>2.93E-01</td><td>1</td><td>7</td></tr>
<tr><td>5</td><td>ENO1|2.1939;SNW1|0.473175</td><td>transcription corepressor activity</td><td>GO:0003714</td><td>1.93E-01</td><td>2.93E-01</td><td>2</td><td>5</td></tr>
<tr><td>4</td><td>MTHFD1L|6.667175;GCH1|4.01735</td><td>hydrolase activity, acting on carbon-nitrogen (but not peptide) bonds</td><td>GO:0016810</td><td>1.95E-01</td><td>2.96E-01</td><td>2</td><td>4</td></tr>
<tr><td>3</td><td>HSPD1|2.88995;ANXA5|3.637725;PLEK|2.071125;ARHGDIA|3.8215;ALB|0.23405</td><td>lipid binding</td><td>GO:0008289</td><td>1.97E-01</td><td>2.97E-01</td><td>5</td><td>3</td></tr>
<tr><td>6</td><td>YWHAG|2.359475</td><td>protein kinase inhibitor activity</td><td>GO:0004860</td><td>1.99E-01</td><td>3.00E-01</td><td>1</td><td>6</td></tr>
<tr><td>5,6</td><td>LCP1|2.170475;MYH10|5.579275</td><td>actin filament binding</td><td>GO:0051015</td><td>2.01E-01</td><td>3.02E-01</td><td>2</td><td>6</td></tr>
<tr><td>5</td><td>ASPH|2.59965</td><td>oxidoreductase activity, acting on paired donors, with incorporation or reduction of molecular oxygen, 2-oxoglutarate as one donor, and incorporation of one atom each of oxygen into both donors</td><td>GO:0016706</td><td>2.04E-01</td><td>3.05E-01</td><td>1</td><td>5</td></tr>
<tr><td>5</td><td>YWHAG|2.359475</td><td>kinase inhibitor activity</td><td>GO:0019210</td><td>2.06E-01</td><td>3.07E-01</td><td>1</td><td>5</td></tr>
<tr><td>6,7,8</td><td>SLC2A1|5.081875</td><td>cation:sugar symporter activity</td><td>GO:0005402</td><td>2.10E-01</td><td>3.12E-01</td><td>1</td><td>8</td></tr>
<tr><td>7,8,9</td><td>SLC2A1|5.081875</td><td>sugar:proton symporter activity</td><td>GO:0005351</td><td>2.10E-01</td><td>3.12E-01</td><td>1</td><td>9</td></tr>
<tr><td>3</td><td>HSPA8|0.3721;HSP90AB1|0.458775</td><td>antigen binding</td><td>GO:0003823</td><td>2.13E-01</td><td>3.15E-01</td><td>2</td><td>3</td></tr>
<tr><td>4</td><td>ATG7|2.120975</td><td>ligase activity, forming carbon-sulfur bonds</td><td>GO:0016877</td><td>2.22E-01</td><td>3.27E-01</td><td>1</td><td>4</td></tr>
<tr><td>5</td><td>NSF|2.017425</td><td>PDZ domain binding</td><td>GO:0030165</td><td>2.24E-01</td><td>3.29E-01</td><td>1</td><td>5</td></tr>
<tr><td>4</td><td>PRDX5|3.0143;PSME3|0.401575</td><td>peptidase regulator activity</td><td>GO:0061134</td><td>2.25E-01</td><td>3.30E-01</td><td>2</td><td>4</td></tr>
<tr><td>5</td><td>NDRG1|10.0073</td><td>cadherin binding</td><td>GO:0045296</td><td>2.26E-01</td><td>3.31E-01</td><td>1</td><td>5</td></tr>
<tr><td>8</td><td>H3F3A|0.20985</td><td>core promoter sequence-specific DNA binding</td><td>GO:0001046</td><td>2.30E-01</td><td>3.34E-01</td><td>1</td><td>8</td></tr>
<tr><td>4</td><td>STAT3|0.4995</td><td>transcription factor activity, direct ligand regulated sequence-specific DNA binding</td><td>GO:0098531</td><td>2.30E-01</td><td>3.34E-01</td><td>1</td><td>4</td></tr>
<tr><td>5</td><td>STAT3|0.4995</td><td>RNA polymerase II transcription factor activity, ligand-activated sequence-specific DNA binding</td><td>GO:0004879</td><td>2.30E-01</td><td>3.34E-01</td><td>1</td><td>5</td></tr>
<tr><td>7</td><td>ALB|0.23405</td><td>copper ion binding</td><td>GO:0005507</td><td>2.37E-01</td><td>3.43E-01</td><td>1</td><td>7</td></tr>
<tr><td>5</td><td>STAT3|0.4995</td><td>RNA polymerase II transcription factor binding</td><td>GO:0001085</td><td>2.39E-01</td><td>3.43E-01</td><td>1</td><td>5</td></tr>
<tr><td>7</td><td>CNDP2|2.0938</td><td>carboxypeptidase activity</td><td>GO:0004180</td><td>2.39E-01</td><td>3.43E-01</td><td>1</td><td>7</td></tr>
</table>

5	ALDH1L2|2.148	oxidoreductase activity, acting on the aldehyde or oxo group of donors, NAD or NADP as acceptor	GO:0016620	2.39E-01	3.43E-01	1	5
7	BAZ1B|0.460625;TTK|0.488375	protein tyrosine kinase activity	GO:0004713	2.41E-01	3.43E-01	2	7
5	ENO1|2.1939	hydro-lyase activity	GO:0016836	2.41E-01	3.43E-01	1	5
7,8	PLEK|2.071125	phosphatidylinositol bisphosphate	GO:1902936	2.41E-01	3.43E-01	1	8
8	BAZ1B|0.460625	non-membrane spanning protein tyrosine kinase activity	GO:0004715	2.43E-01	3.45E-01	1	8
5	YARS|2.288275;STAT3|0.4995	cytokine receptor binding	GO:0005126	2.47E-01	3.50E-01	2	5
5,6	SLC2A1|5.081875	sugar transmembrane transporter activity	GO:0051119	2.48E-01	3.50E-01	1	6
5	LCP1|2.170475;ENO1|2.1939;ASPH|2.59965;NSF|2.017425;MCM2|0.491625;DNMT1|0.172;ANXA5|3.637725;GSN|2.4555;CNDP2|2.0938;GTF2E1|0.300475;LIG1|0.440425;BAZ1B|0.460625;RRM2|0.21675;SQSTM1|6.007525;TOP2A|0.481675;EFHD2|2.281;VAT1|2.702925;S100A11|2.531875;HBA1|2.13735;DNPEP|2.077575;ALB|0.23405;GCH1|4.01735;SOD2|2.175425;CETN3|0.41	metal ion binding	GO:0046872	2.54E-01	3.58E-01	25	5
8	SLC2A1|5.081875	solute:proton symporter activity	GO:0015295	2.56E-01	3.60E-01	1	8
5	HSPD1|2.88995	protease binding	GO:0002020	2.61E-01	3.65E-01	1	5
5	VTN|2.01125	integrin binding	GO:0005178	2.71E-01	3.78E-01	1	5
3,4	LAMTOR2|7.841025	protein complex scaffold	GO:0032947	2.75E-01	3.82E-01	1	4
5	VTN|2.01125	scavenger receptor activity	GO:0005044	2.75E-01	3.82E-01	1	5
4	ERO1L|2.370575	oxidoreductase activity, acting on a sulfur group of donors	GO:0016667	2.79E-01	3.86E-01	1	4
7	PRDX5|3.0143;STAT3|0.4995;ACTB|2.1999;H3F3A|0.20985	transcription regulatory region DNA binding	GO:0044212	2.80E-01	3.86E-01	4	7
6	ENO1|2.1939;TOP2A|0.481675	magnesium ion binding	GO:0000287	2.84E-01	3.91E-01	2	6
4	LCP1|2.170475;ENO1|2.1939;ASPH|2.59965;NSF|2.017425;MCM2|0.491625;DNMT1|0.172;ANXA5|3.637725;GSN|2.4555;CNDP2|2.0938;GTF2E1|0.300475;LIG1|0.440425;BAZ1B|0.460625;RRM2|0.21675;SQSTM1|6.007525;TOP2A|0.481675;EFHD2|2.281;VAT1|2.702925;S100A11|2.531875;HBA1|2.13735;DNPEP|2.077575;ALB|0.23405;GCH1|4.01735;SOD2|2.175425;CETN3|0.41	cation binding	GO:0043169	2.84E-01	3.91E-01	25	4
6	STAT3|0.4995	protein phosphatase binding	GO:0019903	2.87E-01	3.94E-01	1	6
7	VDAC3|2.015475	anion channel activity	GO:0005253	2.89E-01	3.95E-01	1	7
6	HSP90AB1|0.458775;RHOG|2.449775;TUBG1|0.4102;GCH1|4.01735	GTP binding	GO:0005525	2.90E-01	3.95E-01	4	6
3	SLC2A1|5.081875	carbohydrate transporter activity	GO:1901476	2.91E-01	3.95E-01	1	3
4,5	SLC2A1|5.081875	carbohydrate transmembrane transporter activity	GO:0015144	2.91E-01	3.95E-01	1	5
7	ATP6V1B2|2.0575	cation-transporting ATPase activity	GO:0019829	3.00E-01	4.05E-01	1	7
4	ALDOC|2.04725	carbon-carbon lyase activity	GO:0016830	3.02E-01	4.05E-01	1	4
6	ATP6V1B2|2.0575	ATPase activity, coupled to transmembrane movement of ions	GO:0042625	3.02E-01	4.05E-01	1	6
7	SQSTM1|6.007525;MAPK14|0.483325;TTK|0.488375;PLK1|0.415475	protein serine/threonine kinase activity	GO:0004674	3.03E-01	4.05E-01	4	7
9	STAT3|0.4995;ACTB|2.1999	RNA polymerase II core promoter proximal region sequence-specific DNA binding	GO:0000978	3.03E-01	4.05E-01	2	9

8,9	STAT3|0.4995;ACTB|2.1999;H3F3A|0.20985	RNA polymerase II regulatory region sequence-specific DNA binding	GO:0000977	3.04E-01	4.05E-01	3	9
6,7	HSP90AB1|0.458775;RHOG|2.449775;TUBG1|0.4102;GCH1|4.01735	guanyl ribonucleotide binding	GO:0032561	3.05E-01	4.05E-01	4	7
8	STAT3|0.4995;ACTB|2.1999;H3F3A|0.20985	RNA polymerase II regulatory region DNA binding	GO:0001012	3.07E-01	4.05E-01	3	8
5	PRDX5|3.0143;STAT3|0.4995;ACTB|2.1999;H3F3A|0.20985	regulatory region nucleic acid	GO:0001067	3.07E-01	4.05E-01	4	5
6	PRDX5|3.0143;STAT3|0.4995;ACTB|2.1999;H3F3A|0.20985	regulatory region DNA binding	GO:0000975	3.07E-01	4.05E-01	4	6
4	ERO1L|2.370575	intramolecular oxidoreductase activity	GO:0016860	3.07E-01	4.05E-01	1	4
4,5	ALB|0.23405	pyridoxal phosphate binding	GO:0030170	3.11E-01	4.08E-01	1	5
7	EIF4H|0.275675	translation initiation factor	GO:0003743	3.11E-01	4.08E-01	1	7
3	HSPA8|0.3721;HSPD1|2.88995;ATP6V1B2|2.0575;MCM3|0.35205;NSF|2.017425;GMPS|0.496875;MTHFD1L|6.667175;MCM2|0.491625;CNDP2|2.0938;G3BP1|0.3904;ESD|2.155525;PCNA|0.404975;RHOG|2.449775;TOP2A|0.481675;TUBG1|0.4102;PLBD2|2.666725;DNPEP|2.077575;MYH10|5.579275;GCH1|4.01735;PTP4A3|0.3433	hydrolase activity	GO:0016787	3.14E-01	4.11E-01	20	3
6	HSP90AB1|0.458775;RHOG|2.449775;TUBG1|0.4102;GCH1|4.01735	guanyl nucleotide binding	GO:0019001	3.15E-01	4.11E-01	4	6
6	PCNA|0.404975	damaged DNA binding	GO:0003684	3.17E-01	4.12E-01	1	6
3	KRT1|0.4198;VTN|2.01125	carbohydrate binding	GO:0030246	3.17E-01	4.12E-01	2	3
4	ASPH|2.59965	dioxygenase activity	GO:0051213	3.21E-01	4.15E-01	1	4
6,7	PLEK|2.071125	phosphatidylinositol phosphate binding	GO:1901981	3.21E-01	4.15E-01	1	7
8	STAT3|0.4995;ACTB|2.1999	core promoter proximal region sequence-specific DNA binding	GO:0000987	3.34E-01	4.30E-01	2	8
8	H3F3A|0.20985	core promoter binding	GO:0001047	3.35E-01	4.30E-01	1	8
8	STAT3|0.4995;ACTB|2.1999	core promoter proximal region DNA binding	GO:0001159	3.36E-01	4.31E-01	2	8
4	ENO1|2.1939	carbon-oxygen lyase activity	GO:0016835	3.42E-01	4.37E-01	1	4
6	CNDP2|2.0938;DNPEP|2.077575	metallopeptidase activity	GO:0008237	3.52E-01	4.48E-01	2	6
7	HSPD1|2.88995	single-stranded DNA binding	GO:0003697	3.61E-01	4.59E-01	1	7
8	ARHGDIA|3.8215	Rho GTPase binding	GO:0017048	3.66E-01	4.64E-01	1	8
6	ACAT2|2.108325	acetyltransferase activity	GO:0016407	3.68E-01	4.66E-01	1	6
5	STAT3|0.4995	phosphatase binding	GO:0019902	3.72E-01	4.69E-01	1	5
5	DNMT1|0.172;ALDH1L2|2.148	methyltransferase activity	GO:0008168	3.74E-01	4.71E-01	2	5
7	STAT3|0.4995;ACTB|2.1999;H3F3A|0.20985	transcription regulatory region sequence-specific DNA binding	GO:0000976	3.76E-01	4.73E-01	3	7
3	LAMTOR2|7.841025;SOS2|11.48525	guanyl-nucleotide exchange factor activity	GO:0005085	3.82E-01	4.79E-01	2	3
4	DNMT1|0.172;ALDH1L2|2.148	transferase activity, transferring one-carbon groups	GO:0016741	3.95E-01	4.94E-01	2	4
4,5	VTN|2.01125	heparin binding	GO:0008201	4.00E-01	4.98E-01	1	5
5,8	MAPK14|0.483325	receptor signaling protein serine/threonine kinase activity	GO:0004702	4.03E-01	5.01E-01	1	8
7	PCNA|0.404975	double-stranded DNA binding	GO:0003690	4.06E-01	5.04E-01	1	7
5	SOS2|11.48525	Rho guanyl-nucleotide exchange factor	GO:0005089	4.18E-01	5.17E-01	1	5
5,6	ANXA5|3.637725	calcium-dependent phospholipid	GO:0005544	4.20E-01	5.17E-01	1	6

5	YWHAG|2.359475	protein kinase regulator activity	GO:0019887	4.20E-01	5.17E-01	1	5
7	G3BP1|0.3904	mRNA binding	GO:0003729	4.21E-01	5.18E-01	1	7
5	LCP1|2.170475;GSN|2.4555;MYH10|5.579275	actin binding	GO:0003779	4.34E-01	5.32E-01	3	5
8	RHOG|2.449775;TUBG1|0.4102	GTPase activity	GO:0003924	4.41E-01	5.40E-01	2	8
4	ATP6V1B2|2.0575;TFRC|0.403125;SLC2A1|5.081875	active transmembrane transporter activity	GO:0022804	4.43E-01	5.41E-01	3	4
6	EIF4H|0.275675	translation factor activity, RNA binding	GO:0008135	4.50E-01	5.48E-01	1	6
5	NSF|2.017425	syntaxin binding	GO:0019905	4.55E-01	5.52E-01	1	5
5	ESD|2.155525	carboxylic ester hydrolase activity	GO:0052689	4.55E-01	5.52E-01	1	5
4	YWHAG|2.359475	kinase regulator activity	GO:0019207	4.72E-01	5.70E-01	1	4
6	DNMT1|0.172;BAZ1B|0.460625;SQSTM1|6.007525;VAT1|2.702925;HBA1|2.13735;DNPEP|2.077575;ALB|0.23405;GCH1|4.01735;SOD2|2.1754	transition metal ion binding	GO:0046914	4.72E-01	5.70E-01	9	6
10	G3BP1|0.3904	RNA-dependent ATPase activity	GO:0008186	4.75E-01	5.70E-01	1	10
10,11	G3BP1|0.3904	ATP-dependent RNA helicase activity	GO:0004004	4.75E-01	5.70E-01	1	11
9	ATP6V1B2|2.0575	hydrogen ion transmembrane transporter activity	GO:0015078	4.77E-01	5.71E-01	1	9
9	G3BP1|0.3904	RNA helicase activity	GO:0003724	4.78E-01	5.71E-01	1	9
8	PTP4A3|0.3433	protein tyrosine phosphatase	GO:0004725	4.84E-01	5.77E-01	1	8
5	AKR1B1|2.235325	oxidoreductase activity, acting on the CH-OH group of donors, NAD or NADP as acceptor	GO:0016616	4.88E-01	5.81E-01	1	5
6	STAT3|0.4995	transcriptional activator activity, RNA polymerase II core promoter proximal region sequence-specific binding	GO:0001077	4.94E-01	5.86E-01	1	6
6	VDAC3|2.015475;SLC25A1|2.1121	anion transmembrane transporter activity	GO:0008509	4.96E-01	5.87E-01	2	6
6	PRDX5|3.0143	endopeptidase inhibitor activity	GO:0004866	5.15E-01	6.09E-01	1	6
3	ALB|0.23405;GCH1|4.01735	cofactor binding	GO:0048037	5.20E-01	6.11E-01	2	3
4	AKR1B1|2.235325	oxidoreductase activity, acting on CH-OH group of donors	GO:0016614	5.20E-01	6.11E-01	1	4
4	MAPK14|0.483325	receptor signaling protein activity	GO:0005057	5.22E-01	6.13E-01	1	4
4	SOS2|11.48525	Ras guanyl-nucleotide exchange factor	GO:0005088	5.28E-01	6.16E-01	1	4
4	MYH10|5.579275	calmodulin binding	GO:0005516	5.28E-01	6.16E-01	1	4
4	VTN|2.01125	glycosaminoglycan binding	GO:0005539	5.33E-01	6.21E-01	1	4
5,11	ATP6V1B2|2.0575	ATPase activity, coupled to transmembrane movement of substances	GO:0042626	5.37E-01	6.22E-01	1	11
7	SLC2A1|5.081875	solute:cation symporter activity	GO:0015294	5.37E-01	6.22E-01	1	7
5	PRDX5|3.0143	peptidase inhibitor activity	GO:0030414	5.37E-01	6.22E-01	1	5
6	G3BP1|0.3904	endonuclease activity	GO:0004519	5.38E-01	6.22E-01	1	6
4	ATP6V1B2|2.0575	hydrolase activity, acting on acid anhydrides, catalyzing transmembrane movement of substances	GO:0016820	5.46E-01	6.29E-01	1	4

3	VTN|2.01125	sulfur compound binding	GO:1901681	5.53E-01	6.35E-01	1	3
6	DNMT1|0.172	S-adenosylmethionine-dependent methyltransferase activity	GO:0008757	5.54E-01	6.36E-01	1	6
6	BAZ1B|0.460625;SQSTM1|6.007525;MAPK14|0.483325;TTK|0.488375;PLK1|0.415475	protein kinase activity	GO:0004672	5.60E-01	6.41E-01	5	6
6,8	SLC25A1|2.1121	carboxylic acid transmembrane transporter activity	GO:0046943	5.62E-01	6.41E-01	1	8
4,5	ANXA5|3.637725;PLEK|2.071125	phospholipid binding	GO:0005543	5.65E-01	6.44E-01	2	5
3	ATP6V1B2|2.0575;KPNA2|0.186675;VDAC3|2.015475;SLC25A1|2.1121;TFRC|0.403125;SLC2A1|5.081875;HBA1|2.13735	substrate-specific transporter activity	GO:0022892	5.67E-01	6.44E-01	7	3
5	SLC25A1|2.1121	organic acid transmembrane transporter activity	GO:0005342	5.68E-01	6.44E-01	1	5
9	G3BP1|0.3904	purine NTP-dependent helicase activity	GO:0070035	5.79E-01	6.52E-01	1	9
10	G3BP1|0.3904	ATP-dependent helicase activity	GO:0008026	5.79E-01	6.52E-01	1	10
5	HBA1|2.13735	heme binding	GO:0020037	5.79E-01	6.52E-01	1	5
4	PCNA|0.404975	hydrolase activity, acting on glycosyl bonds	GO:0016798	5.91E-01	6.64E-01	1	4
6	SLC2A1|5.081875	symporter activity	GO:0015293	5.92E-01	6.64E-01	1	6
4	HBA1|2.13735	tetrapyrrole binding	GO:0046906	5.93E-01	6.64E-01	1	4
4	NSF|2.017425	SNARE binding	GO:0000149	5.95E-01	6.65E-01	1	4
5	STAT3|0.4995	transcriptional activator activity, RNA polymerase II transcription regulatory region sequence-specific binding	GO:0001228	6.01E-01	6.70E-01	1	5
10	ATP6V1B2|2.0575	ATPase activity, coupled to movement of substances	GO:0043492	6.17E-01	6.86E-01	1	10
5,6	PLEK|2.071125	phosphatidylinositol binding	GO:0035091	6.20E-01	6.88E-01	1	6
4	PSME3|0.401575;ARHGDIA|3.8215	enzyme activator activity	GO:0008047	6.24E-01	6.91E-01	2	4
5	STAT3|0.4995	transcription factor activity, RNA polymerase II core promoter proximal region sequence-specific binding	GO:0000982	6.26E-01	6.91E-01	1	5
7	SLC25A1|2.1121	organic anion transmembrane transporter activity	GO:0008514	6.27E-01	6.91E-01	1	7
4	ENO1|2.1939;SNW1|0.473175	transcription cofactor activity	GO:0003712	6.30E-01	6.93E-01	2	4
7	HBA1|2.13735	iron ion binding	GO:0005506	6.31E-01	6.93E-01	1	7
5	CKB|0.486625;BAZ1B|0.460625;SQSTM1|6.007525;MAPK14|0.483325;TTK|0.488375;PLK1|0.4	kinase activity	GO:0016301	6.34E-01	6.95E-01	6	5
5	SNW1|0.473175	transcription coactivator activity	GO:0003713	6.35E-01	6.95E-01	1	5
8	MYH10|5.579275	motor activity	GO:0003774	6.56E-01	7.14E-01	1	8
4	ASPH|2.59965	oxidoreductase activity, acting on paired donors, with incorporation or reduction of molecular oxygen	GO:0016705	6.57E-01	7.14E-01	1	4
5	ACAT2|2.108325	transferase activity, transferring acyl groups other than amino-acyl groups	GO:0016747	6.57E-01	7.14E-01	1	5
3	ENO1|2.1939;SNW1|0.473175	transcription factor activity, transcription factor binding	GO:0000989	6.65E-01	7.21E-01	2	3
2	ENO1|2.1939;SNW1|0.473175	transcription factor activity, protein binding	GO:0000988	6.67E-01	7.22E-01	2	2

5	ATP6V1B2|2.0575;VDAC3|2.015475;SLC25A1|2.1121;TFRC|0.403125;SLC2A1|5.081875	ion transmembrane transporter activity	GO:0015075	6.69E-01	7.23E-01	5	5
7	PTP4A3|0.3433	phosphoprotein phosphatase	GO:0004721	6.86E-01	7.38E-01	1	7
7	VDAC3|2.015475	voltage-gated channel activity	GO:0022832	6.88E-01	7.38E-01	1	7
7,8	VDAC3|2.015475	voltage-gated ion channel activity	GO:0005244	6.88E-01	7.38E-01	1	8
5	BAZ1B|0.460625;SQSTM1|6.007525;MAPK14|0.483325;TTK|0.488375;PLK1|0.415475	phosphotransferase activity, alcohol group as acceptor	GO:0016773	7.03E-01	7.52E-01	5	5
7	DNMT1|0.172;BAZ1B|0.460625;SQSTM1|6.007525;VAT1|2.702925;DNPEP|2.077575;GCH1|4.0	zinc ion binding	GO:0008270	7.16E-01	7.65E-01	6	7
4	GCH1|4.01735	coenzyme binding	GO:0050662	7.22E-01	7.70E-01	1	4
4	ATP6V1B2|2.0575;VDAC3|2.015475;SLC25A1|2.1121;TFRC|0.403125;SLC2A1|5.081875	substrate-specific transmembrane transporter activity	GO:0022891	7.25E-01	7.72E-01	5	4
5	G3BP1|0.3904	nuclease activity	GO:0004518	7.34E-01	7.79E-01	1	5
2	ATP6V1B2|2.0575;KPNA2|0.186675;VDAC3|2.015475;SLC25A1|2.1121;TFRC|0.403125;SLC2A1|5.081875;HBA1|2.13735	transporter activity	GO:0005215	7.51E-01	7.95E-01	7	2
5,6	ARHGDIA|3.8215	GTPase activator activity	GO:0005096	7.60E-01	8.04E-01	1	6
6	ATP6V1B2|2.0575;TFRC|0.403125;SLC2A1|5.081875	cation transmembrane transporter activity	GO:0008324	7.71E-01	8.14E-01	3	6
4	ACAT2|2.108325	transferase activity, transferring acyl groups	GO:0016746	7.77E-01	8.18E-01	1	4
4	CKB|0.486625;BAZ1B|0.460625;SQSTM1|6.007525;MAPK14|0.483325;TTK|0.488375;PLK1|0.415475	transferase activity, transferring phosphorus-containing groups	GO:0016772	7.88E-01	8.28E-01	6	4
5	ARHGDIA|3.8215	GTPase regulator activity	GO:0030695	7.99E-01	8.38E-01	1	5
3	ATP6V1B2|2.0575;VDAC3|2.015475;SLC25A1|2.1121;TFRC|0.403125;SLC2A1|5.081875	transmembrane transporter activity	GO:0022857	8.02E-01	8.40E-01	5	3
5	SLC2A1|5.081875	secondary active transmembrane transporter activity	GO:0015291	8.07E-01	8.43E-01	1	5
4	ARHGDIA|3.8215	nucleoside-triphosphatase regulator activity	GO:0060589	8.16E-01	8.50E-01	1	4
6	PTP4A3|0.3433	phosphatase activity	GO:0016791	8.24E-01	8.58E-01	1	6
7	ATP6V1B2|2.0575;TFRC|0.403125	inorganic cation transmembrane transporter activity	GO:0022890	8.29E-01	8.61E-01	2	7
4	G3BP1|0.3904;ESD|2.155525;PTP4A3|0.3433	hydrolase activity, acting on ester	GO:0016788	8.43E-01	8.73E-01	3	4
2	KRT1|0.4198;YARS|2.288275;MAPK14|0.483325;TFRC|0.403125;STAT3|0.4995;VTN|2.01125	molecular transducer activity	GO:0060089	8.58E-01	8.88E-01	6	2
6	VDAC3|2.015475	gated channel activity	GO:0022836	8.82E-01	9.10E-01	1	6
8	ATP6V1B2|2.0575	monovalent inorganic cation transmembrane transporter activity	GO:0015077	8.85E-01	9.12E-01	1	8
5	PTP4A3|0.3433	phosphoric ester hydrolase activity	GO:0042578	9.09E-01	9.34E-01	1	5
8	TFRC|0.403125	metal ion transmembrane transporter activity	GO:0046873	9.19E-01	9.43E-01	1	8
4	STAT3|0.4995	RNA polymerase II transcription factor activity, sequence-specific DNA binding	GO:0000981	9.28E-01	9.50E-01	1	4
6	VDAC3|2.015475	ion channel activity	GO:0005216	9.32E-01	9.52E-01	1	6
5	VDAC3|2.015475	substrate-specific channel activity	GO:0022838	9.35E-01	9.53E-01	1	5
5	VDAC3|2.015475	channel activity	GO:0015267	9.40E-01	9.55E-01	1	5
4	VDAC3|2.015475	passive transmembrane transporter activity	GO:0022803	9.40E-01	9.55E-01	1	4
5	CNDP2|2.0938;DNPEP|2.077575	peptidase activity, acting on L-amino acid peptides	GO:0070011	9.53E-01	9.67E-01	2	5
4	CNDP2|2.0938;DNPEP|2.077575	peptidase activity	GO:0008233	9.58E-01	9.69E-01	2	4
3	KRT1|0.4198;TFRC|0.403125;VTN|2.01125	receptor activity	GO:0004872	9.73E-01	9.82E-01	3	3

3	CKB|0.486625;DNMT1|0.172;ALDH1L2|2.148;BAZ1B|0.460625;ACAT2|2.108325;SQSTM1|6.007525;MAPK14|0.483325;TTK|0.488375;PLK1|0	transferase activity	GO:0016740	9.79E-01	9.87E-01	9	3
3	YARS|2.288275;MAPK14|0.483325;STAT3|0.4995	signal transducer activity	GO:0004871	9.82E-01	9.88E-01	3	3
3	ENO1|2.1939;STAT3|0.4995	transcription factor activity, sequence-specific DNA binding	GO:0003700	9.95E-01	9.97E-01	2	3
2	ENO1|2.1939;STAT3|0.4995	nucleic acid binding transcription factor activity	GO:0001071	9.95E-01	9.97E-01	2	2
1	HSPA8|0.3721;ACTG1|2.1999;HSP90AB1|0.458775;LCP1|2.170475;ENO1|2.1939;HSPD1|2.88995;HIST1H2BN|0.481625;RPL3|3.30745;VIM|2.241075;CCT7|2.06135;PABPC1|0.3285;SARS|2.2366;ASPH|2.59965;KRT1|0.4198;YARS|2.288275;ATP6V1B2|2.0575;MCM3|0.35205;KRT9|0.268525;RPL4|2.063325;NSF|2.017425;GMPS|0.496875;MTHFD1L|6.667175;CKB|0.486625;NARS|2.162;MCM2|0.491625;HSPA4L|2.13105;SMC2|0.485025;DNMT1|0.172;ALDH1L2|2.148;HIST1H4A|0.23835;KPNA2|0.186675;VDAC3|2.015475;YWHAG|2.359475;ANXA5|3.637725;SRRT|0.39875;GSN|2.4555;CNDP2|2.0938;NCAPG|0.4528;CBX3|0.485775;GTF2E1|0.300475;AKR1B1|2.235325;PABPC4|0.26245;LIG1|0.440425;SLC25A1|2.1121;G3BP1|0.3904;ERO1L|2.370575;ANKRD28|3.3035;PRDX5|3.0143;BAZ1B|0.460625;ESD|2.155525;ISYNA1|0.49065;ALDOC|2.04725;PSME3|0.401575;RRM2|0.21675;EIF4H|0.275675;ACAT2|2.108325;LAMTOR2|7.841025;MCMBP|0.4769;RPL27|2.15885;SQSTM1|6.007525;ATG7|2.120975;PCNA|0.404975;MAPK14|0.483325;HIST1H3A|0.455625;BTF3|0.2449;TFRC|0.403125;RHOG|2.449775;TOP2A|0.481675;SNW1|0.473175;STAT3|0.4995;FNDC3B|2.328575;TUBG1|0.4102;EFHD2|2.281;NCDN|0.4114;SLC2A1|5.081875;TXNIP|2.0553;VAT1|2.702925;IPO8|0.443925;PLEK|2.071125;NDE1|0.446425;S100A11|2.531875;FAM192A|0.462425;HBA1|2.13735;NDRG1|10.0073;IK|0.480125;VTN|2.01125;RBBP7|0.218475;ARHGDIA|3.8215;PLBD2|2.666725;ACTB|2.1999;DNPEP|2.077575;HIST2H2AA3|0.29265;H3F3A|0.20985;MYH10|5.579275;TTK|0.488375;SOS2|11.48525;ASL|2.252975;ALB|0.23405;PLK1|0.415475;GCH1|4.01735;SOD2|2.175425;PTP4A3|0.3433;TBCA|0.3515;CETN3|0.4171;ZNF706|0.3974;CHAF1B|0.2723;PLP1|3.	molecular_function	GO:0003674	1.00E+00	1.00E+00	107	1

Table S8 PPI analysis of differentially expressed proteins

node1	node1_gene	node1_string	node2	node2_gene	node2_string	score
P11142	HSPA8	9606.ENSP00000227378	O95757	HSPA4L	9606.ENSP00000296464	637
P11142	HSPA8	9606.ENSP00000227378	P10809	HSPD1	9606.ENSP00000340019	972
P11142	HSPA8	9606.ENSP00000227378	P11940	PABPC1	9606.ENSP00000313007	964
P11142	HSPA8	9606.ENSP00000227378	P36578	RPL4	9606.ENSP00000311430	555
P11142	HSPA8	9606.ENSP00000227378	P08238	HSP90AB1	9606.ENSP00000325875	997
P11142	HSPA8	9606.ENSP00000227378	P61289	PSME3	9606.ENSP00000293362	915
P11142	HSPA8	9606.ENSP00000227378	P61981	YWHAG	9606.ENSP00000306330	508
P11142	HSPA8	9606.ENSP00000227378	P12277	CKB	9606.ENSP00000299198	424
P11142	HSPA8	9606.ENSP00000227378	P04179	SOD2	9606.ENSP00000356022	521
P11142	HSPA8	9606.ENSP00000227378	P46459	NSF	9606.ENSP00000381293	404
P11142	HSPA8	9606.ENSP00000227378	P39023	RPL3	9606.ENSP00000346001	525
P11142	HSPA8	9606.ENSP00000227378	P12004	PCNA	9606.ENSP00000368438	556
P11142	HSPA8	9606.ENSP00000227378	Q92597	NDRG1	9606.ENSP00000319977	827
P11142	HSPA8	9606.ENSP00000227378	O95352	ATG7	9606.ENSP00000346437	421
P11142	HSPA8	9606.ENSP00000227378	P02786	TFRC	9606.ENSP00000353224	434
P11142	HSPA8	9606.ENSP00000227378	Q99832	CCT7	9606.ENSP00000258091	808
P11142	HSPA8	9606.ENSP00000227378	P06733	ENO1	9606.ENSP00000234590	676
P11142	HSPA8	9606.ENSP00000227378	Q6UB35	MTHFD1L	9606.ENSP00000356290	504
P11142	HSPA8	9606.ENSP00000227378	P30793	GCH1	9606.ENSP00000378890	735
P11142	HSPA8	9606.ENSP00000227378	P63261	ACTG1	9606.ENSP00000331514	574
P11142	HSPA8	9606.ENSP00000227378	P54577	YARS	9606.ENSP00000362576	739
P11142	HSPA8	9606.ENSP00000227378	P49915	GMPS	9606.ENSP00000419851	632
P63261	ACTG1	9606.ENSP00000331514	P08758	ANXA5	9606.ENSP00000296511	634
P63261	ACTG1	9606.ENSP00000331514	P08238	HSP90AB1	9606.ENSP00000325875	814
P63261	ACTG1	9606.ENSP00000331514	P13796	LCP1	9606.ENSP00000315757	848
P63261	ACTG1	9606.ENSP00000331514	P06396	GSN	9606.ENSP00000362924	693
P63261	ACTG1	9606.ENSP00000331514	Q99832	CCT7	9606.ENSP00000258091	695
P63261	ACTG1	9606.ENSP00000331514	P12004	PCNA	9606.ENSP00000368438	430
P63261	ACTG1	9606.ENSP00000331514	Q9Y277	VDAC3	9606.ENSP00000428845	417
P63261	ACTG1	9606.ENSP00000331514	P35580	MYH10	9606.ENSP00000269243	718
P63261	ACTG1	9606.ENSP00000331514	P06733	ENO1	9606.ENSP00000234590	503
P63261	ACTG1	9606.ENSP00000331514	Q16539	MAPK14	9606.ENSP00000229794	539
P63261	ACTG1	9606.ENSP00000331514	P10809	HSPD1	9606.ENSP00000340019	457
P63261	ACTG1	9606.ENSP00000331514	P84095	RHOG	9606.ENSP00000339467	559
P63261	ACTG1	9606.ENSP00000331514	O95352	ATG7	9606.ENSP00000346437	413
P08238	HSP90AB1	9606.ENSP00000325875	P06733	ENO1	9606.ENSP00000234590	748
P08238	HSP90AB1	9606.ENSP00000325875	P61981	YWHAG	9606.ENSP00000306330	570
P08238	HSP90AB1	9606.ENSP00000325875	P15121	AKR1B1	9606.ENSP00000285930	715
P08238	HSP90AB1	9606.ENSP00000325875	P09972	ALDOC	9606.ENSP00000226253	423

P08238	HSP90AB1	9606.ENSP00000325875	Q9Y277	VDAC3	9606.ENSP00000428845	486
P08238	HSP90AB1	9606.ENSP00000325875	O95757	HSPA4L	9606.ENSP00000296464	965
P08238	HSP90AB1	9606.ENSP00000325875	Q16539	MAPK14	9606.ENSP00000229794	750
P08238	HSP90AB1	9606.ENSP00000325875	Q99832	CCT7	9606.ENSP00000258091	858
P08238	HSP90AB1	9606.ENSP00000325875	P35580	MYH10	9606.ENSP00000269243	744
P08238	HSP90AB1	9606.ENSP00000325875	P84095	RHOG	9606.ENSP00000339467	582
P08238	HSP90AB1	9606.ENSP00000325875	P49736	MCM2	9606.ENSP00000265056	452
P08238	HSP90AB1	9606.ENSP00000325875	P21281	ATP6V1B2	9606.ENSP00000276390	602
P08238	HSP90AB1	9606.ENSP00000325875	P40763	STAT3	9606.ENSP00000264657	668
P08238	HSP90AB1	9606.ENSP00000325875	P39023	RPL3	9606.ENSP00000346001	890
P08238	HSP90AB1	9606.ENSP00000325875	Q07890	SOS2	9606.ENSP00000216373	493
P08238	HSP90AB1	9606.ENSP00000325875	P10809	HSPD1	9606.ENSP00000340019	967
P13796	LCP1	9606.ENSP00000315757	P06396	GSN	9606.ENSP00000362924	584
P13796	LCP1	9606.ENSP00000315757	P35580	MYH10	9606.ENSP00000269243	417
P06733	ENO1	9606.ENSP00000234590	P49915	GMPS	9606.ENSP00000419851	617
P06733	ENO1	9606.ENSP00000234590	P10809	HSPD1	9606.ENSP00000340019	574
P06733	ENO1	9606.ENSP00000234590	O95757	HSPA4L	9606.ENSP00000296464	502
P06733	ENO1	9606.ENSP00000234590	P09972	ALDOC	9606.ENSP00000226253	912
P06733	ENO1	9606.ENSP00000234590	Q99832	CCT7	9606.ENSP00000258091	554
P06733	ENO1	9606.ENSP00000234590	P12004	PCNA	9606.ENSP00000368438	448
P06733	ENO1	9606.ENSP00000234590	P12277	CKB	9606.ENSP00000299198	409
P06733	ENO1	9606.ENSP00000234590	Q6UB35	MTHFD1L	9606.ENSP00000356290	675
P06733	ENO1	9606.ENSP00000234590	P39023	RPL3	9606.ENSP00000346001	483
P06733	ENO1	9606.ENSP00000234590	Q3SY69	ALDH1L2	9606.ENSP00000258494	406
P06733	ENO1	9606.ENSP00000234590	Q9Y277	VDAC3	9606.ENSP00000428845	400
P06733	ENO1	9606.ENSP00000234590	P36578	RPL4	9606.ENSP00000311430	475
P06733	ENO1	9606.ENSP00000234590	P04179	SOD2	9606.ENSP00000356022	442
P06733	ENO1	9606.ENSP00000234590	P49591	SARS	9606.ENSP00000234677	474
P06733	ENO1	9606.ENSP00000234590	P54577	YARS	9606.ENSP00000362576	673
P10809	HSPD1	9606.ENSP00000340019	Q99832	CCT7	9606.ENSP00000258091	558
P10809	HSPD1	9606.ENSP00000340019	O95757	HSPA4L	9606.ENSP00000296464	762
P10809	HSPD1	9606.ENSP00000340019	P49915	GMPS	9606.ENSP00000419851	757
P10809	HSPD1	9606.ENSP00000340019	P39023	RPL3	9606.ENSP00000346001	467
P10809	HSPD1	9606.ENSP00000340019	P04179	SOD2	9606.ENSP00000356022	419
P10809	HSPD1	9606.ENSP00000340019	P54577	YARS	9606.ENSP00000362576	725
P10809	HSPD1	9606.ENSP00000340019	P49591	SARS	9606.ENSP00000234677	553
P10809	HSPD1	9606.ENSP00000340019	P61981	YWHAG	9606.ENSP00000306330	448
P10809	HSPD1	9606.ENSP00000340019	Q9Y277	VDAC3	9606.ENSP00000428845	748
P10809	HSPD1	9606.ENSP00000340019	P12004	PCNA	9606.ENSP00000368438	537
P10809	HSPD1	9606.ENSP00000340019	Q6UB35	MTHFD1L	9606.ENSP00000356290	485
P10809	HSPD1	9606.ENSP00000340019	Q16539	MAPK14	9606.ENSP00000229794	730
Q99877	HIST1H2BN	9606.ENSP00000380177	P84243	H3F3A	9606.ENSP00000355778	715

P39023	RPL3	9606.ENSP00000346001	Q99832	CCT7	9606.ENSP00000258091	579
P39023	RPL3	9606.ENSP00000346001	Q6UB35	MTHFD1L	9606.ENSP00000356290	731
P39023	RPL3	9606.ENSP00000346001	P61353	RPL27	9606.ENSP00000253788	999
P39023	RPL3	9606.ENSP00000346001	P49591	SARS	9606.ENSP00000234677	518
P39023	RPL3	9606.ENSP00000346001	P49915	GMPS	9606.ENSP00000419851	691
P39023	RPL3	9606.ENSP00000346001	P11940	PABPC1	9606.ENSP00000313007	932
P39023	RPL3	9606.ENSP00000346001	P36578	RPL4	9606.ENSP00000311430	999
P39023	RPL3	9606.ENSP00000346001	P54577	YARS	9606.ENSP00000362576	673
P39023	RPL3	9606.ENSP00000346001	P20290	BTF3	9606.ENSP00000369965	713
Q99832	CCT7	9606.ENSP00000258091	P49915	GMPS	9606.ENSP00000419851	816
Q99832	CCT7	9606.ENSP00000258091	P54577	YARS	9606.ENSP00000362576	722
Q99832	CCT7	9606.ENSP00000258091	P36578	RPL4	9606.ENSP00000311430	574
Q99832	CCT7	9606.ENSP00000258091	P49591	SARS	9606.ENSP00000234677	473
Q99832	CCT7	9606.ENSP00000258091	O95757	HSPA4L	9606.ENSP00000296464	761
Q99832	CCT7	9606.ENSP00000258091	Q6UB35	MTHFD1L	9606.ENSP00000356290	485
Q99832	CCT7	9606.ENSP00000258091	P12004	PCNA	9606.ENSP00000368438	429
P11940	PABPC1	9606.ENSP00000313007	P21281	ATP6V1B2	9606.ENSP00000276390	587
P11940	PABPC1	9606.ENSP00000313007	P31350	RRM2	9606.ENSP00000353770	592
P11940	PABPC1	9606.ENSP00000313007	Q16539	MAPK14	9606.ENSP00000229794	892
P11940	PABPC1	9606.ENSP00000313007	Q13573	SNW1	9606.ENSP00000261531	733
P11940	PABPC1	9606.ENSP00000313007	P61353	RPL27	9606.ENSP00000253788	907
P11940	PABPC1	9606.ENSP00000313007	P61289	PSME3	9606.ENSP00000293362	900
P11940	PABPC1	9606.ENSP00000313007	Q13283	G3BP1	9606.ENSP00000348578	831
P11940	PABPC1	9606.ENSP00000313007	P36578	RPL4	9606.ENSP00000311430	970
P49591	SARS	9606.ENSP00000234677	P29083	GTF2E1	9606.ENSP00000283875	530
P49591	SARS	9606.ENSP00000234677	O95352	ATG7	9606.ENSP00000346437	516
P49591	SARS	9606.ENSP00000234677	P36578	RPL4	9606.ENSP00000311430	457
P49591	SARS	9606.ENSP00000234677	Q6UB35	MTHFD1L	9606.ENSP00000356290	612
P49591	SARS	9606.ENSP00000234677	P12004	PCNA	9606.ENSP00000368438	492
P49591	SARS	9606.ENSP00000234677	P54577	YARS	9606.ENSP00000362576	898
P49591	SARS	9606.ENSP00000234677	P49915	GMPS	9606.ENSP00000419851	791
P49591	SARS	9606.ENSP00000234677	O43776	NARS	9606.ENSP00000256854	873
P04264	KRT1	9606.ENSP00000252244	P35527	KRT9	9606.ENSP00000246662	760
P54577	YARS	9606.ENSP00000362576	Q6UB35	MTHFD1L	9606.ENSP00000356290	566
P54577	YARS	9606.ENSP00000362576	P49915	GMPS	9606.ENSP00000419851	737
P54577	YARS	9606.ENSP00000362576	P36578	RPL4	9606.ENSP00000311430	550
P54577	YARS	9606.ENSP00000362576	O43776	NARS	9606.ENSP00000256854	915
P54577	YARS	9606.ENSP00000362576	P04424	ASL	9606.ENSP00000307188	793
P54577	YARS	9606.ENSP00000362576	O95757	HSPA4L	9606.ENSP00000296464	592
P54577	YARS	9606.ENSP00000362576	Q96KP4	CNDP2	9606.ENSP00000325548	405
P21281	ATP6V1B2	9606.ENSP00000276390	P02786	TFRC	9606.ENSP00000353224	900
P21281	ATP6V1B2	9606.ENSP00000276390	P04179	SOD2	9606.ENSP00000356022	404

P25205	MCM3	9606.ENSP00000229854	O95347	SMC2	9606.ENSP00000286398	551
P25205	MCM3	9606.ENSP00000229854	P31350	RRM2	9606.ENSP00000353770	838
P25205	MCM3	9606.ENSP00000229854	P23258	TUBG1	9606.ENSP00000251413	458
P25205	MCM3	9606.ENSP00000229854	P49736	MCM2	9606.ENSP00000265056	999
P25205	MCM3	9606.ENSP00000229854	P53350	PLK1	9606.ENSP00000300093	951
P25205	MCM3	9606.ENSP00000229854	Q9BTE3	MCMBP	9606.ENSP00000353098	999
P25205	MCM3	9606.ENSP00000229854	Q16576	RBBP7	9606.ENSP00000369424	506
P25205	MCM3	9606.ENSP00000229854	P12004	PCNA	9606.ENSP00000368438	986
P25205	MCM3	9606.ENSP00000229854	P18858	LIG1	9606.ENSP00000263274	805
P36578	RPL4	9606.ENSP00000311430	Q13283	G3BP1	9606.ENSP00000348578	504
P36578	RPL4	9606.ENSP00000311430	P49915	GMPS	9606.ENSP00000419851	770
P36578	RPL4	9606.ENSP00000311430	P20290	BTF3	9606.ENSP00000369965	552
P36578	RPL4	9606.ENSP00000311430	P61353	RPL27	9606.ENSP00000253788	999
P46459	NSF	9606.ENSP00000381293	P02786	TFRC	9606.ENSP00000353224	472
P49915	GMPS	9606.ENSP00000419851	O95757	HSPA4L	9606.ENSP00000296464	628
P49915	GMPS	9606.ENSP00000419851	O43776	NARS	9606.ENSP00000256854	678
P49915	GMPS	9606.ENSP00000419851	Q6UB35	MTHFD1L	9606.ENSP00000356290	877
P49915	GMPS	9606.ENSP00000419851	P04179	SOD2	9606.ENSP00000356022	571
P49915	GMPS	9606.ENSP00000419851	P12004	PCNA	9606.ENSP00000368438	536
P49915	GMPS	9606.ENSP00000419851	P04424	ASL	9606.ENSP00000307188	475
P49915	GMPS	9606.ENSP00000419851	Q96C19	EFHD2	9606.ENSP00000365147	538
P49915	GMPS	9606.ENSP00000419851	Q3SY69	ALDH1L2	9606.ENSP00000258494	457
P49915	GMPS	9606.ENSP00000419851	P31350	RRM2	9606.ENSP00000353770	599
Q6UB35	MTHFD1L	9606.ENSP00000356290	P61353	RPL27	9606.ENSP00000253788	468
Q6UB35	MTHFD1L	9606.ENSP00000356290	Q3SY69	ALDH1L2	9606.ENSP00000258494	758
Q6UB35	MTHFD1L	9606.ENSP00000356290	P30793	GCH1	9606.ENSP00000378890	630
Q6UB35	MTHFD1L	9606.ENSP00000356290	P04179	SOD2	9606.ENSP00000356022	483
Q6UB35	MTHFD1L	9606.ENSP00000356290	O95757	HSPA4L	9606.ENSP00000296464	531
Q6UB35	MTHFD1L	9606.ENSP00000356290	P04424	ASL	9606.ENSP00000307188	534
Q6UB35	MTHFD1L	9606.ENSP00000356290	Q9BWD1	ACAT2	9606.ENSP00000356015	421
P12277	CKB	9606.ENSP00000299198	P61981	YWHAG	9606.ENSP00000306330	479
P12277	CKB	9606.ENSP00000299198	P09972	ALDOC	9606.ENSP00000226253	547
O43776	NARS	9606.ENSP00000256854	Q96KP4	CNDP2	9606.ENSP00000325548	705
P49736	MCM2	9606.ENSP00000265056	P12004	PCNA	9606.ENSP00000368438	959
P49736	MCM2	9606.ENSP00000265056	P31350	RRM2	9606.ENSP00000353770	815
P49736	MCM2	9606.ENSP00000265056	P53350	PLK1	9606.ENSP00000300093	729
P49736	MCM2	9606.ENSP00000265056	P61981	YWHAG	9606.ENSP00000306330	702
P49736	MCM2	9606.ENSP00000265056	Q9BTE3	MCMBP	9606.ENSP00000353098	993
P49736	MCM2	9606.ENSP00000265056	P18858	LIG1	9606.ENSP00000263274	875
P49736	MCM2	9606.ENSP00000265056	O95347	SMC2	9606.ENSP00000286398	643
O95347	SMC2	9606.ENSP00000286398	P12004	PCNA	9606.ENSP00000368438	493
O95347	SMC2	9606.ENSP00000286398	P18858	LIG1	9606.ENSP00000263274	467

O95347	SMC2	9606.ENSP00000286398	Q9BPX3	NCAPG	9606.ENSP00000251496	999
O95347	SMC2	9606.ENSP00000286398	P53350	PLK1	9606.ENSP00000300093	951
O95347	SMC2	9606.ENSP00000286398	Q13112	CHAF1B	9606.ENSP00000315700	555
P26358	DNMT1	9606.ENSP00000352516	Q13185	CBX3	9606.ENSP00000336687	713
P26358	DNMT1	9606.ENSP00000352516	P40763	STAT3	9606.ENSP00000264657	951
P26358	DNMT1	9606.ENSP00000352516	Q16576	RBBP7	9606.ENSP00000369424	932
P26358	DNMT1	9606.ENSP00000352516	P12004	PCNA	9606.ENSP00000368438	999
P61981	YWHAG	9606.ENSP00000306330	Q9NXR1	NDE1	9606.ENSP00000345892	916
P61981	YWHAG	9606.ENSP00000306330	P53350	PLK1	9606.ENSP00000300093	901
P61981	YWHAG	9606.ENSP00000306330	Q07890	SOS2	9606.ENSP00000216373	528
P61981	YWHAG	9606.ENSP00000306330	P35580	MYH10	9606.ENSP00000269243	453
P61981	YWHAG	9606.ENSP00000306330	P23258	TUBG1	9606.ENSP00000251413	913
P08758	ANXA5	9606.ENSP00000296511	P40763	STAT3	9606.ENSP00000264657	416
P08758	ANXA5	9606.ENSP00000296511	Q92597	NDRG1	9606.ENSP00000319977	644
P08758	ANXA5	9606.ENSP00000296511	P31949	S100A11	9606.ENSP00000271638	462
P06396	GSN	9606.ENSP00000362924	P52565	ARHGDIA	9606.ENSP00000269321	416
P06396	GSN	9606.ENSP00000362924	P08567	PLEK	9606.ENSP00000234313	470
Q9BPX3	NCAPG	9606.ENSP00000251496	P53350	PLK1	9606.ENSP00000300093	723
Q9BPX3	NCAPG	9606.ENSP00000251496	P33981	TTK	9606.ENSP00000358813	533
Q9BPX3	NCAPG	9606.ENSP00000251496	P31350	RRM2	9606.ENSP00000353770	434
Q13185	CBX3	9606.ENSP00000336687	P40763	STAT3	9606.ENSP00000264657	489
Q13185	CBX3	9606.ENSP00000336687	Q16576	RBBP7	9606.ENSP00000369424	484
Q13185	CBX3	9606.ENSP00000336687	P02786	TFRC	9606.ENSP00000353224	602
Q13185	CBX3	9606.ENSP00000336687	P84243	H3F3A	9606.ENSP00000355778	859
P15121	AKR1B1	9606.ENSP00000285930	P04179	SOD2	9606.ENSP00000356022	441
P18858	LIG1	9606.ENSP00000263274	P31350	RRM2	9606.ENSP00000353770	652
P18858	LIG1	9606.ENSP00000263274	Q9BTE3	MCMBP	9606.ENSP00000353098	485
P18858	LIG1	9606.ENSP00000263274	P12004	PCNA	9606.ENSP00000368438	999
Q13283	G3BP1	9606.ENSP00000348578	P84243	H3F3A	9606.ENSP00000355778	539
P30044	PRDX5	9606.ENSP00000265462	P04179	SOD2	9606.ENSP00000356022	639
P61289	PSME3	9606.ENSP00000293362	P53350	PLK1	9606.ENSP00000300093	900
P31350	RRM2	9606.ENSP00000353770	P12004	PCNA	9606.ENSP00000368438	987
P31350	RRM2	9606.ENSP00000353770	P53350	PLK1	9606.ENSP00000300093	519
Q9BWD1	ACAT2	9606.ENSP00000356015	O15182	CETN3	9606.ENSP00000283122	474
P61353	RPL27	9606.ENSP00000253788	P20290	BTF3	9606.ENSP00000369965	559
Q13501	SQSTM1	9606.ENSP00000374455	Q16539	MAPK14	9606.ENSP00000229794	851
Q13501	SQSTM1	9606.ENSP00000374455	O95352	ATG7	9606.ENSP00000346437	900
P12004	PCNA	9606.ENSP00000368438	Q13112	CHAF1B	9606.ENSP00000315700	987
P12004	PCNA	9606.ENSP00000368438	Q16576	RBBP7	9606.ENSP00000369424	717
P12004	PCNA	9606.ENSP00000368438	P40763	STAT3	9606.ENSP00000264657	539
P12004	PCNA	9606.ENSP00000368438	P53350	PLK1	9606.ENSP00000300093	624
P12004	PCNA	9606.ENSP00000368438	P23258	TUBG1	9606.ENSP00000251413	459

Q16539	MAPK14	9606.ENSP00000229794	P40763	STAT3	9606.ENSP00000264657	983
Q16539	MAPK14	9606.ENSP00000229794	O15397	IPO8	9606.ENSP00000256079	572
Q16539	MAPK14	9606.ENSP00000229794	Q07890	SOS2	9606.ENSP00000216373	721
Q16539	MAPK14	9606.ENSP00000229794	P84095	RHOG	9606.ENSP00000339467	583
P02786	TFRC	9606.ENSP00000353224	P40763	STAT3	9606.ENSP00000264657	407
P84095	RHOG	9606.ENSP00000339467	P08567	PLEK	9606.ENSP00000234313	503
P84095	RHOG	9606.ENSP00000339467	P52565	ARHGDIA	9606.ENSP00000269321	998
P84095	RHOG	9606.ENSP00000339467	P40763	STAT3	9606.ENSP00000264657	800
P84095	RHOG	9606.ENSP00000339467	Q07890	SOS2	9606.ENSP00000216373	929
P40763	STAT3	9606.ENSP00000264657	O75365	PTP4A3	9606.ENSP00000332274	809
P40763	STAT3	9606.ENSP00000264657	P08567	PLEK	9606.ENSP00000234313	450
P23258	TUBG1	9606.ENSP00000251413	P53350	PLK1	9606.ENSP00000300093	978
P23258	TUBG1	9606.ENSP00000251413	O75347	TBCA	9606.ENSP00000369736	470
P23258	TUBG1	9606.ENSP00000251413	Q9NXR1	NDE1	9606.ENSP00000345892	958
P23258	TUBG1	9606.ENSP00000251413	O15182	CETN3	9606.ENSP00000283122	490
Q9UBB6	NCDN	9606.ENSP00000348394	P04424	ASL	9606.ENSP00000307188	538
P08567	PLEK	9606.ENSP00000234313	P52565	ARHGDIA	9606.ENSP00000269321	471
Q9NXR1	NDE1	9606.ENSP00000345892	P53350	PLK1	9606.ENSP00000300093	907
Q9NXR1	NDE1	9606.ENSP00000345892	Q13123	IK	9606.ENSP00000396301	414
Q16576	RBBP7	9606.ENSP00000369424	P84243	H3F3A	9606.ENSP00000355778	613
Q16576	RBBP7	9606.ENSP00000369424	Q13112	CHAF1B	9606.ENSP00000315700	774
P84243	H3F3A	9606.ENSP00000355778	Q13112	CHAF1B	9606.ENSP00000315700	580
P33981	TTK	9606.ENSP00000358813	P53350	PLK1	9606.ENSP00000300093	655
P33981	TTK	9606.ENSP00000358813	O15182	CETN3	9606.ENSP00000283122	889

Table S9 Primer pairs for qRT-PCR	
β-actin-forward	CGTGCTCAGGGCTTCTTGT
β-actin-reverse	CACCTGGGTCATCTTCTCG
cyclinD1-forward	GCGAGGAACAGAAGTGCG
cyclinD1-reverse	GGAGTTGTCGGTGTAGATG
cyclinE-forward	CCTGGATGTTGACTGCCTTGA
cyclinE-reverse	CGCACCACTGATACCCTGAAA
CDK4-forward	TTTTGAGACCAGGGCTTGC
CDK4-reverse	AGGTGCGTGCCTGTAGTGT
p21-forward	TGTCCGTCAGAACCCATGC
p21-reverse	CTGCCTCCTCCCAACTCAT
Bcl-xL-forward	GTGCGTGGAAAGCGTAGACA
Bcl-xL-reverse	AGAGTGAGCCCAGCAGAACC
Bad-forward	CCCAGAGTTTGAGCCGAGTG
Bad-reverse	ATCCCTTCGTCGTCCTCCGT
Smac-forward	GCAGCGTAACTTCATTCTTC
Smac-reverse	TTACTCCAAAGCCAATCGTCA
XIAP-forward	GGCAGATTATGAAGCACGGAT
XIAP-reverse	CCCTCCTCCACAGTGAAAGC
Caspase-3-forward	ATGGACCTGTTGACCTGA
Caspase-3-reverse	ACCAGGTGCTGTGGAGTA
Histone H3.1-forward	ATGACGACGATAAGGGAGGA
Histone H3.1-reverse	CTCGGTCGACTTCTGGTAGC
Cytoskeletal 9-forward	TTGGCTACAGCTACGGCGGAGGAT
Cytoskeletal 9-reverse	TGAGTCATCAATAGTGTTATAAT
Vitronectin-forward	GGCTGTCCTTGTTCTCCAGTG
Vitronectin-reverse	GTGCGAAGATTGACTCGGTAGT

Listed primers are in the 5’ to 3’ orientation.

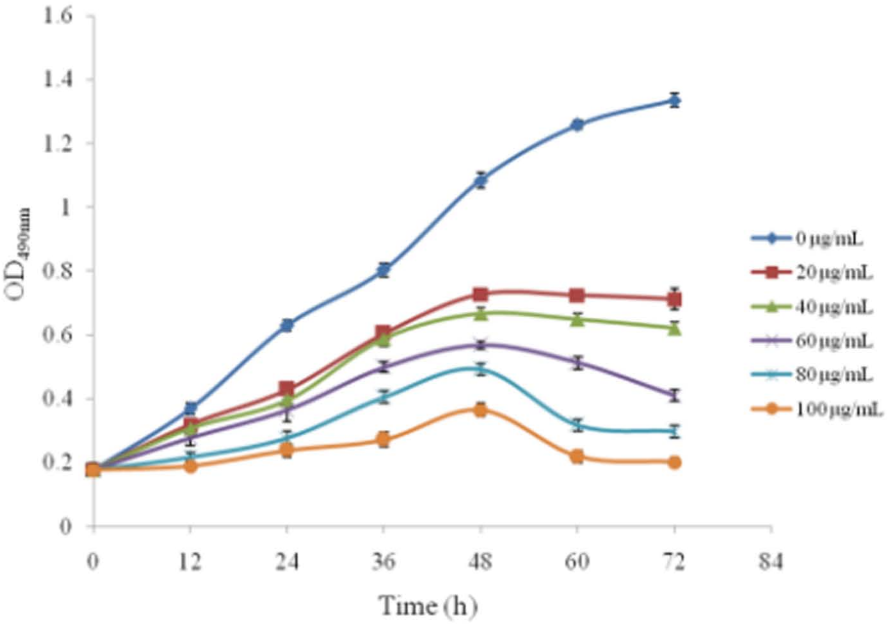

Figure S1 The growth of normal and VCP2-treated HepG2 cells

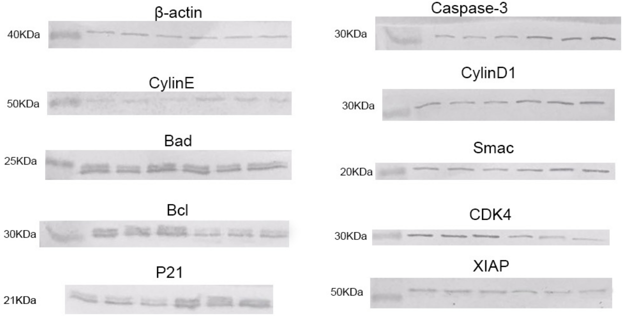

Figure S2 The protein expression in polysaccharides-treated HepG2 cells by WB assay

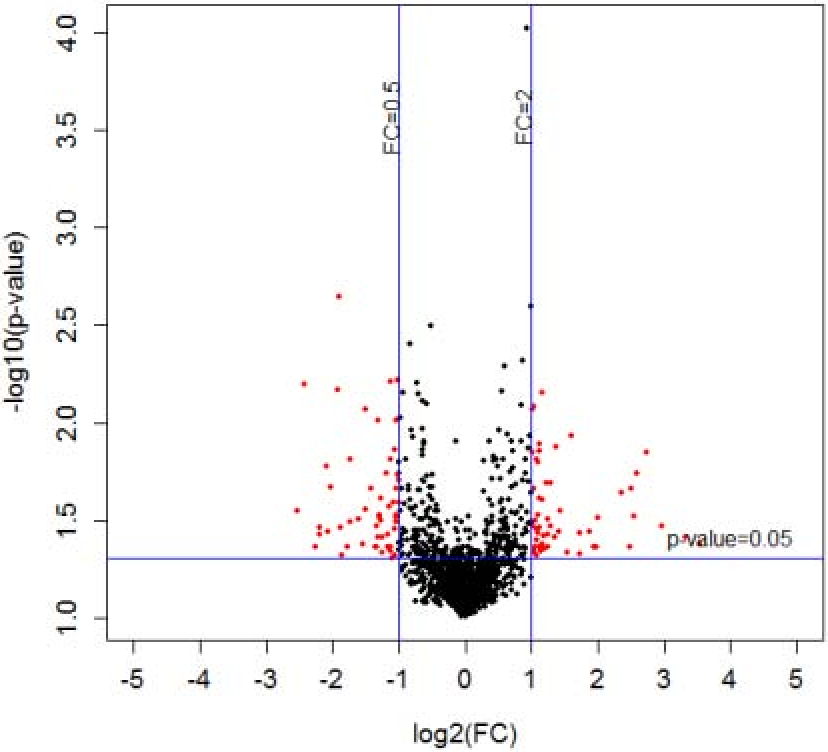

Figure S3 A volcano plot of identified proteins in normal and VCP2-treated HepG2 cells

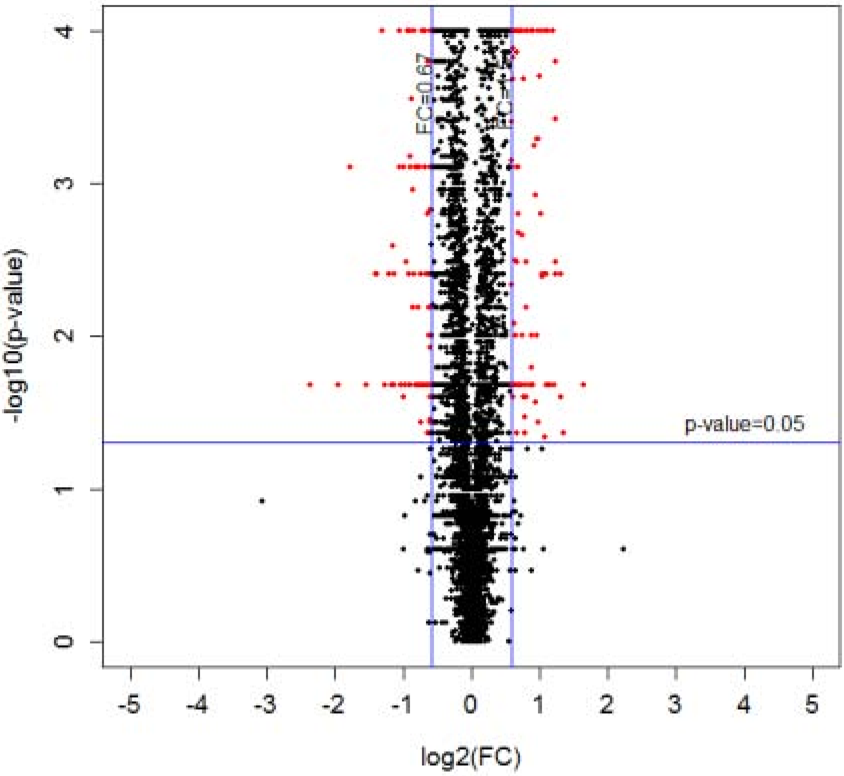

Figure S4 A volcano plot of identified proteins in normal and VCP2-treated Caco2 cells

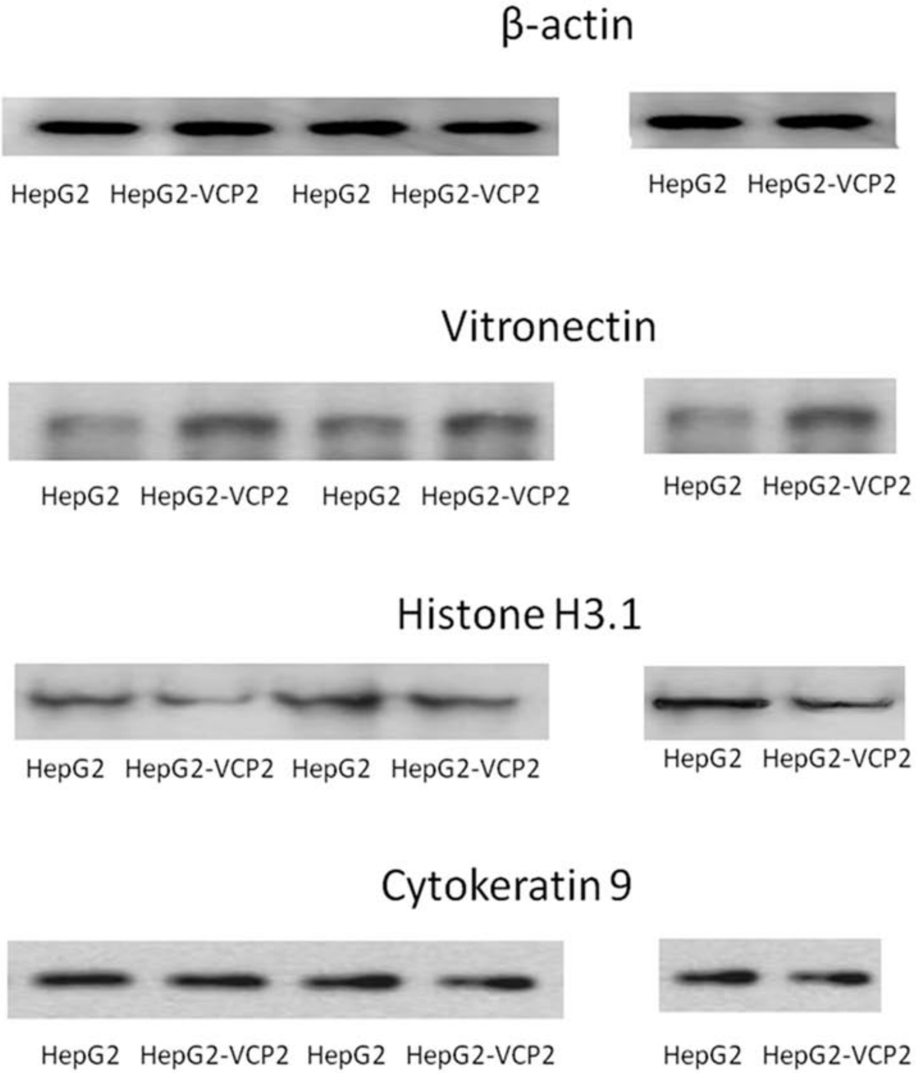

Figure S5 The protein expression validation by WB assay
